# Role of histone modifications and early termination in pervasive transcription and antisense-mediated gene silencing in yeast

**DOI:** 10.1093/nar/gku100

**Published:** 2014-01-31

**Authors:** Manuele Castelnuovo, Judith B. Zaugg, Elisa Guffanti, Andrea Maffioletti, Jurgi Camblong, Zhenyu Xu, Sandra Clauder-Münster, Lars M. Steinmetz, Nicholas M. Luscombe, Françoise Stutz

**Affiliations:** ^1^Department of Cell Biology and NCCR “Frontiers in Genetics”, iGE3, University of Geneva, 1211 Geneva, Switzerland, ^2^EBI-EMBL Hinxton, Cambridge CB101SD, England, ^3^European Molecular Biology Laboratory, 69117 Heidelberg, Germany, ^4^Department of Genetics, Stanford University, Stanford, CA 94395 USA and ^5^Stanford Genome Technology Center, Palo Alto, CA 94303, USA

## Abstract

Most genomes, including yeast *Saccharomyces cerevisiae*, are pervasively transcribed producing numerous non-coding RNAs, many of which are unstable and eliminated by nuclear or cytoplasmic surveillance pathways. We previously showed that accumulation of *PHO84* antisense RNA (asRNA), in cells lacking the nuclear exosome component Rrp6, is paralleled by repression of sense transcription in a process dependent on the Hda1 histone deacetylase (HDAC) and the H3K4 histone methyl transferase Set1. Here we investigate this process genome-wide and measure the whole transcriptome of various histone modification mutants in a *Δrrp6* strain using tiling arrays. We confirm widespread occurrence of potentially antisense-dependent gene regulation and identify three functionally distinct classes of genes that accumulate asRNAs in the absence of Rrp6. These classes differ in whether the genes are silenced by the asRNA and whether the silencing is HDACs and histone methyl transferase-dependent. Among the distinguishing features of asRNAs with regulatory potential, we identify weak early termination by Nrd1/Nab3/Sen1, extension of the asRNA into the open reading frame promoter and dependence of the silencing capacity on Set1 and the HDACs Hda1 and Rpd3 particularly at promoters undergoing extensive chromatin remodelling. Finally, depending on the efficiency of Nrd1/Nab3/Sen1 early termination, asRNA levels are modulated and their capability of silencing is changed.

## INTRODUCTION

The development of high-density tiling arrays and large-scale RNA sequencing approaches revealed that all eukaryotic genomes are pervasively transcribed and synthesize a myriad of non-coding RNAs (ncRNAs). The yeast *Saccharomyces cerevisiae* produces a large number of long ncRNAs between 200 up to a few thousand bases in length, comprising both intergenic RNAs and transcripts antisense to coding open reading frames (ORFs). Antisense transcription can be regulated in a pre-initiation complex-dependent manner independently of divergent sense transcription of a downstream gene (1). Antisense transcripts (asRNAs) have been divided into stable unannotated transcripts (SUTs) detected in wild-type (WT) cells and cryptic unstable transcripts (CUTs), which are rapidly degraded and detectable only in mutants of the nuclear exosome (2,3). The degradation of CUTs by the 3′–5′ exonuclease Rrp6 is tightly linked to transcription termination by the Nrd1/Nab3/Sen1 (NNS) complex and polyadenylation by the TRAMP complex (4–6). NNS is recruited at the 5′ end of transcription units by RNA polymerase II (PolII) phosphorylated on serine 5 and induces termination primarily of <1-kb-long ncRNAs on recognition by Nrd1 and Nab3 of specific sequence motifs on the nascent transcripts (7–12). Recent global studies showed that Nrd1 and Nab3 have a widespread localization and preferentially bind divergent and antisense ncRNAs playing a role in surveillance through early termination of transcripts that originate from bidirectional promoters or from the 3′ end of ORFs (13–15).

Growing evidence suggests that pervasive transcription, and in particular antisense transcription, contributes to the regulation of gene expression. Large-scale analyses of sense/antisense pairs, primarily considering SUTs, revealed anti-correlated expression patterns and antisense-dependent fine-tuning of sense expression (16). Gene-specific studies indicate that ncRNA transcription can modulate gene expression by mechanisms of transcription interference, nucleosome remodelling or via changes in histone modifications (17). Examples include the upstream ncRNA *SRG1,* which interferes with transcription of the downstream *SER3* gene by enhancing nucleosome assembly over the promoter (18–20), or the *RME2* asRNA, proposed to interfere with transcription elongation of the *IME4* gene (21,22). H3K4 dimethylation deposited by the H3K4 histone methyl transferase (HMT) Set1 during antisense or upstream non-coding transcription has also been involved in gene repression by signalling the recruitment of the Rpd3 or Set3 histone deacetylase (HDAC) complexes (23–27), and genes repressed by Set1 are enriched in antisense-producing ORFs (28).

*PHO84* is another gene regulated by an antisense transcript. Our earlier studies indicated that the accumulation of *PHO84* asRNA in the absence of the exosome component Rrp6 is paralleled by repression of sense transcription through the recruitment of the Hda1/2/3 HDAC complex and targeted histone deacetylation at the *PHO84* promoter and 5′ end (29). We also showed that Set1 promotes antisense production and contributes to *PHO84* gene repression (30). More recent single molecule fluorescence *in situ* hybridization (FISH) studies from our laboratory indicate that the expression of *PHO84* sense and asRNAs is anti-correlated in individual cells. We also provided evidence that *PHO84* asRNA early termination by Nrd1 is reduced in *Δrrp6* and that asRNAs escaping this early termination do not accumulate in the nucleus but are rapidly exported. The data suggest that *PHO84* repression depends, at least in part, on constant low-frequency antisense transcription through the promoter, which is increased in *Δrrp6*, reinforcing the tight on–off switch of this highly regulated gene (31).

Although regulation by antisense transcription appears as a common mechanism (16), its molecular basis has not been examined globally. In this study, we aimed at characterizing the mechanistic diversity of antisense-mediated repression. To do so, we investigated the role of chromatin modifications in gene repression following asRNA accumulation in *Δrrp6*. We used high-density tiling arrays to examine the contribution of the HMT Set1 or the HDACs Hda1 and Rpd3 in asRNA production and silencing on a genome-wide level. Our data identify three classes of antisense transcripts: two that are associated with gene repression and differ on whether the silencing mechanism involves histone-modifying activities and one class that does not affect sense transcription. We show that the repressive effect of antisense transcription on sense expression is linked to the efficiency of asRNA early termination by NNS.

## MATERIALS AND METHODS

The yeast strains and sequences of all the primers used in this study are listed in Supplementary Table S1.

### Sample preparations and analyses

#### RNA preparation and tiling arrays

Total RNA was treated with RNase-free DNaseI using Turbo DNA-free kit (Ambion). For first-strand complementary DNA (cDNA) synthesis, 20 μg of total RNA was mixed with 1.72 μg of random hexamers, 0.034 µg of oligo(dT) primer and incubated at 70°C for 10 min followed by 10 min at 25°C and then transferred on ice. The synthesis included 2000U of SuperScript II Reverse Transcriptase, 50 mM Tris-HCl, 75 mM KCl, 3 mM MgCl2, 0.01 M dithiothreitol, dNTP + dUTP mix (0.5 mM for dCTP, dATP and dGTP; 0.4 mM for deoxythymidine triphosphate and 0.1 mM for dUTP, Invitrogen), 20 μg/ml actinomycin D in a total volume of 105 μl. The reaction was carried out in 0.2-ml tubes in a thermal cycler with the following thermal profile: 25°C for 10 min, 37°C for 30 min, 42°C for 30 min followed by 10 min at 70° for heat inactivation and 4°C on hold. Samples were then subjected to RNase treatment of 20 min at 37°C (30U of RNase H, Epicentre, 60U of RNase Cocktail, Ambion). First-strand cDNA was purified using the MinElute PCR purification kit (Qiagen), and 5 µg was fragmented and labelled using the GeneChip WT Terminal labelling kit (Affymetrix) according to the manufacturer’s protocol. The labelled cDNA samples were denatured in a volume of 300 µl containing 50 pM control oligonucleotide B2 (Affymetrix) and Hybridization mix (GeneChip Hybridization, Wash and Stain kit, Affymetrix) of which 250 µl was hybridized per array (*S. cerevisiae* yeast tiling array, Affymetrix, PN 520055). Hybridizations were carried out at 45°C for 16 h with 60 rpm rotation. The staining was carried out using the GeneChip Hybridization, Wash and Stain kit with fluidics protocol FS450_0001 in an Affymetrix Fluidics station.

#### RNA analyses by real-time quantitative polymerase chain reaction

Total RNA was prepared as described (29). For real-time quantitative polymerase chain reaction (RT-qPCR) quantifications, 1 µg of cDNAs of sense or antisense RNAs (asRNAs) was generated by SuperScript II reverse transcriptase (Invitrogen) from total RNAs using strand-specific DNA primers. cDNAs were quantified by RT-qPCR (BioRad). The same amplicon was used to quantify sense and antisense cDNA.

#### Chromatin immunoprecipitation

Chromatin immunoprecipitations (ChIPs) were performed essentially as described previously (29). Yeast strains were grown to OD600 = 0.8 either in yeast extract-peptone-dextrose (YEPD) growth media at 25°C and cross-linked for 10 min by the addition of formaldehyde to a final concentration of 1.2%. Cross-linked and sonicated chromatin extracts from 1.5 mg of Bradford-quantified proteins were immunoprecipitated overnight in the presence of protein G Sepharose (Amersham Pharmacia) with antibodies against H3K4me3 (Abcam 8580) or H3 (Abcam 1791). All immunoprecipitations were repeated at least three times with different chromatin extracts. Immunoprecipitated DNA was purified and quantified by RT-qPCR with primers listed in Supplementary Table S1 and expressed as the percentage of input DNA normalized to H3. Error bars correspond to standard deviations.

#### Half-life measurements

Cells were grown to an OD_600_ = 0.8 in YEPD medium. At T = 0, 100 µg/ml 1,10-phenanthroline (Sigma) was added to the culture as described (32), and samples were collected at different time points and analysed by RT-qPCR. Half-lives were calculated by the equation t_1/2_ = 0.693/k, where k is the rate constant for messenger RNA (mRNA) decay. Values of each time point are normalized for internal variations with *SCR1* RNA, a control that is still stable at the 30-min time point.

#### Anchor away strains

The anchor away strains (Rrp6-AA and Nrd1-AA) were constructed by tagging the endogenous gene of interest at the carboxyl terminus by PCR amplification of the pFA6a-FRB-KanMX6 cassette (see Supplementary Table S1 for primer sequences) and transformation of the PCR product into the parental rapamycin-insensitive strain HHY168 (33). Cells were grown to an OD_600_ = 0.8 in YEPD medium, treated with 1 µg/ml rapamycin (LC laboratories) and collected at different time points. RNA was extracted and analysed by RT-qPCR (or northern blotting). Values of each time point are normalized to *ACT1* mRNA, a control not affected by rapamycin treatment.

### Bioinformatics analyses

#### Gene classification

Our goal was to classify genes according to whether they are regulated by antisense transcription and/or chromatin modification enzymes. To capture the effect of antisense transcription on the sense expression simultaneously for all histone modification mutants, we decided to use an unsupervised clustering approach in which we classify genes based on the combined effect of all mutants on the genes sense and antisense expression levels. For this, we calculated differential expression values in *Δrrp6* versus WT, *Δset1Δrrp6* versus *Δrrp6*, *Δhda2Δrrp6* versus *Δrrp6* and *Δrpd3Δrrp6* versus *Δrrp6* for the sense transcript from −100 to +750 bp of the transcription start site (TSS) and for the antisense transcript −100 to +750 bp of the transcription termination site (TTS) on the antisense strand in bins of 20 bp. We then concatenated these values for each gene and performed pam (partitioning around medoids) clustering (34) using 15 clusters (Supplementary Figure S1). To ensure the robustness of the clustering, we performed 120 additional clusterings with 10–15 clusters (20 for each) randomly selecting 80% of all genes and measured how often each gene is paired with any other gene of the same cluster. Looking at the distributions, we selected a cut-off of 60% to identify the core set of genes for each cluster (Supplementary Figure S1). We then inspected the median profiles of the 15 clusters and grouped them into five classes based on their expression profiles across the different conditions as follows: Class (I): cluster 1; Class (II): cluster 2; Class (III): clusters 6, 8 and 12; Class (IV): cluster 5; and unclassified control set: clusters 3, 4, 7, 9, 10, 11, 13, 14 and 15 (Supplementary Figure S1). The classes are described in Figure 2 and the corresponding genes listed in Supplementary Table S2. We focused here on characteristics that were interesting in the context of this study. Therefore, we did not consider clusters that showed elevated expression levels of antisense transcripts only in some of the double mutants but not in the *Δrrp6* versus WT separately (e.g. elevated antisense in *Δrpd3Δrrp6* versus *Δrrp6* in clusters 9 and 14) (see Supplementary Figure S1). Tiling array heatmaps can be viewed at http://steinmetzlab.embl.de//cgi-bin/viewStutzLabArray.pl?showSamples=stutzLabArray&type=heatmap&gene=rrp6.

The complete tiling array data have been submitted to Array Express (at http://www.ebi.ac.uk/arrayexpress/, accession number E-MTAB-1316).

#### Expression levels of antisense RNA

To calculate the mean expression profile of the antisense transcripts for the different classes, we obtained the expression level in *Δrrp6* on the antisense strand and took the median across the genes in a given class for each bin of 10 bp. We then aligned this matrix once to the ORFs TSS and once to its TTS and calculated a moving average with a window size of 150 bp and a step size of 10 bp for each class across all genomic positions.

#### Promoter structure, Nrd1/Nab3 motifs and Nrd1 binding

To analyse the promoter structure, we used the definition of TATA boxes from Basehoar (35) and the open–closed promoter configuration from Zaugg (36). TF binding sites were taken from MacIsaac (37).

To analyse the occurrence of Nab3 and Nrd1 motifs in antisense transcripts, we used the consensus sequences TCTT and GTA[AG] for Nab3 and Nrd1, respectively (12). For each gene, we then counted the number of motifs within 400–0 bp upstream of the genes TTS on the antisense strand. For analysing Nrd1 binding events, we obtained PAR-CLiP data from the Corden laboratory (13). We summed the binding data within 400–0 bp upstream of the genes TTS on the antisense strand and normalized it to the WT expression level of this region. Using expression level in *Δrrp6* for normalization did not change the results.

#### Identification of the whole population of ncRNAs

To identify specific ncRNA transcripts affected by Set1 (Supplementary Figure S5) we performed an automatic segmentation of the tiling array data in the Δ*rrp6* mutant (38). We then overlapped the expressed segments with annotated transcripts identified by Xu *et al.* (3). Transcripts that did not overlap with the Xu *et al.* data and were at least 200 bases long were annotated as ncRNAs (Supplementary Table S3). We redefined CUTs and SUTs such that CUTs are at least 2-fold upregulated in *Δrrp6* versus WT (Supplementary Table S3 and Supplementary Figures S7A and S7B). We found more ncRNAs than Xu *et al.* (2009), likely because we used total RNA instead of poly(A)-enriched RNA and performed the experiment in a different strain background (W303 instead of S96/S288c). This annotation has been used for the analyses in Supplementary Figures S5 and S7.

#### Comparison of H3K4me3 levels in Δset1-silenced versus not affected genes

We grouped the ORF transcripts as well as the ncRNA transcripts into four quartiles, each based on their expression level in *Δrrp6*. Transcripts were then separated into ‘Δset1-silenced’ and ‘not affected’ based on their differential expression value in *Δset1Δrrp6* versus *Δrrp6*, with thresholds of 2-fold over- or under-expressed (Supplementary Figure S5D). H3K4me3 in their promoter region (−100 to +300 bp of the TSS) was measured using data from (39).

## RESULTS

### A clustering approach identifies different classes of genes potentially regulated by asRNAs

To identify genes regulated by antisense ncRNAs accumulating in the absence of Rrp6 and to characterize the mechanisms by which antisense transcription affects gene expression, we performed tiling array expression profiling of strains lacking Rrp6 alone or in combination with disruptions of histone modification enzymes previously implicated in antisense-mediated regulation of *PHO84*: the H3K4 methyl transferase Set1 and the Hda1/2/3 HDAC complex subunit Hda2. In addition, we selected the HDAC Rpd3, which has been involved in antisense-mediated regulation of other genes (23,24). Total RNA prepared from single and double mutant strains in triplicate was hybridized to strand-specific high-resolution tiling arrays covering the *S. cerevisiae* genome as described (3,38).

**Figure 1.**
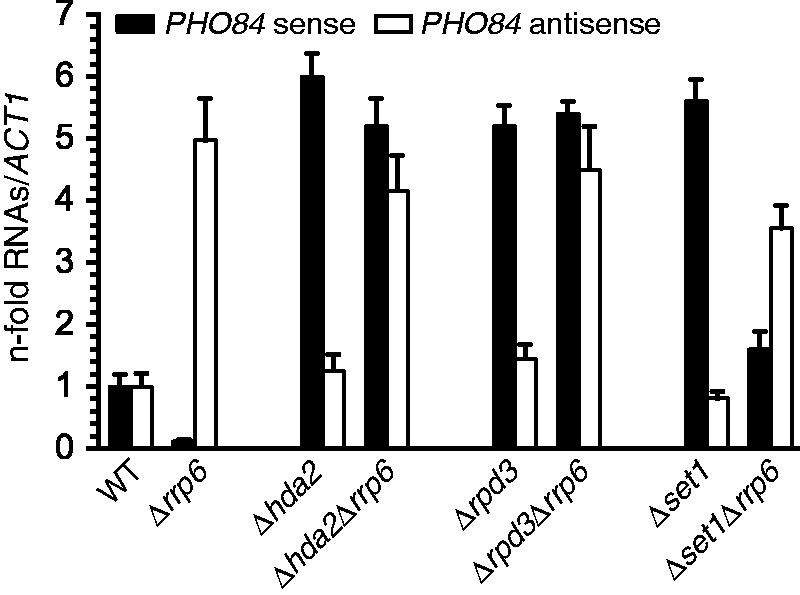
Analysis of *PHO84* sense and antisense transcripts in different chromatin-modifying mutants. *PHO84* sense and asRNA levels were quantified by RT-qPCR in the indicated strains as described (29,30). Sense and antisense values were normalized to *ACT1* and expressed as fold change over WT. Error bars reflect standard deviations of an average obtained from three independent experiments.

We first validated the mutant phenotypes by examining the behaviour of our model gene *PHO84* by quantitative RT-PCR (Figure 1). We confirmed *PHO84* repression in response to antisense accumulation in *Δrrp6* and its de-repression on loss of Hda2, a component of the Hda1 complex. In addition, we found a similar de-repression of the gene on loss of the HDAC Rpd3 in *Δrrp6*, suggesting that the Hda1 and Rpd3 HDACs play partially overlapping roles in antisense-mediated silencing. Notably, the increase of *PHO84* sense RNA levels in *Δhda2* or *Δrpd3* single mutants indicates a repressive effect of these HDACs in WT cells. This repression could be linked to the low levels of *PHO84* antisense expression in WT cells (29,31). Finally, we confirmed that loss of the HMT Set1 reduces antisense levels and *PHO84* repression in *Δrrp6*, supporting a role of Set1 in silencing by acting upstream of asRNA, either by enhancing its production or stability.

To study the potential silencing mechanisms mediated by Rrp6-sensitive asRNAs genome-wide, we used a classification approach that integrates a gene’s response to various chromatin mutants simultaneously for its sense and antisense transcripts. Briefly, we used strand-specific tiling array data to compute differential expression in sense and antisense directions for *Δrrp6* versus *WT, Δrrp6Δset1* versus *Δrrp6, Δrrp6Δhda2* versus *Δrrp6* and *Δrrp6Δrpd3* versus *Δrrp6*, and classified genes based on the behaviour of their sense and antisense transcripts in those conditions. This integrative approach allowed us to detect asRNAs whose differential expression falls below the detection threshold in individual conditions, as long as they show differential expression above 0 in any of the other mutant strains. The differential expression of sense mRNAs was calculated between −100 and +750 bp around the ORF TSS, whereas the differential expression of asRNAs was calculated on the opposite strand between −100 and +750 bp around the ORF TTS. We then applied unsupervised clustering using the concatenated differential expression values as features to group genes with similar sense/antisense behaviours across the different strains (see ‘Materials and Methods’ section, Supplementary Figure S1). A total of 1715 genes in convergent configuration that overlap each other were excluded from the analysis. To further corroborate the clustering, we performed a robustness analysis and selected only the core set of genes that co-segregated in multiple randomized rounds of clustering, strengthening the assessment of their distinctive features (Supplementary Figure S2A).

**Figure 2.**
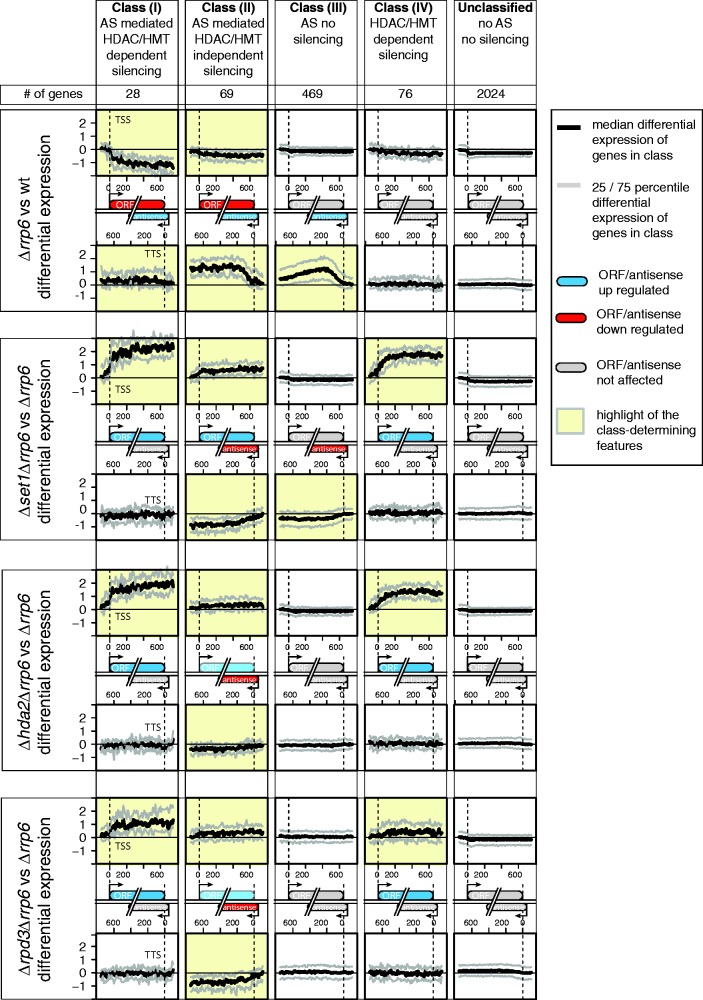
Gene classification based on the silencing mechanism associated with Rrp6-sensitive asRNAs. Genes were clustered into 15 groups based on differential expression of the sense and antisense strands in four conditions: *Δrrp6* versus WT, *Δset1 Δrrp6* versus *Δrrp6*, *Δhda2Δrrp6* versus *Δrrp6* and *Δrpd3Δrrp6* versus *Δrrp6* (see Materials and Methods’ section, Supplementary Figures S1 and S2). The clusters were grouped into four Classes, (I), (II), (III) and (IV), based on the similarity of their median profiles for the different mutants and an unclassified control set (Unclassified). The number of genes in each class is given at the top. The distinguishing features for each class are highlighted in yellow and represented by little schematics where red indicates up- and blue indicates downregulation in a given mutant. Genes where silencing involves Hda1, Rpd3 and Set1 are described as ‘HDAC/HMT-dependent silencing’.

These clusters were then grouped into four classes and a group of unclassified genes based on a combination of three criteria: first whether they exhibit asRNA accumulation in *Δrrp6*, second whether the gene (ORF) itself is repressed in *Δrrp6* and finally whether the *Δrrp6-*dependent repression of the gene is relieved on the loss of histone modification enzymes (Figure 2 and Supplementary Table S2). A class was defined as accumulating or silencing transcripts in a given strain if the median differential expression of the class was above or below 0, respectively. Class (I) contains genes that are repressed and accumulate asRNA in *Δrrp6*, and de-repressed on the loss of Hda2, Rpd3 or the HMT Set1. The asRNAs in this class are essentially not influenced by these HDACs and modestly affected by the HMT Set1. Therefore, this class represents genes potentially regulated by an antisense-mediated mechanism similar to *PHO84*. Genes in Class (II) also accumulate asRNA and are repressed in *Δrrp6*. However, unlike Class (I) genes, the loss of Set1, Hda2 and Rpd3 only marginally affects ORF expression, indicating that potential antisense-mediated silencing is largely independent of these HDACs and the Set1 HMT. Class (III) genes also show increased antisense accumulation in *Δrrp6* but no effect on ORF transcription. Notably, genes in both Classes (II) and (III) show substantial decrease of antisense transcripts in the absence of Set1, rendering these classes of genes the most dependent on Set1 for antisense production in *Δrrp6*. Class (IV) contains genes that are repressed by Set1 and the two HDACs. These genes are related to Class (I) (see below); however, they segregate as a separate group, as they show either no clear asRNA signal within 750 bp upstream of the TTS (in some cases, asRNAs start close to the 5′ end of the gene, e.g. *PDR15*), or asRNA levels that only modestly change in *Δrrp6* (similar to SUTs) and are not paralleled by changes in sense RNA levels (e.g. *ATO2*), or sense transcription is mostly repressed under these experimental conditions (e.g. *CRF1*, *YTP1*). Finally, the remaining genes were labelled as ‘unclassified’ and used as a control set throughout the study. Notably, single mutant versus WT comparisons of the different gene classes, in particular Class (I), do not show substantial increases in ORF expression, supporting the view that the repressive effects of Set1, Hda2 and Rpd3 on these genes in *Δrrp6* are linked to the presence of asRNA (Supplementary Figure S2B). Representative expression patterns of genes from Classes (I) to (III) are shown in Supplementary Figure S3.

In summary, we found ∼100 genes [Class (I) and (II)] that are silenced concomitant to asRNA accumulation in *Δrrp6*. While the proposed antisense-mediated repression of Class (I) (28 genes) depends on the Hda1/Rpd3 HDACs and the Set1 HMT, silencing of Class (II) (69 genes) appears to involve a different mechanism possibly based on transcription interference (see below). However, we cannot exclude that the slight decrease in a fraction of Class (I) and (II) asRNA levels observed in *Δrrp6* in the absence of Set1, Rpd3 or Hda2 is due to increased sense transcription in these mutants or that the chromatin modifiers independently affect the two transcription units. Class (III) contains 469 genes that accumulate asRNAs in *Δrrp6* with no effect on gene expression. In addition, Class (IV) contains 76 genes that are repressed by the two HDACs and the HMT already in WT cells. Many of them produce low, mostly Rrp6-insensitive levels of asRNAs, making them distinct from, yet related to, Class (I). Finally, 2024 genes that have essentially no asRNA and are affected in none of the mutants remained unclassified.

### Functional and non-functional asRNAs show distinct features

**Figure 3.**
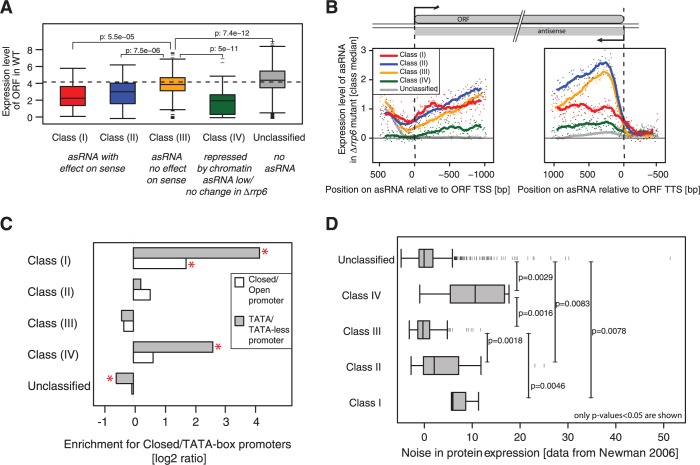
Characterization of the gene classes. (**A**) Genes silenced by asRNA show low expression. Expression levels in WT are shown for the different gene classes. Classes regulated by Rrp6-sensitive asRNA [(I), red, (II), blue] show lower expression levels than genes that have a non-functional or no asRNA [Class (III) yellow and grey for Unclassified]. Genes repressed by the Hda1/Rpd3 HDACs and the Set1 HMT [Class (IV), green] in the absence of an Rrp6-sensitive asRNA are even less expressed, consistent with their repressed state in normal WT condition. *P*-values are given for the Wilcoxon rank sum test. (**B**) asRNA expression levels differ across the gene classes. Expression levels on the antisense strand are shown for the different classes. Median expression levels across each class were calculated using bins of 10 nt (dots) aligned to the TSS (left) and the TTS (right) of the sense ORF and used a moving average (150-nt window) to smooth the profile (solid lines). (**C**) Class (I) has a specific promoter structure. Genes in Class (I) are enriched for TATA box promoters and a closed promoter structure, and genes in Class (IV) are enriched only in TATA box promoters. Enrichments were calculated using Fisher’s exact test. Enrichments where *P* < 0.05 are indicated with a star. (**D**) Gene classes and transcription noise. The distribution of protein expression noise is shown for gene Classes (I) to (V). The noise data were taken from Newman (40).

To gain further insights into antisense-mediated regulatory mechanisms, we investigated general functional features of the sense/antisense pairs in each class. We found that genes regulated by Rrp6-sensitive asRNA [Classes (I) and (II)] have significantly lower basal expression levels in WT compared with those that are not [Class (III)] (Figure 3A). This is in agreement with previous studies reporting that genes affected by antisense SUTs tend to be expressed at medium to low levels (16). Interestingly, Class (IV) genes, which are de-repressed in the HDAC/HMT knockouts, are lowly expressed, consistent with a generally repressed state in current growth conditions. Accordingly, promoters of genes in this class are enriched for binding sites of repressive transcription factors (TFs), such as glucose repression (*MIG1*) and meiosis (*UME6*, *IME1*), but also of several activators (*ADR1*, *MSN2*, *MSN4*, *SPT15*/*TBP* and *STP1*), indicating extensive regulation (Supplementary Table S4 and see below).

To further define the features of antisense transcripts that affect the corresponding sense gene (termed ‘functional asRNAs’), we compared the extent to which they overlap with the sense gene as well as their expression levels with asRNAs that have no effect on sense transcription (termed ‘non-functional asRNAs’). For this, we calculated the average expression in *Δrrp6* along the antisense strand separately for each class. We aligned the expression values once to the TTS of the corresponding sense ORF and once to its TSS (Figure 3B). Interestingly, despite their relatively low expression level, asRNAs in Class (I) extend across the whole length of the gene and even beyond the TSS. In contrast, the expression of Class (III) antisense transcripts, which have no effect on sense expression, shows a strong peak close to the TTS but then drops rapidly, indicating that these transcripts do not extend much into the genes. Class (II) antisense transcripts exhibit an intermediate pattern, as they show an initial peak like in Class (III), but decrease more slowly and appear to extend beyond the TSS, consistent with an effect of these asRNAs on ORF expression (Figure 3B). The asRNA profile for Class (IV) genes shows a similar pattern to Class (I) genes but at a much lower level, while no asRNA was detected over the unclassified control set. Comparing the length of the genes/ORFs across the different classes revealed no specific bias that could explain the difference in the extent of antisense overlap (data not shown).

To follow up on the finding that Class (I) and (II) asRNAs tend to extend into the promoters of their counterpart genes, we examined whether there are any differences in promoter structures, such as TATA boxes or promoter-nucleosome architecture across the five classes. TATA box genes mainly comprise inducible or stress-response genes (35). Closed promoters, characterized by a well-positioned +1 and a fuzzy −1 nucleosome that often occlude important TF binding sites, have been associated with a complex regulatory structure and high variation of expression levels across a population of cells (36). We found that Class (I) is the only class enriched for closed promoters and both Class (I) and Class (IV) are enriched for TATA-box promoters (Figure 3C). Consistently, they also exhibit significantly higher expression noise (40) compared with the other gene classes (Figure 3D).

In summary, our data suggest that the ability of asRNAs to participate in gene repression resides in the extent to which the ncRNA transcription progresses along the gene as well as in the corresponding promoter structure. The enrichment of Class (I) genes for closed promoter configurations suggests that silencing could involve antisense-induced nucleosome repositioning in the sense promoter, whereas another mechanism, less dependent on chromatin remodelling, may be operating at Class (II) genes.

### Set1 is involved in asRNA production

Our clustering analysis revealed that a large fraction of the asRNAs that accumulate in *Δrrp6*, especially those of Class (II) and Class (III), tend to be downregulated on deletion of the HMT Set1 (Figure 2, *Δset1Δrrp6* versus *Δrrp6*). Set1 is the catalytic subunit of the COMPASS complex devoted to the differential methylation of lysine 4 on H3 in *S. cerevisiae*. H3K4 trimethylation by Set1 is enriched at the TSS of transcribed ORFs, while di- and monomethylated H3K4 peak in the middle and 3′ end of the gene, respectively (39,41).

**Figure 4.**
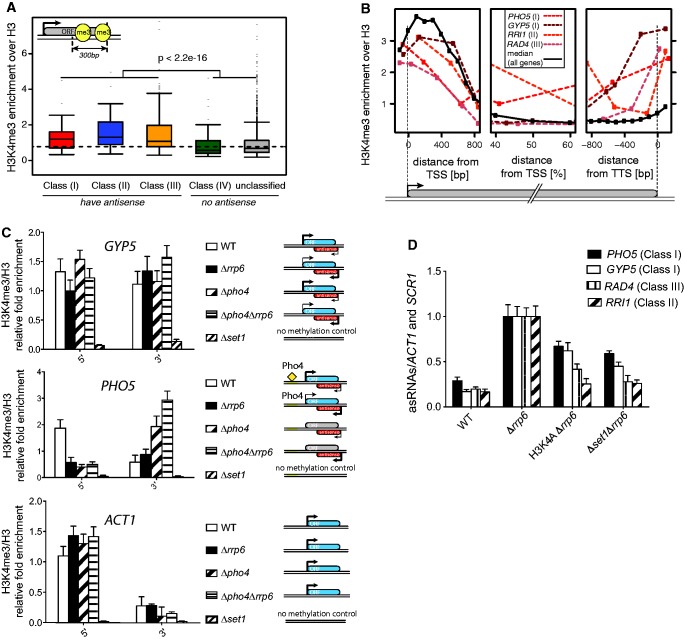
H3K4me3 enrichment at 3′ ends of genes with asRNA. (**A**) Genes with antisense have higher levels of H3K4me3 at their 3′ ends. We calculated the average H3K4me3 enrichment over H3 (39) between 0 and −300 bp from the TTS and plotted the distribution for the five gene classes. Genes that have an antisense transcript [Classes (I), (II), and (III)] show higher H3K4me3 than genes with low or no antisense [Class (IV) and Unclassified]. (**B**) H3K4me3 profiles at single genes. H3K4me3 profiles are plotted for specific genes [*PHO5* and *GYP5* for Class (I), *RRI1* for Class (II) and *RAD4* for Class (III)], once aligned to the gene’s TSS (left), and once to its TTS (right). The average profile (dashed line) is calculated over all genes >1500 bp with at least 200 bp distance to the next downstream gene. (**C**) ChIP analysis of H3K4 trimethylation pattern at antisense-producing genes. ChIP extracts prepared from WT, *Δrrp6*, *Δpho4*, *Δpho4Δrrp6* and *Δset1* strains grown in YEPD were immunoprecipitated with anti-H3K4me3 or anti-H3 antibodies and DNA quantified by real-time PCR with primers specific for the 5′ and 3′ regions of *PHO5*, *GYP5* and *ACT1*. H3K4me3 values were normalized to H3 values. Error bars reflect standard deviations of an average obtained from three independent experiments. Comparison of the mean differences was analysed using the Student’s *t* test. Schemes on the right indicate the sense/antisense transcriptional profile of *GYP5, PHO5* and *ACT1* and are based on experimental RT-qPCR data (Supplementary Figure S4A). (**D**) asRNA levels depend on H3K4 methylation. RT-qPCR analysis of *PHO5*, *GYP5*, *RAD4* and *RRI1* asRNAs in WT, *Δrrp6,* H3K4A*Δrrp6* and *Δset1Δrrp6* strains exponentially grown in SC medium. Values were normalized to *ACT1* and *SCR1* and expressed as fold changes relative to *Δrrp6* set to 1.

We next asked whether Set1 activity is also associated with transcription of asRNA. For this, we compared the genome-wide distribution of H3K4me3 marks at the TTS of genes in different classes using published ChIP–chip data (39). We found that the H3K4me3 levels between 0 and −300 nt from the TTS of ORFs that have a clear asRNA [Classes (I), (II) and (III)] are significantly higher compared with those at ORFs with low or no antisense [Class (IV) and unclassified control set] (Figure 4A), which is in agreement with recently published observations (28). ChIP analysis of H3K4me3 profiles on four specific genes (39), i.e. *PHO5* and *GYP5* (Class I), *RRI1* (Class II) and *RAD4* (Class III), confirmed increased levels of this modification at their 3′ ends (Figure 4B). To further support the hypothesis that the H3K4me3 peak at the 3′ end of antisense-producing genes reflects antisense transcription, we examined H3K4 trimethylation over the Class (I) genes *GYP5* and *PHO5* in WT and *Δpho4* strains (Figure 4C). Pho4 is a TF dispensable for *GYP5* transcription but essential for the expression of several *PHO* genes, including *PHO5* and *PHO84*. In *Δpho4*, H3K4 trimethylation peaks were detected both at the 5′ and 3′ end of the Pho4-independent *GYP5* gene, while for *PHO5* only a single peak of H3K4me3 was detected at the 3′ end, consistent with the absence of sense but maintenance of antisense transcription (see Supplementary Figure S4A for *PHO5* and *GYP5* sense and asRNA levels in these strains). As a control, we performed ChIP on the antisense-deficient *ACT1* gene, where, as expected, we detected a single peak at the 5′ end. Notably, the level of H3K4me3 at the 3′ end of both *GYP5* and *PHO5* was significantly enhanced on loss of Rrp6 in both WT and *Δpho4* backgrounds, indicating an increase of H3K4 trimethylation by Set1 in the absence of the exosome (Figure 4C).

To link the effect of Set1 on asRNA production to H3K4 methylation, we compared the levels of different ncRNAs in *Δset1* and the H3K4A mutant strain. In a *Δrrp6* background, the expression levels of asRNAs belonging to Class (I) (*PHO5* and *GYP5*), Class (II) (*RRI1*) or Class (III) (*RAD4*) were similarly reduced in combination with *Δset1* or *H3K4A* compared with *Δrrp6* alone (Figure 4D and Supplementary Figure S4B), indicating that the positive effect of Set1 on ncRNA production depends on the methylation of H3 on lysine 4. asRNA decay measurements in *Δset1* and K3K4A mutant strains showed similar half-lives compared with WT, supporting the view that H3K4 methylation by Set1 contributes to asRNA production rather than stability (Supplementary Figure S4C). Replacing *Δset1* by a Set1 catalytic mutant (*set1ca*, G951S) (42) in our tiling array analyses had slightly weaker, yet similar, effects on asRNA expression (Spearman correlation *R* = 0.71 and *P* < 2.2 e-16) as well as on ORFs (Spearman correlation *R* = 0.63 and *P* < 2.2 e-16) (Supplementary Figures S4D and see below). These observations together indicate that in addition to H3K4 methylation, the integrity of the Set1 complex may also contribute to the production of asRNAs.

We next sought to investigate the genome-wide contribution of Set1 to ncRNA production. Previous studies on global transcriptional effects of H3K4 methylation focused on the analysis of ORF mRNA expression, and most identified little contribution of Set1 to gene expression (28,43–46). We first compared ORF and antisense differential expression in *Δset1Δrrp6* versus *Δrrp6* versus *Δrrp6* versus WT. These analyses revealed a negative correlation for the asRNAs (−0.43), while no evident correlation was present for the ORFs (−0.096) (Figure S5A). This indicates that loss of Set1 mainly affects the expression of antisense transcripts that accumulate on loss of Rrp6. Additional analyses of the *Δset1* and *set1ca* strains using the whole population of ncRNAs (see Materials and Methods’ section for definition and Supplementary Table S3) confirmed that Set1 preferentially affects CUTs versus SUTs, and is generally more important for the expression of ncRNAs than ORFs (Supplementary Figure S5B and C).

To further understand the role of Set1, we studied the global relationship between H3K4me3 levels and Set1-dependent differential expression. For this, we compared H3K4me3 levels in WT (39) between ORF transcripts and ncRNAs that are downregulated in *Δset1Δrrp6* and those that are not affected by loss of Set1 (Supplementary Figure S5D). Because H3K4me3 correlates strongly with expression levels (39), we grouped genes and ncRNAs into four groups each based on their expression levels and analysed them separately. We found that a large fraction of ncRNAs (>30%) and only a modest subset of genes (<10%) are affected by loss of Set1. The affected ncRNAs and ORFs show increased H3K4 trimethylation compared with non-affected transcripts, and this increase is most significant for lowly expressed ORFs or ncRNAs. These data confirm a specific role of Set1 and H3K4me3 in promoting asRNA production.

In summary, we showed that antisense production is associated with H3K4 methylation by Set1. Strikingly, the H3K4 trimethylation marks at the 3′ end of asRNA-producing genes increase on loss of exosome activity (Figure 4C), suggesting that asRNA accumulation in *Δrrp6* might not be due to only stabilization but also increased transcription elongation, a process stimulated by Set1-dependent H3K4 methylation. These data extend our previous observations on *PHO84* (31) to a more general mechanism of many antisense-producing genes, and further support the view that loss of Rrp6 facilitates escape from early termination.

### Early termination by the NNS complex differentially modulates AS accumulation and silencing capability in the different classes

**Figure 5.**
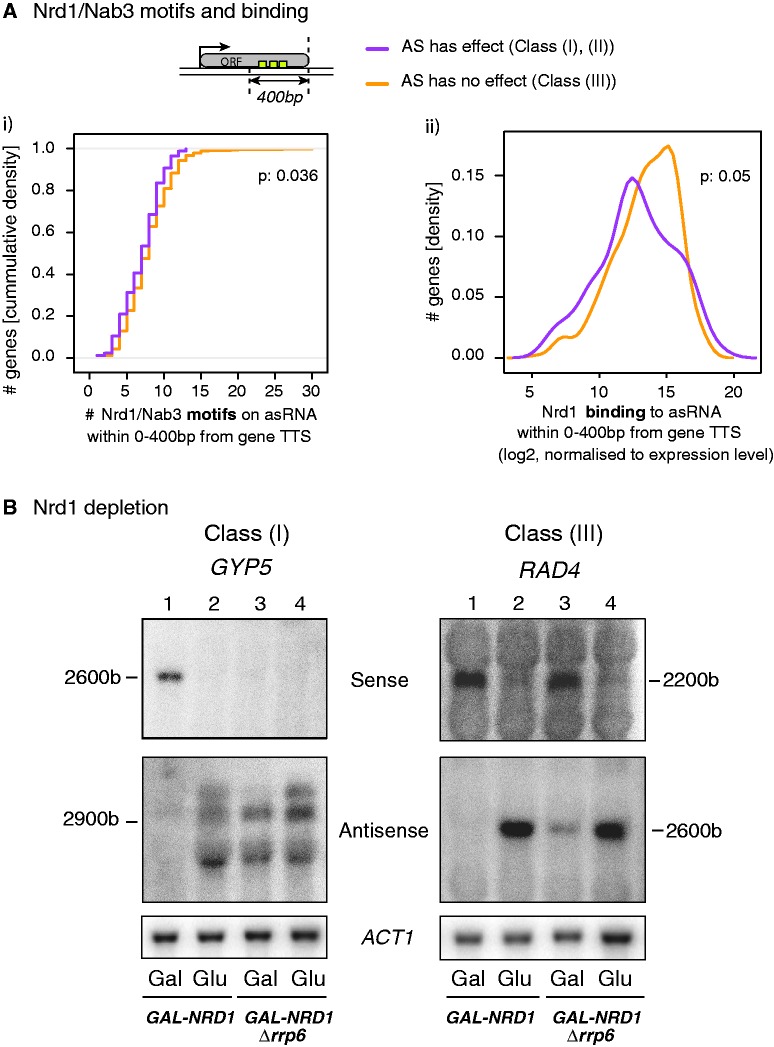
(**A**) Functional antisense transcripts exhibit fewer Nrd1/Nab3 motifs and lower Nrd1 binding than non-functional asRNAs. (i) The number of Nrd1 and Nab3 motifs was counted for each gene on the antisense strand within −400 to 0 nt upstream of the TTS (see schematic representation). Functional asRNAs [Classes (I) and (II), purple] contain fewer motifs than non-functional asRNAs [Class (III), yellow]. (ii) Nrd1 binding within the same region was obtained from the Corden laboratory (13) and normalized to the expression levels in WT. Consistent with (i), non-functional asRNAs (yellow) show more Nrd1 binding than functional classes (purple). *P*-values were calculated using the Wilcoxon rank sum test. (**B**) Nrd1 depletion increases asRNA levels and favours gene repression. The levels of *GYP5* [Class (I)] and *RAD4* [Class (III)] sense and asRNAs in the *GAL-NRD1* and *GAL-NRD1 Δrrp6* strains grown in galactose (Gal) or shifted for 6 h to glucose (Glu) were analysed by northern blotting. *ACT1* was used as a loading control.

Rrp6-sensitive ncRNAs (or CUTs) are normally eliminated in a process that involves transcription termination by the NNS complex. To better understand the role of early termination in the different classes, we next investigated Nrd1 and Nab3 binding across the functional [Classes (I) and (II)] and non-functional [Class (III)] antisense transcripts. Because the number of Nrd1 and Nab3 binding motifs within the first few hundred base pairs correlates with early termination and degradation efficiency (5), we searched for Nrd1 and Nab3 binding motifs on the antisense strand within 400 nt of the ORFs TTS. We then compared the number of motifs across functional and non-functional asRNAs. Interestingly, non-functional antisense transcripts from Class (III) genes contain significantly more Nrd1 and Nab3 motifs than those that are functional [Classes (I) and (II) (Figure 5Ai)]. We also compared the Nrd1 binding from PAR-CLiP data (13), and again found that non-functional asRNAs tend to be bound by Nrd1 more often than functional asRNAs (Figure 5Aii).

To address whether Class (III) genes may undergo more potent asRNA early termination by NNS than Class (I) genes, we examined the levels of a Class (I) (*GYP5*) and a Class (III) (*RAD4*) asRNA by northern blotting and RT-qPCR when depleting the essential Nrd1 protein using the glucose-repressible *GAL1* promoter in the presence or absence of Rrp6 (Figure 5B and Supplementary Figure S6A). Both *GYP5-* and *RAD4*-extended asRNA species strongly accumulate on depletion of Nrd1. Notably, the increase of *GYP5* asRNAs is comparable with that observed in *Δrrp6*, while the levels of *RAD4* asRNA are clearly higher than in *Δrrp6* (compare lanes 2 and 3, *GAL-NRD1* in Glu and *GAL-NRD1Δrrp6* in Gal). These observations indicate that asRNAs with strong Nrd1/Nab3 termination signals are less sensitive to the absence of Rrp6. Northern blot analyses revealed no major differences in the size of asRNAs accumulating on Rrp6 or Nrd1 depletion, indicating that similar termination signals are used in the two conditions (Figure 5B). Our northern blot analyses were not able to detect the short early terminated and unstable asRNAs (CUTs) identified earlier in *Δrrp6* at the *GYP5* and *RAD4* genes by 3′-long SAGE analysis (2), possibly because they are too short and/or too few. Of note, the accumulation of *GYP5* and *RAD4* asRNAs detected on depletion of Nrd1 was not significantly enhanced in the absence of Rrp6 (lane 4), suggesting that these extended asRNAs are not subject to degradation by Rrp6, likely because they are exported into the cytoplasm (Castelnuovo,M., unpublished data). Importantly, while loss of Rrp6 only affects *GYP5* sense expression, Nrd1 depletion was accompanied by a reduction of both Class (I) (*GYP5*) and Class (III) (*RAD4*) gene mRNA levels, indicating that abrogation of early termination can convert a non-functional into a functional asRNA (Figure 5B and Supplementary Figure S6A). This effect is unlikely due to direct modulation of *GYP5* and *RAD4* mRNAs by Nrd1 based both on PAR-CLiP analyses and the recent observation that NNS-mediated mRNA attenuation is a rare event (13,14).

**Figure 6.**
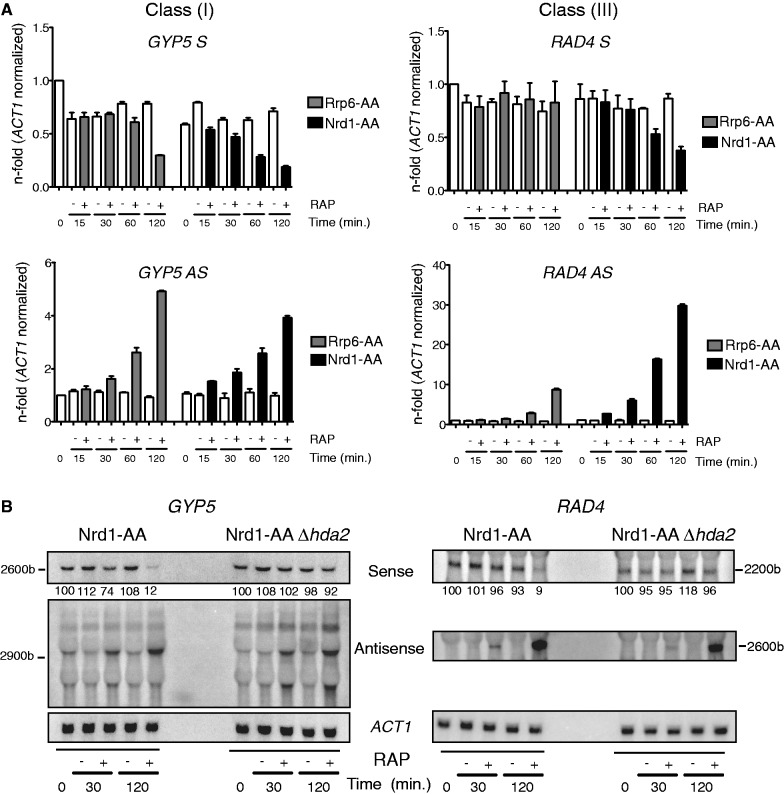
(**A**) Anchor away of Rrp6 and Nrd1 from the nucleus promotes asRNA production and gene repression. *GYP5* and *RAD4* sense (S) and antisense (AS) RNA levels were analysed by RT-qPCR in the anchor away strains Rrp6-AA (grey bars) and Nrd1-AA (black bars) grown in the presence of rapamycin (+) for the indicated times (minutes). The white bars correspond to S and AS RNA levels in the absence of rapamycin (−). All values are expressed as fold change compared with the Rrp6-AA strain at time 0 min and normalized to *ACT1.* (**B**) Hda2 contributes to Class (I) and (III) gene repression on anchor away of Nrd1. Northern blot analysis of *GYP5* and *RAD4* sense and antisense (AS) RNAs in the Nrd1-AA and Nrd1-AA*Δhda2* strains grown either in the absence (−) or presence (+) of rapamycin (RAP) for the indicated time in minutes. *ACT1* was used as a loading control. The *GYP5* and *RAD4* mRNA signals normalized to *ACT1* were expressed as a percentage of the signal detected at time 0 in the same strain.

To more accurately investigate the kinetics of antisense accumulation and sense repression, we induced rapid depletion of Rrp6 or Nrd1 from the nucleus using the rapamycin-inducible anchor away (AA) technique (33), and measured *GYP5* and *RAD4* sense and asRNA levels at various times after rapamycin treatment (Figure 6A and Supplementary Figure S6B). We confirm that loss of Rrp6 and Nrd1 results in a comparable increase in *GYP5* asRNA levels (5-fold versus 4-fold) accompanied by sense repression, while the accumulation of *RAD4* asRNA is weaker in Rrp6-AA compared with Nrd1-AA (8-fold versus 30-fold). Similar to the *GAL-NRD1* depletion experiment (Figure 5B), *RAD4* asRNA accumulation in the Nrd1-AA but not in the Rrp6-AA strain was accompanied by repression of sense transcription. Notably, during Nrd1 nuclear depletion, both *GYP5* and *RAD4* asRNAs accumulate 20–30 min before sense repression. In addition, there is an almost linear correlation between the amount of asRNA and the extent of silencing. We propose that sense repression depends on the frequency of elongated asRNAs escaping from an early termination control.

asRNA-dependent repression of Class (I) genes such as *PHO84* or *GYP5* involves the HDACs Hda1/2/3 and Rpd3 (Figures 1 and 2) and (29). To address whether the sense repression of Class (I) and Class (III) genes observed when rapidly depleting Nrd1 requires histone deacetylation, we deleted Hda2 in the Nrd1-AA strain. Interestingly, repression of *GYP5* and *RAD4* sense transcription observed in the Nrd1-AA strain grown in rapamycin was completely rescued in the absence of Hda2 (Figure 6B). As previously proposed (29,31), the Hda1/2/3 HDAC may trigger promoter deacetylation to consolidate repression initiated by antisense transcription through this region. These data suggest that asRNAs or antisense transcription may recruit histone-modifying enzymes to specific target sites as has already been proposed in higher eukaryotes (47).

## DISCUSSION

### Features of functional Rrp6-sensitive asRNA

Our study addressed the genome-wide effects of antisense transcripts that accumulate in *Δrrp6*, extending the single gene studies on *PHO84*, a model gene for asRNA-mediated transcriptional silencing in *Δrrp6.* We quantified the genes that become repressed through antisense transcription in a mechanism involving Set1 and/or the HDACs Hda1 and Rpd3, and identified major features of functional antisense transcripts (Figure 1). Among the 566 asRNAs that accumulate in *Δrrp6* [Classes (I), (II) and (III)], 97 [Classes (I) and (II)] are associated with the repression of sense gene expression (Figure 2). Earlier studies addressed the global effect of asRNAs that were classified as stable transcripts (SUTs) (16,48), which constitute a fraction of our Rrp6*-*sensitive asRNAs (Supplementary Figure S7A and B). Interestingly, our results suggest that functional asRNAs, whether previously studied SUTs or our Rrp6-sensitive transcripts, have similar features: both extend into the ORF promoter, and their repressive effects are targeting stress and other highly regulated genes (Figure 3) and (16,48).

In addition, we found that the repressive effect of Rrp6-sensitive asRNAs inversely correlates with the efficiency of early transcription termination by the NNS complex. Our recent studies on *PHO84* provided evidence that NNS function is affected in *Δrrp6* (31). Based on the described physical interactions between NNS and the nuclear exosome (6), loss of Rrp6 may compromise early termination by weakening optimal Nrd1 recruitment and binding to the transcript. Alternatively, loss of Rrp6 may indirectly affect early termination through accumulation of ncRNAs bound to Nrd1/Nab3, preventing the recycling of the complex. This Nrd1/Nab3 sequestration in *Δrrp6* may primarily affect transcription attenuation of asRNAs with weak Nrd1/Nab3 binding sites. Accordingly, the fold increase in asRNA levels on depletion of Nrd1 versus Rrp6 is weaker for Class (I) compared with Class (III) asRNAs (Figures 5 and 6A). Class (I) asRNAs, including *PHO84* antisense, but also Class (IV) asRNAs are subjected to weak early termination and can often be detected in WT cells (31) (data not shown). SUTs, which are less prone to early termination and degradation by Rrp6 contain fewer Nrd1/Nab3 binding motifs than CUTs, and this feature is even more pronounced in ORFs (Supplementary Figure S7C).

Our observations on asRNA accumulation following Nrd1 depletion have recently been extended to the whole genome by the Cramer laboratory (14). This study identified 1526 Nrd1 unterminated transcripts or NUTs on rapid nuclear depletion of Nrd1, which overlap with a large fraction of CUTs (68%) as well as SUTs (58%) but poorly with mRNAs. Thus, early termination by Nrd1 ensures proper gene expression mainly by restricting divergent transcription from bidirectional promoters as well as asRNAs at most yeast ORFs.

### Role of Set1 and Rrp6 in the production of asRNAs

Despite the general occurrence of H3K4 methylation on active genes, we found, consistent with previous studies, that loss of Set1 has only minor global effects on steady-state gene expression (28,43,44,46). However, we identified a role for Set1 in promoting ncRNA production, a feature mostly revealed in *Δrrp6* (Supplementary Figure S5), when early termination by Nrd1 is compromised. Support for this role comes from a recent study, which proposed that while H3K4me2 is involved in sense repression, H3K4me3, deposited by Set1, contributes to asRNA production (28). Our results from half-life analyses in *Δset1* and H3K4 mutants further indicate that the positive effect of H3K4me3 on asRNA levels in *Δrrp6* is due to increased asRNA production rather than increased stability (Supplementary Figure S4C). Consistently, we showed that loss of Rrp6 alone results in increased asRNA-dependent H3K4 methylation over the *PHO84* gene (31) and two other Class (I) genes *PHO5* and *GYP5* (Figure 4C).

The preferential role of Set1 in promoting Rrp6-sensitive asRNAs indicates a functional link between Set1 and Rrp6, and hence with NNS. Consistently, the ncRNAs most affected by loss of Set1 contain more Nrd1/Nab3 binding motifs and show higher Nrd1 binding (Supplementary Figure S7D). Interestingly, NNS is recruited through interaction with Ser5 phosphorylated PolII C-terminal domain (7,8,10), and loss of Set1 was recently shown to increase Ser5 phosphorylated C-terminal domain as well as Nab3 and Sen1 recruitment (49). Together, these observations suggest a model in which Set1 and/or H3K4 trimethylation contribute to antisense production by interfering with NNS-mediated transcription attenuation. We speculate that this positive effect of Set1 on asRNA transcription may be preferentially revealed in *Δrrp6*, as our data indicate that the absence of Rrp6 compromises NNS early termination (31) and (Figure 4C).

### Promoter repression by Rrp6- and Nrd1-sensitive asRNAs

Our study expands previous findings on the links between histone modifications and antisense production, and suggests potential mechanisms of how asRNAs contribute to silencing. Antisense-induced repression of the 28 Class (I) genes is strongly dependent on the presence of Set1, Hda1 and Rpd3 (Figures 1 and 2). Set1-dependent H3K4 dimethylation of non-coding upstream or antisense transcription has previously been implicated in gene repression by acting as a signal to recruit the Rpd3 or Set3 HDACs (24–26,28,50–52). Thus, H3K4 tri- and dimethylation deposited by Set1 during non-coding transcription may contribute to silencing in two ways, respectively, through stimulation of antisense production and by signalling the recruitment of specific HDACs to the sense promoter.

Our analysis of promoter structure in the context of antisense classes suggests a link between asRNA production and promoter remodelling. The promoters of Class (I) and (IV) genes, which produce asRNAs and show derepression in the absence of Set1, Hda2 and Rpd3, are enriched for TATA boxes (as well as closed promoter configurations in the case of Class I). These genes are often transcribed in bursts, and the promoters tend to be regulated through extensive nucleosome rearrangements on the on–off switch (36,53), consistent with their sensitivity to histone modifications. Of note, our recent studies on the *PHO84* Class (I) gene using single-molecule FISH indicate anti-correlation of sense and asRNA expression in individual cells (31). Together these observations suggest a model in which, once a transcription burst is over, low-frequency antisense transcription through the promoters of Class (I) and (IV) genes contributes to nucleosome repositioning and occlusion of TF binding sites, thereby raising the threshold of the transcription on-switch (53). The HDACs Hda1 and Rpd3 may either facilitate nucleosome repositioning or act downstream to consolidate repression by further reducing promoter accessibility.

We showed that Nrd1 depletion converts a ‘non-functional’ into a ‘functional’ asRNA (Class III), which is able to repress sense mRNA expression. The data suggest that the repression is linked to the frequency of asRNA molecules that escape early termination and nuclear degradation, so that they can extend into the sense promoter region. The transcription process *per se* over the promoter could trigger chromatin changes altering nucleosome occupancy and/or histone modifications. The almost complete rescue of sense repression observed in Hda2 mutants, even in the presence of high levels of antisense transcription over the promoter in the Nrd1-AA strain, highlights the crucial importance of histone deacetylation in the establishment or consolidation of the silencing process. Nearly 15% of the genes that accumulate antisense NUTs following Nrd1 depletion show mRNA repression (14). It will be interesting to define what fraction of these responsive genes is repressed in a process dependent on Hda2 or through alternate mechanisms based on transcription interference or polymerase collision (54).

### Regulation of antisense-mediated gene repression

Our results indicate that a significant number of antisense transcripts are regulated at the level of early termination through a process implicating NNS and Set1. The recently published data from the Cramer laboratory confirm the global effect of Nrd1 early termination on restricting ncRNA transcription and potential negative effects on gene expression (14). Our results indicate that the efficiency or strength of asRNA early termination may be variable, raising the possibility that leaky early termination represents a selective advantage in the case of Class (I) and (II) genes, possibly because it may contribute to optimal regulation of gene expression. Interestingly, recent studies show that NNS activity is downregulated in response to changes in physiological conditions (55), potentially influencing asRNA expression levels and hence asRNA-dependent silencing. Notably, Rrp6 also undergoes physiological control and is downregulated during the onset of the meiotic program, giving rise to specific ncRNAs, that may be required for the proper expression of genes at the entry of meiosis (56). Our observations predict that these meiosis-specific ncRNAs result from increased read-through transcription of ncRNAs with weak early termination signals.

A recent study indicates that co-transcriptional decapping of nuclear asRNAs by Dcp2 followed by 5′–3′ degradation by the nuclear exonuclease Rat1 may represent an alternative mechanism to regulate antisense transcription and expression of the corresponding gene (27). We found little overlap between the 100 identified Dcp2- and the Rrp6-sensitive asRNAs described in our study, as most correspond to the unclassified genes (data not shown), suggesting that asRNA production may be regulated by at least two pathways depending on the gene: either nuclear decapping and degradation by Rat1 or early termination by NNS linked to degradation by the TRAMP-exosome complex. A fraction of Dcp2- and Rrp6-sensitive asRNAs have also been described as XUTs, non-coding transcripts that accumulate in the absence of the cytoplasmic 5′–3′ exonuclease Xrn1, indicating that they are exported and degraded in the cytoplasm (27,31,52,57). It will be interesting to determine the nucleocytoplasmic distribution of asRNAs from different gene classes in different mutant backgrounds by visualization of single transcripts, as we recently did for *PHO84* (31). Such experiments may reveal the relationship between asRNA transcription regulation, localization and effect on gene expression. The conservation of sense/antisense pairs through evolution and their wide occurrence in higher eukaryotic species (17,48,58) suggest that similar mechanisms may underlie the regulation of antisense production and their effects on the fine-tuning of gene expression in metazoans.

## SUPPLEMENTARY DATA

Supplementary Data are available at NAR Online.

## FUNDING

EMBL (to J.B.Z. and N.M.L.); SystemsX and Novartis fellowships (to M.C.); an EMBO fellowship (to E.G.); NIH (to L.M.S.); EpiGeneSys FP7 Network of Excellence (to N.M.L.); Swiss National Science Foundation [31003A_130292 to F.S.]; NCCR ‘Frontiers in Genetics’, iGE3 and the Canton of Geneva. Funding for open access charge: Swiss National Science Foundation.

*Conflict of interest statement*. None declared.

## Supplementary Material

Supplementary Data
