# Supplementary material for: Role of histone modifications and early termination in pervasive transcription and antisense-mediated gene silencing in yeast
Source: Nucleic Acids Res. 2014 Jan 31;42(7):4348–62. doi: 10.1093/nar/gku100 (PMC3985671; doi:10.1093/nar/gku100)
Supplement: Supplementary Data [file supp_42_7_4348__index.html]

Role of histone modifications and early termination in pervasive transcription and antisense-mediated gene silencing in yeast — Role of histone modifications and early termination in pervasive transcription and antisense-mediated gene silencing in yeast — Supplementary Data 

# Role of histone modifications and early termination in pervasive transcription and antisense-mediated gene silencing in yeast

## Supplementary Data

files

**Files in this Data Supplement:**

- Supplementary Data - pdf file
