# Supplementary material for: Role of histone modifications and early termination in pervasive transcription and antisense-mediated gene silencing in yeast
Source: Nucleic Acids Res. 2014 Jan 31;42(7):4348–62. doi: 10.1093/nar/gku100 (PMC3985671; doi:10.1093/nar/gku100)
Supplement: Supplementary Data [file supp_gku100_nar-01947-x-2013-File008.pdf]

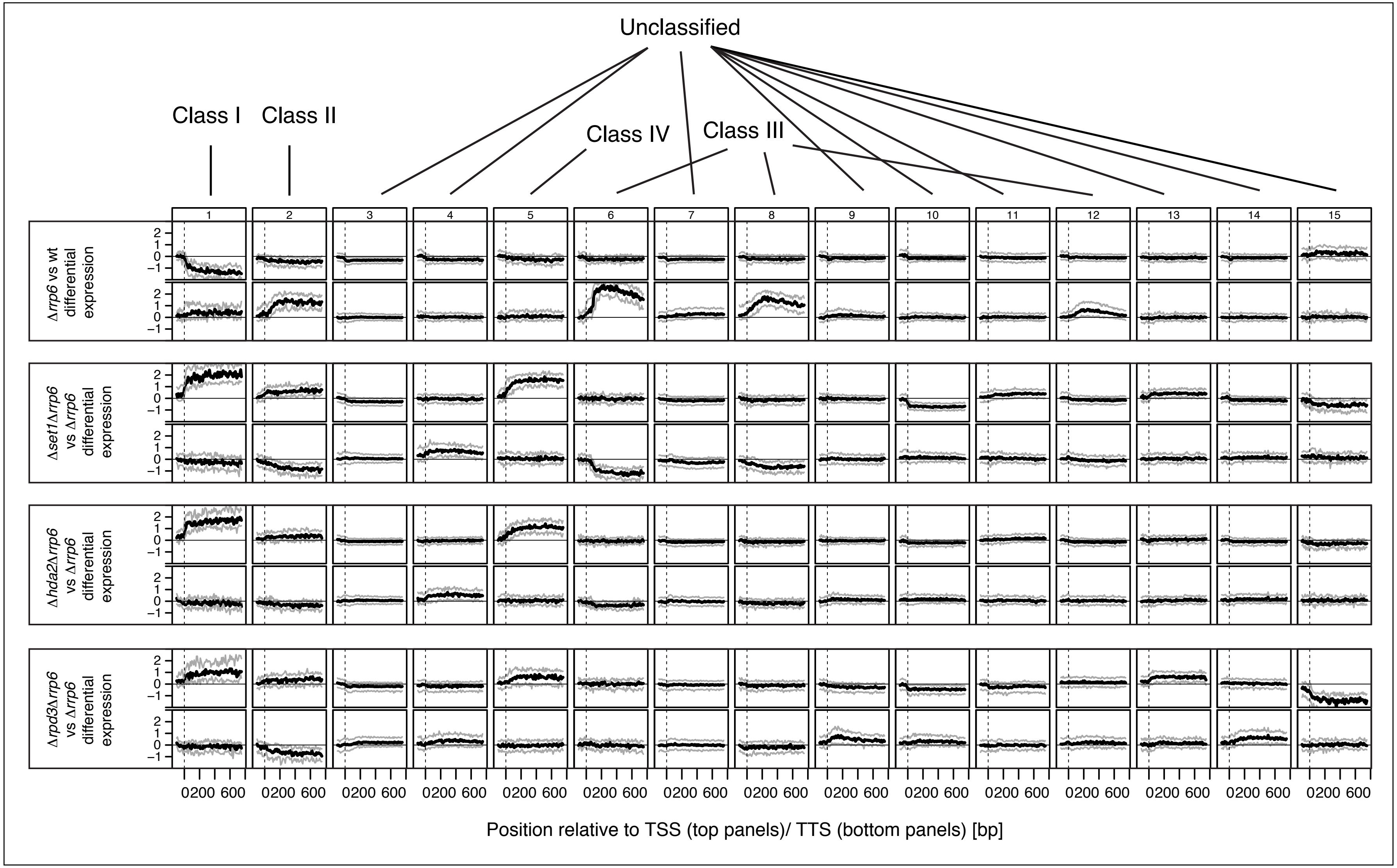

Figure S1: **Description of the 15 initial clusters and their grouping.** The differential expression profiles of the 15 initial clusters are shown as median (black), 25 and 75 percentile (grey) for all mutants (top to bottom). The top panels show the profile in sense direction relative to the TSS, the bottom profiles show the profiles on the antisense strand, relative to the TTS. We used pam (partition around medoids) implementation in R (11) to cluster the genes based on their expression in sense and antisense direction (-100 to +750nt from the TSS in sense and from the TTS in antisense, see Methods). To define the gene Classes, clusters were grouped as shown.

Of note, a recent study by Churchman and Weissman (2011) based on nascent transcript sequencing showed that many antisense transcripts are transcriptionally repressed by Rpd3S recruited through H3K36me3 at 3' end of genes (59). These Rpd3S sensitive asRNAs are not part of our Class (I), (II) or (III) genes, as they do not accumulate in *Δrrp6* alone. However, they are detected in *Δrrp6Δrpd3* and segregate with gene clusters 9 (234 genes) and 14 (259 genes) which belong to the control set of unclassified genes (See also Fig. S2B).

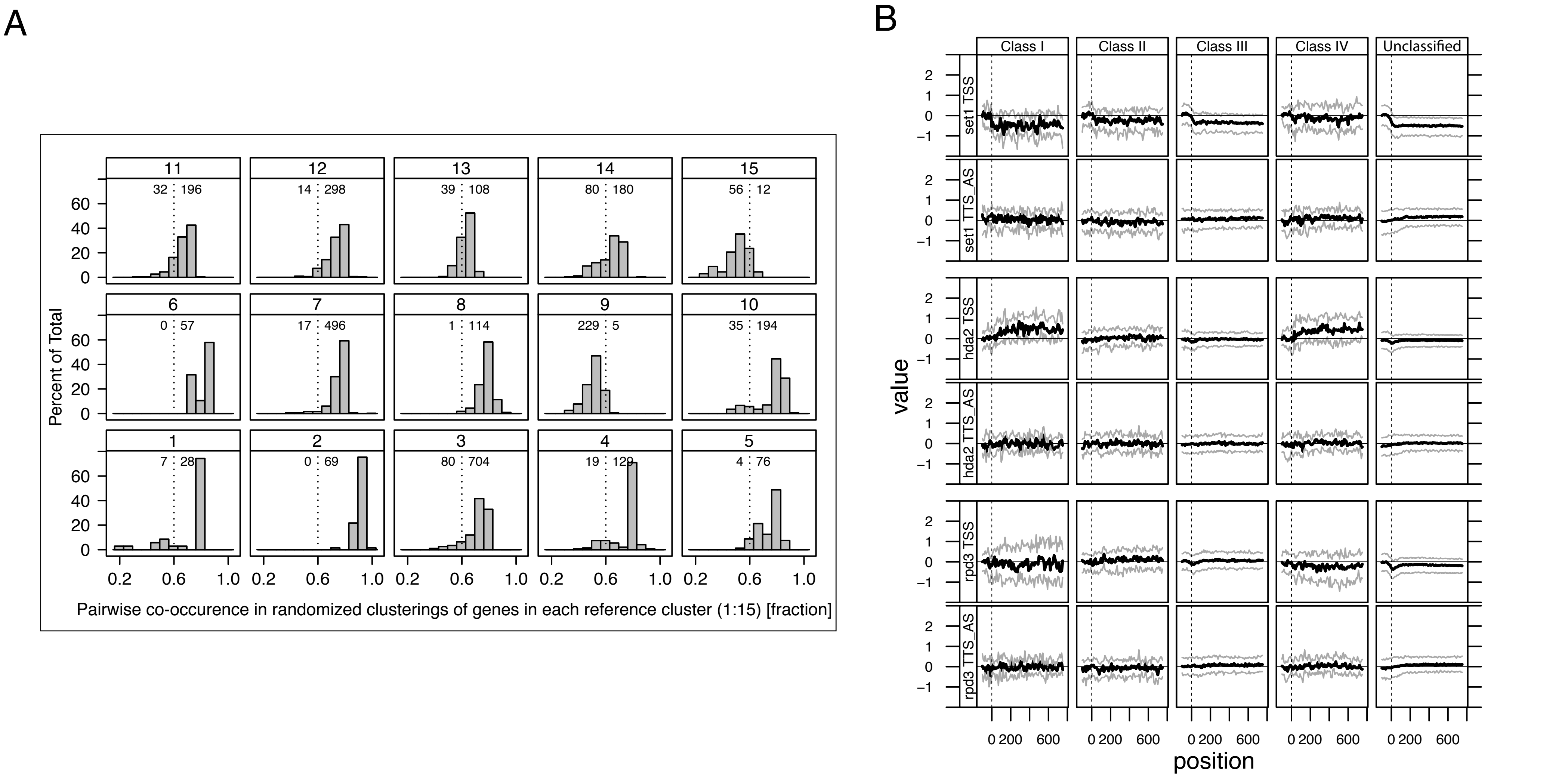

Figure S2: **A) Robustness test**. The distribution of how often 2 genes of the same original cluster are found in randomized clusterings. We performed randomized pam clustering with different numbers of clusters (10-15) only using randomly selected 80% of the genes in each trial. We repeated this 20 times for each number of clusters (120 in total) and then counted how often two genes from the original clustering were found in the same cluster and plotted the values as a histogram. Based on these histograms we set a threshold of 60% for calling a gene robustly set in a given cluster. Non-robustly clustered genes were excluded from further analysis.
B) Profiles of Class I-IV and unclassified genes in single mutants. The differential expression profiles are shown for the 5 Classes for the single mutant vs WT cases. Clusters are shown as median (black), 25 and 75 percentile (grey) for all mutants (top to bottom). The top panels show the profile in sense direction relative to the TSS, the bottom profiles show the profiles on the antisense strand, relative to the TTS.

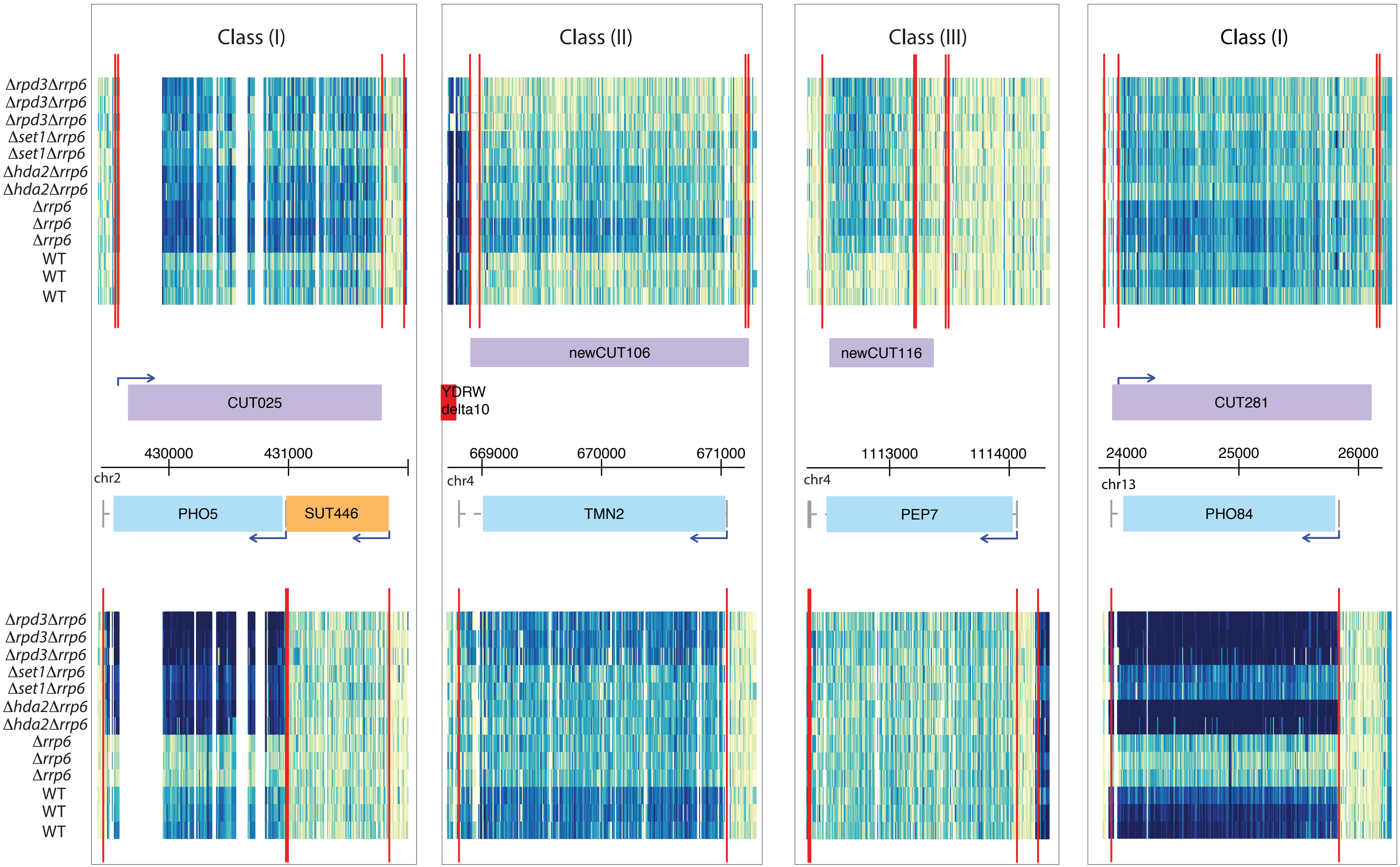

Figure S3: Heatmaps of specific genes from Class (I), (II) and (III), including *PHO84* (Class i).

Supplementary Figure S4

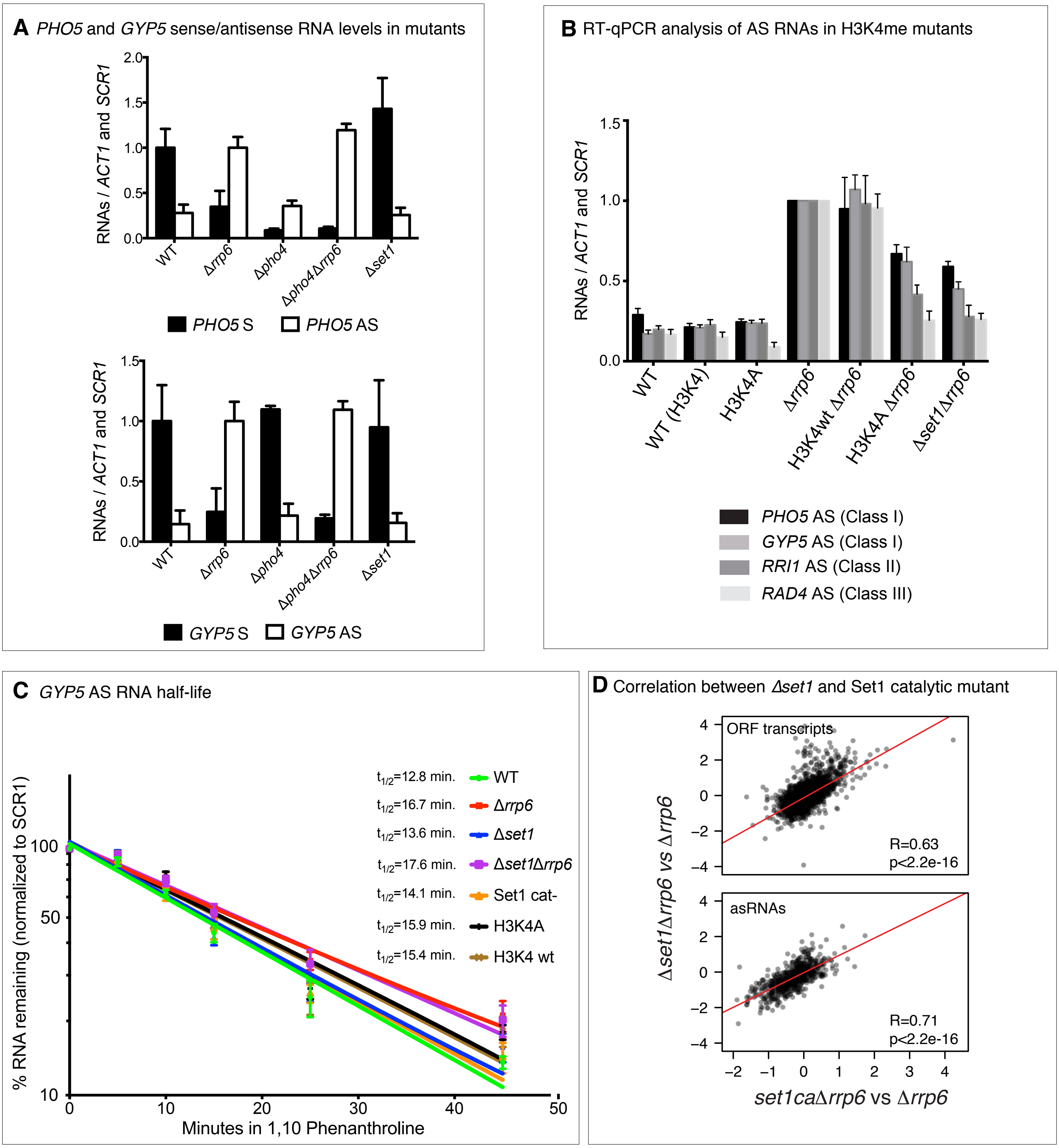

Figure S4: **(A)** RT-qPCR analysis of *PHO5* and *GYP5* sense and AS transcripts in wild-type, *Δrrp6*, *Δpho4*, *Δpho4Δrrp6*, *Δset1* strains exponentially grown in SC medium. Values expressed as fold change normalized to *ACT1* and *SCR1* housekeeping genes. **(B)** RT-qPCR analysis of Class (I), Class(II) and Class(III) AS RNAs in mutants affecting H3K4 methylation. The indicated strains were exponentially grown in SC medium. Values are expressed as fold change normalized to *ACT1* and *SCR1* housekeeping genes. **(C)** Half-life ($t_{1/2}$) measurement of *GYP5* AS RNA in mutants affecting H3K4 methylation. *GYP5* AS RNA levels were defined by RT-qPCR after blocking transcription with 1,10 Phenanthroline for 5, 10, 15, 25 and 45 minutes. **(D)** *Δset1* and the Set1 catalytic mutant (*set1ca* G951S) (42) similarly affect ORFs and asRNAs in *Δrrp6*.

Supplementary Figure S5

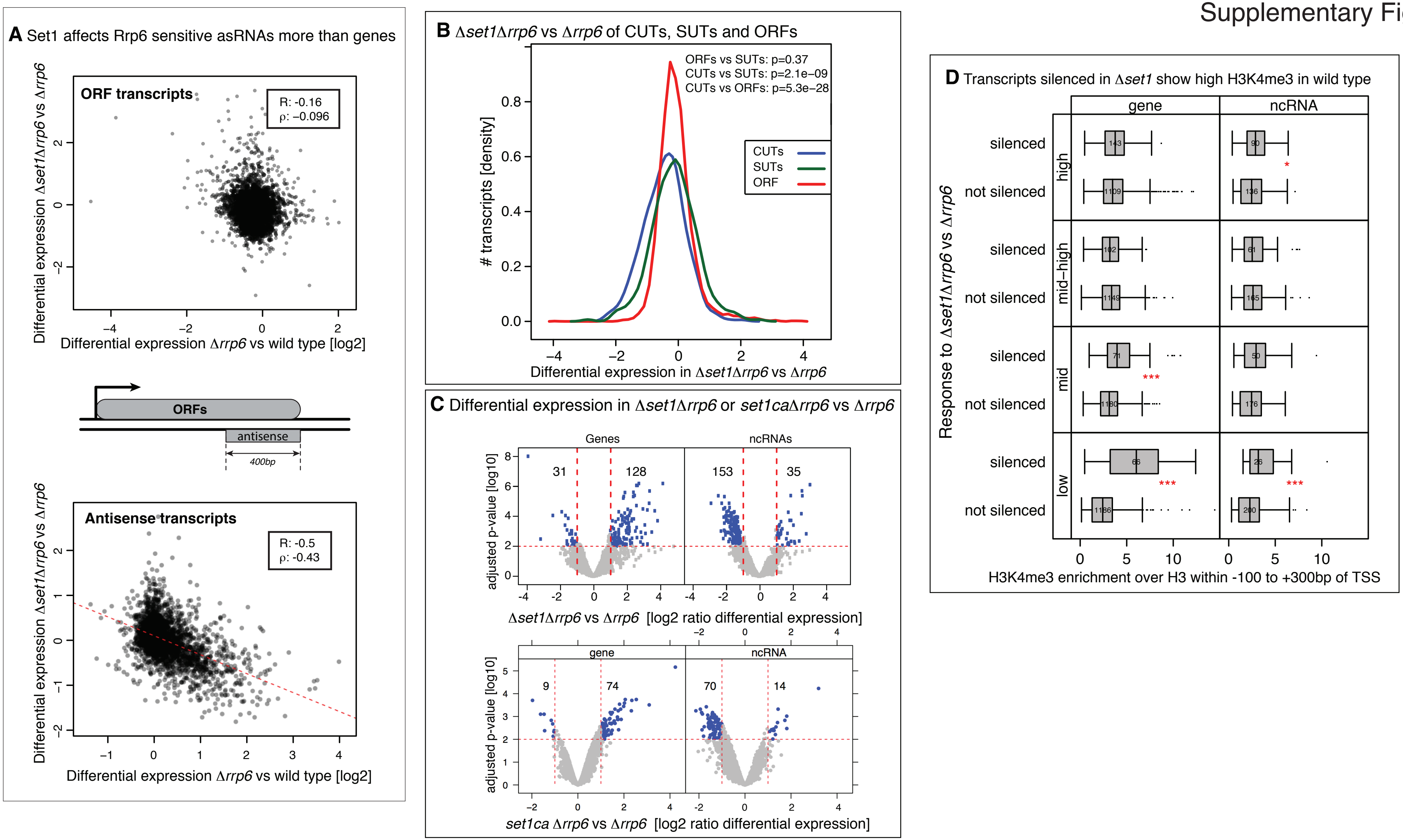

Figure S5: **Set1 preferentially affects CUTs.** **(A)** Set1 mainly affects non-coding RNAs. A scatterplot of *Δset1Δrrp6* vs *Δrrp6* versus *Δrrp6* vs WT expression is shown for genes (top) and the first 400bp of the antisense strand (bottom). There is a clear anti-correlation of *Δset1Δrrp6* vs *Δrrp6* with *Δrrp6* for the antisense transcripts. No such trend is found for genes. **(B)** The distribution of differential expression values in *Δset1Δrrp6* vs *Δrrp6* is shown for CUTs (blue), SUTs (green) and ORFs (red). CUTs are most affected by *Δset1* in *Δrrp6* background, while there is no effect on ORFs. SUTs fall between the two. **(C)** Scatterplot of p-values(-log10 transformed) vs fold-change (log2 transformed) of differential expression in *Δset1Δrrp6* vs *Δrrp6* and *set1caΔrrp6* vs *Δrrp6* is shown for genes (left) and ncRNAs (right). P-values were adjusted using the Benjamini-Hochburg multiple testing correction, transcripts were defined as significantly up/down-regulated (blue dots) below a p-value of 0.01 and above a absolute fold change ratio of 2 (horizontal and vertical red lines respectively). **(D)** H3K4me3 at promoter correlates with response to the *Δset1* mutant. Genes (left) and the whole population of ncRNAs (right) (see Materials and Methods for ncRNA definition) were grouped into four classes each, based on their expression level in *Δrrp6*. For each expression-class we then compared the distribution of H3K4me3 in promoter regions (-100:+300bp of the respective TSS) for transcripts that are silenced, or not-affected in the *Δset1Δrrp6* double mutant versus *Δrrp6* (the number of transcripts of each type is indicated in the boxes). There is a clear trend, that those that are silenced by the double mutant have the highest level of H3K4me3 across all expression-classes. P-values are given for pair-wise wilcoxon rank sum tests (***<0.001, **<0.01, *<0.05).

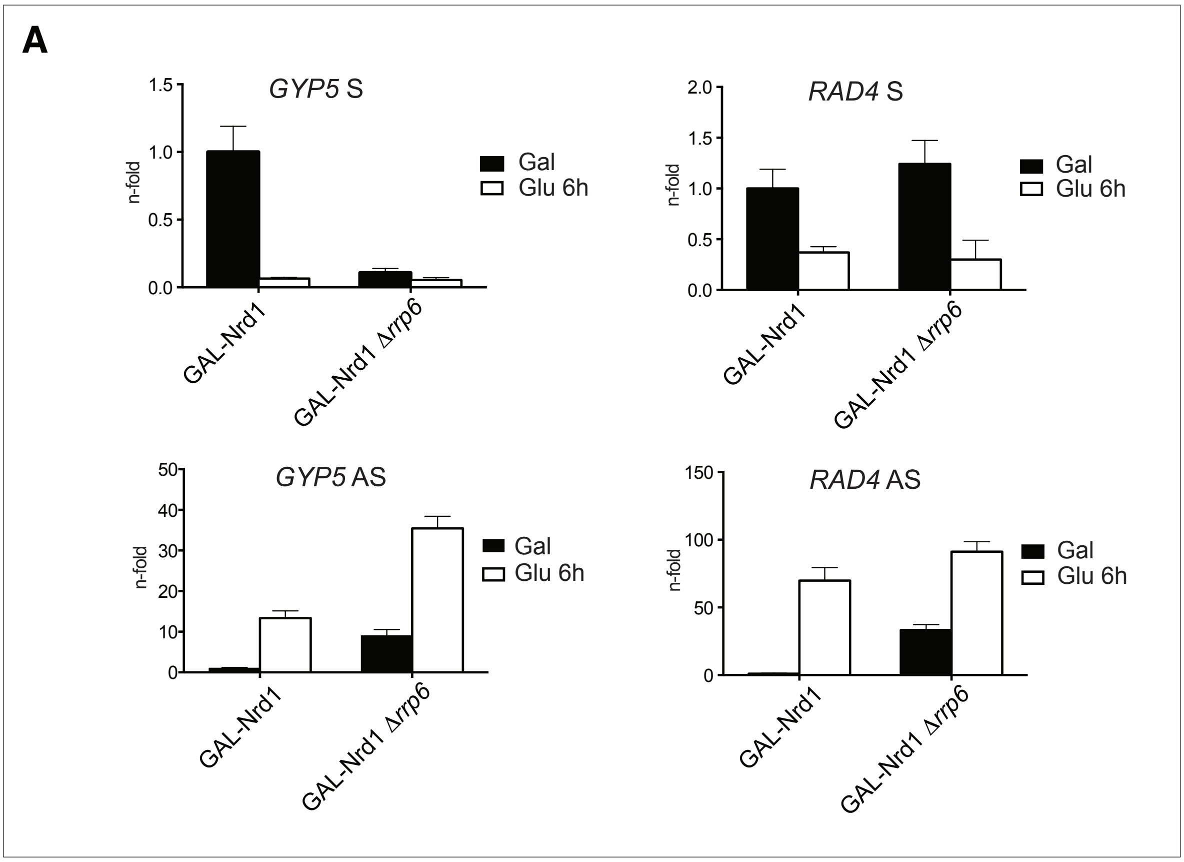

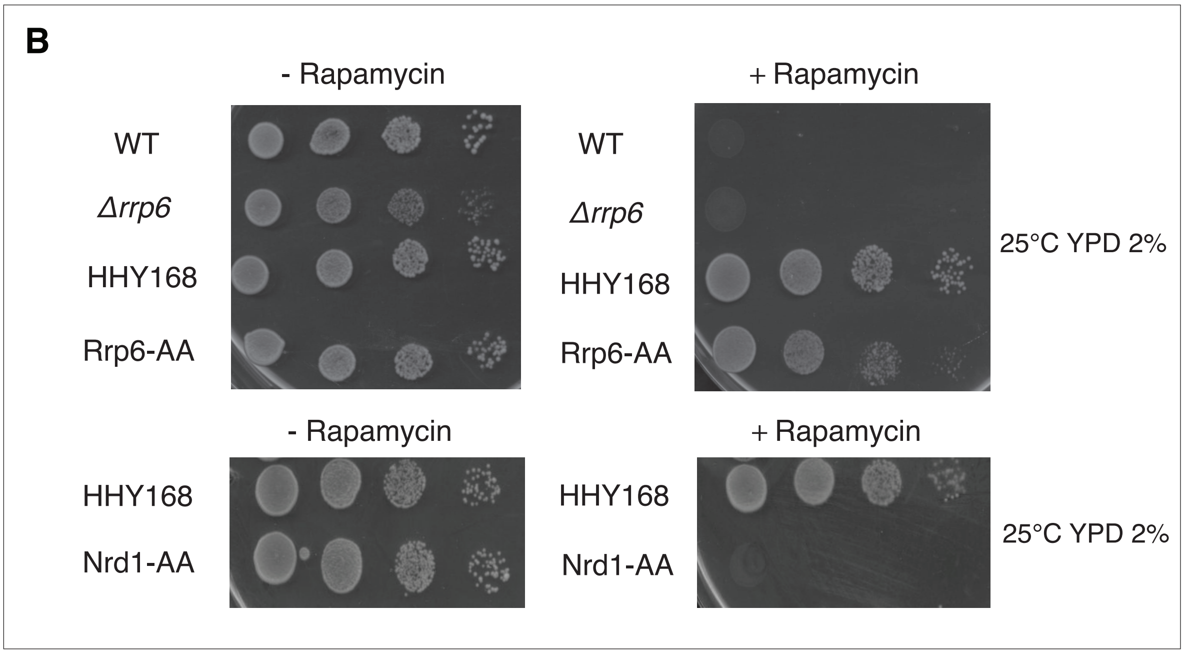

Figure S6: **(A)** Nrd1 depletion increases Class (I) and Class (III) AS RNA levels and favors gene repression. In the *GAL-NRD1* strain, Nrd1 is under the control of the *GAL1* promoter and its expression is repressed by glucose. Sense (S) and antisense (AS) RNAs were measured by RT-qPCR with strand specific oligos in the *GAL-NRD1* and *GAL-NRD1 Δrrp6* strains grown in galactose (Gal) or shifted for 6h to glucose (Glu). Values, normalized to *ACT1,* are expressed as fold change with respect to S or AS RNA levels in the *GAL-NRD1* strain grown in galactose. **(B)** Spot test analysis of the Rrp6-AA and Nrd1-AA strain in the absence (left) or presence (right) of Rapamycin. 10x fold dilutions of the rapamycin sensitive WT and Δ*rrp6* strains, the rapamycin resistant strain HHY168 alone or expressing Rrp6-FRB (Rrp6-AA) or Nrd1-FRB (Nrd1-AA) were spotted and grown for 3 days at 25°C. The Rrp6-AA strain grows as slowly as Δ*rrp6* when rapamycin is present in the medium (1μg/ml). The Nrd1-AA strain is lethal in the presence of rapamycin.

A Percentage of CUTs/SUTs in each class

	Class I		Class II		Class III		Class IV		Class V	
no AS described	21.4%	6	27.5%	19	50.7%	238	63.2%	48	80.3%	1626
AS SUT	57.1%	16	30.4%	21	16.4%	77	23.7%	18	13.0%	264
AS CUT	21.4%	6	42.0%	29	32.8%	154	13.2%	10	6.6%	134

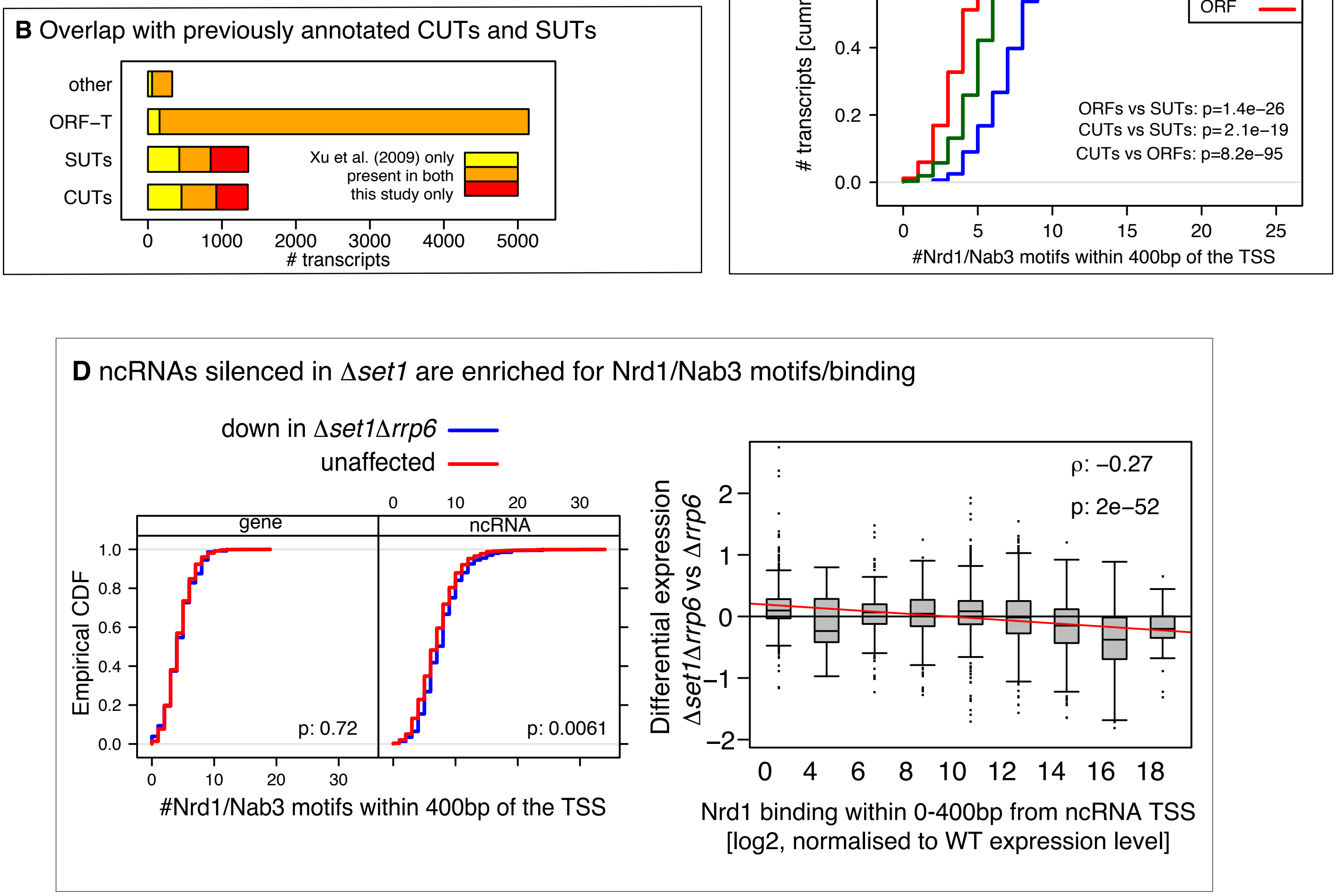

Figure S7: **SUTs, CUTs, Nrd1/Nab3 binding and Set1.** **(A)** To calculate the overlap between our Rrp6 sensitive asRNAs and previously annotated SUTs or CUTs (16), we identified gene-SUT/CUT sense-antisense pairs and considered our asRNAs as previously annotated if the gene in question had a previously annotated antisense SUT/CUT. **(B)** Shows the overlap between transcripts detected in this study and the transcripts annotated by Xu et al 2009 (3). Most ORF transcripts were detected in both studies whereas the overlap of SUTs and CUTs is much lower. These are due to differences in strain background (S96 vs W303 in this study) as well as in RNA preparation (Xu et al. 2009 (3) used polyA enriched RNA whereas this study used total RNA). **(C)** CUTs have more Nrd1 sites than SUTs. The cumulative density of the number of Nrd1/Nab3 motifs within 400nt of the TSS is shown for CUTs (blue), SUTs (green) and ORFs (red). CUTs have most Nrd1/Nab3 motifs within 400nt, ORFs have least and SUTs have an intermediate number. Annotations for ORFs, CUTs and SUTs (A, B, C) or all ncRNAs (D) are as described in Xu et al. 2009 (3) and (SI Methods). These include both antisense and intergenic ncRNAs. The pairwise p-values are given for the Wilcoxon's rank sum test. **(D)** *Δset1*-silenced ncRNAs show more Nrd1/Nab3 motifs and Nrd1 binding. ncRNAs were separated into silenced (blue) and not affected by *Δset1* (red). We then compared the number of Nrd1 and Nab3 motifs within the first 400bp of each transcript. *Δset1*-silenced transcripts show more motifs. This was confirmed when we binned the transcripts based on Nrd1 binding and found that the more the transcripts bound Nrd1, the more silenced they were in *Δset1Δrrp6*. The red regression line was fitted to the data according to the model: Y~X where Y is the differential expression *Δset1Δrrp6* vs *Δrrp6* and X the amount of Nrd1 binding obtained from CLiP experiments (13).

Table S1: Strains and primers used in this study

Strains

Code	Name	Genotype	Reference
W303 background			
FSY1742	WT	*MATa ade2 his3 leu2 trp1 ura3*	
FSY3117	*Δrrp6*	*MATa ade2 his3 leu2 trp1 ura3 Δrrp6::KANr*	Camblong et al., 2007 (29)
FSY3518	*Δhda2*	*MATa ade2 his3 leu2 trp1 ura3 Δhda2::TRP1*	Camblong et al., 2007 (29)
FSY3018	*Δhda2Δrrp6*	*MATa ade2 his3 leu2 trp1 ura3 Δrrp6::KANr Δhda2::TRP1*	this study
FSY3383	*Δrpd3*	*MATa ade2 his3 leu2 trp1 ura3 Δrpd3::TRP1*	this study
FSY3384	*Δrpd3Δrrp6*	*MATa ade2 his3 leu2 trp1 ura3 Δrrp6::KANr Δrpd3::TRP1*	this study
FSY3517	*Δset1*	*MATa ade2 his3 leu2 trp1 ura3 Δset1::TRP1*	this study
FSY3833	*Δset1Δrrp6*	*MATa ade2 his3 leu2 trp1 ura3 Δrrp6::KANr Δset1::TRP1*	this study
FSY4763	*Set1ca G951S*	*MATa ade2 his3 leu2 trp1 ura3 set1::set1 ca*	Sollier et al., 2004 (42)
FSY4776	*Set1ca G951S Δrrp6*	*MATa ade2 his3 leu2 trp1 ura3 Δrrp6::LEU2 set1::set1 ca*	this study
FSY3353	H3H4 wt (yAM212)	*MATa ade2 his3 leu2 trp1 ura3 hht1-hhf1::LEU2*	Santos-Rosa et al., 2002 (45)
		hht2-hhf2::HIS3 pRS314-HHT2-HHF2	
FSY3831	H3H4 *Δrrp6*	*MATa ade2 his3 leu2 trp1 ura3 Δrrp6::KANr hht1-hhf1::LEU2*	Santos-Rosa et al., 2002 (45)
		hht2-hhf2::HIS3 pRS314-HHT2-HHF2	and this study
FSY3357	H3K4A (yAM216)	*MATa ade2 his3 leu2 trp1 ura3 hht1-hhf1::LEU2*	Santos-Rosa et al., 2002 (45)
		hht2-hhf2::HIS3 pRS314-hht2K4A-HHF2	
FSY3832	H3K4A *Δrrp6*	*MATa ade2 his3 leu2 trp1 ura3 Δrrp6::KANr hht1-hhf1::LEU2*	Santos-Rosa et al., 2002 (45)
		hht2-hhf2::HIS3 pRS314-hht2K4A-HHF2	and this study
FSY4275	*GAL-NRD1*	*MATa ade2 his3 leu2 trp1 ura3 HisMX6-pGAL-NRD1*	Thiebaut et al., 2006 (5)
FSY4282	*GAL-NRD1 Δrrp6*	*MATa ade2 his3 leu2 trp1 ura3 HisMX6-pGAL-NRD1 Δrrp6::KANr*	Thiebaut et al., 2006 (5)
FSY4886	*HHY168 parental*	*MATalpha tor1-1 fpr1::NAT RPL13A-2×FKBP12::TRP1*	Haruki et al., 2008 (33)
FSY5015	Rrp6-AA	*MATalpha tor1-1 fpr1::NAT RPL13A-2xFKB12::TRP1 Rrp6-FRB::kanMX6*	this study
FSY5065	Nrd1-AA	*MATalpha tor1-1 fpr1::NAT RPL13A-2xFKB12::TRP1 Nrd1-FRB::kanMX6*	this study

Primers

Code	Name	Sequence
OFS1741	*ACT1* F Mid	5'-TTCCAGCCTTCTACGTTTCCATC-3'
OFS1742	*ACT1* R Mid	5'-CGTGAGGTAGAGAGAAACCAGC-3'
OFS735	*ACT1* F 3'	5'-TACTCCGTCTGGATTGGTGGTT-3'
OFS736	*ACT1* R 3'	5'-GGTGAACGATAGATGGACCACTT-3'
OFS737	*ACT1* F 5'	5'-TGGTATGTTCTAGCGCTTGCAC-3'
OFS738	*ACT1* R 5'	5'-GTCAATATAGGAGGTTATGGGAGAGTG-3'
OFS2034	*GYP5* F Mid	5'-TACAGTGGCAAGCAACGAAC-3'
OFS2035	*GYP5* R Mid	5'-TTGGAATTCGCCATCAGC-3'
OFS2026	*GYP5* F 3'	5'-CAATGAGGTGATGAGGGAGAA-3'
OFS2027	*GYP5* R 3'	5'-AAACCAGTCCAGCCTTTCCT-3'
OFS2024	*GYP5* F 5'	5'-CCTCTGAAGTTCACGAAGGTG-3'
OFS2025	*GYP5* R 5'	5'-TGGCCTAAACTTTGTTGCTTG-3'
OFS2028	*PHO5* F 3'	5'-CAACGCCAGTCTATTGAGACA-3'
OFS2029	*PHO5* R 3'	5'-CAGATTTTAATCTTTCGGCAAA-3'
OFS2030	*PHO5* F 5'	5'-TCAATTTTAGCCGCTTCTTTG-3'
OFS2031	*PHO5* R 5'	5'-CCACCCAAAAATGGGAAGAT-3'
OFS1828	*RAD4* F	5'-AGGTGATAAAGAGGACAGTTGGAAG-3'
OFS1829	*RAD4* R	5'-ACTTCGATGTTCCAAATGTATTCTTG-3'
OFS1830	*RRI1* F	5'-CTAAAGGAAAGGTACACCGTGGAA-3'
OFS1831	*RRI1* R	5'-AGATTGGCTTAGCGCATGAAA-3'
OFS1077	*PHO84* F	5'-GAAATTAACGAGCTATACCACGATGAAATC-3'
OFS1078	*PHO84* R	5'-CATGTTGAAGTTGAGATGGGCTGG-3'
OFS2217	*RAD4* Sp6 antisense probe	5'-CGGATTTAGGTGACACTATAGAATACAAAAAGCGCCCCTGAGTAG-3'
OFS2218	*RAD4* T7 sense probe	5'-CGGTAATACGACTCACTATAGGGAGAGTGGGAGTTCCTCATCTTTCTG-3'
OFS2216	*GYP5* T7 sense probe	5'-CGGTAATACGACTCACTATAGGGAGACGTCCGGGTTCGGTAAATTT-3'
OFS2160	*GYP5* Sp6 antisense probe	5'-CGGATTTAGGTGACACTATAGAATACGGCATGGGGTTCATTGCAG-3'
OFS1961	Nrd1-FRB F2	5'-GCTCAATTGAATTCTTTGATGAATATGCTTAACCAACAGCAGCAGCAACAACAACAAAGCCGGATCCCCGGGTTAATTAA-3'
OFS1962	Nrd1-FRB R1	5'-GGAAAAAACAGAAATTATATATAGAGGTAGATTAGTTTTATGTACTATGAGCAAATAAAGGAATTCGAGCTCGTTTAAAC-3'
OFS711	Rrp6-FRB F2	5'-GAGGCCTGCCGCCAAAGGTAAGAATCTGTCATTTAAAAGGCGGATCCCCGGGTTAATTAA-3'
OFS710	Rrp6-FRB R1	5'-GAAAATTACCATAATTTATAAATAAAAAAATACGCTTGTTTTACATAAGAATTCGAGCTCGTTTAAAC-3'

Table S2: List of genes in each Class

Common Name	Cluster Name	Robustness	Class Number
BDH2	PHO84-like	core	Class (I)
HSP26	PHO84-like	core	Class (I)
PHO5	PHO84-like	core	Class (I)
YBR116C, TKL2	PHO84-like	core	Class (I)
SSE2	PHO84-like	core	Class (I)
HBT1	PHO84-like	core	Class (I)
NDE2	PHO84-like	core	Class (I)
AMS1	PHO84-like	core	Class (I)
FMP48	PHO84-like	core	Class (I)
CTT1	PHO84-like	core	Class (I)
RIM4	PHO84-like	core	Class (I)
SPL2	PHO84-like	core	Class (I)
SDP1	PHO84-like	core	Class (I)
TIR3	PHO84-like	core	Class (I)
YJL163C	PHO84-like	core	Class (I)
SIP4	PHO84-like	core	Class (I)
BOP2	PHO84-like	core	Class (I)
PHO84	PHO84-like	core	Class (I)
CYB2	PHO84-like	core	Class (I)
HXT2	PHO84-like	core	Class (I)
SPG4	PHO84-like	core	Class (I)
YNL195C	PHO84-like	core	Class (I)
YNL194C	PHO84-like	core	Class (I)
PHM7	PHO84-like	core	Class (I)
GSP2	PHO84-like	core	Class (I)
GYP5	PHO84-like	core	Class (I)
YPL230W	PHO84-like	core	Class (I)
CSR2	PHO84-like	core	Class (I)
GLC3	PHO84-like	other	Class (I)
YER079W	PHO84-like	other	Class (I)
MNN4	PHO84-like	other	Class (I)
MSC1	PHO84-like	other	Class (I)
ISF1	PHO84-like	other	Class (I)
MLS1	PHO84-like	other	Class (I)
GAC1	PHO84-like	other	Class (I)
GDH3	AS-affecting-sense	core	Class (II)
UGA2	AS-affecting-sense	core	Class (II)
REG2	AS-affecting-sense	core	Class (II)
ATG14	AS-affecting-sense	core	Class (II)
YDL237W	AS-affecting-sense	core	Class (II)
RRI1	AS-affecting-sense	core	Class (II)
PRR2	AS-affecting-sense	core	Class (II)
ARF2	AS-affecting-sense	core	Class (II)
TMN2	AS-affecting-sense	core	Class (II)
TFB3	AS-affecting-sense	core	Class (II)
YDR506C	AS-affecting-sense	core	Class (II)
MEI4	AS-affecting-sense	core	Class (II)
GIP2	AS-affecting-sense	core	Class (II)
MOT2	AS-affecting-sense	core	Class (II)
VTC1	AS-affecting-sense	core	Class (II)
YER184C	AS-affecting-sense	core	Class (II)
ATG18	AS-affecting-sense	core	Class (II)
ZIP2	AS-affecting-sense	core	Class (II)
POX1	AS-affecting-sense	core	Class (II)
YGL185C	AS-affecting-sense	core	Class (II)

YGL146C	AS-affecting-sense	core	Class (II)
MMS2	AS-affecting-sense	core	Class (II)
ALG13	AS-affecting-sense	core	Class (II)
UGA1	AS-affecting-sense	core	Class (II)
SPR3	AS-affecting-sense	core	Class (II)
SPT4	AS-affecting-sense	core	Class (II)
VPS62	AS-affecting-sense	core	Class (II)
XKS1	AS-affecting-sense	core	Class (II)
LSC2	AS-affecting-sense	core	Class (II)
YSC84	AS-affecting-sense	core	Class (II)
SAM35	AS-affecting-sense	core	Class (II)
HXT5	AS-affecting-sense	core	Class (II)
QDR1	AS-affecting-sense	core	Class (II)
BCY1	AS-affecting-sense	core	Class (II)
YIR014W	AS-affecting-sense	core	Class (II)
DAL4	AS-affecting-sense	core	Class (II)
DAL3	AS-affecting-sense	core	Class (II)
PHO90	AS-affecting-sense	core	Class (II)
TPK1	AS-affecting-sense	core	Class (II)
TAX4	AS-affecting-sense	core	Class (II)
MHP1	AS-affecting-sense	core	Class (II)
TES1	AS-affecting-sense	core	Class (II)
YJR030C	AS-affecting-sense	core	Class (II)
YJR096W	AS-affecting-sense	core	Class (II)
CAF17	AS-affecting-sense	core	Class (II)
LAP4	AS-affecting-sense	core	Class (II)
YKL071W	AS-affecting-sense	core	Class (II)
YKR011C	AS-affecting-sense	core	Class (II)
YLL023C	AS-affecting-sense	core	Class (II)
YLR001C	AS-affecting-sense	core	Class (II)
STU2	AS-affecting-sense	core	Class (II)
XYL2	AS-affecting-sense	core	Class (II)
SYM1, YLR252W	AS-affecting-sense	core	Class (II)
PDR8	AS-affecting-sense	core	Class (II)
HMG2	AS-affecting-sense	core	Class (II)
RIF2	AS-affecting-sense	core	Class (II)
PGM2	AS-affecting-sense	core	Class (II)
JLP2	AS-affecting-sense	core	Class (II)
ALD3	AS-affecting-sense	core	Class (II)
RGM1	AS-affecting-sense	core	Class (II)
SPG5	AS-affecting-sense	core	Class (II)
YNL193W	AS-affecting-sense	core	Class (II)
YNL045W	AS-affecting-sense	core	Class (II)
GAS4	AS-affecting-sense	core	Class (II)
AUS1	AS-affecting-sense	core	Class (II)
FMP40	AS-affecting-sense	core	Class (II)
DBP1	AS-affecting-sense	core	Class (II)
JID1	AS-affecting-sense	core	Class (II)
GPH1, YPR160W-A	AS-affecting-sense	core	Class (II)
BDH1	AS-no-effect-on-sense	core	Class (III)
ECM1	AS-no-effect-on-sense	core	Class (III)
SPC72	AS-no-effect-on-sense	core	Class (III)
PTA1	AS-no-effect-on-sense	core	Class (III)
LTE1	AS-no-effect-on-sense	core	Class (III)
ATS1	AS-no-effect-on-sense	core	Class (III)
SYN8	AS-no-effect-on-sense	core	Class (III)
SPO7	AS-no-effect-on-sense	core	Class (III)
BUD14	AS-no-effect-on-sense	core	Class (III)

BRN1	AS-no-effect-on-sense	core	Class (III)
YBL086C	AS-no-effect-on-sense	core	Class (III)
SSA3, AAR2	AS-no-effect-on-sense	core	Class (III)
FUI1	AS-no-effect-on-sense	core	Class (III)
POL12	AS-no-effect-on-sense	core	Class (III)
STU1	AS-no-effect-on-sense	core	Class (III)
HEK2	AS-no-effect-on-sense	core	Class (III)
YBL028C	AS-no-effect-on-sense	core	Class (III)
HAP3	AS-no-effect-on-sense	core	Class (III)
RCR1	AS-no-effect-on-sense	core	Class (III)
YBR030W	AS-no-effect-on-sense	core	Class (III)
PDX3	AS-no-effect-on-sense	core	Class (III)
SCO1	AS-no-effect-on-sense	core	Class (III)
TCM62	AS-no-effect-on-sense	core	Class (III)
YBR062C	AS-no-effect-on-sense	core	Class (III)
NRG2	AS-no-effect-on-sense	core	Class (III)
ALG14	AS-no-effect-on-sense	core	Class (III)
RDH54	AS-no-effect-on-sense	core	Class (III)
RFC5	AS-no-effect-on-sense	core	Class (III)
NHP6B, YBR090C	AS-no-effect-on-sense	core	Class (III)
PHO3	AS-no-effect-on-sense	core	Class (III)
SIF2	AS-no-effect-on-sense	core	Class (III)
MRPL36	AS-no-effect-on-sense	core	Class (III)
NPL4	AS-no-effect-on-sense	core	Class (III)
YBR197C	AS-no-effect-on-sense	core	Class (III)
SWC5	AS-no-effect-on-sense	core	Class (III)
TSC10	AS-no-effect-on-sense	core	Class (III)
REI1	AS-no-effect-on-sense	core	Class (III)
KAR4	AS-no-effect-on-sense	core	Class (III)
STP22	AS-no-effect-on-sense	core	Class (III)
POL4	AS-no-effect-on-sense	core	Class (III)
BPH1	AS-no-effect-on-sense	core	Class (III)
BUD23	AS-no-effect-on-sense	core	Class (III)
YIH1	AS-no-effect-on-sense	core	Class (III)
RSA4	AS-no-effect-on-sense	core	Class (III)
SOL2	AS-no-effect-on-sense	core	Class (III)
SRB8	AS-no-effect-on-sense	core	Class (III)
DTD1	AS-no-effect-on-sense	core	Class (III)
MRPL11	AS-no-effect-on-sense	core	Class (III)
ASF2	AS-no-effect-on-sense	core	Class (III)
LYS20	AS-no-effect-on-sense	core	Class (III)
PCL9	AS-no-effect-on-sense	core	Class (III)
FAP7	AS-no-effect-on-sense	core	Class (III)
YDL129W	AS-no-effect-on-sense	core	Class (III)
PCL2	AS-no-effect-on-sense	core	Class (III)
LUC7	AS-no-effect-on-sense	core	Class (III)
THI3	AS-no-effect-on-sense	core	Class (III)
SLC1	AS-no-effect-on-sense	core	Class (III)
PUS9	AS-no-effect-on-sense	core	Class (III)
SLM3	AS-no-effect-on-sense	core	Class (III)
RAD28	AS-no-effect-on-sense	core	Class (III)
NRG1	AS-no-effect-on-sense	core	Class (III)
TPI1	AS-no-effect-on-sense	core	Class (III)
YDR051C	AS-no-effect-on-sense	core	Class (III)
RAD55	AS-no-effect-on-sense	core	Class (III)
SSS1	AS-no-effect-on-sense	core	Class (III)
UBC13	AS-no-effect-on-sense	core	Class (III)
GRX3	AS-no-effect-on-sense	core	Class (III)

BMH2	AS-no-effect-on-sense	core	Class (III)
APC4, YDR118W-A	AS-no-effect-on-sense	core	Class (III)
YDR119W	AS-no-effect-on-sense	core	Class (III)
SWF1	AS-no-effect-on-sense	core	Class (III)
RPA14, YDR157W	AS-no-effect-on-sense	core	Class (III)
YDR179W-A	AS-no-effect-on-sense	core	Class (III)
MSC2	AS-no-effect-on-sense	core	Class (III)
UPC2	AS-no-effect-on-sense	core	Class (III)
HTA1	AS-no-effect-on-sense	core	Class (III)
EXG2	AS-no-effect-on-sense	core	Class (III)
DON1	AS-no-effect-on-sense	core	Class (III)
RNH202	AS-no-effect-on-sense	core	Class (III)
BFR2	AS-no-effect-on-sense	core	Class (III)
HNT2	AS-no-effect-on-sense	core	Class (III)
PEP7	AS-no-effect-on-sense	core	Class (III)
YCG1	AS-no-effect-on-sense	core	Class (III)
YDR336W	AS-no-effect-on-sense	core	Class (III)
SVF1	AS-no-effect-on-sense	core	Class (III)
YDR352W	AS-no-effect-on-sense	core	Class (III)
YPR1	AS-no-effect-on-sense	core	Class (III)
SPT3	AS-no-effect-on-sense	core	Class (III)
NCB2	AS-no-effect-on-sense	core	Class (III)
UTP5	AS-no-effect-on-sense	core	Class (III)
HPT1	AS-no-effect-on-sense	core	Class (III)
YDR415C	AS-no-effect-on-sense	core	Class (III)
SYF1	AS-no-effect-on-sense	core	Class (III)
DYN2	AS-no-effect-on-sense	core	Class (III)
SNX41	AS-no-effect-on-sense	core	Class (III)
GPI19, THI74, LRS4	AS-no-effect-on-sense	core	Class (III)
SSN2	AS-no-effect-on-sense	core	Class (III)
PFA5	AS-no-effect-on-sense	core	Class (III)
STP1	AS-no-effect-on-sense	core	Class (III)
JIP4	AS-no-effect-on-sense	core	Class (III)
YDR476C	AS-no-effect-on-sense	core	Class (III)
DIG2	AS-no-effect-on-sense	core	Class (III)
VPS60	AS-no-effect-on-sense	core	Class (III)
IZH1	AS-no-effect-on-sense	core	Class (III)
SEC20	AS-no-effect-on-sense	core	Class (III)
GIN4	AS-no-effect-on-sense	core	Class (III)
APA2	AS-no-effect-on-sense	core	Class (III)
RMD6	AS-no-effect-on-sense	core	Class (III)
VMA8	AS-no-effect-on-sense	core	Class (III)
YEL047C	AS-no-effect-on-sense	core	Class (III)
IES6	AS-no-effect-on-sense	core	Class (III)
ANP1	AS-no-effect-on-sense	core	Class (III)
GEA2	AS-no-effect-on-sense	core	Class (III)
PRE1	AS-no-effect-on-sense	core	Class (III)
BIM1	AS-no-effect-on-sense	core	Class (III)
FCY2	AS-no-effect-on-sense	core	Class (III)
PET117	AS-no-effect-on-sense	core	Class (III)
PCL6	AS-no-effect-on-sense	core	Class (III)
FCY21	AS-no-effect-on-sense	core	Class (III)
YER078C	AS-no-effect-on-sense	core	Class (III)
GET2	AS-no-effect-on-sense	core	Class (III)
PUP3	AS-no-effect-on-sense	core	Class (III)
RPS8B	AS-no-effect-on-sense	core	Class (III)
LCP5	AS-no-effect-on-sense	core	Class (III)
YER128W	AS-no-effect-on-sense	core	Class (III)

PMD1	AS-no-effect-on-sense	core	Class (III)
YER139C	AS-no-effect-on-sense	core	Class (III)
YER140W	AS-no-effect-on-sense	core	Class (III)
FTR1	AS-no-effect-on-sense	core	Class (III)
YER156C	AS-no-effect-on-sense	core	Class (III)
RAD4	AS-no-effect-on-sense	core	Class (III)
BLM10	AS-no-effect-on-sense	core	Class (III)
LOC1	AS-no-effect-on-sense	core	Class (III)
RPL2A	AS-no-effect-on-sense	core	Class (III)
RPL29, YFR032C-B	AS-no-effect-on-sense	core	Class (III)
NIF3	AS-no-effect-on-sense	core	Class (III)
KIP3	AS-no-effect-on-sense	core	Class (III)
YPT32	AS-no-effect-on-sense	core	Class (III)
EMP24	AS-no-effect-on-sense	core	Class (III)
YGL176C	AS-no-effect-on-sense	core	Class (III)
SAE2	AS-no-effect-on-sense	core	Class (III)
NUP49	AS-no-effect-on-sense	core	Class (III)
RAD54	AS-no-effect-on-sense	core	Class (III)
PEX14	AS-no-effect-on-sense	core	Class (III)
GPI10	AS-no-effect-on-sense	core	Class (III)
RSM23, CWC23	AS-no-effect-on-sense	core	Class (III)
CDC20	AS-no-effect-on-sense	core	Class (III)
MLC1	AS-no-effect-on-sense	core	Class (III)
SEH1	AS-no-effect-on-sense	core	Class (III)
SRM1	AS-no-effect-on-sense	core	Class (III)
SPC105	AS-no-effect-on-sense	core	Class (III)
MPS2	AS-no-effect-on-sense	core	Class (III)
YBP2	AS-no-effect-on-sense	core	Class (III)
SNU71	AS-no-effect-on-sense	core	Class (III)
YGR017W	AS-no-effect-on-sense	core	Class (III)
PEF1	AS-no-effect-on-sense	core	Class (III)
COX18	AS-no-effect-on-sense	core	Class (III)
UPF3	AS-no-effect-on-sense	core	Class (III)
PAC10	AS-no-effect-on-sense	core	Class (III)
PRP31	AS-no-effect-on-sense	core	Class (III)
YGR093W	AS-no-effect-on-sense	core	Class (III)
TPC1	AS-no-effect-on-sense	core	Class (III)
YGR125W	AS-no-effect-on-sense	core	Class (III)
YGR151C, RSR1	AS-no-effect-on-sense	core	Class (III)
PTI1	AS-no-effect-on-sense	core	Class (III)
RBG2	AS-no-effect-on-sense	core	Class (III)
PBP1	AS-no-effect-on-sense	core	Class (III)
RNR4	AS-no-effect-on-sense	core	Class (III)
HIP1	AS-no-effect-on-sense	core	Class (III)
PDX1	AS-no-effect-on-sense	core	Class (III)
YGR210C	AS-no-effect-on-sense	core	Class (III)
YAP1802	AS-no-effect-on-sense	core	Class (III)
YGR251W	AS-no-effect-on-sense	core	Class (III)
BUD32	AS-no-effect-on-sense	core	Class (III)
HUA1	AS-no-effect-on-sense	core	Class (III)
ERV29	AS-no-effect-on-sense	core	Class (III)
WSC4	AS-no-effect-on-sense	core	Class (III)
APM2	AS-no-effect-on-sense	core	Class (III)
OTU2	AS-no-effect-on-sense	core	Class (III)
MRS11	AS-no-effect-on-sense	core	Class (III)
RPL27A	AS-no-effect-on-sense	core	Class (III)
DIA4	AS-no-effect-on-sense	core	Class (III)
RRM3	AS-no-effect-on-sense	core	Class (III)

VMA10	AS-no-effect-on-sense	core	Class (III)
AAP1	AS-no-effect-on-sense	core	Class (III)
SMF2	AS-no-effect-on-sense	core	Class (III)
MED6	AS-no-effect-on-sense	core	Class (III)
RPP1	AS-no-effect-on-sense	core	Class (III)
RRP3	AS-no-effect-on-sense	core	Class (III)
HTD2	AS-no-effect-on-sense	core	Class (III)
NOP10	AS-no-effect-on-sense	core	Class (III)
MSR1	AS-no-effect-on-sense	core	Class (III)
BIG1	AS-no-effect-on-sense	core	Class (III)
ERP5	AS-no-effect-on-sense	core	Class (III)
LSM12	AS-no-effect-on-sense	core	Class (III)
SPS100	AS-no-effect-on-sense	core	Class (III)
RPL42B	AS-no-effect-on-sense	core	Class (III)
DSE2	AS-no-effect-on-sense	core	Class (III)
CDC23	AS-no-effect-on-sense	core	Class (III)
MTG2	AS-no-effect-on-sense	core	Class (III)
DBP8	AS-no-effect-on-sense	core	Class (III)
STB5	AS-no-effect-on-sense	core	Class (III)
YHR182W	AS-no-effect-on-sense	core	Class (III)
RRD1	AS-no-effect-on-sense	core	Class (III)
CCT2	AS-no-effect-on-sense	core	Class (III)
CSM2	AS-no-effect-on-sense	core	Class (III)
RSM25	AS-no-effect-on-sense	core	Class (III)
SDS3	AS-no-effect-on-sense	core	Class (III)
YIL083C	AS-no-effect-on-sense	core	Class (III)
SER33	AS-no-effect-on-sense	core	Class (III)
SPO22	AS-no-effect-on-sense	core	Class (III)
MMF1	AS-no-effect-on-sense	core	Class (III)
FAF1	AS-no-effect-on-sense	core	Class (III)
SNL1	AS-no-effect-on-sense	core	Class (III)
CFD1	AS-no-effect-on-sense	core	Class (III)
MPH1	AS-no-effect-on-sense	core	Class (III)
SQT1	AS-no-effect-on-sense	core	Class (III)
MRS1	AS-no-effect-on-sense	core	Class (III)
PRP21	AS-no-effect-on-sense	core	Class (III)
GON7	AS-no-effect-on-sense	core	Class (III)
INO1	AS-no-effect-on-sense	core	Class (III)
YJL150W, YJL149W	AS-no-effect-on-sense	core	Class (III)
MRS3	AS-no-effect-on-sense	core	Class (III)
TRK1	AS-no-effect-on-sense	core	Class (III)
YJL127C-B	AS-no-effect-on-sense	core	Class (III)
SPT10	AS-no-effect-on-sense	core	Class (III)
LSM1	AS-no-effect-on-sense	core	Class (III)
PRY1	AS-no-effect-on-sense	core	Class (III)
YJL062W-A	AS-no-effect-on-sense	core	Class (III)
IRC8	AS-no-effect-on-sense	core	Class (III)
RTT101	AS-no-effect-on-sense	core	Class (III)
RNR2	AS-no-effect-on-sense	core	Class (III)
RPC17	AS-no-effect-on-sense	core	Class (III)
AVT1	AS-no-effect-on-sense	core	Class (III)
POL31	AS-no-effect-on-sense	core	Class (III)
GPI14	AS-no-effect-on-sense	core	Class (III)
GEA1	AS-no-effect-on-sense	core	Class (III)
ISY1	AS-no-effect-on-sense	core	Class (III)
BFA1	AS-no-effect-on-sense	core	Class (III)
RPA12	AS-no-effect-on-sense	core	Class (III)
OPI3	AS-no-effect-on-sense	core	Class (III)

MOG1	AS-no-effect-on-sense	core	Class (III)
YJR129C	AS-no-effect-on-sense	core	Class (III)
MCM22	AS-no-effect-on-sense	core	Class (III)
DPH2	AS-no-effect-on-sense	core	Class (III)
ASH1	AS-no-effect-on-sense	core	Class (III)
LOT5	AS-no-effect-on-sense	core	Class (III)
LTV1	AS-no-effect-on-sense	core	Class (III)
RRN3	AS-no-effect-on-sense	core	Class (III)
YKL115C, APN1	AS-no-effect-on-sense	core	Class (III)
RAD27	AS-no-effect-on-sense	core	Class (III)
UTP11	AS-no-effect-on-sense	core	Class (III)
MIF2	AS-no-effect-on-sense	core	Class (III)
DHR2	AS-no-effect-on-sense	core	Class (III)
YKL069W	AS-no-effect-on-sense	core	Class (III)
MPE1	AS-no-effect-on-sense	core	Class (III)
OAR1	AS-no-effect-on-sense	core	Class (III)
TUL1	AS-no-effect-on-sense	core	Class (III)
TFA1	AS-no-effect-on-sense	core	Class (III)
MEH1	AS-no-effect-on-sense	core	Class (III)
BCH2	AS-no-effect-on-sense	core	Class (III)
SPC34	AS-no-effect-on-sense	core	Class (III)
NAP1	AS-no-effect-on-sense	core	Class (III)
RPS21A	AS-no-effect-on-sense	core	Class (III)
GLG1	AS-no-effect-on-sense	core	Class (III)
CCP1	AS-no-effect-on-sense	core	Class (III)
MSA2	AS-no-effect-on-sense	core	Class (III)
PCC1	AS-no-effect-on-sense	core	Class (III)
PRP19	AS-no-effect-on-sense	core	Class (III)
GPI13	AS-no-effect-on-sense	core	Class (III)
HIF1	AS-no-effect-on-sense	core	Class (III)
PPR1	AS-no-effect-on-sense	core	Class (III)
IZH3	AS-no-effect-on-sense	core	Class (III)
RAD5	AS-no-effect-on-sense	core	Class (III)
RIC1	AS-no-effect-on-sense	core	Class (III)
TRX1	AS-no-effect-on-sense	core	Class (III)
YLR049C	AS-no-effect-on-sense	core	Class (III)
YLR050C	AS-no-effect-on-sense	core	Class (III)
FCF2	AS-no-effect-on-sense	core	Class (III)
OSW2	AS-no-effect-on-sense	core	Class (III)
YLR063W	AS-no-effect-on-sense	core	Class (III)
BUD20	AS-no-effect-on-sense	core	Class (III)
RPL10	AS-no-effect-on-sense	core	Class (III)
YLR091W	AS-no-effect-on-sense	core	Class (III)
GIS3	AS-no-effect-on-sense	core	Class (III)
ICT1	AS-no-effect-on-sense	core	Class (III)
YLR099W-A	AS-no-effect-on-sense	core	Class (III)
RNH203	AS-no-effect-on-sense	core	Class (III)
IDP2	AS-no-effect-on-sense	core	Class (III)
YLR177W	AS-no-effect-on-sense	core	Class (III)
YLR194C	AS-no-effect-on-sense	core	Class (III)
NMT1	AS-no-effect-on-sense	core	Class (III)
MSC3	AS-no-effect-on-sense	core	Class (III)
YLR222C-A, IFH1	AS-no-effect-on-sense	core	Class (III)
EST1	AS-no-effect-on-sense	core	Class (III)
CDD1	AS-no-effect-on-sense	core	Class (III)
CDC46	AS-no-effect-on-sense	core	Class (III)
STT4	AS-no-effect-on-sense	core	Class (III)
CDC3	AS-no-effect-on-sense	core	Class (III)

CWC24	AS-no-effect-on-sense	core	Class (III)
YLR352W	AS-no-effect-on-sense	core	Class (III)
ILV5	AS-no-effect-on-sense	core	Class (III)
YLR361C-A	AS-no-effect-on-sense	core	Class (III)
ROM2	AS-no-effect-on-sense	core	Class (III)
IKI3, SWC7	AS-no-effect-on-sense	core	Class (III)
VPS33	AS-no-effect-on-sense	core	Class (III)
AFG2	AS-no-effect-on-sense	core	Class (III)
YLR426W	AS-no-effect-on-sense	core	Class (III)
YLR437C	AS-no-effect-on-sense	core	Class (III)
FPR4	AS-no-effect-on-sense	core	Class (III)
TAF8	AS-no-effect-on-sense	core	Class (III)
ZDS2	AS-no-effect-on-sense	core	Class (III)
MDM1	AS-no-effect-on-sense	core	Class (III)
GIM5	AS-no-effect-on-sense	core	Class (III)
DUS1	AS-no-effect-on-sense	core	Class (III)
COG8	AS-no-effect-on-sense	core	Class (III)
PIF1	AS-no-effect-on-sense	core	Class (III)
SPC2	AS-no-effect-on-sense	core	Class (III)
SUR7	AS-no-effect-on-sense	core	Class (III)
YML037C	AS-no-effect-on-sense	core	Class (III)
USA1	AS-no-effect-on-sense	core	Class (III)
TAF11	AS-no-effect-on-sense	core	Class (III)
YML007C-A	AS-no-effect-on-sense	core	Class (III)
SUB1	AS-no-effect-on-sense	core	Class (III)
RIM9, AEP1	AS-no-effect-on-sense	core	Class (III)
NAT4	AS-no-effect-on-sense	core	Class (III)
MED11	AS-no-effect-on-sense	core	Class (III)
RPL15B	AS-no-effect-on-sense	core	Class (III)
PKR1	AS-no-effect-on-sense	core	Class (III)
CIN4	AS-no-effect-on-sense	core	Class (III)
RPL13B	AS-no-effect-on-sense	core	Class (III)
TIF34	AS-no-effect-on-sense	core	Class (III)
TPP1	AS-no-effect-on-sense	core	Class (III)
INP2	AS-no-effect-on-sense	core	Class (III)
YMR178W	AS-no-effect-on-sense	core	Class (III)
ADD37	AS-no-effect-on-sense	core	Class (III)
CIK1	AS-no-effect-on-sense	core	Class (III)
ERG2	AS-no-effect-on-sense	core	Class (III)
HFA1	AS-no-effect-on-sense	core	Class (III)
FSH2	AS-no-effect-on-sense	core	Class (III)
BCH1	AS-no-effect-on-sense	core	Class (III)
YHM2	AS-no-effect-on-sense	core	Class (III)
YMR244C-A	AS-no-effect-on-sense	core	Class (III)
GTO3	AS-no-effect-on-sense	core	Class (III)
YMR259C	AS-no-effect-on-sense	core	Class (III)
RIT1	AS-no-effect-on-sense	core	Class (III)
GOT1	AS-no-effect-on-sense	core	Class (III)
ATM1	AS-no-effect-on-sense	core	Class (III)
ADH2	AS-no-effect-on-sense	core	Class (III)
VNX1	AS-no-effect-on-sense	core	Class (III)
ZIM17	AS-no-effect-on-sense	core	Class (III)
TRF5	AS-no-effect-on-sense	core	Class (III)
RFC3	AS-no-effect-on-sense	core	Class (III)
POP3	AS-no-effect-on-sense	core	Class (III)
PDR17	AS-no-effect-on-sense	core	Class (III)
YIF1	AS-no-effect-on-sense	core	Class (III)
POL2	AS-no-effect-on-sense	core	Class (III)

YNL247W	AS-no-effect-on-sense	core	Class (III)
NAR1	AS-no-effect-on-sense	core	Class (III)
LAP3	AS-no-effect-on-sense	core	Class (III)
JJJ1	AS-no-effect-on-sense	core	Class (III)
IPI3	AS-no-effect-on-sense	core	Class (III)
YNL181W	AS-no-effect-on-sense	core	Class (III)
RIA1	AS-no-effect-on-sense	core	Class (III)
YNL155W	AS-no-effect-on-sense	core	Class (III)
PGA2	AS-no-effect-on-sense	core	Class (III)
LSM7	AS-no-effect-on-sense	core	Class (III)
YSF3	AS-no-effect-on-sense	core	Class (III)
FYV6	AS-no-effect-on-sense	core	Class (III)
SPC98	AS-no-effect-on-sense	core	Class (III)
NOP15	AS-no-effect-on-sense	core	Class (III)
POL1	AS-no-effect-on-sense	core	Class (III)
PHO23	AS-no-effect-on-sense	core	Class (III)
EOS1	AS-no-effect-on-sense	core	Class (III)
NIS1	AS-no-effect-on-sense	core	Class (III)
VAC7	AS-no-effect-on-sense	core	Class (III)
MSG5	AS-no-effect-on-sense	core	Class (III)
YNL035C	AS-no-effect-on-sense	core	Class (III)
YNL024C-A	AS-no-effect-on-sense	core	Class (III)
YNL024C	AS-no-effect-on-sense	core	Class (III)
NRM1	AS-no-effect-on-sense	core	Class (III)
YNR018W	AS-no-effect-on-sense	core	Class (III)
RSM19	AS-no-effect-on-sense	core	Class (III)
COQ2	AS-no-effect-on-sense	core	Class (III)
PET494	AS-no-effect-on-sense	core	Class (III)
ESF2	AS-no-effect-on-sense	core	Class (III)
MED7	AS-no-effect-on-sense	core	Class (III)
YGK3	AS-no-effect-on-sense	core	Class (III)
TRM11	AS-no-effect-on-sense	core	Class (III)
RPS19A	AS-no-effect-on-sense	core	Class (III)
SKM1	AS-no-effect-on-sense	core	Class (III)
YOL097W-A	AS-no-effect-on-sense	core	Class (III)
WRS1	AS-no-effect-on-sense	core	Class (III)
COQ3	AS-no-effect-on-sense	core	Class (III)
HMI1	AS-no-effect-on-sense	core	Class (III)
AVO1	AS-no-effect-on-sense	core	Class (III)
RIB2	AS-no-effect-on-sense	core	Class (III)
MSE1	AS-no-effect-on-sense	core	Class (III)
RPB11	AS-no-effect-on-sense	core	Class (III)
HST3	AS-no-effect-on-sense	core	Class (III)
BUB3	AS-no-effect-on-sense	core	Class (III)
EXO1	AS-no-effect-on-sense	core	Class (III)
IRC23	AS-no-effect-on-sense	core	Class (III)
RAT1	AS-no-effect-on-sense	core	Class (III)
YOR052C	AS-no-effect-on-sense	core	Class (III)
YNG1	AS-no-effect-on-sense	core	Class (III)
VAM3	AS-no-effect-on-sense	core	Class (III)
RGS2	AS-no-effect-on-sense	core	Class (III)
YOR111W	AS-no-effect-on-sense	core	Class (III)
RPO31	AS-no-effect-on-sense	core	Class (III)
LEO1	AS-no-effect-on-sense	core	Class (III)
VPS17	AS-no-effect-on-sense	core	Class (III)
RUP1	AS-no-effect-on-sense	core	Class (III)
ELG1	AS-no-effect-on-sense	core	Class (III)
PNO1	AS-no-effect-on-sense	core	Class (III)

YOR152C	AS-no-effect-on-sense	core	Class (III)
ISU2	AS-no-effect-on-sense	core	Class (III)
DFR1	AS-no-effect-on-sense	core	Class (III)
YOR238W	AS-no-effect-on-sense	core	Class (III)
YOR262W	AS-no-effect-on-sense	core	Class (III)
CAF20	AS-no-effect-on-sense	core	Class (III)
FSH3	AS-no-effect-on-sense	core	Class (III)
HUA2	AS-no-effect-on-sense	core	Class (III)
RRS1	AS-no-effect-on-sense	core	Class (III)
SNU66	AS-no-effect-on-sense	core	Class (III)
KRE5	AS-no-effect-on-sense	core	Class (III)
HAP5	AS-no-effect-on-sense	core	Class (III)
SCP1	AS-no-effect-on-sense	core	Class (III)
GPB1	AS-no-effect-on-sense	core	Class (III)
ACM1	AS-no-effect-on-sense	core	Class (III)
RBD2	AS-no-effect-on-sense	core	Class (III)
YPL236C	AS-no-effect-on-sense	core	Class (III)
SSO1	AS-no-effect-on-sense	core	Class (III)
YPL229W	AS-no-effect-on-sense	core	Class (III)
IPL1	AS-no-effect-on-sense	core	Class (III)
RKM1	AS-no-effect-on-sense	core	Class (III)
YIG1	AS-no-effect-on-sense	core	Class (III)
CTI6	AS-no-effect-on-sense	core	Class (III)
TRE1	AS-no-effect-on-sense	core	Class (III)
YPL158C	AS-no-effect-on-sense	core	Class (III)
POC4	AS-no-effect-on-sense	core	Class (III)
SPP1	AS-no-effect-on-sense	core	Class (III)
HHO1	AS-no-effect-on-sense	core	Class (III)
SSU1	AS-no-effect-on-sense	core	Class (III)
YPL077C	AS-no-effect-on-sense	core	Class (III)
ALD6	AS-no-effect-on-sense	core	Class (III)
YPL056C	AS-no-effect-on-sense	core	Class (III)
LGE1	AS-no-effect-on-sense	core	Class (III)
OAZ1	AS-no-effect-on-sense	core	Class (III)
YPL041C	AS-no-effect-on-sense	core	Class (III)
YPL039W, YPL038W-A	AS-no-effect-on-sense	core	Class (III)
PHO85	AS-no-effect-on-sense	core	Class (III)
YPR011C	AS-no-effect-on-sense	core	Class (III)
ERV2	AS-no-effect-on-sense	core	Class (III)
YPR039W, TIP41	AS-no-effect-on-sense	core	Class (III)
MCM16	AS-no-effect-on-sense	core	Class (III)
TAH18	AS-no-effect-on-sense	core	Class (III)
BRR1	AS-no-effect-on-sense	core	Class (III)
UBA3	AS-no-effect-on-sense	core	Class (III)
MRL1	AS-no-effect-on-sense	core	Class (III)
PIS1	AS-no-effect-on-sense	core	Class (III)
CTF4	AS-no-effect-on-sense	core	Class (III)
MEP3	AS-no-effect-on-sense	core	Class (III)
YPR146C, YPR147C	AS-no-effect-on-sense	core	Class (III)
YPR153W	AS-no-effect-on-sense	core	Class (III)
MMS1	AS-no-effect-on-sense	core	Class (III)
MET16	AS-no-effect-on-sense	core	Class (III)
NUT2	AS-no-effect-on-sense	core	Class (III)
SMX3	AS-no-effect-on-sense	core	Class (III)
ECM13	AS-no-effect-on-sense	other	Class (III)
YBL029C-A	AS-no-effect-on-sense	other	Class (III)
CDC28	AS-no-effect-on-sense	other	Class (III)
YCR051W	AS-no-effect-on-sense	other	Class (III)

COX9	AS-no-effect-on-sense	other	Class (III)
TRS31	AS-no-effect-on-sense	other	Class (III)
SLF1	AS-no-effect-on-sense	other	Class (III)
DAM1	AS-no-effect-on-sense	other	Class (III)
YGR131W	AS-no-effect-on-sense	other	Class (III)
YGR277C	AS-no-effect-on-sense	other	Class (III)
MRPL44	AS-no-effect-on-sense	other	Class (III)
YNL254C	AS-no-effect-on-sense	other	Class (III)
TYE7	AS-no-effect-on-sense	other	Class (III)
YPL216W	AS-no-effect-on-sense	other	Class (III)
TFB2	AS-no-effect-on-sense	other	Class (III)
PRX1	no-AS-sense-affected	core	Class (IV)
YBR285W	no-AS-sense-affected	core	Class (IV)
ADY2	no-AS-sense-affected	core	Class (IV)
FMP45	no-AS-sense-affected	core	Class (IV)
UGX2	no-AS-sense-affected	core	Class (IV)
MRK1	no-AS-sense-affected	core	Class (IV)
YDR018C	no-AS-sense-affected	core	Class (IV)
FMP16	no-AS-sense-affected	core	Class (IV)
ADR1	no-AS-sense-affected	core	Class (IV)
CRF1	no-AS-sense-affected	core	Class (IV)
PHM6	no-AS-sense-affected	core	Class (IV)
PDR15, YDR406W-A	no-AS-sense-affected	core	Class (IV)
YDR461C-A	no-AS-sense-affected	core	Class (IV)
YEF1	no-AS-sense-affected	core	Class (IV)
TIR1	no-AS-sense-affected	core	Class (IV)
FAA2	no-AS-sense-affected	core	Class (IV)
YAT2	no-AS-sense-affected	core	Class (IV)
SHC1	no-AS-sense-affected	core	Class (IV)
HSP12	no-AS-sense-affected	core	Class (IV)
YFR017C	no-AS-sense-affected	core	Class (IV)
RMR1	no-AS-sense-affected	core	Class (IV)
RCK1	no-AS-sense-affected	core	Class (IV)
YGL081W	no-AS-sense-affected	core	Class (IV)
YGR053C	no-AS-sense-affected	core	Class (IV)
YGR067C	no-AS-sense-affected	core	Class (IV)
PDC6	no-AS-sense-affected	core	Class (IV)
YGR110W	no-AS-sense-affected	core	Class (IV)
YGR201C	no-AS-sense-affected	core	Class (IV)
FMP43	no-AS-sense-affected	core	Class (IV)
SOL4	no-AS-sense-affected	core	Class (IV)
YHR140W	no-AS-sense-affected	core	Class (IV)
SUC2	no-AS-sense-affected	core	Class (IV)
GUT2	no-AS-sense-affected	core	Class (IV)
YIL057C	no-AS-sense-affected	core	Class (IV)
YJL218W	no-AS-sense-affected	core	Class (IV)
GLG2	no-AS-sense-affected	core	Class (IV)
GSM1	no-AS-sense-affected	core	Class (IV)
IME1	no-AS-sense-affected	core	Class (IV)
JEN1	no-AS-sense-affected	core	Class (IV)
YKL133C	no-AS-sense-affected	core	Class (IV)
ECM4	no-AS-sense-affected	core	Class (IV)
PUT1	no-AS-sense-affected	core	Class (IV)
TFS1	no-AS-sense-affected	core	Class (IV)
ECI1	no-AS-sense-affected	core	Class (IV)
QNQ1	no-AS-sense-affected	core	Class (IV)
YLR356W	no-AS-sense-affected	core	Class (IV)
YMR084W, YMR085W	no-AS-sense-affected	core	Class (IV)

SIP18	no-AS-sense-affected	core	Class (IV)
YMR206W	no-AS-sense-affected	core	Class (IV)
GAD1	no-AS-sense-affected	core	Class (IV)
CAT8	no-AS-sense-affected	core	Class (IV)
YTP1	no-AS-sense-affected	core	Class (IV)
YPT53	no-AS-sense-affected	core	Class (IV)
YNL092W	no-AS-sense-affected	core	Class (IV)
NCE103	no-AS-sense-affected	core	Class (IV)
ATO2	no-AS-sense-affected	core	Class (IV)
HPF1	no-AS-sense-affected	core	Class (IV)
MDH2	no-AS-sense-affected	core	Class (IV)
IZH4	no-AS-sense-affected	core	Class (IV)
YOR019W	no-AS-sense-affected	core	Class (IV)
DCS2	no-AS-sense-affected	core	Class (IV)
YOR338W	no-AS-sense-affected	core	Class (IV)
YOR352W	no-AS-sense-affected	core	Class (IV)
ALD4	no-AS-sense-affected	core	Class (IV)
FIT2	no-AS-sense-affected	core	Class (IV)
GRE1	no-AS-sense-affected	core	Class (IV)
UIP4	no-AS-sense-affected	core	Class (IV)
OYE3	no-AS-sense-affected	core	Class (IV)
PXA1	no-AS-sense-affected	core	Class (IV)
ODC1	no-AS-sense-affected	core	Class (IV)
YPL119C-A	no-AS-sense-affected	core	Class (IV)
CIT3	no-AS-sense-affected	core	Class (IV)
PDH1	no-AS-sense-affected	core	Class (IV)
YPR015C	no-AS-sense-affected	core	Class (IV)
ATH1	no-AS-sense-affected	core	Class (IV)
GDB1	no-AS-sense-affected	core	Class (IV)
NTH2	no-AS-sense-affected	other	Class (IV)
BIO2	no-AS-sense-affected	other	Class (IV)
YOL083W	no-AS-sense-affected	other	Class (IV)
BAG7	no-AS-sense-affected	other	Class (IV)
SEO1	nothing	core	Class (V)
CNE1	nothing	core	Class (V)
FLC2	nothing	core	Class (V)
GEM1	nothing	core	Class (V)
YAL046C	nothing	core	Class (V)
ERV46	nothing	core	Class (V)
CYC3	nothing	core	Class (V)
CDC19	nothing	core	Class (V)
FUN12	nothing	core	Class (V)
GIP4	nothing	core	Class (V)
FRT2	nothing	core	Class (V)
MAK16	nothing	core	Class (V)
PMT2	nothing	core	Class (V)
FUN26	nothing	core	Class (V)
CCR4	nothing	core	Class (V)
PSK1	nothing	core	Class (V)
DEP1	nothing	core	Class (V)
CYS3	nothing	core	Class (V)
SSA1	nothing	core	Class (V)
SEN34	nothing	core	Class (V)
CDC15	nothing	core	Class (V)
YAT1	nothing	core	Class (V)
SRO77	nothing	core	Class (V)
PKC1	nothing	core	Class (V)
RTG3	nothing	core	Class (V)

SFT2	nothing	core	Class (V)
ECM21	nothing	core	Class (V)
ATP1	nothing	core	Class (V)
RPL32	nothing	core	Class (V)
SCS22	nothing	core	Class (V)
MAP2	nothing	core	Class (V)
MRP21	nothing	core	Class (V)
RPL23A	nothing	core	Class (V)
BOI1	nothing	core	Class (V)
YBL083C, ALG3	nothing	core	Class (V)
NUP170	nothing	core	Class (V)
ATG8	nothing	core	Class (V)
ILS1	nothing	core	Class (V)
KTI11	nothing	core	Class (V)
AST1, YBL068W-A	nothing	core	Class (V)
PRS4	nothing	core	Class (V)
UBP13	nothing	core	Class (V)
SEF1	nothing	core	Class (V)
KIP1	nothing	core	Class (V)
SKT5	nothing	core	Class (V)
SAS3	nothing	core	Class (V)
YBL039W-B	nothing	core	Class (V)
URA7, YBL039C-A	nothing	core	Class (V)
MRPL16	nothing	core	Class (V)
RIB1	nothing	core	Class (V)
YBL029W	nothing	core	Class (V)
RPL19B	nothing	core	Class (V)
LSM2	nothing	core	Class (V)
RRN10	nothing	core	Class (V)
NCL1	nothing	core	Class (V)
MCM2	nothing	core	Class (V)
PIM1	nothing	core	Class (V)
PEP1	nothing	core	Class (V)
RRN6	nothing	core	Class (V)
FMT1	nothing	core	Class (V)
ALK2	nothing	core	Class (V)
SLA1	nothing	core	Class (V)
RER2	nothing	core	Class (V)
DSF2	nothing	core	Class (V)
FLR1	nothing	core	Class (V)
HHF1	nothing	core	Class (V)
GRX6	nothing	core	Class (V)
MNN2	nothing	core	Class (V)
GAL7, GAL10	nothing	core	Class (V)
GAL1	nothing	core	Class (V)
CDS1	nothing	core	Class (V)
RPL4A	nothing	core	Class (V)
CHS2	nothing	core	Class (V)
ATP3	nothing	core	Class (V)
FAT1	nothing	core	Class (V)
CST26	nothing	core	Class (V)
QDR3	nothing	core	Class (V)
FMP23	nothing	core	Class (V)
RPS11B	nothing	core	Class (V)
REB1	nothing	core	Class (V)
YBR053C	nothing	core	Class (V)
YBR056W	nothing	core	Class (V)
MUM2	nothing	core	Class (V)

TSC3	nothing	core	Class (V)
ORC2	nothing	core	Class (V)
TRM7	nothing	core	Class (V)
TIP1	nothing	core	Class (V)
BAP2	nothing	core	Class (V)
TAT1	nothing	core	Class (V)
YBR071W	nothing	core	Class (V)
YBR074W	nothing	core	Class (V)
SEC18	nothing	core	Class (V)
UBC4	nothing	core	Class (V)
TEC1	nothing	core	Class (V)
AAC3	nothing	core	Class (V)
YBR085C-A	nothing	core	Class (V)
IST2	nothing	core	Class (V)
POL30	nothing	core	Class (V)
YBR096W	nothing	core	Class (V)
VPS15	nothing	core	Class (V)
EXO84	nothing	core	Class (V)
YSA1	nothing	core	Class (V)
TEF2	nothing	core	Class (V)
PTC4	nothing	core	Class (V)
SHE3	nothing	core	Class (V)
HSL7	nothing	core	Class (V)
CKS1	nothing	core	Class (V)
MEC1	nothing	core	Class (V)
MRPS9	nothing	core	Class (V)
YSW1	nothing	core	Class (V)
APD1	nothing	core	Class (V)
ICS2	nothing	core	Class (V)
AMN1	nothing	core	Class (V)
IFA38	nothing	core	Class (V)
UMP1	nothing	core	Class (V)
FZO1	nothing	core	Class (V)
RPS6B	nothing	core	Class (V)
SMP1	nothing	core	Class (V)
YBR184W	nothing	core	Class (V)
MBA1	nothing	core	Class (V)
RPS9B	nothing	core	Class (V)
PGI1	nothing	core	Class (V)
TAF5	nothing	core	Class (V)
KTR4	nothing	core	Class (V)
BEM1	nothing	core	Class (V)
CDC47	nothing	core	Class (V)
COS111	nothing	core	Class (V)
YBR204C	nothing	core	Class (V)
KTR3, YBR206W	nothing	core	Class (V)
NGR1	nothing	core	Class (V)
MET8	nothing	core	Class (V)
SDS24	nothing	core	Class (V)
YBR219C, YBR220C	nothing	core	Class (V)
PDB1	nothing	core	Class (V)
PCS60	nothing	core	Class (V)
TDP1	nothing	core	Class (V)
OM14	nothing	core	Class (V)
PBP2	nothing	core	Class (V)
YBR239C	nothing	core	Class (V)
THI2	nothing	core	Class (V)
YBR241C	nothing	core	Class (V)

ARO4	nothing	core	Class (V)
SPO23	nothing	core	Class (V)
MRPS5	nothing	core	Class (V)
DUT1	nothing	core	Class (V)
YBR261C	nothing	core	Class (V)
FMP51	nothing	core	Class (V)
MRPL37	nothing	core	Class (V)
BIT2	nothing	core	Class (V)
UBX7	nothing	core	Class (V)
PPS1	nothing	core	Class (V)
DPB3	nothing	core	Class (V)
SSH1	nothing	core	Class (V)
APE3	nothing	core	Class (V)
SNF5	nothing	core	Class (V)
VBA2	nothing	core	Class (V)
MAL33	nothing	core	Class (V)
MAL31	nothing	core	Class (V)
KRR1	nothing	core	Class (V)
LRE1	nothing	core	Class (V)
APA1	nothing	core	Class (V)
YCL045C	nothing	core	Class (V)
MGR1	nothing	core	Class (V)
PDI1, YCL041C	nothing	core	Class (V)
HIS4	nothing	core	Class (V)
BIK1	nothing	core	Class (V)
HBN1	nothing	core	Class (V)
AGP1	nothing	core	Class (V)
KCC4	nothing	core	Class (V)
DCC1	nothing	core	Class (V)
BUD3	nothing	core	Class (V)
SGF29	nothing	core	Class (V)
ILV6	nothing	core	Class (V)
LDB16	nothing	core	Class (V)
RER1	nothing	core	Class (V)
CDC10	nothing	core	Class (V)
MRPL32	nothing	core	Class (V)
YCP4	nothing	core	Class (V)
CIT2	nothing	core	Class (V)
YCR007C	nothing	core	Class (V)
SAT4	nothing	core	Class (V)
RVS161	nothing	core	Class (V)
ADP1	nothing	core	Class (V)
PGK1	nothing	core	Class (V)
HSP30	nothing	core	Class (V)
YCR023C	nothing	core	Class (V)
SLM5	nothing	core	Class (V)
RHB1	nothing	core	Class (V)
FEN2	nothing	core	Class (V)
RIM1	nothing	core	Class (V)
RPS14A	nothing	core	Class (V)
SNT1	nothing	core	Class (V)
YCR043C	nothing	core	Class (V)
PER1	nothing	core	Class (V)
ARE1	nothing	core	Class (V)
RSC6	nothing	core	Class (V)
PWP2	nothing	core	Class (V)
YCR061W	nothing	core	Class (V)
HCM1	nothing	core	Class (V)

ATG15	nothing	core	Class (V)
SSK22	nothing	core	Class (V)
ERS1	nothing	core	Class (V)
PAT1	nothing	core	Class (V)
PTC6	nothing	core	Class (V)
AHC2	nothing	core	Class (V)
CSM1	nothing	core	Class (V)
LUG1	nothing	core	Class (V)
CDC39	nothing	core	Class (V)
YCR095W-A	nothing	core	Class (V)
GIT1	nothing	core	Class (V)
YDL241W	nothing	core	Class (V)
LRG1	nothing	core	Class (V)
ADY3	nothing	core	Class (V)
GUD1	nothing	core	Class (V)
YDL233W	nothing	core	Class (V)
PTP1	nothing	core	Class (V)
SSB1	nothing	core	Class (V)
GCS1	nothing	core	Class (V)
CDC13	nothing	core	Class (V)
GDH2	nothing	core	Class (V)
NOP6	nothing	core	Class (V)
CWC2	nothing	core	Class (V)
NHP2	nothing	core	Class (V)
GLE1	nothing	core	Class (V)
RTN2	nothing	core	Class (V)
ACK1	nothing	core	Class (V)
YDL199C	nothing	core	Class (V)
SEC31	nothing	core	Class (V)
NUS1	nothing	core	Class (V)
ARF1	nothing	core	Class (V)
RBS1	nothing	core	Class (V)
TFP1	nothing	core	Class (V)
RPL41A	nothing	core	Class (V)
YDL183C	nothing	core	Class (V)
INH1	nothing	core	Class (V)
YDL180W	nothing	core	Class (V)
DLD1	nothing	core	Class (V)
YDL173W	nothing	core	Class (V)
GLT1	nothing	core	Class (V)
UGA3	nothing	core	Class (V)
DHH1	nothing	core	Class (V)
YDL156W	nothing	core	Class (V)
CLB3	nothing	core	Class (V)
MSH5	nothing	core	Class (V)
SAS10	nothing	core	Class (V)
RPC53	nothing	core	Class (V)
RPN5	nothing	core	Class (V)
YDL144C	nothing	core	Class (V)
SCM3	nothing	core	Class (V)
RGT2	nothing	core	Class (V)
PPH21	nothing	core	Class (V)
RPL41B	nothing	core	Class (V)
YDL133W	nothing	core	Class (V)
LYS21	nothing	core	Class (V)
STF1	nothing	core	Class (V)
RPP1B	nothing	core	Class (V)
VCX1	nothing	core	Class (V)

YDL124W	nothing	core	Class (V)
CYK3	nothing	core	Class (V)
NUP84	nothing	core	Class (V)
IWR1	nothing	core	Class (V)
ATG20	nothing	core	Class (V)
TMA17	nothing	core	Class (V)
YDL109C	nothing	core	Class (V)
KIN28	nothing	core	Class (V)
PHO2	nothing	core	Class (V)
GET3	nothing	core	Class (V)
RPN6	nothing	core	Class (V)
PMT1	nothing	core	Class (V)
PMT5	nothing	core	Class (V)
RAM1	nothing	core	Class (V)
RPL13A	nothing	core	Class (V)
RPP1A	nothing	core	Class (V)
MDH3	nothing	core	Class (V)
RXT3	nothing	core	Class (V)
RPL31A	nothing	core	Class (V)
BRE1	nothing	core	Class (V)
YDL073W	nothing	core	Class (V)
YET3	nothing	core	Class (V)
IDP1	nothing	core	Class (V)
PEX19	nothing	core	Class (V)
RPS29B	nothing	core	Class (V)
USO1	nothing	core	Class (V)
MBP1	nothing	core	Class (V)
PSA1	nothing	core	Class (V)
MCH1	nothing	core	Class (V)
STP4	nothing	core	Class (V)
SIT4	nothing	core	Class (V)
NPC2	nothing	core	Class (V)
PRP11	nothing	core	Class (V)
SIR2	nothing	core	Class (V)
GPR1	nothing	core	Class (V)
DBP10	nothing	core	Class (V)
PRP9	nothing	core	Class (V)
YDL027C	nothing	core	Class (V)
DIA3, YDL022C-A	nothing	core	Class (V)
GPD1	nothing	core	Class (V)
ERP3	nothing	core	Class (V)
NOP1	nothing	core	Class (V)
GRX7	nothing	core	Class (V)
APC11	nothing	core	Class (V)
PTC1	nothing	core	Class (V)
ATP16	nothing	core	Class (V)
MCD1	nothing	core	Class (V)
NHP10	nothing	core	Class (V)
RMD1	nothing	core	Class (V)
NTH1	nothing	core	Class (V)
YRB1	nothing	core	Class (V)
GAL3	nothing	core	Class (V)
SNQ2	nothing	core	Class (V)
RPL4B	nothing	core	Class (V)
PSF1	nothing	core	Class (V)
RAD61	nothing	core	Class (V)
HED1	nothing	core	Class (V)
KCS1	nothing	core	Class (V)

MIC14	nothing	core	Class (V)
PST2	nothing	core	Class (V)
MRH1	nothing	core	Class (V)
YDR034W-B	nothing	core	Class (V)
ARO3	nothing	core	Class (V)
EHD3	nothing	core	Class (V)
KRS1	nothing	core	Class (V)
RSM10	nothing	core	Class (V)
BAP3	nothing	core	Class (V)
HEM12	nothing	core	Class (V)
YDR049W	nothing	core	Class (V)
DBF4	nothing	core	Class (V)
PST1	nothing	core	Class (V)
YOS9	nothing	core	Class (V)
TGL2	nothing	core	Class (V)
MAK21	nothing	core	Class (V)
YDR061W	nothing	core	Class (V)
LCB2	nothing	core	Class (V)
YDR063W	nothing	core	Class (V)
RPS13	nothing	core	Class (V)
OCA6	nothing	core	Class (V)
PAA1	nothing	core	Class (V)
IPT1	nothing	core	Class (V)
SNF11	nothing	core	Class (V)
TPS2	nothing	core	Class (V)
PPH3	nothing	core	Class (V)
RRP1	nothing	core	Class (V)
RLI1	nothing	core	Class (V)
DNF2, YDR094W	nothing	core	Class (V)
GIS1	nothing	core	Class (V)
MSH6	nothing	core	Class (V)
TVP15	nothing	core	Class (V)
ARX1	nothing	core	Class (V)
TMS1	nothing	core	Class (V)
GSG1	nothing	core	Class (V)
YDR109C	nothing	core	Class (V)
PDS1	nothing	core	Class (V)
FIN1	nothing	core	Class (V)
YCF1	nothing	core	Class (V)
HPR1	nothing	core	Class (V)
MKC7	nothing	core	Class (V)
TAF12	nothing	core	Class (V)
EKI1	nothing	core	Class (V)
KGD2	nothing	core	Class (V)
NUM1	nothing	core	Class (V)
GIR2	nothing	core	Class (V)
YDR154C, CPR1	nothing	core	Class (V)
HOM2	nothing	core	Class (V)
SAC3	nothing	core	Class (V)
SSY1	nothing	core	Class (V)
TAF10	nothing	core	Class (V)
CDC37	nothing	core	Class (V)
STB3	nothing	core	Class (V)
SEC7	nothing	core	Class (V)
HSP42	nothing	core	Class (V)
UBC1	nothing	core	Class (V)
SCC2	nothing	core	Class (V)
SAS4	nothing	core	Class (V)

YDR183C-A, ATC1	nothing	core	Class (V)
YDR185C	nothing	core	Class (V)
YDR186C	nothing	core	Class (V)
CCT6	nothing	core	Class (V)
HST4	nothing	core	Class (V)
NUP42	nothing	core	Class (V)
MSS116	nothing	core	Class (V)
REF2	nothing	core	Class (V)
YDR196C	nothing	core	Class (V)
COQ4	nothing	core	Class (V)
EBS1	nothing	core	Class (V)
UME6	nothing	core	Class (V)
MSS4	nothing	core	Class (V)
YDR210W	nothing	core	Class (V)
TCP1	nothing	core	Class (V)
AHA1	nothing	core	Class (V)
RAD9	nothing	core	Class (V)
GTB1	nothing	core	Class (V)
YDR222W	nothing	core	Class (V)
HTB1	nothing	core	Class (V)
ADK1	nothing	core	Class (V)
SIR4	nothing	core	Class (V)
IVY1	nothing	core	Class (V)
COX20	nothing	core	Class (V)
HEM1	nothing	core	Class (V)
RTN1	nothing	core	Class (V)
LYS4	nothing	core	Class (V)
YDR239C	nothing	core	Class (V)
SNU56	nothing	core	Class (V)
MNN10	nothing	core	Class (V)
TRS23	nothing	core	Class (V)
PAM1	nothing	core	Class (V)
HSP78	nothing	core	Class (V)
YAP6	nothing	core	Class (V)
SWM1	nothing	core	Class (V)
PEX10	nothing	core	Class (V)
YDR266C	nothing	core	Class (V)
CIA1	nothing	core	Class (V)
MSW1	nothing	core	Class (V)
CCC2	nothing	core	Class (V)
GLO2	nothing	core	Class (V)
MTH1	nothing	core	Class (V)
RRP45	nothing	core	Class (V)
DPP1	nothing	core	Class (V)
YDR287W	nothing	core	Class (V)
MHR1	nothing	core	Class (V)
ATP5	nothing	core	Class (V)
PRO1	nothing	core	Class (V)
CFT1	nothing	core	Class (V)
GPI11	nothing	core	Class (V)
RSC3	nothing	core	Class (V)
CPR5	nothing	core	Class (V)
SUM1	nothing	core	Class (V)
TFB1	nothing	core	Class (V)
SSF2	nothing	core	Class (V)
PIB1	nothing	core	Class (V)
OMS1	nothing	core	Class (V)
HIM1	nothing	core	Class (V)

SWA2	nothing	core	Class (V)
DAD4	nothing	core	Class (V)
ASP1	nothing	core	Class (V)
UTP4	nothing	core	Class (V)
YSP2	nothing	core	Class (V)
SKP1	nothing	core	Class (V)
PEX3	nothing	core	Class (V)
GPI8	nothing	core	Class (V)
MRPS28	nothing	core	Class (V)
YDR338C	nothing	core	Class (V)
FCF1	nothing	core	Class (V)
YDR341C	nothing	core	Class (V)
HXT3	nothing	core	Class (V)
YPS7	nothing	core	Class (V)
TRR1	nothing	core	Class (V)
TRP4	nothing	core	Class (V)
GGA1	nothing	core	Class (V)
EAF1	nothing	core	Class (V)
BCP1	nothing	core	Class (V)
TFC6	nothing	core	Class (V)
ESC2	nothing	core	Class (V)
ESF1	nothing	core	Class (V)
YDR367W	nothing	core	Class (V)
XRS2	nothing	core	Class (V)
YDR370C	nothing	core	Class (V)
FRQ1	nothing	core	Class (V)
YDR374W-A	nothing	core	Class (V)
ARO10	nothing	core	Class (V)
ATO3	nothing	core	Class (V)
EFT2	nothing	core	Class (V)
RVS167	nothing	core	Class (V)
YDR391C	nothing	core	Class (V)
SHE9	nothing	core	Class (V)
RPT3	nothing	core	Class (V)
SXM1	nothing	core	Class (V)
URH1	nothing	core	Class (V)
DIT1	nothing	core	Class (V)
RPB7	nothing	core	Class (V)
MRP20	nothing	core	Class (V)
TRS120	nothing	core	Class (V)
RPL12B	nothing	core	Class (V)
RAD30	nothing	core	Class (V)
HKR1	nothing	core	Class (V)
CAD1	nothing	core	Class (V)
TIF35	nothing	core	Class (V)
NPL3, YDR433W	nothing	core	Class (V)
YDR444W	nothing	core	Class (V)
RPS17B	nothing	core	Class (V)
UTP6	nothing	core	Class (V)
RPS18A	nothing	core	Class (V)
YHP1	nothing	core	Class (V)
NHX1	nothing	core	Class (V)
TOM1	nothing	core	Class (V)
HEH2	nothing	core	Class (V)
MRPL28	nothing	core	Class (V)
RPL27B	nothing	core	Class (V)
PRP3	nothing	core	Class (V)
SNF1	nothing	core	Class (V)

PHO8	nothing	core	Class (V)
CWC21	nothing	core	Class (V)
RIB3	nothing	core	Class (V)
PAC11	nothing	core	Class (V)
FMP36	nothing	core	Class (V)
ITR1	nothing	core	Class (V)
PLM2	nothing	core	Class (V)
SAM2	nothing	core	Class (V)
LPP1	nothing	core	Class (V)
SPG3, PSP1	nothing	core	Class (V)
GNP1	nothing	core	Class (V)
SMT3	nothing	core	Class (V)
GRH1	nothing	core	Class (V)
EUG1	nothing	core	Class (V)
SPS1	nothing	core	Class (V)
AGE1	nothing	core	Class (V)
HLR1	nothing	core	Class (V)
QCR7	nothing	core	Class (V)
STL1	nothing	core	Class (V)
PAD1	nothing	core	Class (V)
YDR539W	nothing	core	Class (V)
YDR541C	nothing	core	Class (V)
DLD3	nothing	core	Class (V)
CAN1	nothing	core	Class (V)
PRB1	nothing	core	Class (V)
SOM1	nothing	core	Class (V)
RPL12A	nothing	core	Class (V)
RML2	nothing	core	Class (V)
YEL043W	nothing	core	Class (V)
GDA1	nothing	core	Class (V)
UTR4	nothing	core	Class (V)
RAD23	nothing	core	Class (V)
MCM3	nothing	core	Class (V)
SPF1	nothing	core	Class (V)
ECM10	nothing	core	Class (V)
BUD16	nothing	core	Class (V)
CUP5	nothing	core	Class (V)
RIP1	nothing	core	Class (V)
YEL023C	nothing	core	Class (V)
URA3	nothing	core	Class (V)
TIM9	nothing	core	Class (V)
MMS21	nothing	core	Class (V)
UBC8	nothing	core	Class (V)
GCN4	nothing	core	Class (V)
YEA6	nothing	core	Class (V)
IRC22	nothing	core	Class (V)
MNN1	nothing	core	Class (V)
FMP52	nothing	core	Class (V)
YND1	nothing	core	Class (V)
NUG1	nothing	core	Class (V)
PRP22	nothing	core	Class (V)
AFG3	nothing	core	Class (V)
SPC25	nothing	core	Class (V)
GPA2	nothing	core	Class (V)
RPN3	nothing	core	Class (V)
SRB4	nothing	core	Class (V)
PRO3	nothing	core	Class (V)
GCD11	nothing	core	Class (V)

CHO1	nothing	core	Class (V)
MIG3	nothing	core	Class (V)
SMB1	nothing	core	Class (V)
FIR1	nothing	core	Class (V)
ZRG8	nothing	core	Class (V)
ARB1	nothing	core	Class (V)
GLN3	nothing	core	Class (V)
MXR1	nothing	core	Class (V)
SAH1	nothing	core	Class (V)
ERG28	nothing	core	Class (V)
TPA1	nothing	core	Class (V)
RSM18	nothing	core	Class (V)
YER053C-A	nothing	core	Class (V)
HIS1	nothing	core	Class (V)
RPL34A	nothing	core	Class (V)
HMF1	nothing	core	Class (V)
HOR2	nothing	core	Class (V)
ICL1	nothing	core	Class (V)
YER066W	nothing	core	Class (V)
YER067W	nothing	core	Class (V)
ARG5%2C6	nothing	core	Class (V)
RPS24A	nothing	core	Class (V)
PTP3	nothing	core	Class (V)
YER076C	nothing	core	Class (V)
YER077C	nothing	core	Class (V)
ILV1	nothing	core	Class (V)
DOT6	nothing	core	Class (V)
YER088C-A, PTC2	nothing	core	Class (V)
TRP2	nothing	core	Class (V)
MET6	nothing	core	Class (V)
YER093C-A	nothing	core	Class (V)
RAD51	nothing	core	Class (V)
YER097W, UBP9	nothing	core	Class (V)
AST2	nothing	core	Class (V)
GLE2	nothing	core	Class (V)
FLO8	nothing	core	Class (V)
KAP123	nothing	core	Class (V)
SWI4	nothing	core	Class (V)
LSM4	nothing	core	Class (V)
TMN3	nothing	core	Class (V)
BOI2	nothing	core	Class (V)
SCS2	nothing	core	Class (V)
YCK3	nothing	core	Class (V)
DSE1	nothing	core	Class (V)
RPS26B	nothing	core	Class (V)
GLC7	nothing	core	Class (V)
MAG1	nothing	core	Class (V)
SPT15	nothing	core	Class (V)
PEA2	nothing	core	Class (V)
SPI1	nothing	core	Class (V)
UBP3	nothing	core	Class (V)
BEM2	nothing	core	Class (V)
COG3	nothing	core	Class (V)
YER158C	nothing	core	Class (V)
SPT2	nothing	core	Class (V)
YER163C	nothing	core	Class (V)
PAB1	nothing	core	Class (V)
BCK2	nothing	core	Class (V)

CCA1	nothing	core	Class (V)
RPH1	nothing	core	Class (V)
ADK2	nothing	core	Class (V)
ECM32	nothing	core	Class (V)
BMH1	nothing	core	Class (V)
PDA1	nothing	core	Class (V)
DMC1	nothing	core	Class (V)
ISC10, SLO1	nothing	core	Class (V)
YER185W	nothing	core	Class (V)
YER186C	nothing	core	Class (V)
AGP3	nothing	core	Class (V)
YFL054C	nothing	core	Class (V)
YFL052W	nothing	core	Class (V)
ALR2	nothing	core	Class (V)
FET5	nothing	core	Class (V)
ACT1	nothing	core	Class (V)
YPT1	nothing	core	Class (V)
TUB2	nothing	core	Class (V)
RPO41	nothing	core	Class (V)
MOB2	nothing	core	Class (V)
RPL22B	nothing	core	Class (V)
YFL034W	nothing	core	Class (V)
RIM15	nothing	core	Class (V)
CAF16	nothing	core	Class (V)
GYP8	nothing	core	Class (V)
BST1	nothing	core	Class (V)
LPD1	nothing	core	Class (V)
IES1	nothing	core	Class (V)
WWM1	nothing	core	Class (V)
CDC4	nothing	core	Class (V)
SMC1	nothing	core	Class (V)
VTC2	nothing	core	Class (V)
MSH4	nothing	core	Class (V)
SPB4	nothing	core	Class (V)
DEG1	nothing	core	Class (V)
YFR007W	nothing	core	Class (V)
FAR7	nothing	core	Class (V)
GCN20, YFR009W-A	nothing	core	Class (V)
YFR012W, YFR012W-A	nothing	core	Class (V)
YFR016C	nothing	core	Class (V)
FAB1	nothing	core	Class (V)
ROG3	nothing	core	Class (V)
PES4	nothing	core	Class (V)
LSB3	nothing	core	Class (V)
HIS2	nothing	core	Class (V)
MET10	nothing	core	Class (V)
PHO4	nothing	core	Class (V)
YFR039C	nothing	core	Class (V)
DUG1	nothing	core	Class (V)
BNA6	nothing	core	Class (V)
RET2	nothing	core	Class (V)
COS12	nothing	core	Class (V)
MNT2	nothing	core	Class (V)
ZRT1	nothing	core	Class (V)
FZF1	nothing	core	Class (V)
HFM1	nothing	core	Class (V)
PDE1	nothing	core	Class (V)
RTF1	nothing	core	Class (V)

DOC1	nothing	core	Class (V)
CSE1	nothing	core	Class (V)
HAP2	nothing	core	Class (V)
MTO1	nothing	core	Class (V)
ADE5%2C7	nothing	core	Class (V)
SEC15	nothing	core	Class (V)
YGL230C	nothing	core	Class (V)
SAP4	nothing	core	Class (V)
COG1	nothing	core	Class (V)
EDC1	nothing	core	Class (V)
CLG1	nothing	core	Class (V)
SKI8	nothing	core	Class (V)
NCS6	nothing	core	Class (V)
SIP2	nothing	core	Class (V)
YIP4	nothing	core	Class (V)
MDS3	nothing	core	Class (V)
YGL196W	nothing	core	Class (V)
HOS2	nothing	core	Class (V)
CDC55	nothing	core	Class (V)
RPS26A, YGL188C-A	nothing	core	Class (V)
TPN1	nothing	core	Class (V)
ATG1	nothing	core	Class (V)
TOS3	nothing	core	Class (V)
MPT5, YGL177W	nothing	core	Class (V)
ROK1	nothing	core	Class (V)
YGL159W	nothing	core	Class (V)
YGL157W	nothing	core	Class (V)
ARO2	nothing	core	Class (V)
RPL9A	nothing	core	Class (V)
MRF1	nothing	core	Class (V)
FLC3	nothing	core	Class (V)
RPL1B	nothing	core	Class (V)
PCL10	nothing	core	Class (V)
SOH1	nothing	core	Class (V)
SCS3	nothing	core	Class (V)
MON1	nothing	core	Class (V)
RPS2	nothing	core	Class (V)
GPG1	nothing	core	Class (V)
PRP43	nothing	core	Class (V)
ABC1	nothing	core	Class (V)
SNF4	nothing	core	Class (V)
YGL114W	nothing	core	Class (V)
YGL108C	nothing	core	Class (V)
RMD9	nothing	core	Class (V)
RPL28	nothing	core	Class (V)
YGL101W	nothing	core	Class (V)
LSG1	nothing	core	Class (V)
TOS8	nothing	core	Class (V)
VPS45	nothing	core	Class (V)
PAN2	nothing	core	Class (V)
NUP145	nothing	core	Class (V)
MAD1	nothing	core	Class (V)
SCY1	nothing	core	Class (V)
HNM1	nothing	core	Class (V)
RPL7A	nothing	core	Class (V)
MNP1	nothing	core	Class (V)
NPY1	nothing	core	Class (V)
MRH4	nothing	core	Class (V)

PUS2	nothing	core	Class (V)
DUO1	nothing	core	Class (V)
RAD6	nothing	core	Class (V)
YGL057C	nothing	core	Class (V)
SDS23	nothing	core	Class (V)
OLE1	nothing	core	Class (V)
ERV14	nothing	core	Class (V)
RPT6	nothing	core	Class (V)
DST1	nothing	core	Class (V)
YGL036W	nothing	core	Class (V)
MIG1	nothing	core	Class (V)
AGA2	nothing	core	Class (V)
RPL24A	nothing	core	Class (V)
RPL30	nothing	core	Class (V)
CGR1	nothing	core	Class (V)
SCW11	nothing	core	Class (V)
CWH41	nothing	core	Class (V)
TRP5	nothing	core	Class (V)
PGD1	nothing	core	Class (V)
PIB2	nothing	core	Class (V)
STT3	nothing	core	Class (V)
KAP122	nothing	core	Class (V)
PUF4	nothing	core	Class (V)
RPN14	nothing	core	Class (V)
SWC4	nothing	core	Class (V)
CUL3	nothing	core	Class (V)
PEX31	nothing	core	Class (V)
TFG2	nothing	core	Class (V)
PRP18	nothing	core	Class (V)
SEC9	nothing	core	Class (V)
NMA2, YGR011W	nothing	core	Class (V)
YGR016W	nothing	core	Class (V)
VMA7	nothing	core	Class (V)
YGR021W	nothing	core	Class (V)
MTL1	nothing	core	Class (V)
MSP1	nothing	core	Class (V)
GSC2	nothing	core	Class (V)
TIM21	nothing	core	Class (V)
RPL26B	nothing	core	Class (V)
CAX4	nothing	core	Class (V)
ACB1	nothing	core	Class (V)
ORM1	nothing	core	Class (V)
KSS1	nothing	core	Class (V)
BUD9	nothing	core	Class (V)
UFD1	nothing	core	Class (V)
YGR054W	nothing	core	Class (V)
MUP1	nothing	core	Class (V)
RSC1	nothing	core	Class (V)
LST7	nothing	core	Class (V)
VHT1	nothing	core	Class (V)
YGR068C	nothing	core	Class (V)
MRPL25	nothing	core	Class (V)
PEX8	nothing	core	Class (V)
YGR079W	nothing	core	Class (V)
TOM20	nothing	core	Class (V)
GCD2	nothing	core	Class (V)
MRP13	nothing	core	Class (V)
RPL11B	nothing	core	Class (V)

PIL1	nothing	core	Class (V)
UTP22	nothing	core	Class (V)
DBF2	nothing	core	Class (V)
VAS1	nothing	core	Class (V)
TEL2	nothing	core	Class (V)
NOP7	nothing	core	Class (V)
CLB1	nothing	core	Class (V)
CLB6	nothing	core	Class (V)
COG2	nothing	core	Class (V)
PPT1	nothing	core	Class (V)
PEX4	nothing	core	Class (V)
PRE9	nothing	core	Class (V)
LSB1, YGR137W	nothing	core	Class (V)
TPO2	nothing	core	Class (V)
CBF2	nothing	core	Class (V)
SKN1	nothing	core	Class (V)
THI4	nothing	core	Class (V)
ENP2	nothing	core	Class (V)
YGR146C	nothing	core	Class (V)
NAT2	nothing	core	Class (V)
RPL24B	nothing	core	Class (V)
CYS4	nothing	core	Class (V)
NSR1	nothing	core	Class (V)
RTS3	nothing	core	Class (V)
YGR161W-C	nothing	core	Class (V)
MRPS35	nothing	core	Class (V)
KRE11	nothing	core	Class (V)
PUS6	nothing	core	Class (V)
YGR169C-A	nothing	core	Class (V)
YIP1	nothing	core	Class (V)
CBP4	nothing	core	Class (V)
YGR174W-A	nothing	core	Class (V)
ERG1	nothing	core	Class (V)
OKP1	nothing	core	Class (V)
QCR9	nothing	core	Class (V)
UBR1	nothing	core	Class (V)
TYS1	nothing	core	Class (V)
BUB1	nothing	core	Class (V)
CRH1	nothing	core	Class (V)
TDH3	nothing	core	Class (V)
SNG1	nothing	core	Class (V)
YPP1	nothing	core	Class (V)
PCT1	nothing	core	Class (V)
YGR203W	nothing	core	Class (V)
ADE3	nothing	core	Class (V)
YGR205W	nothing	core	Class (V)
TRX2	nothing	core	Class (V)
ZPR1	nothing	core	Class (V)
SLI1	nothing	core	Class (V)
RPS0A	nothing	core	Class (V)
CCH1	nothing	core	Class (V)
CRM1	nothing	core	Class (V)
MRPL9	nothing	core	Class (V)
TOS2	nothing	core	Class (V)
AMA1	nothing	core	Class (V)
KEL2	nothing	core	Class (V)
PEX21	nothing	core	Class (V)
PFK1	nothing	core	Class (V)

SDA1	nothing	core	Class (V)
BRF1	nothing	core	Class (V)
CPD1	nothing	core	Class (V)
MGA1	nothing	core	Class (V)
YGR250C	nothing	core	Class (V)
ENO1	nothing	core	Class (V)
COQ6	nothing	core	Class (V)
RAD2	nothing	core	Class (V)
APL6	nothing	core	Class (V)
SAY1	nothing	core	Class (V)
MES1	nothing	core	Class (V)
TAF1	nothing	core	Class (V)
PXR1	nothing	core	Class (V)
YGR283C	nothing	core	Class (V)
ZUO1	nothing	core	Class (V)
MAL11	nothing	core	Class (V)
YHL044W	nothing	core	Class (V)
RPL8A	nothing	core	Class (V)
ECM29	nothing	core	Class (V)
OCA5	nothing	core	Class (V)
RMD11	nothing	core	Class (V)
FMP12	nothing	core	Class (V)
OPI1	nothing	core	Class (V)
YHL018W, YHL017W	nothing	core	Class (V)
DUR3	nothing	core	Class (V)
YLF2	nothing	core	Class (V)
YAP3	nothing	core	Class (V)
YHL008C	nothing	core	Class (V)
STE20	nothing	core	Class (V)
HSE1	nothing	core	Class (V)
RPL14B	nothing	core	Class (V)
OSH7	nothing	core	Class (V)
QCR10	nothing	core	Class (V)
NEM1	nothing	core	Class (V)
GPA1	nothing	core	Class (V)
ERG11	nothing	core	Class (V)
YHR009C	nothing	core	Class (V)
MIP6	nothing	core	Class (V)
DED81	nothing	core	Class (V)
YHR020W	nothing	core	Class (V)
RPS27B	nothing	core	Class (V)
YHR022C-A	nothing	core	Class (V)
THR1	nothing	core	Class (V)
DAP2	nothing	core	Class (V)
SLT2	nothing	core	Class (V)
PIH1	nothing	core	Class (V)
YHR035W	nothing	core	Class (V)
BRL1	nothing	core	Class (V)
PUT2	nothing	core	Class (V)
DOG1	nothing	core	Class (V)
YHR048W	nothing	core	Class (V)
CIC1	nothing	core	Class (V)
RSC30	nothing	core	Class (V)
CPR2	nothing	core	Class (V)
FYV4	nothing	core	Class (V)
VMA22	nothing	core	Class (V)
GIC1	nothing	core	Class (V)
SSZ1	nothing	core	Class (V)

SSF1	nothing	core	Class (V)
PCL5	nothing	core	Class (V)
STE12	nothing	core	Class (V)
IPI1	nothing	core	Class (V)
NAM8	nothing	core	Class (V)
YHR097C	nothing	core	Class (V)
SFB3	nothing	core	Class (V)
KIC1	nothing	core	Class (V)
SBE22	nothing	core	Class (V)
GGA2	nothing	core	Class (V)
CTM1	nothing	core	Class (V)
YHR113W	nothing	core	Class (V)
SET1	nothing	core	Class (V)
YHR122W	nothing	core	Class (V)
EPT1	nothing	core	Class (V)
NDT80	nothing	core	Class (V)
YHR127W	nothing	core	Class (V)
IGO2	nothing	core	Class (V)
YCK1	nothing	core	Class (V)
CHS7	nothing	core	Class (V)
CRP1	nothing	core	Class (V)
MRPL6	nothing	core	Class (V)
RTT107	nothing	core	Class (V)
PRP8	nothing	core	Class (V)
THP2	nothing	core	Class (V)
NMD3	nothing	core	Class (V)
ATG7	nothing	core	Class (V)
SPC97	nothing	core	Class (V)
ENO2	nothing	core	Class (V)
FMO1	nothing	core	Class (V)
YHR177W	nothing	core	Class (V)
OYE2	nothing	core	Class (V)
SVP26	nothing	core	Class (V)
SSP1	nothing	core	Class (V)
KOG1	nothing	core	Class (V)
MDM31	nothing	core	Class (V)
SCH9	nothing	core	Class (V)
BAT1	nothing	core	Class (V)
CRG1	nothing	core	Class (V)
YHR210C	nothing	core	Class (V)
YIL166C	nothing	core	Class (V)
IMP2'	nothing	core	Class (V)
MLP2	nothing	core	Class (V)
PAN6	nothing	core	Class (V)
TPM2	nothing	core	Class (V)
TMA108	nothing	core	Class (V)
FKH1	nothing	core	Class (V)
ASG1	nothing	core	Class (V)
TAO3	nothing	core	Class (V)
STH1	nothing	core	Class (V)
KGD1	nothing	core	Class (V)
AYR1	nothing	core	Class (V)
SIM1	nothing	core	Class (V)
HIS5	nothing	core	Class (V)
NUP159	nothing	core	Class (V)
POR2	nothing	core	Class (V)
HOS4	nothing	core	Class (V)
COX5B	nothing	core	Class (V)

SHQ1	nothing	core	Class (V)
ICE2	nothing	core	Class (V)
YIL089W	nothing	core	Class (V)
AVT7	nothing	core	Class (V)
AIR1	nothing	core	Class (V)
THS1	nothing	core	Class (V)
YIL077C	nothing	core	Class (V)
SEC28	nothing	core	Class (V)
RPN2	nothing	core	Class (V)
PCI8	nothing	core	Class (V)
MAM33	nothing	core	Class (V)
RPS24B	nothing	core	Class (V)
ARC15	nothing	core	Class (V)
SNP1	nothing	core	Class (V)
PCL7	nothing	core	Class (V)
NEO1	nothing	core	Class (V)
YIL047C-A, SYG1	nothing	core	Class (V)
YIL046W-A	nothing	core	Class (V)
MET30	nothing	core	Class (V)
CBR1	nothing	core	Class (V)
GVP36	nothing	core	Class (V)
APQ12	nothing	core	Class (V)
TED1	nothing	core	Class (V)
NOT3	nothing	core	Class (V)
PRM2	nothing	core	Class (V)
CAP2	nothing	core	Class (V)
KRE27	nothing	core	Class (V)
IRR1	nothing	core	Class (V)
YKE4	nothing	core	Class (V)
TIM44	nothing	core	Class (V)
YIL014C-A	nothing	core	Class (V)
FAA3	nothing	core	Class (V)
URM1	nothing	core	Class (V)
YIL001W	nothing	core	Class (V)
DJP1	nothing	core	Class (V)
MUC1	nothing	core	Class (V)
DAL1	nothing	core	Class (V)
DAL7	nothing	core	Class (V)
IRC24	nothing	core	Class (V)
YIR042C	nothing	core	Class (V)
YJL217W	nothing	core	Class (V)
HXT8	nothing	core	Class (V)
PEX2	nothing	core	Class (V)
LAA1	nothing	core	Class (V)
YJL206C	nothing	core	Class (V)
NCE101	nothing	core	Class (V)
RCY1	nothing	core	Class (V)
CDC6	nothing	core	Class (V)
RPL39	nothing	core	Class (V)
SWE1	nothing	core	Class (V)
MNN11	nothing	core	Class (V)
RPL17B	nothing	core	Class (V)
SWI3	nothing	core	Class (V)
CPS1	nothing	core	Class (V)
SET2	nothing	core	Class (V)
ERG20	nothing	core	Class (V)
JJJ2	nothing	core	Class (V)
SSY5	nothing	core	Class (V)

VPS35	nothing	core	Class (V)
SNA3	nothing	core	Class (V)
SFH5	nothing	core	Class (V)
YJL144W	nothing	core	Class (V)
TIF2	nothing	core	Class (V)
RPS21B	nothing	core	Class (V)
URA2	nothing	core	Class (V)
YJL123C	nothing	core	Class (V)
ASF1	nothing	core	Class (V)
CCT7	nothing	core	Class (V)
GZF3	nothing	core	Class (V)
UTP10	nothing	core	Class (V)
SET4	nothing	core	Class (V)
MEF2	nothing	core	Class (V)
GSH1	nothing	core	Class (V)
CHS6	nothing	core	Class (V)
SAP185	nothing	core	Class (V)
MRPL49	nothing	core	Class (V)
TOK1	nothing	core	Class (V)
DPB11	nothing	core	Class (V)
SCP160	nothing	core	Class (V)
UTP18	nothing	core	Class (V)
YJL068C	nothing	core	Class (V)
MPM1	nothing	core	Class (V)
LAS21	nothing	core	Class (V)
NUP82	nothing	core	Class (V)
BNA3	nothing	core	Class (V)
IKS1	nothing	core	Class (V)
ZAP1	nothing	core	Class (V)
YJL055W	nothing	core	Class (V)
TIM54	nothing	core	Class (V)
PEP8	nothing	core	Class (V)
MTR4	nothing	core	Class (V)
YJL046W	nothing	core	Class (V)
YJL043W	nothing	core	Class (V)
YJL038C	nothing	core	Class (V)
IRC18	nothing	core	Class (V)
KAR2	nothing	core	Class (V)
BBC1	nothing	core	Class (V)
VTC4	nothing	core	Class (V)
NOP9	nothing	core	Class (V)
CCT8	nothing	core	Class (V)
CYR1	nothing	core	Class (V)
SYS1	nothing	core	Class (V)
SAG1	nothing	core	Class (V)
YJR005C-A	nothing	core	Class (V)
SUI2	nothing	core	Class (V)
MET3	nothing	core	Class (V)
SPC1	nothing	core	Class (V)
YJR012C	nothing	core	Class (V)
TMA22	nothing	core	Class (V)
REC107	nothing	core	Class (V)
YJR039W	nothing	core	Class (V)
SSC1	nothing	core	Class (V)
TAH11	nothing	core	Class (V)
ANB1	nothing	core	Class (V)
OSM1	nothing	core	Class (V)
RAD7	nothing	core	Class (V)

YJR054W	nothing	core	Class (V)
HIT1	nothing	core	Class (V)
YJR056C	nothing	core	Class (V)
CBF1	nothing	core	Class (V)
LIA1	nothing	core	Class (V)
MIR1	nothing	core	Class (V)
ACF4	nothing	core	Class (V)
YJR088C	nothing	core	Class (V)
JSN1	nothing	core	Class (V)
RPL43B	nothing	core	Class (V)
SFC1	nothing	core	Class (V)
ADO1	nothing	core	Class (V)
NNF1	nothing	core	Class (V)
YJR115W, YJR116W	nothing	core	Class (V)
YJR120W, ATP2	nothing	core	Class (V)
VPS70	nothing	core	Class (V)
STR2	nothing	core	Class (V)
NMD5	nothing	core	Class (V)
ECM17	nothing	core	Class (V)
HIR3	nothing	core	Class (V)
YJR141W	nothing	core	Class (V)
MGM101	nothing	core	Class (V)
RPS4A	nothing	core	Class (V)
YJR146W, HMS2	nothing	core	Class (V)
DAN4	nothing	core	Class (V)
DAL5	nothing	core	Class (V)
MCH2	nothing	core	Class (V)
FRE2	nothing	core	Class (V)
COS9	nothing	core	Class (V)
SRY1	nothing	core	Class (V)
YRA2	nothing	core	Class (V)
DOA1	nothing	core	Class (V)
CBT1	nothing	core	Class (V)
LOS1	nothing	core	Class (V)
PTK1	nothing	core	Class (V)
YKT6	nothing	core	Class (V)
SDS22	nothing	core	Class (V)
CNB1	nothing	core	Class (V)
YKL187C	nothing	core	Class (V)
MTR2	nothing	core	Class (V)
SPE1	nothing	core	Class (V)
PRS1	nothing	core	Class (V)
STE3	nothing	core	Class (V)
LST4	nothing	core	Class (V)
EBP2	nothing	core	Class (V)
MRP49	nothing	core	Class (V)
MCD4	nothing	core	Class (V)
PIR1	nothing	core	Class (V)
YKL162C	nothing	core	Class (V)
YKL161C	nothing	core	Class (V)
SRP102	nothing	core	Class (V)
GPM1	nothing	core	Class (V)
DBR1	nothing	core	Class (V)
SDH1	nothing	core	Class (V)
MRP8	nothing	core	Class (V)
SDH3	nothing	core	Class (V)
TGL1	nothing	core	Class (V)
MRPL31	nothing	core	Class (V)

RMA1	nothing	core	Class (V)
SHE2	nothing	core	Class (V)
MYO3	nothing	core	Class (V)
PMU1	nothing	core	Class (V)
PGM1	nothing	core	Class (V)
YKL121W	nothing	core	Class (V)
HAP4	nothing	core	Class (V)
SLD2	nothing	core	Class (V)
GFA1	nothing	core	Class (V)
YKL098W	nothing	core	Class (V)
CWP2	nothing	core	Class (V)
CWP1	nothing	core	Class (V)
YJU2	nothing	core	Class (V)
YJU3	nothing	core	Class (V)
YKL091C	nothing	core	Class (V)
CUE2	nothing	core	Class (V)
SRX1	nothing	core	Class (V)
MDH1	nothing	core	Class (V)
VMA5	nothing	core	Class (V)
MUD2	nothing	core	Class (V)
LHS1	nothing	core	Class (V)
STB6	nothing	core	Class (V)
YKL068W-A	nothing	core	Class (V)
NUP100	nothing	core	Class (V)
MNR2	nothing	core	Class (V)
YKL063C	nothing	core	Class (V)
TMA19	nothing	core	Class (V)
DEF1	nothing	core	Class (V)
ASK1	nothing	core	Class (V)
CSE4	nothing	core	Class (V)
ELM1	nothing	core	Class (V)
PRI2	nothing	core	Class (V)
SPC42	nothing	core	Class (V)
UGP1	nothing	core	Class (V)
YKL033W-A	nothing	core	Class (V)
YKL033W	nothing	core	Class (V)
MAE1	nothing	core	Class (V)
PAN3	nothing	core	Class (V)
CDC16, MAK11	nothing	core	Class (V)
ATP7	nothing	core	Class (V)
ARC19	nothing	core	Class (V)
UFD4	nothing	core	Class (V)
RPL14A	nothing	core	Class (V)
BYE1	nothing	core	Class (V)
AUR1	nothing	core	Class (V)
MRP17	nothing	core	Class (V)
VPS1	nothing	core	Class (V)
PAP1	nothing	core	Class (V)
YKR005C	nothing	core	Class (V)
MRPL13	nothing	core	Class (V)
TOF2	nothing	core	Class (V)
YKR015C	nothing	core	Class (V)
VPS51	nothing	core	Class (V)
SAP190	nothing	core	Class (V)
SET3	nothing	core	Class (V)
GAP1	nothing	core	Class (V)
YKR041W	nothing	core	Class (V)
YSR3	nothing	core	Class (V)

RHO4, TRM2	nothing	core	Class (V)
TRM2	nothing	core	Class (V)
TIF1	nothing	core	Class (V)
UTP30	nothing	core	Class (V)
MET1	nothing	core	Class (V)
MRPL20	nothing	core	Class (V)
TVP38	nothing	core	Class (V)
TGL4	nothing	core	Class (V)
PXL1	nothing	core	Class (V)
PTR2	nothing	core	Class (V)
RPL40B	nothing	core	Class (V)
YKR096W	nothing	core	Class (V)
SIR1	nothing	core	Class (V)
FLO10	nothing	core	Class (V)
NFT1	nothing	core	Class (V)
MHT1	nothing	core	Class (V)
YLL058W	nothing	core	Class (V)
JLP1	nothing	core	Class (V)
YLL054C	nothing	core	Class (V)
FRE6	nothing	core	Class (V)
COF1	nothing	core	Class (V)
RPL8B	nothing	core	Class (V)
UBI4	nothing	core	Class (V)
ENT4	nothing	core	Class (V)
YLL029W	nothing	core	Class (V)
TPO1	nothing	core	Class (V)
HSP104	nothing	core	Class (V)
PAU17	nothing	core	Class (V)
SSA2	nothing	core	Class (V)
BPT1	nothing	core	Class (V)
YEH1	nothing	core	Class (V)
COX17	nothing	core	Class (V)
MMM1	nothing	core	Class (V)
SFI1	nothing	core	Class (V)
RTT109	nothing	core	Class (V)
DNM1	nothing	core	Class (V)
NOC3	nothing	core	Class (V)
YLR003C	nothing	core	Class (V)
THI73	nothing	core	Class (V)
SSK1	nothing	core	Class (V)
UBR2	nothing	core	Class (V)
RPL15A	nothing	core	Class (V)
YLR036C	nothing	core	Class (V)
YLR042C	nothing	core	Class (V)
PDC1	nothing	core	Class (V)
RPS0B	nothing	core	Class (V)
IES3	nothing	core	Class (V)
SPT8	nothing	core	Class (V)
ERG3	nothing	core	Class (V)
REX2	nothing	core	Class (V)
FRS1	nothing	core	Class (V)
RPL22A	nothing	core	Class (V)
RGR1	nothing	core	Class (V)
SIC1	nothing	core	Class (V)
EMP70	nothing	core	Class (V)
RAX2	nothing	core	Class (V)
ARP6	nothing	core	Class (V)
GAA1	nothing	core	Class (V)

IOC2	nothing	core	Class (V)
CHA4	nothing	core	Class (V)
CDC45	nothing	core	Class (V)
MDN1	nothing	core	Class (V)
REX3	nothing	core	Class (V)
AHP1	nothing	core	Class (V)
CCW12	nothing	core	Class (V)
HOG1	nothing	core	Class (V)
AVL9	nothing	core	Class (V)
CFT2	nothing	core	Class (V)
APC2	nothing	core	Class (V)
CKI1	nothing	core	Class (V)
PDC5	nothing	core	Class (V)
YLR137W	nothing	core	Class (V)
NHA1	nothing	core	Class (V)
YLR152C	nothing	core	Class (V)
ACS2	nothing	core	Class (V)
MAS1	nothing	core	Class (V)
SEC10	nothing	core	Class (V)
APS1	nothing	core	Class (V)
DPH5	nothing	core	Class (V)
YLR173W	nothing	core	Class (V)
YLR179C	nothing	core	Class (V)
RPL37A	nothing	core	Class (V)
EMG1	nothing	core	Class (V)
SKG3	nothing	core	Class (V)
MDL1	nothing	core	Class (V)
ATG26	nothing	core	Class (V)
MMR1	nothing	core	Class (V)
UPS1	nothing	core	Class (V)
PWP1	nothing	core	Class (V)
TUB4	nothing	core	Class (V)
CRR1	nothing	core	Class (V)
CPR6	nothing	core	Class (V)
YLR218C	nothing	core	Class (V)
CCC1	nothing	core	Class (V)
RSA3	nothing	core	Class (V)
UTP13	nothing	core	Class (V)
CDC42	nothing	core	Class (V)
TOP3	nothing	core	Class (V)
THI7	nothing	core	Class (V)
VPS34	nothing	core	Class (V)
YEF3	nothing	core	Class (V)
SSP120	nothing	core	Class (V)
YLR257W	nothing	core	Class (V)
SEC22	nothing	core	Class (V)
DCS1	nothing	core	Class (V)
PIG1	nothing	core	Class (V)
YLR287C	nothing	core	Class (V)
RPS30A	nothing	core	Class (V)
MEC3	nothing	core	Class (V)
GCD7	nothing	core	Class (V)
SEC72	nothing	core	Class (V)
GSP1	nothing	core	Class (V)
YLR294C, ATP14	nothing	core	Class (V)
ECM38	nothing	core	Class (V)
EXG1	nothing	core	Class (V)
MET17	nothing	core	Class (V)

ACO1	nothing	core	Class (V)
UBC12	nothing	core	Class (V)
NMA1	nothing	core	Class (V)
RPS25B	nothing	core	Class (V)
VRP1	nothing	core	Class (V)
RPP0	nothing	core	Class (V)
RPL26A	nothing	core	Class (V)
KAP95	nothing	core	Class (V)
RSC2	nothing	core	Class (V)
ADE13	nothing	core	Class (V)
YLR363W-A	nothing	core	Class (V)
RPS22B, SNR44	nothing	core	Class (V)
FBP1	nothing	core	Class (V)
SEC61	nothing	core	Class (V)
CSR1	nothing	core	Class (V)
RPS29A	nothing	core	Class (V)
ECM19	nothing	core	Class (V)
SKI2	nothing	core	Class (V)
BDF1	nothing	core	Class (V)
DUS3	nothing	core	Class (V)
YLR404W	nothing	core	Class (V)
UTP21	nothing	core	Class (V)
VIP1	nothing	core	Class (V)
YLR412W	nothing	core	Class (V)
YLR419W	nothing	core	Class (V)
SPP382	nothing	core	Class (V)
TUS1	nothing	core	Class (V)
RPS1A	nothing	core	Class (V)
SIR3	nothing	core	Class (V)
ECM7	nothing	core	Class (V)
RPL6B	nothing	core	Class (V)
FMP27	nothing	core	Class (V)
YLR455W	nothing	core	Class (V)
COX14	nothing	core	Class (V)
RSC9	nothing	core	Class (V)
ERG13	nothing	core	Class (V)
PGA3	nothing	core	Class (V)
TUB3	nothing	core	Class (V)
YML119W	nothing	core	Class (V)
NGL3	nothing	core	Class (V)
NAB6, YML116W-A	nothing	core	Class (V)
ATR1	nothing	core	Class (V)
VAN1	nothing	core	Class (V)
DAT1	nothing	core	Class (V)
URA5	nothing	core	Class (V)
SEC65	nothing	core	Class (V)
YML100W-A	nothing	core	Class (V)
TSL1	nothing	core	Class (V)
TAF13	nothing	core	Class (V)
VPS9	nothing	core	Class (V)
UTP14	nothing	core	Class (V)
PRE8	nothing	core	Class (V)
RPM2	nothing	core	Class (V)
TUB1	nothing	core	Class (V)
BET5	nothing	core	Class (V)
WAR1	nothing	core	Class (V)
HMG1	nothing	core	Class (V)
RPL6A	nothing	core	Class (V)

TCB3	nothing	core	Class (V)
POB3	nothing	core	Class (V)
SMA2	nothing	core	Class (V)
RPS1B	nothing	core	Class (V)
MFT1	nothing	core	Class (V)
SML1	nothing	core	Class (V)
CMP2	nothing	core	Class (V)
IMD4, SNR54	nothing	core	Class (V)
YML053C	nothing	core	Class (V)
YML050W	nothing	core	Class (V)
RSE1	nothing	core	Class (V)
GSF2	nothing	core	Class (V)
PRP39	nothing	core	Class (V)
RRN11	nothing	core	Class (V)
YMD8	nothing	core	Class (V)
NDC1	nothing	core	Class (V)
YML030W	nothing	core	Class (V)
TSA1	nothing	core	Class (V)
YOX1	nothing	core	Class (V)
RPS18B	nothing	core	Class (V)
YML6	nothing	core	Class (V)
RPS17A	nothing	core	Class (V)
NSE5	nothing	core	Class (V)
PSP2	nothing	core	Class (V)
PPZ1	nothing	core	Class (V)
TRM9	nothing	core	Class (V)
UBX2	nothing	core	Class (V)
YAP1	nothing	core	Class (V)
YPT7	nothing	core	Class (V)
MIC17	nothing	core	Class (V)
YMR003W	nothing	core	Class (V)
PLB2	nothing	core	Class (V)
ADI1	nothing	core	Class (V)
YMR010W	nothing	core	Class (V)
ERG5	nothing	core	Class (V)
SOK2	nothing	core	Class (V)
UBC7	nothing	core	Class (V)
MSS1	nothing	core	Class (V)
MRPL3	nothing	core	Class (V)
RSF1	nothing	core	Class (V)
MSN2	nothing	core	Class (V)
CCS1	nothing	core	Class (V)
YET2	nothing	core	Class (V)
ARA2	nothing	core	Class (V)
ARG80	nothing	core	Class (V)
MCM1	nothing	core	Class (V)
IOC4	nothing	core	Class (V)
NUP116	nothing	core	Class (V)
CSM3	nothing	core	Class (V)
ERB1	nothing	core	Class (V)
AAC1	nothing	core	Class (V)
RNA14	nothing	core	Class (V)
ECM40	nothing	core	Class (V)
AVO2	nothing	core	Class (V)
MOT3	nothing	core	Class (V)
TVP18	nothing	core	Class (V)
CTF18	nothing	core	Class (V)
YMR087W	nothing	core	Class (V)

VBA1	nothing	core	Class (V)
NPL6	nothing	core	Class (V)
UTP15	nothing	core	Class (V)
MUB1	nothing	core	Class (V)
YMR102C	nothing	core	Class (V)
YKU80	nothing	core	Class (V)
SPC24	nothing	core	Class (V)
YMR118C	nothing	core	Class (V)
YMR124W	nothing	core	Class (V)
STO1	nothing	core	Class (V)
DLT1	nothing	core	Class (V)
SAS2	nothing	core	Class (V)
ECM16	nothing	core	Class (V)
POM152	nothing	core	Class (V)
YMR130W	nothing	core	Class (V)
RRB1	nothing	core	Class (V)
RIM11	nothing	core	Class (V)
SIP5	nothing	core	Class (V)
RPS16A	nothing	core	Class (V)
YMR144W	nothing	core	Class (V)
YMR147W	nothing	core	Class (V)
YMR148W	nothing	core	Class (V)
FMP39	nothing	core	Class (V)
MRPS8	nothing	core	Class (V)
YMR160W	nothing	core	Class (V)
DNF3	nothing	core	Class (V)
MSS11	nothing	core	Class (V)
PAH1	nothing	core	Class (V)
YMR171C	nothing	core	Class (V)
HOT1	nothing	core	Class (V)
MMT1	nothing	core	Class (V)
YMR185W	nothing	core	Class (V)
MRPS17	nothing	core	Class (V)
MRPL24	nothing	core	Class (V)
RPL36A	nothing	core	Class (V)
ICY1	nothing	core	Class (V)
CLN1	nothing	core	Class (V)
ROT1	nothing	core	Class (V)
PFK2	nothing	core	Class (V)
YMR210W	nothing	core	Class (V)
SCJ1	nothing	core	Class (V)
GAS3	nothing	core	Class (V)
SKY1	nothing	core	Class (V)
TMA29	nothing	core	Class (V)
MTF1	nothing	core	Class (V)
RRP5	nothing	core	Class (V)
RPS10B	nothing	core	Class (V)
PEP5	nothing	core	Class (V)
TAF9	nothing	core	Class (V)
DFG5	nothing	core	Class (V)
RNT1	nothing	core	Class (V)
CUS1	nothing	core	Class (V)
RPL20A	nothing	core	Class (V)
ZRC1	nothing	core	Class (V)
HOR7	nothing	core	Class (V)
YMR252C, YMR253C	nothing	core	Class (V)
PET111	nothing	core	Class (V)
TIF11	nothing	core	Class (V)

SAP30	nothing	core	Class (V)
RRN9	nothing	core	Class (V)
URA10	nothing	core	Class (V)
SCS7	nothing	core	Class (V)
ZDS1	nothing	core	Class (V)
RCE1	nothing	core	Class (V)
BUL1	nothing	core	Class (V)
FCP1	nothing	core	Class (V)
YMR278W	nothing	core	Class (V)
MRPL33	nothing	core	Class (V)
DSS1	nothing	core	Class (V)
HSH155	nothing	core	Class (V)
YMR291W	nothing	core	Class (V)
YMR293C	nothing	core	Class (V)
LCB1	nothing	core	Class (V)
PRC1	nothing	core	Class (V)
ADE4	nothing	core	Class (V)
UBP15	nothing	core	Class (V)
YMR304C-A, SCW10	nothing	core	Class (V)
NIP1	nothing	core	Class (V)
YMR310C	nothing	core	Class (V)
PRE5	nothing	core	Class (V)
YMR315W	nothing	core	Class (V)
DIA1	nothing	core	Class (V)
FET4	nothing	core	Class (V)
RPD3	nothing	core	Class (V)
PEX6, MDJ2	nothing	core	Class (V)
ATP11	nothing	core	Class (V)
MCK1	nothing	core	Class (V)
YPT11, YNL303W	nothing	core	Class (V)
RPS19B	nothing	core	Class (V)
YNL295W	nothing	core	Class (V)
RIM21	nothing	core	Class (V)
PCL1	nothing	core	Class (V)
SEC21	nothing	core	Class (V)
MRPL10	nothing	core	Class (V)
CAF120	nothing	core	Class (V)
YNL277W-A	nothing	core	Class (V)
TOF1	nothing	core	Class (V)
SEC2	nothing	core	Class (V)
ALP1	nothing	core	Class (V)
ATX1	nothing	core	Class (V)
DSL1	nothing	core	Class (V)
SIP3	nothing	core	Class (V)
RPA49	nothing	core	Class (V)
SUI1	nothing	core	Class (V)
SLA2	nothing	core	Class (V)
ATG2	nothing	core	Class (V)
ZWF1	nothing	core	Class (V)
KEX2	nothing	core	Class (V)
YNL234W	nothing	core	Class (V)
BNI4	nothing	core	Class (V)
ELA1	nothing	core	Class (V)
URE2	nothing	core	Class (V)
CNM67	nothing	core	Class (V)
SSU72	nothing	core	Class (V)
POP1	nothing	core	Class (V)
ALG9	nothing	core	Class (V)

YNL217W	nothing	core	Class (V)
RAP1	nothing	core	Class (V)
PEX17	nothing	core	Class (V)
YNL213C	nothing	core	Class (V)
SSB2	nothing	core	Class (V)
YNL208W	nothing	core	Class (V)
YNL200C	nothing	core	Class (V)
WHI3	nothing	core	Class (V)
CHS1	nothing	core	Class (V)
YNL190W	nothing	core	Class (V)
KAR1	nothing	core	Class (V)
YNL187W	nothing	core	Class (V)
NPR1	nothing	core	Class (V)
MDG1	nothing	core	Class (V)
FMP41	nothing	core	Class (V)
SKO1	nothing	core	Class (V)
BNI5	nothing	core	Class (V)
YNL165W	nothing	core	Class (V)
YNL162W-A	nothing	core	Class (V)
RPL42A	nothing	core	Class (V)
YGP1	nothing	core	Class (V)
ASI2	nothing	core	Class (V)
PGA1	nothing	core	Class (V)
IGO1	nothing	core	Class (V)
NSG2	nothing	core	Class (V)
YNL152W	nothing	core	Class (V)
RPC31	nothing	core	Class (V)
ALF1	nothing	core	Class (V)
YNL146W	nothing	core	Class (V)
MFA2	nothing	core	Class (V)
AAH1	nothing	core	Class (V)
YNL140C, RLR1	nothing	core	Class (V)
YNL134C	nothing	core	Class (V)
KRE33	nothing	core	Class (V)
NRK1	nothing	core	Class (V)
NAF1	nothing	core	Class (V)
NMA111	nothing	core	Class (V)
YNL122C	nothing	core	Class (V)
TOM70	nothing	core	Class (V)
RPC19	nothing	core	Class (V)
DBP2	nothing	core	Class (V)
YNL108C	nothing	core	Class (V)
YAF9	nothing	core	Class (V)
INP52	nothing	core	Class (V)
RPS7B	nothing	core	Class (V)
YNL095C	nothing	core	Class (V)
APP1	nothing	core	Class (V)
NST1	nothing	core	Class (V)
TOP2	nothing	core	Class (V)
TCB2	nothing	core	Class (V)
YNL086W	nothing	core	Class (V)
SAL1	nothing	core	Class (V)
TPM1	nothing	core	Class (V)
MSK1	nothing	core	Class (V)
RNH201	nothing	core	Class (V)
LAT1	nothing	core	Class (V)
TOM7	nothing	core	Class (V)
RPL16B	nothing	core	Class (V)

FKH2	nothing	core	Class (V)
RPL9B	nothing	core	Class (V)
SUN4	nothing	core	Class (V)
AQR1	nothing	core	Class (V)
YDJ1	nothing	core	Class (V)
COX5A	nothing	core	Class (V)
COG5	nothing	core	Class (V)
YNL050C	nothing	core	Class (V)
YNL046W	nothing	core	Class (V)
BDP1	nothing	core	Class (V)
CRZ1	nothing	core	Class (V)
SAM50	nothing	core	Class (V)
SSN8	nothing	core	Class (V)
FAP1	nothing	core	Class (V)
HDA1	nothing	core	Class (V)
ARK1	nothing	core	Class (V)
PUB1	nothing	core	Class (V)
PBI2	nothing	core	Class (V)
HEF3	nothing	core	Class (V)
YNL010W	nothing	core	Class (V)
SIS1	nothing	core	Class (V)
RLP7	nothing	core	Class (V)
DOM34	nothing	core	Class (V)
CIT1	nothing	core	Class (V)
RPC34	nothing	core	Class (V)
YNR004W	nothing	core	Class (V)
LRO1	nothing	core	Class (V)
YNR014W	nothing	core	Class (V)
SMM1	nothing	core	Class (V)
ARE2	nothing	core	Class (V)
ATP23	nothing	core	Class (V)
SNF12	nothing	core	Class (V)
BUD17	nothing	core	Class (V)
ALG12	nothing	core	Class (V)
SSK2	nothing	core	Class (V)
SOL1	nothing	core	Class (V)
YNR036C	nothing	core	Class (V)
DBP6	nothing	core	Class (V)
ZRG17	nothing	core	Class (V)
YNR040W	nothing	core	Class (V)
MVD1	nothing	core	Class (V)
TRM112	nothing	core	Class (V)
YNR047W	nothing	core	Class (V)
POP2	nothing	core	Class (V)
NOG2, SNR191	nothing	core	Class (V)
MNT4	nothing	core	Class (V)
YNR061C	nothing	core	Class (V)
YNR062C	nothing	core	Class (V)
YNR065C, YNR066C	nothing	core	Class (V)
DSE4	nothing	core	Class (V)
YNR070W	nothing	core	Class (V)
BDS1	nothing	core	Class (V)
ENB1	nothing	core	Class (V)
ZPS1	nothing	core	Class (V)
FRE7	nothing	core	Class (V)
DCP1	nothing	core	Class (V)
SPT20	nothing	core	Class (V)
PEX11	nothing	core	Class (V)

PSF3	nothing	core	Class (V)
CTR9	nothing	core	Class (V)
NOP8	nothing	core	Class (V)
RIB4	nothing	core	Class (V)
RRP40	nothing	core	Class (V)
PPM2	nothing	core	Class (V)
ARG8	nothing	core	Class (V)
CDC33	nothing	core	Class (V)
HRT1	nothing	core	Class (V)
ALR1	nothing	core	Class (V)
VPS68	nothing	core	Class (V)
RPL25	nothing	core	Class (V)
TRM13	nothing	core	Class (V)
HRP1	nothing	core	Class (V)
SMF1	nothing	core	Class (V)
RPL18A	nothing	core	Class (V)
MCH4	nothing	core	Class (V)
RRI2	nothing	core	Class (V)
MSN1	nothing	core	Class (V)
PAP2	nothing	core	Class (V)
SHR5	nothing	core	Class (V)
ZEO1	nothing	core	Class (V)
INO4	nothing	core	Class (V)
YOL107W	nothing	core	Class (V)
ITR2	nothing	core	Class (V)
TPT1	nothing	core	Class (V)
RFC4	nothing	core	Class (V)
YOL092W	nothing	core	Class (V)
YOL086W-A	nothing	core	Class (V)
ADH1	nothing	core	Class (V)
ATG19	nothing	core	Class (V)
YOL073C	nothing	core	Class (V)
INP54	nothing	core	Class (V)
ARG1	nothing	core	Class (V)
YOL057W	nothing	core	Class (V)
GPM3	nothing	core	Class (V)
THI20	nothing	core	Class (V)
PSH1	nothing	core	Class (V)
SPE2	nothing	core	Class (V)
GAL11	nothing	core	Class (V)
PSK2	nothing	core	Class (V)
PEX15	nothing	core	Class (V)
RPS15	nothing	core	Class (V)
RPP2A	nothing	core	Class (V)
YOL038C-A	nothing	core	Class (V)
PRE6	nothing	core	Class (V)
MDM38	nothing	core	Class (V)
MIM1	nothing	core	Class (V)
LAG2	nothing	core	Class (V)
IFM1	nothing	core	Class (V)
YOL022C	nothing	core	Class (V)
TAT2	nothing	core	Class (V)
YOL019W-A	nothing	core	Class (V)
IRC10	nothing	core	Class (V)
HTZ1	nothing	core	Class (V)
PLB3	nothing	core	Class (V)
TOP1	nothing	core	Class (V)
SIN3	nothing	core	Class (V)

PFA4	nothing	core	Class (V)
RRP6	nothing	core	Class (V)
YSP3	nothing	core	Class (V)
YOR006C	nothing	core	Class (V)
SLG1	nothing	core	Class (V)
TIR2	nothing	core	Class (V)
YOR011W-A	nothing	core	Class (V)
PET127	nothing	core	Class (V)
ROD1	nothing	core	Class (V)
HSP10	nothing	core	Class (V)
YOR020W-A	nothing	core	Class (V)
YOR021C	nothing	core	Class (V)
YOR022C	nothing	core	Class (V)
AHC1	nothing	core	Class (V)
CIN5	nothing	core	Class (V)
DFG16, CRS5	nothing	core	Class (V)
AKR2	nothing	core	Class (V)
PEP12	nothing	core	Class (V)
CYC2	nothing	core	Class (V)
HIR2	nothing	core	Class (V)
CKB2	nothing	core	Class (V)
TOM6	nothing	core	Class (V)
DBP5	nothing	core	Class (V)
STD1	nothing	core	Class (V)
RSB1	nothing	core	Class (V)
YOR051C	nothing	core	Class (V)
ASE1	nothing	core	Class (V)
YOR059C, YOR060C	nothing	core	Class (V)
CKA2	nothing	core	Class (V)
YOR062C	nothing	core	Class (V)
RPL3	nothing	core	Class (V)
ALG8	nothing	core	Class (V)
SKI7	nothing	core	Class (V)
RTS2	nothing	core	Class (V)
YOR084W	nothing	core	Class (V)
OST3	nothing	core	Class (V)
TCB1	nothing	core	Class (V)
YVC1	nothing	core	Class (V)
ECM3	nothing	core	Class (V)
YOR093C	nothing	core	Class (V)
RPS7A	nothing	core	Class (V)
YOR097C	nothing	core	Class (V)
NUP1	nothing	core	Class (V)
PIN2, YOR105W	nothing	core	Class (V)
LEU9	nothing	core	Class (V)
TFC7	nothing	core	Class (V)
CEX1	nothing	core	Class (V)
UBP2	nothing	core	Class (V)
CAT5	nothing	core	Class (V)
IAH1	nothing	core	Class (V)
RGA1	nothing	core	Class (V)
ADE2	nothing	core	Class (V)
YOR129C	nothing	core	Class (V)
YOR131C	nothing	core	Class (V)
EFT1	nothing	core	Class (V)
SFL1	nothing	core	Class (V)
THI80	nothing	core	Class (V)
MRPL23	nothing	core	Class (V)

RPB2	nothing	core	Class (V)
NFI1	nothing	core	Class (V)
MED4	nothing	core	Class (V)
SYC1	nothing	core	Class (V)
RPS30B	nothing	core	Class (V)
TUF1	nothing	core	Class (V)
MSB1	nothing	core	Class (V)
IES4	nothing	core	Class (V)
MRM1	nothing	core	Class (V)
HIS3, YOR203W	nothing	core	Class (V)
YOR203W, DED1	nothing	core	Class (V)
FMP38	nothing	core	Class (V)
YOR215C	nothing	core	Class (V)
MCT1	nothing	core	Class (V)
YOR227W	nothing	core	Class (V)
YOR228C	nothing	core	Class (V)
WTM2	nothing	core	Class (V)
WTM1	nothing	core	Class (V)
MKK1	nothing	core	Class (V)
MGE1	nothing	core	Class (V)
KIN4	nothing	core	Class (V)
RPL33B	nothing	core	Class (V)
HES1	nothing	core	Class (V)
PUS7	nothing	core	Class (V)
YOR251C	nothing	core	Class (V)
TMA16	nothing	core	Class (V)
TRE2	nothing	core	Class (V)
GCD1	nothing	core	Class (V)
DSE3	nothing	core	Class (V)
RBL2	nothing	core	Class (V)
PNT1	nothing	core	Class (V)
HRK1	nothing	core	Class (V)
FSF1	nothing	core	Class (V)
PLP2	nothing	core	Class (V)
YOR283W	nothing	core	Class (V)
YOR285W	nothing	core	Class (V)
MPD1	nothing	core	Class (V)
YOR289W	nothing	core	Class (V)
SNF2	nothing	core	Class (V)
RPS10A	nothing	core	Class (V)
UAF30	nothing	core	Class (V)
YOR296W	nothing	core	Class (V)
TIM18	nothing	core	Class (V)
BUD7, YOR300W	nothing	core	Class (V)
RAX1	nothing	core	Class (V)
YOR302W, CPA1	nothing	core	Class (V)
MCH5	nothing	core	Class (V)
YOR309C, NOP58	nothing	core	Class (V)
RPL20B	nothing	core	Class (V)
SPS4	nothing	core	Class (V)
YOR316C-A	nothing	core	Class (V)
GNT1	nothing	core	Class (V)
PMT3	nothing	core	Class (V)
LDB19	nothing	core	Class (V)
PRO2	nothing	core	Class (V)
FRT1	nothing	core	Class (V)
MIP1	nothing	core	Class (V)
VMA4	nothing	core	Class (V)

MRS2	nothing	core	Class (V)
ALA1	nothing	core	Class (V)
TEA1	nothing	core	Class (V)
UBC11	nothing	core	Class (V)
RPA43	nothing	core	Class (V)
RPA190	nothing	core	Class (V)
YOR342C	nothing	core	Class (V)
MSC6	nothing	core	Class (V)
GDS1	nothing	core	Class (V)
VTS1	nothing	core	Class (V)
PDE2	nothing	core	Class (V)
PRT1	nothing	core	Class (V)
PRE10	nothing	core	Class (V)
RAD17	nothing	core	Class (V)
RPS12	nothing	core	Class (V)
MRS6	nothing	core	Class (V)
NUD1	nothing	core	Class (V)
GDH1	nothing	core	Class (V)
RDR1	nothing	core	Class (V)
FRE3	nothing	core	Class (V)
FIT3	nothing	core	Class (V)
FRE5	nothing	core	Class (V)
SAM3	nothing	core	Class (V)
SAM4	nothing	core	Class (V)
ATP15	nothing	core	Class (V)
KAR9	nothing	core	Class (V)
DIM1	nothing	core	Class (V)
DIP5	nothing	core	Class (V)
YPL264C	nothing	core	Class (V)
FUM1	nothing	core	Class (V)
BBP1	nothing	core	Class (V)
ICY2	nothing	core	Class (V)
RPL36B	nothing	core	Class (V)
GAL4	nothing	core	Class (V)
YPL247C	nothing	core	Class (V)
IQG1	nothing	core	Class (V)
CIN2	nothing	core	Class (V)
SUI3	nothing	core	Class (V)
NSL1	nothing	core	Class (V)
FAS2	nothing	core	Class (V)
CET1	nothing	core	Class (V)
ALG5	nothing	core	Class (V)
NEW1	nothing	core	Class (V)
YPL225W	nothing	core	Class (V)
MMT2	nothing	core	Class (V)
FLC1	nothing	core	Class (V)
RPL1A	nothing	core	Class (V)
PCL8	nothing	core	Class (V)
SAR1	nothing	core	Class (V)
BMS1	nothing	core	Class (V)
YPL206C	nothing	core	Class (V)
TPK2	nothing	core	Class (V)
AFT2	nothing	core	Class (V)
APL5	nothing	core	Class (V)
DDC1	nothing	core	Class (V)
NAB3	nothing	core	Class (V)
POS5	nothing	core	Class (V)
MF(ALPHA)1	nothing	core	Class (V)

MRN1	nothing	core	Class (V)
TCO89	nothing	core	Class (V)
PPQ1	nothing	core	Class (V)
CBC2	nothing	core	Class (V)
CUP9	nothing	core	Class (V)
SPT14	nothing	core	Class (V)
NIP100	nothing	core	Class (V)
MRPL40	nothing	core	Class (V)
COX10	nothing	core	Class (V)
YPL168W	nothing	core	Class (V)
REV3	nothing	core	Class (V)
SVS1	nothing	core	Class (V)
YPL162C	nothing	core	Class (V)
BEM4	nothing	core	Class (V)
CDC60	nothing	core	Class (V)
KIP2	nothing	core	Class (V)
RAD53	nothing	core	Class (V)
NOP53	nothing	core	Class (V)
KES1	nothing	core	Class (V)
RPL33A	nothing	core	Class (V)
YPL141C	nothing	core	Class (V)
MKK2	nothing	core	Class (V)
UME1	nothing	core	Class (V)
ISU1	nothing	core	Class (V)
RDS2	nothing	core	Class (V)
COX11	nothing	core	Class (V)
TAF14	nothing	core	Class (V)
TBF1	nothing	core	Class (V)
NAN1	nothing	core	Class (V)
MRP51	nothing	core	Class (V)
IDI1	nothing	core	Class (V)
HOS3	nothing	core	Class (V)
BEM3	nothing	core	Class (V)
CAR1	nothing	core	Class (V)
YPL109C	nothing	core	Class (V)
SSE1	nothing	core	Class (V)
YPL105C	nothing	core	Class (V)
ELP4	nothing	core	Class (V)
ATG21	nothing	core	Class (V)
MGR2	nothing	core	Class (V)
PNG1	nothing	core	Class (V)
SEC62	nothing	core	Class (V)
NOG1	nothing	core	Class (V)
GLR1	nothing	core	Class (V)
RPS6A	nothing	core	Class (V)
YDC1	nothing	core	Class (V)
ELP3	nothing	core	Class (V)
MOT1	nothing	core	Class (V)
RPS9A	nothing	core	Class (V)
RPL21B	nothing	core	Class (V)
ATP4	nothing	core	Class (V)
GPI2	nothing	core	Class (V)
YTA6	nothing	core	Class (V)
YPL068C, YPL067C	nothing	core	Class (V)
TIM50	nothing	core	Class (V)
SUR1	nothing	core	Class (V)
ARL3	nothing	core	Class (V)
MNN9	nothing	core	Class (V)

CAM1	nothing	core	Class (V)
VPS16	nothing	core	Class (V)
NOP4	nothing	core	Class (V)
SSN3	nothing	core	Class (V)
PMA2	nothing	core	Class (V)
SVL3	nothing	core	Class (V)
TRM44	nothing	core	Class (V)
SUV3	nothing	core	Class (V)
ERG10	nothing	core	Class (V)
SKS1	nothing	core	Class (V)
MET12	nothing	core	Class (V)
RAD1	nothing	core	Class (V)
ULP1	nothing	core	Class (V)
IRC15	nothing	core	Class (V)
HST2	nothing	core	Class (V)
NCR1	nothing	core	Class (V)
HAT1	nothing	core	Class (V)
YPR003C	nothing	core	Class (V)
YPR004C	nothing	core	Class (V)
HAL1	nothing	core	Class (V)
ICL2	nothing	core	Class (V)
REC8	nothing	core	Class (V)
HAA1	nothing	core	Class (V)
YPR010C-A	nothing	core	Class (V)
YPR013C	nothing	core	Class (V)
TIF6	nothing	core	Class (V)
DSS4	nothing	core	Class (V)
CDC54	nothing	core	Class (V)
AGC1	nothing	core	Class (V)
EAF3	nothing	core	Class (V)
YME1	nothing	core	Class (V)
CCL1	nothing	core	Class (V)
NTO1	nothing	core	Class (V)
SRO7	nothing	core	Class (V)
HTS1	nothing	core	Class (V)
ARP7	nothing	core	Class (V)
VMA13	nothing	core	Class (V)
RPL43A	nothing	core	Class (V)
MNI2	nothing	core	Class (V)
MSF1	nothing	core	Class (V)
ATG11	nothing	core	Class (V)
SEC8	nothing	core	Class (V)
TFB4	nothing	core	Class (V)
SPE3	nothing	core	Class (V)
MED1	nothing	core	Class (V)
TKL1	nothing	core	Class (V)
OPY2	nothing	core	Class (V)
DIB1	nothing	core	Class (V)
SRP54	nothing	core	Class (V)
SYT1, YPR096C	nothing	core	Class (V)
MRPL51	nothing	core	Class (V)
RPN7	nothing	core	Class (V)
DBF20	nothing	core	Class (V)
MRD1	nothing	core	Class (V)
YPR114W	nothing	core	Class (V)
YPR115W	nothing	core	Class (V)
YPR116W, YPR117W	nothing	core	Class (V)
MRI1	nothing	core	Class (V)

CLB2	nothing	core	Class (V)
CLB5	nothing	core	Class (V)
CTR1	nothing	core	Class (V)
YLH47	nothing	core	Class (V)
TOM5	nothing	core	Class (V)
MSS18	nothing	core	Class (V)
RRP9	nothing	core	Class (V)
KAR3	nothing	core	Class (V)
RRP15	nothing	core	Class (V)
NOC4	nothing	core	Class (V)
ASN1	nothing	core	Class (V)
YPR148C	nothing	core	Class (V)
URN1	nothing	core	Class (V)
TPO3	nothing	core	Class (V)
YPR158W	nothing	core	Class (V)
SGV1	nothing	core	Class (V)
TIF3	nothing	core	Class (V)
BSP1	nothing	core	Class (V)
YPR174C	nothing	core	Class (V)
PRP4	nothing	core	Class (V)
HDA3	nothing	core	Class (V)
SEC23	nothing	core	Class (V)
DPM1	nothing	core	Class (V)
SKI3	nothing	core	Class (V)
RPC82	nothing	core	Class (V)
QCR2	nothing	core	Class (V)
ARR1	nothing	core	Class (V)
GPB2	nothing	other	Class (V)
EFB1, SNR18	nothing	other	Class (V)
YAR028W, YAR029W	nothing	other	Class (V)
YBL104C	nothing	other	Class (V)
BNA4	nothing	other	Class (V)
CDC27	nothing	other	Class (V)
YBL059W	nothing	other	Class (V)
PIN4	nothing	other	Class (V)
SEC17	nothing	other	Class (V)
RFT1	nothing	other	Class (V)
ACH1	nothing	other	Class (V)
HHT1	nothing	other	Class (V)
IPP1	nothing	other	Class (V)
FUR4	nothing	other	Class (V)
GIP1	nothing	other	Class (V)
ZTA1	nothing	other	Class (V)
RFS1	nothing	other	Class (V)
UBP14	nothing	other	Class (V)
AKL1	nothing	other	Class (V)
SPT7	nothing	other	Class (V)
GRS1, YBR121C-A	nothing	other	Class (V)
TFC1	nothing	other	Class (V)
TPS1	nothing	other	Class (V)
OPY1	nothing	other	Class (V)
ADH5	nothing	other	Class (V)
YSY6	nothing	other	Class (V)
SWD3	nothing	other	Class (V)
YPC1	nothing	other	Class (V)
HIS7	nothing	other	Class (V)
RIB5	nothing	other	Class (V)
FMP21	nothing	other	Class (V)

DUG2	nothing	other	Class (V)
MRPL27	nothing	other	Class (V)
YBR284W	nothing	other	Class (V)
YCL049C	nothing	other	Class (V)
YCL048W-A	nothing	other	Class (V)
YCL047C	nothing	other	Class (V)
YCL042W, GLK1	nothing	other	Class (V)
SRO9	nothing	other	Class (V)
RNQ1	nothing	other	Class (V)
YCL012C	nothing	other	Class (V)
GBP2	nothing	other	Class (V)
YCR015C	nothing	other	Class (V)
HTL1	nothing	other	Class (V)
SYP1	nothing	other	Class (V)
TAF2	nothing	other	Class (V)
TAH1	nothing	other	Class (V)
BUD31	nothing	other	Class (V)
ABP1	nothing	other	Class (V)
YCR099C, YCR100C, YCR101(	nothing	other	Class (V)
GYP7	nothing	other	Class (V)
TIM22	nothing	other	Class (V)
UGA4	nothing	other	Class (V)
YDL206W	nothing	other	Class (V)
HEM3	nothing	other	Class (V)
GGC1	nothing	other	Class (V)
SNF3	nothing	other	Class (V)
PPH22	nothing	other	Class (V)
CDC53	nothing	other	Class (V)
CDC48	nothing	other	Class (V)
HNT1	nothing	other	Class (V)
SNA4	nothing	other	Class (V)
MSS2	nothing	other	Class (V)
QRI1	nothing	other	Class (V)
YDL085C-A	nothing	other	Class (V)
VAM6	nothing	other	Class (V)
YDL057W	nothing	other	Class (V)
PBP4	nothing	other	Class (V)
MTF2	nothing	other	Class (V)
NAT1	nothing	other	Class (V)
YDL025C	nothing	other	Class (V)
OSH2	nothing	other	Class (V)
RPT2	nothing	other	Class (V)
MED2	nothing	other	Class (V)
SOK1	nothing	other	Class (V)
DAD1	nothing	other	Class (V)
VPS54	nothing	other	Class (V)
REG1	nothing	other	Class (V)
LYS14	nothing	other	Class (V)
HEM13	nothing	other	Class (V)
RPC11	nothing	other	Class (V)
CDC34	nothing	other	Class (V)
YDR056C	nothing	other	Class (V)
UBC5	nothing	other	Class (V)
STN1	nothing	other	Class (V)
AFR1	nothing	other	Class (V)
TMA64	nothing	other	Class (V)
DPB4	nothing	other	Class (V)
ARO1	nothing	other	Class (V)

YDR131C	nothing	other	Class (V)
YDR132C	nothing	other	Class (V)
RGP1	nothing	other	Class (V)
RUB1	nothing	other	Class (V)
PEX7	nothing	other	Class (V)
SAN1	nothing	other	Class (V)
SWI5	nothing	other	Class (V)
CTH1	nothing	other	Class (V)
ENT5	nothing	other	Class (V)
NGG1	nothing	other	Class (V)
PLP1	nothing	other	Class (V)
VPS64	nothing	other	Class (V)
GCD6	nothing	other	Class (V)
PCF11	nothing	other	Class (V)
PEX5	nothing	other	Class (V)
CTA1	nothing	other	Class (V)
SET7	nothing	other	Class (V)
AKR1	nothing	other	Class (V)
YDR282C	nothing	other	Class (V)
GCN2	nothing	other	Class (V)
SSD1	nothing	other	Class (V)
DPL1	nothing	other	Class (V)
HDA2	nothing	other	Class (V)
SUR2	nothing	other	Class (V)
YDR306C	nothing	other	Class (V)
GIC2	nothing	other	Class (V)
RAD34	nothing	other	Class (V)
IPK1	nothing	other	Class (V)
UBX5	nothing	other	Class (V)
SWR1	nothing	other	Class (V)
MSN5	nothing	other	Class (V)
ATP22	nothing	other	Class (V)
SBE2	nothing	other	Class (V)
SAC7	nothing	other	Class (V)
UBA2	nothing	other	Class (V)
ADE8	nothing	other	Class (V)
DFM1	nothing	other	Class (V)
CYM1	nothing	other	Class (V)
PPZ2	nothing	other	Class (V)
ADA2	nothing	other	Class (V)
GUK1	nothing	other	Class (V)
MFA1	nothing	other	Class (V)
KRE2	nothing	other	Class (V)
PUF6	nothing	other	Class (V)
EMI2	nothing	other	Class (V)
HSP31	nothing	other	Class (V)
IRC4	nothing	other	Class (V)
MAK10	nothing	other	Class (V)
AFG1	nothing	other	Class (V)
YEL048C	nothing	other	Class (V)
EDC3	nothing	other	Class (V)
YEL007W	nothing	other	Class (V)
VAB2	nothing	other	Class (V)
YEA4	nothing	other	Class (V)
SEC3	nothing	other	Class (V)
HEM14	nothing	other	Class (V)
GAL83	nothing	other	Class (V)
YEN1	nothing	other	Class (V)

ACA1	nothing	other	Class (V)
SPO73	nothing	other	Class (V)
SAP1	nothing	other	Class (V)
CAJ1	nothing	other	Class (V)
ISD11	nothing	other	Class (V)
PIC2	nothing	other	Class (V)
CEM1	nothing	other	Class (V)
ALD5	nothing	other	Class (V)
YOS1	nothing	other	Class (V)
FMP29	nothing	other	Class (V)
SSA4	nothing	other	Class (V)
SPR6, SLX8	nothing	other	Class (V)
YER134C	nothing	other	Class (V)
COX15	nothing	other	Class (V)
BUR6	nothing	other	Class (V)
CHD1	nothing	other	Class (V)
DNF1	nothing	other	Class (V)
TMT1	nothing	other	Class (V)
YER187W	nothing	other	Class (V)
RGD2	nothing	other	Class (V)
OTU1	nothing	other	Class (V)
YFL042C	nothing	other	Class (V)
YFL041W-A	nothing	other	Class (V)
YFL032W, HAC1	nothing	other	Class (V)
STE2	nothing	other	Class (V)
EPL1	nothing	other	Class (V)
MDJ1	nothing	other	Class (V)
SEC4	nothing	other	Class (V)
YFR006W	nothing	other	Class (V)
GSY1	nothing	other	Class (V)
YFR018C	nothing	other	Class (V)
CDC14	nothing	other	Class (V)
PTR3	nothing	other	Class (V)
SMC2	nothing	other	Class (V)
QCR6	nothing	other	Class (V)
IRC5	nothing	other	Class (V)
RMD8	nothing	other	Class (V)
ADH4	nothing	other	Class (V)
GUS1	nothing	other	Class (V)
KAP114	nothing	other	Class (V)
SHE10	nothing	other	Class (V)
VAM7	nothing	other	Class (V)
MIG2	nothing	other	Class (V)
KEX1	nothing	other	Class (V)
COX13	nothing	other	Class (V)
STR3	nothing	other	Class (V)
GTS1	nothing	other	Class (V)
YGL160W	nothing	other	Class (V)
MET13	nothing	other	Class (V)
NAB2	nothing	other	Class (V)
YGL117W	nothing	other	Class (V)
USE1	nothing	other	Class (V)
NBP35	nothing	other	Class (V)
YGL082W	nothing	other	Class (V)
FMP37	nothing	other	Class (V)
PYC1	nothing	other	Class (V)
YGL059W	nothing	other	Class (V)
PNC1	nothing	other	Class (V)

ATE1	nothing	other	Class (V)
PDR1	nothing	other	Class (V)
PMA1	nothing	other	Class (V)
CDH1	nothing	other	Class (V)
AML1	nothing	other	Class (V)
YGR012W	nothing	other	Class (V)
THG1	nothing	other	Class (V)
ERV1	nothing	other	Class (V)
YGR031W	nothing	other	Class (V)
YGR035C	nothing	other	Class (V)
SCM4	nothing	other	Class (V)
NNF2	nothing	other	Class (V)
RRP46	nothing	other	Class (V)
MDR1	nothing	other	Class (V)
SRB5	nothing	other	Class (V)
YGR111W	nothing	other	Class (V)
SHY1	nothing	other	Class (V)
ASN2	nothing	other	Class (V)
YGR126W	nothing	other	Class (V)
PHB1	nothing	other	Class (V)
CAF130	nothing	other	Class (V)
BTN2	nothing	other	Class (V)
TIF4631	nothing	other	Class (V)
ATF2	nothing	other	Class (V)
SER2	nothing	other	Class (V)
SPG1	nothing	other	Class (V)
YGR237C	nothing	other	Class (V)
TNA1	nothing	other	Class (V)
MAL13	nothing	other	Class (V)
YHL042W	nothing	other	Class (V)
ARN1	nothing	other	Class (V)
SBP1	nothing	other	Class (V)
GUT1	nothing	other	Class (V)
SNF6	nothing	other	Class (V)
RPS20	nothing	other	Class (V)
YHL010C	nothing	other	Class (V)
STP2	nothing	other	Class (V)
PAN5	nothing	other	Class (V)
OSH3, YHR073W-A	nothing	other	Class (V)
YHR080C	nothing	other	Class (V)
YHR087W	nothing	other	Class (V)
YNG2	nothing	other	Class (V)
HXT4	nothing	other	Class (V)
HXT1	nothing	other	Class (V)
GRE3	nothing	other	Class (V)
MSH1	nothing	other	Class (V)
YHR131C	nothing	other	Class (V)
ECM14	nothing	other	Class (V)
YSP1	nothing	other	Class (V)
KEL1	nothing	other	Class (V)
PTH1	nothing	other	Class (V)
NVJ1	nothing	other	Class (V)
UTP9	nothing	other	Class (V)
FMP34, YHR199C-A	nothing	other	Class (V)
MNL1	nothing	other	Class (V)
YIL161W	nothing	other	Class (V)
UBP7	nothing	other	Class (V)
ECM37	nothing	other	Class (V)

POG1	nothing	other	Class (V)
QDR2	nothing	other	Class (V)
RPI1	nothing	other	Class (V)
YIL108W	nothing	other	Class (V)
PFK26	nothing	other	Class (V)
FMC1	nothing	other	Class (V)
SEC6	nothing	other	Class (V)
YIL067C	nothing	other	Class (V)
FIS1	nothing	other	Class (V)
DFG10	nothing	other	Class (V)
PKP1	nothing	other	Class (V)
BAR1	nothing	other	Class (V)
SGN1	nothing	other	Class (V)
YIR003W	nothing	other	Class (V)
MSL1	nothing	other	Class (V)
DAL2	nothing	other	Class (V)
DCG1	nothing	other	Class (V)
YIR035C	nothing	other	Class (V)
YPS6	nothing	other	Class (V)
YJL171C	nothing	other	Class (V)
FBP26	nothing	other	Class (V)
IDS2	nothing	other	Class (V)
PBS2	nothing	other	Class (V)
MDV1	nothing	other	Class (V)
LSB6	nothing	other	Class (V)
PHS1	nothing	other	Class (V)
DLS1	nothing	other	Class (V)
MRPL8	nothing	other	Class (V)
TDH1	nothing	other	Class (V)
YJL047C-A, SNR60	nothing	other	Class (V)
CTK2	nothing	other	Class (V)
PRE3	nothing	other	Class (V)
APL1	nothing	other	Class (V)
YJR011C	nothing	other	Class (V)
BNA1	nothing	other	Class (V)
PET191	nothing	other	Class (V)
VPS55	nothing	other	Class (V)
PTK2	nothing	other	Class (V)
NPA3	nothing	other	Class (V)
EAF6	nothing	other	Class (V)
JHD2	nothing	other	Class (V)
ENT3	nothing	other	Class (V)
MNS1	nothing	other	Class (V)
BAT2	nothing	other	Class (V)
YJR149W	nothing	other	Class (V)
PEX1	nothing	other	Class (V)
ACP1	nothing	other	Class (V)
FAS1	nothing	other	Class (V)
SNU114	nothing	other	Class (V)
YKL171W	nothing	other	Class (V)
APE2	nothing	other	Class (V)
AVT3	nothing	other	Class (V)
Oct-01	nothing	other	Class (V)
YPK1	nothing	other	Class (V)
TEF4, SNR38	nothing	other	Class (V)
SMY1	nothing	other	Class (V)
MSN4	nothing	other	Class (V)
MDM35	nothing	other	Class (V)

PTM1	nothing	other	Class (V)
MAK11, SPT23	nothing	other	Class (V)
YPT52	nothing	other	Class (V)
YKR018C	nothing	other	Class (V)
IRS4	nothing	other	Class (V)
DAL80	nothing	other	Class (V)
KAE1	nothing	other	Class (V)
DYN1	nothing	other	Class (V)
YKR074W	nothing	other	Class (V)
HBS1	nothing	other	Class (V)
MLP1	nothing	other	Class (V)
MMP1	nothing	other	Class (V)
YLL053C, AQY2	nothing	other	Class (V)
SDH2	nothing	other	Class (V)
VPS13	nothing	other	Class (V)
ISA1	nothing	other	Class (V)
DPS1	nothing	other	Class (V)
ORC3	nothing	other	Class (V)
SSL1	nothing	other	Class (V)
LOT6	nothing	other	Class (V)
AAT2	nothing	other	Class (V)
MLH2	nothing	other	Class (V)
COX12	nothing	other	Class (V)
YLR053C	nothing	other	Class (V)
EMP46	nothing	other	Class (V)
YLR108C	nothing	other	Class (V)
YLR118C	nothing	other	Class (V)
DCN1	nothing	other	Class (V)
ENT2	nothing	other	Class (V)
HRD3	nothing	other	Class (V)
BNA5	nothing	other	Class (V)
ERF2	nothing	other	Class (V)
IRC20	nothing	other	Class (V)
RCK2	nothing	other	Class (V)
YSH1	nothing	other	Class (V)
YLR278C	nothing	other	Class (V)
IMH1	nothing	other	Class (V)
CDC25	nothing	other	Class (V)
CHS5	nothing	other	Class (V)
MID2	nothing	other	Class (V)
MDM30	nothing	other	Class (V)
CCW14	nothing	other	Class (V)
YLR392C	nothing	other	Class (V)
MAG2	nothing	other	Class (V)
CRN1	nothing	other	Class (V)
CTK3	nothing	other	Class (V)
NUP188	nothing	other	Class (V)
ARG81	nothing	other	Class (V)
ALO1	nothing	other	Class (V)
YML081W	nothing	other	Class (V)
FPR3	nothing	other	Class (V)
DAK1	nothing	other	Class (V)
GAL80	nothing	other	Class (V)
YML020W	nothing	other	Class (V)
ERG6	nothing	other	Class (V)
GIS4	nothing	other	Class (V)
YML003W, YML002W	nothing	other	Class (V)
MVP1	nothing	other	Class (V)

TAF4	nothing	other	Class (V)
PLB1	nothing	other	Class (V)
BUD22	nothing	other	Class (V)
STB4	nothing	other	Class (V)
YMR027W	nothing	other	Class (V)
YMR031C	nothing	other	Class (V)
FET3	nothing	other	Class (V)
KAR5	nothing	other	Class (V)
VPS20	nothing	other	Class (V)
ADH3	nothing	other	Class (V)
YMR086W	nothing	other	Class (V)
YTA12	nothing	other	Class (V)
YMR090W	nothing	other	Class (V)
AIP1	nothing	other	Class (V)
MTG1	nothing	other	Class (V)
YMR098C	nothing	other	Class (V)
YMR099C	nothing	other	Class (V)
ILV2	nothing	other	Class (V)
YMR111C	nothing	other	Class (V)
YMR122W-A	nothing	other	Class (V)
GAT2	nothing	other	Class (V)
PSO2	nothing	other	Class (V)
NDE1	nothing	other	Class (V)
YIM1	nothing	other	Class (V)
YMR155W	nothing	other	Class (V)
ATG16	nothing	other	Class (V)
HLJ1	nothing	other	Class (V)
YMR166C	nothing	other	Class (V)
ALD2	nothing	other	Class (V)
ECM5	nothing	other	Class (V)
GCV2	nothing	other	Class (V)
SGS1	nothing	other	Class (V)
GYL1	nothing	other	Class (V)
RAD14	nothing	other	Class (V)
CEF1	nothing	other	Class (V)
ESC1	nothing	other	Class (V)
TAF7	nothing	other	Class (V)
TRI1	nothing	other	Class (V)
RKR1	nothing	other	Class (V)
YMR258C	nothing	other	Class (V)
TPS3	nothing	other	Class (V)
YMR262W	nothing	other	Class (V)
RSN1	nothing	other	Class (V)
TMA23	nothing	other	Class (V)
DSK2	nothing	other	Class (V)
YME2	nothing	other	Class (V)
GLC8	nothing	other	Class (V)
ADH6	nothing	other	Class (V)
FIG4	nothing	other	Class (V)
RPL18B	nothing	other	Class (V)
YNL300W	nothing	other	Class (V)
MSB3	nothing	other	Class (V)
CAF40	nothing	other	Class (V)
CUS2	nothing	other	Class (V)
WSC2	nothing	other	Class (V)
BNI1	nothing	other	Class (V)
LYP1	nothing	other	Class (V)
NRD1	nothing	other	Class (V)

SIN4	nothing	other	Class (V)
SQS1	nothing	other	Class (V)
ATG4	nothing	other	Class (V)
ADE12	nothing	other	Class (V)
MGS1	nothing	other	Class (V)
IES2	nothing	other	Class (V)
PSY2	nothing	other	Class (V)
GCR2, YNL198C	nothing	other	Class (V)
DUG3	nothing	other	Class (V)
SRP1	nothing	other	Class (V)
YNL176C	nothing	other	Class (V)
NOP13	nothing	other	Class (V)
IBD2	nothing	other	Class (V)
CBK1	nothing	other	Class (V)
YCK2, GIM3	nothing	other	Class (V)
YNL144C	nothing	other	Class (V)
MEP2	nothing	other	Class (V)
FAR11	nothing	other	Class (V)
ESBP6	nothing	other	Class (V)
CYB5	nothing	other	Class (V)
LEU4	nothing	other	Class (V)
MET4	nothing	other	Class (V)
AVT4	nothing	other	Class (V)
RHO2	nothing	other	Class (V)
APJ1	nothing	other	Class (V)
MKS1	nothing	other	Class (V)
SFB2	nothing	other	Class (V)
YNL040W	nothing	other	Class (V)
YNL022C	nothing	other	Class (V)
ABZ1	nothing	other	Class (V)
YNR034W-A	nothing	other	Class (V)
ARC35	nothing	other	Class (V)
AGA1	nothing	other	Class (V)
LYS9	nothing	other	Class (V)
HOL1	nothing	other	Class (V)
YNR071C	nothing	other	Class (V)
YNR073C	nothing	other	Class (V)
YOL159C-A	nothing	other	Class (V)
YOL159C	nothing	other	Class (V)
YOL138C	nothing	other	Class (V)
YOL114C	nothing	other	Class (V)
WSC3	nothing	other	Class (V)
TRM10	nothing	other	Class (V)
MPD2	nothing	other	Class (V)
YOL087C	nothing	other	Class (V)
THP1	nothing	other	Class (V)
MET22	nothing	other	Class (V)
CRT10	nothing	other	Class (V)
APM4	nothing	other	Class (V)
GPD2	nothing	other	Class (V)
NTG2	nothing	other	Class (V)
YOL036W	nothing	other	Class (V)
SMC5	nothing	other	Class (V)
DIS3	nothing	other	Class (V)
YOL014W	nothing	other	Class (V)
ALG6	nothing	other	Class (V)
SGT2	nothing	other	Class (V)
STI1	nothing	other	Class (V)

WHI2	nothing	other	Class (V)
NOB1	nothing	other	Class (V)
SGT1	nothing	other	Class (V)
CYT1	nothing	other	Class (V)
MSA1	nothing	other	Class (V)
THI71	nothing	other	Class (V)
UFE1	nothing	other	Class (V)
DIA2	nothing	other	Class (V)
WHI5	nothing	other	Class (V)
VPS21	nothing	other	Class (V)
INP53	nothing	other	Class (V)
AZF1	nothing	other	Class (V)
RPT5	nothing	other	Class (V)
ORT1	nothing	other	Class (V)
ARP8	nothing	other	Class (V)
LSC1	nothing	other	Class (V)
SMP3	nothing	other	Class (V)
PDR5	nothing	other	Class (V)
PUP1	nothing	other	Class (V)
MTR10	nothing	other	Class (V)
YRR1	nothing	other	Class (V)
RPS28A	nothing	other	Class (V)
YRM1	nothing	other	Class (V)
RUD3	nothing	other	Class (V)
RCN2	nothing	other	Class (V)
ABP140	nothing	other	Class (V)
YOR246C	nothing	other	Class (V)
CLP1	nothing	other	Class (V)
CDC31	nothing	other	Class (V)
RPN8	nothing	other	Class (V)
MBF1	nothing	other	Class (V)
YOR305W	nothing	other	Class (V)
SLY41	nothing	other	Class (V)
HSD1	nothing	other	Class (V)
FAA1	nothing	other	Class (V)
PUT4	nothing	other	Class (V)
SOG2	nothing	other	Class (V)
NDD1	nothing	other	Class (V)
YOR385W	nothing	other	Class (V)
YPL272C	nothing	other	Class (V)
MDL2	nothing	other	Class (V)
PLC1	nothing	other	Class (V)
KEL3	nothing	other	Class (V)
YAH1	nothing	other	Class (V)
SRP68	nothing	other	Class (V)
HSP82	nothing	other	Class (V)
YAR1	nothing	other	Class (V)
TYW1	nothing	other	Class (V)
HRR25	nothing	other	Class (V)
RPL7B, SNR59	nothing	other	Class (V)
OXR1	nothing	other	Class (V)
RSA1	nothing	other	Class (V)
PET20	nothing	other	Class (V)
PEP4	nothing	other	Class (V)
GIP3	nothing	other	Class (V)
RPL5	nothing	other	Class (V)
KAP120	nothing	other	Class (V)
VPS30	nothing	other	Class (V)

PEX25	nothing	other	Class (V)
GDE1	nothing	other	Class (V)
YPL108W, YPL107W	nothing	other	Class (V)
FMP14	nothing	other	Class (V)
EEB1	nothing	other	Class (V)
SEC16	nothing	other	Class (V)
GCR1	nothing	other	Class (V)
LPE10	nothing	other	Class (V)
DIG1	nothing	other	Class (V)
ISM1	nothing	other	Class (V)
YPL034W	nothing	other	Class (V)
SWI1	nothing	other	Class (V)
RLF2	nothing	other	Class (V)
ATP20	nothing	other	Class (V)
YPR022C	nothing	other	Class (V)
GLN1	nothing	other	Class (V)
YPR036W-A	nothing	other	Class (V)
ROX1	nothing	other	Class (V)
YPR071W	nothing	other	Class (V)
MDM36	nothing	other	Class (V)
SUA7	nothing	other	Class (V)
COG4	nothing	other	Class (V)
AXL1	nothing	other	Class (V)
VPS66	nothing	other	Class (V)
TAZ1	nothing	other	Class (V)
YPR145C-A	nothing	other	Class (V)
NCE102	nothing	other	Class (V)
YPR157W	nothing	other	Class (V)
KRE6	nothing	other	Class (V)
ORC4	nothing	other	Class (V)
JIP5	nothing	other	Class (V)
AOS1	nothing	other	Class (V)
OPT2	nothing	other	Class (V)
SGE1	nothing	other	Class (V)
YAL019W-A, FUN30	nothing	NA	Class (V)
YAR019W-A	nothing	NA	Class (V)
YBL054W, YBL053W	nothing	NA	Class (V)
YBL008W-A, HIR1	nothing	NA	Class (V)
ETR1, YBR027C	nothing	NA	Class (V)
YBR027C, YBR028C	nothing	NA	Class (V)
YBR056W-A	nothing	NA	Class (V)
YBR063C, ECM2	nothing	NA	Class (V)
YBR076C-A	nothing	NA	Class (V)
YBR076C-A, SLM4	nothing	NA	Class (V)
YBR090C, MRS5	nothing	NA	Class (V)
YBR109W-A, ALG1	nothing	NA	Class (V)
YBR147W, YSW1	nothing	NA	Class (V)
YBR190W, RPL21A, YBR191W	nothing	NA	Class (V)
YBR292C	nothing	NA	Class (V)
YCR006C, SUF2	nothing	NA	Class (V)
SNR33	nothing	NA	Class (V)
YCR024C-B, PMP1	nothing	NA	Class (V)
YCR025C, NPP1	nothing	NA	Class (V)
SNR65	nothing	NA	Class (V)
SNR189	nothing	NA	Class (V)
HMRA1	nothing	NA	Class (V)
YDL187C	nothing	NA	Class (V)
SNR63	nothing	NA	Class (V)

PRM7, YDL038C, BSC1	nothing	NA	Class (V)
RCR2, YDR003W-A	nothing	NA	Class (V)
GCV1, YDR020C	nothing	NA	Class (V)
SES1, FYV1	nothing	NA	Class (V)
YDR042C, SNR47	nothing	NA	Class (V)
SNR47	nothing	NA	Class (V)
SLU7, tR(ACG)D	nothing	NA	Class (V)
YDR133C, YDR134C	nothing	NA	Class (V)
CDC1, YDR182W-A	nothing	NA	Class (V)
MFB1, YDR220C	nothing	NA	Class (V)
YDR249C, YDR250C	nothing	NA	Class (V)
SNR13	nothing	NA	Class (V)
YDR524C-B, YDR524C-A	nothing	NA	Class (V)
FIT1, YDR535C	nothing	NA	Class (V)
YEL068C	nothing	NA	Class (V)
SNR67	nothing	NA	Class (V)
GLY1, YEL045C	nothing	NA	Class (V)
HYP2, YEL033W	nothing	NA	Class (V)
YEL033W	nothing	NA	Class (V)
YEL028W	nothing	NA	Class (V)
RPR1	nothing	NA	Class (V)
YELCtau1, YEL020C	nothing	NA	Class (V)
YEL008C-A	nothing	NA	Class (V)
YER034W, EDC2	nothing	NA	Class (V)
YER066C-A	nothing	NA	Class (V)
AVT6, YER119C-A	nothing	NA	Class (V)
SNR4	nothing	NA	Class (V)
SNR52	nothing	NA	Class (V)
SCR1	nothing	NA	Class (V)
YER152C, PET122	nothing	NA	Class (V)
YFR020W	nothing	NA	Class (V)
YGL204C	nothing	NA	Class (V)
YGL188C, COX4	nothing	NA	Class (V)
YGLWtau2	nothing	NA	Class (V)
SNR82	nothing	NA	Class (V)
SOE1	nothing	NA	Class (V)
YGL088W, SNR10	nothing	NA	Class (V)
SNR39B	nothing	NA	Class (V)
YGR039W, KSS1	nothing	NA	Class (V)
RME1, YGR045C	nothing	NA	Class (V)
YGR045C	nothing	NA	Class (V)
YGR050C	nothing	NA	Class (V)
SNR48	nothing	NA	Class (V)
tL(GAG)G	nothing	NA	Class (V)
YGR107W	nothing	NA	Class (V)
SNR7-L, SNR7-S	nothing	NA	Class (V)
YGR269W, YTA7	nothing	NA	Class (V)
SHU1, YHL005C	nothing	NA	Class (V)
YHR007C-A, SOD2	nothing	NA	Class (V)
YHRWtau3	nothing	NA	Class (V)
YHR032W, YHR032W-A	nothing	NA	Class (V)
YHR050W-A, COX6	nothing	NA	Class (V)
YHR130C, YHR131C	nothing	NA	Class (V)
SNR32	nothing	NA	Class (V)
SNR71	nothing	NA	Class (V)
CTR2, YHR175W-A	nothing	NA	Class (V)
YHR180W	nothing	NA	Class (V)
YIL168W, SDL1	nothing	NA	Class (V)

YIL165C, NIT1	nothing	NA	Class (V)
YIL156W-A, YIL156W-B	nothing	NA	Class (V)
SNR68	nothing	NA	Class (V)
YIL100W, SGA1	nothing	NA	Class (V)
YIL087C, YIL086C	nothing	NA	Class (V)
YIL086C, KTR7	nothing	NA	Class (V)
YIL028W	nothing	NA	Class (V)
YIL025C, YIL024C	nothing	NA	Class (V)
MUC1, YIR020C	nothing	NA	Class (V)
YIR021W-A, SEC11	nothing	NA	Class (V)
YIR043C	nothing	NA	Class (V)
YJL216C, YJL215C	nothing	NA	Class (V)
MBB1	nothing	NA	Class (V)
SNR128	nothing	NA	Class (V)
SNR190	nothing	NA	Class (V)
tD(GUC)J1	nothing	NA	Class (V)
MPS3, YJL016W	nothing	NA	Class (V)
ECM27, YJR107W	nothing	NA	Class (V)
SNR64	nothing	NA	Class (V)
TPK3, YKL165C-A	nothing	NA	Class (V)
YKL165C-A	nothing	NA	Class (V)
tE(UUC)K	nothing	NA	Class (V)
tK(CUU)K	nothing	NA	Class (V)
YNK1, YKL066W	nothing	NA	Class (V)
YKL044W	nothing	NA	Class (V)
YKL044W, PHD1	nothing	NA	Class (V)
SNR69	nothing	NA	Class (V)
URA6, YKL023C-A	nothing	NA	Class (V)
SNR87	nothing	NA	Class (V)
SIS2, YKR073C	nothing	NA	Class (V)
GTT2, YLL059C	nothing	NA	Class (V)
YLL059C	nothing	NA	Class (V)
YLL017W, SDC25	nothing	NA	Class (V)
YLR046C, FRE8	nothing	NA	Class (V)
SNR79	nothing	NA	Class (V)
SNR6	nothing	NA	Class (V)
YLR111W, YLR112W	nothing	NA	Class (V)
YLR122C, YLR123C	nothing	NA	Class (V)
ACE2, YLR132C	nothing	NA	Class (V)
YLR202C, MSS51	nothing	NA	Class (V)
YLRCtau1, ECM22	nothing	NA	Class (V)
YLR296W	nothing	NA	Class (V)
YLR311C	nothing	NA	Class (V)
SNR55, SNR57	nothing	NA	Class (V)
FKS1, YLR342W-A	nothing	NA	Class (V)
YLR342W-A, GAS2	nothing	NA	Class (V)
YLR366W, RPS22B	nothing	NA	Class (V)
YLR402W, SFP1	nothing	NA	Class (V)
SNR85	nothing	NA	Class (V)
YML089C	nothing	NA	Class (V)
YML079W, CPR3	nothing	NA	Class (V)
CDC5, YMR001C-A	nothing	NA	Class (V)
SNR78	nothing	NA	Class (V)
SNR77	nothing	NA	Class (V)
SNR76	nothing	NA	Class (V)
SNR75	nothing	NA	Class (V)
SNR73, YMR013W-A	nothing	NA	Class (V)
SNR72	nothing	NA	Class (V)

HOF1, YMR030W-A	nothing	NA	Class (V)
YMR075C-A, PDS5	nothing	NA	Class (V)
YMR103C, YPK2	nothing	NA	Class (V)
YMR316C-B	nothing	NA	Class (V)
YNL320W, YNL319W	nothing	NA	Class (V)
SNR40	nothing	NA	Class (V)
SNR19	nothing	NA	Class (V)
RHO5, YNL179C	nothing	NA	Class (V)
YNL143C	nothing	NA	Class (V)
RAS2, YNL097C-B	nothing	NA	Class (V)
NME1	nothing	NA	Class (V)
SNR66	nothing	NA	Class (V)
YNR068C, BSC5	nothing	NA	Class (V)
YOL153C	nothing	NA	Class (V)
SNR81	nothing	NA	Class (V)
SNR50	nothing	NA	Class (V)
YOR008W-B, TIR4	nothing	NA	Class (V)
YOR012W, IRC11	nothing	NA	Class (V)
YOR034C-A, SHE4	nothing	NA	Class (V)
tK(UUU)O	nothing	NA	Class (V)
TGL5, YOR082C	nothing	NA	Class (V)
tD(GUC)O	nothing	NA	Class (V)
SNR8	nothing	NA	Class (V)
SNR31	nothing	NA	Class (V)
SNR5	nothing	NA	Class (V)
YOR343C	nothing	NA	Class (V)
YOR376W-A, ATF1	nothing	NA	Class (V)
PRM3, YPL191C	nothing	NA	Class (V)
YPL152W-A, RRD2	nothing	NA	Class (V)
YPR053C	nothing	NA	Class (V)
SNR51, SNR70	nothing	NA	Class (V)
SNR70	nothing	NA	Class (V)
SNR41	nothing	NA	Class (V)
SNR45	nothing	NA	Class (V)
YPR169W-A, YPR170W-B, YPR	nothing	NA	Class (V)
YPR195C	nothing	NA	Class (V)
YPR196W, YPRWtau4	nothing	NA	Class (V)

Table S3: ncRNAs from Xu et al. 2009 plus newly identified ncRNAs from this study

# Genomic Coordinates				# Annotatior	# type in Xu (	# our annota	# differential expression (log2 fold change)				
start	end	chr	strand	commonNan	type_Xu	type_new	rrp6vsWT	set1vsWT	rrp6set1vsrrp6	rrp6hda2vsrrp6	rrp6rpd3vsrrp6
30229	31429	1	-	CUT437	CUTs	CUTs	0.242194403	0.095452996	1.230136904	1.965135518	-0.395569711
32973	34349	1	-	SUT435	SUTs	SUTs	1.33322871	-0.780472522	-1.036500607	-0.597073083	-1.134340275
34349	34749	1	-	CUT438	CUTs	CUTs	2.377176242	-0.238283606	0.109067525	-0.157624012	-0.852801063
35789	36349	1	-	CUT439	CUTs	CUTs	2.029461631	0.480346047	-1.429800824	-0.77593634	0.960804352
36349	39029	1	-	unknown	no_ovlp	SUTs	0.326084246	0.318603595	0.347440919	0.345848525	1.048134858
42181	42741	1	-	unknown	no_ovlp2	CUTs	1.657677288	-0.078196347	-0.942261966	-0.506516599	-0.702493585
42741	43565	1	-	ACS1	ORF-T	ORF-T	-1.316366781	-0.899397933	1.968309022	0.877382344	2.42602343
43581	48885	1	-	ACS1	ORF-T	ORF-T	-0.699213955	0.033616577	2.903907623	1.130529471	4.85854128
45069	48885	1	-	unknown	no_ovlp2	SUTs	-0.047901508	0.289854571	0.050035224	0.096420592	0.105262055
51731	52643	1	-	YAL049C	ORF-T	ORF-T	0.243273369	0.047423689	0.088272651	-0.025618036	0.692957992
52643	54779	1	-	GEM1	ORF-T	ORF-T	0.235313267	-0.181208889	-0.389941377	-0.236941587	-0.837090205
54939	57003	1	-	SPC72	ORF-T	ORF-T	0.574299922	0.265546349	0.072063472	-0.29872094	0.050239004
57003	57379	1	-	YAL046C	ORF-T	ORF-T	-0.121210779	0.024166259	0.338437921	-0.220540584	-0.276563417
57867	58475	1	-	GCV3	ORF-T	ORF-T	0.863724331	-0.745527054	-0.368155888	-0.047520481	-0.603626137
58635	61075	1	-	PTA1	ORF-T	ORF-T	0.041553893	-0.034947746	-0.23334801	-0.047264312	0.036001408
65635	67843	1	-	CLN3	ORF-T	ORF-T	-0.274852879	-0.65662359	-0.60763939	-0.39582417	-0.081435353
68651	69587	1	-	CYC3	ORF-T	ORF-T	-0.434266553	0.188659561	0.380303399	-0.016394041	0.092410411
69587	71755	1	-	unknown	no_ovlp	SUTs	0.706799936	0.385420314	0.231282504	-0.095184956	0.100585169
71755	73051	1	-	unknown	no_ovlp	SUTs	0.633748056	0.937720956	-0.387449115	0.492304156	-0.366997367
74867	76195	1	-	RBG1	ORF-T	ORF-T	-0.194436396	-0.280125765	-0.114098523	0.156690767	0.373984542
76195	77563	1	-	unknown	no_ovlp	SUTs	-0.229931569	0.376170003	0.09891584	0.232920539	0.411324974
82027	82563	1	-	FUN19	ORF-T	ORF-T	0.492936748	0.114813887	-0.209860323	-0.213748714	-0.702442359
83219	84523	1	-	PRP45	ORF-T	ORF-T	0.112047085	-0.236268054	-0.103473101	-0.08021511	0.081345581
84643	87075	1	-	GIP4	ORF-T	ORF-T	0.312970375	-0.148404066	0.121651963	0.103400387	0.00912995
87075	87707	1	-	unknown	no_ovlp	SUTs	-0.160147028	0.387094943	0.107136462	0.088398184	0.110927354
87883	92499	1	-	MYO4	ORF-T	ORF-T	-0.075589912	-0.305568755	-0.12608234	-0.192312358	0.057460995
92499	94771	1	-	unknown	no_ovlp	SUTs	0.768664493	0.352628925	-0.685824416	-0.53941687	-0.441640376
95491	99787	1	-	YAL026C-A, [	ORF-T	ORF-T	-0.237884183	-0.379322429	-0.725557449	-0.022762472	-0.339818745
100035	101171	1	-	MAK16	ORF-T	ORF-T	0.303611674	-0.198089002	-0.870493767	-0.179822992	0.564005758
101395	105963	1	-	LTE1	ORF-T	ORF-T	-0.334635279	-0.562066215	-0.149927783	-0.192481586	0.872518125
106155	108667	1	-	PMT2	ORF-T	ORF-T	-0.995022312	-0.412260015	-0.839413579	-0.04090307	-0.054578754
108835	110451	1	-	FUN26	ORF-T	ORF-T	-0.251848212	-0.329893735	-0.540409553	-0.34652982	-0.311482935
110627	113403	1	-	CCR4	ORF-T	ORF-T	-0.253992621	-0.286694337	-0.273587961	0.046621557	-0.007163201
113403	114619	1	-	ATS1	ORF-T	ORF-T	-0.164903563	-0.115898746	-0.018339959	-0.574804731	0.217529008
124459	125083	1	-	unknown	no_ovlp	CUTs	1.042084546	0.105358429	-0.666029075	-0.627510242	1.2712865
126819	128123	1	-	NTG1	ORF-T	ORF-T	0.004046284	-0.565396449	-0.114411884	-0.187214856	0.011153431
128123	129067	1	-	SYN8	ORF-T	ORF-T	-0.20710287	-0.296951918	-0.236791112	-0.181382884	-0.139660101
132235	132603	1	-	unknown	no_ovlp	CUTs	1.025381537	-0.325485186	-0.745121859	-0.933188491	0.832147638
134827	135683	1	-	MDM10	ORF-T	ORF-T	-0.116947393	-0.050945627	0.279493073	0.049279992	-0.226049366
137579	138427	1	-	ERP2	ORF-T	ORF-T	-0.639059525	-0.700510156	-0.378687094	-0.273276935	-0.004834134
139371	141491	1	-	SSA1	ORF-T	ORF-T	-0.471845932	-0.597020911	0.367386646	0.164019719	0.037459408
147595	151211	1	-	TFC3	ORF-T	ORF-T	0.031340624	-0.029331417	-0.145088919	-0.096751997	-0.794218076
151635	152027	1	-	CUT441	CUTs	CUTs	1.85795696	0.088134298	-0.249201363	-0.299143779	-1.008887053
153995	154779	1	-	ERP1	ORF-T	ORF-T	-0.356307498	-0.423414762	-0.015376121	-0.153593926	0.157935288
156707	158683	1	-	RFA1	ORF-T	ORF-T	-0.490891332	-0.221791179	0.221950311	0.020872814	0.704950585
160243	166163	1	-	unknown	no_ovlp	SUTs	0.222476841	0.007217338	-0.161760565	-0.172620488	-0.854678838
166579	169011	1	-	BUD14	ORF-T	ORF-T	0.211121905	-0.032870724	-0.002011864	0.12051242	0.409072267
170259	171715	1	-	KIN3	ORF-T	ORF-T	-0.199053137	-0.035283632	-0.132668061	-0.235013833	0.549258319
173027	175163	1	-	CDC15	ORF-T	ORF-T	-0.05187232	-0.223681677	0.095422284	0.386962132	-0.299654396
176539	180051	1	-	unknown	no_ovlp	SUTs	0.464807828	-0.096558753	-0.226273602	0.201088933	-1.280964127
182971	183539	1	-	unknown	no_ovlp	SUTs	0.61360095	-1.007521116	-0.258552176	0.143724442	-1.71360956
183539	184715	1	-	unknown	no_ovlp	SUTs	0.957647616	0.046618774	-0.640275841	0.111483509	-0.984527042
185963	192195	1	-	SUT436	SUTs	SUTs	-0.19134258	0.550946337	0.686985568	0.505395578	-0.091366262
25465	28129	1	+	unknown	no_ovlp	SUTs	0.940573591	-0.28890244	-0.462964315	-0.005673355	-2.072297645
30073	30777	1	+	CUT001	CUTs	CUTs	1.916095111	0.229648536	-0.6722735	0.112681345	-1.635819665
31153	31633	1	+	GDH3	ORF-T	ORF-T	0.487920353	0.326072313	1.718074978	1.881166283	-1.430917432
33513	34689	1	+	BDH2	ORF-T	ORF-T	-1.090226059	0.085647952	2.725615299	2.452284527	-0.140397116
34689	35073	1	+	unknown	no_ovlp	CUTs	1.084675195	0.142667186	1.368613212	0.932452961	-0.144506848
35073	36417	1	+	BDH1	ORF-T	ORF-T	-0.02139427	-0.940398846	0.407103697	0.393198363	-0.846470271
36545	37297	1	+	ECM1	ORF-T	ORF-T	0.212569242	-0.144159784	-0.399453074	-0.03922675	0.646621785
37481	39081	1	+	CNE1	ORF-T	ORF-T	-0.527381358	-0.178697753	-0.206570181	0.060221362	-0.452428392
39225	42001	1	+	GPB2	ORF-T	ORF-T	-0.028905015	-0.388818881	0.027040069	0.32724467	-0.352175544
42161	42721	1	+	PEX22	ORF-T	ORF-T	-0.203100534	-0.049628307	0.794768464	-0.006103062	0.965141619
43449	45217	1	+	SUT002	SUTs	SUTs	1.604269281	0.316706616	-0.705970674	-0.177207081	-1.827659391
45769	48321	1	+	FLC2	ORF-T	ORF-T	-0.300856985	-0.223749027	-0.216175778	0.004827902	0.207231391
48553	51855	1	+	OAF1	ORF-T	ORF-T	-0.07132727	-0.575141259	0.130776136	0.032980483	-0.214312901
57143	57535	1	+	unknown	no_ovlp	SUTs	0.53683619	-0.253321769	-0.338882995	-0.750964211	-0.167515953
57535	57959	1	+	YAL044W-A	ORF-T	ORF-T	1.015168124	1.020981033	0.187884013	-0.204570097	-0.243399818
61295	62655	1	+	ERV46	ORF-T	ORF-T	-0.296099594	-0.341846556	-0.125816771	-0.086373674	0.138282231
62791	65447	1	+	CDC24	ORF-T	ORF-T	-0.106691683	-0.600091679	-0.30696832	-0.338192525	-0.178985998
68727	69511	1	+	SUT003	SUTs	SUTs	0.922877528	-0.512775816	-0.47375143	-0.511440134	-0.751002464
71775	73447	1	+	CDC19	ORF-T	ORF-T	-0.388251988	0.027881426	-0.141956219	-0.064945027	0.010467036
73447	73935	1	+	unknown	no_ovlp2	SUTs	0.508860352	-0.006946188	-0.525353323	-0.132517662	-0.722825059
73935	74967	1	+	YAL037W	ORF-T	ORF-T	0.963700721	0.518462528	0.5325524	-0.297563273	-1.632162971
76407	79559	1	+	FUN12	ORF-T	ORF-T	-0.25844418	-0.219007672	-0.575018087	0.153282095	-0.157425604
79719	80655	1	+	MTW1	ORF-T	ORF-T	-0.211565513	-0.647477892	-0.107438364	-0.036270372	0.753540868
82271	82711	1	+	unknown	no_ovlp	SUTs	0.4818482	-0.266664463	0.355817017	0.259442609	0.870014165

82711	83311	1	+	POP5	ORF-T	ORF-T	0.938338438	0.092493671	-0.177474878	-0.165663018	-0.076701833
87503	87807	1	+	SNC1	ORF-T	ORF-T	-0.102721997	-0.088064261	0.485977181	0.108319886	0.652366232
92871	94463	1	+	FRT2	ORF-T	ORF-T	-0.814559346	-0.919441787	0.400961918	0.469651874	0.190298168
94663	95567	1	+	YAL027W	ORF-T	ORF-T	0.142075655	-0.37783192	0.166837332	-0.025129476	-0.474195162
112783	113583	1	+	unknown	no_ovlp	SUTs	-0.297333475	0.515668752	0.014168129	0.218592217	0.210507037
113583	114823	1	+	unknown	no_ovlp	SUTs	0.660192314	0.14243152	-0.633899239	-0.293985122	0.379955912
114823	118399	1	+	YAL019W-A,	other	other	0.050287558	-0.132828775	-0.65057111	-0.109568227	0.363595507
119871	121983	1	+	PSK1	ORF-T	ORF-T	-0.240558785	-0.430649933	0.339808848	0.101865224	-0.015957694
124799	126903	1	+	TPD3	ORF-T	ORF-T	0.001741464	-0.407411895	0.001046255	0.156594814	-0.020143072
129247	130591	1	+	DEP1	ORF-T	ORF-T	-0.372121386	-0.300855891	-0.085109161	0.078471806	0.098838881
130751	132087	1	+	CYS3	ORF-T	ORF-T	-0.435330661	-0.16912399	-0.413477026	-0.175553992	0.18809805
132207	134143	1	+	SWC3	ORF-T	ORF-T	0.092322985	0.057356193	-0.391186543	-0.220616872	0.023649947
135847	136759	1	+	SPO7	ORF-T	ORF-T	-0.188331674	-0.058744112	0.026328873	-0.325465088	-0.304383566
136919	137639	1	+	FUN14	ORF-T	ORF-T	-0.2171083	-0.346170179	0.152640182	0.025308605	-0.284821835
142631	143447	1	+	EFB1, SNR18	ORF-T	ORF-T	-0.1233822	-0.302062245	-0.373064433	-0.250538217	-0.411312286
143607	144895	1	+	VPS8	ORF-T	ORF-T	-0.286937101	-0.496883484	-0.205546505	-0.193141466	-0.279208823
152223	153959	1	+	NUP60	ORF-T	ORF-T	0.030176338	-0.279442566	-0.350604479	-0.040933024	-0.223961554
154943	156855	1	+	SWD1	ORF-T	ORF-T	-0.22015235	-0.580832135	-0.245243478	-0.169342375	0.182838201
158879	159879	1	+	SEN34	ORF-T	ORF-T	-0.599467497	-0.428086779	-0.190721643	-0.184299612	1.05216177
169207	170495	1	+	ADE1	ORF-T	ORF-T	0.976813185	0.478163072	-0.142063719	-0.194206432	-0.435898847
175319	176223	1	+	YAR019W-A	other	other	0.231372666	-0.895224034	0.161326638	-0.200598205	-0.352622621
180255	180839	1	+	CUT005	CUTs	CUTs	1.878966199	0.19043775	-0.029694592	0.273169128	-1.348630647
183623	184639	1	+	unknown	no_ovlp	SUTs	-0.043391328	-0.751445174	0.260511632	0.040453519	-1.026103882
184879	185815	1	+	YAR028W, YA	ORF-T	ORF-T	0.115913236	-0.520912953	-0.292472988	0.440259434	-1.56925226
190663	192599	1	+	YAT1	ORF-T	ORF-T	-0.600266075	0.558261154	0.739266212	0.374066888	0.803349553
192391	192599	1	+	unknown	no_ovlp2	SUTs	0.440103101	2.075762439	0.193396196	0.007500407	0.134833535
192599	196663	1	+	SWH1	ORF-T	ORF-T	-0.149336893	0.073731094	0.15768059	0.190424012	0.193415149
196255	196663	1	+	unknown	no_ovlp2	SUTs	-0.27400477	-0.632430215	-0.008381154	0.105089029	-0.016359326
202775	210655	1	+	unknown	no_ovlp	CUTs	1.30370693	-0.124829686	-0.513015348	0.233044658	-2.925275059
9829	10549	2	-	YBL107C	ORF-T	ORF-T	0.163510652	-0.613434013	-0.483830217	-0.633500006	-1.558462392
10853	14133	2	-	SRO77	ORF-T	ORF-T	0.184604587	0.081060708	-0.174849942	-0.158616929	0.556244217
13917	14133	2	-	unknown	no_ovlp2	SUTs	-0.258618434	-1.288557392	-0.372058828	-0.126602707	-0.429497332
14133	17941	2	-	PKC1	ORF-T	ORF-T	-0.103952663	-0.382947422	-0.230046991	0.099498973	-0.294444516
19717	21269	2	-	YBL104C	ORF-T	ORF-T	0.142066474	-0.268236007	-0.410953942	-0.445794994	-0.48950185
22645	23797	2	-	RTG3	ORF-T	ORF-T	0.048925611	-0.741344361	-0.464314872	-0.299033062	-0.567300957
24845	28509	2	-	ECM21	ORF-T	ORF-T	-0.034407169	-0.145823605	0.375734433	0.062451575	0.232925182
28509	28773	2	-	unknown	no_ovlp	SUTs	0.534008357	0.166175267	0.598129805	0.14241516	0.043879438
44165	45013	2	-	ROX3	ORF-T	ORF-T	-0.096301351	-0.369607634	-0.065727935	-0.21391047	0.653436133
46517	47277	2	-	SCS22	ORF-T	ORF-T	0.164540295	0.127646416	0.009481133	-0.178490713	-0.366380493
47277	48653	2	-	MAP2	ORF-T	ORF-T	-0.468718825	-0.40592598	-0.246633202	-0.03507566	0.138777389
48653	49429	2	-	unknown	no_ovlp	CUTs	1.842527303	0.070177784	-0.905561156	-0.767385493	-0.970439284
51021	59541	2	-	TEL1	ORF-T	ORF-T	0.307491702	0.039142055	-0.280648457	-0.310745763	0.156526366
60189	60709	2	-	RPL23A	ORF-T	ORF-T	0.692695903	-0.239543754	-0.976914214	-0.678725212	-0.082785629
61165	62693	2	-	YBL086C	ORF-T	ORF-T	0.070736983	-0.740837879	0.525277292	0.442808801	0.496097337
68357	69549	2	-	CDC27	ORF-T	ORF-T	-0.463565755	-0.586938382	0.639991431	0.56604235	0.681047454
69741	71165	2	-	YBL083C, AL(	ORF-T	ORF-T	-0.562949605	-0.458963	-0.374733138	-0.30887829	0.668263909
73029	74709	2	-	PET112	ORF-T	ORF-T	-0.826807373	-0.390746876	0.492311758	-0.203601203	0.251297344
80157	80733	2	-	ATG8	ORF-T	ORF-T	-0.283194902	-0.297345491	0.944349684	0.294992422	0.336334468
80957	84301	2	-	ILS1	ORF-T	ORF-T	-0.066565656	-0.20516536	-0.777915004	-0.025753145	-0.356738991
84397	87773	2	-	SSA3, AAR2	ORF-T	ORF-T	0.68726155	-0.136908684	1.225310173	-0.083284548	0.378006632
89125	89493	2	-	RPS8A, YBL0	other	other	0.989000629	-0.87947766	-1.072859256	-1.497317178	-0.201047375
91149	92197	2	-	unknown	no_ovlp	CUTs	1.081263179	-0.090476453	-0.399847678	-0.246784086	0.263639207
93565	96142	2	-	UBP13	ORF-T	ORF-T	-0.144073827	-0.085779636	-0.065047305	-0.185326076	0.325371826
96358	100147	2	-	SEF1	ORF-T	ORF-T	-0.267747864	-0.197637614	0.011927135	0.002132621	0.147037978
100355	101219	2	-	PRX1	ORF-T	ORF-T	0.502273677	-0.053902142	1.336282517	0.408182004	1.034438936
105227	107507	2	-	SKT5	ORF-T	ORF-T	0.113274562	-0.005009908	-0.361263513	-0.132668107	0.033464608
109867	110419	2	-	YBL059C-A	ORF-T	ORF-T	-0.160228461	-0.358812254	0.139916548	0.096211014	0.129286083
110419	111203	2	-	unknown	no_ovlp	SUTs	-0.307538574	-1.08929891	-0.619189916	-0.509180769	0.180534857
112731	113427	2	-	PTH2	ORF-T	ORF-T	-0.168680878	-0.612549094	-0.517508191	-0.118966618	0.364324819
115467	116803	2	-	YBL055C	ORF-T	ORF-T	0.18875048	-0.333477588	-0.359626243	0.053880607	-0.08742076
119355	122115	2	-	SAS3	ORF-T	ORF-T	-0.088899375	-0.390561527	-0.705215316	-0.363884303	0.040453272
122323	124835	2	-	PIN4	ORF-T	ORF-T	-0.127984346	0.025127436	0.083690681	0.110560628	-0.149343246
127691	132211	2	-	EDE1	ORF-T	ORF-T	-0.484844304	-0.116639353	-0.211875322	0.003523343	-0.051081238
133851	135731	2	-	COR1	ORF-T	ORF-T	-0.120322523	-0.058702586	0.514338318	0.094297314	0.221966776
138163	140339	2	-	FUI1	ORF-T	ORF-T	-0.03268552	-0.168916748	-0.703651517	-0.21729861	0.213306217
142043	142747	2	-	ERD2	ORF-T	ORF-T	-0.322427199	-0.196291349	0.053887573	-0.100245707	0.353863526
143851	145787	2	-	URA7, YBL03	ORF-T	ORF-T	0.306607401	-0.063408912	-0.694548698	0.019627677	0.285150626
146491	146955	2	-	CUT444	CUTs	CUTs	1.922445748	-0.098366221	-0.617941692	0.416280115	0.358179199
150307	151235	2	-	YBL036C	ORF-T	ORF-T	-0.034294992	-0.812866681	-0.525349823	-0.074324469	-0.112802053
151235	153739	2	-	unknown	no_ovlp2	SUTs	-0.461224809	-0.079436662	-0.605177912	-0.17064064	0.444466614
153739	158451	2	-	STU1	ORF-T	ORF-T	-0.308128113	-0.146530578	0.03381218	-0.07954181	0.579493146
158587	159715	2	-	RIB1	ORF-T	ORF-T	-0.118810589	-0.493130002	-0.109163068	0.076138912	0.099465274
159715	161379	2	-	unknown	no_ovlp	SUTs	0.211243797	0.676855485	0.083228548	0.671725903	0.894921831
162755	164147	2	-	PET9	ORF-T	ORF-T	-0.532025895	-0.172597428	0.306934635	0.092339604	-0.007990582
164347	164875	2	-	YBL029C-A	ORF-T	ORF-T	0.039649861	-0.677393251	0.547258586	-0.225344347	-0.334045295
167283	167883	2	-	YBL028C	ORF-T	ORF-T	0.707936938	-0.83556287	-1.056688257	-0.314042783	-0.269982642
169451	170035	2	-	SUT440	SUTs	SUTs	0.663585241	-0.289658598	-0.053847664	-0.097515139	-0.210896781
170035	170371	2	-	unknown	no_ovlp	CUTs	1.308644844	0.089442606	0.597311592	-1.098722006	-1.064965935
174779	177579	2	-	MCM2	ORF-T	ORF-T	-0.170000398	0.110105824	-0.393875933	0.092752401	0.39068685
177811	181403	2	-	PIM1	ORF-T	ORF-T	-0.619502679	-0.491841963	0.038355589	-0.06273891	0.043105988
181499	182139	2	-	HAP3	ORF-T	ORF-T	-0.218543053	-0.622320995	0.195483357	-0.257693555	0.36087781
185827	186499	2	-	POP8	ORF-T	ORF-T	-0.389853062	-0.798816756	-0.38549515	-0.107962335	0.542650127

186779	191763	2 -	PEP1	ORF-T	ORF-T	-0.366039184	-0.812031338	-0.573149211	0.159358035	-0.245369915
191763	192179	2 -	unknown	no_ovlp	CUTs	1.037475957	0.505098556	-0.265350645	-0.378081631	-1.099342056
198955	201771	2 -	RRN6	ORF-T	ORF-T	0.764474897	-0.023055694	-0.564957752	-0.477528969	0.179008249
201771	202979	2 -	unknown	no_ovlp	SUTs	-0.515432317	0.140779166	0.277701774	0.263050508	1.503158329
205940	206964	2 -	YBL010C	ORF-T	ORF-T	-0.537614701	-0.363020921	0.492457598	-0.312574522	1.10670323
212556	216316	2 -	SLA1	ORF-T	ORF-T	-0.478409569	-0.191948899	-0.425609777	-0.047670103	-0.220978094
216316	217156	2 -	LDB7	ORF-T	ORF-T	-0.021732202	-0.187101627	-0.326848404	-0.320499349	-0.230473898
220028	226948	2 -	unknown	no_ovlp	SUTs	0.73205194	-0.888125305	-0.846007132	-0.317692282	-0.069295533
235260	235884	2 -	HTA2	ORF-T	ORF-T	-0.550765809	-1.487299193	-0.45076546	-0.101533686	0.03449765
236964	237532	2 -	ECM15	ORF-T	ORF-T	-0.081801442	-0.663426961	0.38928576	-0.022829732	-0.484034798
238860	241428	2 -	NTH2	ORF-T	ORF-T	-0.402897935	-0.325874841	0.875230514	1.179571371	0.485987522
241580	242596	2 -	RER2	ORF-T	ORF-T	0.408354131	-0.19774514	-0.886250074	-0.56899396	-0.260857246
244284	245620	2 -	GPI18	ORF-T	ORF-T	0.319447661	-0.347747933	-0.261966041	0.105794009	0.15817202
249316	250948	2 -	DSF2	ORF-T	ORF-T	-0.200499642	-0.318353385	-0.405969464	-0.288540773	-0.205573708
254532	255196	2 -	FLR1	ORF-T	ORF-T	1.224670345	-0.313136328	-0.828330584	-0.380798686	-0.099449139
255196	255716	2 -	HHF1	ORF-T	ORF-T	-0.29631501	-1.21665382	-0.32099266	-0.339486912	-0.106805189
256996	258028	2 -	IPP1	ORF-T	ORF-T	-0.17134456	0.077106649	-0.159142312	-0.042742647	0.17423167
266644	267316	2 -	GRX6	ORF-T	ORF-T	0.640377756	-0.018151845	0.278654425	0.050553664	0.241472226
267580	269588	2 -	MNN2	ORF-T	ORF-T	-0.319769215	-0.45982957	-0.452079461	0.019641855	0.155299674
269588	270372	2 -	unknown	no_ovlp	SUTs	0.014174969	0.261763543	0.220040725	0.11358481	0.534581803
270836	273756	2 -	KAP104	ORF-T	ORF-T	0.328401844	-0.300636043	-0.606332515	-0.132307557	-0.292987134
278572	280908	2 -	CUT445	CUTs	CUTs	1.880658206	0.260755578	-1.253409913	-0.709676914	-0.579354409
284300	288068	2 -	CHS3	ORF-T	ORF-T	-0.433930105	-0.133956182	-0.438437747	0.016822676	0.100162758
290452	291932	2 -	OLA1	ORF-T	ORF-T	-0.096094237	-0.862110832	-0.604976049	-0.242507434	-0.223339323
292764	294084	2 -	ETR1, YBR02	other	other	-0.242659857	-0.218133422	0.897763011	0.225994755	0.495490562
294260	296100	2 -	YBR027C, YB	other	other	-0.004600327	-0.638972628	-1.027017537	-0.565251017	-0.480284
296284	297820	2 -	CDS1	ORF-T	ORF-T	-0.313361332	-0.55002538	-0.765635052	-0.401750446	-0.038822839
304780	306020	2 -	HMT1	ORF-T	ORF-T	0.499912441	-0.248224873	-1.197597435	-0.383446832	0.111794701
306156	307148	2 -	PDX3	ORF-T	ORF-T	-0.30231164	-0.445493232	-0.102739355	-0.085840125	-0.354117779
307348	308076	2 -	unknown	no_ovlp	SUTs	0.928959507	0.708463486	0.013854003	-0.783374292	-0.813498136
308900	310364	2 -	CSG2	ORF-T	ORF-T	-0.52611486	-0.650374109	0.088053566	0.257639777	0.124344083
310580	311460	2 -	SCO1	ORF-T	ORF-T	-0.97206361	-0.571987436	0.586642608	0.127095747	0.469266514
320460	321652	2 -	CST26	ORF-T	ORF-T	-0.077352029	-0.195756787	-0.737564486	-0.327434112	0.001044451
321740	324068	2 -	QDR3	ORF-T	ORF-T	-0.896614486	-1.08949392	-0.525874789	-0.281146292	0.006117157
324276	326084	2 -	TCM62	ORF-T	ORF-T	-0.160573356	-0.303097215	0.248100061	-0.572002838	-0.319657179
326084	328148	2 -	unknown	no_ovlp2	SUTs	-0.24557755	0.23352056	0.969001207	-0.00913468	0.118840504
328148	330220	2 -	GIP1	ORF-T	ORF-T	-0.062245515	0.592838608	0.459921052	-0.492465574	0.515899882
330220	331540	2 -	ZTA1	ORF-T	ORF-T	-0.122009298	0.191631961	1.163323386	0.27971235	0.762883453
334188	336948	2 -	REB1	ORF-T	ORF-T	-0.016812728	-0.554403442	-0.551641201	-0.260196695	-0.210604095
337148	338668	2 -	REG2	ORF-T	ORF-T	0.15670257	0.650964963	1.824761915	1.793473602	-0.712113945
338324	338668	2 -	unknown	no_ovlp2	SUTs	0.436962992	-0.103085096	0.605226268	0.403630294	0.140111398
338668	339412	2 -	RFS1	ORF-T	ORF-T	-0.287627896	-0.355987093	0.580477127	0.425537419	0.366347068
339596	340772	2 -	YBR053C	ORF-T	ORF-T	-0.251060765	-0.286752844	0.520787891	-0.128470709	0.124721549
340892	341492	2 -	CUT449	CUTs	CUTs	2.996939977	0.175782172	-0.814535967	-0.230563064	-1.122403636
344500	347308	2 -	PRP6	ORF-T	ORF-T	-0.285132808	-0.869998536	-0.592648169	-0.17089133	-0.530962644
349420	350724	2 -	unknown	no_ovlp	SUTs	0.937237542	-0.107599181	0.15402699	0.530166288	-0.216009142
352084	353380	2 -	MUM2	ORF-T	ORF-T	0.03161881	-0.4607072	-0.154771512	0.142866428	-0.262307073
353524	356036	2 -	UBP14	ORF-T	ORF-T	0.016136886	-0.295222597	0.099360414	0.118432228	-0.347340547
356156	356620	2 -	TSC3	ORF-T	ORF-T	-0.075245969	-1.143590113	-0.26556341	-0.449441169	-0.028946077
356780	360316	2 -	AKL1	ORF-T	ORF-T	-0.242686245	-0.282811541	-0.166070611	-0.130204348	-0.496818506
360604	362588	2 -	ORC2	ORF-T	ORF-T	-0.081969713	-0.72441812	-0.322010571	-0.443604012	0.127168433
364668	365732	2 -	TRM7	ORF-T	ORF-T	0.194099051	-0.558078263	-0.709000257	-0.229457512	-0.297755942
365876	366515	2 -	YBR062C	ORF-T	ORF-T	0.188878301	-0.313659347	0.470171052	0.282523725	0.318317528
366859	369707	2 -	YBR063C, EC	other	other	0.307403023	-0.101764769	-0.072740508	-0.023699865	0.065571706
369987	371123	2 -	NRG2	ORF-T	ORF-T	-0.176725595	-0.84064974	0.354115558	0.49190044	-0.286999109
370763	371123	2 -	unknown	no_ovlp2	CUTs	1.073565012	-0.103382096	0.152743436	-0.051154685	-0.098405782
371971	372787	2 -	TIP1	ORF-T	ORF-T	-0.549428341	-1.50095942	0.061441142	0.312098081	0.146775979
373651	375747	2 -	BAP2	ORF-T	ORF-T	-0.374857289	-1.036734951	0.031275628	0.191965641	-0.030857321
376459	378507	2 -	TAT1	ORF-T	ORF-T	-0.508308671	-1.02534985	-0.497154699	-0.152370308	0.160934599
379171	379931	2 -	ALG14	ORF-T	ORF-T	-0.259646905	-0.115947811	-0.08890667	-0.216680112	0.957055428
382507	382955	2 -	unknown	no_ovlp	CUTs	1.972737062	-8.58E-05	0.052519004	0.439767671	0.767816881
390995	391523	2 -	YBR076C-A	other	other	2.01639092	-0.558414172	-0.195779461	-0.311426934	-0.461558252
391523	392347	2 -	YBR076C-A, !	other	other	-0.131692242	-0.69337315	0.404313277	-0.522365748	0.291396515
392347	392723	2 -	unknown	no_ovlp	SUTs	-0.119881084	0.001754576	-0.203306	-0.054032461	-0.127154452
395275	398299	2 -	RPG1	ORF-T	ORF-T	0.001073549	-0.457383347	-0.839622302	-0.16743886	-0.436835974
398515	400915	2 -	SEC18	ORF-T	ORF-T	0.14349029	-0.172758733	-0.290153691	-0.048348117	-0.568897109
401147	405339	2 -	SPT7	ORF-T	ORF-T	-0.203093634	-0.509776838	-0.183659461	-0.099707547	0.014079506
406451	407019	2 -	UBC4	ORF-T	ORF-T	-0.028841833	-0.328281105	0.028159713	-0.106556342	-0.080178251
407819	408443	2 -	CUT453	CUTs	CUTs	3.004857641	0.068171245	-0.287938585	-0.048543471	-1.832428826
414747	415266	2 -	RPL19A	ORF-T	ORF-T	0.510032748	-0.832549681	-0.928001473	-1.327676158	-0.014663421
418523	419211	2 -	YBR085C-A	ORF-T	ORF-T	0.582090221	-0.341554306	0.811914287	0.162102804	0.040122232
419211	419611	2 -	unknown	no_ovlp	CUTs	1.267964383	0.033428942	-0.779736933	-0.57384202	-1.157488061
420107	423243	2 -	IST2	ORF-T	ORF-T	-0.46093975	-0.372665378	-0.205336338	-0.113112819	0.412914337
424987	425819	2 -	POL30	ORF-T	ORF-T	-0.457350791	-0.529984835	-0.456074553	0.112140272	0.691218658
426498	426907	2 -	NHP6B, YBR(	ORF-T	ORF-T	0.427329577	-0.58055074	0.093186113	-0.216877109	-0.098758868
427075	427515	2 -	YBR090C, MI	other	other	0.042842344	0.007126868	0.1530353	-0.292551954	0.407271294
427651	429123	2 -	PHO3	ORF-T	ORF-T	-0.495038345	-0.484928056	-0.760452703	0.294869193	0.869172492
429123	429459	2 -	unknown	no_ovlp2	SUTs	0.020541572	0.302631686	-0.130459273	0.097633645	0.139447628
429459	431851	2 -	PHO5	ORF-T	ORF-T	-1.97967041	3.786798063	4.163741709	4.203723904	5.387661686
430987	431851	2 -	unknown	no_ovlp2	SUTs	-0.345983614	-0.573396848	0.436416261	0.055775078	0.574079225
434355	435715	2 -	RXT2	ORF-T	ORF-T	-0.260324841	-0.468573949	-0.047609179	-0.166540166	-0.086585792
443747	444707	2 -	FES1	ORF-T	ORF-T	0.724448068	-0.488429456	-0.120364178	0.088395307	-0.510097551

444707	447395	2 -	EXO84	ORF-T	ORF-T	0.081783007	-0.393162189	-0.365869613	-0.180710505	-0.157902239
448899	449331	2 -	unknown	no_ovlp	CUTs	3.888656871	0.213991948	-1.215892099	-0.395593458	0.520649521
449331	450691	2 -	unknown	no_ovlp	SUTs	-0.068189024	0.157138501	-0.016316588	-0.100629953	0.093396104
450835	452019	2 -	VID24	ORF-T	ORF-T	0.242484712	-0.592087642	0.545786691	0.230344149	0.306599271
453131	453363	2 -	unknown	no_ovlp2	CUTs	2.394751447	0.726410075	-1.303889452	-0.751839054	-0.213266933
453363	454571	2 -	IML3	ORF-T	ORF-T	-0.010618533	-0.34228123	0.315713728	-0.435192288	0.338723126
454571	457628	2 -	unknown	no_ovlp	SUTs	0.288446401	0.352499806	-0.355780357	-0.1426827	0.130675974
457716	458412	2 -	CMD1	ORF-T	ORF-T	-0.019999048	-0.738530484	-0.169808536	-0.148465295	0.004453954
461108	461852	2 -	YSA1	ORF-T	ORF-T	-0.366351391	-0.544033291	0.433086563	0.482375556	0.265652136
462700	465964	2 -	CYC8	ORF-T	ORF-T	-0.466949515	-0.640187782	-0.549151401	0.07036525	-0.538947817
465964	466636	2 -	unknown	no_ovlp	SUTs	-0.135473936	0.34996411	0.416065585	-0.294716374	0.025129705
469644	473996	2 -	LYS2	ORF-T	ORF-T	0.21432188	-0.189666795	-0.875119911	0.186394097	-0.5952409
473996	474436	2 -	unknown	no_ovlp2	SUTs	0.320627851	0.242596709	0.881624348	-0.375473736	-0.219440252
474436	477028	2 -	YBR116C, TK	ORF-T	ORF-T	-1.01873415	-0.039879677	3.8481902	2.892970237	2.530709649
476428	477028	2 -	unknown	no_ovlp2	SUTs	-0.011790797	0.330766508	0.801363077	0.323469749	0.01263712
477028	477340	2 -	unknown	no_ovlp	SUTs	0.999004684	-0.119651936	0.077788968	-0.332200076	-0.303344727
480372	480980	2 -	CBP6	ORF-T	ORF-T	-0.938400202	-1.35066433	0.064056259	-0.0849345	-0.281501365
481204	483476	2 -	GRS1, YBR12	ORF-T	ORF-T	-0.06847316	-0.487202138	-0.74577929	-0.270709041	-0.383758961
483772	484524	2 -	MRPL36	ORF-T	ORF-T	-0.718547825	-0.269344332	0.656645918	0.155579354	0.68726086
484748	486732	2 -	TFC1	ORF-T	ORF-T	-0.211821528	-0.787251675	-0.059603527	-0.19106081	-0.15867271
487012	488556	2 -	PTC4	ORF-T	ORF-T	0.054677544	-0.431796773	0.229313304	-0.165873955	0.513641639
488772	490484	2 -	TPS1	ORF-T	ORF-T	-0.41513795	-0.678348261	0.469597636	0.243638842	-0.096762453
491100	492876	2 -	VMA2	ORF-T	ORF-T	-0.267937072	-0.202459095	-0.388506358	-0.112256651	-0.248497574
492876	494276	2 -	ATG14	ORF-T	ORF-T	-0.906448635	-0.308208293	1.236226478	0.221969633	0.914774694
494276	495372	2 -	OPY1	ORF-T	ORF-T	-0.525799147	-0.777647284	0.112856711	0.401217283	0.19862474
495484	496924	2 -	SHE3	ORF-T	ORF-T	-0.109836518	0.053581659	-0.327305804	-0.334876969	-0.087471508
499348	501388	2 -	AGP2	ORF-T	ORF-T	-0.797072723	-0.013690423	1.363990893	1.485571828	1.149832246
501388	501660	2 -	unknown	no_ovlp2	SUTs	-0.505888793	-0.459556591	0.846464148	0.679059605	-0.106858435
501660	504428	2 -	HSL7	ORF-T	ORF-T	-0.055114027	-0.25676033	-0.433736227	-0.420030558	-0.060633043
513604	515388	2 -	YBR138C	ORF-T	ORF-T	-0.063145046	0.180312184	0.851835259	0.109631797	0.681621843
523356	526908	2 -	IRA1	ORF-T	ORF-T	-0.204686612	-0.371180707	0.029925518	0.033821112	0.272271397
526908	528044	2 -	YBR141C	ORF-T	ORF-T	0.360938239	0.056996114	-0.500992845	-0.222770674	0.730334346
530644	532236	2 -	SUP45	ORF-T	ORF-T	-0.150350768	-0.679871081	-0.584696039	0.03610596	-0.288276947
532988	533412	2 -	unknown	no_ovlp	CUTs	3.810619931	0.344114234	-0.71410162	-0.104813091	-1.602667292
535716	536252	2 -	CUT454	CUTs	CUTs	2.496504092	0.366570315	-0.778561685	0.26600732	-0.563772602
539332	539692	2 -	CUT455	CUTs	CUTs	2.3048066	0.176933611	0.705417572	0.340317645	-0.752914451
541180	544508	2 -	TBS1	ORF-T	ORF-T	-0.178492634	-0.680746678	-0.210179593	-0.058236425	-0.138500721
546492	547276	2 -	unknown	no_ovlp	CUTs	1.779205572	0.288761094	-1.024748259	-0.362620626	0.42676965
548268	549108	2 -	RPB5	ORF-T	ORF-T	0.28263959	-1.062576675	-0.710710622	-0.339249866	-0.332392462
549108	549532	2 -	CUT456	CUTs	CUTs	2.977382421	0.206302052	-0.152966197	-0.335977039	0.098417587
550932	553244	2 -	SLI15	ORF-T	ORF-T	-0.125615574	-0.52163628	0.436739008	-0.120203923	0.94309735
555116	555668	2 -	ICS2	ORF-T	ORF-T	-0.357935266	-0.717150097	0.245970921	-0.657994361	-1.303682606
558140	558476	2 -	unknown	no_ovlp	CUTs	1.653362403	0.588821698	0.841849201	-0.440463701	-0.292771859
562868	564788	2 -	TOS1	ORF-T	ORF-T	-0.558380132	-0.567379957	-0.392655904	-0.027715636	0.254512893
567756	568540	2 -	ARL1	ORF-T	ORF-T	0.185549204	-0.248651843	-0.363817164	-0.207909532	0.016304456
569732	571236	2 -	TYR1	ORF-T	ORF-T	0.212489375	-0.606532656	-0.448490953	-0.231352328	-0.491050384
571932	572252	2 -	POP7	ORF-T	ORF-T	0.211342595	-1.070489741	0.206966649	-0.334524152	-0.895479282
573764	578124	2 -	SSE2	ORF-T	ORF-T	0.046645055	0.068709964	1.662338597	1.118277254	0.315834744
576060	578124	2 -	unknown	no_ovlp2	SUTs	0.012988456	-0.389432033	-0.118830909	-0.226012831	-0.069092589
579044	581484	2 -	SMY2	ORF-T	ORF-T	0.156721126	-0.156093813	-0.099597478	-0.238611862	-0.138580956
581580	582196	2 -	UMP1	ORF-T	ORF-T	0.14134346	-0.131032252	0.532320386	-0.143553202	-0.0280665
584668	586188	2 -	EHT1	ORF-T	ORF-T	0.079713092	-0.541270352	-0.662349044	-0.184471184	-0.470442899
586500	589172	2 -	FZO1	ORF-T	ORF-T	-0.509461179	-0.459882761	0.122026617	0.125218938	0.312581439
592412	592772	2 -	RPS6B	ORF-T	ORF-T	0.52374517	-0.974618898	-0.934595543	-0.825467678	0.469475852
593196	595836	2 -	SMP1	ORF-T	ORF-T	0.265231216	-0.395169937	-0.651870792	0.351098895	-1.987801401
595020	595836	2 -	unknown	no_ovlp2	CUTs	2.028103489	-0.632016358	-0.539226879	-0.254571053	-0.904001317
598940	599988	2 -	MBA1	ORF-T	ORF-T	-0.891189838	-0.746790906	0.237820348	0.079947841	0.789337023
601388	602204	2 -	SUT449	SUTs	SUTs	1.058965379	-1.210249837	-0.962618559	-0.674989118	0.02994026
603524	604180	2 -	NTC20	ORF-T	ORF-T	-0.464534092	-0.282310573	0.604985422	-0.311580721	0.851495721
608692	609780	2 -	MED8	ORF-T	ORF-T	0.025456111	-0.219829172	-0.062142741	-0.221953508	-0.623203993
610492	611876	2 -	MSI1	ORF-T	ORF-T	0.119094449	-0.277701273	-0.797837876	-0.2259913	0.15941646
612060	613972	2 -	PGI1	ORF-T	ORF-T	-0.262219403	0.08283599	-0.168524641	-0.004901493	-0.159026011
613972	614764	2 -	unknown	no_ovlp2	SUTs	0.83941653	-0.145738782	-0.806013043	-0.400477996	0.226030512
614764	615876	2 -	YBR197C	ORF-T	ORF-T	0.302392454	0.013012398	-0.411371512	-0.464694609	-0.008120681
616036	618596	2 -	TAF5	ORF-T	ORF-T	-0.272811085	-0.396109369	-0.502540074	0.001574214	-0.146098001
624252	624716	2 -	YBR201C-A	ORF-T	ORF-T	0.440233475	0.501847211	0.910325138	1.069057344	-0.935621943
624716	625460	2 -	unknown	no_ovlp	SUTs	0.296440007	-0.099565721	0.174883896	0.026561324	-0.554513148
628940	629700	2 -	unknown	no_ovlp	CUTs	1.82182957	1.128465434	-0.614690591	-0.923066885	-0.047137736
632084	633412	2 -	YBR204C	ORF-T	ORF-T	-0.434426073	-0.247411183	0.593013859	-0.219240654	0.61099668
642364	642636	2 -	DUR1%2C2	ORF-T	ORF-T	0.25961073	-1.003443515	-0.554847441	-0.169282803	-0.900092904
646012	647204	2 -	AME1	ORF-T	ORF-T	-0.413658662	-0.948541578	0.245260697	0.352222294	0.19676563
650332	650988	2 -	unknown	no_ovlp	CUTs	1.360450233	0.322958312	0.993518348	0.831464326	0.662199759
655188	657620	2 -	YBP1	ORF-T	ORF-T	0.089990325	-0.434307934	-0.465083712	-0.226053935	-0.538749469
658548	662468	2 -	PYC2	ORF-T	ORF-T	0.093015775	-0.489734652	-0.782201211	0.076408286	-0.93733564
662828	664740	2 -	YBR219C, YB	ORF-T	ORF-T	-0.584846843	-0.700676916	-0.642540443	-0.19399533	-0.148964337
664916	666476	2 -	PDB1	ORF-T	ORF-T	-0.388464453	-0.390062854	-0.274667771	-0.115313456	-0.009423051
666612	668380	2 -	PCS60	ORF-T	ORF-T	-0.015995248	1.036271433	0.796191446	0.319213768	1.361152315
668380	670316	2 -	TDP1	ORF-T	ORF-T	-0.349819124	-0.691897568	-0.21531172	-0.096175113	-0.599873068
670316	671708	2 -	unknown	no_ovlp	SUTs	-0.31725541	0.408061866	0.090068248	0.16205249	0.320787952
673476	675188	2 -	YBR226C, M(	other	other	0.260963294	-0.254167231	-0.141139028	-0.237205693	-0.32180002
676228	679236	2 -	ROT2	ORF-T	ORF-T	-0.379579541	-0.124965802	-0.009780973	-0.182643375	0.633163181
679412	680076	2 -	OM14	ORF-T	ORF-T	-0.319838034	-0.578634272	1.356237158	0.587658787	0.838555969

680076	680692	2	-	unknown	no_ovlp	CUTs	1.008719643	-0.19494924	0.401862173	0.49451637	-0.032966973
680836	681860	2	-	LSR1	other	other	-0.12715094	0.054612723	-0.014026419	-0.081219008	0.054093684
681860	683116	2	-	SWC5	ORF-T	ORF-T	-0.149623581	-0.147131051	-0.145230961	-0.314575325	0.340835454
685380	686620	2	-	ARC40	ORF-T	ORF-T	-0.432289171	-0.126766922	-0.068281447	-0.041775618	0.421573876
690260	691732	2	-	ABD1	ORF-T	ORF-T	-0.068918189	-0.078968387	-0.071986473	-0.073053561	0.089140284
694588	697428	2	-	YBR238C	ORF-T	ORF-T	0.585142411	-0.51241125	-0.452578224	0.009377635	-0.77382189
698420	700012	2	-	YBR239C	ORF-T	ORF-T	0.261015288	-0.314671327	-0.368395389	-0.072554197	-0.410988154
700012	700396	2	-	unknown	no_ovlp2	SUTs	0.005583333	0.287141451	0.226554349	0.03179928	-0.359429585
700396	702532	2	-	THI2	ORF-T	ORF-T	-0.440080263	0.058420215	0.655114173	0.820660675	-0.557515808
703100	704108	2	-	YBR241C	ORF-T	ORF-T	-0.739094129	-0.129434499	0.818559548	0.451671352	0.611350334
705252	706828	2	-	ALG7	ORF-T	ORF-T	-0.767578856	-0.475911943	-0.213739979	-0.058320105	0.775684452
708028	711404	2	-	ISW1	ORF-T	ORF-T	-0.199530611	-0.353160942	-0.468227154	-0.153759954	0.135328943
712860	714492	2	-	ENP1	ORF-T	ORF-T	0.260379526	-0.251640697	-0.923112121	-0.17726478	0.296481959
714732	716524	2	-	HIS7	ORF-T	ORF-T	-0.096890776	-0.352990009	-0.866347988	-0.321415532	-0.449944164
716772	718124	2	-	ARO4	ORF-T	ORF-T	0.057266452	-0.361751852	-0.563050672	-0.01370382	-0.754669503
718124	718348	2	-	unknown	no_ovlp	SUTs	-0.754944823	-0.549977329	0.856459468	0.151959812	1.301480003
720716	721172	2	-	CUT458	CUTs	CUTs	2.29708017	0.158942631	0.298741474	0.203163785	0.019922561
723596	724268	2	-	TRS20	ORF-T	ORF-T	0.496622424	-0.400365342	-0.280619978	-0.612322734	0.080093656
726516	726908	2	-	YBR255C-A	ORF-T	ORF-T	0.577975189	-0.422781231	-0.052034048	0.335215188	0.171909049
727324	728132	2	-	RIB5	ORF-T	ORF-T	0.109961197	-0.903819745	0.05069501	-0.097296367	-0.343422207
728132	728636	2	-	unknown	no_ovlp	SUTs	0.461494325	-0.312021315	0.507133283	-0.41597953	1.051827944
729364	730188	2	-	SHG1	ORF-T	ORF-T	-0.274865868	-0.894227816	-0.175235253	-0.162726653	-0.074743262
732820	734132	2	-	RGD1	ORF-T	ORF-T	0.103764512	-0.027982783	-0.223714263	-0.163359603	0.039811944
734812	735540	2	-	YBR261C	ORF-T	ORF-T	-0.221124913	-0.288378568	-0.624138572	-0.287517378	-0.654053311
735644	736076	2	-	FMP51	ORF-T	ORF-T	-0.709772754	-1.062823492	0.049464849	-0.056839942	0.058093393
737772	738372	2	-	YPT10	ORF-T	ORF-T	-0.625226975	-0.968875194	0.260876694	-0.047608266	0.412019676
738372	739660	2	-	unknown	no_ovlp	CUTs	2.003912651	0.131114242	-1.657629621	-0.518500429	0.114885328
742004	742580	2	-	FMP21	ORF-T	ORF-T	-0.210504626	-0.368759293	0.606436449	0.019395787	-0.205353954
742732	744556	2	-	BIT2	ORF-T	ORF-T	0.256314427	-0.223918408	0.01815906	-0.352015138	0.262036674
746180	747820	2	-	HSM3	ORF-T	ORF-T	0.148319505	-0.247566672	0.182581139	-0.099274573	-0.346119806
748004	749388	2	-	UBX7	ORF-T	ORF-T	0.174781881	-0.164573501	0.045617388	0.035184565	0.042924445
753900	757212	2	-	RIF1	ORF-T	ORF-T	-0.058680536	-0.327857538	-0.412276159	-0.126768211	0.097344137
757556	760132	2	-	PPS1	ORF-T	ORF-T	-0.103917742	-0.070628254	-0.21738585	-0.303860996	-0.136964056
760132	760900	2	-	unknown	no_ovlp	SUTs	-0.006441236	-0.878410475	-0.065280941	-0.241287982	1.087428402
762652	764708	2	-	SAF1	ORF-T	ORF-T	0.019661106	-0.032855508	0.659859666	0.465494121	-0.096303847
764868	767628	2	-	DUG2	ORF-T	ORF-T	0.752068752	0.393763862	-0.200405091	-0.330682243	0.178887116
769300	770532	2	-	SSH1	ORF-T	ORF-T	-0.414427337	-0.495139269	-0.509717674	0.005645602	-0.255629691
773684	774516	2	-	unknown	no_ovlp	CUTs	1.002781121	-0.535293293	0.973087385	0.446891184	0.284974881
775740	776244	2	-	unknown	no_ovlp	CUTs	1.591757378	-0.093059143	-0.785853976	-0.504778267	0.749480786
777884	779500	2	-	APM3	ORF-T	ORF-T	-0.082165425	-0.391272006	-0.401340687	-0.116660411	-0.075794545
779500	780468	2	-	unknown	no_ovlp	SUTs	-0.600056145	0.402909606	0.067877089	0.384350369	0.257219483
783604	784580	2	-	CTP1	ORF-T	ORF-T	0.572382317	-0.38668986	-0.352123938	0.804837773	0.148381937
784876	786388	2	-	YBR292C	other	other	-0.149675955	-0.257274995	0.298998412	-0.228228768	-0.810956488
785852	786388	2	-	unknown	no_ovlp2	SUTs	-0.749689145	-0.565250179	0.728183658	0.62101985	-0.610524924
786388	786868	2	-	SUT451	SUTs	SUTs	1.434392871	-1.200295845	-0.579081919	-0.332469667	0.014968446
789084	792524	2	-	SUT452	SUTs	SUTs	0.365174974	-0.2661944	0.283850773	-0.057676351	-1.667509589
802556	804924	2	-	MAL31	ORF-T	ORF-T	-0.04469697	-1.205887754	0.683115044	0.95931696	-0.563187914
24001	24945	2	+	SFT2	ORF-T	ORF-T	-0.761981244	-0.735333582	0.169262236	0.10438053	-0.035012948
27865	36729	2	+	unknown	no_ovlp	SUTs	0.168507685	0.400828419	0.154835488	-0.090065275	0.138070316
36729	38953	2	+	ATP1	ORF-T	ORF-T	-0.413625572	0.000511755	0.302935533	0.081265805	0.051479834
39137	40649	2	+	BNA4	ORF-T	ORF-T	1.238245129	1.201624292	0.377970739	-0.487152415	-1.510889483
40785	43265	2	+	BRN1	ORF-T	ORF-T	-0.305019661	-0.181612255	-0.114796479	-0.372277199	0.66539052
43265	44217	2	+	YBL095W	ORF-T	ORF-T	-0.334840956	-0.57802861	0.158093888	0.164697861	0.968627688
45609	45985	2	+	RPL32	ORF-T	ORF-T	1.139939056	-0.997550297	-1.620241453	-1.051409509	-0.068357531
48793	49473	2	+	MRP21	ORF-T	ORF-T	-1.135030599	-0.953847721	0.372684861	-0.049116313	0.252431169
49569	50985	2	+	AVT5	ORF-T	ORF-T	0.238217803	-0.125140626	-0.229068351	-0.13365817	-0.320807486
59265	63705	2	+	unknown	no_ovlp	SUTs	0.028322117	0.444733859	-0.112345908	0.07491662	0.122002894
63705	64793	2	+	BOI1	ORF-T	ORF-T	0.022228169	-0.508860563	-0.383754747	-0.017580161	-0.110138372
71561	73081	2	+	YBL081W	ORF-T	ORF-T	0.350957469	0.269774176	-0.265499466	-0.074259152	0.255750233
75217	77737	2	+	NUP170	ORF-T	ORF-T	0.168724278	-0.052615157	-0.395998239	0.00790723	0.137872466
84545	85081	2	+	unknown	no_ovlp	CUTs	2.869597388	0.809720492	-0.792768366	-0.007651399	0.790927084
88185	88473	2	+	unknown	no_ovlp	CUTs	3.986583457	-1.373060439	-0.226216311	-0.061783967	-0.063312734
89953	90249	2	+	KTI11	ORF-T	ORF-T	-0.15086329	-0.181019088	0.822785527	-0.040827201	0.950106493
90705	92169	2	+	AST1, YBL068	ORF-T	ORF-T	0.314458035	-0.004103698	0.351003623	-0.201244201	0.437221644
92369	93577	2	+	PRS4	ORF-T	ORF-T	0.250520903	-0.174117251	-0.134271676	-0.064059149	0.217325909
96354	96957	2	+	CUT006	CUTs	CUTs	0.792584027	0.343399994	1.044547233	0.796186508	1.071868736
101895	105295	2	+	KIP1	ORF-T	ORF-T	-0.067933421	-0.311801617	-0.183076797	-0.257082959	1.065925377
107751	110031	2	+	YEL1	ORF-T	ORF-T	0.169300831	-0.400714237	-0.174560671	-0.25463758	0.423308187
110711	111423	2	+	YBL059W	ORF-T	ORF-T	0.456504063	0.349507422	0.294680886	-0.613853457	-0.473748198
111423	112823	2	+	SHP1	ORF-T	ORF-T	-0.446981096	-0.351144679	0.247989595	0.151844251	0.188463816
113671	115567	2	+	PTC3	ORF-T	ORF-T	-0.301370836	-0.996523168	-0.508702407	0.023311035	-0.313284203
117495	119351	2	+	YBL054W, YB	other	other	0.58137567	-0.104855322	-0.161599006	0.063299296	0.621356869
125286	126191	2	+	SEC17	ORF-T	ORF-T	-0.331923566	-0.558134458	0.022548548	0.110009914	-0.253400069
126407	126647	2	+	unknown	no_ovlp	SUTs	-0.405524295	-0.028727945	1.049474863	0.370739994	2.407138887
126647	127503	2	+	MOH1, YBL0	other	other	-0.064945885	0.655859972	2.107850553	0.65792679	1.865082326
132439	133935	2	+	PSY4	ORF-T	ORF-T	-0.021176937	-0.488613432	0.101181426	0.085824828	0.673354391
136543	137631	2	+	ECM13	ORF-T	ORF-T	0.446186435	0.305470351	-0.026730508	-0.237259711	0.219690367
140975	141223	2	+	PRE7	ORF-T	ORF-T	0.218216235	-0.616273101	0.027332518	-0.384700411	-1.112949383
143127	143703	2	+	YBL039W-B	ORF-T	ORF-T	-0.469103582	-0.24284143	1.418773313	0.369859119	0.208661506
146127	146951	2	+	MRPL16	ORF-T	ORF-T	-0.369396129	-0.63877616	0.294360669	-0.096611155	0.061611369
147135	150423	2	+	APL3	ORF-T	ORF-T	-0.180496554	-0.383050653	-0.409270134	-0.148811126	-0.356760853
151439	153743	2	+	unknown	no_ovlp	SUTs	0.882854891	0.224129576	-0.41036227	-0.504838639	0.065078603

160103	161439	2 +	HEK2	ORF-T	ORF-T	-0.96474252	-0.634297772	-0.53321144	-0.160163075	0.494514872
161711	162727	2 +	SHE1	ORF-T	ORF-T	-0.1597372	-0.345011388	-0.309384313	-0.541829236	0.979320126
164495	164999	2 +	SUT006	SUTs	SUTs	1.836993045	-0.149803907	0.406045344	-0.938032512	0.290825787
166063	167335	2 +	YBL029W	ORF-T	ORF-T	-0.173973996	-0.201976139	-0.153809613	-0.161830045	0.633801588
168423	168855	2 +	RPL19B	ORF-T	ORF-T	0.655447585	-0.726421054	-1.079716196	-1.232380585	-0.081723611
170823	171175	2 +	LSM2	ORF-T	ORF-T	-0.171030534	-0.35539943	0.03808078	-0.169144302	0.321404419
171175	171423	2 +	unknown	no_ovlp2	SUTs	-0.056151287	-0.83982813	-0.387406144	0.123765231	-0.277736354
171423	172511	2 +	RRN10	ORF-T	ORF-T	0.653431248	0.024847952	-0.484384082	-0.457027881	-0.06436034
172207	172511	2 +	unknown	no_ovlp2	CUTs	1.389470928	-0.223033526	-0.751210349	-1.118051564	-1.024502079
172511	174791	2 +	NCL1	ORF-T	ORF-T	-0.088419785	-0.499781678	-0.887263511	0.132793516	0.249915369
180743	182327	2 +	unknown	no_ovlp	SUTs	0.321263803	0.411557259	0.149260352	-0.353921218	0.624382579
182327	184199	2 +	RFT1	ORF-T	ORF-T	-0.223078931	-0.362261428	-0.419150535	-0.005788972	0.29833085
184487	186031	2 +	APN2	ORF-T	ORF-T	-0.121275315	-0.260914394	0.146851279	0.1559683	-0.601038292
192023	193647	2 +	CUT007	CUTs	CUTs	0.229937073	-0.022192453	-0.287835637	0.176390883	-0.11490307
194039	195751	2 +	ACH1	ORF-T	ORF-T	-0.773188354	-0.788039948	0.658490602	0.428190281	0.777788923
202079	203448	2 +	FMT1	ORF-T	ORF-T	-1.147947543	-0.477899914	0.267690197	0.138331931	1.762294765
203448	205984	2 +	SCT1	ORF-T	ORF-T	-0.327405375	-0.586497472	-0.600097976	0.033983789	-0.160825212
207176	209336	2 +	ALK2	ORF-T	ORF-T	-0.2724572	0.064459601	0.116696735	-0.160004501	1.178864445
209640	212288	2 +	YBL008W-A,	other	other	-0.484932895	-0.390308594	-0.258587797	-0.184538394	0.516800306
216776	217568	2 +	YBL006W-A	other	other	0.206259539	-0.082361699	0.329061146	0.366800905	0.105462945
217256	217568	2 +	unknown	no_ovlp2	SUTs	-0.642867758	-1.077872662	-0.329555467	0.128172244	-0.562532309
217568	220408	2 +	PDR3, YBLW(	other	other	-0.146921061	-0.378561706	-0.125933003	-0.10470595	-0.210594152
221368	226952	2 +	unknown	no_ovlp	SUTs	-0.186363427	-0.532497773	0.065856268	-0.250997123	0.033707284
227560	235208	2 +	UTP20	ORF-T	ORF-T	0.175812872	-0.423218533	-0.547379082	0.029840255	0.10972062
236376	237176	2 +	HTB2	ORF-T	ORF-T	-0.334113985	-0.983676914	-0.84632812	-0.176823579	-0.238902951
238512	238976	2 +	SUT008	SUTs	SUTs	0.995536315	-0.348339001	-0.063349736	-0.32057817	-1.153139633
241752	242792	2 +	SUT009	SUTs	SUTs	0.705541069	-0.013829593	0.500863443	0.601635144	1.167486944
242792	244360	2 +	COQ1	ORF-T	ORF-T	-0.566569807	-0.678534403	0.243629181	0.162605846	0.114902216
245856	246744	2 +	RCR1	ORF-T	ORF-T	0.359245441	-0.064183081	-0.343938557	0.338123504	-0.005398752
246744	246976	2 +	UGA2	ORF-T	ORF-T	0.055500993	0.205124787	-0.325682463	-0.252709372	0.434463339
251624	252232	2 +	SUT010	SUTs	SUTs	0.80163838	-0.135172865	0.231110988	0.73168028	-0.435140046
254640	255024	2 +	SUT011	SUTs	SUTs	1.400389397	0.012287035	-0.168722015	-0.114927449	-0.114058131
256312	256896	2 +	HHT1	ORF-T	ORF-T	-0.393997363	-0.627084589	0.141075261	-0.097769611	0.148056242
263136	266184	2 +	unknown	no_ovlp	SUTs	-1.006951621	-0.736882149	0.641915995	-0.316263322	-0.597736874
270208	270904	2 +	YBR016W	ORF-T	ORF-T	-0.193198824	-0.304448855	0.274258031	0.455242881	0.311917851
276760	278688	2 +	SUT013	SUTs	SUTs	-1.023402001	-0.535001054	0.468032801	0.661692458	0.487942365
278688	281360	2 +	GAL1	ORF-T	ORF-T	-0.848968966	-0.174843572	0.044543241	0.210411093	0.191816783
281360	283520	2 +	FUR4	ORF-T	ORF-T	0.50023012	-0.42821121	-0.730211776	-0.281476098	-0.451101759
283696	284408	2 +	POA1	ORF-T	ORF-T	0.064136497	-0.235329621	-0.360737244	0.004834522	-0.299070298
289352	290568	2 +	SCO2	ORF-T	ORF-T	-0.938553441	-0.453487284	0.670587952	0.339986631	0.781700674
292416	293040	2 +	unknown	no_ovlp	CUTs	2.595372472	0.268541194	-0.432332464	0.005841341	-0.049588859
298208	300152	2 +	YBR030W	ORF-T	ORF-T	0.849098308	0.180649644	-0.650534789	-0.383006656	0.188517598
300152	301376	2 +	RPL4A	ORF-T	ORF-T	-0.820927407	-0.584488912	0.1125897	0.240131619	-0.116323417
306304	307016	2 +	CUT011	CUTs	CUTs	3.632815877	0.069899144	-1.020237445	-0.093853548	-0.08436607
307568	308480	2 +	TLC1	other	other	0.004796851	-0.868508337	-0.490541786	-0.383751923	-0.32734742
310592	310864	2 +	CUT013	CUTs	CUTs	1.588927174	0.586904489	-0.060726159	0.099315084	0.335534202
311856	314968	2 +	CHS2	ORF-T	ORF-T	-0.103037931	0.09477978	-0.320393379	-0.148436033	0.171469013
315504	316712	2 +	ATP3	ORF-T	ORF-T	-0.450213454	-0.210222758	0.397742987	0.115648581	0.008960307
318232	319128	2 +	FAT1	ORF-T	ORF-T	-0.267240793	-0.388148111	-0.247795257	-0.062444088	-0.5121117
324456	325336	2 +	CUT014	CUTs	CUTs	0.95904888	-1.368348317	-1.7046415	-0.21227034	0.050815548
326296	326776	2 +	CUT015	CUTs	CUTs	0.376000011	0.535049542	0.323965384	0.069628643	-0.643156269
331824	332336	2 +	FMP23	ORF-T	ORF-T	-0.128364381	0.109737566	1.317252704	-0.180462587	1.958082366
332872	333392	2 +	RPS11B	ORF-T	ORF-T	0.813279723	-0.330813181	-1.007127893	-0.383234768	-0.55685425
337208	337824	2 +	unknown	no_ovlp	CUTs	2.215751448	0.591310473	0.116023986	0.277339545	-1.050513417
339584	340552	2 +	CUT016	CUTs	CUTs	1.182963128	-0.753931705	0.110785419	0.821875975	0.480806919
341016	342784	2 +	CUT017	CUTs	CUTs	2.1142451	-0.147311738	-0.496670761	-0.639070216	-0.908266564
342784	344504	2 +	YRO2	ORF-T	ORF-T	0.419196829	-0.27220046	0.182867917	-0.083194422	-1.337630468
346856	347816	2 +	unknown	no_ovlp	CUTs	1.408434277	0.396535335	-0.751613548	-0.266666882	0.229708386
347816	349440	2 +	YBR056W	ORF-T	ORF-T	-0.262463594	-0.34510014	0.619105297	0.142055461	0.3188172
351240	351912	2 +	YBR056W-A	other	other	-0.025885118	-0.812687892	-0.439251748	0.222162173	-0.394090326
360632	362856	2 +	CUT018	CUTs	CUTs	0.874815659	0.409395496	-0.868918725	0.112350235	0.628108551
362856	364696	2 +	SUT014	SUTs	SUTs	0.705017547	-0.535766999	-0.482446299	-0.494492	-0.166167001
365896	366623	2 +	CUT019	CUTs	CUTs	2.785780261	0.074834844	-0.901880107	-0.362861912	-0.116834054
370015	370831	2 +	CUT020	CUTs	CUTs	1.933634218	0.344471554	0.283781855	0.12298896	-0.758041944
371471	372743	2 +	SUT016	SUTs	SUTs	0.173533565	-0.184484786	-0.654964743	-0.397615898	-1.118170643
376383	376831	2 +	CUT021	CUTs	CUTs	2.3575297	-0.976733918	-1.136156803	-0.342659468	-1.053034776
380351	381319	2 +	YBR071W	ORF-T	ORF-T	-0.173521073	-0.387406827	0.689204939	0.136092209	0.52336247
381319	381943	2 +	unknown	no_ovlp2	CUTs	2.353629017	0.442281251	-0.084538678	-0.008011227	0.551739932
381943	382839	2 +	HSP26	ORF-T	ORF-T	-0.925363085	-0.590051351	3.243108514	1.411036952	0.648436628
383143	386039	2 +	RDH54	ORF-T	ORF-T	-0.07717447	0.114123747	0.143927281	0.119628447	0.756564547
386279	389335	2 +	YBR074W	ORF-T	ORF-T	-0.189837677	-0.349878147	-0.467834798	-0.172596191	-0.277757791
390911	391879	2 +	unknown	no_ovlp	SUTs	-1.106687149	-0.310609178	1.688265973	0.805387454	-0.60889751
391879	392399	2 +	unknown	no_ovlp	CUTs	2.358262096	0.422367875	-0.098565917	-0.372110197	0.079814956
392951	393175	2 +	ECM33	ORF-T	ORF-T	-0.46186452	-0.286419584	-0.355488373	-0.260898788	0.06166083
395295	395951	2 +	unknown	no_ovlp	SUTs	-0.127051595	0.148758379	0.257814271	-0.064385007	0.144097862
406055	407583	2 +	unknown	no_ovlp	SUTs	0.306886321	0.256011102	0.192840123	0.453184626	0.035154262
407583	408023	2 +	TEC1	ORF-T	ORF-T	1.704868084	-0.770194915	0.605668999	0.355427559	-0.823701446
410999	414135	2 +	MIS1	ORF-T	ORF-T	0.282796661	-0.300227035	-0.750525168	-0.020200973	0.105641204
419559	420895	2 +	unknown	no_ovlp	CUTs	1.387347612	-0.12286913	-0.172420278	0.161085657	-0.91330059
423567	424887	2 +	RFC5	ORF-T	ORF-T	0.690238834	0.081364595	-0.022933215	-0.152394711	0.306021017
427175	429575	2 +	unknown	no_ovlp	CUTs	1.144742385	0.440236643	-0.199845375	-0.290925312	-0.092266597
429575	431799	2 +	CUT025	CUTs	CUTs	1.586513116	-0.609209086	-0.940206572	-0.179734549	-0.287111597

431983	434399	2 +	PBY1	ORF-T	ORF-T	-0.017531859	-0.288803506	-0.242713213	-0.058201261	0.252984359
435935	436783	2 +	YBR096W	ORF-T	ORF-T	0.050835246	-0.495217847	-0.025761216	-0.181136725	-0.176313582
436911	441383	2 +	VPS15	ORF-T	ORF-T	-0.129438546	-0.082821834	0.08242747	-0.149611079	0.354371483
441383	443679	2 +	MMS4	ORF-T	ORF-T	-0.523351227	-0.342591712	0.41914822	-0.242869848	1.135734805
447583	449375	2 +	SIF2	ORF-T	ORF-T	-0.219075987	-0.215557598	0.206691597	0.094666245	0.479453535
449591	450759	2 +	YMC2	ORF-T	ORF-T	0.741889861	-0.597801951	-1.71993309	-0.634073223	-0.178517928
452591	453367	2 +	PHO88	ORF-T	ORF-T	-0.616398259	-0.43675365	-0.450336422	-0.092518854	0.07264703
453367	454807	2 +	unknown	no_ovlp2	SUTs	-0.001660978	-0.455636456	-0.865418356	-0.108703725	0.367567668
454807	457287	2 +	YBR108W	ORF-T	ORF-T	-0.117285295	0.086582551	0.267950284	-0.130794092	0.161167883
458688	460320	2 +	YBR109W-A,	other	other	-0.170629988	-0.3549172	-0.220470299	-0.325166444	-0.373422669
462120	462424	2 +	SUS1	ORF-T	ORF-T	0.605368679	-0.448385109	-0.18057812	-0.271523847	-0.431701421
462503	462784	2 +	unknown	no_ovlp	SUTs	0.335484441	-0.235992008	0.090678383	0.082395133	-0.210722405
467160	467728	2 +	RAD16	ORF-T	ORF-T	0.841990301	-0.005357877	-0.370497555	0.049422438	-0.200802699
474328	476016	2 +	SUT018	SUTs	SUTs	1.287190698	-0.374511225	-0.972379484	-0.303984744	-0.1276043
477560	479160	2 +	TEF2	ORF-T	ORF-T	-0.296064714	-0.882208995	0.117009953	0.144690865	-0.589640304
479160	480464	2 +	MUD1	ORF-T	ORF-T	-0.02753983	-0.75772635	-0.129867102	-0.031810605	-0.068582874
483776	484064	2 +	CUT026	CUTs	CUTs	3.16270854	-0.409547806	-1.772444607	-0.23345266	-0.181128372
490824	491240	2 +	YBR126W-A,	other	other	-0.167455424	-0.430559369	0.071392725	0.091318553	0.051365085
493072	493824	2 +	CUT027	CUTs	CUTs	2.030153834	-0.357703439	-0.759867718	0.0902443	0.297000084
497176	499408	2 +	CCZ1	ORF-T	ORF-T	0.078038778	-0.210987867	-0.81442622	-0.663018126	-0.233243922
504760	505456	2 +	CKS1	ORF-T	ORF-T	-0.298833293	-0.500825173	-0.246201597	-0.108916484	0.259309178
505640	509128	2 +	MEC1	ORF-T	ORF-T	0.215135002	-0.031589073	0.073398653	0.067444078	0.710504315
513040	513744	2 +	YBR137W	ORF-T	ORF-T	-0.765053507	-1.256273348	0.130077202	0.185201333	-1.017499321
515672	517304	2 +	YBR139W	ORF-T	ORF-T	-0.5237836	0.114118161	0.710391765	0.320521571	0.769586329
517448	520280	2 +	unknown	no_ovlp	SUTs	0.485197298	0.426355849	0.358443893	-0.366497106	0.324599589
527160	527768	2 +	YBR141W-A	other	other	1.08855802	-0.537821483	-0.57780345	-0.068861545	0.625345506
528248	530832	2 +	MAK5	ORF-T	ORF-T	-0.010995851	-0.613690911	-0.610494059	0.007130289	0.243922962
532544	533728	2 +	SUT019	SUTs	SUTs	0.529011376	0.171283635	-0.324499881	-0.408244079	-0.310596579
533728	534896	2 +	ADH5	ORF-T	ORF-T	-0.30387147	-1.472135947	-1.233575259	0.085938781	-2.857427826
534984	535224	2 +	unknown	no_ovlp	SUTs	-0.292596714	-0.06386832	-0.026424533	0.330239706	0.130923934
535224	536288	2 +	MRPS9	ORF-T	ORF-T	-0.779901951	-0.624782766	-0.018790548	-0.09501598	-0.260838761
536464	537440	2 +	YBR147W, YS	other	other	-0.821527371	-0.584137885	1.222126011	1.150837462	-1.768243305
537440	537928	2 +	unknown	no_ovlp2	SUTs	-0.56793648	0.182163873	0.396487471	0.56300799	-0.938961421
537928	539928	2 +	YSW1	ORF-T	ORF-T	0.002553663	0.825684849	0.421474228	-0.236487014	-0.687867839
539632	539928	2 +	unknown	no_ovlp2	SUTs	-1.443944378	-0.590914487	0.682194325	0.40864831	-0.013202253
539928	541256	2 +	ARA1	ORF-T	ORF-T	0.059839105	-0.39192531	0.617825635	0.137909758	0.191149678
544888	546096	2 +	APD1	ORF-T	ORF-T	-0.688127686	-0.760569956	0.456518171	0.297370915	0.569849535
546096	546312	2 +	unknown	no_ovlp2	SUTs	-0.340907692	-1.198127195	-0.232754165	-0.56325422	-0.411369163
546312	547408	2 +	SPP381, RIB7	ORF-T	ORF-T	0.234687045	-0.277566044	-0.41151139	-0.224444863	-0.099193673
549712	551000	2 +	CNS1	ORF-T	ORF-T	0.285794665	-0.013319507	-0.953650136	-0.607007767	0.325338361
553496	555360	2 +	SUT020	SUTs	SUTs	0.29590519	0.269549638	0.039420818	0.120195824	0.405030135
555360	556264	2 +	SUT020	SUTs	SUTs	0.27307806	0.11636536	0.591901404	0.172946277	-0.626518224
556264	558336	2 +	AMN1	ORF-T	ORF-T	-0.070906802	-0.057361755	-0.65000149	-0.25253618	0.015946292
558656	559784	2 +	IFA38	ORF-T	ORF-T	-0.332399542	-0.866457491	-0.40706604	0.025022133	-0.094210953
559976	561048	2 +	CDC28	ORF-T	ORF-T	0.178600571	-0.029211039	0.086561027	-0.136222903	-0.180668036
561416	562912	2 +	CSH1	ORF-T	ORF-T	-0.062868411	-0.859888747	-0.015254854	0.033896034	-0.132889714
565192	565512	2 +	YSY6	ORF-T	ORF-T	-0.126886861	-0.991370873	-0.110239927	-0.462284274	0.034567615
565512	567400	2 +	DEM1	ORF-T	ORF-T	0.533478153	-0.310225902	-0.532638499	-0.236291547	-0.213682536
568768	569752	2 +	UBS1	ORF-T	ORF-T	-0.25371103	-0.711851258	0.069229618	-0.049975813	0.441454312
571440	572000	2 +	unknown	no_ovlp2	CUTs	2.4841322	0.291735189	-0.142769491	-0.782934424	-1.067861619
572000	573816	2 +	PEX32	ORF-T	ORF-T	-0.220070781	-0.856855836	-0.523545306	-0.569601274	-1.446924543
576368	576704	2 +	CUT029	CUTs	CUTs	2.222939949	0.043003093	0.912414632	0.810709163	1.332034881
578344	579112	2 +	SEC66	ORF-T	ORF-T	-0.055540148	-1.046010978	-1.040655076	-0.184563338	-0.614152833
581744	582416	2 +	unknown	no_ovlp	CUTs	1.247920816	0.238730457	-0.73905583	-0.385857604	-0.406429484
582416	583464	2 +	SWD3	ORF-T	ORF-T	0.370028204	-0.369579587	-0.218125877	-0.496990238	0.292060451
583632	584736	2 +	ECM31	ORF-T	ORF-T	0.589726605	0.032000669	-0.131653268	-0.396126402	0.604102592
586560	587616	2 +	unknown	no_ovlp	CUTs	1.635821714	0.591156271	-1.291575964	-0.981036603	-0.915402101
596104	597160	2 +	YPC1	ORF-T	ORF-T	-0.089784734	-1.060823115	0.018650559	0.448859671	-0.51766272
597160	598616	2 +	YBR184W	ORF-T	ORF-T	-0.364686903	0.351937701	0.16532468	0.216900056	2.27810365
598616	599048	2 +	unknown	no_ovlp	SUTs	0.156386832	-0.205398605	0.324193329	-0.027261126	0.470884543
602512	603632	2 +	GDT1	ORF-T	ORF-T	-0.411813549	-0.926112068	-0.651305751	-0.168465768	0.351295825
604456	604935	2 +	RPS9B	ORF-T	ORF-T	0.942273934	-0.318322882	0.260887002	-0.722060319	0.284625288
606272	606720	2 +	YBR190W, RI	other	other	1.153166721	-0.681630714	-1.226890712	-1.198421615	-0.312643568
607464	609048	2 +	RIM2	ORF-T	ORF-T	-0.517848221	-0.737380686	-0.390024329	-0.397515828	0.022399795
609960	610656	2 +	SOY1	ORF-T	ORF-T	0.095154547	-1.223664377	0.03068811	-0.516933365	-0.310743024
614224	614472	2 +	unknown	no_ovlp	CUTs	1.818977424	-0.092699413	-0.52074675	-0.931585356	-0.360549609
614472	614880	2 +	CUT030	CUTs	CUTs	2.013468769	-0.548982058	-0.719120882	-0.397570482	-0.371626998
615408	616176	2 +	unknown	no_ovlp	SUTs	0.1374126	-1.133428935	-0.446533936	-0.286383411	0.645321828
618816	620432	2 +	KTR4	ORF-T	ORF-T	-0.266111354	-0.291992154	0.09434316	0.361679429	0.123583453
620552	620816	2 +	unknown	no_ovlp	SUTs	-0.724018218	-0.026862913	-0.168126884	0.16398022	0.123326
620816	622744	2 +	BEM1	ORF-T	ORF-T	0.024630612	-0.164539967	-0.195666593	0.180043702	-0.059735452
623480	624328	2 +	DER1	ORF-T	ORF-T	-0.289425971	-0.565232988	0.033215471	-0.292811921	-0.54453336
625360	625768	2 +	CUT031	CUTs	CUTs	1.827060874	0.179116573	0.055620577	-0.312538612	-0.889898836
625768	628488	2 +	CDC47	ORF-T	ORF-T	0.011902616	-0.322315161	-0.699765929	-0.25835	-0.272964508
628976	631224	2 +	COS111	ORF-T	ORF-T	-0.311813067	-0.848228822	0.704378167	0.596402494	-0.075517414
633576	634992	2 +	KTR3, YBR20	ORF-T	ORF-T	-0.35596278	-0.350279661	-0.208807825	-0.16846907	0.162766018
635112	636656	2 +	FTH1	ORF-T	ORF-T	-0.51202748	-0.720912748	-0.340960353	0.082255072	-0.200979719
643280	644560	2 +	unknown	no_ovlp	SUTs	0.876395307	-0.183809163	-0.88295793	-0.074015129	-0.835878383
645512	646216	2 +	ERV15	ORF-T	ORF-T	-0.279794279	-1.232665758	-0.239854989	-0.081527718	-0.24433254
647504	650104	2 +	NGR1	ORF-T	ORF-T	-0.743547362	-0.468081248	0.685480147	0.394315607	0.541299915
650104	651328	2 +	MET8	ORF-T	ORF-T	-0.385304162	0.122586306	-1.005890712	-0.541143652	0.095463106
651328	653184	2 +	SDS24	ORF-T	ORF-T	-0.52454025	-0.748438954	0.536531409	0.240506358	0.29845196

653456	654984	2	+	HPC2	ORF-T	ORF-T	0.1572547	-0.552915048	-0.006818453	-0.246831855	0.037271332
657792	658736	2	+	ATG12	ORF-T	ORF-T	0.219609017	-0.431667578	0.122176278	-0.345276788	-0.036332349
670488	673576	2	+	YBR224W, YE	ORF-T	ORF-T	-0.220805601	-0.637513669	0.401211722	0.193099039	-0.202696286
675336	676304	2	+	SLX1	ORF-T	ORF-T	0.827578964	-0.018494565	-0.478898317	-0.177250918	-0.540051008
679440	680352	2	+	unknown	no_ovlp	SUTs	-0.003338398	0.624312393	0.221263873	0.147563242	0.12931374
680352	680696	2	+	YBR230W-A	ORF-T	ORF-T	-1.0836189	-0.877049414	1.050134518	0.380493095	-0.612050322
683304	684720	2	+	PBP2	ORF-T	ORF-T	-0.055994304	-0.233125261	-0.306988316	-0.027831805	-0.133157092
684952	685504	2	+	DAD3	ORF-T	ORF-T	-0.91185937	-1.404727525	-0.205091039	-0.167341346	0.972199214
686816	690360	2	+	YBR235W	ORF-T	ORF-T	-0.081718597	-0.100503073	-0.00030633	0.043253408	0.249814566
691944	694632	2	+	PRP5	ORF-T	ORF-T	0.214181284	-0.323269957	-0.350424679	-0.066049371	-0.656868577
697872	698768	2	+	SUT021	SUTs	SUTs	0.289079931	-0.91633172	-0.510397329	-0.309275301	-0.515811439
704520	705472	2	+	YBR242W	ORF-T	ORF-T	0.462781416	-0.350893668	-0.234075244	-0.196352571	-0.04183391
707032	707312	2	+	unknown	no_ovlp2	CUTs	1.743185902	0.137906518	-0.344089118	-0.351118047	-0.40224031
707312	708168	2	+	GPX2	ORF-T	ORF-T	0.067734912	-0.297353627	0.234151907	-0.396473651	-0.679040765
711560	712896	2	+	YBR246W	ORF-T	ORF-T	0.284906182	-0.210294504	-0.262938821	-0.152664669	-0.028103489
714688	715472	2	+	CUT037	CUTs	CUTs	2.398260758	-0.6298459	-0.835789667	0.110949436	1.479751102
718408	718992	2	+	SPO23	ORF-T	ORF-T	2.004629494	-0.386434107	-0.808361336	-0.622203211	-1.715473426
721328	722400	2	+	MRPS5	ORF-T	ORF-T	-0.805510615	-1.226815634	-0.139675077	0.134138384	-0.188674284
722584	723128	2	+	DUT1	ORF-T	ORF-T	0.565920743	0.030546791	-0.490789497	-0.134426112	0.293276611
723216	723720	2	+	SRB6	ORF-T	ORF-T	0.249465386	-0.238591365	0.374287374	-0.064886545	0.399072193
724440	726600	2	+	YBR255W	ORF-T	ORF-T	0.182102424	-0.234799182	0.021505887	-0.142592496	0.08894734
728856	729872	2	+	POP4	ORF-T	ORF-T	-0.058762943	-0.666820445	-0.030407749	0.022610007	0.790250555
730360	732568	2	+	YBR259W	ORF-T	ORF-T	0.291878842	-0.407355382	-0.354704783	-0.566230444	-0.233924189
734816	736224	2	+	CUT038	CUTs	CUTs	2.054522805	-0.021226824	-0.480455991	-0.361897283	0.273530089
736224	737808	2	+	SHM1	ORF-T	ORF-T	-0.079131683	-0.135689017	-0.415760532	0.006553086	-0.128079982
738560	739680	2	+	TSC10	ORF-T	ORF-T	-0.489636957	-0.420112758	-0.395374418	0.142468812	0.233032448
739832	741136	2	+	REI1	ORF-T	ORF-T	0.823202741	0.016622188	-0.589412997	-0.099572954	0.449013632
741280	741712	2	+	MRPL37	ORF-T	ORF-T	-0.861379006	-0.779248345	0.329843234	0.156954338	0.174514092
742856	743424	2	+	unknown	no_ovlp	CUTs	1.681947823	-0.006283998	-0.410044374	0.089311211	-0.388042513
744824	746224	2	+	YBR271W	ORF-T	ORF-T	0.148533199	-0.295232954	-0.32981392	-0.348879003	0.209691672
749560	751384	2	+	CHK1	ORF-T	ORF-T	-0.13376883	-0.538933158	-0.141456419	-0.094518586	0.150152875
760320	761008	2	+	DPB3	ORF-T	ORF-T	0.685352501	-0.011481997	0.44288392	-0.314408098	-0.253356368
761224	762720	2	+	PAF1	ORF-T	ORF-T	-0.10570094	-0.646962998	-0.341797146	0.283363855	-0.563907166
763824	764928	2	+	unknown	no_ovlp	CUTs	1.345665979	0.319204366	-1.375108058	-0.903345184	-0.929989934
768208	768760	2	+	MRPL27	ORF-T	ORF-T	-1.009910755	-0.899739873	-0.316972045	-0.020927999	0.067363394
768968	770880	2	+	unknown	no_ovlp	SUTs	0.682335224	-0.165417105	0.463723138	0.094512864	0.46747428
770880	774688	2	+	YBR284W	ORF-T	ORF-T	1.317968052	0.518508484	0.506082804	0.438655582	0.888076741
773776	774688	2	+	unknown	no_ovlp2	SUTs	0.016914241	0.631680697	3.015044923	2.277402184	-1.260016984
774688	776376	2	+	APE3	ORF-T	ORF-T	-0.290372883	-0.345406852	0.109493589	0.383991593	0.134320505
776544	778008	2	+	ZSP1	ORF-T	ORF-T	-0.123951857	-0.466940471	-0.18795179	0.109077943	0.087276014
779672	782376	2	+	SNF5	ORF-T	ORF-T	-0.028404388	-0.092404841	0.182352996	-0.012673635	0.113667478
782576	783680	2	+	BSD2	ORF-T	ORF-T	0.086336719	-0.780299029	-0.070095007	0.132538847	-0.376252756
785048	786416	2	+	SUT023	SUTs	SUTs	1.307091497	-0.080815151	-0.476274744	-0.038673056	-0.580556962
787104	788664	2	+	VBA2	ORF-T	ORF-T	0.020615592	-0.612221435	-0.342968015	-0.103043717	-0.720000093
792800	796656	2	+	PCA1	ORF-T	ORF-T	0.331689254	0.01748508	-0.079515979	0.022491826	-0.342509535
800488	802168	2	+	MAL33	ORF-T	ORF-T	1.560785889	0.005047143	0.449276688	0.831736662	-1.365680737
18701	22149	3	-	MRC1	ORF-T	ORF-T	-0.044706543	-0.303615455	-0.32004715	-0.355040281	0.299599117
22325	23413	3	-	KRR1	ORF-T	ORF-T	0.435135714	0.06933503	-0.438488779	-0.255304846	0.270533858
23901	24357	3	-	FYV5, YCL05	other	other	-0.229758425	0.450380047	0.738418808	-0.10363597	0.273027329
26861	27501	3	-	YCL056C	ORF-T	ORF-T	0.073704037	-0.517329909	0.021018764	-0.101719697	-0.69415898
28629	28941	3	-	CUT460	CUTs	CUTs	3.402888965	0.24806812	-1.332784537	-0.569983576	0.43675273
28941	29501	3	-	unknown	no_ovlp	SUTs	0.738202653	-0.573447313	-1.536495503	-1.240664157	-1.885094096
29893	30405	3	-	CUT461	CUTs	CUTs	2.335530248	0.111351247	-1.074707595	-0.823762335	-0.885452738
30853	31189	3	-	CUT462	CUTs	CUTs	1.955770011	0.721141763	0.756906598	0.00429532	-1.612192266
33981	35413	3	-	PBN1	ORF-T	ORF-T	0.082183408	-0.240156112	0.158732345	-0.212811873	-0.099525979
37725	38869	3	-	APA1	ORF-T	ORF-T	0.450188648	-0.211123916	-0.835506814	-0.148652842	-0.841708547
39629	40789	3	-	YCL049C	ORF-T	ORF-T	0.781117882	0.037367505	-0.218932014	-0.008917339	-1.165916732
43557	44461	3	-	YCL047C	ORF-T	ORF-T	-0.061336017	-0.199157157	0.032039231	-0.210581205	-0.543730952
45869	46893	3	-	YCL045C	ORF-T	ORF-T	-0.406252971	-0.448926237	-0.389606122	0.007906606	-0.049661561
46893	48405	3	-	MGR1	ORF-T	ORF-T	-0.644261674	-0.174905186	0.24092191	-0.103472862	0.502334559
48573	50277	3	-	PDI1, YCL041	ORF-T	ORF-T	-0.643020221	-0.023916519	-0.339035673	-0.178984877	0.065178815
54885	56565	3	-	ATG22	ORF-T	ORF-T	0.25466733	0.246780715	0.560886894	0.339023533	0.946207712
57261	58741	3	-	SRO9	ORF-T	ORF-T	0.141692708	0.096906946	-0.461550439	-0.258427365	-0.194312705
60741	61237	3	-	GRX1	ORF-T	ORF-T	0.107891053	0.033892722	0.673992099	0.129808337	-0.103284734
62709	63253	3	-	YCL033C	ORF-T	ORF-T	0.253901702	0.685817095	0.687704876	-0.058875935	0.838415972
64581	65613	3	-	RRP7	ORF-T	ORF-T	0.128990345	-0.702552541	-0.540006539	-0.152890088	-0.03785952
65805	68389	3	-	HIS4	ORF-T	ORF-T	-0.249518697	-0.749669823	-0.718579564	-0.411734651	-0.87341123
68389	69957	3	-	BIK1	ORF-T	ORF-T	0.398655829	0.252761269	-0.310578239	-0.621281499	0.041323126
73357	73997	3	-	HBN1	ORF-T	ORF-T	-0.720538879	-0.118683134	0.673237882	0.957131958	0.273782122
75933	77958	3	-	AGP1	ORF-T	ORF-T	-0.484290236	-0.425217575	-0.492982181	-0.29601633	-0.193819344
77958	78214	3	-	unknown	no_ovlp	SUTs	0.617142153	0.213197997	0.277493936	0.032740282	-0.088111428
82326	83038	3	-	unknown	no_ovlp	SUTs	0.752524657	-0.333678073	-0.817040753	0.189463483	-0.714464895
84622	90766	3	-	unknown	no_ovlp	SUTs	-0.333277356	-0.702083343	-0.098662336	0.054614552	-0.355498297
92478	94382	3	-	NFS1	ORF-T	ORF-T	-0.444368462	-0.029090456	0.117808289	-0.036147634	0.292526029
94494	95790	3	-	DCC1	ORF-T	ORF-T	-0.215105886	-0.457282977	0.223325435	-0.130737584	0.346445148
101198	101790	3	-	YCL012C	ORF-T	ORF-T	-0.24946907	-0.546164907	0.349985321	0.070095251	0.782011834
101974	103382	3	-	GBP2	ORF-T	ORF-T	-0.266188605	-0.749918571	-0.549825391	0.184484762	0.030993339
103382	104374	3	-	SGF29	ORF-T	ORF-T	-0.020982199	-0.379726723	-0.010285673	-0.044837149	-0.318228511
104494	105590	3	-	ILV6	ORF-T	ORF-T	0.010818133	-0.167038793	-0.670592438	-0.277207897	-0.362282329
105590	106862	3	-	STP22	ORF-T	ORF-T	-0.079468698	-0.401434911	-0.081236231	-0.251862536	0.004738921
106862	107262	3	-	unknown	no_ovlp	SUTs	0.980456908	0.108174831	0.109726701	-0.44156663	-0.162163676
107342	107710	3	-	unknown	no_ovlp	CUTs	2.695744354	0.30865427	-0.247323619	-0.2285231	-0.021106284

107814	108614	3	-	unknown	no_ovlp	SUTs	-0.017354228	-0.086424634	0.185679359	-0.052512904	0.341337161
110550	111574	3	-	YCL002C	ORF-T	ORF-T	-0.072348674	-0.688828967	-0.401297982	-0.02991589	0.112693459
113214	114230	3	-	SUT455	SUTs	SUTs	1.167496758	-0.555606945	-0.958719889	-0.473635139	-1.330309728
115422	117174	3	-	SUT456	SUTs	SUTs	0.179314193	-0.033123263	0.516956589	-0.145761265	-1.11109502
117366	118390	3	-	CDC10	ORF-T	ORF-T	-0.13564118	-0.134007701	0.064060649	-0.026963897	-0.062387392
119374	120510	3	-	YCP4	ORF-T	ORF-T	-0.686914871	-0.607123778	0.464806188	0.754225084	0.364108694
120838	122382	3	-	CIT2	ORF-T	ORF-T	0.016760131	-0.016623337	0.116698742	0.254111469	0.281616645
122382	123486	3	-	YCR006C, SU	other	other	0.092779184	-0.542422536	0.041076285	-0.255702826	-1.233055272
124462	125014	3	-	YCRCdelta6	other	other	3.432716244	-0.454120591	-0.711793464	-0.582448201	-2.272080367
126742	127518	3	-	YCR007C	ORF-T	ORF-T	0.211943229	0.010182561	0.333530073	-0.575584464	-0.792433609
130550	131598	3	-	RVS161	ORF-T	ORF-T	0.031539898	-0.506555917	-0.38652195	0.13621113	-0.305621131
133670	137054	3	-	ADP1	ORF-T	ORF-T	-0.380231449	-0.55669094	0.220833528	0.29127357	0.204731912
139182	141926	3	-	POL4	ORF-T	ORF-T	0.343913449	-0.029820323	0.44356917	-0.163795785	-0.026649178
141094	141926	3	-	unknown	no_ovlp2	SUTs	0.133936389	-0.576267507	-0.180835741	-0.280933672	-0.433356423
141926	142174	3	-	YCR015C	ORF-T	ORF-T	2.760438292	-0.023467586	-1.095926266	-0.88160802	-0.510055262
142174	142542	3	-	SNR33	other	other	0.41285874	-1.670771595	-0.120358256	0.087354801	-0.393547796
144574	147662	3	-	CWH43	ORF-T	ORF-T	-0.381500264	-0.405114484	-0.148352592	0.00621672	0.030762334
148126	151830	3	-	unknown	no_ovlp	CUTs	1.779475324	-0.419116345	-1.052233293	-0.133935225	-0.641466103
151830	152694	3	-	unknown	no_ovlp	CUTs	1.693726359	0.020021834	-0.173951866	-0.202948276	-0.369884111
154766	155102	3	-	PET18, MAK:	ORF-T	ORF-T	0.241000147	-0.350493216	0.025078565	-0.49622657	-0.262898065
155934	157198	3	-	HSP30	ORF-T	ORF-T	0.033994196	-1.13197041	-0.106970118	-0.335493222	-1.033614582
157198	157470	3	-	unknown	no_ovlp	CUTs	1.181541693	-1.12418919	-0.550737859	-0.286042598	-0.015878391
158302	160446	3	-	YCR023C	ORF-T	ORF-T	0.025196684	-0.460352335	-0.589936296	-0.147918301	-0.304249056
160758	162414	3	-	SLM5	ORF-T	ORF-T	-0.485104195	-0.127782367	0.271555169	-0.452765833	0.221412608
162414	163262	3	-	YCR024C-B, I	other	other	-0.334506465	-0.383423753	-0.051182458	-0.077174673	-0.142306501
163918	166390	3	-	YCR025C, NP	other	other	0.017844932	-0.148203933	-0.125410867	0.016666097	-0.528727877
166734	168182	3	-	RHB1	ORF-T	ORF-T	0.113599507	-1.061641151	-0.633770022	-0.186458134	-0.390162838
169062	169534	3	-	unknown	no_ovlp	CUTs	2.112798604	-0.870314667	-0.713985041	-0.688268653	-0.805096536
170142	172486	3	-	FEN2	ORF-T	ORF-T	-0.184932892	-0.634985975	-0.364492704	0.090444056	0.066470885
173214	173510	3	-	RIM1	ORF-T	ORF-T	-0.275701918	-0.751863269	-0.400495178	-0.347428601	0.067031187
173694	176494	3	-	SYP1	ORF-T	ORF-T	-0.399322801	-0.292793681	0.158792354	0.058981378	0.062509313
176494	176942	3	-	SUT457	SUTs	SUTs	0.159841717	-0.733403688	0.617569226	-0.127083983	-0.258033963
177902	178206	3	-	RPS14A	ORF-T	ORF-T	0.559371881	-1.284348051	-1.974917747	-1.408681961	-0.313002783
178494	178798	3	-	SNR189	other	other	0.41567605	-0.207731712	-0.147410504	0.013639675	-0.276978645
185254	186030	3	-	unknown	no_ovlp	CUTs	2.514856905	0.274575656	-0.284123058	0.503405441	0.705127359
191678	193038	3	-	RRP43	ORF-T	ORF-T	0.26522233	-0.573321941	-0.678837121	-0.178927046	-0.112458249
196342	197222	3	-	PHO87	ORF-T	ORF-T	0.061807163	-0.541764003	-0.267936703	-0.37321973	-0.288484815
197574	201153	3	-	unknown	no_ovlp2	SUTs	0.204275265	-0.459451818	0.104020602	0.278032028	-0.152436538
201153	205398	3	-	TAF2	ORF-T	ORF-T	0.025473989	-0.043696214	-0.139449093	-0.098122103	-0.289650266
206070	206686	3	-	YCR043C	ORF-T	ORF-T	-0.189674804	-0.704122443	-0.45311342	-0.65490014	-0.042020987
206782	207950	3	-	PER1	ORF-T	ORF-T	-0.400744334	-0.3018943	0.08878305	0.077274275	0.188744542
209830	210430	3	-	IMG1	ORF-T	ORF-T	-1.035363375	-0.335300278	0.401109286	0.222523584	0.184595192
210686	211630	3	-	BUD23	ORF-T	ORF-T	-0.161671565	-0.154114415	0.0151	0.199863343	0.412150286
213942	214806	3	-	unknown	no_ovlp	CUTs	1.133877631	-0.166519846	-0.731716277	-0.375304701	0.211490214
218310	220166	3	-	CTR86	ORF-T	ORF-T	0.75600484	-0.136838427	-0.278634047	-0.029083149	0.612897555
220350	221686	3	-	PWP2	ORF-T	ORF-T	0.078830958	-0.152879357	-0.615289578	0.109415988	0.265110536
221686	223318	3	-	unknown	no_ovlp2	SUTs	0.279007319	0.072423017	-0.879297273	-0.161486254	0.27181257
223318	224230	3	-	YIH1	ORF-T	ORF-T	0.4231491	0.815151934	0.036287459	-0.35391015	-0.071972103
224230	225430	3	-	unknown	no_ovlp	SUTs	0.622856205	1.136648456	0.72705214	0.005935543	0.77541938
225454	226278	3	-	SUT458	SUTs	SUTs	0.2525466	1.325292006	1.098132648	0.31739571	0.925242857
228086	228502	3	-	unknown	no_ovlp	CUTs	2.573029108	0.40881746	-0.652842211	-0.460832554	0.127265845
228502	228782	3	-	unknown	no_ovlp	SUTs	0.295896154	-0.046433515	-0.343824703	-0.166785789	0.363142263
233054	236326	3	-	SED4	ORF-T	ORF-T	-0.429079683	-0.500878163	-0.140623624	-0.209673835	0.027129981
236326	236830	3	-	unknown	no_ovlp	CUTs	1.956504463	0.394433132	0.006063695	-0.423972546	-1.680506047
237814	238702	3	-	CUT467	CUTs	CUTs	2.046722313	-0.009404398	-1.211780163	0.063242642	0.299450557
240022	240582	3	-	IMG2	ORF-T	ORF-T	-0.511271476	-0.799680538	-0.369441841	0.001418645	-0.321525526
240726	242374	3	-	RSA4	ORF-T	ORF-T	0.645667861	0.348563556	-0.819441299	-0.29500251	0.412327481
242526	246606	3	-	SSK22	ORF-T	ORF-T	0.272921931	0.026591009	-0.100447236	-0.137017898	0.112265892
247918	248790	3	-	ERS1	ORF-T	ORF-T	-0.9974103	-0.836871577	0.432267133	-0.050325619	0.947748955
249278	250014	3	-	YCR076C	ORF-T	ORF-T	-0.146500424	-0.539845264	-0.18556189	-0.542359686	0.100624671
250158	252670	3	-	PAT1	ORF-T	ORF-T	-0.017207043	-0.299555148	-0.497361913	0.175687374	-0.215745436
253342	254158	3	-	unknown	no_ovlp	CUTs	1.473334048	0.021031044	-1.022192629	-0.206078131	-0.186782526
259982	262662	3	-	TUP1	ORF-T	ORF-T	-0.454774184	-0.327506811	-0.394899435	0.072499979	-5.44E-05
263950	264486	3	-	LUG1	ORF-T	ORF-T	0.405581282	-0.832858929	-0.687467529	-0.204026159	0.410608061
264486	266638	3	-	unknown	no_ovlp	SUTs	-0.452445304	0.390999539	0.142985345	0.099549746	0.790820915
272238	272878	3	-	YCR090C	ORF-T	ORF-T	-0.124213863	-0.901140593	-0.051463346	-0.38804199	-0.603539949
276614	279846	3	-	MSH3	ORF-T	ORF-T	-0.047152445	-0.531225395	-0.457957712	-0.307732954	-0.105453041
288102	289294	3	-	OCA4	ORF-T	ORF-T	0.140753888	-0.399614005	-0.219956399	-0.182283199	-0.581094626
293846	294254	3	-	unknown	no_ovlp	CUTs	1.111212978	0.103634495	0.553571015	0.433093245	-0.130358663
296870	299062	3	-	GIT1	ORF-T	ORF-T	-1.133949606	0.787296899	0.557855361	1.370026797	-0.340893934
298798	299062	3	-	unknown	no_ovlp2	SUTs	0.612431839	-0.067075679	0.069330233	-0.116228097	-1.567991314
300614	303238	3	-	YCR099C, YC	ORF-T	ORF-T	0.377606377	-0.052541359	0.057322654	-0.00805522	-2.503663648
302990	303238	3	-	unknown	no_ovlp2	SUTs	-0.003898879	0.259463491	0.853089183	0.459685206	-2.498431019
304550	306038	3	-	unknown	no_ovlp	SUTs	-0.615887991	-0.645064787	-0.433423956	-0.113436386	-1.658068696
308310	310078	3	-	SUT459	SUTs	SUTs	-0.057186563	-0.068747527	0.773972767	0.137733461	-2.362553809
312486	315374	3	-	unknown	no_ovlp	CUTs	4.190774114	0.342592439	-2.061619419	-2.129439143	-4.283155119
17257	18705	3	+	VAC17	ORF-T	ORF-T	-0.102359168	-0.467531979	-0.554098731	-0.340775374	0.470598604
23569	24017	3	+	YCL058W-A	ORF-T	ORF-T	2.129176914	0.436720076	-0.679027682	-0.62366848	0.008197908
24769	27041	3	+	PRD1	ORF-T	ORF-T	-0.096101134	-0.101474428	0.143375806	-0.275847924	0.099815429
27929	30497	3	+	KAR4	ORF-T	ORF-T	0.678747189	0.065602259	0.040064036	-0.282111956	-0.125834108
28945	30497	3	+	unknown	no_ovlp2	SUTs	0.090697638	-0.631516258	-0.528641216	-0.814885065	-0.407131877
30873	31233	3	+	unknown	no_ovlp	SUTs	-0.573299143	-1.0653709	-1.429963153	-2.591164135	-2.144722717

31441	31961	3	+	SPB1	ORF-T	ORF-T	0.194501103	-0.008729998	-0.864658515	-0.07741793	0.582675581
35593	37105	3	+	LRE1	ORF-T	ORF-T	-0.05416043	-0.255986517	-0.058786676	0.032178274	-0.430793538
39065	39337	3	+	CUT040	CUTs	CUTs	2.36967727	0.12345992	0.062548249	0.258012229	-1.061867837
40953	41369	3	+	SUT025	SUTs	SUTs	0.914574492	-0.190183766	0.674101539	0.193467445	-1.1163431
41369	41705	3	+	YCL048W-A	ORF-T	ORF-T	-0.694739737	-0.220439362	2.301443345	0.390603558	-0.770573838
47137	50801	3	+	SUT026	SUTs	SUTs	0.207763565	0.320891752	-0.318297587	-0.064609292	0.88233795
50801	52513	3	+	YCL042W, GL	ORF-T	ORF-T	-0.55175063	-0.410370372	0.429748123	0.345455012	0.133358137
52633	54953	3	+	GID7	ORF-T	ORF-T	-0.123192287	-0.201442249	0.141455395	-0.054297732	0.343602377
59009	60825	3	+	GFD2	ORF-T	ORF-T	0.396695313	0.156782945	-0.480080174	-0.03509195	1.501655916
61633	62793	3	+	LSB5	ORF-T	ORF-T	-0.166781874	-0.171611741	0.374368151	0.263736075	0.212711128
63417	64217	3	+	STE50	ORF-T	ORF-T	0.089913754	-0.364547176	0.301594501	0.053282166	0.279544131
68881	69425	3	+	CUT041	CUTs	CUTs	2.954443585	-0.560913821	-0.648150632	-0.457236058	-0.06102373
70129	71489	3	+	RNQ1	ORF-T	ORF-T	0.022560387	-0.052317297	-0.284622164	-0.013063078	-0.276927148
71817	73473	3	+	unknown	no_ovlp	SUTs	0.565604387	0.782435974	0.205267026	-0.443357849	-1.292375817
79114	82322	3	+	KCC4	ORF-T	ORF-T	-0.067943147	0.127236941	-0.399738474	-0.137052309	0.889798844
83146	83802	3	+	unknown	no_ovlp	CUTs	1.673958296	0.210964443	-1.330010288	-0.543610744	-0.474038372
91322	92498	3	+	LEU2	ORF-T	ORF-T	-0.566337705	-1.041715971	-0.021319305	0.531879179	0.385296095
96194	99442	3	+	BUD3	ORF-T	ORF-T	-0.37041342	0.063700963	-0.065702351	-0.049435024	0.72716533
103602	104410	3	+	unknown	no_ovlp	SUTs	0.892069333	-0.620456129	-1.147055484	-0.141668469	0.370378502
105802	106410	3	+	CUT042	CUTs	CUTs	2.173757251	0.17250667	-1.239102473	-0.487808684	-0.33670857
107298	107538	3	+	VMA9	ORF-T	ORF-T	-0.326178714	-0.479922126	0.115510546	-0.100015556	-0.048742484
108010	108882	3	+	LDB16	ORF-T	ORF-T	-0.279120773	-0.700217114	-0.058905402	-0.268902513	0.032497676
109058	110714	3	+	PGS1	ORF-T	ORF-T	0.823893805	-0.239195005	-0.457914906	-0.142721983	-0.477430698
111858	112586	3	+	RER1	ORF-T	ORF-T	0.091225015	-1.05021577	-0.737358693	-0.375729173	-0.512885127
118610	119274	3	+	MRPL32	ORF-T	ORF-T	-0.611717281	-0.589204339	0.048723236	-0.445098378	0.094233918
120330	121098	3	+	CUT043	CUTs	CUTs	1.574467471	0.354294022	0.359816035	-0.038863067	0.156840613
123066	123562	3	+	unknown	no_ovlp	CUTs	1.760769753	0.432187905	0.191610764	0.176709687	-0.961888425
125514	126994	3	+	unknown	no_ovlp	SUTs	0.887472869	-0.415285795	-0.203209985	0.112149622	-0.618663994
128042	129074	3	+	SAT4	ORF-T	ORF-T	-0.190184285	-0.978016804	-0.648018627	-0.031328903	-0.626485445
131826	132242	3	+	CUT044	CUTs	CUTs	2.007239799	-0.315554564	0.870441668	0.361216755	-1.883519686
137722	139170	3	+	PGK1	ORF-T	ORF-T	-0.641196711	-0.229935257	-0.190716603	0.030088957	-0.151472628
139306	140170	3	+	CUT045	CUTs	CUTs	3.386609837	0.215856569	-2.148977527	-0.86439202	0.114784429
143618	144658	3	+	YCR016W	ORF-T	ORF-T	0.279594993	-0.378953192	-0.289580053	-0.151121509	0.127799495
147890	152818	3	+	unknown	no_ovlp	CUTs	1.28501099	0.250727612	-0.150659233	-0.113543812	-0.827365642
152818	154034	3	+	MAK32	ORF-T	ORF-T	-0.237998381	-0.337196454	0.15437819	-0.19101604	-0.456218652
155306	156034	3	+	HTL1	ORF-T	ORF-T	0.331276888	-0.859914201	-0.484825566	-0.122812524	-0.463434541
157882	158426	3	+	SUT032	SUTs	SUTs	0.684376148	-0.814834086	0.011894683	0.090306712	-0.672579406
160690	161794	3	+	unknown	no_ovlp	CUTs	1.513775151	0.041833546	-1.016043822	-0.087207393	-0.435943973
164018	164306	3	+	CUT047	CUTs	CUTs	2.82774008	0.749937659	-0.637758196	-0.317606313	-0.510943759
169066	169866	3	+	unknown	no_ovlp	SUTs	0.46085816	-0.562559039	-0.7004327	-0.129559628	-0.197002324
179026	179234	3	+	CUT048	CUTs	CUTs	1.283440864	-0.97324706	-0.26271101	-0.205560259	1.094492834
179442	180642	3	+	BPH1	ORF-T	ORF-T	0.21058788	-0.308408316	0.091139077	0.072646818	-0.117412665
186298	190330	3	+	SNT1	ORF-T	ORF-T	0.132387974	-0.376274398	-0.335113951	-0.210627637	-0.397311411
190570	191802	3	+	FEN1	ORF-T	ORF-T	-0.745803523	-0.522056286	-0.506585227	0.003772292	0.279986062
191882	193282	3	+	unknown	no_ovlp	SUTs	0.189932521	0.570788352	0.010233997	-0.11416224	0.23136668
193282	194378	3	+	RBK1	ORF-T	ORF-T	0.011698724	-0.408083579	0.283130517	-0.217539464	-0.148936345
201306	202706	3	+	unknown	no_ovlp	SUTs	-0.066489213	0.671352084	0.137299812	-0.038568876	0.421397317
202706	204394	3	+	SUT034	SUTs	SUTs	1.297766005	-0.10667074	-1.085399357	0.028467118	0.606104321
206882	208154	3	+	SUT036	SUTs	SUTs	1.620608493	-0.204014695	-1.713514193	-0.956201251	0.202036345
208154	210138	3	+	YCR045W-A	other	other	0.453152658	-0.160098523	-0.403062338	-0.382066317	-0.166962248
209858	210138	3	+	unknown	no_ovlp2	SUTs	0.897936884	-0.536741079	-0.209227702	0.376279699	-0.067722928
211882	213962	3	+	ARE1	ORF-T	ORF-T	-0.331130144	0.147122341	-0.063345167	0.012794259	0.317689283
214050	214866	3	+	YCR051W	ORF-T	ORF-T	-0.026727377	0.032488452	-0.150151515	0.005497865	0.100880765
214954	216634	3	+	RSC6	ORF-T	ORF-T	-0.106330328	-0.187737871	-0.418724564	-0.049895699	-0.428226444
216634	218330	3	+	THR4	ORF-T	ORF-T	-0.094071282	-0.268023903	-0.452114586	-0.030932301	-0.346366445
223530	224474	3	+	SUT037	SUTs	SUTs	1.780564463	-0.355450446	-0.972828033	-0.442240735	0.535837435
224474	224850	3	+	unknown	no_ovlp	SUTs	0.70557706	0.309361461	0.231609534	-0.234238422	-0.117997719
225514	227826	3	+	YCR061W	ORF-T	ORF-T	0.338478745	-0.248057128	0.092633878	0.292904634	0.641792886
228306	229794	3	+	BUD31	ORF-T	ORF-T	0.767317766	0.715859133	-0.170797489	-0.410987081	-0.236408588
229274	229794	3	+	unknown	no_ovlp2	SUTs	-0.597703329	-0.343762054	-0.050460147	0.031049525	0.27143378
229794	231154	3	+	HCM1	ORF-T	ORF-T	-0.453415081	-0.230727169	-0.302431319	-0.241032161	0.156855331
231458	233026	3	+	RAD18	ORF-T	ORF-T	0.274154293	-0.224821792	0.249846742	0.188728424	0.251246235
236818	237402	3	+	unknown	no_ovlp	SUTs	0.196403277	-0.692683521	0.021984484	0.017320775	-0.137323947
237402	238930	3	+	ATG15	ORF-T	ORF-T	-0.259638254	-0.069829365	0.38413267	0.007377876	0.333156033
238930	240130	3	+	CPR4	ORF-T	ORF-T	-0.433765069	-0.347475815	-0.216040769	0.00805758	0.502011984
242618	244114	3	+	SUT038	SUTs	SUTs	-0.058161948	-1.076765766	-0.884526025	-0.287254983	0.712681625
246954	248018	3	+	SOL2	ORF-T	ORF-T	0.099767553	0.074268008	-0.022453045	0.002952432	0.181512972
248970	249258	3	+	YCR075W-A	ORF-T	ORF-T	-0.401456194	-0.374265408	0.957329075	0.091471834	0.996420275
252842	254298	3	+	PTC6	ORF-T	ORF-T	-0.427128902	-0.321429666	0.564206279	-0.022535863	0.390975698
254378	257442	3	+	SRB8	ORF-T	ORF-T	0.237184064	-0.155273474	-0.066210196	-0.454497246	-0.055045037
258850	259434	3	+	AHC2	ORF-T	ORF-T	-0.31094615	-0.979460193	0.197302596	0.116490753	0.478929095
259578	260082	3	+	TRX3	ORF-T	ORF-T	0.501183289	0.576389806	1.070498396	0.371150648	0.685351832
261850	262170	3	+	unknown	no_ovlp	SUTs	-0.863212852	-0.814961177	0.196866998	0.647811583	-0.446365013
263010	263338	3	+	unknown	no_ovlp	SUTs	0.479503655	-0.428775443	-0.511939247	-0.825204041	0.042580774
263338	263994	3	+	CSM1	ORF-T	ORF-T	0.357401286	0.429991919	0.465202618	-0.372279324	0.951973202
265026	266986	3	+	ABP1	ORF-T	ORF-T	-0.238128406	0.069000566	-0.173093435	0.180182371	-0.138028337
267146	268930	3	+	FIG2	ORF-T	ORF-T	-0.211506314	-0.0705977	0.215407162	-0.057679699	0.483975266
274354	276546	3	+	KIN82	ORF-T	ORF-T	-0.596180505	-0.985430266	0.782986917	1.226676689	-0.75899959
280090	286538	3	+	CDC39	ORF-T	ORF-T	-0.227963039	-0.356678527	-0.11317071	0.02238176	-0.141564184
286714	288210	3	+	CDC50	ORF-T	ORF-T	-0.119526942	-0.613207836	-0.043660576	0.138732441	-0.592104664
289546	290730	3	+	YCR095W-A	ORF-T	ORF-T	0.465848781	-0.268439388	-0.24138523	-0.216617798	-2.089187513
293746	294002	3	+	unknown	no_ovlp	SUTs	0.651300314	0.030770935	-0.410986271	-0.631687134	-1.050636713

294002	294234	3	+	HMRA1	other	other	0.58455946	0.080516942	0.476200369	-0.206473813	-0.326627889
299154	300602	3	+	unknown	no_ovlp	SUTs	-0.247056458	0.491951937	1.126262372	1.226556731	-0.888629774
17469	20149	4	-	unknown	no_ovlp	CUTs	1.448002873	0.135582567	-0.116073683	-0.272122536	-1.392994462
21773	22461	4	-	CUT468	CUTs	CUTs	2.461997874	-0.038317703	-0.032502885	0.068836012	-0.609087225
26317	27821	4	-	ADY3	ORF-T	ORF-T	1.185542107	0.78775169	0.149948909	-0.217598433	0.032316151
27821	28957	4	-	unknown	no_ovlp2	SUTs	0.788596644	0.365956063	0.330030741	-0.259459141	-0.207780503
28957	30445	4	-	GUD1	ORF-T	ORF-T	0.75373343	0.816936729	0.654036291	0.000282219	0.030605059
31669	32045	4	-	CUT469	CUTs	CUTs	2.320386905	-0.866064489	-0.74081806	-0.136275949	-0.411677393
33301	33957	4	-	YPD1	ORF-T	ORF-T	-0.197531558	-0.457721085	-0.107857107	-0.196911835	0.113831401
34413	36605	4	-	GYP7	ORF-T	ORF-T	0.062556915	-0.371858627	0.48578866	0.632994324	-0.279776373
38877	42349	4	-	BRE4	ORF-T	ORF-T	0.061126355	-0.564440087	-0.418172267	-0.222620874	-0.402456257
46277	48021	4	-	unknown	no_ovlp	SUTs	-2.258990708	-0.68815233	-1.203026791	1.1869925	2.081370336
50917	52197	4	-	GCS1	ORF-T	ORF-T	-0.425217816	-0.410120421	-0.534443164	-0.087760964	-0.113935341
54181	56725	4	-	WHI4	ORF-T	ORF-T	-0.341693674	-0.499634366	-0.23662198	-0.113145873	-0.110463496
56957	59949	4	-	HBT1	ORF-T	ORF-T	-1.385180082	-0.536569552	2.969935102	1.697365818	1.334563575
59949	61957	4	-	unknown	no_ovlp2	SUTs	-1.366438818	-1.045748913	2.809972061	1.812682356	1.11077874
61957	65901	4	-	CDC13	ORF-T	ORF-T	-0.283605581	-0.241019773	-0.247252586	-0.152602903	0.129832863
65045	65901	4	-	unknown	no_ovlp2	CUTs	1.781201973	0.127242692	-1.19033746	-0.441813664	0.558329168
67653	68765	4	-	TIM22	ORF-T	ORF-T	-0.176105054	-0.654259414	-0.289502461	-0.095340022	-0.375689233
68765	70533	4	-	RRI1	ORF-T	ORF-T	-0.810792653	-0.824078055	0.750108371	0.539138598	0.517501669
70533	74125	4	-	GDH2	ORF-T	ORF-T	0.224686371	-0.368740303	0.70527057	0.435485816	-0.371818952
74125	74437	4	-	unknown	no_ovlp2	SUTs	-1.144311907	0.445966781	2.133237212	1.545400111	0.249298332
74437	77261	4	-	PRR2	ORF-T	ORF-T	-0.574113149	0.726378839	2.221885442	1.917889804	-0.039367853
76613	77261	4	-	unknown	no_ovlp2	SUTs	-0.29002374	0.133456325	0.456843064	0.031163243	0.491280261
77989	78237	4	-	NOP6	ORF-T	ORF-T	-0.298446454	-0.16453454	0.565668888	-0.127214489	0.29028517
79189	80453	4	-	YDL211C	ORF-T	ORF-T	-0.378542272	0.01237341	-0.259015282	-0.670067844	0.746460798
86085	87245	4	-	CWC2	ORF-T	ORF-T	-0.320192294	-0.346378906	-0.128723215	-0.590672746	0.880703735
89477	89869	4	-	CUT471	CUTs	CUTs	1.798833599	0.508365934	0.653331091	0.952451389	0.406577813
92525	93949	4	-	HEM3	ORF-T	ORF-T	0.040140447	-0.620404595	-0.428933045	-0.004276542	-0.598709609
96013	98125	4	-	ACK1	ORF-T	ORF-T	-0.267232659	-0.441473284	-0.278314162	-0.174365702	-0.125666867
98837	99301	4	-	CUT472	CUTs	CUTs	2.384614689	0.281900633	-0.875850031	0.224775567	0.799418188
100389	101109	4	-	unknown	no_ovlp2	SUTs	-0.02821272	-0.359518257	0.722208256	0.295513073	0.298548284
101109	103541	4	-	YDL199C	ORF-T	ORF-T	-0.306736163	-0.286972268	0.914260294	0.786710471	1.20117761
103541	104637	4	-	GGC1	ORF-T	ORF-T	-0.740734917	-1.02712151	-1.089191634	-0.248868249	-0.49581564
104637	106869	4	-	ASF2	ORF-T	ORF-T	-0.623323244	-1.037135883	0.114551254	0.27418538	0.253168637
115165	116101	4	-	CUT473	CUTs	CUTs	1.274934819	0.025261936	-0.945100637	-0.417590433	0.549626403
119725	121789	4	-	UFD2	ORF-T	ORF-T	-0.126678094	-0.206786949	-0.185973294	-0.092359155	-0.047178713
123701	125181	4	-	PPH22	ORF-T	ORF-T	-0.526678726	-0.616239326	0.175807106	-0.165514205	0.164558567
125541	126461	4	-	YDL187C	other	other	0.741291959	-0.405324445	-0.350672647	-0.217928886	-0.289126204
130205	130541	4	-	RPL41A	ORF-T	ORF-T	-0.160583681	-0.04985055	-0.113967229	-0.169441904	0.070103
130749	132405	4	-	YDL183C	ORF-T	ORF-T	0.81124138	0.078819704	0.658090537	0.476755137	1.096681675
131901	132405	4	-	unknown	no_ovlp2	CUTs	1.833489829	0.111042999	-1.043245471	0.491996708	1.06849012
132405	132645	4	-	CUT474	CUTs	CUTs	2.514444705	-0.5655953	-0.371554783	0.048007381	1.197988527
133725	134733	4	-	CUT475	CUTs	CUTs	3.278233368	0.157821403	-0.265823021	-0.454688745	0.039403901
134733	135525	4	-	SUT460	SUTs	SUTs	0.346705198	-0.064136964	-0.116210607	-0.420954573	-1.029361525
137549	137933	4	-	unknown	no_ovlp	CUTs	2.34881927	0.625532678	1.143326807	-0.312474681	-0.956102525
138629	139325	4	-	CUT476	CUTs	CUTs	2.145715805	0.608581032	-0.053500686	-0.039817982	-0.64595678
141133	141741	4	-	YDL177C	ORF-T	ORF-T	-0.082487512	0.004623616	-0.235623237	-0.762288687	0.158528509
144277	145541	4	-	AIR2	ORF-T	ORF-T	0.211690831	-0.672614847	0.197209555	0.252829717	0.034253803
145741	147629	4	-	DLD1	ORF-T	ORF-T	-0.440540456	-0.728823789	-0.180622781	0.066786045	-0.020270192
147629	147973	4	-	unknown	no_ovlp	SUTs	0.107009529	-0.023204448	-0.413207509	-0.05105921	0.592604395
149085	155957	4	-	GLT1	ORF-T	ORF-T	-0.015807242	-0.432079953	-0.542073352	0.043339546	-0.25599088
157965	158765	4	-	UGX2	ORF-T	ORF-T	-0.80488622	-0.314927634	1.604491145	0.879256814	1.239298094
160853	163197	4	-	NRP1	ORF-T	ORF-T	0.750700444	0.277828655	-0.873403053	-0.163111069	0.118872584
163381	164085	4	-	FAP7	ORF-T	ORF-T	-0.270056977	-0.560504666	-0.316667024	-0.12014983	0.175527435
164861	167285	4	-	CDC9	ORF-T	ORF-T	-0.108618029	0.107223184	-0.298089913	-0.031847354	0.396445561
169165	169629	4	-	YDL160C-A	ORF-T	ORF-T	0.094430613	-0.470791483	-0.166976681	-0.039521982	-0.099075511
169789	172213	4	-	DHH1	ORF-T	ORF-T	-0.194272876	-0.340275292	-0.295794999	0.019236148	-0.209247463
173973	174629	4	-	YDL158C, YD	other	other	-0.05023842	-0.652941233	-0.696481886	-0.524166286	0.208888532
174629	176437	4	-	unknown	no_ovlp	SUTs	0.406846513	0.478687567	0.059293753	-0.12272161	1.909941595
181093	183045	4	-	SAS10	ORF-T	ORF-T	0.271542338	-0.432639267	-0.554800235	-0.010961297	0.378949827
187861	190597	4	-	NOP14	ORF-T	ORF-T	0.244490825	-0.398995661	-0.483350152	-0.033780132	0.368510232
194405	198437	4	-	COP1	ORF-T	ORF-T	-0.393831087	-0.225490052	-0.118313507	0.038075632	-0.19441217
198597	199733	4	-	YDL144C	ORF-T	ORF-T	-0.874226767	-0.291493514	0.332908457	-0.468312328	0.501510799
201605	202621	4	-	CRD1	ORF-T	ORF-T	-0.151325244	-0.496789367	0.498536679	-0.21150365	0.200044254
208693	211101	4	-	RPO21	ORF-T	ORF-T	-0.316805947	-0.125052104	-0.260342551	0.094390507	-0.146676342
211101	212085	4	-	SCM3	ORF-T	ORF-T	-0.127934343	0.042198544	0.397918851	-0.252358482	0.695129144
215325	217173	4	-	CUT480	CUTs	CUTs	1.749035792	-0.459263595	-1.883026586	-0.505906619	0.225016228
217173	218429	4	-	unknown	no_ovlp	SUTs	0.307848913	0.499125216	-0.136436437	-0.209393127	-0.2107414
218621	219357	4	-	RDI1	ORF-T	ORF-T	0.036005413	-0.390708836	-0.045458027	-0.295845247	0.126581171
219565	221245	4	-	PPH21	ORF-T	ORF-T	-0.036922052	-0.361794719	0.009150747	-0.159168365	-0.052374448
221517	221869	4	-	RPL41B	ORF-T	ORF-T	-0.651715029	-0.156592426	0.09268085	-0.155980734	0.277119087
221869	222509	4	-	unknown	no_ovlp	CUTs	1.538687227	0.345835564	0.079588886	-0.11446254	-0.09510542
226069	228877	4	-	unknown	no_ovlp	CUTs	1.522232099	0.197998812	-0.909914636	0.421946645	0.402564375
230725	232333	4	-	unknown	no_ovlp	CUTs	1.99855031	0.279252976	-1.332315259	-0.504248395	-2.203744779
236069	238717	4	-	CDC48	ORF-T	ORF-T	-0.085579478	-0.185038913	0.103863067	-0.000355765	-0.250888818
238885	239789	4	-	HNT1	ORF-T	ORF-T	0.277332362	-0.467253799	-0.36707488	-0.348288722	-0.210136004
239789	242021	4	-	unknown	no_ovlp	CUTs	1.009329873	0.01757083	-0.521914714	-0.220919816	-1.010023336
244925	245605	4	-	YDL121C	ORF-T	ORF-T	-0.190582591	-0.421923929	0.083415217	0.017580603	0.946259818
246549	247709	4	-	YDL119C	ORF-T	ORF-T	-0.121883317	-0.466042318	0.074534841	-0.411029228	-0.785734508
247709	248013	4	-	CUT481	CUTs	CUTs	3.198196588	-0.824704018	0.259409377	-0.060495223	0.044432472
253909	255165	4	-	IWR1	ORF-T	ORF-T	0.017753273	-0.761913165	-0.054352804	-0.076203181	-0.491195235

256549	258629	4	-	ATG20	ORF-T	ORF-T	0.230504588	-0.405123128	0.033637535	-0.018743455	-0.060681963
263261	264173	4	-	RRP42	ORF-T	ORF-T	-0.042589648	-0.479950447	-0.483110375	-0.239429781	-0.08550496
264341	265021	4	-	TMA17	ORF-T	ORF-T	0.366560485	0.090196413	0.808982326	0.705413832	0.679830575
265021	267349	4	-	YDL109C	ORF-T	ORF-T	-0.0393614	-0.596844646	-0.252562204	-0.157380121	-0.44825274
268509	269669	4	-	unknown	no_ovlp	CUTs	1.836610176	0.736685216	-0.006410964	0.209332386	0.32696496
270229	271965	4	-	PHO2	ORF-T	ORF-T	-0.239293026	-0.548865987	0.659209337	0.100643369	0.32948963
273573	275053	4	-	QRI7	ORF-T	ORF-T	-0.256458017	0.188926663	0.19788609	-0.124241973	0.59536353
275053	276645	4	-	QRI1	ORF-T	ORF-T	-0.118680981	-0.210988636	0.029980101	-0.209334842	0.467980969
280205	281853	4	-	DUN1	ORF-T	ORF-T	0.086661429	0.407563393	0.49474761	-0.094675818	1.519781283
282045	283205	4	-	GET3	ORF-T	ORF-T	-0.204917435	-0.336392084	0.126901546	-0.12262603	0.278007137
284469	285173	4	-	SNU23	ORF-T	ORF-T	-0.040426122	-0.481609035	-0.604731371	-0.695751607	0.165796784
285317	286733	4	-	RPN6	ORF-T	ORF-T	-0.071994713	-0.189128814	0.104139823	0.078837229	-0.039015215
286733	290405	4	-	unknown	no_ovlp	SUTs	0.430379623	0.519370629	-0.248344328	0.024105554	-0.215474841
292277	292573	4	-	CUT482	CUTs	CUTs	2.203162629	0.423060043	0.575236199	0.425588875	0.561570109
293325	294837	4	-	UBX3	ORF-T	ORF-T	0.889275943	0.723145839	0.327016167	0.051194886	0.118957125
294989	296373	4	-	RAM1	ORF-T	ORF-T	-0.262546292	-0.188818866	-0.165690397	-0.467555765	0.091299683
298309	300133	4	-	ASM4	ORF-T	ORF-T	-0.266558499	-1.209135056	0.063143588	0.189823557	-0.006479802
300133	301013	4	-	LUC7	ORF-T	ORF-T	0.052086206	-0.186773877	-0.102016961	-0.153796911	0.183545555
301445	302413	4	-	YDL086C-A	other	other	1.782790718	-0.2649071	-0.648569434	0.056697306	-0.349132065
302149	302413	4	-	unknown	no_ovlp2	SUTs	0.629842377	0.029010062	-0.238118469	0.668501011	-1.30335854
302413	302725	4	-	YDL085C-A	ORF-T	ORF-T	-1.283442186	-0.996342306	0.035884346	-0.106367599	-0.301178263
303957	304845	4	-	CUT483	CUTs	CUTs	2.144988098	-0.309486695	-0.474247697	-0.485165405	-0.012941038
307333	307773	4	-	RPS16B	ORF-T	ORF-T	0.156839919	-0.642301898	-0.861149847	-0.712934139	0.262345359
309701	310189	4	-	RPP1A	ORF-T	ORF-T	-0.597320382	-0.236703399	0.125283706	-0.122728181	-0.034082182
310485	312637	4	-	THI3	ORF-T	ORF-T	0.316388258	-0.588484691	-0.758849612	-0.272292049	-0.407016293
314301	314837	4	-	MRK1	ORF-T	ORF-T	-0.605589496	-1.665292676	1.622573099	1.550547619	0.18571624
315277	316453	4	-	MDH3	ORF-T	ORF-T	-0.062131425	-0.379139555	0.446046189	0.236369381	0.380294736
316885	320261	4	-	VAM6	ORF-T	ORF-T	-0.224225162	-0.434120059	-0.057089022	-0.294371725	0.242304956
320453	321693	4	-	RXT3	ORF-T	ORF-T	-0.401592214	-1.032077774	0.110057689	-0.326128911	0.334171651
323061	323469	4	-	SNR63	other	other	2.006721401	0.099344666	-0.293504599	-0.258874313	-0.068246731
323941	326229	4	-	BRE1	ORF-T	ORF-T	0.311877837	-0.587206362	-0.520174867	-0.103729988	-0.083785917
329757	330517	4	-	YET3	ORF-T	ORF-T	-0.388237659	-0.318028794	0.328987517	-0.034371002	0.352984228
330517	330901	4	-	unknown	no_ovlp	CUTs	1.398451704	-0.313682921	-0.131420705	-0.577870917	-0.827586438
333085	333997	4	-	CBS1	ORF-T	ORF-T	0.045657768	-0.238363434	-0.048031355	-0.412701324	-0.822191723
333997	334445	4	-	COX9	ORF-T	ORF-T	0.30792719	0.311831514	0.633089688	-0.15213325	0.420881299
336181	337293	4	-	PEX19	ORF-T	ORF-T	0.356320709	-0.186391776	0.157759077	-0.091666448	0.128637021
338237	340229	4	-	YDL063C	ORF-T	ORF-T	0.776141111	0.079446149	-0.496426042	-0.119592457	0.526754535
340805	341269	4	-	RPS29B	ORF-T	ORF-T	0.338933207	-0.97346508	-0.537227722	-1.148533885	-0.349556099
343981	345357	4	-	RAD59	ORF-T	ORF-T	0.282830142	0.354679025	0.38930187	-0.868303444	-0.105433357
345077	345357	4	-	unknown	no_ovlp2	SUTs	0.487930253	0.192182948	0.256907578	-0.30814137	-0.173737475
351773	352621	4	-	CUT487	CUTs	CUTs	1.441902839	0.066981035	-0.452991451	-0.159918554	0.854356197
355429	356909	4	-	PSA1	ORF-T	ORF-T	-0.344844596	-0.095734671	-0.608468201	-0.318027353	-0.072119009
358229	359037	4	-	unknown	no_ovlp	CUTs	2.231654319	1.129943169	0.440589156	0.179391814	-1.010550808
359757	361373	4	-	MCH1	ORF-T	ORF-T	-0.16510512	-0.083462933	0.256918	0.173034899	0.908416341
361661	362405	4	-	PBP4	ORF-T	ORF-T	0.056844479	-0.177808488	0.182592988	0.02604861	-0.026723362
362629	363629	4	-	SLC1	ORF-T	ORF-T	-0.275600147	-0.493576261	-0.399871828	-0.109689041	0.347382027
364853	366045	4	-	KNH1	ORF-T	ORF-T	-0.404949986	-0.866680364	-0.350128102	-0.15794635	0.052689332
366629	368293	4	-	STP4	ORF-T	ORF-T	0.271319678	-0.344023112	-0.265043825	-0.338730415	-0.542125708
368293	368725	4	-	CUT489	CUTs	CUTs	1.167654826	-0.544814289	0.010891756	0.001766683	-0.432230407
371037	371989	4	-	SUT462	SUTs	SUTs	0.854420671	0.24851374	-0.883943074	-0.038256752	-0.032868428
372573	373741	4	-	FAD1	ORF-T	ORF-T	-0.084834677	-0.327679915	0.184896043	-0.315991665	0.210000705
373893	375381	4	-	MTF2	ORF-T	ORF-T	-0.611602812	-0.991503958	-0.003007305	0.058812734	-0.108202258
375381	376509	4	-	PRP11	ORF-T	ORF-T	0.114447726	-0.525247929	-0.346695825	-0.491339777	-0.117408936
376733	378477	4	-	SIR2	ORF-T	ORF-T	-0.612157414	-0.219551374	-0.469723407	-0.58017814	-0.464494073
378845	381469	4	-	NAT1	ORF-T	ORF-T	-0.059456457	-0.199582692	-0.666344569	-0.008516946	-0.521915154
381877	385765	4	-	PRM7, YDL03	other	other	0.802089068	1.32140455	0.143873176	0.860277769	-3.072720672
387413	388853	4	-	PUS9	ORF-T	ORF-T	0.257992293	-0.509690709	-0.506980684	-0.101655777	-0.449742686
389029	392133	4	-	GPR1	ORF-T	ORF-T	-0.257297026	-0.294397387	0.171389204	0.276505169	-0.14378069
392133	392493	4	-	unknown	no_ovlp2	SUTs	-0.750692331	-0.538802816	0.289513301	0.198594769	-0.164796294
392493	393949	4	-	SLM3	ORF-T	ORF-T	0.731036999	-0.212957586	-0.433162868	-0.502957693	-0.022158653
398541	399069	4	-	unknown	no_ovlp	SUTs	0.944888494	-0.087101449	0.259973118	-0.093684771	1.414390584
400845	403789	4	-	MPS1	ORF-T	ORF-T	-0.117557541	-0.275752223	0.163592333	-0.017803739	0.241775347
403421	403789	4	-	unknown	no_ovlp2	SUTs	0.015145528	0.068176077	0.959815998	0.203309525	-0.049065034
403789	404989	4	-	YDL027C	ORF-T	ORF-T	0.34530486	0.547665338	0.696662029	0.045142695	-0.201761639
406517	407381	4	-	YDL025C	ORF-T	ORF-T	-0.514426181	-1.018798427	0.145418298	0.488511467	-0.674621719
407381	408205	4	-	unknown	no_ovlp2	SUTs	-0.209951276	0.022867755	1.069965223	0.766582544	-0.183397016
408205	411429	4	-	DIA3, YDL02	ORF-T	ORF-T	-0.058971076	0.878205353	1.739026569	0.672456483	-0.508286687
409837	411429	4	-	unknown	no_ovlp2	SUTs	0.018820102	0.458047099	0.61202826	0.313689612	-0.086463102
414213	414789	4	-	CUT491	CUTs	CUTs	2.436051992	-0.04168384	-0.087886596	0.164994612	-0.660314198
416405	416941	4	-	RPN4	ORF-T	ORF-T	0.337869059	-0.864322616	0.775056646	0.380031388	-0.344369138
417557	421717	4	-	OSH2	ORF-T	ORF-T	-0.267588172	-0.573188908	-0.062371163	0.123920019	-0.118521598
422661	423605	4	-	ERP3	ORF-T	ORF-T	-0.573375192	-0.618139145	0.495085007	0.110666167	0.418027438
425781	426981	4	-	YDL016C, TS(	other	other	-0.17521511	-0.567795174	-0.328631939	-0.1375746	-0.256686062
428429	428717	4	-	CUT492	CUTs	CUTs	2.057530925	-0.280622035	-0.604501467	0.299212427	-0.520826111
430949	432029	4	-	YDL012C	ORF-T	ORF-T	1.24863722	-0.328920679	-0.201650915	0.074953358	-0.290421982
432901	434237	4	-	SUT464	SUTs	SUTs	0.741488581	-0.074788339	-0.842048856	-0.237787355	0.021454249
440797	442333	4	-	MED2	ORF-T	ORF-T	-0.026980697	-0.218520315	0.066940547	0.13895261	-0.013779728
443789	444253	4	-	SUT465	SUTs	SUTs	3.296812722	-0.414549305	-0.536181546	-0.509416434	0.199419612
446621	447637	4	-	NHP10	ORF-T	ORF-T	-0.036264302	-0.906266955	-0.416762159	0.067736915	-0.389866249
450109	452717	4	-	NTH1	ORF-T	ORF-T	-0.502208144	-0.665545848	0.456865638	0.294091695	-0.262251224
454037	455013	4	-	unknown	no_ovlp	CUTs	1.591681667	0.026700987	-1.090285288	-0.144390306	0.507302745
456677	458237	4	-	MAF1	ORF-T	ORF-T	-0.27263788	-0.811376488	-0.040244836	0.563396237	0.097580212

458469	461349	4	-	SOK1	ORF-T	ORF-T	0.222941335	-0.616373222	-0.00880232	-0.127711469	-0.2902123
461821	462397	4	-	YDR008C	other	other	2.548592429	0.443341485	0.097167565	0.207305075	0.157393905
462397	465773	4	-	YDR008C	other	other	-0.078806943	0.413267324	1.459381812	0.843595446	0.177890541
463149	465773	4	-	unknown	no_ovlp2	SUTs	0.415610889	0.406919258	-0.234246686	0.301016522	-0.250768669
470541	471501	4	-	SUT466	SUTs	SUTs	1.921519957	0.049810755	-0.263615428	-0.3539388	-0.094983321
478334	478790	4	-	DAD1	ORF-T	ORF-T	-0.989825027	-1.542619753	0.186659442	0.332684552	0.708549617
478950	482270	4	-	KCS1	ORF-T	ORF-T	-0.090436964	-0.609479908	-0.326356496	-0.031121909	0.08426486
482270	482494	4	-	unknown	no_ovlp2	SUTs	0.213153662	-0.385900215	-0.629576303	-0.14281187	-0.923755464
482494	483990	4	-	YDR018C	ORF-T	ORF-T	-0.382056808	0.419113079	1.95409654	1.112650638	1.336935387
485390	486542	4	-	GCV1, YDR02	other	other	0.743536058	0.046277433	-0.347474774	-0.392535768	0.301288064
487966	488942	4	-	CIS1	ORF-T	ORF-T	-0.688762327	-0.531934863	0.548055978	0.220623069	1.069251995
488942	489206	4	-	SUT467	SUTs	SUTs	3.788416962	-0.496844903	-0.683763541	-0.675229811	-0.869385707
492422	494406	4	-	YDR026C	ORF-T	ORF-T	0.409912186	-0.157916885	-0.661719464	-0.346677424	-0.189138426
495502	497430	4	-	VPS54	ORF-T	ORF-T	-0.120156881	-0.278368103	0.088280789	0.059339001	0.381917976
497534	501382	4	-	REG1	ORF-T	ORF-T	0.047720352	-0.487284519	-0.209989202	0.00366048	-0.555115762
501382	501662	4	-	unknown	no_ovlp2	SUTs	-1.568503906	-1.364083854	-0.124813164	-0.063044852	0.558062379
501662	503318	4	-	RAD28	ORF-T	ORF-T	-0.226671932	-0.434270994	0.877310181	-0.062994222	1.093621947
503943	504791	4	-	PST2	ORF-T	ORF-T	-0.635219619	-0.401336408	0.628708376	0.377352067	0.545118795
506535	506831	4	-	SUT468	SUTs	SUTs	1.475402674	-0.936521158	-0.43756028	-0.082177101	-0.595798471
506831	507159	4	-	SUT468	SUTs	SUTs	0.831054388	0.048994809	0.10553591	-0.521765644	-0.058662268
509631	512239	4	-	LYS14	ORF-T	ORF-T	0.270328357	-0.495408867	-0.352660129	0.071619176	-0.385434739
512239	512735	4	-	unknown	no_ovlp	CUTs	1.326421479	1.383357925	0.234055935	0.24271151	-2.033469563
523103	524775	4	-	EHD3	ORF-T	ORF-T	-0.300802822	0.185144238	0.199235683	-0.527163176	0.608845814
527263	538191	4	-	unknown	no_ovlp	SUTs	-0.406849674	-0.39837326	-0.18838415	0.076093363	0.410620006
538191	539535	4	-	unknown	no_ovlp	SUTs	0.318709033	0.885644583	1.185353208	0.532291757	0.412738763
541191	541695	4	-	YDR042C, SN	other	other	4.553993942	-0.857155826	-1.07569971	-0.696015503	-0.062534751
542558	543567	4	-	NRG1	ORF-T	ORF-T	0.379566361	-0.730163127	0.373238907	0.14752092	-0.275640483
544863	545831	4	-	SUT469	SUTs	SUTs	0.079296153	-0.288942195	-0.078505825	0.336153671	-0.887379068
547759	548431	4	-	RPC11	ORF-T	ORF-T	0.764681211	0.293349606	-0.107843739	-0.114262905	0.338950749
548631	550671	4	-	BAP3	ORF-T	ORF-T	0.040223879	-0.594253169	-0.957000568	-0.43102229	-0.475852001
550671	551623	4	-	unknown	no_ovlp	CUTs	1.222521007	0.531145495	-0.614987218	-0.52051794	-0.108134855
555639	556527	4	-	TPI1	ORF-T	ORF-T	-0.331925036	-0.397327894	-0.210977977	-0.058795028	-0.27128741
557007	558103	4	-	YDR051C	ORF-T	ORF-T	0.016109491	-0.320067191	0.159671354	0.065832174	0.363951895
558279	560767	4	-	DBF4	ORF-T	ORF-T	-0.122846786	-0.382877437	-0.189292034	-0.28761674	0.385249413
560967	562471	4	-	CDC34	ORF-T	ORF-T	0.292905195	-0.308509186	0.083860483	0.068598692	0.001900587
562471	562855	4	-	CUT498	CUTs	CUTs	2.303530057	0.160892163	0.514288624	0.326957044	-0.15856427
564919	565703	4	-	YDR056C	ORF-T	ORF-T	0.148103622	-0.663776276	-0.165661353	-0.045386182	0.038797445
567743	568727	4	-	TGL2	ORF-T	ORF-T	0.103285729	-0.570371187	0.499936166	-0.096465841	-1.054306002
569087	569631	4	-	UBC5	ORF-T	ORF-T	0.470606997	0.918483136	1.333756793	0.485673191	1.290175619
575023	575823	4	-	SUT470	SUTs	SUTs	0.795927126	-1.255081521	-1.054584495	0.290666013	0.2274265
578455	579103	4	-	CUT499	CUTs	CUTs	2.550390641	-0.251285798	-1.49388106	-1.240867939	-0.465284458
580567	581799	4	-	unknown	no_ovlp2	SUTs	0.611307916	0.133141029	-0.09309497	-0.083029653	0.363799309
581799	582711	4	-	YDR066C	ORF-T	ORF-T	-0.356831092	-0.722415075	-0.054273812	0.318211477	0.317752347
582711	583527	4	-	OCA6	ORF-T	ORF-T	0.040348596	-0.253493358	0.120737868	0.113949827	-0.036132545
584615	587767	4	-	DOA4	ORF-T	ORF-T	-0.584133358	-0.472709878	0.488577568	0.314694322	0.96230585
587967	588495	4	-	FMP16	ORF-T	ORF-T	-1.688893939	-1.177026523	2.524177149	1.968130097	-0.01450291
588727	589463	4	-	PAA1	ORF-T	ORF-T	0.036843693	-0.797516618	-0.392391724	-0.085252165	-0.271336837
589671	591511	4	-	IPT1	ORF-T	ORF-T	-0.072401283	-0.839859089	-0.85826827	-0.089415302	-0.333990551
592647	593455	4	-	SUT471	SUTs	SUTs	0.343008679	-0.516484625	0.58674581	0.357030479	0.044703799
598887	599599	4	-	SUT472	SUTs	SUTs	1.964669643	-0.529402765	-0.302634502	0.167851614	-0.315676982
599599	600271	4	-	SUT472	SUTs	SUTs	0.916997186	0.611197955	0.293554903	0.624853113	-0.125038293
600935	601679	4	-	unknown	no_ovlp	SUTs	-0.361623913	1.383410771	1.296617123	0.765004085	0.25925392
602087	602879	4	-	SHU2	ORF-T	ORF-T	1.304851941	0.095840251	-0.357598645	-0.551083556	-0.183850274
603383	603839	4	-	TFB5	ORF-T	ORF-T	0.423720193	-0.272728562	0.426614533	-0.241135463	-0.292512127
608655	610159	4	-	PDC2	ORF-T	ORF-T	0.287466811	-0.296538508	-0.164009918	-0.191122311	-0.209623222
613320	614064	4	-	TVP23	ORF-T	ORF-T	-0.449297755	-0.568112279	0.126758848	0.080053199	0.285585832
614192	616320	4	-	AFR1	ORF-T	ORF-T	0.397523228	-0.066569474	0.208866149	0.506635371	-0.219784077
616728	617256	4	-	SSS1	ORF-T	ORF-T	-0.37321761	-0.778241784	-0.068290551	-0.299078427	0.084078701
617408	618344	4	-	RRP1	ORF-T	ORF-T	0.433650806	-0.245271128	-0.389088984	0.257285309	0.597249134
618344	620048	4	-	SLU7, tR(ACG	other	other	-0.057788268	-0.430741881	-0.078816056	-0.177559463	-0.581255313
620904	621744	4	-	SUT473	SUTs	SUTs	1.774838198	-0.595947756	-0.258989143	-0.242666223	-1.029012521
624728	626088	4	-	YDR090C	ORF-T	ORF-T	-0.0711218	-0.457509783	-0.571388461	-0.343864477	-0.117609061
626368	628600	4	-	RLI1	ORF-T	ORF-T	-0.02300198	-0.682038947	-0.591477462	0.066835417	0.176447876
629688	630600	4	-	CUT503	CUTs	CUTs	2.846517373	0.134303991	-1.620083062	-0.194710422	-0.24586361
636216	636680	4	-	unknown	no_ovlp	CUTs	2.043554276	0.498027425	-0.123512793	-0.603157396	-0.809411476
640080	643840	4	-	MSH6	ORF-T	ORF-T	-0.252445213	-0.23211279	-0.291840853	-0.00645836	0.683493657
644128	644976	4	-	GRX3	ORF-T	ORF-T	-0.142320008	-0.212278733	-0.155425663	-0.129266938	-0.071768709
644976	651936	4	-	unknown	no_ovlp2	SUTs	0.067056444	0.29837391	-0.097710621	0.049521808	0.23722187
651936	655568	4	-	YDRCdelta9	other	other	0.220111382	-0.102406063	0.406497339	0.533755669	0.707734401
653120	655568	4	-	unknown	no_ovlp2	SUTs	-0.040886256	0.449175274	-0.014029478	0.019061468	0.153708183
655568	657520	4	-	ARX1	ORF-T	ORF-T	0.108904127	-0.531439181	-0.917761514	-0.068200023	0.259202361
661136	664904	4	-	SPO71	ORF-T	ORF-T	-0.13646059	-0.241800754	0.343896563	0.210846709	0.353665003
665256	666824	4	-	TMS1	ORF-T	ORF-T	-0.597424838	-0.730165453	0.124145154	0.205893696	0.000348815
669000	671032	4	-	TMN2	ORF-T	ORF-T	-1.472055792	-0.710952216	0.280428652	0.27668014	1.331330816
673440	675648	4	-	YDR109C	ORF-T	ORF-T	0.162604465	-0.382312244	-0.115454484	-0.201583427	-0.11366516
677968	680120	4	-	ALT2	ORF-T	ORF-T	0.08815422	0.023830163	-0.638459469	-0.419316868	-0.683587849
680472	681704	4	-	PDS1	ORF-T	ORF-T	-0.4160557	-0.381518773	0.196647313	-0.031577765	0.391113627
682624	683600	4	-	MRPL1	ORF-T	ORF-T	-0.634994205	-0.691893788	-0.117866316	-0.079831971	0.024193757
683960	685672	4	-	TMA64	ORF-T	ORF-T	-0.278770356	-0.422104116	-0.115864299	-0.158474136	0.263693372
687248	687864	4	-	CUT504	CUTs	CUTs	1.756102696	-0.484658508	-0.11517257	-0.012161295	0.80121852
690144	690488	4	-	CUT505	CUTs	CUTs	3.91788323	0.324716873	0.187678101	0.622004975	-1.219283086
691368	693232	4	-	TRM1	ORF-T	ORF-T	-4.88E-05	-0.247565718	-0.686199239	0.253878721	0.439468186

698472	699488	4 -	INO2	ORF-T	ORF-T	0.330155469	-0.12319802	-0.56899595	0.332297124	0.770673415
700176	702672	4 -	ECM18	ORF-T	ORF-T	0.184220012	-0.178477851	0.393833923	0.010757471	-1.843196891
703840	704176	4 -	CUT507	CUTs	CUTs	3.303112121	-1.290632861	-1.057970394	-0.602706507	-1.390328737
713200	715240	4 -	SAC6	ORF-T	ORF-T	-0.012788143	-0.609321278	-0.383304073	0.270297955	-0.396596551
715416	716768	4 -	unknown	no_ovlp2	SUTs	-0.524460992	-0.714233372	0.477181646	-0.208173312	0.423823256
716768	718488	4 -	YDR131C	ORF-T	ORF-T	0.65075041	-0.119410142	0.037957619	-0.481093026	-0.554295976
719016	720416	4 -	YDR132C	ORF-T	ORF-T	0.239335713	-0.523927492	-0.039569214	-0.273156991	-0.886683391
720792	721520	4 -	YDR133C, YD	other	other	-0.675780384	-0.638421558	0.364704685	0.310269623	0.745478675
722912	727592	4 -	YCF1	ORF-T	ORF-T	-0.771547883	-0.68473912	-0.102650578	0.178804467	0.17387119
727592	727968	4 -	unknown	no_ovlp	CUTs	1.990385977	0.009697291	-0.093310197	-0.463263125	-0.374321822
733440	733960	4 -	RUB1	ORF-T	ORF-T	-0.25398834	-0.680951545	-0.15285937	-0.275898764	0.023347307
734656	740024	4 -	DOP1	ORF-T	ORF-T	-0.140416319	-0.448296206	-0.191550186	-0.078283928	-0.251300732
740360	741736	4 -	PEX7	ORF-T	ORF-T	0.164049794	-0.440757506	-0.042048843	0.057910996	-0.104726953
741880	744032	4 -	SAN1	ORF-T	ORF-T	0.502876081	-0.126609245	-0.476452076	-0.311067758	-0.368030396
744232	746232	4 -	MKC7	ORF-T	ORF-T	-0.247420808	-0.420811868	-1.076606654	-0.522815567	0.336962438
749112	750848	4 -	SWI5	ORF-T	ORF-T	-0.107836985	-0.231666851	-0.055129851	-0.040880491	0.280717527
750848	751312	4 -	CUT508	CUTs	CUTs	2.632520807	0.333439051	-0.770070916	-1.019835489	-1.813692421
753392	755320	4 -	KGD2	ORF-T	ORF-T	-0.580468462	-0.396883567	1.008607166	0.351455778	0.394049188
764048	765328	4 -	CTH1	ORF-T	ORF-T	0.777027306	-0.934144447	-0.072820519	0.058049888	-0.66468023
766616	768104	4 -	ENT5	ORF-T	ORF-T	0.235643842	-0.280176505	0.009144938	0.082226509	-0.153987493
768104	768336	4 -	CUT509	CUTs	CUTs	1.719111947	0.131399875	-1.011071402	-0.535373474	-0.274985193
768400	769048	4 -	YDR154C, CP	ORF-T	ORF-T	-0.453474405	-0.200279891	0.154718711	0.063282558	0.195788739
769520	770256	4 -	CUT510	CUTs	CUTs	2.600595202	0.398054591	-0.823443634	-0.427899613	-0.373010683
780184	781224	4 -	NBP2	ORF-T	ORF-T	0.610778413	-0.585861132	-0.246288053	-0.229496254	-0.266191275
782024	784248	4 -	SEC1	ORF-T	ORF-T	-0.016294797	-0.388070204	-0.317135861	-0.326407706	-0.94427915
784384	784640	4 -	CUT513	CUTs	CUTs	3.646183507	0.402457216	-0.383407028	-0.241311918	-0.253100096
786208	789264	4 -	SEC5	ORF-T	ORF-T	-0.159143801	-0.467662507	0.025208033	-0.024389096	-0.179417168
789264	790136	4 -	unknown	no_ovlp	CUTs	1.836544136	0.280889125	-0.77097863	-0.144386824	0.229831119
792360	794248	4 -	STB3	ORF-T	ORF-T	0.052074647	-0.059964792	0.012919327	0.148296607	-0.177790329
795984	802432	4 -	SEC7	ORF-T	ORF-T	-0.364799363	-0.725885179	-0.572152147	0.073090167	-0.220490169
805976	806200	4 -	unknown	no_ovlp	CUTs	2.719958181	0.35674742	0.228311978	0.154211869	-1.01192641
810456	811688	4 -	ARG82	ORF-T	ORF-T	0.860405741	-0.785982013	-0.21578908	-0.378955477	0.192956825
813080	814192	4 -	RSM24	ORF-T	ORF-T	-0.632927064	-0.608420194	0.057510731	-0.002671542	-0.178800729
814192	815152	4 -	unknown	no_ovlp	SUTs	-0.010826488	0.334648708	-0.108000195	0.179205424	-0.142198379
818712	819176	4 -	CSN9	ORF-T	ORF-T	0.252412287	-0.43672164	0.380041609	-0.435788752	0.476877181
825753	827361	4 -	SAS4	ORF-T	ORF-T	-0.431773874	-0.613493892	0.14957531	-0.257041774	0.523355893
830417	831569	4 -	YDR183C-A, .	ORF-T	ORF-T	0.690722588	-0.674537325	-0.763270539	-0.34315008	0.066668791
831753	832577	4 -	YDR185C	ORF-T	ORF-T	0.706015249	-0.570608065	-0.110053092	-0.406496997	-0.20480125
832737	835601	4 -	YDR186C	ORF-T	ORF-T	0.042343768	-0.210240301	0.373270764	0.178209316	-0.035427504
835601	836049	4 -	unknown	no_ovlp	SUTs	0.262807838	0.427441985	0.020604683	0.625285126	0.419454981
840505	842049	4 -	RVB1	ORF-T	ORF-T	0.200558809	-0.574098844	-0.462887012	-0.022301199	-0.235915839
843489	844897	4 -	NUP42	ORF-T	ORF-T	-0.048423144	0.03519801	-0.115026654	-0.227357826	0.112310264
845841	847969	4 -	MSS116	ORF-T	ORF-T	-0.122961849	-0.330329517	-0.552943055	-0.29139016	-0.305842074
850185	851033	4 -	YDR196C	ORF-T	ORF-T	0.062138888	-0.813490445	0.088932592	-0.163733415	-0.114885087
852441	853993	4 -	RKM2	ORF-T	ORF-T	-0.022253375	-0.567635489	-0.24077066	-0.168234759	0.399230583
855609	856161	4 -	VPS64	ORF-T	ORF-T	-0.00455102	-0.27280324	-0.125673609	0.196554312	-0.120238817
856881	857977	4 -	RAV2	ORF-T	ORF-T	0.774994121	0.354598544	0.274157478	-0.229891333	-0.010044059
857977	859137	4 -	unknown	no_ovlp	CUTs	1.435659521	-0.412811369	-1.526227159	-0.472130683	-0.113037952
864985	867585	4 -	UME6	ORF-T	ORF-T	-0.057257623	-0.507103772	-0.063800514	-0.11077467	1.253237378
870193	870545	4 -	unknown	no_ovlp	CUTs	1.80341085	0.394876608	-1.366443666	-0.159253237	-0.822510361
886713	887065	4 -	unknown	no_ovlp	CUTs	1.924707481	0.070524223	-0.375165847	0.41634241	1.88868674
892025	892449	4 -	CUT515	CUTs	CUTs	4.19406537	-0.280016532	-0.158274231	-0.23785363	-0.277923378
892449	894065	4 -	unknown	no_ovlp	SUTs	-0.002829178	-0.021357859	0.398081207	0.854902875	1.256575957
899553	903489	4 -	RAD9	ORF-T	ORF-T	0.127151717	-0.108874234	-0.443066035	-0.325195222	0.041631512
905321	907025	4 -	MFB1, YDR2:	other	other	0.105314249	-0.843886315	-0.057767348	0.057686719	-0.1920938
908329	909401	4 -	SUT474	SUTs	SUTs	1.440043673	-0.610677481	-0.793136914	-0.362674222	0.417567383
911393	913745	4 -	SUT475	SUTs	SUTs	1.066763285	0.601959398	-0.033619707	-0.677306052	0.191758643
914193	914793	4 -	HTB1	ORF-T	ORF-T	-0.236162461	-0.294638969	0.037024802	-0.011814937	-0.142262519
915745	916297	4 -	SUT476	SUTs	SUTs	1.628109188	-0.11933735	-0.741537184	-0.915369902	-1.019115831
921913	923889	4 -	PCF11	ORF-T	ORF-T	0.309236984	-0.608803999	-0.244817459	0.067733122	0.084000523
923889	924537	4 -	SUT477	SUTs	SUTs	0.702972517	-0.674315994	-0.153091724	-0.575331478	-0.463421509
925833	926233	4 -	unknown	no_ovlp2	SUTs	0.861995915	0.686490603	0.204504213	0.358651072	0.936291798
926233	926913	4 -	COX20	ORF-T	ORF-T	-0.566078519	-0.284256333	1.150810281	0.374209129	0.512439207
926913	927513	4 -	unknown	no_ovlp	SUTs	-0.153901865	0.196560566	0.086411522	0.006312566	0.227456213
929337	930409	4 -	RTN1	ORF-T	ORF-T	-0.383750323	-0.224884837	-0.102972714	-0.075156829	-0.074098404
935129	935937	4 -	FMN1	ORF-T	ORF-T	-0.38837331	-0.603378293	-0.373782938	-0.469191663	-0.108924453
935937	936305	4 -	unknown	no_ovlp	SUTs	0.4094986	-0.082533286	0.056257596	-0.41943426	0.466094451
936305	937625	4 -	unknown	no_ovlp	SUTs	0.101542308	0.250748867	0.012829959	-0.021792673	1.20696301
937729	940841	4 -	SEC26	ORF-T	ORF-T	-0.468323427	-0.498901218	-0.189064112	-0.01171343	0.052017372
940985	943425	4 -	YDR239C	ORF-T	ORF-T	0.130555871	-0.174818649	-0.238623951	-0.3221019	-0.040992285
943649	945169	4 -	SNU56	ORF-T	ORF-T	0.171691975	-0.511388025	-0.604600857	-0.587304865	0.116939317
946737	950305	4 -	PRP28	ORF-T	ORF-T	0.317216943	-0.134858859	-0.312897467	-0.162439193	0.36570231
954145	954865	4 -	unknown	no_ovlp	CUTs	2.404826413	0.054220142	0.024849749	0.500829849	0.235380564
954865	955417	4 -	unknown	no_ovlp	SUTs	0.252557046	-0.418372408	-0.283699501	-0.240696851	-0.276257262
957665	958401	4 -	YDR248C	ORF-T	ORF-T	0.139994772	0.391633237	0.490505051	0.114509083	-0.160783942
958593	960081	4 -	YDR249C, YD	other	other	0.579535101	-0.58055077	-0.690977929	-0.512913918	-0.371269108
963801	964017	4 -	unknown	no_ovlp2	SUTs	0.16297979	0.132288106	-0.181607408	0.155325027	0.004659913
964017	965441	4 -	MET32	ORF-T	ORF-T	0.524676016	0.1212935	0.488348879	-0.117851578	0.571739238
964801	965441	4 -	unknown	no_ovlp2	SUTs	-0.014417277	0.307709582	0.297199836	0.04775505	0.102672269
966345	967857	4 -	RMD5	ORF-T	ORF-T	-0.168728419	-0.252276809	0.569197047	0.213586862	0.185339224
967857	969681	4 -	CTA1	ORF-T	ORF-T	0.424966661	-0.307758554	0.521447822	-0.026119461	-0.122451097
969681	971489	4 -	SET7	ORF-T	ORF-T	0.152139549	-0.891296503	-0.436254865	-0.364435853	-0.315634568

971705	974265	4	-	HSP78	ORF-T	ORF-T	-0.393494224	-0.598033278	0.597518695	0.364583145	-0.653304317
975121	975985	4	-	YAP6	ORF-T	ORF-T	0.733178768	-0.051011591	0.291433678	0.331723719	-0.574815837
976353	977401	4	-	SWM1	ORF-T	ORF-T	-0.020504808	-0.439972263	-0.562990316	-0.415971039	-0.71789509
977401	979297	4	-	EXG2	ORF-T	ORF-T	0.591633413	-0.66991593	-0.380015437	-0.251496433	-0.296805822
980385	981057	4	-	unknown	no_ovlp	SUTs	-0.302055211	-1.108665688	0.26431047	0.134810167	-1.353965359
994033	995761	4	-	DIN7	ORF-T	ORF-T	0.103377925	-0.286068713	0.130962454	-0.416232598	0.375423226
995761	998377	4	-	AKR1	ORF-T	ORF-T	-0.364228882	-0.601804937	-0.204029606	0.148657499	-0.034003844
1000041	1002225	4	-	YDR266C	ORF-T	ORF-T	0.077824919	-0.57805279	-0.566319325	0.037248211	-0.124000678
1002345	1003553	4	-	CIA1	ORF-T	ORF-T	0.172370001	-0.313710209	-0.303646371	0.067077198	0.028326622
1004425	1005329	4	-	CUT516	CUTs	CUTs	1.499857123	-0.563465519	-1.020309972	0.319325481	0.124725632
1007321	1008537	4	-	unknown	no_ovlp	CUTs	1.039296891	0.323985459	-0.345806488	-0.941136126	0.662133308
1010921	1011489	4	-	CUT517	CUTs	CUTs	2.159301151	0.401485704	-0.300984252	-0.13103442	-0.415107758
1013233	1013745	4	-	PMP3	ORF-T	ORF-T	-0.364620426	-0.871148236	0.052813358	-0.158413473	0.17864234
1014193	1015881	4	-	MTH1	ORF-T	ORF-T	-0.172090781	-0.965954782	0.577696043	0.811702984	-0.485096605
1018057	1019097	4	-	SUT480	SUTs	SUTs	0.904002466	-0.219917081	-0.679512863	-0.107631922	-0.316013314
1019097	1021737	4	-	unknown	no_ovlp2	SUTs	0.214995443	0.430266169	-0.045788323	0.062555108	0.039569603
1021737	1023505	4	-	PHM6	ORF-T	ORF-T	-0.212736837	2.427793566	1.389076165	1.966748687	0.776859259
1022425	1023505	4	-	unknown	no_ovlp2	SUTs	-0.137848789	0.446621564	0.097915828	0.014749719	0.032284339
1023505	1024753	4	-	YDR282C	ORF-T	ORF-T	-0.124100454	0.040151191	0.475008062	0.259336072	-0.202896796
1024929	1030265	4	-	GCN2	ORF-T	ORF-T	0.039202265	-0.441650443	-0.249725307	-0.170314389	-0.177718587
1030465	1031697	4	-	DPP1	ORF-T	ORF-T	-0.226993757	-0.355239991	0.085570345	-0.014779769	0.419474369
1031697	1032025	4	-	CUT519	CUTs	CUTs	2.391849871	0.663005664	0.13190639	-0.061967032	0.340954367
1035153	1035569	4	-	YDR286C	ORF-T	ORF-T	-0.84997998	-1.303984915	0.513574889	0.046680114	0.240610489
1036633	1037001	4	-	CUT520	CUTs	CUTs	2.072247822	-0.42473454	-0.609114115	0.104021522	0.353153831
1038177	1039513	4	-	RTT103	ORF-T	ORF-T	0.211027847	-0.642959178	-0.178674722	0.100409449	-0.295113718
1043097	1045145	4	-	SRP101	ORF-T	ORF-T	-0.164962501	-0.396560718	-0.364723414	0.008341538	-0.248698729
1045377	1050241	4	-	SSD1	ORF-T	ORF-T	0.249569128	-0.53636343	-0.146779113	-0.141727075	-0.456643543
1050401	1052265	4	-	DPL1	ORF-T	ORF-T	0.214594264	-0.111985431	-0.112403648	0.354758207	-0.214732889
1052601	1054897	4	-	HDA2	ORF-T	ORF-T	0.065691686	-0.663545869	-0.20201541	-3.657149882	-0.526279893
1055417	1056113	4	-	unknown	no_ovlp	CUTs	2.445861164	0.673422307	-1.237002026	-0.04094862	-0.024299549
1058001	1058985	4	-	ATP5	ORF-T	ORF-T	-0.205957321	-0.269391271	0.465699283	-0.062310353	0.032323007
1061401	1062833	4	-	PRO1	ORF-T	ORF-T	0.192374161	-0.185537468	-0.446513034	0.024628454	0.061239451
1069633	1071681	4	-	RSC3	ORF-T	ORF-T	0.090772564	-0.594876444	-0.930525819	-0.199379076	-0.125144121
1071809	1072577	4	-	CPR5	ORF-T	ORF-T	-0.135736949	-0.546673953	-0.180707402	-0.198311113	-0.314036575
1072577	1073665	4	-	unknown	no_ovlp2	SUTs	0.006100328	-0.319338966	0.203745859	0.154964789	0.210366014
1073665	1075217	4	-	YDR306C	ORF-T	ORF-T	-0.620834622	-0.520584206	0.595392761	0.408218426	0.153025061
1077889	1078473	4	-	SRB7	ORF-T	ORF-T	0.153221059	-1.174553147	-0.419143938	-0.09524598	-0.393122035
1078785	1080273	4	-	GIC2	ORF-T	ORF-T	0.549003559	-0.373875687	0.042392088	-0.263175533	-0.586302869
1080929	1084601	4	-	SUM1	ORF-T	ORF-T	-0.226902092	-0.425946825	-0.636641134	-0.081293356	-0.414052419
1089041	1090089	4	-	PIB1	ORF-T	ORF-T	0.126832892	-0.275840108	0.5834069	0.157309566	0.749682931
1090089	1093585	4	-	RAD34	ORF-T	ORF-T	-0.080583302	-0.722890106	0.115320249	0.23361102	-0.19656154
1092553	1093585	4	-	unknown	no_ovlp2	SUTs	0.154341074	0.152331758	0.799136937	-0.7291414	0.840715672
1102305	1103601	4	-	SUT481	SUTs	SUTs	0.47550362	-0.746577873	-0.682735265	-0.169749279	-0.365064402
1104905	1105865	4	-	YDR319C	ORF-T	ORF-T	0.265004447	-0.647698595	-0.262106103	0.193093039	-0.511478312
1106009	1108217	4	-	SWA2	ORF-T	ORF-T	-0.426557512	-0.330457985	-0.168325527	-0.243628848	-0.277801355
1108217	1108505	4	-	DAD4	ORF-T	ORF-T	-0.560662133	-0.984667535	0.526394085	-0.270985683	0.63256748
1111785	1112329	4	-	TIM11	ORF-T	ORF-T	-0.255937799	-0.871456339	0.532319935	0.112496632	0.040263765
1112329	1114049	4	-	PEP7	ORF-T	ORF-T	-0.360464336	-0.568762189	0.085707371	-0.434947518	0.121856565
1114233	1116705	4	-	UTP4	ORF-T	ORF-T	0.475022724	-0.549192208	-0.956293372	-0.215974029	0.040833764
1120481	1125105	4	-	YSP2	ORF-T	ORF-T	-0.121919956	-0.211314192	-0.399467702	0.084933213	0.256076083
1125217	1126073	4	-	SKP1	ORF-T	ORF-T	-0.071795172	-0.258827959	-0.024738688	0.115505006	0.136765826
1126193	1127617	4	-	PEX3	ORF-T	ORF-T	-0.0723391	-0.419403005	0.415604954	0.138713599	0.443687158
1130897	1131201	4	-	SUT482	SUTs	SUTs	0.916331569	-0.378700709	0.979379603	0.141560021	-0.770422096
1133065	1135441	4	-	YDR333C	ORF-T	ORF-T	0.212117446	-0.427854137	-0.647135111	-0.063461273	-0.123157556
1139537	1140513	4	-	unknown	no_ovlp	SUTs	0.885630616	0.418772244	-0.266257637	0.856035466	2.225400816
1145809	1146049	4	-	CUT522	CUTs	CUTs	2.396139364	-0.031311703	-0.376155477	0.075021406	0.1331252
1147209	1149497	4	-	YDR338C	ORF-T	ORF-T	-0.149442476	-0.256551005	0.015456125	0.227022478	-0.090550313
1149841	1150545	4	-	FCF1	ORF-T	ORF-T	0.336730608	-0.479781709	-0.202999366	0.120641527	-0.116251284
1150545	1150785	4	-	unknown	no_ovlp	SUTs	0.728426452	0.379250631	-0.330324607	-0.001250688	-0.494342323
1151641	1153641	4	-	YDR341C	ORF-T	ORF-T	0.002144165	-0.763705651	-0.664039959	0.062947993	-0.503879611
1154057	1156009	4	-	unknown	no_ovlp	SUTs	-1.774474789	-2.491824125	1.13041749	1.342150369	-0.188312335
1162809	1164681	4	-	HXT3	ORF-T	ORF-T	-0.222579708	-0.501840503	-0.112518415	-0.061362917	-0.56738569
1164681	1166153	4	-	unknown	no_ovlp	CUTs	1.449617318	0.315218033	-0.095095647	0.257159409	-0.774454283
1166905	1168809	4	-	SVF1	ORF-T	ORF-T	-0.321601979	-0.727660738	-0.03177102	0.162618662	0.274516185
1170177	1172017	4	-	YDR348C	ORF-T	ORF-T	-0.446202404	-0.477769541	-0.248817551	-0.091534907	0.09435227
1172273	1174265	4	-	YPS7	ORF-T	ORF-T	0.251620473	-0.119614749	-0.256465904	-0.341193251	0.191899404
1174265	1174857	4	-	SUT483	SUTs	SUTs	1.804292715	0.321868432	-0.712147395	-0.214372497	-2.297150035
1176049	1178201	4	-	ATP22	ORF-T	ORF-T	0.108375094	-0.631300371	-0.063077493	-0.375251461	-0.318659082
1182385	1183513	4	-	CUT524	CUTs	CUTs	0.044537455	-1.244890114	-0.330426713	0.020131222	-0.371763102
1183513	1184241	4	-	unknown	no_ovlp	CUTs	1.232897797	-0.060930822	-1.195383418	-0.347218316	-0.443681024
1189129	1189665	4	-	YDR357C	ORF-T	ORF-T	-0.208831479	-0.651763258	0.87547977	0.599334015	-1.282127364
1191913	1195161	4	-	EAF1	ORF-T	ORF-T	-0.867473708	-0.500940185	-0.156316233	-0.35033468	-0.877068703
1195161	1196289	4	-	BCP1	ORF-T	ORF-T	0.814760046	-0.138283712	-0.683244057	-0.20337185	0.169330592
1196625	1198801	4	-	TFC6	ORF-T	ORF-T	-0.374330957	-0.852866492	-0.241841746	-0.238054889	0.419253304
1202465	1204201	4	-	CDC40	ORF-T	ORF-T	0.404968142	-0.205671843	-0.41358383	-0.2164198	0.009243183
1204569	1206441	4	-	ESF1	ORF-T	ORF-T	0.005258355	-0.336847879	-0.604691375	0.231322284	0.418184559
1213953	1215017	4	-	unknown	no_ovlp2	CUTs	1.185938102	0.495030999	-0.866161808	-0.160707794	0.458186825
1215017	1217577	4	-	XRS2	ORF-T	ORF-T	0.096314497	-0.054883589	-0.176724071	-0.449130959	0.0204031
1217713	1219089	4	-	YDR370C	ORF-T	ORF-T	-0.088181176	-0.831402587	-0.451221675	-0.179701663	-0.069350569
1220929	1222457	4	-	VPS74	ORF-T	ORF-T	-0.363575585	-0.809527674	-0.215556687	0.223150896	0.062322141
1225137	1226537	4	-	BCS1	ORF-T	ORF-T	-0.731199361	-0.567991667	-0.012901672	-0.087226452	0.359925881
1227449	1228297	4	-	BCS1	ORF-T	ORF-T	1.370809497	-0.25146915	-0.607188192	0.624605981	0.861483874

1229129	1229617	4	-	LSM6	ORF-T	ORF-T	0.005457237	-0.265679392	-0.387650103	-0.220551296	0.013474493
1233105	1233681	4	-	YDR379C-A	ORF-T	ORF-T	0.970256437	-1.079281234	0.233766772	-0.384517796	0.418840949
1233681	1236337	4	-	SUT485	SUTs	SUTs	-0.630014272	-0.51984529	0.112372801	0.038222243	-0.120837288
1238137	1238801	4	-	YDR381C-A	ORF-T	ORF-T	0.991123794	0.909314881	1.181609646	0.460204736	-0.412384172
1239961	1240641	4	-	NKP1	ORF-T	ORF-T	0.482798425	-0.276551195	-0.321669067	-0.399300231	-0.426381067
1241033	1242105	4	-	ATO3	ORF-T	ORF-T	-0.03439955	-1.423365839	-0.254055992	0.236424696	0.195997089
1242105	1242601	4	-	unknown	no_ovlp	SUTs	-0.278595644	-0.448220773	0.046100124	-0.364806961	-0.629104752
1248025	1249937	4	-	YDR387C	ORF-T	ORF-T	-0.140691807	-0.953520789	-0.211698244	-0.012743713	-0.154003614
1251057	1251641	4	-	SUT486	SUTs	SUTs	0.664171579	0.438401655	-0.614607746	0.342697476	0.927485196
1254857	1256865	4	-	UBA2	ORF-T	ORF-T	0.118314382	-0.514907526	0.101767267	-0.122160677	0.299307122
1258057	1258481	4	-	YDR391C	ORF-T	ORF-T	0.746807695	-0.531325186	-0.073292959	-0.182755164	-0.068963757
1260465	1261369	4	-	SUT488	SUTs	SUTs	1.081937765	-0.703787445	-0.288747353	-0.129981921	0.313100498
1266193	1266969	4	-	NCB2	ORF-T	ORF-T	-0.213680008	-0.72543592	0.049663317	0.104329059	0.098813962
1268881	1269673	4	-	CUT528	CUTs	CUTs	2.204328805	0.324935903	-0.179091103	-0.251359801	-0.145190027
1276505	1277361	4	-	RPB7	ORF-T	ORF-T	-0.176425685	-0.402467075	-0.338743928	-0.192121384	0.092991225
1277905	1279929	4	-	SUT489	SUTs	SUTs	1.215837544	0.05849608	0.38639518	-0.073570037	0.222217687
1284057	1288089	4	-	TRS120	ORF-T	ORF-T	-0.247299222	-0.259870842	-0.299817177	-0.150336563	0.333403621
1288089	1288913	4	-	ADE8	ORF-T	ORF-T	0.398094045	-0.153749625	-0.251280906	-0.104766742	-0.927372501
1288913	1289857	4	-	unknown	no_ovlp	SUTs	-0.018358516	0.472478806	0.043612083	-0.074340449	0.125083182
1292289	1293129	4	-	STE14	ORF-T	ORF-T	0.014073478	-0.678840532	-0.796238523	-0.476290803	-0.296021141
1293305	1294433	4	-	DFM1	ORF-T	ORF-T	-0.655594269	-0.464825059	0.022586901	-0.144744956	-0.083740367
1295425	1298177	4	-	YDR413C, ER	other	other	0.037576839	-0.578848326	-1.167718338	-0.232109812	-0.342386549
1296865	1298177	4	-	unknown	no_ovlp2	SUTs	0.331530872	-0.247143492	-0.350201583	-0.448098661	-0.185323097
1300305	1300969	4	-	CUT532	CUTs	CUTs	3.610863014	-0.044637468	-1.340478378	-0.440429124	0.114436899
1300969	1304193	4	-	unknown	no_ovlp	SUTs	0.063582027	0.285560589	-0.270154676	-0.18500291	0.112634907
1311689	1312553	4	-	unknown	no_ovlp	CUTs	1.127409673	0.141160113	-1.00135835	-1.103817279	0.575456221
1315009	1317841	4	-	SIP1	ORF-T	ORF-T	0.127832527	-0.188797794	-0.207140496	-0.038044005	0.162210559
1317977	1319233	4	-	CAD1	ORF-T	ORF-T	-0.482442267	-0.881079314	-0.199734205	-0.150466659	-0.446917762
1319353	1319609	4	-	DYN2	ORF-T	ORF-T	0.098548965	-0.005883704	0.669805468	0.099890982	0.03503588
1321393	1322017	4	-	unknown	no_ovlp	CUTs	3.955286759	0.266456281	-1.035066431	-0.51161419	0.294913561
1323321	1324377	4	-	YDR428C	ORF-T	ORF-T	-0.80726635	0.196645971	0.257398604	0.082725887	1.141091158
1324377	1325313	4	-	TIF35	ORF-T	ORF-T	-0.051897529	-0.579221062	-0.553578872	-0.111795273	-0.168300921
1325473	1328537	4	-	CYM1	ORF-T	ORF-T	-0.647440027	-0.455062106	-0.184236525	-0.365332869	-0.078138504
1328833	1329137	4	-	unknown	no_ovlp	SUTs	-1.079520294	-0.858554109	0.773571412	1.09510369	-0.349376343
1332793	1333969	4	-	PPM1	ORF-T	ORF-T	0.374667872	0.099642112	0.348362469	0.011689193	0.197234477
1340761	1342177	4	-	SUT492	SUTs	SUTs	0.284110245	-0.331013676	-0.550072973	-0.541879086	-0.857241185
1344249	1345209	4	-	APT2	ORF-T	ORF-T	0.091656786	-0.806679372	-0.483931856	-0.015135567	-0.272532452
1345209	1349969	4	-	SSN2	ORF-T	ORF-T	-0.255786845	-0.275560731	0.287778694	-0.096367674	0.366617914
1353041	1353545	4	-	SUT493	SUTs	SUTs	0.334224336	0.007963053	0.244132848	0.428940178	-0.076695335
1353545	1354593	4	-	unknown	no_ovlp	SUTs	-0.328473918	0.374006837	0.181823593	0.052207011	0.547592083
1355233	1355601	4	-	RPS17B	ORF-T	ORF-T	1.065040529	-0.755345785	-0.656312667	-0.719824198	-0.301492891
1357537	1358929	4	-	UTP6	ORF-T	ORF-T	0.386724886	-0.070495408	-0.484323068	-0.513185319	0.571618136
1360929	1362817	4	-	YHP1	ORF-T	ORF-T	-0.176995564	-0.172626874	-0.927206523	-0.515090804	0.587465921
1362225	1362817	4	-	unknown	no_ovlp2	CUTs	2.588274786	0.420524331	-1.545559308	-0.687615427	-0.34510884
1362817	1364505	4	-	unknown	no_ovlp	CUTs	2.741040522	0.921090069	-1.144782806	-0.536139351	-0.027962731
1365009	1365385	4	-	TSA2	ORF-T	ORF-T	-1.024577499	-0.702187035	1.744577721	1.343617342	0.771737488
1366137	1366841	4	-	GUK1	ORF-T	ORF-T	0.235116905	-0.291784143	-0.745240755	-0.089888918	-0.290932877
1379825	1382065	4	-	HEH2	ORF-T	ORF-T	0.599600175	-0.057149392	-0.512312577	-0.062796419	-0.589608699
1382209	1383521	4	-	PFA5	ORF-T	ORF-T	-0.227400757	-0.478594146	0.162037402	-0.273337626	0.590865569
1384001	1384801	4	-	CUT534	CUTs	CUTs	2.380744747	0.098463725	-1.712293119	0.431158571	-0.28513746
1385425	1385769	4	-	YDR461C-A	ORF-T	ORF-T	-0.046415547	0.726152084	1.523648777	0.436832057	1.270880631
1393177	1394625	4	-	RMT2	ORF-T	ORF-T	0.453621588	-0.187556511	-0.442377264	-0.025949885	0.416654562
1397913	1398841	4	-	TLG1	ORF-T	ORF-T	0.121089216	-0.481797951	-0.169162549	-0.262989735	0.000689851
1399585	1401281	4	-	UGO1	ORF-T	ORF-T	-1.092849687	-0.633359661	0.373827786	-0.077012079	0.822392605
1404361	1405889	4	-	PRP3	ORF-T	ORF-T	0.0752988	-0.396912908	0.096662749	-0.350047595	-0.095323539
1407353	1410161	4	-	JIP4	ORF-T	ORF-T	-0.156367094	-0.164607266	0.438081434	0.346014274	-0.122446804
1410249	1411145	4	-	YDR476C	ORF-T	ORF-T	-0.063925999	0.48296276	0.254671986	0.053300454	-0.375957427
1411145	1412073	4	-	unknown	no_ovlp	SUTs	0.031736273	-0.269948182	0.774550175	0.426774561	0.4572128
1415105	1416921	4	-	PEX29	ORF-T	ORF-T	-0.525356191	-0.709380568	0.372669108	0.331841764	-0.034033853
1417329	1418225	4	-	unknown	no_ovlp	CUTs	1.520128576	0.729242698	-0.570530128	0.039410056	1.03344406
1418441	1420121	4	-	PHO8	ORF-T	ORF-T	-0.032563553	0.543072778	0.375450557	0.728632322	0.875510676
1420121	1420353	4	-	unknown	no_ovlp2	SUTs	-0.879818683	-0.170246511	0.373728553	0.426293335	0.275018517
1420353	1420889	4	-	CWC21	ORF-T	ORF-T	0.019111077	-0.435763782	-0.416513444	-0.358552755	-0.154945229
1424665	1427457	4	-	VPS72	ORF-T	ORF-T	0.066361655	-0.61332244	-0.152375043	0.005283179	0.100675116
1427217	1427457	4	-	unknown	no_ovlp2	SUTs	-0.103129565	-0.533096211	0.719675438	-0.188237966	-0.165709172
1427457	1428137	4	-	VPS60	ORF-T	ORF-T	0.033044755	-0.403547123	0.507556703	-0.204025939	0.355625482
1428297	1429017	4	-	RIB3	ORF-T	ORF-T	0.267328773	-0.644379026	-0.476478015	-0.099083222	-0.307277458
1429017	1430737	4	-	PAC11	ORF-T	ORF-T	0.062485557	0.349612952	-0.180549863	-0.193039776	1.326909142
1431801	1434337	4	-	PKH1	ORF-T	ORF-T	-0.060568703	-0.431347609	0.291509232	0.049467964	0.794847099
1436161	1436689	4	-	CUT536	CUTs	CUTs	2.476917695	0.241470713	-0.608758151	-0.093181198	0.044403287
1437953	1441337	4	-	VPS3	ORF-T	ORF-T	-0.407344059	-0.57982252	-0.338901788	0.149496728	-0.00533974
1441337	1443425	4	-	PUF6	ORF-T	ORF-T	0.666272882	-0.289824169	-0.516396745	-0.031645506	0.094592028
1443665	1445553	4	-	ITR1	ORF-T	ORF-T	-0.516955727	-0.281613764	-0.728308737	0.074385853	-0.367946442
1445833	1447025	4	-	SEC20	ORF-T	ORF-T	-0.269462152	-0.138962975	0.245636427	0.044036307	0.660511071
1450457	1450865	4	-	RPL37B	ORF-T	ORF-T	0.895244584	-1.304411058	-0.835481137	-1.093999233	-0.319961118
1453001	1454505	4	-	SAM2	ORF-T	ORF-T	-0.678804346	-0.59280664	-1.289906666	-0.268196201	-0.011995283
1454945	1456081	4	-	LPP1	ORF-T	ORF-T	-0.509606858	0.347560034	0.845293584	-0.083697602	0.847344297
1456697	1459401	4	-	SPG3, PSP1	ORF-T	ORF-T	-0.026067248	-0.648556305	-0.046302906	-0.102365597	-0.067014911
1459401	1459633	4	-	unknown	no_ovlp2	SUTs	-0.697219341	-0.380999954	0.569305454	0.58471494	0.721722345
1459633	1461561	4	-	YDR506C	ORF-T	ORF-T	-0.768950515	-0.399722217	0.5824147	0.583952108	1.035129363
1462177	1465897	4	-	GIN4	ORF-T	ORF-T	-0.159414666	-0.1638761	-0.172652237	-0.096161914	0.627664753
1466233	1468489	4	-	GNP1	ORF-T	ORF-T	-0.135814506	-0.907827687	-0.824704522	0.057764092	-0.310228185

1470393	1470793	4	-	EMI1	ORF-T	ORF-T	-0.273729409	-0.615356561	0.774093213	-0.406328804	0.275917946
1471457	1473049	4	-	YDR514C	ORF-T	ORF-T	0.357880988	-0.126352411	-0.985610933	-0.57015476	0.111572177
1474857	1476577	4	-	EMI2	ORF-T	ORF-T	-0.202500701	-0.727691693	0.628993541	0.566232097	-1.671257339
1476577	1476897	4	-	unknown	no_ovlp	SUTs	0.107376386	-0.570253476	0.498534259	-0.358719027	0.078947094
1477825	1478337	4	-	unknown	no_ovlp	CUTs	1.654230655	0.961349617	-0.8753926	-0.050088312	0.691693497
1480953	1483497	4	-	YDR520C	ORF-T	ORF-T	-0.200984553	-0.149034438	0.057107392	-0.257318394	-0.059034769
1485497	1487249	4	-	SPS1	ORF-T	ORF-T	1.104994264	-0.07885289	-0.791524753	-0.971062945	-0.611656655
1487025	1487249	4	-	unknown	no_ovlp2	CUTs	1.122886138	-0.502344937	-1.329281651	-1.161234819	-1.692981731
1487249	1489049	4	-	AGE1	ORF-T	ORF-T	0.192580782	-0.764717592	-0.322493556	-0.290750834	-0.276250266
1489321	1489833	4	-	YDR524C-B,	other	other	-0.57095762	-0.291176159	0.00678942	0.08682195	0.038870389
1492457	1493017	4	-	SNR84	other	other	0.904513558	-0.548751519	-0.488834556	-0.18366917	-0.077793604
1495985	1496569	4	-	QCR7	ORF-T	ORF-T	0.042193449	-0.240097544	0.505341251	0.167271475	0.100025543
1496569	1497889	4	-	APA2	ORF-T	ORF-T	-0.094982299	-0.828629208	-0.036804299	0.160412514	-0.181854858
1499361	1500545	4	-	YDR532C	ORF-T	ORF-T	0.625963263	-0.342671018	-0.532375856	-0.650787772	-1.365393184
1500545	1501145	4	-	unknown	no_ovlp	SUTs	-0.157017297	-0.12598631	-0.364213805	-0.229942017	-0.571338014
1501345	1502241	4	-	HSP31	ORF-T	ORF-T	-0.712572215	-1.127205366	0.418874119	-0.465167026	-3.3819639
1503369	1508473	4	-	FIT1, YDR535	other	other	-0.016141507	0.291443643	0.248449513	0.663300515	-1.762084944
1507073	1508473	4	-	unknown	no_ovlp2	SUTs	0.538076649	-0.010086476	-0.072823847	0.038888514	-1.112140415
1508473	1509817	4	-	SUT498	SUTs	SUTs	0.620454819	-0.649016642	-0.037751363	0.164586041	-1.653894927
1515761	1516865	4	-	unknown	no_ovlp2	SUTs	0.349695181	-0.233795993	-0.042068214	0.01091167	-2.179497632
1516865	1517737	4	-	IRC4	ORF-T	ORF-T	-0.969150264	-1.451598029	0.050068959	0.290755417	-2.361269922
1519473	1520913	4	-	YDR541C	ORF-T	ORF-T	0.204230872	-0.104361936	-0.489259416	-1.127220736	-3.62376234
20337	21273	4	+	YDL241W	ORF-T	ORF-T	0.471677447	0.037901101	0.171882398	0.636451231	-1.091328335
22657	26161	4	+	LRG1	ORF-T	ORF-T	-0.128540003	-0.613665837	-1.075405045	-0.253465383	-0.479924602
30641	32025	4	+	YDL237W	ORF-T	ORF-T	-0.460844453	0.047499367	0.457346495	-0.221473471	0.03979631
32265	33409	4	+	PHO13	ORF-T	ORF-T	0.101326825	-0.508366353	-0.515284035	0.035098814	-0.101489108
36793	38337	4	+	YDL233W	ORF-T	ORF-T	-0.222171561	-0.301264728	0.188625013	0.559888862	-0.063603038
38473	38889	4	+	OST4	ORF-T	ORF-T	-0.707280933	-0.902780646	-0.144636772	0.132865053	0.186787667
42681	43745	4	+	PTP1	ORF-T	ORF-T	-0.682350073	-0.258213954	0.930502757	0.238501369	1.302898105
44049	46009	4	+	SSB1	ORF-T	ORF-T	-0.147126577	-0.231325125	-0.338233422	-0.188217713	-0.429046805
50505	50945	4	+	unknown	no_ovlp	SUTs	-0.074016513	-1.205895712	-1.111264042	0.807925429	0.19765128
52409	54193	4	+	SHS1	ORF-T	ORF-T	-0.004791441	-0.524349912	-0.248285943	-0.048531188	0.013822536
57033	57513	4	+	CUT049	CUTs	CUTs	2.035667546	0.407800451	-0.575779821	0.624398556	-0.504847893
59161	60593	4	+	SUT041	SUTs	SUTs	1.774435567	-0.018457349	-2.004232035	-2.303717099	-0.338932792
65393	66073	4	+	DTD1	ORF-T	ORF-T	-0.332076118	-0.703391222	0.134009613	-0.368783391	-0.224447006
68985	69977	4	+	unknown	no_ovlp	CUTs	2.894263315	0.083988487	-2.45916258	-0.823173676	-1.322302054
73585	75201	4	+	CUT050	CUTs	CUTs	2.117951997	0.474280771	-0.581348026	-0.014393609	-2.174936477
78377	79217	4	+	SHR3	ORF-T	ORF-T	-0.079995308	-0.33598689	-0.16394283	0.09174638	0.002491718
80713	83417	4	+	SUT042	SUTs	SUTs	0.337382361	-0.139703857	0.002269673	-0.255625829	-0.350249111
84025	84537	4	+	unknown	no_ovlp2	SUTs	0.066190763	-0.489714593	-0.561049579	0.239984396	-1.861763703
84537	86081	4	+	UGA4	ORF-T	ORF-T	-0.146127374	-0.397530472	-0.330261259	-0.055034756	-2.749666164
87473	88121	4	+	NHP2	ORF-T	ORF-T	-0.095362712	-0.894380044	-0.900702987	-0.353570425	-0.545308053
88217	89945	4	+	GLE1	ORF-T	ORF-T	0.027263748	-0.201197494	-0.264155355	-0.094412229	-0.024393458
90025	92489	4	+	YDL206W	ORF-T	ORF-T	0.198792965	-0.44651016	-0.18637893	0.085744355	-0.384327799
94121	94569	4	+	RTN2	ORF-T	ORF-T	0.862596701	-0.694682371	-0.311784695	-0.381678175	-0.118347062
94569	95769	4	+	RTN2	ORF-T	ORF-T	-0.652620389	-0.62156968	2.349802257	1.343908498	1.257043342
98433	99297	4	+	MRPL11	ORF-T	ORF-T	-0.818398098	-0.503098064	0.090660687	-0.148373915	0.064686339
99513	100497	4	+	TRM8	ORF-T	ORF-T	0.404279979	-0.45410963	-1.068725198	-0.262348959	-0.146954744
104945	105457	4	+	unknown	no_ovlp	CUTs	1.889972345	0.111098593	-0.005956601	-0.360632539	0.833046404
106705	107097	4	+	unknown	no_ovlp	CUTs	1.064722972	0.854796715	0.625714127	-0.61295822	0.317449187
107097	111169	4	+	SEC31	ORF-T	ORF-T	-0.52963437	-0.39098279	-0.390128373	0.00726363	-0.344544268
111385	114289	4	+	SNF3	ORF-T	ORF-T	-0.332651049	-0.619600099	0.672151224	0.652027234	0.35149842
114577	115969	4	+	NUS1	ORF-T	ORF-T	-0.416684042	-0.171563689	-0.0056557	0.204455959	0.456178413
116241	117105	4	+	ARF1	ORF-T	ORF-T	-0.392886212	-1.017003789	-0.798640928	-0.41783522	-0.588464934
117665	118161	4	+	RPL35A	ORF-T	ORF-T	0.251153788	-0.444378465	-0.898537378	-0.864255948	0.419194046
122001	123697	4	+	RBS1	ORF-T	ORF-T	0.087155094	-0.711664861	-0.877776254	-0.289246377	-0.75758202
126689	130097	4	+	TFP1	ORF-T	ORF-T	-0.332847595	-0.152519709	-0.275320847	0.145852904	-0.124201687
130177	132137	4	+	unknown	no_ovlp	SUTs	0.021142058	0.399391595	-0.116223571	-0.127650264	0.245465473
132137	133417	4	+	unknown	no_ovlp	SUTs	0.619956933	0.280221147	0.325784454	-0.133332943	0.965277964
133417	134793	4	+	LYS20	ORF-T	ORF-T	-0.604093794	-1.252503815	-0.454181279	0.401452637	0.313984487
135089	135553	4	+	INH1	ORF-T	ORF-T	-0.784892735	-0.409599913	0.860304405	0.011515566	0.430543761
135793	137585	4	+	YDL180W	ORF-T	ORF-T	-0.498358089	-0.863713932	-0.487941936	-0.486055486	-0.169296064
138129	139345	4	+	PCL9	ORF-T	ORF-T	-0.429151681	-0.482680628	-0.528905141	-0.231858365	-0.348737217
139513	141193	4	+	DLD2	ORF-T	ORF-T	0.49172822	-0.234124236	-0.260753112	0.14254427	-0.631249821
141969	144369	4	+	YDL176W	ORF-T	ORF-T	-0.070850249	-0.389866812	-0.26343823	-0.094056705	0.348309973
147497	148153	4	+	unknown	no_ovlp	SUTs	0.171945039	0.277074659	-0.203047562	0.034157419	-0.022590829
148153	149185	4	+	YDL173W	ORF-T	ORF-T	0.019024294	-0.203923337	-0.2098602	0.059596915	-0.177057806
156321	158057	4	+	UGA3	ORF-T	ORF-T	0.100324306	-0.514034545	-0.424080264	-0.147173855	-0.590525699
159553	160913	4	+	SFA1	ORF-T	ORF-T	0.130384555	-0.411127804	0.077166171	0.057097274	0.308477515
163433	163921	4	+	CUT051	CUTs	CUTs	2.583677855	0.185415003	-0.736498711	-0.528145105	1.001504239
164297	164889	4	+	CDC36	ORF-T	ORF-T	0.14176704	-0.543335564	-0.003171024	-0.224875866	0.054862968
167705	169201	4	+	ENT1	ORF-T	ORF-T	-0.136984875	-0.298588417	0.019319827	-0.036363542	0.217398866
172449	173289	4	+	STE7	ORF-T	ORF-T	0.276368068	-0.328945537	0.040828195	-0.105464279	-0.29266155
174841	176657	4	+	YDL156W	ORF-T	ORF-T	-0.396720282	-0.268774112	-0.470052972	-0.210058781	0.476112397
176657	178201	4	+	CLB3	ORF-T	ORF-T	-0.422814139	-0.664856943	-0.360802428	0.207154188	0.139901761
178201	181201	4	+	MSH5	ORF-T	ORF-T	0.638635149	0.228190474	-0.22571859	-0.522042128	0.124184971
183257	184705	4	+	RPC53	ORF-T	ORF-T	0.149558528	-0.253530401	-0.422087272	0.0228105	0.156980886
184945	188001	4	+	ATG9	ORF-T	ORF-T	-0.439490106	-0.181730259	0.718897409	0.1256285	1.249759546
190881	192409	4	+	RPN5	ORF-T	ORF-T	-0.035911621	-0.183457102	0.299530405	-0.099907619	0.243134762
192577	194465	4	+	LDB17	ORF-T	ORF-T	0.277337	-0.506396869	-0.485815534	-0.065919839	-0.397205064
198641	199569	4	+	unknown	no_ovlp	CUTs	1.928854038	0.125968264	-0.823864922	-0.550061934	-0.18504725
199969	201681	4	+	CCT4	ORF-T	ORF-T	0.010388948	-0.31125089	-0.27891523	-0.015988909	-0.082948562

201801	202817	4 +	unknown	no_ovlp	SUTs	0.128998427	0.237009169	-0.270018608	-0.080563096	0.946828662
202817	205305	4 +	BPL1	ORF-T	ORF-T	-0.29799709	-0.307871805	-0.303338744	-0.276612768	0.144402297
211409	213185	4 +	unknown	no_ovlp	SUTs	0.190791911	0.453930638	-0.061990504	0.181460676	0.087637375
213185	215753	4 +	RGT2	ORF-T	ORF-T	0.40199867	-0.10714096	-0.536982563	-0.493743595	-0.164361087
216153	216513	4 +	ARF2	ORF-T	ORF-T	0.222310413	-0.755787281	0.01152144	-0.231611722	-0.175390429
217601	218361	4 +	RPL35B	ORF-T	ORF-T	1.351188471	0.071107696	-1.507317103	-1.172422879	0.165882317
218497	222297	4 +	unknown	no_ovlp	SUTs	0.487696881	0.425749513	-0.36184561	-0.209691365	0.502309164
222297	223841	4 +	YDL133W	ORF-T	ORF-T	-0.056882611	0.049873007	0.284064942	0.095142111	0.424353015
224001	226913	4 +	CDC53	ORF-T	ORF-T	0.014369654	-0.066711664	0.46772817	-0.005167108	0.389809379
227313	228825	4 +	LYS21	ORF-T	ORF-T	-0.374176134	-0.905377844	-0.311169857	0.384810286	-0.549302552
229113	229593	4 +	STF1	ORF-T	ORF-T	-0.339855883	-0.188783179	1.389640781	0.362350027	0.280008275
230017	230329	4 +	RPP1B	ORF-T	ORF-T	-0.055553401	-1.018365374	-0.75291047	-0.910298953	0.06134468
230961	232257	4 +	YDL129W	ORF-T	ORF-T	0.82566892	-0.204777506	-0.334045764	0.17074481	0.416790054
232593	234057	4 +	VCX1	ORF-T	ORF-T	0.02544902	-0.37092094	-0.359640164	-0.249965262	-0.218932212
234873	235993	4 +	PCL2	ORF-T	ORF-T	0.285238085	-0.087772194	0.169945614	-0.351511047	0.305208739
238929	239521	4 +	unknown	no_ovlp	SUTs	0.885854339	-0.544473342	0.573923577	0.377098536	-0.070398624
240209	241265	4 +	YDL124W	ORF-T	ORF-T	-0.217717133	-0.23873669	0.272797305	-0.122017332	0.098533785
241265	242241	4 +	unknown	no_ovlp2	SUTs	0.047018705	-0.149053783	0.494904361	0.28603622	0.80812084
242241	245041	4 +	UBP1	ORF-T	ORF-T	0.055663417	-0.38948668	-0.55780337	-0.211792348	-0.41909343
245841	246617	4 +	YFH1	ORF-T	ORF-T	-0.14996999	-0.531704134	-0.008984163	-0.150331096	0.060801078
248249	251369	4 +	CYK3	ORF-T	ORF-T	-0.040451811	-0.411928899	-0.162153276	-0.340437675	0.283964988
251513	253761	4 +	NUP84	ORF-T	ORF-T	-0.185187792	-0.717940533	-0.077090378	0.066215993	-0.061917877
258897	263337	4 +	TRM3	ORF-T	ORF-T	-0.027311974	-0.486286193	-0.621413873	0.104017102	0.213953653
264465	264913	4 +	unknown	no_ovlp	CUTs	1.10484732	0.407292263	0.182168151	0.02827305	0.166874488
267817	268761	4 +	KIN28	ORF-T	ORF-T	0.097793379	-0.664333505	-0.158436547	-0.430330743	-0.19523534
268761	270113	4 +	MSS2	ORF-T	ORF-T	-0.516496706	-0.713979069	0.250187927	-0.19983101	-0.059904085
272385	273729	4 +	NSE4	ORF-T	ORF-T	-0.685720451	-0.147140667	0.63952206	0.145432359	1.432109352
276785	280289	4 +	POL3	ORF-T	ORF-T	-0.21816444	-0.145250212	-0.087163942	-0.043548271	0.383098963
283369	284529	4 +	BUG1	ORF-T	ORF-T	0.160999212	-0.315017939	0.088650169	-0.095426683	0.012199061
287001	289593	4 +	PMT1	ORF-T	ORF-T	-1.082609072	-0.480843782	-0.273612326	0.057817488	0.219018225
290441	292409	4 +	PMT5	ORF-T	ORF-T	-0.650646052	-0.547053121	-0.308465116	0.223085041	0.455857157
292769	293369	4 +	SRP14	ORF-T	ORF-T	0.221794419	-0.448232179	-0.441074858	-0.179998219	-0.386983404
296569	298321	4 +	YDL089W	ORF-T	ORF-T	-0.438835327	-0.54468221	0.266627629	0.106871759	-0.301659946
299865	301417	4 +	CUT053	CUTs	CUTs	-0.02384485	0.534127315	0.025007488	0.230648624	0.440293165
301417	302369	4 +	YDL086W	ORF-T	ORF-T	0.732163302	1.004438734	0.311931738	-0.016922732	-0.016436292
302369	303145	4 +	unknown	no_ovlp2	SUTs	-0.174849427	0.585152839	0.989502354	0.464677307	0.467866124
303145	305081	4 +	NDE2	ORF-T	ORF-T	-1.12341727	0.0970197	2.82684276	1.614219618	2.366941737
305081	306665	4 +	SUB2	ORF-T	ORF-T	-0.308915627	-0.387576108	-0.496946246	-0.048215765	0.052167627
308425	308801	4 +	RPL13A	ORF-T	ORF-T	0.515761799	-0.770484685	-1.321611951	-1.195694607	-0.145031359
310457	311073	4 +	CUT054	CUTs	CUTs	2.212482169	0.203617071	-1.125506966	-0.31062333	0.560952375
312873	313937	4 +	SUT043	SUTs	SUTs	0.305842799	-0.934845467	-1.485186442	-0.628260731	-0.712299895
315193	316977	4 +	SUT044	SUTs	SUTs	0.602741321	-0.277686861	0.842293954	1.244179692	0.751774676
320393	321009	4 +	SUT045	SUTs	SUTs	0.941426993	-0.299419109	-0.112172518	0.098123425	0.321440061
322281	322713	4 +	RPL31A	ORF-T	ORF-T	0.786736408	-0.978519995	-1.797790848	-1.65868181	-0.198681245
323793	324481	4 +	SUT046	SUTs	SUTs	1.07741435	0.054724328	-0.847236175	-0.183829237	-0.101106168
326441	329689	4 +	YDL073W	ORF-T	ORF-T	0.172412909	-0.274016195	-0.629749612	-0.58491288	-0.100801304
330785	331017	4 +	SUT047	SUTs	SUTs	0.427891516	-0.011771416	-0.407944767	-0.772498477	-1.393811919
331017	331777	4 +	BDF2	ORF-T	ORF-T	-0.085333734	-1.017468814	-0.863176842	-0.117154271	0.04916699
334809	336201	4 +	IDP1	ORF-T	ORF-T	-0.431836337	-0.391618427	-0.347390369	-0.05059031	-0.505486741
336321	337457	4 +	unknown	no_ovlp	SUTs	-0.333700544	-0.121850211	0.052496304	-0.186833337	1.014964737
337649	338201	4 +	UBC9	ORF-T	ORF-T	-0.068088278	-0.218115162	-0.308391375	-0.271532584	-0.327987445
341593	343985	4 +	TSR1	ORF-T	ORF-T	0.453556368	-0.15100783	-0.703729614	-0.109582576	0.226438115
345609	349833	4 +	USO1	ORF-T	ORF-T	-0.296385976	-0.382743443	-0.362896445	-0.167206114	0.18670208
351417	352809	4 +	YDL057W	ORF-T	ORF-T	0.009933065	0.645467367	0.997087323	-0.076846078	-0.329115262
352433	352809	4 +	unknown	no_ovlp2	SUTs	0.500155275	-0.095861726	0.145626963	-0.130211853	-0.303825187
352809	355433	4 +	MBP1	ORF-T	ORF-T	-0.005145378	-0.433357862	-0.145061499	0.109536119	0.018869841
357225	359681	4 +	unknown	no_ovlp	CUTs	1.53718008	0.415508626	0.309601648	-0.28829112	-1.220248369
359817	360633	4 +	unknown	no_ovlp	CUTs	1.317042547	-0.011514313	0.418293678	1.128525734	0.216500832
363913	364937	4 +	LHP1	ORF-T	ORF-T	-0.085466372	-0.593765665	-0.416417543	-0.206285338	0.320135179
369313	369609	4 +	SIT4	ORF-T	ORF-T	-0.155795315	-0.267665167	-1.058330863	-0.159020451	-1.004737867
371169	372033	4 +	NPC2	ORF-T	ORF-T	-0.486498658	-0.347439847	0.460560781	0.141924611	0.418947879
372209	372657	4 +	MRP10	ORF-T	ORF-T	-0.377710105	-0.586449568	-0.624526034	0.077121844	-0.747200015
381713	382065	4 +	SUT048	SUTs	SUTs	1.433328572	-0.449183674	0.058239264	0.256639853	-1.595370021
387025	387881	4 +	unknown	no_ovlp	CUTs	2.264421934	-0.204135753	-0.705408498	-0.171377114	-0.772131793
392409	394177	4 +	unknown	no_ovlp	SUTs	0.669291262	0.155834569	0.041430709	-0.419276717	-0.018768564
394177	397289	4 +	DBP10	ORF-T	ORF-T	0.582434074	-0.09299798	-0.880698883	-0.074187664	0.237704173
397497	399217	4 +	PRP9	ORF-T	ORF-T	0.402670987	-0.18157034	-0.561935454	-0.277596569	-0.15330815
399489	400769	4 +	ARP2	ORF-T	ORF-T	-0.054651517	-0.05727477	0.095829131	-0.02386016	-0.295828477
405225	408577	4 +	SUT049	SUTs	SUTs	0.620203344	0.462712877	-0.098127272	0.324983763	-0.273315288
411737	413225	4 +	GPD1	ORF-T	ORF-T	-0.536075526	-0.446286814	0.329712505	0.122609046	-0.380604391
413945	414929	4 +	GPM2	ORF-T	ORF-T	1.235114159	1.98060799	1.967719911	1.434338605	-1.563122719
422017	422369	4 +	CUT058	CUTs	CUTs	1.636759322	0.121424966	0.688561114	0.708537596	-0.773992879
423737	424097	4 +	unknown	no_ovlp2	CUTs	2.185501356	0.292457347	-0.894795464	-0.64994887	0.357293504
424097	425817	4 +	CDC7	ORF-T	ORF-T	0.208890021	-0.199407601	0.309684983	-0.091303893	0.122087548
427225	428537	4 +	NOP1	ORF-T	ORF-T	-0.292594857	-0.310640752	-0.755317474	-0.032357887	-0.392781016
428593	428937	4 +	unknown	no_ovlp	SUTs	-0.115001928	0.407690935	-0.158587177	0.111208242	-0.080509644
428937	431009	4 +	SLX5	ORF-T	ORF-T	-0.022004227	-0.055096766	0.284866095	0.312076143	0.267042025
432297	433313	4 +	GRX7	ORF-T	ORF-T	-0.088792826	-0.878577665	-0.26799278	0.080739459	-0.129969677
433521	434113	4 +	APC11	ORF-T	ORF-T	1.010545135	0.725719596	-0.113573498	-0.153031961	-0.438031016
438017	439457	4 +	RPT2	ORF-T	ORF-T	0.009792465	-0.284888954	0.026617635	-0.055591024	-0.406449332
439625	440865	4 +	PTC1	ORF-T	ORF-T	0.146398578	-0.660185509	-0.301863193	-0.258503709	0.041664218
442985	443761	4 +	ATP16	ORF-T	ORF-T	-0.278600377	0.175417887	0.612879838	-0.132762215	0.647386107

444561	446497	4	+	MCD1	ORF-T	ORF-T	3.79E-05	0.122818884	-0.257104831	-0.415792033	0.539013619
447889	449385	4	+	RMD1	ORF-T	ORF-T	-0.0339385	-0.317539499	-0.22436331	0.129866038	0.319798736
452993	453849	4	+	YRB1	ORF-T	ORF-T	-0.179771207	-0.69893621	-0.315230748	0.04900684	-0.059556914
454089	455081	4	+	RCR2, YDR00	other	other	-0.067297571	-0.466275092	0.222687615	0.02242315	0.011932616
455241	456753	4	+	RAD57	ORF-T	ORF-T	0.174150364	-0.462953886	-0.312025097	-0.399459192	-0.193135861
457233	458361	4	+	unknown	no_ovlp	CUTs	1.395317252	0.482502206	-0.822010238	-1.129900528	0.148549532
461689	462585	4	+	TRP1	ORF-T	ORF-T	0.24958913	2.106735829	2.392728174	1.191031121	1.086936719
462585	463249	4	+	unknown	no_ovlp2	SUTs	0.006982685	0.172930669	1.20899433	0.210197156	0.088046901
463249	465569	4	+	GAL3	ORF-T	ORF-T	0.473277769	1.122586434	1.14167932	0.832144136	-0.533464098
465569	466841	4	+	SNQ2	ORF-T	ORF-T	-0.49518936	-0.735301489	0.29801145	0.139306227	-0.0173512
471025	471817	4	+	unknown	no_ovlp	SUTs	0.474332176	0.564109272	0.478838644	-0.415220516	-0.358198627
471817	472993	4	+	RPL4B	ORF-T	ORF-T	-1.446152225	-0.634037526	-0.048146951	0.242959941	0.498115574
472993	473873	4	+	PSF1	ORF-T	ORF-T	-0.106844289	-1.000273469	-0.122007461	-0.227965043	-0.181936376
474025	476361	4	+	RAD61	ORF-T	ORF-T	0.527449727	0.004569622	0.075370963	-0.053593404	-0.2579057
476033	476361	4	+	unknown	no_ovlp2	SUTs	0.0615945	0.209996929	0.401275822	0.383248639	-0.456099063
482602	483770	4	+	CUT062	CUTs	CUTs	1.092448713	0.291881397	0.264100765	0.609881782	0.691728636
485634	486338	4	+	unknown	no_ovlp	CUTs	2.893894063	0.212226799	-0.965657126	-0.529754523	-0.557011322
486722	488082	4	+	FAL1	ORF-T	ORF-T	0.869210763	-0.048131129	-0.427081834	-0.044864932	0.6828412
489474	491050	4	+	SES1, FYV1	other	other	0.261957233	-0.26760556	-0.621077511	-0.196395855	-0.45792956
491554	491922	4	+	FYV1, RPS11.	other	other	0.764160893	-0.68545857	-1.17517982	-1.341986307	-0.277804808
492490	493978	4	+	unknown	no_ovlp	SUTs	-0.078526501	0.357506204	0.03825785	0.035772488	0.582710038
493978	495394	4	+	unknown	no_ovlp	SUTs	0.691463223	0.362576577	-0.388806899	-0.379007374	1.141149231
501458	501786	4	+	SUT052	SUTs	SUTs	-1.083413502	-1.079129875	1.672618831	0.394212325	-0.050342115
501786	502266	4	+	CUT064	CUTs	CUTs	3.302377633	0.169420056	-1.344724395	0.230489383	-0.16389885
503498	504027	4	+	MIC14	ORF-T	ORF-T	-0.600190833	-0.7286655	0.772564991	-0.01310209	0.617117658
506331	506987	4	+	SUT053	SUTs	SUTs	1.239661115	-0.841185999	-0.360389013	-0.30611393	-0.784767268
507811	509587	4	+	MRH1	ORF-T	ORF-T	-0.497023119	-0.306208091	-0.402042998	0.003734776	-0.079291214
512491	513259	4	+	unknown	no_ovlp	SUTs	0.481243123	-0.929416692	-0.099676812	-0.260770464	-0.661307676
521299	521771	4	+	YDR034W-B	ORF-T	ORF-T	-1.125094521	-1.095560153	2.979164118	1.228814321	2.868653377
521563	521771	4	+	unknown	no_ovlp2	SUTs	-1.098627822	-2.085096393	2.57714599	1.24643832	2.874733316
521771	523171	4	+	ARO3	ORF-T	ORF-T	0.199238392	-0.678288537	-0.585584046	0.05922913	-0.741309832
525011	525395	4	+	unknown	no_ovlp	SUTs	0.9492525	0.260917322	0.139930462	-0.576530556	-0.501938651
525395	527291	4	+	KRS1	ORF-T	ORF-T	-0.117992171	-0.254580111	-0.441599794	-0.112198277	-0.446432272
539795	540579	4	+	RSM10	ORF-T	ORF-T	-0.956609143	-0.964003327	0.327977186	0.528764988	0.297932538
542635	543163	4	+	CUT065	CUTs	CUTs	2.443791593	-0.224081609	-0.708950204	-0.436395683	-0.02778713
544099	544803	4	+	SUT055	SUTs	SUTs	0.17218766	-0.330970889	0.000193489	-0.001427638	-0.930376661
546555	547771	4	+	HEM13	ORF-T	ORF-T	-0.828310787	-0.405773024	-0.01132949	0.816017314	0.390342978
551403	553019	4	+	HEM12	ORF-T	ORF-T	-0.1689957	-0.926070595	-0.549477128	-0.244720704	-0.483601687
553227	555219	4	+	YDR049W	ORF-T	ORF-T	0.53773273	0.081974579	-0.333501414	-0.310183898	0.006656692
556771	557523	4	+	SUT056	SUTs	SUTs	1.769472957	0.212966888	-0.227816102	0.161484553	0.45097785
561035	561715	4	+	unknown	no_ovlp	CUTs	2.057476296	0.251941186	-1.257262572	-0.482785928	-0.128910354
563243	563467	4	+	CUT066	CUTs	CUTs	1.7592543	-0.583076797	-0.338316201	0.218109867	0.498933558
563467	565003	4	+	PST1	ORF-T	ORF-T	-0.307861815	0.009022653	0.226541236	0.016982385	0.484050206
565907	567635	4	+	YOS9	ORF-T	ORF-T	-0.059270193	-0.453024346	0.311552631	-0.144468542	0.297317916
570579	573771	4	+	MAK21	ORF-T	ORF-T	0.222267008	-0.216828648	-0.770962285	-0.021939112	0.156485706
573771	574155	4	+	unknown	no_ovlp2	SUTs	-0.384137301	-0.199402549	0.109552123	-0.237493941	0.314822728
574155	575835	4	+	YDR061W	ORF-T	ORF-T	0.036391359	-0.165481476	-0.036981235	-0.311840273	0.143170851
576099	578443	4	+	LCB2	ORF-T	ORF-T	-0.340532419	-0.38738247	-0.358159876	-0.024829799	-0.253234573
578563	579219	4	+	YDR063W	ORF-T	ORF-T	-0.34927967	-0.834574747	0.137625764	-0.297989251	0.197957137
579411	580027	4	+	RPS13	ORF-T	ORF-T	1.209170761	-0.201442839	-1.015549868	-0.950174607	-0.249956705
580547	582371	4	+	YDR065W	ORF-T	ORF-T	0.277632259	-0.574560369	-0.545691308	-0.58367167	-0.541644682
583683	584787	4	+	DOS2	ORF-T	ORF-T	0.01688017	-0.849606574	-0.240271235	-0.384906541	-0.384212798
588043	588507	4	+	CUT067	CUTs	CUTs	1.852864501	0.106856758	0.10789091	0.163878153	0.292977658
591651	592227	4	+	unknown	no_ovlp	SUTs	0.032481029	-0.206844697	0.15933915	-0.019159118	0.175721303
592227	593227	4	+	SNF11	ORF-T	ORF-T	0.330479343	-1.107541325	-0.653421819	-0.211327975	-0.429941973
593795	594907	4	+	TPS2	ORF-T	ORF-T	-0.452525429	-0.879408421	0.476215172	0.47595344	-0.180345609
596963	598267	4	+	PPH3	ORF-T	ORF-T	0.053569236	-0.71836493	-0.34757152	-0.248617936	0.292096177
598459	600571	4	+	RAD55	ORF-T	ORF-T	-0.084523946	0.393030105	-0.295095099	0.083245974	0.797525477
600747	602139	4	+	SED1	ORF-T	ORF-T	-0.51269624	0.056265148	0.312450637	0.045675174	0.181529932
603043	603683	4	+	PET100	ORF-T	ORF-T	-0.21916299	-0.746454922	0.335548426	-0.550484403	-0.191819753
603995	607227	4	+	VPS41	ORF-T	ORF-T	-0.099924116	-0.185330273	0.399535808	0.027021754	-0.275246185
610371	612067	4	+	STN1	ORF-T	ORF-T	0.390632469	-0.030344414	0.200015173	-0.32998652	0.512238894
612067	613348	4	+	RRP8	ORF-T	ORF-T	0.59245338	-0.317739125	-0.859908027	-0.42536478	0.036153406
614228	615076	4	+	SUT057	SUTs	SUTs	1.396517742	-0.267202637	-1.590411437	-0.583018055	-0.519454735
616740	617084	4	+	unknown	no_ovlp	CUTs	2.958403707	-0.068404938	0.140395986	0.177717739	-0.096637538
622036	624900	4	+	YDR089W	ORF-T	ORF-T	0.512143275	-0.392136822	-0.341744108	-0.294467655	0.05238726
629900	630180	4	+	UBC13	ORF-T	ORF-T	1.050857461	0.195292338	-0.821894743	-0.577345409	-0.891363244
631020	636180	4	+	DNF2, YDR09	ORF-T	ORF-T	-0.355350829	-0.622771497	-0.414096335	-0.038729839	-0.35937545
636540	637124	4	+	GIS1	ORF-T	ORF-T	-0.76908729	-1.559832914	0.492420037	0.19606332	-0.554044682
652660	652876	4	+	BMH2	ORF-T	ORF-T	0.36556984	-0.444100342	0.257558348	-0.134177333	-0.131217112
654764	654980	4	+	unknown	no_ovlp	SUTs	0.123109452	0.617279579	-0.047020782	-0.052489758	-0.036697905
654980	655724	4	+	TVP15	ORF-T	ORF-T	-0.25855349	-1.173408039	0.040552203	-0.083282116	0.830918333
658292	659348	4	+	STE5	ORF-T	ORF-T	0.331754506	0.106309385	-0.141567481	0.222982712	-0.061424559
666988	668436	4	+	unknown	no_ovlp	CUTs	1.413911183	-0.058833658	-0.170259053	-0.674021996	-0.36822225
668436	668908	4	+	unknown	no_ovlp	SUTs	0.01106586	-1.128059645	-0.115463363	-0.862736154	-2.109416713
668908	671228	4	+	unknown	no_ovlp2	CUTs	2.079430834	0.192777278	-0.622014412	-0.903788995	-2.592078101
671228	673460	4	+	GSG1	ORF-T	ORF-T	0.162357412	-0.339738582	-0.13404958	-0.315650877	0.299844828
674548	676068	4	+	unknown	no_ovlp	SUTs	-0.345124896	0.43394682	0.252121862	0.133767108	1.018773932
676068	677996	4	+	FOB1	ORF-T	ORF-T	0.487981568	-0.148667558	-0.78113361	-0.310027396	-0.287330701
680236	681084	4	+	SUT058	SUTs	SUTs	1.909157441	0.173921158	0.116277028	0.536413154	0.675421483
682140	682652	4	+	YDR115W	ORF-T	ORF-T	-0.59686775	-1.052680915	0.399017677	0.091549013	0.199302588
685916	688140	4	+	APC4, YDR11	ORF-T	ORF-T	-0.128888126	-0.575918242	-0.038921077	-0.264224851	0.36569014

688140	690588	4 +	YDR119W	ORF-T	ORF-T	-0.335946259	-0.701170423	-0.331613151	-0.095784665	-0.213773061
690972	691460	4 +	YDR119W-A	ORF-T	ORF-T	-0.250796224	-0.30393477	1.780480541	1.977373554	-2.015076856
693460	694284	4 +	DPB4	ORF-T	ORF-T	0.384713866	-0.245028392	-0.110713454	-0.195381729	0.243664066
694404	698060	4 +	KIN1	ORF-T	ORF-T	-0.205029529	-0.157723049	-0.151995449	0.05016715	-0.07886064
699820	701724	4 +	YDR124W	ORF-T	ORF-T	0.998568069	-0.387815163	-0.57434098	0.330786554	0.248228477
701380	701724	4 +	unknown	no_ovlp2	SUTs	0.315858987	-0.590454641	-0.626989788	1.010704471	0.310635198
703244	704412	4 +	SWF1	ORF-T	ORF-T	0.677430917	0.7473828	0.463721683	0.043875167	0.006091643
704412	709388	4 +	ARO1	ORF-T	ORF-T	-0.371183724	-0.616908954	-0.825809234	0.058549772	-0.871248945
709516	713180	4 +	YDR128W	ORF-T	ORF-T	-0.032605137	-0.21019222	-0.107120945	0.068058092	0.15423941
715652	716316	4 +	CUT068	CUTs	CUTs	1.177684613	0.232126047	-0.176412946	0.686827935	0.555847601
718596	719892	4 +	SUT059	SUTs	SUTs	-0.058919073	-0.688953059	-0.050070702	0.311103655	-0.362961535
722588	725580	4 +	SUT060	SUTs	SUTs	-0.072577461	-0.070261498	1.047016009	1.420576544	0.099444394
728164	730396	4 +	RGP1	ORF-T	ORF-T	0.251447282	-0.569428818	0.05605788	0.029129628	-0.212647313
730572	733468	4 +	HPR1	ORF-T	ORF-T	0.066898543	-0.631106818	-0.484230599	-0.290501623	-0.331729392
734132	734884	4 +	MTQ2	ORF-T	ORF-T	0.140526362	-0.244244221	0.272871905	-0.011045722	0.022495654
746660	748580	4 +	TAF12	ORF-T	ORF-T	0.006305159	-0.349399103	-0.069098557	-0.076956094	-0.137822588
751156	751516	4 +	EKI1	ORF-T	ORF-T	0.471985324	-0.523308457	-0.53290441	-1.212171812	-0.057352678
755572	757244	4 +	NUM1	ORF-T	ORF-T	0.018183525	-0.33993168	-0.0147226	0.099271135	0.303161343
765668	766556	4 +	GIR2	ORF-T	ORF-T	0.260953057	-0.237868338	-0.455043976	-0.029893188	-0.051655686
769204	769484	4 +	unknown	no_ovlp	SUTs	0.423196179	0.446073753	0.797240513	-0.227515198	0.307612497
769484	770028	4 +	RPA14, YDR1	ORF-T	ORF-T	0.37925535	-0.249539707	-0.648480858	-0.460500688	0.017435685
770324	771652	4 +	HOM2	ORF-T	ORF-T	-0.111810767	-1.051441853	-0.489385751	-0.06712812	-0.458896683
771788	775772	4 +	SAC3	ORF-T	ORF-T	0.001091651	-0.332964741	-0.153619857	-0.227589369	-0.091008328
776004	778844	4 +	SSY1	ORF-T	ORF-T	-0.21747777	-0.363030024	-0.348617994	-0.030887078	0.00189299
779004	780292	4 +	YDR161W	ORF-T	ORF-T	0.134490698	-0.455804927	-0.6124418	0.035213822	0.308745072
781380	781964	4 +	CWC15	ORF-T	ORF-T	0.643590782	0.404672345	-0.299226405	-0.412852934	-0.009827624
784836	786308	4 +	TRM82	ORF-T	ORF-T	0.445851742	-0.3265359	-0.727597549	-0.154741005	0.011969181
789420	790172	4 +	TAF10	ORF-T	ORF-T	0.165309024	-0.095717896	0.299243231	-0.336610018	-0.005635258
790292	791940	4 +	CDC37	ORF-T	ORF-T	-0.190501625	-0.541799574	-0.138974623	-0.046116444	-0.201720952
794484	795276	4 +	CUT071	CUTs	CUTs	1.755301478	-0.279484433	-0.272305046	-0.041117302	-0.238369435
803132	805924	4 +	unknown	no_ovlp	SUTs	-0.343014427	-1.449285831	0.058903641	-0.582552144	-0.659151377
806556	807828	4 +	HSP42	ORF-T	ORF-T	0.056450224	-1.013067299	0.577633718	0.370979639	-0.3969555
808276	810556	4 +	SUP35	ORF-T	ORF-T	0.032534628	-0.485346084	-0.523242797	0.029972408	-0.191431706
812084	813076	4 +	HMO1	ORF-T	ORF-T	0.070226214	-0.506400443	-0.979595908	-0.369406695	-0.344885625
814436	816604	4 +	NGG1	ORF-T	ORF-T	-0.363758559	-0.70019742	-0.457623064	-0.249546473	-0.242737631
816788	817644	4 +	UBC1	ORF-T	ORF-T	0.180571707	-0.507367179	0.063391568	-0.085771307	-0.028058995
817852	818692	4 +	SDH4	ORF-T	ORF-T	-0.706104473	-0.401856087	1.00648041	0.408137425	0.702230445
819372	820901	4 +	YDR179W-A	ORF-T	ORF-T	0.10162009	-0.081622266	-0.464833236	-0.383304432	0.680073828
821165	825813	4 +	SCC2	ORF-T	ORF-T	-0.011518846	-0.576587307	-0.721162423	-0.108573382	1.033909539
827541	829157	4 +	CDC1, YDR18	other	other	-0.258331482	-0.48603536	-0.075217513	0.24391151	-0.118873856
829557	830325	4 +	PLP1	ORF-T	ORF-T	0.749326774	-0.395952798	-0.633437001	-0.434811802	-1.189660194
831813	832621	4 +	SUT061	SUTs	SUTs	0.511327375	-1.345957315	-0.958827138	-0.935503771	-1.175982427
836341	838221	4 +	CCT6	ORF-T	ORF-T	-0.244540385	-0.477148679	-0.491819402	0.008420944	-0.161946452
838349	840493	4 +	SLY1	ORF-T	ORF-T	-0.205384114	-0.380107088	-0.474880278	-0.110805757	-0.031737413
842317	843485	4 +	HST4	ORF-T	ORF-T	0.560837581	0.435954619	-0.39641763	-0.97368041	-0.318408692
848365	850277	4 +	REF2	ORF-T	ORF-T	0.572559463	-0.844733951	-0.535975403	-0.111920165	0.063323853
851197	852501	4 +	CBS2	ORF-T	ORF-T	-0.318386251	-0.699491787	0.411030733	-0.083568795	-0.044578305
856277	856925	4 +	SPC19	ORF-T	ORF-T	-0.650715987	-0.723697363	0.144375375	0.021461307	0.437792374
858141	859261	4 +	COQ4	ORF-T	ORF-T	0.482445853	0.507272522	0.493153044	-0.101398334	0.006768771
859317	861557	4 +	MSC2	ORF-T	ORF-T	-0.388356297	-0.656983175	-0.176428715	-0.224338322	-0.018529134
861821	864861	4 +	EBS1	ORF-T	ORF-T	0.156028593	-0.326777764	-0.307034176	-0.077380766	-0.077563348
868101	870677	4 +	MSS4	ORF-T	ORF-T	-0.40130354	-0.802662243	0.08451783	0.072051898	0.132710672
871045	871717	4 +	YDR210W	ORF-T	ORF-T	0.122097894	-0.392792664	-0.150889262	-0.324247161	-0.133659872
884213	884701	4 +	unknown	no_ovlp	CUTs	1.895643003	1.071681483	-0.294151883	-0.926247334	-1.14313934
884701	887053	4 +	GCD6	ORF-T	ORF-T	0.147308671	-0.364398366	-0.281452728	-0.01808248	0.064540582
887157	888997	4 +	TCP1	ORF-T	ORF-T	-0.092248839	-0.722646074	-0.562399737	0.113671768	-0.168150073
889549	892757	4 +	UPC2	ORF-T	ORF-T	-0.23432809	-0.563665338	-0.416448017	0.660852746	0.138285069
892757	894013	4 +	AHA1	ORF-T	ORF-T	0.037892588	-0.502311531	0.11160603	0.088329977	-0.516637189
894573	897109	4 +	ADR1	ORF-T	ORF-T	-1.014485098	-0.648148864	1.421092351	1.330965208	0.93023984
907325	909981	4 +	GTB1	ORF-T	ORF-T	-0.305224022	-0.019137563	-0.095763797	-0.029482929	0.161558166
909645	909981	4 +	unknown	no_ovlp2	CUTs	1.066589643	-0.16665663	-1.006287564	-0.574769883	-0.974754986
909981	911445	4 +	YDR222W	ORF-T	ORF-T	0.337637375	-1.897058173	0.039857602	-0.378687873	-0.096079067
911445	915229	4 +	CRF1	ORF-T	ORF-T	0.087305491	0.295427623	1.332873064	1.541801419	0.473288827
913701	915229	4 +	unknown	no_ovlp2	SUTs	0.099078038	0.266405061	0.614730873	0.092273858	-0.032364981
915229	915461	4 +	CUT073	CUTs	CUTs	1.765078915	-0.614797917	-0.305426838	0.577583442	-0.547097708
915461	916101	4 +	HTA1	ORF-T	ORF-T	-0.447675253	-0.75288726	-0.210765252	0.050421263	-0.03381503
916445	917261	4 +	ADK1	ORF-T	ORF-T	0.021865993	-0.290604751	-0.443153393	-0.009442308	-0.251919551
917461	921837	4 +	SIR4	ORF-T	ORF-T	-0.060680522	-0.075254903	0.073356397	-0.117920385	0.337770818
924725	926197	4 +	IVY1	ORF-T	ORF-T	-0.067121026	-0.753142535	-0.010654376	-0.049891367	-0.192116399
926565	927213	4 +	unknown	no_ovlp	CUTs	1.666498124	0.220658555	-0.546396365	-0.469672298	0.132741509
927213	929277	4 +	HEM1	ORF-T	ORF-T	-0.302537198	-0.255615571	-0.26167068	0.088122568	0.189283059
930933	933333	4 +	LYS4	ORF-T	ORF-T	0.34105986	-0.920265222	-1.099900205	0.003352759	-0.349265301
933493	935573	4 +	PRP42	ORF-T	ORF-T	0.055378895	-0.854644849	-0.801454807	-0.296059874	-0.63794299
936509	937613	4 +	MRPL7	ORF-T	ORF-T	-0.87030166	-0.672759739	-0.071191088	-0.217228439	-0.19223702
945341	946285	4 +	unknown	no_ovlp	SUTs	0.405195478	-1.305714776	0.025354898	-0.674124827	-0.870907271
946733	948573	4 +	AMD2	ORF-T	ORF-T	-0.041289516	-0.415882062	-0.233505821	-0.317426581	-2.311823817
950557	952589	4 +	PEX5	ORF-T	ORF-T	-0.407074975	-0.669674548	0.152440804	0.126964853	0.175555439
952773	954133	4 +	MNN10	ORF-T	ORF-T	0.041200253	-0.360506539	-0.723963891	-0.31170533	0.124792491
954285	954989	4 +	TRS23	ORF-T	ORF-T	-0.164598878	-0.725716078	0.285067394	-0.06216864	0.252686691
955709	957757	4 +	VHS1	ORF-T	ORF-T	-0.102048475	-0.756899623	-0.305497894	0.273557605	-0.885421373
960309	963277	4 +	PAM1	ORF-T	ORF-T	-0.309243701	-0.550341142	-0.399567225	-0.105813175	-0.437690087
963413	964757	4 +	BTT1	ORF-T	ORF-T	0.592309236	-0.359298388	-0.424275563	-0.287675442	-0.085125956

965045	967021	4	+	CHL4	ORF-T	ORF-T	-0.134442032	-0.832094944	-0.303792544	-0.398965802	0.125195923
968069	969749	4	+	SUT063	SUTs	SUTs	0.467180393	-0.062908371	-0.355495235	-0.067210075	0.296074222
974629	974981	4	+	CUT075	CUTs	CUTs	1.89061106	0.428602434	0.579367625	0.91016278	0.206617457
976509	977045	4	+	unknown	no_ovlp	CUTs	1.1728462	-0.365183565	0.064430218	-0.397568574	-1.084665055
977045	977317	4	+	SUT064	SUTs	SUTs	1.523348055	-1.051960668	0.285182556	-0.545394159	-0.570188497
979533	980117	4	+	unknown	no_ovlp	CUTs	2.149412867	0.213095183	0.024989789	0.297449393	-1.883660621
992829	993141	4	+	unknown	no_ovlp	CUTs	1.151820965	-0.257462157	-0.330072928	-0.570236085	-1.933036814
993141	994141	4	+	YDR262W	ORF-T	ORF-T	0.255185298	-0.589405984	-0.144246129	0.117623762	-0.236936755
998669	1000037	4	+	PEX10	ORF-T	ORF-T	0.220296841	-0.447763298	-0.162699851	-0.220543232	0.095772825
1003997	1005581	4	+	unknown	no_ovlp2	SUTs	-0.178204837	-0.186834416	-0.066930484	-0.652916269	0.41339393
1005581	1008789	4	+	CCC2	ORF-T	ORF-T	-0.311203159	-0.496286298	0.169275313	0.438609238	0.441356659
1008965	1009917	4	+	GLO2	ORF-T	ORF-T	-0.379209717	-0.855943277	0.146973707	0.39393511	-0.570236969
1009917	1011333	4	+	DON1	ORF-T	ORF-T	0.547786895	0.085191242	-0.424976853	-0.272132655	-1.231429107
1011589	1012285	4	+	BSC2	ORF-T	ORF-T	0.058221138	0.004517186	0.140760994	0.467864447	0.537123818
1013829	1014333	4	+	unknown	no_ovlp	CUTs	1.316149775	0.495990617	-0.100155923	0.127271933	0.562690215
1014333	1015293	4	+	SUT065	SUTs	SUTs	1.121644323	-0.257676037	-1.256239422	-0.11098193	0.147647122
1019269	1020445	4	+	RNH202	ORF-T	ORF-T	-0.25150835	-0.085904093	0.206459346	-0.081216006	0.652520718
1020621	1021765	4	+	RRP45	ORF-T	ORF-T	0.214594039	-0.716914651	-0.525844156	-0.041964924	0.161569244
1030517	1030981	4	+	CUT076	CUTs	CUTs	2.565551856	-0.100170779	-1.021224846	-0.047778638	-0.416974522
1030981	1033389	4	+	unknown	no_ovlp2	CUTs	1.269716397	0.399239252	-0.609588539	-0.352645502	0.706721461
1033389	1035189	4	+	ZIP1	ORF-T	ORF-T	-0.480430519	-0.855963181	-0.136084813	-0.39067897	2.329031405
1035933	1036949	4	+	YDR287W	ORF-T	ORF-T	-0.225354697	0.019363026	0.473133122	0.288540239	-0.082075845
1037157	1038261	4	+	NSE3	ORF-T	ORF-T	-0.169460274	-0.993254796	-0.179872786	0.10017559	-0.199666504
1039725	1043069	4	+	HRQ1	ORF-T	ORF-T	-0.133464137	-0.330336449	-0.467695507	-0.403620271	0.03387341
1049861	1050685	4	+	unknown	no_ovlp	CUTs	1.256660039	-0.431167485	-0.207299894	-0.048251509	0.20607662
1055165	1055925	4	+	MHR1	ORF-T	ORF-T	-0.803273908	-0.798532941	0.203101103	-0.322363467	-0.175139007
1056485	1057765	4	+	SUR2	ORF-T	ORF-T	0.309557833	-0.294505794	-0.308442824	-0.071568621	-0.140719201
1059605	1061349	4	+	BFR2	ORF-T	ORF-T	0.927003472	0.207163474	-0.822253559	-0.551205506	0.525199189
1063285	1067557	4	+	CFT1	ORF-T	ORF-T	-0.305963081	-0.385575487	-0.280629774	0.011220029	-0.067824725
1067685	1068469	4	+	GPI11	ORF-T	ORF-T	-0.371972079	-0.485988923	-0.562591087	-0.345472403	-0.216394117
1071461	1072789	4	+	unknown	no_ovlp	SUTs	-0.130547845	-0.659871156	-0.698214235	-0.124329278	-0.081120783
1072789	1073285	4	+	CUT078	CUTs	CUTs	2.465180907	0.085056745	0.316313634	-0.134577734	-0.542533335
1075829	1077981	4	+	YDR307W	ORF-T	ORF-T	-0.712675395	-0.680365138	-0.000759176	-0.067665449	-0.505724075
1080789	1081365	4	+	SUT066	SUTs	SUTs	0.7928445	-0.547982866	0.219388583	-0.229689288	0.07082271
1085069	1087093	4	+	TFB1	ORF-T	ORF-T	-0.06518728	-0.680095538	-0.276027222	-0.162030084	-0.200290314
1087533	1089061	4	+	SSF2	ORF-T	ORF-T	-0.2927367	-0.288717113	-0.24404149	-0.155389467	0.696866264
1090373	1090813	4	+	CUT079	CUTs	CUTs	3.24671699	0.832661148	-0.448797055	0.066374131	0.250754486
1092725	1093773	4	+	CUT080	CUTs	CUTs	1.60550382	-0.008205353	-0.338188044	-0.319742715	-0.346296323
1093773	1095253	4	+	OMS1	ORF-T	ORF-T	-0.379984714	-0.450052241	0.059549456	-0.206097399	-0.310339421
1095253	1102133	4	+	unknown	no_ovlp2	SUTs	-0.036183695	0.368824916	0.190148577	-0.19634425	0.136696078
1102133	1103733	4	+	HIM1	ORF-T	ORF-T	-0.175028166	0.747434422	0.753269984	0.053967945	-0.153272431
1103461	1103733	4	+	unknown	no_ovlp2	SUTs	-0.10534552	0.141642818	-0.080810654	0.144422982	-0.066236919
1103733	1105053	4	+	MCM21	ORF-T	ORF-T	0.235221726	-0.667020694	-0.44208833	-0.249009559	-0.163169841
1106077	1106789	4	+	CUT081	CUTs	CUTs	2.413843742	0.404138403	-0.471877439	0.159922526	0.438396113
1108253	1108669	4	+	unknown	no_ovlp	SUTs	0.29347098	-0.234083907	-0.026388905	-0.791715043	-1.130366163
1108669	1109997	4	+	ASP1	ORF-T	ORF-T	-0.128249056	-0.492880235	-1.035242663	-0.442828567	0.303679267
1110573	1111765	4	+	MRPL35	ORF-T	ORF-T	-1.179408657	-0.951389885	0.083914742	0.251006949	-0.146895637
1112501	1113365	4	+	unknown	no_ovlp	CUTs	2.441152175	0.500569681	0.097705195	-0.206081712	0.20089594
1117109	1120277	4	+	YCG1	ORF-T	ORF-T	0.067639186	0.022328797	-0.25712882	-0.395452045	0.963023333
1127805	1130933	4	+	UBX5	ORF-T	ORF-T	-0.144188454	-0.136170047	0.380006158	0.219599759	0.411555974
1129469	1130933	4	+	unknown	no_ovlp2	SUTs	-0.025259731	-0.558272994	-0.899416655	-0.114203492	-0.249510633
1130933	1133141	4	+	IRC3	ORF-T	ORF-T	0.218299295	-0.758087268	-0.493242183	-0.351880449	-0.238485489
1135773	1140565	4	+	SWR1	ORF-T	ORF-T	-0.19712209	-0.98343318	-0.527677572	-0.01296222	-0.263361555
1140813	1144933	4	+	MSN5	ORF-T	ORF-T	0.031505179	-0.391750409	-0.105555969	-0.109718372	-0.133200362
1145093	1146125	4	+	YDR336W	ORF-T	ORF-T	0.202351562	-0.694010854	-0.48939787	-0.300913205	-0.114987546
1146277	1147229	4	+	MRPS28	ORF-T	ORF-T	-0.725597019	-1.068898233	-0.42133385	-0.225924504	0.094230602
1154053	1159045	4	+	SUT068	SUTs	SUTs	-0.186464183	-0.28663277	2.095953457	2.141084205	-0.294037594
1162341	1162925	4	+	SUT069	SUTs	SUTs	0.500447264	0.223808902	0.674135688	0.001260969	-0.598930133
1165405	1166797	4	+	CUT085	CUTs	CUTs	1.295411802	-0.304763255	-0.255744472	0.130424692	-1.342206214
1166797	1167461	4	+	unknown	no_ovlp	CUTs	2.987307352	0.254880771	-1.056997042	-0.304174825	-0.578461591
1169093	1170245	4	+	MRP1	ORF-T	ORF-T	-0.755334744	-0.547391126	-0.234143534	-0.030611861	0.26380979
1174445	1175637	4	+	SUT071	SUTs	SUTs	0.723513053	-0.215685217	-0.00374375	-0.103606383	-1.611270655
1178445	1179525	4	+	SBE2	ORF-T	ORF-T	0.025418441	-0.352120004	-0.125977656	-0.238094799	0.11433026
1181629	1182981	4	+	YDR352W	ORF-T	ORF-T	-0.341634988	-0.775190033	-0.579464805	-0.079248811	0.054083441
1183229	1184277	4	+	TRR1	ORF-T	ORF-T	-0.058878442	-0.104627831	-0.450047649	0.012465133	-0.339510571
1184277	1184629	4	+	unknown	no_ovlp2	SUTs	0.489838472	-0.864376791	-0.565290334	-0.597129252	-0.582602652
1184629	1185949	4	+	TRP4	ORF-T	ORF-T	0.1952747	-0.503251388	-0.975792433	-0.262555837	-0.295442879
1186085	1189565	4	+	SPC110	ORF-T	ORF-T	0.055703991	-0.418903862	-0.433084904	-0.295617457	0.452943921
1189213	1189565	4	+	unknown	no_ovlp2	SUTs	0.939560075	-0.097032711	0.269957741	0.006479252	-1.24043959
1190013	1191909	4	+	GGA1	ORF-T	ORF-T	-0.080894989	-0.46904502	0.725839109	0.186565873	0.36451313
1195341	1196237	4	+	unknown	no_ovlp	CUTs	1.629167687	0.176819361	-0.367045729	-0.26706552	0.547544281
1199005	1201365	4	+	ESC2	ORF-T	ORF-T	-0.101344179	-0.968640684	-0.5552348	-0.025420868	-0.207415172
1202101	1202669	4	+	SEM1	ORF-T	ORF-T	-0.357189915	-1.39563811	-0.569700079	-0.329153113	-0.411949225
1206661	1212981	4	+	unknown	no_ovlp	CUTs	1.154125353	-0.776418073	-0.134834037	-0.155070974	0.61582497
1212981	1213645	4	+	YDR367W	ORF-T	ORF-T	-0.137429843	-0.356190684	0.116723001	-0.265183983	0.434748282
1213861	1214981	4	+	YPR1	ORF-T	ORF-T	-0.094946348	-0.000916956	-0.043400789	0.143073016	0.559725249
1219333	1221053	4	+	CTS2	ORF-T	ORF-T	0.189398835	-0.053654457	0.149431327	-0.021513997	-0.722301021
1222677	1223437	4	+	FRQ1	ORF-T	ORF-T	-0.337639391	-0.537871203	0.107203639	-0.161235189	0.505649729
1224741	1225229	4	+	YDR374W-A	ORF-T	ORF-T	0.175336408	0.349708877	0.66928598	0.098375419	0.677138032
1226781	1228349	4	+	ARH1	ORF-T	ORF-T	-0.682190638	-0.410853093	0.307091228	0.012925042	-0.042564997
1228533	1229293	4	+	ATP17	ORF-T	ORF-T	-0.496856782	-0.654474266	0.118176499	-0.185612071	-0.089335589
1229397	1230093	4	+	unknown	no_ovlp	SUTs	0.491302168	0.31876666	-0.334097641	-0.451681928	-0.457089089

1230093	1233205	4	+	RGA2	ORF-T	ORF-T	-0.399063688	-0.534580216	0.020979764	-0.231394707	0.037654013
1234117	1236245	4	+	ARO10	ORF-T	ORF-T	0.31891713	-0.771993838	-0.157013655	0.037449975	0.441082278
1236485	1236829	4	+	YRA1	ORF-T	ORF-T	0.170033484	-0.658370976	-0.184821059	0.119212716	-0.349843324
1239397	1239997	4	+	RPP2B	ORF-T	ORF-T	-0.148300353	-0.035051321	-0.169124456	-0.225739303	-0.218453805
1240101	1241053	4	+	CUT088	CUTs	CUTs	3.199979861	-0.200660119	-0.951811639	-0.672067832	-0.534646882
1242741	1243165	4	+	CUT089	CUTs	CUTs	0.901886259	-0.800880196	1.279898988	0.652937515	-0.240319839
1243165	1245965	4	+	EFT2	ORF-T	ORF-T	0.267694024	0.044732332	-0.368987127	-0.334330138	-0.750574207
1245965	1248021	4	+	MUS81	ORF-T	ORF-T	-0.371853228	-0.331924972	0.462018215	-0.189769333	0.793724238
1250141	1251749	4	+	RVS167	ORF-T	ORF-T	-0.432728446	-0.340259392	-0.23838749	-0.156691629	-0.338945415
1252005	1254637	4	+	SAC7	ORF-T	ORF-T	0.087743496	-0.175946294	-0.467994535	-0.173727229	-0.027524596
1258325	1258653	4	+	unknown	no_ovlp	SUTs	-0.703160338	-1.093184544	0.226260498	-0.552441506	-0.764831349
1258653	1259845	4	+	unknown	no_ovlp2	SUTs	-0.2875689	-0.75164158	-0.005125418	0.034549057	0.396342566
1259845	1261381	4	+	SHE9	ORF-T	ORF-T	0.095340776	-0.041620779	-0.256669498	-0.202269178	-0.086764775
1261573	1263053	4	+	RPT3	ORF-T	ORF-T	-0.045729738	-0.483911003	-0.022896758	-0.03419335	-0.268104743
1263221	1266285	4	+	SXM1	ORF-T	ORF-T	0.028811731	-0.449034507	-0.53715402	-0.156379644	0.042336553
1267045	1267445	4	+	unknown	no_ovlp	SUTs	0.938962217	-0.119236204	0.515911117	-0.208581862	0.527441423
1267445	1269517	4	+	UTP5	ORF-T	ORF-T	0.442482457	0.052209791	-0.905160898	-0.296309066	-0.00283233
1269989	1270837	4	+	HPT1	ORF-T	ORF-T	0.801917623	-0.302244533	-0.543781442	-0.334739114	0.230804494
1270837	1272269	4	+	URH1	ORF-T	ORF-T	-0.173080732	0.196472197	-0.17203307	0.119073325	-0.286172822
1272269	1275381	4	+	unknown	no_ovlp2	SUTs	-0.201272664	0.410626791	1.148244655	-0.062935179	-0.112416866
1275381	1277573	4	+	DIT1	ORF-T	ORF-T	-0.576304369	1.181813954	2.196294978	1.218334173	-0.458894802
1276309	1277573	4	+	unknown	no_ovlp2	SUTs	0.220173271	0.413657045	0.011602287	0.194243033	-0.258066944
1277573	1278573	4	+	MRP20	ORF-T	ORF-T	-0.676796446	-0.647684417	0.328289286	-0.136089348	-0.061755235
1279781	1283845	4	+	PDR15, YDR4	ORF-T	ORF-T	-0.741866717	-0.238682248	0.898337745	0.481521407	0.184460132
1288245	1288997	4	+	unknown	no_ovlp	SUTs	0.557135787	-0.007712251	0.454040046	-0.174011237	1.101291788
1289381	1292453	4	+	SIZ1	ORF-T	ORF-T	-0.4279563	-0.387717547	-0.120759735	-0.155216917	-0.038723678
1294637	1295541	4	+	RRP17	ORF-T	ORF-T	0.228303883	-0.654662971	-0.724892572	0.022103301	0.548436839
1297021	1297637	4	+	unknown	no_ovlp	CUTs	1.296169918	-0.473119457	-0.76574834	-0.030141586	0.61288804
1298365	1301037	4	+	SYF1	ORF-T	ORF-T	-0.121661857	-0.605807952	-0.238419902	-0.119079946	0.086140106
1301557	1302213	4	+	RPL12B	ORF-T	ORF-T	-0.445946146	-0.133102585	-0.181090997	-0.106623335	0.099985247
1303165	1305309	4	+	RAD30	ORF-T	ORF-T	0.282043848	0.008701313	-0.176946529	-0.267723185	-0.259263192
1305989	1311837	4	+	HKR1	ORF-T	ORF-T	0.030934176	-0.276608887	-0.381177809	-0.058658939	-0.240570158
1312029	1314965	4	+	ARO80	ORF-T	ORF-T	-0.332075213	-0.558301705	0.421454792	-0.034071254	0.856357665
1319437	1319757	4	+	CUT092	CUTs	CUTs	3.325798464	-0.381254406	-0.532099735	-0.654139864	-0.659957926
1320021	1321053	4	+	SNX41	ORF-T	ORF-T	0.254974433	0.158523692	0.90533353	0.179158841	0.169846696
1322173	1323461	4	+	RPN9	ORF-T	ORF-T	0.195688036	-0.553681044	0.034617954	-0.037650668	-0.228498965
1328749	1330365	4	+	NPL3, YDR43	ORF-T	ORF-T	0.068062627	0.082156057	-0.373190168	0.065590518	-0.441791096
1331149	1332941	4	+	GPI17	ORF-T	ORF-T	-0.599490718	-0.51911	-0.397659535	0.076951105	-0.212716185
1334205	1334573	4	+	unknown	no_ovlp2	CUTs	2.069222182	-0.315585307	-0.072727452	-0.073633075	0.014598765
1334573	1336981	4	+	PPZ2	ORF-T	ORF-T	-0.397102286	-0.41109716	0.240163492	0.212399237	-0.304955565
1337301	1340869	4	+	GPI19, THI74	ORF-T	ORF-T	0.424135108	-0.01031323	-0.060879648	0.025685401	-0.033901235
1342405	1344269	4	+	DOT1	ORF-T	ORF-T	-0.213473362	-0.391381245	-0.512776443	-0.318787517	0.20429394
1350197	1352413	4	+	YDR444W	ORF-T	ORF-T	-0.002237432	-0.437555684	-0.497994333	-0.223721453	0.117644707
1356005	1357381	4	+	ADA2	ORF-T	ORF-T	-0.141270444	-0.153048792	-0.225952831	-0.378501704	0.15341673
1359141	1359893	4	+	CUT095	CUTs	CUTs	0.984618513	0.29526182	-0.285228309	-0.474192196	-0.768963201
1359965	1360413	4	+	RPS18A	ORF-T	ORF-T	0.363899006	-0.167264288	-0.410368854	-0.24018697	-0.17600612
1361045	1362741	4	+	unknown	no_ovlp	SUTs	-0.022867979	0.448166866	1.061428419	0.764757686	0.887939143
1362741	1365061	4	+	PPN1	ORF-T	ORF-T	-0.484602596	-0.417237536	0.213603737	0.089325817	0.566847029
1367445	1369485	4	+	NHX1	ORF-T	ORF-T	-0.480984961	-0.57622185	-0.009361805	0.068348176	0.486485252
1369693	1372109	4	+	TOM1	ORF-T	ORF-T	-0.006320696	-0.464544217	-0.539493649	-0.090554243	-0.259842817
1382253	1382581	4	+	unknown	no_ovlp	CUTs	2.261278209	-0.03548206	-0.676668466	-0.143036684	0.131773312
1383677	1384869	4	+	TFB3	ORF-T	ORF-T	-0.03070021	-0.083231459	0.4107027	-0.210293252	0.475564658
1385125	1385429	4	+	MFA1	ORF-T	ORF-T	0.733704627	0.100460901	-0.738149466	0.613089883	-1.361458085
1385589	1386029	4	+	unknown	no_ovlp	SUTs	0.053685212	0.382205633	0.380040939	0.435268027	0.427966112
1386029	1386637	4	+	MRPL28	ORF-T	ORF-T	-1.009199075	-0.442884034	0.907295151	0.245898485	0.017026602
1386781	1388573	4	+	STP1	ORF-T	ORF-T	0.244671102	-0.281589276	0.071267342	-0.221808437	0.201814096
1388789	1390605	4	+	SPP41	ORF-T	ORF-T	-0.117376163	-0.38890437	-0.53017284	-0.304429718	0.490142862
1395021	1397981	4	+	PKH3	ORF-T	ORF-T	0.050058671	-0.329726578	-0.024896826	0.07658845	0.149320425
1398997	1399813	4	+	SDC1	ORF-T	ORF-T	0.082473668	-0.548753412	-0.265158806	-0.494296946	0.199183897
1401789	1402181	4	+	RPL27B	ORF-T	ORF-T	0.39914612	-1.037090779	-1.03623213	-0.850248422	-0.009327783
1403101	1403397	4	+	unknown	no_ovlp	CUTs	1.525127066	-0.438455584	-0.88496645	-0.383016821	0.131340915
1406085	1408741	4	+	SUT075	SUTs	SUTs	1.692833029	-0.389876355	-0.716348566	-0.385389112	-0.609066481
1410493	1411949	4	+	SUT076	SUTs	SUTs	1.422628819	-0.572274381	-0.16504857	-0.146973258	0.093882645
1412085	1414397	4	+	SNF1	ORF-T	ORF-T	-0.475537273	-0.612675483	-0.088474881	0.098568511	-0.205836974
1414541	1416237	4	+	SNM1	ORF-T	ORF-T	0.025458151	-0.303235558	-0.465457213	-0.017505859	0.535839406
1417125	1417333	4	+	unknown	no_ovlp2	SUTs	-0.074544511	-1.139131006	0.329394226	-0.100068992	-0.471005246
1417333	1418517	4	+	DIG2	ORF-T	ORF-T	-0.691848344	-0.651554579	0.338562124	0.174261981	0.621738314
1420429	1420717	4	+	unknown	no_ovlp	SUTs	0.894460151	0.03754143	-0.022865155	-0.100897813	-0.070846707
1420733	1421117	4	+	unknown	no_ovlp	CUTs	1.009412719	0.154278331	0.333893041	0.043261069	0.134548304
1421117	1422629	4	+	KRE2	ORF-T	ORF-T	-0.038030719	-0.316652826	-0.435455874	0.01507492	-0.337277129
1422741	1424773	4	+	VPS52	ORF-T	ORF-T	0.237610175	-0.457437421	0.072330032	0.003598354	-0.037539607
1427413	1427853	4	+	CUT098	CUTs	CUTs	2.825478933	0.482711072	-0.30903002	-0.143980087	0.429818405
1430997	1432157	4	+	SLD5	ORF-T	ORF-T	0.767457745	-0.819054487	-0.122795295	-0.233162021	0.329076856
1434773	1435941	4	+	IZH1	ORF-T	ORF-T	0.095954009	-0.132501217	-0.791601439	0.042926644	1.032989473
1436133	1436677	4	+	FMP36	ORF-T	ORF-T	-0.132480656	0.026904693	-0.103822934	-0.146135519	-0.815464423
1436925	1438085	4	+	RSM28	ORF-T	ORF-T	-0.643004306	-1.130428272	-0.041338594	-0.36693584	-0.098729982
1445853	1446861	4	+	unknown	no_ovlp	CUTs	1.844638401	-0.553131795	-1.448020406	-0.049150484	-0.105074998
1447845	1448349	4	+	LCD1	ORF-T	ORF-T	0.405415359	0.146180907	-0.348525566	-0.488440172	0.479251041
1451325	1453069	4	+	PLM2	ORF-T	ORF-T	-0.515823803	-0.354867781	1.015155397	0.405834293	1.270955371
1454957	1456677	4	+	SUT078	SUTs	SUTs	0.862755201	0.080435477	-0.837687334	-0.437628927	0.618770621
1459749	1460637	4	+	SUT079	SUTs	SUTs	2.867066577	-0.468302124	-1.986132533	-1.034250385	-1.00258292
1469317	1469845	4	+	SMT3	ORF-T	ORF-T	-0.834166696	-0.807043314	0.237593511	0.04674765	0.075313013

1469997	1470437	4	+	ACN9	ORF-T	ORF-T	-0.380391674	-0.051101348	0.532142146	-0.100995563	-0.002253882
1471005	1471541	4	+	GRX2	ORF-T	ORF-T	-0.50605823	-0.795361076	0.834404272	0.275297422	0.311304824
1473221	1474837	4	+	SLF1	ORF-T	ORF-T	-0.150861291	-0.2367125	0.138982037	-0.468829047	-0.033877435
1476853	1477157	4	+	unknown	no_ovlp	SUTs	-0.134161193	-0.574844949	-0.688436059	-0.559379005	-0.215243849
1477157	1478301	4	+	GRH1	ORF-T	ORF-T	-0.523299597	-0.562537188	0.061006569	0.100384046	-0.20894734
1478301	1478573	4	+	unknown	no_ovlp2	SUTs	-0.617710246	-0.723204681	-0.478781152	0.245700932	-0.430928064
1478573	1480213	4	+	EUG1	ORF-T	ORF-T	-0.96866773	-0.419481624	-0.156837238	0.3192888	0.16135657
1480373	1481109	4	+	FPR2	ORF-T	ORF-T	-0.736123388	-0.38907144	0.009785334	-0.136964996	0.151022434
1483725	1485501	4	+	CUT100	CUTs	CUTs	1.620116506	-0.036810979	-0.730433628	-0.255969734	-2.267215327
1487581	1490517	4	+	SUT082	SUTs	SUTs	0.207607462	0.455440792	0.184872951	0.538979242	0.050069244
1490517	1490869	4	+	SNA2, RBA5	ORF-T	ORF-T	0.032052952	-0.011642242	0.562256467	-0.146261629	-0.147531278
1493453	1493773	4	+	unknown	no_ovlp	CUTs	2.058596193	0.384852571	0.602290549	0.015302471	0.616429174
1494285	1496061	4	+	HLR1	ORF-T	ORF-T	-0.302880399	0.159871792	-1.364866218	-0.791714217	-0.202190616
1496805	1497157	4	+	CUT101	CUTs	CUTs	2.285152429	0.118264074	-0.256411833	-0.036419998	0.640000744
1498197	1499429	4	+	YDR531W	ORF-T	ORF-T	0.758418524	0.238398455	-0.847058584	-0.340405243	-0.758735896
1500709	1501613	4	+	SUT083	SUTs	SUTs	1.35157394	0.116026558	0.510871446	-0.462701228	-0.407184667
1509981	1510853	4	+	PAD1	ORF-T	ORF-T	-0.59281688	-0.438173714	0.961759615	0.873619884	-0.685978771
1511853	1513693	4	+	YDR539W	ORF-T	ORF-T	0.212988455	0.958699406	0.241034942	0.033147776	-1.413585186
1518085	1518789	4	+	YDRWdelta3	other	other	-0.744419177	-1.241865085	0.067246905	0.460952563	-1.57559927
1521157	1522061	4	+	SUT085	SUTs	SUTs	0.471259227	-0.107256883	-0.000308335	-1.569483854	-1.993991915
6541	7573	5	-	unknown	no_ovlp	SUTs	-0.179459313	-0.790788643	0.032540642	0.055890185	-4.365096175
8461	9765	5	-	SUT500	SUTs	SUTs	0.199322373	-0.600072409	-0.046584249	-0.093976406	-1.522399538
15613	15981	5	-	SUT503	SUTs	SUTs	3.748457693	-0.534524581	-0.010705089	-0.035360409	-1.173853716
25445	25957	5	-	YEL068C	other	other	-0.358556329	-0.464427369	-0.079384157	0.083961881	-0.198512213
25957	27181	5	-	YEL067C	ORF-T	ORF-T	-0.504670249	-0.107744991	1.00090955	1.007077767	0.729413146
26933	27181	5	-	unknown	no_ovlp2	SUTs	0.863000004	0.896163823	0.788882652	0.326881757	1.430101598
29557	31437	5	-	AVT2	ORF-T	ORF-T	-0.447483668	-0.307864128	0.37648661	-0.051323496	0.876317215
33349	33637	5	-	CAN1	ORF-T	ORF-T	0.217653301	-0.29915502	-0.959141674	-0.548219156	-1.049564215
33637	34445	5	-	unknown	no_ovlp	CUTs	1.109574816	-0.163534803	0.098071918	-0.868590304	-1.397238675
36317	39557	5	-	CIN8	ORF-T	ORF-T	-0.210854925	-0.50766327	-0.35120255	-0.177090397	0.474749244
39853	42013	5	-	PRB1	ORF-T	ORF-T	0.018141179	0.022353838	0.042017588	0.296670591	0.013847397
42357	42701	5	-	SOM1	ORF-T	ORF-T	0.316054819	0.584369997	0.699681739	-0.143984015	0.205715695
42701	43069	5	-	unknown	no_ovlp	SUTs	0.999012502	0.470161936	-0.509829374	-0.322483115	-1.365006613
44653	44901	5	-	unknown	no_ovlp2	CUTs	1.590992913	0.479107706	-0.850265729	0.248119582	0.575734705
44901	48309	5	-	YEL057C	ORF-T	ORF-T	1.368865269	1.405051923	2.315461219	2.207514283	-1.369935218
45853	48309	5	-	unknown	no_ovlp2	SUTs	0.476810008	0.173446055	0.232857524	-0.141253074	0.742799474
48309	51781	5	-	POL5	ORF-T	ORF-T	-0.111930504	-0.673994622	-0.500230718	-0.081556194	0.131482628
52133	52357	5	-	unknown	no_ovlp	CUTs	2.280579465	-1.01076788	-0.505026959	-0.262249879	-0.476813393
52485	53277	5	-	RPL12A	ORF-T	ORF-T	-0.280254657	-0.338740492	-0.310756785	-0.323987656	-0.029557038
53853	56157	5	-	MAK10	ORF-T	ORF-T	0.01982124	-0.351721553	-0.492188792	-0.433859091	-0.166436926
58861	59173	5	-	CUT545	CUTs	CUTs	3.753641315	-0.133978087	-1.202519858	-0.365086806	0.204582985
59549	60901	5	-	RML2	ORF-T	ORF-T	-0.852139963	-0.800613891	-0.073536869	-0.20509701	-0.253548259
64197	65189	5	-	YEL048C	ORF-T	ORF-T	0.59014268	-0.503986535	-0.654066624	-0.416028916	-0.125074754
65381	66821	5	-	YEL047C	ORF-T	ORF-T	-0.23725361	-0.316655801	-0.074801599	0.557254744	0.066689121
67045	68909	5	-	GLY1, YEL04	other	other	-0.0673968	-0.309330442	-0.604753978	-0.01201473	-0.320712665
68909	70213	5	-	unknown	no_ovlp	CUTs	1.861893968	0.521496726	-0.235075177	-0.537340287	-0.210979457
75837	77453	5	-	unknown	no_ovlp	CUTs	1.084603315	0.051435651	-0.542472515	-0.035248036	0.037496569
79549	80061	5	-	CYC7	ORF-T	ORF-T	-0.014242596	-0.659722443	1.909604701	1.158465433	0.476151003
81261	82765	5	-	RAD23	ORF-T	ORF-T	-0.284423279	-0.591073989	-0.570283272	-0.155609337	-0.575192008
82981	84701	5	-	ANP1	ORF-T	ORF-T	-0.353228457	0.009323627	-0.575310971	-0.141485198	0.333653577
84701	86341	5	-	unknown	no_ovlp	SUTs	0.652758179	0.116507267	-0.112356472	0.044788626	-0.59333267
89189	89965	5	-	unknown	no_ovlp	SUTs	0.854732786	0.219350544	-0.388185546	0.081578856	0.237239501
96805	97861	5	-	BUD16	ORF-T	ORF-T	0.161791668	-0.599685132	-0.733448147	-0.347680947	-0.348519449
97861	98365	5	-	unknown	no_ovlp2	SUTs	0.293545164	0.187621736	-0.532275691	-0.577271932	-0.277099859
98365	102397	5	-	YELCdelta3	other	other	0.970277884	0.110771402	-0.071760365	0.326065064	0.173967165
99501	102397	5	-	unknown	no_ovlp2	SUTs	-0.147023339	0.210238991	0.021555385	-0.024282827	0.150304499
106149	106885	5	-	YEL025C	ORF-T	ORF-T	-0.046775801	0.039145709	0.556731381	-0.398817728	1.68462709
108501	111101	5	-	YEL023C	ORF-T	ORF-T	0.056613577	-0.104117757	0.124291718	-0.310876723	-0.93841108
115413	115941	5	-	unknown	no_ovlp	CUTs	1.467856644	0.593232678	0.780944061	0.656998066	0.957394293
117669	118117	5	-	RPR1	other	other	0.852454737	0.47543845	-0.177788248	-0.28954837	0.098866659
118541	121301	5	-	YELCtau1, YE	other	other	-0.032199516	-0.139566246	0.654328116	0.109395301	1.050690032
120381	121301	5	-	unknown	no_ovlp2	SUTs	0.178165722	0.414022134	0.305935455	-0.56715681	0.856375437
122325	122997	5	-	PMP2	ORF-T	ORF-T	-0.207198976	-0.690332226	-0.088600157	-0.085620752	-0.037881816
124693	126405	5	-	NPP2	ORF-T	ORF-T	-0.587260812	-0.82353796	-0.57319167	-0.537838097	0.036411895
134229	135501	5	-	SUT504	SUTs	SUTs	0.828394992	-1.375556034	-1.947090574	-1.925080374	-0.37058091
138829	140389	5	-	GCN4	ORF-T	ORF-T	-0.192756979	-0.202071386	-0.091918614	-0.019997109	-0.083971235
140389	140981	5	-	YEL008C-A	other	other	1.086798878	0.238652368	-0.263843841	-0.400257466	-0.565617319
145845	146773	5	-	VAB2	ORF-T	ORF-T	1.064652821	0.497933917	0.218760298	0.149996233	-0.881183684
146773	147957	5	-	unknown	no_ovlp	SUTs	0.904233181	-0.181850051	-0.020187005	0.233943906	-0.330149269
148829	150077	5	-	WBP1	ORF-T	ORF-T	-0.394763348	-0.155714753	-0.195177366	-0.063252097	0.011716096
150221	151005	5	-	IRC22	ORF-T	ORF-T	-0.229199301	-0.277149214	-0.245418986	-0.176713528	-0.529880186
152157	152637	5	-	CUT547	CUTs	CUTs	2.711073551	0.142799173	0.327871359	-0.228758479	-0.471835186
156021	156493	5	-	SUT506	SUTs	SUTs	2.340114385	0.184082305	0.166339263	-0.135568261	-0.445836487
157661	158997	5	-	PMI40	ORF-T	ORF-T	-0.430728723	-0.199985253	-0.53273117	-0.114606138	-0.049054044
161421	162437	5	-	SUT507	SUTs	SUTs	0.61121476	-0.583282337	-0.816623792	-0.478205997	0.957382872
164629	166013	5	-	unknown	no_ovlp	CUTs	1.029873219	0.218417346	-0.523437177	-0.576205584	-0.020942051
166189	166765	5	-	TMA20	ORF-T	ORF-T	0.248308322	-0.364128991	-0.399314427	-0.270197567	-0.619765172
167325	167589	5	-	unknown	no_ovlp	CUTs	2.085220543	0.436913055	-0.16463648	0.234079192	-0.482421889
167733	171877	5	-	SEC3	ORF-T	ORF-T	-0.248669871	-0.417481591	-0.476822965	-0.130585452	-0.433832936
172981	173357	5	-	YER010C	ORF-T	ORF-T	0.349285759	0.397372004	0.761477529	0.162589694	-0.277024371
177189	177573	5	-	SUT508	SUTs	SUTs	1.431949429	0.974712365	-0.256171997	-0.404939225	-0.203407946
178149	178573	5	-	CUT550	CUTs	CUTs	3.439797128	0.3530442	-0.944544046	0.147988453	0.397299767

184349	186149	5	-	unknown	no_ovlp	SUTs	0.295844462	-0.736309712	-1.343958602	-1.464340012	-1.405368188
186957	188069	5	-	SUT509	SUTs	SUTs	0.118807665	-0.951982353	0.11436051	-0.218038778	0.218057174
189373	191877	5	-	AFG3	ORF-T	ORF-T	-0.349452908	-0.38675706	0.100861201	0.149321125	0.096571377
191877	192669	5	-	SPC25	ORF-T	ORF-T	-0.334653735	-0.148058231	-0.083003775	-0.318098829	-0.016250745
194197	194653	5	-	SBH2	ORF-T	ORF-T	-0.477266999	-1.098248163	-0.114384647	0.354088365	-0.112933495
194653	195333	5	-	SUT511	SUTs	SUTs	-0.884642621	-0.530486602	1.012695327	0.421302961	0.949441362
201405	204973	5	-	SUT512	SUTs	SUTs	1.623687211	0.201007477	-1.425607272	-0.598376857	0.344615747
207517	208493	5	-	CHO1	ORF-T	ORF-T	-0.415496981	-0.288841121	-0.150014974	0.201154684	-0.154254956
208893	210341	5	-	GAL83	ORF-T	ORF-T	-0.269704591	-0.344830675	-0.1969784	0.023096179	-0.118192051
210693	211989	5	-	MIG3	ORF-T	ORF-T	-0.031878455	-0.695845054	-0.27964832	-0.140253426	-0.530161101
212485	213237	5	-	SMB1	ORF-T	ORF-T	-0.067216851	-0.299818469	0.00632932	0.064871326	0.321619777
213901	214781	5	-	YPT31	ORF-T	ORF-T	-0.157131494	-0.269940371	-0.144947499	-0.146065005	0.384711505
214781	216565	5	-	unknown	no_ovlp	SUTs	-0.10639127	0.214354536	-0.077291533	0.131845277	0.373644728
217877	221373	5	-	ZRG8	ORF-T	ORF-T	-0.53010271	-0.664480956	0.067520667	0.420038608	-0.083904987
222501	223149	5	-	CUT551	CUTs	CUTs	2.202333197	-0.046420308	0.354488249	0.068556327	0.383409648
223269	225285	5	-	ARB1	ORF-T	ORF-T	0.120771223	-0.519218729	-0.834824652	-0.222761476	-0.363143737
226101	228189	5	-	KRE29	ORF-T	ORF-T	0.71431327	0.093171984	-0.078987221	-0.370986047	-0.716426673
228189	230181	5	-	HVG1, YER03	other	other	-0.526290879	-0.470199723	0.31508462	-0.475813176	1.051834106
229509	230181	5	-	unknown	no_ovlp2	SUTs	-0.256353289	0.351639827	0.006201417	-0.005376788	0.044184948
235541	237173	5	-	SAH1	ORF-T	ORF-T	-0.598185132	-0.003288903	-0.47235164	-0.030088751	-0.147659885
237453	238085	5	-	ERG28	ORF-T	ORF-T	0.240161201	-0.657744536	-0.311468942	0.202216104	-0.133277349
238085	238413	5	-	MEI4	ORF-T	ORF-T	1.102731399	0.588983673	0.911514466	0.418745144	0.755609049
240493	241709	5	-	ACA1	ORF-T	ORF-T	1.331142485	0.555579684	0.687006033	0.98121352	-2.360474383
241709	242709	5	-	SUT513	SUTs	SUTs	-0.117949317	-0.565637591	0.263991832	0.442874399	-0.236030564
243725	246701	5	-	SAP1	ORF-T	ORF-T	-0.342820152	-0.507195339	0.100115343	0.221440243	0.027217571
246837	248221	5	-	CAJ1	ORF-T	ORF-T	-0.25111819	-0.829936316	-0.398530958	-0.156453959	-0.08333182
248597	249781	5	-	YERCdelta8	other	other	1.471631594	1.249246994	-0.77265214	0.04928341	-3.587361135
253869	254445	5	-	RSM18	ORF-T	ORF-T	0.024204768	-0.842461667	-0.278329598	-0.252891068	-0.285951992
254445	254917	5	-	unknown	no_ovlp	CUTs	1.06198906	0.576622842	0.233533162	-0.660984004	0.904700517
256245	258053	5	-	HOM3	ORF-T	ORF-T	0.074748153	-0.79311278	-1.327314184	-0.353229648	-0.622368695
258589	259789	5	-	PIC2	ORF-T	ORF-T	0.639689727	-0.798499535	0.130075502	0.010863407	-0.831892721
259789	260005	5	-	unknown	no_ovlp	CUTs	1.12037338	0.289822394	-0.515803904	-0.93111386	-1.064874585
260597	261125	5	-	YER053C-A	ORF-T	ORF-T	0.185342035	-1.005855998	0.336003123	0.100049358	0.077764849
261125	262021	5	-	unknown	no_ovlp2	CUTs	1.161372052	0.000301485	-0.173601916	-0.377513511	-1.199233762
262021	264173	5	-	GIP2	ORF-T	ORF-T	-0.927576352	-0.703144125	2.108069805	1.999120431	1.181831479
264765	265789	5	-	HIS1	ORF-T	ORF-T	0.09239232	-0.534369181	-1.331844919	-0.413473345	-0.723156808
266397	268197	5	-	FCY2	ORF-T	ORF-T	0.054626659	-0.372778303	-0.762309075	-0.389634756	0.082995287
269749	270181	5	-	RPL34A	ORF-T	ORF-T	0.622503625	-0.334217279	-0.909417473	-0.768809445	-0.720783653
270645	271189	5	-	HMF1	ORF-T	ORF-T	0.081793231	0.768272564	0.400730344	0.16693419	0.372398942
275069	279621	5	-	unknown	no_ovlp	CUTs	1.214955459	0.295775828	-0.301439358	-0.532374582	-0.780723003
279773	280797	5	-	HOR2	ORF-T	ORF-T	0.22487922	0.64451175	0.617406077	0.346635978	0.714940124
282453	284317	5	-	YER064C	ORF-T	ORF-T	-0.161719263	-0.764522767	-0.559218462	-0.382079921	-0.104085975
285141	287221	5	-	ICL1	ORF-T	ORF-T	0.815049507	0.103342724	0.960276439	0.63396433	-0.349128853
288685	289333	5	-	unknown	no_ovlp	CUTs	1.208896871	0.667633178	-0.191775136	0.262448852	-0.464349491
290869	291533	5	-	YER066C-A	other	other	0.399188808	-0.785243902	0.787652145	0.99099194	-0.829504886
294445	294821	5	-	CUT558	CUTs	CUTs	3.215195922	-0.084369153	-1.577231666	-0.460238046	-2.386847254
301725	302637	5	-	YER071C	ORF-T	ORF-T	0.758741968	-0.00355499	0.084259333	-0.220850078	-0.649160438
302365	302637	5	-	unknown	no_ovlp2	CUTs	2.265670839	-0.498084363	-1.254038291	0.082151118	-0.883110509
302637	303261	5	-	CUT561	CUTs	CUTs	1.057405939	-1.822992959	-0.543323528	-0.352248746	-0.955599835
308269	311341	5	-	PTP3	ORF-T	ORF-T	0.68852377	-0.273122757	-0.440437575	-0.018735253	0.432542155
312277	314509	5	-	YER076C	ORF-T	ORF-T	0.172825541	-0.097136354	0.172489904	-0.043789941	-0.733878837
313821	314509	5	-	unknown	no_ovlp2	CUTs	1.328550074	-0.004334989	0.101546576	0.94378077	-1.193390154
314509	316541	5	-	YER077C	ORF-T	ORF-T	0.019178675	-0.364314762	-0.125998131	-0.405315558	-0.006344251
316749	318533	5	-	YER078C	ORF-T	ORF-T	-0.077746868	-0.152579791	-0.094032996	0.257725161	0.257384803
319325	319741	5	-	CUT562	CUTs	CUTs	2.09942733	-0.049993379	-1.409942654	-0.994911444	-0.664646081
321485	321877	5	-	CUT563	CUTs	CUTs	1.969465213	0.285897038	0.272276629	0.64745317	1.30952189
324189	325981	5	-	UTP7	ORF-T	ORF-T	0.285523422	-0.053410826	-0.515121519	0.016470955	0.173345357
326165	327061	5	-	GET2	ORF-T	ORF-T	0.135599336	-0.282264682	0.111268288	-0.281029337	0.271492684
327061	327333	5	-	unknown	no_ovlp	SUTs	-0.309168598	0.521072469	-0.002514148	-0.016359599	0.059679731
332365	332869	5	-	SBH1	ORF-T	ORF-T	-0.327337043	0.122011161	0.045504498	-0.363296584	0.310006224
333045	335277	5	-	DOT6	ORF-T	ORF-T	-0.2359874	-0.195509962	-0.193312952	-0.205811334	0.039908192
335837	337653	5	-	YER088C-A, I	ORF-T	ORF-T	-0.216431082	-0.410352983	-0.507734514	-0.180950698	-0.831494448
339637	342229	5	-	MET6	ORF-T	ORF-T	-0.423421783	0.103230728	-0.496831432	-0.028957701	0.263614506
343213	347661	5	-	TSC11	ORF-T	ORF-T	-0.080330778	-0.262575857	-0.071007289	-0.205972662	0.037171421
347821	348613	5	-	YER093C-A	ORF-T	ORF-T	-0.820588343	-0.603334783	0.421928832	0.148786837	0.426644808
348405	348613	5	-	unknown	no_ovlp2	SUTs	0.424887421	0.761859904	0.46494019	0.03073013	0.072685478
348613	349445	5	-	PUP3	ORF-T	ORF-T	-0.079221617	-0.038994272	0.311877846	-0.010541156	0.247650944
349445	350117	5	-	unknown	no_ovlp	SUTs	-0.001593528	0.486390525	-0.040128163	-0.114838558	0.568597347
358061	359085	5	-	PRS2	ORF-T	ORF-T	0.095835717	-0.496020921	0.154984831	0.155756805	0.214497571
360437	362181	5	-	AST2	ORF-T	ORF-T	-0.246960388	-0.234480487	0.980221246	-0.163287562	0.49283002
361853	362181	5	-	unknown	no_ovlp2	CUTs	1.122143311	-0.261479733	-0.093498283	-0.458824651	0.151046002
363125	364293	5	-	SUT515	SUTs	SUTs	0.479747904	0.370394741	0.59895418	0.441881626	-0.255234592
367605	372037	5	-	NUP157	ORF-T	ORF-T	-0.015194846	-0.422555976	-0.673824378	-0.069115217	-0.181159555
373341	374725	5	-	GLE2	ORF-T	ORF-T	-0.284880454	-0.723830914	-0.442793641	-0.191349593	-0.022752247
375005	377765	5	-	FLO8	ORF-T	ORF-T	-0.220366797	-0.697094069	-0.39236843	-0.597276594	0.207110119
378613	382237	5	-	KAP123	ORF-T	ORF-T	-0.356917499	-0.206006024	-0.672965543	0.155964023	0.485501212
382445	386181	5	-	SWI4	ORF-T	ORF-T	0.088433808	-0.312044222	-0.050790651	-0.172224914	0.270768982
387845	390029	5	-	TMN3	ORF-T	ORF-T	-0.091929197	-0.422635474	-0.431872176	-0.133464265	0.087734282
390605	394013	5	-	BOI2	ORF-T	ORF-T	-0.241764859	-0.4891121	-0.305504466	0.030491868	0.390059396
394197	396245	5	-	SPR6, SLX8	ORF-T	ORF-T	-0.021957187	-0.185848273	-0.107082909	-0.252293439	0.255590033
397765	399229	5	-	SHO1	ORF-T	ORF-T	-0.213661484	-0.087812384	-0.220754649	-0.33856238	0.709220757
399413	400869	5	-	AVT6, YER11	other	other	-0.512605792	-0.333078532	0.364666188	-0.022106216	0.750831817

402733	404421	5	-	GLO3	ORF-T	ORF-T	-0.074104378	-0.18435933	-0.029206848	0.053149795	0.37060369
406973	409101	5	-	DSE1	ORF-T	ORF-T	-0.081085943	-0.542893523	-0.324900367	-0.162715257	0.371295195
409101	409373	5	-	CUT564	CUTs	CUTs	-0.34917349	-1.140685025	-0.20122474	-0.069721636	-0.276838001
413229	414245	5	-	NSA2	ORF-T	ORF-T	0.661467476	-0.157126245	-0.377120766	-0.483330859	0.137310406
416117	416557	5	-	CUT566	CUTs	CUTs	3.153236217	0.266263613	-0.113287331	-0.127117357	0.352046172
420909	422533	5	-	YER130C	ORF-T	ORF-T	0.919127866	-0.519125222	-0.989397849	-0.840743122	-0.69707593
422533	423005	5	-	unknown	no_ovlp	SUTs	0.864293338	-0.706262511	-0.886157125	-0.878669751	-0.935060149
425197	430701	5	-	PMD1	ORF-T	ORF-T	0.263984842	-0.24616927	-0.357118922	-0.407140027	-0.444978734
430701	431133	5	-	unknown	no_ovlp	SUTs	0.79051896	-0.821231387	-0.652368285	-0.692134608	-0.511267354
431821	432229	5	-	unknown	no_ovlp	SUTs	0.263413883	-0.995323981	0.741940184	0.713906483	-0.166781855
432541	432853	5	-	CUT568	CUTs	CUTs	3.489601171	0.384696501	-0.224949267	-0.366536639	-0.514500619
434965	435597	5	-	unknown	no_ovlp	SUTs	0.068584726	-1.320808997	0.08276093	0.020533202	-0.484140765
437013	437853	5	-	YER134C	ORF-T	ORF-T	0.313874678	-0.392418972	0.116288335	0.036885195	-0.598771486
441285	442373	5	-	YER137C	ORF-T	ORF-T	0.441266592	-0.197022742	0.317428606	-0.36363846	0.793372201
441853	442373	5	-	unknown	no_ovlp2	SUTs	0.842025333	0.867241569	-0.070753277	0.048095403	-0.46985534
442373	442677	5	-	unknown	no_ovlp	SUTs	-0.250595146	0.428748776	0.128926603	0.109060584	0.075915055
449821	451349	5	-	YER139C	ORF-T	ORF-T	-0.567604099	-0.664487442	0.636977502	0.234308848	0.544904693
452589	453117	5	-	unknown	no_ovlp	CUTs	2.021545104	0.286039588	-0.029999168	1.063706506	1.715729504
455061	456045	5	-	MAG1	ORF-T	ORF-T	0.25201005	-0.112091548	0.943771605	-0.283466367	-0.451117878
457693	460229	5	-	UBP5	ORF-T	ORF-T	0.369089577	-0.0728609	-0.205686756	-0.124794786	-0.136860488
460405	461773	5	-	FTR1	ORF-T	ORF-T	-0.00357891	-0.241847111	-0.162964596	0.423932953	0.402369021
462869	464845	5	-	SCC4	ORF-T	ORF-T	-0.423854692	-0.305656953	-0.115295993	-0.259920424	0.092616019
466613	467517	5	-	PEA2	ORF-T	ORF-T	0.327144696	-0.208299957	-0.327583852	-0.087978639	-0.046684393
469629	472485	5	-	UBP3	ORF-T	ORF-T	0.02444432	-0.131697834	-0.338351825	0.07047654	0.12926528
472629	474085	5	-	YER152C, PE	other	other	0.141839489	0.023870089	-0.41164228	-0.077936013	0.50625457
474101	474717	5	-	PET122	ORF-T	ORF-T	-0.023127737	-0.091204137	-0.718976602	-0.476315886	0.246617931
482845	483093	5	-	BEM2	ORF-T	ORF-T	-0.940173342	-1.125747845	0.03984157	0.047502027	-0.766878693
483213	484357	5	-	YER156C	ORF-T	ORF-T	-0.283590071	-0.362321748	-0.497950674	-0.11506092	-0.118026729
489149	490893	5	-	YER158C	ORF-T	ORF-T	-0.158804867	-1.124647017	-0.388735919	0.393657518	-0.504630981
491389	492125	5	-	BUR6	ORF-T	ORF-T	0.098224286	-0.779770679	0.185142365	-0.429169474	-0.531651188
492125	498805	5	-	unknown	no_ovlp	SUTs	0.197136393	-1.257498393	-0.609074697	-0.802704214	-0.90760626
499445	500349	5	-	SPT2	ORF-T	ORF-T	-0.310927744	-0.506238965	-0.33250805	0.015892415	-0.167777598
500661	503085	5	-	RAD4	ORF-T	ORF-T	-0.4482712	-0.299561069	0.444498844	0.425245297	0.792663784
503085	503781	5	-	YER163C	ORF-T	ORF-T	-0.419178814	-0.648944138	0.013342815	-0.50938093	0.459280393
509317	510301	5	-	SUT517	SUTs	SUTs	0.763338731	0.518610666	0.4636202	-0.175196998	1.141457189
516109	517533	5	-	unknown	no_ovlp	SUTs	0.773004456	0.456911896	0.597666225	0.51011297	1.812325815
520973	522685	5	-	CCA1	ORF-T	ORF-T	-0.09928375	-0.568638023	-0.036916948	-0.018806798	0.09175219
525157	526781	5	-	unknown	no_ovlp	CUTs	1.177189134	0.559128407	-0.422715702	-0.013703473	0.107039012
531605	536053	5	-	BRR2	ORF-T	ORF-T	0.197111324	-0.409262399	-0.490965505	0.052890034	-0.136677589
538341	539301	5	-	unknown	no_ovlp2	SUTs	-1.380672392	-1.514356026	-0.107415533	0.087944498	0.685631684
539301	541445	5	-	TMT1	ORF-T	ORF-T	0.883570049	-0.131990798	-0.25168412	-0.107295501	-0.48781515
540373	541445	5	-	unknown	no_ovlp2	CUTs	1.361290154	-0.566428294	-0.191621945	-0.378158646	-0.359528615
544653	545061	5	-	CUT571	CUTs	CUTs	2.491703366	-0.222401867	-0.370030192	-0.107083718	-0.603501129
548533	549221	5	-	SUT521	SUTs	SUTs	0.358651942	0.172130229	0.64462715	0.496110031	-0.065969095
549685	551157	5	-	ISC10, SLO1	ORF-T	ORF-T	0.024746531	-0.331521933	-0.235740201	-0.148556311	-0.034299794
553253	553965	5	-	FAU1	ORF-T	ORF-T	0.327148786	-0.599689518	-0.623715171	-0.247544693	-0.806090724
555685	558997	5	-	YER184C	ORF-T	ORF-T	-0.149943769	-0.753242968	-0.159493233	0.17453109	0.024535869
561485	562813	5	-	YER186C	ORF-T	ORF-T	-0.372029381	-1.308676765	-0.356676723	-0.045002784	-0.667690109
7905	8217	5	+	unknown	no_ovlp	SUTs	0.880627437	-1.006080543	0.372783572	0.487122963	-5.075059102
9977	10409	5	+	CUT103	CUTs	CUTs	2.287025286	-0.516332025	0.173412188	-0.549586193	-2.021016919
13617	14665	5	+	RMD6	ORF-T	ORF-T	1.106928239	-0.247434493	-0.379190145	-0.490933169	-0.579305878
16305	17961	5	+	DLD3	ORF-T	ORF-T	-0.359605323	-0.348009376	-0.458176719	0.066653726	-0.532401405
21537	25497	5	+	unknown	no_ovlp	SUTs	0.92964673	0.28222554	0.300283871	0.564191931	-1.681057666
26137	26689	5	+	HPA3	ORF-T	ORF-T	0.716067951	-0.555310133	-0.25821206	-0.725487846	-0.455385863
27049	27625	5	+	unknown	no_ovlp2	SUTs	0.807797066	0.818718229	0.536456655	0.067349542	0.28745407
27625	29617	5	+	SIT1	ORF-T	ORF-T	-0.778635385	-1.090724195	0.162521655	1.440695057	0.010847189
34057	34393	5	+	CUT104	CUTs	CUTs	0.911508387	0.137830296	0.209854251	-0.113443839	-0.470646494
34393	35809	5	+	NPR2	ORF-T	ORF-T	-0.120920068	-0.780721576	0.10019833	-0.130999369	-0.827858456
39913	41857	5	+	unknown	no_ovlp	SUTs	0.576670493	0.249813489	-0.389786055	-0.406679269	-0.654712898
41969	42441	5	+	unknown	no_ovlp	SUTs	0.282314619	0.049952531	-0.040934835	0.075175638	-0.26283348
42441	43233	5	+	unknown	no_ovlp	SUTs	0.781816827	0.317400815	-0.106327973	-0.045428893	-0.482434109
43233	44977	5	+	PCM1	ORF-T	ORF-T	0.004670996	0.208920958	-0.183642416	-0.280042896	-0.248405467
47121	48457	5	+	HAT2	ORF-T	ORF-T	-0.29853123	-0.936543253	-0.153164551	0.192882573	-0.32864186
56553	58169	5	+	AFG1	ORF-T	ORF-T	-0.091332424	-0.027760311	0.123229226	-0.099833402	0.033715182
58337	59305	5	+	VMA8	ORF-T	ORF-T	-0.374814931	-0.950274281	-0.590466435	0.013134995	-0.103369204
61113	61353	5	+	unknown	no_ovlp	SUTs	0.766124036	-0.900952175	-0.77603424	-0.601290146	0.108768094
61353	61601	5	+	unknown	no_ovlp	CUTs	2.769793549	-0.195958499	-0.23545703	-0.545157873	-0.133556716
61809	62089	5	+	unknown	no_ovlp	CUTs	2.79785025	-0.211639985	-0.958116159	-1.029959736	-1.221565237
69121	69729	5	+	IES6	ORF-T	ORF-T	-0.363089858	0.288292044	-0.213066631	0.181482572	-0.105611489
69729	72001	5	+	unknown	no_ovlp	SUTs	-0.23273229	-0.405695544	-0.0702845	-0.363150971	-0.025663697
72001	72993	5	+	YEL043W	ORF-T	ORF-T	-0.069493603	-0.119969948	-0.322388071	-0.374202343	0.120303826
73585	75473	5	+	GDA1	ORF-T	ORF-T	-0.518800044	-0.451804816	-0.434110774	-0.095933055	0.374960654
78057	79585	5	+	UTR2	ORF-T	ORF-T	-0.175650181	-0.24113549	-1.372238759	-0.522770707	-0.295811847
80441	81177	5	+	UTR4	ORF-T	ORF-T	0.175402051	0.369207112	0.252688443	-0.071365885	0.686534326
85201	85593	5	+	unknown	no_ovlp	CUTs	1.334027531	-0.053215034	-0.57373694	-0.887385108	-0.731997103
85593	86377	5	+	HYP2, YEL03	other	other	-0.385738123	-0.058038888	-0.062666452	-0.105036498	0.090339327
86473	86897	5	+	unknown	no_ovlp	SUTs	-0.300347896	-0.529214033	-0.225477167	-0.384790378	-0.541062679
86897	89961	5	+	MCM3	ORF-T	ORF-T	0.105845075	-0.106485311	-0.862473168	0.036248195	-0.042934272
90233	91057	5	+	SPF1	ORF-T	ORF-T	-0.127159995	0.086185603	-1.220322061	-0.324471716	-0.329877305
94017	95137	5	+	ECM10	ORF-T	ORF-T	-0.461817618	0.279437042	0.938466446	0.047064584	0.148418361
98081	99201	5	+	YEL028W	other	other	0.445506774	-0.7613084	-0.046372632	-0.33444718	0.25463944
100705	101521	5	+	CUP5	ORF-T	ORF-T	-0.189872579	-0.21559331	-0.085406745	0.014304434	-0.045808843

101801	102401	5	+	SNU13	ORF-T	ORF-T	-0.214942347	-0.403286029	-0.562156007	-0.077729872	-0.196035094
106385	107161	5	+	unknown	no_ovlp	SUTs	0.430504398	0.44409051	1.127712878	-0.091394957	1.718603282
107161	108425	5	+	RIP1	ORF-T	ORF-T	-0.346954272	-0.371829553	0.634092621	0.215051007	0.225584765
111337	115881	5	+	GEA2	ORF-T	ORF-T	-0.072743184	-0.425823481	-0.579703707	-0.064698646	0.07246385
116121	117105	5	+	URA3	ORF-T	ORF-T	-0.164492956	0.73606963	-0.509166598	-0.033157534	0.06414607
117153	117585	5	+	TIM9	ORF-T	ORF-T	-0.524505621	-0.857544456	-0.278063162	-0.251985605	-0.425601942
121489	122401	5	+	EAF5	ORF-T	ORF-T	-0.218850812	-0.405107276	0.093448482	-0.25341525	0.375951663
123193	123625	5	+	GTT3	ORF-T	ORF-T	0.179723607	-0.140380294	-0.150944004	-0.528424099	-0.244202677
126601	128385	5	+	EDC3	ORF-T	ORF-T	0.082191432	-0.470796884	0.008857543	-0.02981619	-0.336649871
128585	131001	5	+	VAC8	ORF-T	ORF-T	-0.239692434	-0.519935022	-0.017267431	0.155611147	-0.025811038
131912	132657	5	+	UBC8	ORF-T	ORF-T	-0.350689855	-0.460372208	1.227287589	0.431811203	1.298208067
133105	133905	5	+	GLC3	ORF-T	ORF-T	-0.738118383	-0.624361163	1.16269134	1.63499556	0.075738296
140649	141649	5	+	unknown	no_ovlp	SUTs	0.998729322	-0.081145739	-0.19204495	-0.215732038	-0.111181683
141649	143921	5	+	YEL007W	ORF-T	ORF-T	-0.520924536	-0.583461251	-0.566516405	-0.182122253	-0.417341636
144281	145697	5	+	YEA6	ORF-T	ORF-T	-0.040052513	-0.579182001	-0.405360108	-0.260028516	-0.356714819
146929	148313	5	+	YEA4	ORF-T	ORF-T	1.72622133	1.367883155	-0.197548746	-0.159580817	-2.218324696
148313	148705	5	+	GIM4	ORF-T	ORF-T	0.485527741	-0.669101664	-0.461225795	-0.135629508	-0.198640365
150273	151897	5	+	CUT106	CUTs	CUTs	1.343440168	0.079982911	-0.245644561	-0.23763777	-0.457825938
152889	153473	5	+	unknown	no_ovlp	SUTs	-0.111852877	-0.330946943	-0.046234382	-0.343467093	-0.666464204
153473	156097	5	+	MNN1	ORF-T	ORF-T	-0.057974869	-0.498552454	-1.036748789	-0.192345206	-0.386937652
156753	157593	5	+	NOP16	ORF-T	ORF-T	0.266059876	-0.467880866	-0.604683864	-0.178572705	0.183483455
159585	160537	5	+	FMP52	ORF-T	ORF-T	-0.31863521	-0.15000965	0.368856206	0.022637344	0.742756687
160537	162585	5	+	YND1	ORF-T	ORF-T	0.199909971	-0.185916923	-0.350672712	0.105930209	0.03988419
162697	164345	5	+	NUG1	ORF-T	ORF-T	0.183465059	-0.511649283	-0.782914259	0.101404295	0.177903507
164345	166241	5	+	PAC2	ORF-T	ORF-T	0.55579124	0.38994497	-0.343856078	-0.520048645	-0.428672309
167841	168289	5	+	unknown	no_ovlp	CUTs	1.318108821	0.374249385	-0.216763246	0.590106047	1.490357075
172065	172673	5	+	NTF2	ORF-T	ORF-T	-0.228615584	-0.611205199	-0.193618722	-0.194212187	-0.187215293
175281	175905	5	+	TIR1	ORF-T	ORF-T	-1.392766966	-0.755135905	0.897893934	3.09421131	-0.176531932
176009	177097	5	+	unknown	no_ovlp	SUTs	0.291106063	0.529315344	0.415677146	0.467578445	-0.398573919
177097	177777	5	+	unknown	no_ovlp	SUTs	0.62634609	-0.248336529	0.035005421	-0.500165411	0.045687075
177777	178561	5	+	PRE1	ORF-T	ORF-T	-0.00019393	-0.498627675	0.045732154	-0.224835223	-0.003881269
178841	182361	5	+	PRP22	ORF-T	ORF-T	-0.384171763	-0.677806128	0.006003841	0.142038781	-0.16452522
182649	184217	5	+	HEM14	ORF-T	ORF-T	-0.503659678	0.02272214	0.710740874	0.578394961	0.619519102
184217	184473	5	+	unknown	no_ovlp2	SUTs	-0.524550788	0.248991156	0.535286626	0.411881741	0.336455624
184473	185873	5	+	FAA2	ORF-T	ORF-T	-0.522135202	0.29984676	2.066282143	1.38388695	1.275206215
188265	189425	5	+	BIM1	ORF-T	ORF-T	-0.346968195	-0.348170844	-0.309178018	-0.252976275	0.356485314
192393	192857	5	+	unknown	no_ovlp	CUTs	1.182410093	1.109646881	0.248145171	-0.390348992	0.771381862
192857	194321	5	+	ISC1	ORF-T	ORF-T	0.249698265	0.465205474	-0.049441163	-0.441589967	0.754509123
195113	196689	5	+	GPA2	ORF-T	ORF-T	0.130373314	-0.069579269	-0.026466178	0.374204943	-0.185174934
196897	198649	5	+	RPN3	ORF-T	ORF-T	0.119536604	-0.712440488	0.055466346	-0.216327966	-0.390779682
198801	200969	5	+	SRB4	ORF-T	ORF-T	-0.100651176	-0.404002951	0.123510631	-0.035407958	0.138202021
201049	202089	5	+	PRO3	ORF-T	ORF-T	-0.217987283	-0.103806492	-0.367535669	-0.044613885	-0.147563057
202089	205225	5	+	YAT2	ORF-T	ORF-T	-0.195874869	0.063870532	1.051039187	1.071602985	-0.027100162
205225	207097	5	+	GCD11	ORF-T	ORF-T	-0.115511166	-0.318109083	-0.538514832	0.072676741	-0.230979213
210505	210833	5	+	CUT108	CUTs	CUTs	1.713835985	0.141531883	0.02655864	0.440035108	1.186486173
212321	213433	5	+	unknown	no_ovlp	CUTs	1.902846689	0.400714464	-0.269326359	0.365349487	-0.784751835
213433	214001	5	+	CHZ1	ORF-T	ORF-T	-0.38914102	-0.643313962	-0.364212204	-0.349904962	0.501582744
215001	217961	5	+	FIR1	ORF-T	ORF-T	-0.449927832	-0.217974401	-0.157941125	-0.147091642	0.91329463
221785	222569	5	+	YER034W, EI	other	other	0.436903223	0.540993327	-0.029911918	-0.314167288	0.293605896
223385	225817	5	+	unknown	no_ovlp	SUTs	0.092418037	0.437608197	0.009377535	0.147365927	0.164422202
225817	227073	5	+	PHM8	ORF-T	ORF-T	0.679862682	-0.305702462	0.06829438	0.053522685	-0.343907228
228457	229721	5	+	FMP49	other	other	1.695811529	0.119207771	-0.712919421	-0.557802447	-0.907597247
229449	229721	5	+	unknown	no_ovlp2	SUTs	0.352276505	0.232325965	-0.428594867	-0.263969195	-0.329038632
229721	232121	5	+	GLN3	ORF-T	ORF-T	-0.298312695	-0.400166085	-0.013937196	-0.071260184	0.208978286
233025	234929	5	+	YEN1	ORF-T	ORF-T	0.600797417	-0.037200724	-0.179559497	0.007628651	-0.333127935
234929	235697	5	+	MXR1	ORF-T	ORF-T	0.421258252	0.046953501	0.102271016	-0.085980127	1.353531225
238473	239129	5	+	CUT109	CUTs	CUTs	3.155946197	-0.14287187	-0.559296677	0.447294119	-0.209504364
242545	243641	5	+	SPO73	ORF-T	ORF-T	0.017536884	-0.98523789	0.414507147	0.26146931	-0.194662178
250649	251297	5	+	ISD11	ORF-T	ORF-T	0.117575511	-0.571229357	-0.020150931	-0.107377254	-0.107020219
251737	253753	5	+	TPA1	ORF-T	ORF-T	-0.018102788	-0.395876218	-0.699094493	0.077186528	0.010575252
254649	256353	5	+	JHD1	ORF-T	ORF-T	0.301592808	-0.169264799	0.255497953	-0.313600472	0.380842419
258353	258857	5	+	unknown	no_ovlp	CUTs	1.468012146	-0.122934052	-1.002809299	-1.026492167	0.771744891
261961	262809	5	+	CUT110	CUTs	CUTs	2.365305451	-0.525031086	-0.919004	-0.156981538	-0.4779805
268849	269385	5	+	SUT091	SUTs	SUTs	0.262405082	-0.938745541	-0.391836449	-0.121635349	-0.702574474
270697	271625	5	+	SUT092	SUTs	SUTs	0.878847214	-0.964986692	-0.711624068	-0.906120657	-0.303535188
271737	272081	5	+	PET117	ORF-T	ORF-T	-0.863923015	-0.03262759	0.678492556	-0.368509218	0.624092914
272457	273953	5	+	PCL6	ORF-T	ORF-T	-0.33926966	-0.899694017	-0.026875634	0.105781654	0.070202546
274433	276425	5	+	FCY21	ORF-T	ORF-T	0.176537495	-0.081847598	-0.693444748	0.232830088	0.492678092
277081	278145	5	+	unknown	no_ovlp	SUTs	0.486857868	-0.247393901	-0.72689316	0.074982183	0.441993181
279857	280881	5	+	unknown	no_ovlp	CUTs	1.531222857	0.727937864	-0.231382781	-0.663296862	-0.115597943
281137	281657	5	+	unknown	no_ovlp	SUTs	-0.931593205	-0.657124418	0.887486796	0.042583493	0.021897305
281657	282513	5	+	THO1	ORF-T	ORF-T	-0.882891187	-0.976485175	-0.04525848	0.288354905	-0.1518657
285193	286001	5	+	SUT093	SUTs	SUTs	1.874506748	-0.591837151	-1.07175731	-0.436144217	0.184029667
289345	289625	5	+	YER066W	ORF-T	ORF-T	0.835190032	-0.271472464	-0.134698234	-0.176229023	-0.849032435
292001	292665	5	+	YER067W	ORF-T	ORF-T	-0.763966952	-1.993155436	0.201373464	1.206389611	-0.630234716
292969	294905	5	+	MOT2	ORF-T	ORF-T	0.161574756	-0.142821974	-0.316248589	-0.166329314	-0.259248416
295345	298145	5	+	ARG5%2C6	ORF-T	ORF-T	-0.341380936	-0.365466736	-1.102616433	-0.17241149	-0.777336298
298881	301769	5	+	RNR1	ORF-T	ORF-T	-0.002196218	-0.001325773	-1.439201638	-0.135796042	0.249533996
302729	303337	5	+	VTC1	ORF-T	ORF-T	-0.670344599	0.953031796	1.066147293	1.286231094	1.516301177
303529	303961	5	+	ALD5	ORF-T	ORF-T	-0.059988326	-0.459694999	0.365422024	0.167531434	-0.663074415
306273	306793	5	+	RPS24A	ORF-T	ORF-T	0.695069536	-1.023057319	-1.014020828	-1.242971993	-0.06218932
308065	308385	5	+	unknown	no_ovlp	SUTs	0.354549705	-0.493309214	0.353004279	-0.10271308	0.243701615

313585	315289	5	+	unknown	no_ovlp	CUTs	1.40411018	0.466857444	0.317425006	0.395879267	-0.411497962
316761	317385	5	+	CUT111	CUTs	CUTs	2.601237865	0.453806153	-0.727719039	-0.367304441	0.55635162
318849	319657	5	+	YER079W	ORF-T	ORF-T	-0.46558868	-0.481229487	1.236174878	0.784673591	0.735762928
319937	321905	5	+	FMP29	ORF-T	ORF-T	-0.057694503	-0.259133794	0.141251629	-0.072650381	-0.156492992
322217	324177	5	+	SRG1, SER3	ORF-T	ORF-T	-0.400890267	-0.487719252	0.023312943	-0.358337621	-0.144450358
326193	327593	5	+	CUT112	CUTs	CUTs	0.595651408	0.512690571	-0.073986686	-0.258678222	-0.158958311
328401	330353	5	+	ILV1	ORF-T	ORF-T	-0.069636918	-0.014133314	-0.58680978	0.037169073	-0.302328017
330537	332441	5	+	YER087W	ORF-T	ORF-T	0.044811985	-0.10527743	0.206026026	-0.050080533	-0.038606423
335737	336937	5	+	unknown	no_ovlp	CUTs	1.170757522	0.277938529	-0.294918419	-0.175719301	1.309791203
337881	339633	5	+	TRP2	ORF-T	ORF-T	-0.014148069	-0.039768979	-0.273117239	-0.244450241	-0.179460255
342857	343281	5	+	IES5	ORF-T	ORF-T	0.176445347	0.02726097	0.368164019	-0.725981156	0.515760421
347833	348553	5	+	CUT114	CUTs	CUTs	2.429297532	0.395347466	-0.742051256	-0.247198039	0.135414367
349929	351409	5	+	RAD51	ORF-T	ORF-T	-0.032781938	-0.008957555	0.548053945	-0.07518627	1.10663772
351409	354921	5	+	SHC1	ORF-T	ORF-T	0.270063306	0.87626721	1.673990503	1.272644405	3.760512203
353225	354921	5	+	unknown	no_ovlp2	SUTs	0.574736307	0.391097465	0.897624518	0.269973469	-0.168615064
354921	355225	5	+	unknown	no_ovlp2	CUTs	1.329486414	-0.363563283	-1.094172168	-1.209450074	0.019511284
355225	357945	5	+	YER097W, UI	ORF-T	ORF-T	-0.025671274	-0.383588211	0.43485276	0.614036604	-0.332484999
359289	360401	5	+	UBC6	ORF-T	ORF-T	-0.104568616	-0.58767439	-0.256510946	-0.11576933	0.032495516
361649	362705	5	+	unknown	no_ovlp	SUTs	0.394183862	0.363509263	0.360820075	0.107582854	1.609665663
362705	363097	5	+	RPS8B	ORF-T	ORF-T	0.702974469	-0.807439261	-0.562630597	-0.996482444	-0.047339441
364593	366649	5	+	SSA4	ORF-T	ORF-T	-0.088008319	-0.260845216	1.529955393	0.571043985	-0.40283454
366785	367793	5	+	RTT105	ORF-T	ORF-T	-0.269112963	-0.266110183	0.475774986	-0.232820009	0.960286457
376153	377961	5	+	unknown	no_ovlp	SUTs	-0.183554963	0.514331389	0.315048666	0.560630504	1.198936108
377961	378825	5	+	SUT096	SUTs	SUTs	-0.140984455	-0.668392448	-0.915536023	-0.69661656	-0.059990682
387161	387905	5	+	LSM4	ORF-T	ORF-T	0.046212333	-0.704195558	-0.332605224	0.047518389	0.087646256
395289	395769	5	+	CUT115	CUTs	CUTs	2.441220223	0.253016709	-1.521738041	0.086308421	0.635352753
396809	397289	5	+	RPL23B	ORF-T	ORF-T	0.65599266	-0.872753476	-1.304139445	-1.232353141	-0.549251936
399505	401097	5	+	unknown	no_ovlp	CUTs	1.039387806	0.174581082	-0.361339376	-0.188131757	0.613874263
401097	402009	5	+	SCS2	ORF-T	ORF-T	-0.304209989	-0.482088833	-0.349135344	-0.012127196	0.111547592
402097	402881	5	+	YER121W	ORF-T	ORF-T	-0.563857681	0.217293481	2.394001324	1.868420574	0.396841113
404633	406809	5	+	YCK3	ORF-T	ORF-T	-0.136822252	-0.5265211	-0.030374916	0.091170425	0.276434415
409673	413009	5	+	RSP5	ORF-T	ORF-T	-0.191427127	-0.264874736	-0.180518284	0.00806635	-0.032267689
414465	415657	5	+	LCP5	ORF-T	ORF-T	0.289961025	-0.178132385	-0.746456933	-0.266844801	0.859811727
415833	416553	5	+	YER128W	ORF-T	ORF-T	-0.263857612	0.344502966	0.330770326	-0.167520043	1.103378101
416785	420881	5	+	SAK1	ORF-T	ORF-T	0.041738818	-0.61467278	-0.341197031	-0.004789494	-0.071317063
423585	424009	5	+	RPS26B	ORF-T	ORF-T	1.016509614	-1.347377318	-1.074144624	-0.903617094	-0.179040028
424705	425209	5	+	SNR4	other	other	-0.235234101	-0.077073014	-0.054578399	-0.088798236	0.382446614
424849	425209	5	+	unknown	no_ovlp2	CUTs	3.545251226	-1.248125563	-0.216637198	-0.091977319	-0.179176714
432433	432665	5	+	GLC7	ORF-T	ORF-T	0.058947286	-0.631782599	-0.112065797	0.178896717	-0.581585136
437993	438425	5	+	unknown	no_ovlp	CUTs	3.072257075	0.080411726	0.05026105	-0.232425615	-0.563995389
439497	441289	5	+	GDI1	ORF-T	ORF-T	-0.024741213	-0.363043824	-0.245360631	-0.275817094	-0.488910791
441993	442497	5	+	SCR1	other	other	-0.338835076	0.048639061	-0.052812524	-0.166658535	-0.062754202
449625	449913	5	+	unknown	no_ovlp	CUTs	1.440067391	-0.379108945	-1.338359625	-0.66829922	-0.898433607
451529	453433	5	+	YER140W	ORF-T	ORF-T	0.039482441	-0.330103848	-0.958271257	-0.23947336	-0.376451635
453433	455153	5	+	COX15	ORF-T	ORF-T	-0.478111718	-0.60397711	-0.009520068	0.159859272	-0.282903307
456289	457441	5	+	DDI1	ORF-T	ORF-T	0.225641759	-0.250172191	0.331730424	0.13142747	-0.070253435
460505	461601	5	+	unknown	no_ovlp	CUTs	1.741343095	-0.388300228	-0.4097079	-0.274003028	0.799998092
462553	462905	5	+	LSM5	ORF-T	ORF-T	-0.38314989	-0.48538546	-0.39702307	-0.269721032	-0.121445121
465137	466209	5	+	SPT15	ORF-T	ORF-T	-0.054273099	-0.324986153	-0.769332791	-0.114623937	-0.294226557
467721	468345	5	+	unknown	no_ovlp	CUTs	2.011627285	0.372237045	-0.244097368	-0.844827702	-1.207976255
468345	468993	5	+	SPI1	ORF-T	ORF-T	0.07451076	-0.848544624	0.428674041	0.80482921	-0.946265433
474065	475009	5	+	unknown	no_ovlp	SUTs	-0.679002652	0.103915199	0.115733517	0.173189915	0.794564714
475009	476289	5	+	OXA1	ORF-T	ORF-T	-0.362912043	-0.324530569	-0.302578595	-0.164367301	-0.226393395
483145	484105	5	+	CUT118	CUTs	CUTs	2.189774601	0.072784016	-0.890417759	0.025273391	0.355843101
484537	487249	5	+	COG3	ORF-T	ORF-T	0.086243623	-0.406114918	-0.407708818	-0.246908043	-0.131614386
488689	491545	5	+	SUT097	SUTs	SUTs	0.132507774	0.790402123	1.086732672	-0.0236272	0.545489701
491745	499089	5	+	unknown	no_ovlp	CUTs	1.450014526	0.213674306	-0.891100543	-0.035916874	-0.687669605
500617	501297	5	+	CUT119	CUTs	CUTs	3.135086596	0.161674218	-2.298696523	-0.447536228	0.029442742
504017	504809	5	+	SUT098	SUTs	SUTs	0.635683692	-0.491235044	-0.309754799	-0.115260549	-0.187323397
505097	508057	5	+	CHD1	ORF-T	ORF-T	-0.116832633	-0.699043064	-0.773518798	0.254371565	-0.245382089
510257	512289	5	+	PAB1	ORF-T	ORF-T	-0.18325857	-0.235240009	-0.535727281	-0.05634836	-0.537222011
512449	516169	5	+	DNF1	ORF-T	ORF-T	-0.7176727	-0.428165295	0.014543312	0.088228732	0.318256682
517649	517929	5	+	unknown	no_ovlp	SUTs	-0.604422332	0.015998878	0.740490613	0.786930764	2.509699837
517929	520905	5	+	BCK2	ORF-T	ORF-T	-0.138916056	-0.4333611	-0.331476789	-0.093507172	0.113781856
523105	524057	5	+	RPH1	ORF-T	ORF-T	0.293827779	-0.336625762	-0.013918753	-0.469454226	-0.001168897
525873	526969	5	+	ADK2	ORF-T	ORF-T	-0.896246614	-0.935061109	0.54201028	0.098026671	0.194673817
526969	529513	5	+	RAD3	ORF-T	ORF-T	0.220057904	-0.447692631	-0.718448577	-0.247215582	-0.201669816
536281	538417	5	+	RAD24	ORF-T	ORF-T	0.102260031	-0.18218291	-0.033294548	-0.592870807	0.236972914
539585	541657	5	+	unknown	no_ovlp	SUTs	-0.89765246	-0.531454905	0.105260151	0.560720346	1.203939909
541657	545145	5	+	ECM32	ORF-T	ORF-T	-0.097522655	-0.257265958	-0.372418379	-0.039388759	-0.079700324
545561	546577	5	+	BMH1	ORF-T	ORF-T	-0.354769369	-0.337831181	-0.041542156	0.07441635	-0.019881935
546793	548217	5	+	PDA1	ORF-T	ORF-T	-0.175660997	-0.404280611	-0.134716456	0.151729939	0.073760105
552497	553345	5	+	FMP10	ORF-T	ORF-T	-0.048465405	-0.419543702	0.515813626	0.078985437	-0.630028795
554145	555145	5	+	SUT099	SUTs	SUTs	0.74198116	-0.45725996	-0.031485304	-0.489234865	-1.061253023
556505	559305	5	+	unknown	no_ovlp2	SUTs	0.512593555	0.498385376	0.000137084	-0.249484015	-0.112241001
559305	561321	5	+	YER185W	ORF-T	ORF-T	-0.075785951	-0.253824165	0.446795597	0.622540566	1.297979915
560401	561321	5	+	unknown	no_ovlp2	SUTs	0.375991727	0.10060626	-0.10582087	0.000330492	0.078756205
562985	563361	5	+	SUT100	SUTs	SUTs	1.853182651	0.262975152	1.174988817	0.797472056	-0.86022576
565601	566937	5	+	YER187W	ORF-T	ORF-T	1.621151286	0.910441085	-0.46742623	-0.389101409	-2.478672334
566697	566937	5	+	unknown	no_ovlp2	CUTs	1.591665717	2.342216735	0.965976061	-0.263393764	-1.811798177
567945	568921	5	+	YER188W, YE	other	other	0.711729314	-0.336802535	-0.225530633	0.32291721	-2.739218447
14245	14900	6	-	unknown	no_ovlp2	SUTs	0.499770306	-0.761239616	-0.416903567	-0.476490312	-1.422772819

14900	16453	6	-	AAD6	ORF-T	ORF-T	0.467201932	-0.492425775	-0.14995139	-0.380403037	-0.931143518
27117	27885	6	-	YFL054C	ORF-T	ORF-T	-0.084409861	-0.643411417	0.894999066	1.248027899	-1.329392017
32997	36581	6	-	ALR2	ORF-T	ORF-T	0.719617489	-0.180484449	-0.172471723	0.059455138	-0.284472744
38733	40205	6	-	EMP47	ORF-T	ORF-T	-0.102907955	-0.402765453	-0.501820532	-0.068271272	-0.38166841
43477	44477	6	-	SEC53	ORF-T	ORF-T	-0.678881432	-0.601374122	-0.381299299	-0.14396706	0.208273011
44477	45573	6	-	OTU1	ORF-T	ORF-T	0.164351145	0.109210154	-0.104368164	-0.155379882	-0.612368377
45669	47765	6	-	YFL042C	ORF-T	ORF-T	-0.206204686	-0.023427494	0.40359437	0.249762984	-0.151437728
51773	52981	6	-	unknown	no_ovlp	SUTs	-1.256619606	-0.196685316	1.460281444	-0.078436031	-0.553449623
54381	54693	6	-	ACT1	ORF-T	ORF-T	0.581631533	-1.143734808	-0.705239647	-1.325918455	-0.751353206
55261	55997	6	-	YPT1	ORF-T	ORF-T	-0.204612213	-0.252925466	0.129129577	0.177578565	-0.185196073
62909	63853	6	-	MOB2	ORF-T	ORF-T	-0.115813637	-0.143497222	-0.022468462	-0.208494068	0.342102541
64165	64981	6	-	RPL22B	ORF-T	ORF-T	-1.069123836	-1.232297054	-0.310267852	-0.712741541	0.197376562
68877	74669	6	-	RIM15	ORF-T	ORF-T	-0.062701182	-0.562190859	-0.182274552	-0.026794268	0.057496867
78061	79181	6	-	CAK1	ORF-T	ORF-T	0.208029499	-0.390561263	-0.161044099	-0.423600536	-0.295771535
79277	80221	6	-	CAF16	ORF-T	ORF-T	0.604344064	-0.639106973	-0.878372756	-0.479945191	-0.654173282
80357	82013	6	-	GYP8	ORF-T	ORF-T	0.455269988	-0.335010149	-0.542634774	-0.161967381	-0.750114577
85229	87309	6	-	BST1	ORF-T	ORF-T	-0.044792886	-0.344343223	-0.545844943	-0.350250848	0.07442924
89037	90613	6	-	EPL1	ORF-T	ORF-T	-1.408343056	-1.302932316	0.375704515	-0.693093934	-1.460438001
93429	95029	6	-	FRS2	ORF-T	ORF-T	-0.220666821	-0.452406115	-0.959749978	-0.112987381	-0.335525208
95669	95957	6	-	YFL021C-A	other	other	0.343099036	-0.133913377	0.710912951	0.792966449	0.661067085
95957	97645	6	-	unknown	no_ovlp	SUTs	-0.230502503	0.29528491	0.137057124	0.310711116	0.100464818
101541	103221	6	-	LPD1	ORF-T	ORF-T	-0.294090222	-0.243859044	0.241386252	0.205868009	-0.238270185
103877	104485	6	-	GNA1	ORF-T	ORF-T	-0.792279724	-0.716227575	0.274145849	-0.064223176	1.012207785
104605	106293	6	-	MDJ1	ORF-T	ORF-T	0.212406173	-0.389304345	-0.153354869	-0.134690411	-0.826608345
107797	109949	6	-	IES1	ORF-T	ORF-T	-9.92E-05	-0.385881888	-0.21740228	0.16651622	-0.331359826
111117	111821	6	-	unknown	no_ovlp	SUTs	0.488437537	-0.114793251	0.727506908	-0.184835499	-1.552991274
114949	115765	6	-	WWM1	ORF-T	ORF-T	0.323994976	-0.069304885	-0.267645374	-0.174286785	-0.828260028
118637	119061	6	-	CUT575	CUTs	CUTs	2.4290239	0.689402365	-0.022161031	-0.550764152	-1.29668731
131117	131613	6	-	SUT527	SUTs	SUTs	1.567905439	-0.095923882	-0.630870432	-1.056449733	-0.412836643
131613	134549	6	-	unknown	no_ovlp2	SUTs	-0.03735258	0.327716554	0.025909231	0.00083866	0.087322602
134549	144981	6	-	MSH4	ORF-T	ORF-T	-0.226668345	0.157522708	0.658107169	-0.089268048	0.504500268
135837	144981	6	-	unknown	no_ovlp2	SUTs	-0.159473165	0.257491985	0.094325853	0.002655215	0.161088067
144981	146957	6	-	SPB4	ORF-T	ORF-T	0.436135101	-0.264030261	-0.492457556	-0.07799227	-0.205860882
148693	149781	6	-	CUT576	CUTs	CUTs	0.77134061	0.235530507	-0.243432989	-0.094727767	-0.063129434
152573	153173	6	-	YPI1	ORF-T	ORF-T	-0.538429532	-0.694878015	0.339535794	0.015034495	0.10309043
154341	155901	6	-	SAD1	ORF-T	ORF-T	-0.06855721	-0.802903912	-0.560571948	-0.274033477	-0.18441569
158373	160357	6	-	SUT528	SUTs	SUTs	1.511326033	-0.350121876	-0.464255489	-0.250425362	-1.17114511
166589	167285	6	-	YFR011C	ORF-T	ORF-T	0.090703243	-0.636595095	0.184956663	-0.209187959	0.172625964
172317	173925	6	-	CMK1	ORF-T	ORF-T	-0.54348337	-0.591927245	0.76598957	1.004119823	0.373910861
174149	176637	6	-	GSY1	ORF-T	ORF-T	-1.979880938	-1.800736495	0.98101977	1.655204147	-1.058386526
176981	180781	6	-	YFR016C	ORF-T	ORF-T	-0.32561674	-0.438409545	0.142165436	0.146923954	-0.022062225
180781	181037	6	-	unknown	no_ovlp	SUTs	-0.131907304	-2.205185659	-1.458825932	-1.483458845	-1.919408745
182197	182925	6	-	YFR017C	ORF-T	ORF-T	-0.945301686	-1.299987546	1.034187917	0.993524386	0.494760323
182925	184277	6	-	YFR018C	ORF-T	ORF-T	-0.153832739	0.454873958	0.367670641	-0.376042072	0.427798873
191605	192461	6	-	unknown	no_ovlp	SUTs	0.706365181	0.41746128	-0.263030538	-0.253066094	-0.41592035
195501	196349	6	-	CUT578	CUTs	CUTs	3.190479739	0.680687863	-1.572913711	-0.651133164	-0.721969998
201757	203277	6	-	LSB3	ORF-T	ORF-T	-0.315638455	-0.130247954	0.222838311	0.133205079	-0.059367438
203637	204789	6	-	HIS2	ORF-T	ORF-T	-0.142863379	-0.708378916	-0.31763151	-0.575260676	0.088038479
205669	206293	6	-	YFR026C	ORF-T	ORF-T	0.218592017	-0.52863362	-0.390040586	-1.334229788	-0.05213827
208413	210117	6	-	CDC14	ORF-T	ORF-T	-0.456753238	-0.127960751	-0.152155419	-0.039220401	0.503341014
210117	213381	6	-	SUT530	SUTs	SUTs	0.267833401	0.505636147	-0.025215287	-0.04401543	1.202342083
216557	220069	6	-	SMC2	ORF-T	ORF-T	0.058806513	-0.022531484	-0.050222209	-0.309553286	0.595540053
220341	221253	6	-	RPL2A	ORF-T	ORF-T	-0.35249372	0.127226994	-0.187673884	0.045470378	-0.036778981
223421	223773	6	-	RPL29, YFR0	ORF-T	ORF-T	0.493445215	-0.175929887	-0.531701535	-0.837019343	0.341273611
224181	224869	6	-	QCR6	ORF-T	ORF-T	-0.272149111	-0.58326297	0.398599063	0.082601609	0.054237503
224869	225957	6	-	PHO4	ORF-T	ORF-T	0.20392425	0.300016436	-0.268704361	-0.238124664	-0.015654583
225957	227397	6	-	YFR035C	ORF-T	ORF-T	0.934835178	0.260277514	-0.531564236	0.009608108	0.041831284
226453	227397	6	-	unknown	no_ovlp2	SUTs	0.055211479	0.227452598	-0.090472148	0.487458121	1.18230761
227397	229197	6	-	RSC8	ORF-T	ORF-T	-0.237220346	-0.293532364	-0.108676054	0.17491725	0.26029005
231941	233653	6	-	YFR039C	ORF-T	ORF-T	-0.515336535	-0.419955855	-0.025708475	-0.224036144	0.373516668
237229	238269	6	-	ERJ5	ORF-T	ORF-T	-0.266009905	-0.306099553	-0.131255309	-0.359231406	0.118145452
239093	239925	6	-	IRC6	ORF-T	ORF-T	0.379383148	-0.9602912	-0.351259478	-0.153220955	-0.440305947
239925	241445	6	-	DUG1	ORF-T	ORF-T	-0.211151132	-0.319198993	-0.071467595	-0.096785698	-0.054372203
243021	244197	6	-	CNN1	ORF-T	ORF-T	0.047740153	-0.265674743	-0.373318713	-0.224506112	-0.205629928
244197	245181	6	-	BNA6	ORF-T	ORF-T	0.997948081	0.824306119	-0.08449558	-0.312742398	-2.08463866
245181	245965	6	-	CUT579	CUTs	CUTs	2.306941402	0.002754297	-1.042152791	-0.923998649	-2.117785196
248925	249901	6	-	PRE4	ORF-T	ORF-T	0.026760616	-0.103750545	0.145652236	-0.026236365	0.026766929
250085	251797	6	-	RET2	ORF-T	ORF-T	-0.444903551	0.103608984	-0.354010093	-0.031942989	-0.032188912
251965	252277	6	-	CUT580	CUTs	CUTs	3.129917351	0.244149567	-0.208715456	-0.326689485	0.1725496
253421	255133	6	-	YFR052C-A, I	ORF-T	ORF-T	-0.436187983	-0.604965346	0.986104463	0.962481516	-0.258493685
255173	259205	6	-	unknown	no_ovlp	SUTs	0.781994498	0.528180802	0.145535144	0.259693307	-1.07304936
259205	262261	6	-	unknown	no_ovlp	SUTs	-0.107822971	0.208029083	0.158804177	1.050116246	-0.165821596
262461	263349	6	-	SUT531	SUTs	SUTs	0.145763867	-1.417648952	-0.071608566	-0.082656909	-2.383369945
263349	263781	6	-	CUT581	CUTs	CUTs	1.538577255	-0.459158921	-0.944904668	-0.34351774	-1.048269823
16681	16977	6	+	AGP3	ORF-T	ORF-T	2.615626976	-0.293047905	0.803539171	0.383071109	0.012487622
23105	25225	6	+	CUT120	CUTs	CUTs	0.717055236	0.61539396	2.041062476	2.665685927	-0.842687432
28225	30825	6	+	YFL052W	ORF-T	ORF-T	0.564454935	-0.290799974	0.803213271	1.356355582	-1.610046524
36257	36801	6	+	unknown	no_ovlp	CUTs	1.856801184	0.253295718	-1.340830082	0.169874019	-1.167183664
36801	38825	6	+	SWP82	ORF-T	ORF-T	-0.058225136	-0.593427781	-0.186948718	-0.081073479	-0.085992047
40425	42609	6	+	RGD2	ORF-T	ORF-T	-0.027909707	-0.519742007	-0.65993702	-0.421247799	-0.95923569
42809	43537	6	+	FMP32	ORF-T	ORF-T	0.206877998	0.034571401	0.085239093	-0.367073017	0.207461987
44729	45929	6	+	CUT121	CUTs	CUTs	1.996686656	0.147248376	-0.534836856	-0.259210307	0.266677417

47889	48953	6	+	YFL041W-A	ORF-T	ORF-T	-0.047421195	-0.381014388	0.4236523	0.276039475	-0.312542003
49137	51193	6	+	FET5	ORF-T	ORF-T	-0.188799309	-0.335749337	-0.152393475	0.328434955	0.01575408
54633	55529	6	+	SUT101	SUTs	SUTs	0.992170591	0.068556793	0.258850315	-0.18864881	-0.584935953
56273	57937	6	+	TUB2	ORF-T	ORF-T	-0.327921758	-0.306183083	-0.118541817	-0.015826828	0.368775633
58577	59609	6	+	RPO41	ORF-T	ORF-T	-0.3930851	-0.98123617	0.015656596	0.157997544	-0.13432831
65321	68809	6	+	YFL034W	ORF-T	ORF-T	-0.245301681	-0.181161992	-0.126999909	-0.10381304	0.269224649
75121	76561	6	+	YFL032W, HA	ORF-T	ORF-T	-0.506689595	-0.594026085	-0.293940866	-0.08535743	-0.508152572
76561	78073	6	+	AGX1	ORF-T	ORF-T	-1.645477669	-0.691033973	2.401718208	0.716711995	1.537983255
80425	80897	6	+	unknown	no_ovlp	CUTs	1.749105477	0.49918227	-0.827522693	-0.253708147	0.047979201
82585	84065	6	+	STE2	ORF-T	ORF-T	0.168931687	-0.140149414	-0.72050205	-0.100592046	-0.598430397
89097	89625	6	+	unknown	no_ovlp	SUTs	-0.141392199	0.520327941	-0.106513741	-0.149099113	0.207964178
89625	90657	6	+	SUT103	SUTs	SUTs	3.650862785	1.919627155	-1.369450112	0.064373275	0.22651161
90985	93449	6	+	BUD27	ORF-T	ORF-T	0.111154529	0.085121291	-0.48319048	-0.013237298	0.760729214
95593	96017	6	+	GAT1	ORF-T	ORF-T	0.369787216	-0.151739109	0.002743086	-0.464292921	-1.491336217
96017	97593	6	+	GAT1	ORF-T	ORF-T	-0.42335075	-0.379006834	-0.429113389	-0.27803206	-0.149263259
103649	103977	6	+	SMX2	ORF-T	ORF-T	-0.150879354	-0.981931128	0.05852506	-0.425976679	-0.093631718
106937	107201	6	+	CUT122	CUTs	CUTs	0.289434618	0.374337443	1.080467667	1.091569363	1.72686532
107201	107785	6	+	HSP12	ORF-T	ORF-T	-0.831835787	-1.042388106	1.917407417	0.986926125	0.955414236
110177	110609	6	+	SUT105	SUTs	SUTs	1.853693722	-0.364084497	-0.317336874	0.406983268	-1.217664195
114665	115377	6	+	unknown	no_ovlp	CUTs	1.02283817	0.730412404	0.207843638	-0.415886991	-1.050630342
115953	118561	6	+	CDC4	ORF-T	ORF-T	-0.291181846	-0.503149649	-0.339196334	0.079330189	0.127923748
119393	121025	6	+	SMC1	ORF-T	ORF-T	-0.252340138	-0.346098035	-0.137229902	-0.051579313	0.686868798
123345	125945	6	+	BLM10	ORF-T	ORF-T	-0.040639219	-0.225337837	0.093761205	0.237896866	0.7447362
130169	131177	6	+	SEC4	ORF-T	ORF-T	-0.492455811	-0.844968921	-0.184098419	-0.127633138	-0.313022034
131777	134473	6	+	VTC2	ORF-T	ORF-T	0.206027671	0.263076158	-0.704438552	0.474499987	-0.125870534
137665	144257	6	+	unknown	no_ovlp	SUTs	-0.80857189	-0.763141483	0.431990094	0.368762143	-0.449484483
147129	148481	6	+	DEG1	ORF-T	ORF-T	0.185293961	-0.507810781	-0.500289574	-0.23915087	-0.231372728
149089	149801	6	+	LOC1	ORF-T	ORF-T	0.722991804	-0.186181364	-0.572999616	-0.323636189	0.090418106
150009	152625	6	+	NIC96	ORF-T	ORF-T	0.098819102	-0.514963903	-0.003865386	0.181710855	-0.11160049
153353	154393	6	+	RPN11	ORF-T	ORF-T	0.108755208	-0.706483792	-0.244987369	0.002797906	-0.35341072
156185	157849	6	+	YFR006W	ORF-T	ORF-T	-0.069100127	-0.55197595	0.021893446	-0.089772467	-0.121958539
159289	161633	6	+	YFR007W	ORF-T	ORF-T	-1.001906125	-0.323496928	0.623832865	0.093193453	-0.1843127
160417	161633	6	+	unknown	no_ovlp2	SUTs	-0.200938966	-0.796291284	0.063492766	-0.407620373	-0.511790557
162417	164833	6	+	GCN20, YFR(	ORF-T	ORF-T	0.323801183	-0.679865862	-0.362562705	-0.046558174	-0.510618044
164993	166649	6	+	UBP6	ORF-T	ORF-T	0.039005509	-0.421625014	-0.001237774	-0.05035193	-0.316505485
167153	167977	6	+	unknown	no_ovlp2	SUTs	0.255146694	0.414578366	-0.225132559	-0.253086564	-0.168432855
167977	169865	6	+	YFR012W, YF	ORF-T	ORF-T	-0.324979982	0.499322795	0.320873431	-0.150470937	-0.171609422
169297	169865	6	+	unknown	no_ovlp2	SUTs	-0.399836428	-0.133653888	0.213547079	0.072944845	0.281099545
169865	172425	6	+	IOC3	ORF-T	ORF-T	-0.005205069	0.018197839	-0.180029901	-0.02707825	-0.53227948
175689	177473	6	+	unknown	no_ovlp	SUTs	0.618854697	0.856469906	0.08147804	-0.253059719	-0.436777219
182345	184489	6	+	unknown	no_ovlp	SUTs	-0.260722027	0.273998535	0.149312077	-0.040510334	0.744549384
184489	186825	6	+	FAB1	ORF-T	ORF-T	0.201011343	0.172900173	0.387644244	-0.296023397	0.576108753
191769	192289	6	+	YFR020W	other	other	-0.11348163	-1.14767709	0.285856487	-0.276284372	-0.597706426
194713	196409	6	+	ATG18	ORF-T	ORF-T	-0.592089133	-0.200177641	0.125960246	-0.033606845	0.455828129
196569	196865	6	+	ROG3	ORF-T	ORF-T	0.466148956	-0.816062481	-0.138544713	0.137399505	-0.090806296
199161	200281	6	+	unknown	no_ovlp2	SUTs	0.015481387	0.42172255	0.128554644	-0.227974158	-0.139117547
200281	202121	6	+	PES4	ORF-T	ORF-T	-0.170904094	1.109961442	1.822944412	-0.193450248	-0.215080021
201473	202121	6	+	unknown	no_ovlp2	SUTs	-0.211103022	0.391652887	0.514094658	-0.149971195	0.124586129
202121	207385	6	+	unknown	no_ovlp	SUTs	0.061643077	0.320287009	0.231599182	0.155204854	0.139804114
207385	208329	6	+	ECO1	ORF-T	ORF-T	-0.444804272	-0.412233661	0.514309374	-0.090253047	0.902807999
210873	213009	6	+	PTR3	ORF-T	ORF-T	-0.287980475	-0.361987261	0.103077772	-0.246630346	0.446197196
213265	216489	6	+	MET10	ORF-T	ORF-T	-0.231368034	0.324356806	0.379042632	0.045578097	0.813750235
220297	222185	6	+	unknown	no_ovlp	SUTs	0.006976871	0.409470859	0.013066477	-0.155720603	0.05217691
223281	223561	6	+	unknown	no_ovlp	CUTs	1.79231684	0.972473224	-0.664229515	-0.666100729	-0.659062504
225569	226185	6	+	unknown	no_ovlp2	CUTs	1.043921839	-0.284550164	0.004263764	-0.239920068	0.635155031
226185	226633	6	+	YFR034W-A	other	other	-0.26503184	-0.670933604	-0.082500104	-0.492295215	0.946313243
226937	227385	6	+	CDC26	ORF-T	ORF-T	0.250529679	-0.787975552	0.185769653	-0.115839924	0.528821601
229401	232017	6	+	IRC5	ORF-T	ORF-T	-0.412413072	-0.306077976	0.01172946	0.033576598	0.79737499
233945	236105	6	+	SAP155	ORF-T	ORF-T	-0.022527899	-0.571639679	-0.175332484	0.192856402	-0.574500693
238409	239153	6	+	KEG1	ORF-T	ORF-T	-0.036992591	-0.485917347	0.218109588	-0.853361774	0.118652524
241977	243033	6	+	YFR045W	ORF-T	ORF-T	-0.312410314	-0.36857453	0.366530546	-0.054811731	-0.223226317
246121	248185	6	+	RMD8	ORF-T	ORF-T	-0.056958244	-0.793184896	-0.624723685	-0.286866904	-0.680932643
248353	248993	6	+	YMR31	ORF-T	ORF-T	-0.565215316	-0.437096917	0.965629914	0.175760743	0.774854535
251641	252465	6	+	unknown	no_ovlp	SUTs	0.360793968	0.63548243	-0.263365009	0.078435975	0.122063994
252465	253297	6	+	RPN12	ORF-T	ORF-T	-0.10441537	-0.703574798	0.316219437	-0.151786524	-0.125950562
255697	256913	6	+	unknown	no_ovlp	SUTs	0.008022927	-0.248361997	1.161758769	0.768538573	-0.638749725
263441	264177	6	+	unknown	no_ovlp	CUTs	1.136803686	-0.796183871	-0.698541048	-0.502433583	-1.488532046
264177	265689	6	+	IRC7	ORF-T	ORF-T	0.167910739	-1.082413749	-1.297796139	-0.705402093	-2.074540472
266449	268241	6	+	unknown	no_ovlp	CUTs	1.125568284	0.429812368	0.002534239	-0.69540545	-1.518578159
5005	5709	7	-	unknown	no_ovlp	SUTs	-1.090727704	-0.902184562	-0.046488424	-0.029725953	-0.14384293
5709	11285	7	-	unknown	no_ovlp	SUTs	0.090729676	0.341878696	-0.055864842	-0.06316188	0.011022524
12365	14301	7	-	MNT2	ORF-T	ORF-T	-0.036705776	-1.05559421	-1.060764069	-0.824557359	-0.112544784
14301	14517	7	-	unknown	no_ovlp	CUTs	1.886889734	0.035244757	-0.914448669	-0.528769738	-1.508659753
18677	20109	7	-	unknown	no_ovlp	SUTs	0.521388461	-1.469652447	-1.841266219	-0.091309529	-0.650596127
22269	23301	7	-	CUT583	CUTs	CUTs	1.870782784	0.03677853	0.281188406	-0.013208979	-0.623386374
25653	27541	7	-	RTG2	ORF-T	ORF-T	-0.361085215	-0.522072373	-0.024802569	0.110095651	-0.065922519
27541	27749	7	-	unknown	no_ovlp2	SUTs	0.034312261	0.04347592	0.21659802	-0.352689838	-0.343831477
27749	30213	7	-	HFM1	ORF-T	ORF-T	-1.080339886	-0.219837233	0.991257543	0.726062981	-0.72087042
34005	35301	7	-	unknown	no_ovlp	CUTs	1.566845607	-1.099363196	-0.526479922	-0.444904154	-1.396595176
37453	38813	7	-	RAI1	ORF-T	ORF-T	0.55853523	-0.509779005	-0.623347726	-0.319565253	-0.126835621
44533	45221	7	-	YGL242C	ORF-T	ORF-T	-0.060593382	-0.317506792	-0.294586613	-0.820650643	0.01342479
48493	49309	7	-	unknown	no_ovlp	SUTs	0.724131606	-1.398346694	-1.845511315	-0.572152976	0.334848545

52525	53597	7 -	HAP2	ORF-T	ORF-T	-0.405897168	-0.428950515	0.343138413	0.046262652	0.284536594
53765	55702	7 -	MTO1	ORF-T	ORF-T	-0.856351227	-0.546400804	-0.525388232	-0.091615519	-0.533634945
55702	56182	7 -	CUT585	CUTs	CUTs	2.559199742	-0.387389058	-0.909748837	-0.239846183	-0.138354939
62998	63638	7 -	YGL231C	ORF-T	ORF-T	0.378736397	-0.42985554	-0.23140705	-0.11964929	-0.156562439
63638	64414	7 -	YGL230C	ORF-T	ORF-T	-0.889705796	-0.505471037	0.219523251	0.505020886	-0.31842475
64414	67182	7 -	SAP4	ORF-T	ORF-T	-0.808196965	-0.692829777	0.135021749	0.231629143	-0.015067263
72606	72982	7 -	OST5	ORF-T	ORF-T	-0.062163594	-0.23808758	0.023814294	-0.293303882	0.522615849
73974	74262	7 -	tV(AAC)G3	other	other	3.135570479	-0.416079496	-0.278752625	-0.205848411	-0.397131184
74262	74742	7 -	unknown	no_ovlp	CUTs	1.181345555	0.402263601	-0.078226903	-0.423672837	-0.25823786
77918	79094	7 -	unknown	no_ovlp2	SUTs	-0.434882359	-0.874054379	-0.155324215	0.211411422	0.115891799
79094	80366	7 -	COG1	ORF-T	ORF-T	0.233856803	-0.508701529	-0.127552903	-0.539416231	-0.411944503
80838	81230	7 -	EDC1	ORF-T	ORF-T	-0.075845504	-0.62208303	0.342926211	0.268684336	-0.047471425
81334	82310	7 -	NIF3	ORF-T	ORF-T	0.416907765	0.035443868	-0.196723522	-0.290894405	-0.04100912
82798	84278	7 -	MDM34	ORF-T	ORF-T	0.048158358	-0.156776084	0.06505214	-0.122903358	-0.140637809
89942	91270	7 -	SKI8	ORF-T	ORF-T	0.081661383	-0.443208859	-0.585758122	0.072455942	0.067583478
94295	94743	7 -	SUT535	SUTs	SUTs	3.263239093	-0.354628056	-0.008513044	-0.236093775	-0.330854806
94743	95295	7 -	SUT536	SUTs	SUTs	1.472161812	-0.114423065	0.825614307	-0.186958975	-0.450364879
97159	98479	7 -	unknown	no_ovlp	CUTs	1.499906173	0.249051005	-0.928065143	-0.247235251	-1.065781859
102311	107599	7 -	CHC1	ORF-T	ORF-T	-0.494296419	-0.348689765	0.085959284	0.196122437	-0.112760173
108631	110295	7 -	SUT537	SUTs	SUTs	0.911679085	-0.461113097	-0.662915693	-0.766894572	-3.176775104
111223	111687	7 -	unknown	no_ovlp2	SUTs	0.908970448	-0.329190367	0.231887083	-0.716808878	-3.494054356
111687	112143	7 -	YGL204C	other	other	0.225292777	-0.556496772	0.372029596	0.072801579	-3.811839928
112415	114735	7 -	KEX1	ORF-T	ORF-T	-0.089981227	-0.781825026	-0.397635799	-0.11450846	-0.54723132
117631	120975	7 -	MCM6	ORF-T	ORF-T	-0.281103734	-0.475394519	-0.574457083	-0.05543036	0.267240664
122591	123367	7 -	EMP24	ORF-T	ORF-T	-0.400075116	-0.32954976	-0.079203725	-0.248865128	0.395930494
123743	124263	7 -	unknown	no_ovlp	SUTs	0.866852336	-0.870040748	-0.87086335	-0.008744382	0.116703579
124263	124647	7 -	unknown	no_ovlp	SUTs	0.536848793	0.503412997	0.090398745	-0.276300998	-0.304252158
130974	131319	7 -	CUT587	CUTs	CUTs	2.510920736	-0.121044111	-0.700020003	0.057283116	0.481114719
139607	140031	7 -	YGL194C-A	ORF-T	ORF-T	0.778880788	-0.182740071	-0.413537814	-0.042970489	-0.595930368
140487	141783	7 -	HOS2	ORF-T	ORF-T	0.430786132	-0.151117125	-0.040451301	0.018571229	-0.079575759
141879	144135	7 -	YGL193C	ORF-T	ORF-T	0.165142377	-0.589174925	-0.260547519	0.185149051	-0.091524271
145615	147927	7 -	CDC55	ORF-T	ORF-T	-0.090980552	-0.501708997	-0.052654385	0.00557605	-0.376586416
148599	149015	7 -	RPS26A, YGL	ORF-T	ORF-T	0.698436744	-0.60567289	-1.007632807	-1.599534071	-0.690075102
150183	150551	7 -	YGL188C, CO	other	other	0.213696832	-1.088260004	-0.238421311	-0.220901193	-0.359373083
150999	152863	7 -	TPN1	ORF-T	ORF-T	-0.006691811	-0.713270615	-0.882411912	0.13016758	-0.412016645
152863	153103	7 -	unknown	no_ovlp2	SUTs	-0.359274783	0.311077024	0.13132963	-0.03493479	0.169465805
153103	154623	7 -	YGL185C	ORF-T	ORF-T	-0.499561453	1.179500459	2.122424409	-0.105555148	0.647548478
154343	154623	7 -	unknown	no_ovlp2	SUTs	-0.317023054	0.423420302	0.460879413	-0.038339682	0.121059801
154623	156263	7 -	STR3	ORF-T	ORF-T	-0.380344853	-0.243093129	0.599675159	0.084934898	1.347070099
156263	159751	7 -	SUT538	SUTs	SUTs	0.687543812	0.309693933	-0.101422863	-0.145220253	1.046672133
163327	165175	7 -	TOS3	ORF-T	ORF-T	1.484715997	0.087245507	-0.860950243	-0.441209993	-0.573211608
165175	165959	7 -	unknown	no_ovlp	SUTs	0.645068689	-0.275511472	0.162381788	-0.159739501	-0.241694657
166863	167487	7 -	CUT590	CUTs	CUTs	0.942582891	-1.174884159	-0.47840782	-0.554436394	-0.157788364
171311	173095	7 -	YGL176C	ORF-T	ORF-T	0.289945991	-0.229938477	-0.522922589	-0.327394793	0.448736112
173095	175375	7 -	SAE2	ORF-T	ORF-T	-0.113122072	-0.452754629	-0.022704749	-0.48321651	0.592157994
174351	175375	7 -	unknown	no_ovlp2	CUTs	2.261514488	0.268160693	-1.089862696	-0.37976537	-0.692105108
178047	180447	7 -	KEM1	ORF-T	ORF-T	-0.283804506	-0.425359118	-0.427973555	0.020268314	-0.271323265
180447	182103	7 -	unknown	no_ovlp	SUTs	0.218093066	0.232038053	-0.242543379	-0.002216122	0.547456047
187447	190895	7 -	PMR1	ORF-T	ORF-T	-0.222897956	-0.4233031	-0.369503516	-0.06364959	-0.33491588
191911	193327	7 -	YGL165C, YR	other	other	0.03537876	-0.631628288	0.025015261	0.178847565	-0.163296163
193703	196511	7 -	RAD54	ORF-T	ORF-T	0.123420199	0.019031928	0.14050522	-0.168841323	0.783311721
196511	197407	7 -	SUT540	SUTs	SUTs	-0.108956633	-0.462310603	-0.073672377	-0.424831384	-0.496821005
199071	200255	7 -	YIP5	ORF-T	ORF-T	-0.314103917	-0.364052214	0.266680542	0.115653156	0.096093256
210431	212719	7 -	SUT541	SUTs	SUTs	2.714102021	-0.460419221	-1.266026831	-0.54172451	-0.549325491
212719	213727	7 -	SUT541	SUTs	SUTs	0.415499811	-1.093373196	-1.043738562	-0.393404578	-0.46816253
215255	215895	7 -	LYS5	ORF-T	ORF-T	0.354502129	-0.187865583	0.346169241	-0.213184471	0.433867758
215895	217255	7 -	unknown	no_ovlp	CUTs	2.328941292	-0.180265903	-2.130912122	-0.123369766	-0.03513325
225751	226135	7 -	INO80	ORF-T	ORF-T	0.374010867	-1.142300715	-0.184673707	-0.50210384	-0.859817892
227639	228367	7 -	RPL9A	ORF-T	ORF-T	-0.468596208	-0.867598415	-0.38137527	-0.13534036	0.065673053
228511	229743	7 -	YGL146C	ORF-T	ORF-T	-0.722860917	0.402371712	1.61577036	0.735865644	-0.200720871
232383	236223	7 -	ROG1	ORF-T	ORF-T	0.259605569	-0.341437322	-0.574184628	-0.102558491	-0.055799886
234623	236223	7 -	unknown	no_ovlp2	SUTs	-0.859101934	-0.811143343	-0.036466066	0.056339131	-0.30798375
236223	238167	7 -	GPI10	ORF-T	ORF-T	0.065672429	-0.112708424	0.016876239	-0.214044314	0.171110107
241119	245199	7 -	YGL140C	ORF-T	ORF-T	-0.192423876	-0.556720203	-0.360036971	0.157421366	-0.126927196
252807	253919	7 -	MRM2	ORF-T	ORF-T	0.056858553	-0.337406294	0.156814542	-0.023265873	-0.087370465
256183	257263	7 -	CUT592	CUTs	CUTs	1.335282646	0.314054484	-0.248130299	0.352981025	0.713191061
260303	265935	7 -	SNT2	ORF-T	ORF-T	0.325545322	-0.118699931	-0.431945469	-0.193567043	-0.177424962
267615	269127	7 -	RSM23	ORF-T	ORF-T	-0.645011027	-0.874917386	-0.043981544	-0.052406985	-0.230934647
269127	270167	7 -	RSM23, CWC	ORF-T	ORF-T	0.673334045	0.609276045	0.05456191	-0.432616731	-0.056591241
270263	270831	7 -	SOH1	ORF-T	ORF-T	-0.771080212	-0.224958423	0.360359723	0.015684488	0.711978224
270831	272879	7 -	SUT542	SUTs	SUTs	-0.208586221	0.461759925	0.454266693	-0.019410404	0.206637909
274679	277071	7 -	MON1	ORF-T	ORF-T	0.337089033	-0.058131645	0.309734058	0.078657789	0.412217843
276751	277071	7 -	unknown	no_ovlp2	CUTs	2.440543804	0.437025771	0.488142861	0.026260503	-0.274013367
278775	280559	7 -	NAB2	ORF-T	ORF-T	0.999450566	-0.355768222	-0.410470122	0.11147281	-0.215515594
280559	281279	7 -	GPG1	ORF-T	ORF-T	0.085897626	0.304645729	1.871548357	0.599812739	0.699627279
281511	283991	7 -	PRP43	ORF-T	ORF-T	0.415788863	0.001909453	-0.880007298	-0.108071966	0.289718544
286295	289391	7 -	CUT594	CUTs	CUTs	1.944283492	0.221993234	-0.746847571	-0.152375327	-1.66921193
291191	291671	7 -	unknown	no_ovlp	CUTs	1.73536317	0.224945606	0.404565215	0.642897922	1.374019575
292767	293247	7 -	unknown	no_ovlp	SUTs	0.52778056	-0.680844175	-0.631541779	-0.743934136	0.227380705
298047	299767	7 -	TAF6	ORF-T	ORF-T	-0.094454062	-0.223626712	-0.256453911	0.064716479	0.371034032
301383	304087	7 -	CUE3	ORF-T	ORF-T	-0.139577231	-0.621154667	-0.208846598	0.143811609	-0.106162131
303503	304087	7 -	unknown	no_ovlp2	SUTs	-0.548556646	-0.234921818	0.288279456	0.481799213	0.083041454

304255	306287	7 -	RMD9	ORF-T	ORF-T	-0.326567272	-0.670808887	-0.178163944	-0.263760095	0.215533022
306287	307151	7 -	unknown	no_ovlp	CUTs	1.016082887	0.50908662	-0.848985989	-0.129242866	-0.212834277
308591	310191	7 -	VPS73	ORF-T	ORF-T	0.68568937	0.339397694	0.163106372	-0.163395193	0.434830025
314175	314383	7 -	CUT595	CUTs	CUTs	2.940469003	-0.150505052	-0.822829022	-0.217479956	0.021831126
322655	324095	7 -	SUT543	SUTs	SUTs	1.047331428	-0.032357404	0.239807879	0.182358758	0.553669958
324095	325943	7 -	unknown	no_ovlp	SUTs	0.458685262	1.076651545	0.85659395	0.655169709	-0.638281311
328895	330663	7 -	VPS45	ORF-T	ORF-T	0.003243446	-0.36862091	-0.108352985	-0.049786561	-0.289515819
330983	334647	7 -	PAN2	ORF-T	ORF-T	-0.222612849	-0.267457085	0.048680231	-0.088601278	0.265937207
337207	337591	7 -	CUT596	CUTs	CUTs	3.124867292	0.271838845	-0.6035679	-0.047125317	0.425244168
341919	343095	7 -	NBP35	ORF-T	ORF-T	0.021496766	-0.499782449	-0.099807549	-0.128993442	-0.146894511
343095	344807	7 -	unknown	no_ovlp2	SUTs	0.118827503	0.421131141	0.06799651	0.14102239	1.364810942
344807	345311	7 -	MF(ALPHA)2	ORF-T	ORF-T	-1.201865783	-0.371309377	0.366526864	0.429586608	0.238276656
344967	345311	7 -	unknown	no_ovlp2	SUTs	-0.412923762	-0.009943048	0.681015827	0.70522036	0.531504642
345311	345751	7 -	CUT597	CUTs	CUTs	1.996249998	0.600218056	0.196380626	0.409164817	-0.17101074
346359	346807	7 -	MMS2	ORF-T	ORF-T	-0.330826513	0.087170429	0.956585	0.453882049	-0.282659703
350543	352335	7 -	GUP1	ORF-T	ORF-T	-0.728934384	-0.490947014	-0.010469189	0.024285873	0.317724581
354319	355543	7 -	SUT544	SUTs	SUTs	0.695929371	-0.943095961	-0.261617355	0.311961048	-0.269832179
357415	358519	7 -	CUT598	CUTs	CUTs	1.65581668	0.186411006	-0.475455756	0.175356077	0.028350563
360143	361935	7 -	DBP3	ORF-T	ORF-T	0.21213143	-0.344146581	-0.82773107	0.033432523	-0.047199151
362167	364087	7 -	HNM1	ORF-T	ORF-T	0.018721915	-0.155583698	-0.96171074	-0.232864078	-0.286449577
364215	364967	7 -	unknown	no_ovlp	SUTs	-0.319897043	-0.427606827	-0.124301514	-0.104695045	-0.103740968
365527	365991	7 -	RPL7A	ORF-T	ORF-T	0.902976586	-0.637589762	-0.388092952	-0.754830353	0.257029292
366647	368191	7 -	MPS2	ORF-T	ORF-T	-0.206222366	-0.405191678	0.158599509	0.057178967	1.40018464
370479	371223	7 -	YGL072C	other	other	0.517177188	-0.976605718	-0.062538314	0.253467254	0.061547693
374167	374855	7 -	RPB9	ORF-T	ORF-T	0.108307469	-1.02873343	-0.184874112	-0.015867404	0.163751631
374855	377247	7 -	unknown	no_ovlp	SUTs	0.432890003	0.199010628	-0.29500896	-0.352509535	0.596743216
379735	381303	7 -	ALG2	ORF-T	ORF-T	-0.352024574	-0.282936894	0.072254386	0.034793713	-0.005580346
381479	383207	7 -	MRH4	ORF-T	ORF-T	-0.518413916	-0.542298245	-0.503793974	-0.881136744	-0.265729856
383487	384551	7 -	unknown	no_ovlp	SUTs	0.214361211	-0.310612459	0.220342112	0.407135837	0.782669161
388919	389783	7 -	DUO1	ORF-T	ORF-T	-0.338523343	-0.154003103	0.134605252	-0.032032521	1.020578969
391319	392007	7 -	unknown	no_ovlp	CUTs	2.023713376	0.395842408	-0.231707145	-0.12243426	0.429502005
394823	395799	7 -	YGL057C	ORF-T	ORF-T	-0.194578535	-0.423663188	-0.055085156	-0.204923164	0.317314406
395975	397743	7 -	SDS23	ORF-T	ORF-T	0.181697005	-0.214786997	-0.290410716	-0.508048398	-0.070646885
400583	401375	7 -	ERV14	ORF-T	ORF-T	0.130692272	-1.330817938	-0.564579917	-0.411616677	-0.610271201
401887	405143	7 -	unknown	no_ovlp	CUTs	1.231406959	0.335101645	-0.414807221	-0.240157535	-0.560105788
406703	409775	7 -	TIF4632	ORF-T	ORF-T	-0.528476452	-0.590844466	-0.219326461	0.184982564	-0.228897886
409983	411311	7 -	RPT6	ORF-T	ORF-T	0.052385495	-0.557081727	0.181116213	0.186247334	0.035862862
411311	412103	7 -	unknown	no_ovlp	CUTs	2.241679611	0.223770415	-0.851186977	-0.164275336	-0.513524079
412687	413183	7 -	CUT600	CUTs	CUTs	1.954351401	-0.341119985	0.449206231	0.964781005	-1.308374291
415871	417263	7 -	RNA15	ORF-T	ORF-T	0.304252041	-0.56999198	-0.277310491	-0.288915492	-0.024972006
419311	420663	7 -	HEM2	ORF-T	ORF-T	-0.581497664	-0.953353358	-0.298451685	0.135064448	-0.07872095
425111	426895	7 -	OCH1	ORF-T	ORF-T	-0.127040922	-0.533397909	0.115340863	-0.277838284	0.298420275
427191	428055	7 -	PNC1	ORF-T	ORF-T	-0.393094787	-0.780962048	0.470373016	0.21102183	-0.433940392
428055	428591	7 -	unknown	no_ovlp	SUTs	0.126845468	0.48419043	0.281434632	-0.263742694	0.20155977
431511	433167	7 -	MIG1	ORF-T	ORF-T	-0.397127134	-0.589044013	0.473355596	0.365521874	0.079796058
436479	436999	7 -	AGA2	ORF-T	ORF-T	0.373424525	0.428984749	0.954006192	0.395057445	0.772066855
436999	437247	7 -	unknown	no_ovlp	SUTs	-0.172725719	0.962728467	0.305081966	0.123796297	0.025414941
437943	438415	7 -	RPL24A	ORF-T	ORF-T	0.341186621	-0.533728625	-1.053744157	-0.809504969	0.026250559
439727	440543	7 -	CUT601	CUTs	CUTs	2.14521102	0.29237362	-0.250076886	-0.153347111	1.045306743
442247	443079	7 -	SCW11	ORF-T	ORF-T	-0.183065752	-0.249452599	-0.719582096	-0.500272274	0.282742084
443567	446167	7 -	CWH41	ORF-T	ORF-T	-0.506455009	-0.063959718	-0.325145308	0.16639532	-0.0345189
446311	448583	7 -	TRP5	ORF-T	ORF-T	-0.103841987	-0.071443101	-0.52837409	-0.03496045	0.035626812
448711	449991	7 -	PGD1	ORF-T	ORF-T	-0.136813383	-0.392596796	0.174173378	0.064861698	0.120393912
450087	452143	7 -	PIB2	ORF-T	ORF-T	-0.135214701	0.218933097	-0.258333471	-0.13777306	0.345228119
457119	457927	7 -	GET1	ORF-T	ORF-T	-0.104320196	-0.626342176	-0.415112844	-0.102788178	-0.371414069
459023	459703	7 -	JAC1	ORF-T	ORF-T	-0.735347394	-0.456279953	0.534960689	0.159441247	0.24007639
460847	461447	7 -	unknown	no_ovlp	CUTs	1.333650591	-0.039605846	-0.519347047	-0.126713467	0.608871008
469079	472431	7 -	PDR1	ORF-T	ORF-T	-0.113637465	-0.181005081	0.081231575	-0.225095846	0.389810691
474303	475255	7 -	SCL1	ORF-T	ORF-T	-0.092806641	-0.215670039	-0.073975045	0.023059309	0.045352204
476151	478727	7 -	LEU1	ORF-T	ORF-T	-0.229752303	-0.640853692	0.472871865	0.360051989	-0.002505934
479423	482903	7 -	PMA1	ORF-T	ORF-T	-0.417572292	-0.233759984	-0.142549884	-0.000626511	-0.102502085
482903	483975	7 -	SUT548	SUTs	SUTs	0.803692806	0.15561615	-0.947920508	-0.425199203	-1.012426317
484799	485351	7 -	unknown	no_ovlp	CUTs	1.806317067	-0.01296279	0.610185268	0.347239767	-0.919501289
489559	490551	7 -	COG7	ORF-T	ORF-T	0.562275485	-0.362753782	-0.308829201	-0.167217071	-0.419640332
490551	491959	7 -	RPN14	ORF-T	ORF-T	-0.011742741	-0.108548797	0.237754326	0.264928077	-0.249275788
493007	494279	7 -	CDH1	ORF-T	ORF-T	-0.179159721	-0.408422314	-0.355440813	-0.001362062	-0.067184699
495287	496575	7 -	ERG26	ORF-T	ORF-T	0.518215003	0.005610337	-0.63950492	-0.078373161	-1.333494449
497479	497935	7 -	AML1	ORF-T	ORF-T	0.589023412	-0.424954666	-0.83643347	0.051166939	-0.890517818
498239	499919	7 -	SWC4	ORF-T	ORF-T	0.127151337	-0.273245116	0.029245581	0.027530708	-0.152048027
504607	505887	7 -	TFG2	ORF-T	ORF-T	0.205035579	-0.331518129	-0.102017939	-0.054031627	-0.067449203
507959	508455	7 -	STF2	ORF-T	ORF-T	-0.207106656	-0.733652628	0.211372399	0.298555589	-0.349282491
508975	511047	7 -	SEC9	ORF-T	ORF-T	-0.26574691	-0.520267888	-0.07945546	0.098099173	-0.151336961
511551	512911	7 -	unknown	no_ovlp	CUTs	1.821781638	0.24175605	-1.043247457	-0.173045296	0.646120919
515903	516271	7 -	CUT604	CUTs	CUTs	2.988693149	0.532337224	0.540072849	0.003057522	0.857888312
520991	521951	7 -	YGR015C	ORF-T	ORF-T	0.221497859	-0.128515104	-0.243128379	-0.308761564	-0.342087728
524599	525615	7 -	CUT605	CUTs	CUTs	1.881359626	-1.160912332	-0.475843241	0.408149556	0.230205954
526847	527391	7 -	VMA7	ORF-T	ORF-T	-0.22390357	-0.29362617	-0.176756719	-0.169963808	0.226886891
527391	528735	7 -	unknown	no_ovlp	CUTs	1.371386419	-0.150723838	-0.928916333	0.152680686	0.335173126
531799	532647	7 -	THG1	ORF-T	ORF-T	0.11292981	-0.158143463	0.145653886	-0.402423679	0.387379723
534471	534799	7 -	RPS25A	ORF-T	ORF-T	0.34552977	-0.495144917	-0.870262259	-0.867250998	0.195516786
543471	544927	7 -	SUT550	SUTs	SUTs	-0.403505302	-1.720862418	0.512146089	0.469522937	-0.730298498
545615	546167	7 -	POP6	ORF-T	ORF-T	0.464666982	0.173673675	0.750208138	0.082683301	0.441871995

546167	547479	7	-	unknown	no_ovlp	SUTs	0.433646648	0.319768841	0.930967831	1.004700587	1.713638301
553975	555079	7	-	TIM21	ORF-T	ORF-T	-0.200677541	-0.651259622	-0.046599247	-0.375998927	0.310360883
556967	557551	7	-	YGR035C	ORF-T	ORF-T	1.050135088	-0.408786005	0.3050814	0.750538959	-0.340245656
557551	559039	7	-	CAX4	ORF-T	ORF-T	-0.082615877	-0.389323593	-0.24914665	-0.240486635	-0.057649752
559559	560055	7	-	ACB1	ORF-T	ORF-T	-0.73222958	-0.517702665	-0.29015697	-0.076624482	0.184196588
571983	575207	7	-	unknown	no_ovlp	CUTs	2.636614524	0.017624801	-0.108560122	-0.591083292	-0.667335734
575687	576527	7	-	CUT607	CUTs	CUTs	2.549422591	-0.204774085	-2.114088981	-0.727056203	-0.003079259
576527	579303	7	-	SUT551	SUTs	SUTs	0.737692001	0.511583441	1.017206156	0.313261457	0.339770253
580335	581719	7	-	NQM1	ORF-T	ORF-T	-0.460331166	-0.857787446	2.84968049	1.859678351	-1.807330773
581431	581719	7	-	unknown	no_ovlp2	CUTs	2.646471679	0.195143635	0.024779667	-0.24824709	-0.811250853
582759	584031	7	-	RME1, YGR0	other	other	0.525590017	0.525873442	0.407312163	0.150024295	0.657855599
584031	584519	7	-	YGR045C	other	other	0.00277075	-0.264194461	-0.032260161	-0.92136969	-0.674157021
586327	589487	7	-	TFC4	ORF-T	ORF-T	-0.29062823	-0.595422165	-0.2187341	-0.145083859	0.708499317
591263	592319	7	-	unknown	no_ovlp	SUTs	0.185452377	0.213066492	0.633402366	-0.149512751	-0.978139626
594919	595839	7	-	YGR053C	ORF-T	ORF-T	-0.943239063	-0.497438097	1.241831139	0.977777919	1.335302627
604455	605575	7	-	LST7	ORF-T	ORF-T	0.355509077	-0.18202163	-0.0769624	-0.571720605	-0.082377027
606079	607127	7	-	CUT608	CUTs	CUTs	1.91447147	0.211039001	-0.605698026	0.194960143	0.056421392
608599	609103	7	-	CUT609	CUTs	CUTs	3.829117896	0.466645838	-0.057028813	0.721793981	-1.91830747
611807	616071	7	-	ADE6	ORF-T	ORF-T	-0.09254071	-0.436277886	-0.542507711	0.022797708	-0.539462742
616071	617503	7	-	COX18	ORF-T	ORF-T	0.013382578	0.078303313	0.063357967	-0.134770536	-0.431757216
617503	617855	7	-	SPT4	ORF-T	ORF-T	-0.033961302	-0.216023675	-0.238498999	-0.271928226	0.083028765
618031	619975	7	-	VHT1	ORF-T	ORF-T	-0.582917219	-0.460182454	-0.226342479	0.726929346	0.640930142
622287	623583	7	-	YGR067C	ORF-T	ORF-T	-0.861707576	0.322109083	3.642057683	2.644906922	-0.032743588
623583	624935	7	-	YGR067C	ORF-T	ORF-T	-0.664030006	-0.098254984	3.696219326	2.486288129	0.21417679
624935	627135	7	-	YGR068C	ORF-T	ORF-T	-0.477547723	-0.357453136	0.014599196	-0.248059561	0.276499234
631311	634111	7	-	YGR071C	ORF-T	ORF-T	0.448475111	-0.08554817	-0.068020661	-0.236289642	0.050663247
635671	637015	7	-	YGR073C, PR	other	other	-0.298315073	-0.402421069	-0.343415571	-0.611479638	-0.449197007
637015	637623	7	-	MRPL25	ORF-T	ORF-T	-0.632883761	-0.864946451	-0.041847863	0.061372071	-0.098101386
637703	640407	7	-	PEX8	ORF-T	ORF-T	0.223097989	-0.240522141	-0.139549956	-0.039541551	0.287039167
639559	640407	7	-	unknown	no_ovlp2	SUTs	0.227336432	-0.039172567	-0.584531687	-0.325406858	-0.42485689
643007	643823	7	-	SLX9	ORF-T	ORF-T	0.878614885	-0.489262057	-0.552621333	-0.484246386	-0.002078617
644759	646879	7	-	GCD2	ORF-T	ORF-T	-0.101391933	-0.038762772	-0.089943275	0.165540588	0.271008022
647079	648095	7	-	MRP13	ORF-T	ORF-T	-0.34700062	-0.553701573	-0.047187366	-0.362422745	0.12706658
648303	648959	7	-	RPL11B	ORF-T	ORF-T	-0.165264104	-0.142783432	0.179177613	0.076889413	0.40331854
649271	650719	7	-	PIL1	ORF-T	ORF-T	-0.507139392	-0.495859137	0.352081566	0.311315229	0.349903497
652711	654279	7	-	unknown	no_ovlp	SUTs	0.170207179	0.297799962	0.922899098	1.213627	-0.196887312
656223	656455	7	-	CUT610	CUTs	CUTs	2.353905495	0.71844471	0.126528251	-0.17980642	-0.002711105
667343	667807	7	-	CUT612	CUTs	CUTs	3.124205385	-0.372885865	-0.477841464	-0.089902993	0.514795034
671495	671935	7	-	CUT614	CUTs	CUTs	3.074171595	0.243579585	-1.506226495	-0.207494113	0.564366603
675519	676415	7	-	RRP46	ORF-T	ORF-T	0.173977321	-0.994985128	0.171981086	0.214641275	0.487907983
676415	677559	7	-	SUT554	SUTs	SUTs	0.692341345	-0.233423523	0.837946538	0.852393703	0.270961124
677559	678287	7	-	SUT555	SUTs	SUTs	0.103158396	-0.320723733	0.255264617	0.191960091	-0.100335323
682335	687679	7	-	ESP1	ORF-T	ORF-T	-0.255530282	-0.050384502	0.103057143	-0.128130893	0.909666419
694367	695135	7	-	YGR102C	ORF-T	ORF-T	-0.063669966	-0.539058784	0.336752195	-0.075770889	0.515579309
697359	698439	7	-	SRB5	ORF-T	ORF-T	0.510597398	-0.319060447	-0.010567243	-0.362841105	0.17497203
698895	699847	7	-	YGR106C	ORF-T	ORF-T	-0.301095178	-0.743141276	-0.305764535	-0.052639641	0.201167799
702135	702663	7	-	CUT615	CUTs	CUTs	2.967938732	0.498444316	-0.663922255	-0.292758676	-0.323290071
715279	715607	7	-	SUT558	SUTs	SUTs	0.479550928	-0.519055152	0.477939615	-0.440213098	-0.021932588
719703	720095	7	-	unknown	no_ovlp	CUTs	1.934955051	0.420207585	1.156996112	0.850700862	1.614806021
724815	726495	7	-	YGR117C	ORF-T	ORF-T	-0.05550675	-0.741144679	-0.251870755	-0.419962952	0.036062727
727919	729767	7	-	NUP57	ORF-T	ORF-T	-0.171984515	-0.413061994	-0.192069392	0.11189973	0.172397381
729855	730863	7	-	COG2	ORF-T	ORF-T	0.098425805	-0.411083918	0.115703307	-0.388289191	0.128050292
731319	733015	7	-	MEP1	ORF-T	ORF-T	0.068768848	-0.428580608	-0.471228859	-0.158398827	0.008036483
733015	733535	7	-	CUT616	CUTs	CUTs	1.44251669	-0.688491825	0.208152116	0.06679914	-0.252521242
735175	735727	7	-	unknown	no_ovlp	SUTs	-0.542170003	-0.548182982	0.387507303	0.208039271	0.503037329
736503	738247	7	-	PPT1	ORF-T	ORF-T	0.203960547	-0.362327104	-1.086548405	-0.094529796	-0.157230898
739295	739647	7	-	CUT617	CUTs	CUTs	3.296522457	-0.392333104	-0.265283391	-0.454081949	-1.175882321
741551	742079	7	-	SUT559	SUTs	SUTs	0.803073196	-0.432483852	0.895490289	0.424331545	1.438555684
744615	746631	7	-	CUT618	CUTs	CUTs	1.552890887	0.260920147	-0.114026183	0.343910687	-0.067203526
747879	750143	7	-	UTP8	ORF-T	ORF-T	0.517239569	-0.344791622	-0.594856245	-0.095747816	-0.036094031
751231	753951	7	-	YGR130C	ORF-T	ORF-T	-0.286732982	-0.908903301	0.350364603	0.353961536	0.044779298
755375	756535	7	-	PHB1	ORF-T	ORF-T	-0.727040819	-0.73606401	0.007675154	0.00666573	0.148051069
756535	757415	7	-	SUT560	SUTs	SUTs	-0.803300143	-0.973676552	0.409489791	-0.338200707	0.376680773
761735	762159	7	-	CUT620	CUTs	CUTs	2.664809305	-0.49357151	-0.785770982	-0.051813736	0.601246177
763615	765879	7	-	TPO2	ORF-T	ORF-T	0.558381377	0.284340795	-0.401069142	-0.277428246	-0.37541299
765879	766847	7	-	unknown	no_ovlp	SUTs	0.429780352	0.4247889	0.563578438	0.292995885	0.236735578
771215	771951	7	-	unknown	no_ovlp	CUTs	2.356843467	0.275990183	-0.273776313	-0.156714546	-1.843015555
784023	785359	7	-	YGR146C	ORF-T	ORF-T	0.328988416	-0.546118888	0.837513908	0.64810151	0.232746099
785951	786975	7	-	NAT2	ORF-T	ORF-T	0.012351576	-0.601605113	-0.042311571	-0.269595922	-0.02676397
787783	788247	7	-	RPL24B	ORF-T	ORF-T	0.263857817	-1.148829263	-0.796678209	-1.218043181	-0.214101144
788247	788751	7	-	unknown	no_ovlp	SUTs	0.771386583	-0.186948749	0.067863832	-0.601083057	-0.722328807
790375	793095	7	-	YGR150C	ORF-T	ORF-T	-0.246079852	-0.611352506	-0.415404346	-0.36309398	-0.20259308
794583	795623	7	-	YGR151C, RS	ORF-T	ORF-T	-0.390676251	-0.79504933	-0.451476438	-0.050731219	0.189717697
796647	798143	7	-	GTO1	ORF-T	ORF-T	1.316188876	-1.040073267	-0.778768012	-0.785004484	-0.777495829
800975	801871	7	-	SUT564	SUTs	SUTs	0.853092062	-1.428593972	-1.069034473	-0.402249102	-0.023391136
805063	806095	7	-	MTR3	ORF-T	ORF-T	0.259258776	-0.077158153	-0.748685132	0.106380128	0.374155488
806279	807711	7	-	NSR1	ORF-T	ORF-T	-0.100120586	-0.438436125	-0.838072361	-0.089108195	-0.052667988
808375	809527	7	-	RTS3	ORF-T	ORF-T	0.134858372	-0.242862391	0.647182841	0.227046353	-0.30417644
810447	810903	7	-	unknown	no_ovlp	CUTs	1.001349776	-0.210774377	0.121415396	-0.414533925	-0.248822287
828575	828815	7	-	tR(UCU)G2	other	other	0.237788039	-1.239169746	-1.159670481	-0.524266714	-0.092431963
833271	834503	7	-	YGR168C	ORF-T	ORF-T	0.415295031	-0.319875838	-0.001446875	-0.306908466	-0.003935633
834503	836039	7	-	PUS6	ORF-T	ORF-T	-0.533449805	-0.449528711	0.087042802	-0.021443123	0.277661959

836039	836767	7	-	YGR169C-A	ORF-T	ORF-T	0.481032167	-1.148661937	-0.823580709	-0.514184162	-0.40708398
837303	840383	7	-	unknown	no_ovlp	CUTs	2.178033707	0.917774859	-0.223045025	0.164677834	0.240232372
840751	842719	7	-	MSM1	ORF-T	ORF-T	-0.21720565	-0.556763588	-0.260980995	-0.320512966	-0.801927953
842719	843631	7	-	YIP1	ORF-T	ORF-T	-0.38125572	-0.41782978	-0.355595291	0.052565418	-0.396652609
845799	846359	7	-	CBP4	ORF-T	ORF-T	-0.189403973	0.000137264	0.709706164	0.501273814	0.221874955
846775	848439	7	-	ERG1	ORF-T	ORF-T	-0.208857045	-0.297425145	-0.564512298	0.162900147	-0.421627779
848439	850503	7	-	ATF2	ORF-T	ORF-T	1.47665382	-0.865838127	-2.427225779	-0.120272297	-0.979935939
850943	853439	7	-	PBP1	ORF-T	ORF-T	-0.671298196	-0.39660548	-0.086408173	0.073048597	-0.289898982
853583	854919	7	-	OKP1	ORF-T	ORF-T	0.200641283	-0.505144612	-0.237530811	-0.404387692	0.245427681
855191	856511	7	-	RNR4	ORF-T	ORF-T	0.789975659	0.777825151	0.008738951	0.345323424	0.234152393
856511	858615	7	-	unknown	no_ovlp	SUTs	0.083571905	0.263866414	0.415949274	0.087414406	-0.224001294
858703	859263	7	-	YGR182C, Q(	other	other	-0.373847564	-0.852537554	0.649802576	0.098632198	0.168808169
859263	859575	7	-	QCR9	ORF-T	ORF-T	-0.166632858	-1.508003494	-0.013956226	-0.295022562	0.067154095
859831	866151	7	-	UBR1	ORF-T	ORF-T	-0.035125529	-0.135205848	0.024416106	-0.009015397	-0.09348556
866319	867559	7	-	TYS1	ORF-T	ORF-T	0.154861524	-0.157413075	-0.611316672	-0.056196964	-0.421409366
870103	871431	7	-	HGH1	ORF-T	ORF-T	-0.297945346	-0.723426421	-1.178934003	-0.308622692	-0.221005913
871431	871911	7	-	unknown	no_ovlp2	SUTs	-0.546208779	-0.077483891	0.36553659	-0.069370465	0.401925346
871911	875151	7	-	BUB1	ORF-T	ORF-T	-0.528703092	-0.086402657	0.355375995	-0.315577561	1.170276417
876567	878287	7	-	CRH1	ORF-T	ORF-T	-0.297426544	-0.105499468	0.308208259	-0.010813728	0.196580886
879047	879903	7	-	SUT566	SUTs	SUTs	2.995095446	0.343227193	0.78075054	0.209397882	-0.602050645
882311	882575	7	-	CUT627	CUTs	CUTs	2.533009651	-0.309429846	-1.803359262	-1.082858859	-0.460674958
882719	883903	7	-	TDH3	ORF-T	ORF-T	-0.202003601	0.022782475	-0.207052064	-0.133433174	-0.397862611
884407	885831	7	-	PDX1	ORF-T	ORF-T	-0.144470702	-0.489177851	-0.218996838	-0.092018329	-0.103417575
885975	887847	7	-	XKS1	ORF-T	ORF-T	-0.266201857	-0.502227229	0.744769895	0.241731439	0.853940222
889695	892223	7	-	FYV8	ORF-T	ORF-T	-0.212834845	-0.50957334	-0.210982652	-0.114344403	0.392877649
892471	894335	7	-	SNG1	ORF-T	ORF-T	-0.00704235	-0.699643394	-0.038615165	-0.169949904	-0.085479581
901383	902343	7	-	ELP2	ORF-T	ORF-T	0.089347815	-0.357356917	-0.612112501	0.127877586	0.138794356
902471	903159	7	-	YGR201C	ORF-T	ORF-T	0.54472614	1.128168061	1.309219788	0.557816082	1.337708029
903391	904847	7	-	PCT1	ORF-T	ORF-T	0.188748207	-0.581056314	-0.646839744	0.010326814	-0.363411189
904847	905735	7	-	SUT567	SUTs	SUTs	-0.038372017	-0.589598081	0.407822335	-0.453858665	-0.471465511
908847	910239	7	-	unknown	no_ovlp	CUTs	2.086140662	0.192457449	-1.260198881	-0.084979125	-0.698363584
910751	911687	7	-	YGR207C	ORF-T	ORF-T	0.324059638	-0.213696751	-0.222614753	-0.158484047	-0.160545873
912839	913311	7	-	TRX2	ORF-T	ORF-T	-0.990735542	-1.004758792	0.511640922	0.569881684	0.936458788
913415	914831	7	-	YGR210C	ORF-T	ORF-T	0.060133404	-0.122189716	-0.275214573	-0.060243019	0.137832195
919351	919855	7	-	unknown	no_ovlp	SUTs	0.848613109	-0.269829493	0.200818318	0.29812477	0.392332055
922503	924511	7	-	GPI1	ORF-T	ORF-T	-0.114770395	-0.26349764	-0.06283967	-0.308185765	0.19320903
931375	932007	7	-	unknown	no_ovlp	SUTs	0.364983351	-0.864372668	-0.466414543	-0.294215197	-0.780563802
936007	936895	7	-	MRPL9	ORF-T	ORF-T	-0.899953117	-0.677781298	-0.033404097	0.092922906	-0.049836967
937303	939087	7	-	TOS2	ORF-T	ORF-T	-0.782126714	-0.717420087	-0.608565031	-0.168757137	0.033960742
939479	939743	7	-	SNR7-L, SNR	other	other	0.020406012	0.240552775	0.090239943	-0.124459566	0.017638154
940807	942263	7	-	HSV2	ORF-T	ORF-T	-0.151420489	-0.632673436	0.317302389	0.252046873	0.216889239
943015	944823	7	-	SUT569	SUTs	SUTs	0.091185159	-0.635217389	-0.322634895	-0.408760246	-0.122454855
949192	951224	7	-	SMI1	ORF-T	ORF-T	-0.140056911	-0.330662028	-0.323134888	-0.178728517	-0.146236286
952328	953551	7	-	PHB2	ORF-T	ORF-T	-0.402376665	-0.693414118	0.146679035	-0.099683751	0.128829777
954615	958679	7	-	PHO81	ORF-T	ORF-T	-0.60990762	0.252600886	-0.077816184	0.813710782	0.609207761
958415	958679	7	-	unknown	no_ovlp2	CUTs	1.26806355	-0.861468071	-1.189272898	-0.77247249	-1.347381225
958679	959079	7	-	unknown	no_ovlp	CUTs	2.769340961	-0.146484571	-0.369447788	-0.537869634	-0.920548348
961255	962103	7	-	YGR235C	ORF-T	ORF-T	-0.411782876	-0.510035298	0.544667762	0.176688461	0.386570424
962103	962391	7	-	unknown	no_ovlp2	SUTs	-0.175612712	0.043612231	1.210116999	0.238389196	-0.240722875
962391	963110	7	-	SPG1	ORF-T	ORF-T	-1.206027555	-0.613904982	4.054430457	2.243911769	1.64181101
963110	965734	7	-	YGR237C	ORF-T	ORF-T	-0.018418171	-0.479350158	0.054018745	0.190597449	-0.159336102
965958	968750	7	-	KEL2	ORF-T	ORF-T	-0.833144516	-0.762240128	-0.10855857	0.310223755	0.712760483
969110	970070	7	-	PEX21	ORF-T	ORF-T	0.680936659	0.317905515	-0.062567907	0.065875374	0.411369315
970550	973974	7	-	PFK1	ORF-T	ORF-T	-0.200198605	-0.179998507	-0.224789849	0.052273081	-0.353257871
973974	974438	7	-	CUT630	CUTs	CUTs	2.098444358	-0.688152529	-0.206802262	-0.678530701	-1.180804861
974742	976758	7	-	YAP1802	ORF-T	ORF-T	0.093731012	-0.356904071	-0.036763103	-0.2083749	-0.181249243
977966	979430	7	-	LSC2	ORF-T	ORF-T	-0.249064732	0.276261556	0.748301523	0.646464008	1.156438093
979638	982158	7	-	SDA1	ORF-T	ORF-T	0.295662391	-0.100855338	-0.708898813	0.21181624	0.23596785
982334	984326	7	-	BRF1	ORF-T	ORF-T	-0.018493861	-0.136862526	0.371440587	0.052867725	0.579974726
985750	986718	7	-	unknown	no_ovlp	SUTs	0.425532975	-0.272473134	-0.174931401	0.579154008	-0.81378351
989166	989558	7	-	unknown	no_ovlp	CUTs	1.756122524	0.273291149	0.574874552	1.147061833	-1.558232183
991078	993726	7	-	YGR250C	ORF-T	ORF-T	1.015545177	0.297402058	0.323412828	0.185301527	-0.757374389
993726	994078	7	-	CUT632	CUTs	CUTs	0.831341126	-1.297054121	-0.086514355	-0.199211678	-0.124649841
995822	996438	7	-	unknown	no_ovlp	CUTs	2.483669005	0.50785637	-0.845476795	0.092136009	-0.849700449
998310	999198	7	-	PUP2	ORF-T	ORF-T	0.058334918	-1.058856207	-0.305093167	0.037991196	-0.337050107
999614	999910	7	-	SUT571	SUTs	SUTs	0.325527711	-1.488915225	-0.055272118	-0.437053621	-1.674219838
1002398	1004038	7	-	COQ6	ORF-T	ORF-T	-0.275278928	-0.289828899	0.34177986	0.036431141	-0.687307149
1006142	1007422	7	-	MTM1	ORF-T	ORF-T	-0.239589233	-0.221934353	-0.234891345	-0.224335681	-0.608856794
1007702	1010798	7	-	RAD2	ORF-T	ORF-T	0.237001251	0.06274739	0.135673579	-0.200195549	0.284935499
1014238	1016758	7	-	APL6	ORF-T	ORF-T	-0.072257337	-0.152480043	-0.174409877	-0.108055317	-0.447782573
1016942	1017790	7	-	BUD32	ORF-T	ORF-T	-0.088657359	-0.328092917	-0.054910081	-0.069841912	0.211675616
1017910	1019414	7	-	SAY1	ORF-T	ORF-T	-0.11628169	0.381790054	0.135543368	-0.111978948	0.051772715
1019414	1021862	7	-	MES1	ORF-T	ORF-T	0.218994829	-0.525368504	-0.917841552	-0.135462526	-0.499755349
1021862	1022366	7	-	SUT572	SUTs	SUTs	0.301661209	-0.064234052	-0.38126159	-0.669585453	1.259985824
1023398	1024846	7	-	unknown	no_ovlp	CUTs	1.494213323	0.330361779	-1.275747614	-0.306396293	-0.553640923
1024982	1025902	7	-	FOL2	ORF-T	ORF-T	-0.115481851	-0.921940914	-0.622950831	-0.068820553	-0.230108125
1025902	1026670	7	-	HUA1	ORF-T	ORF-T	0.68702553	0.352009552	-0.244204928	-0.132612625	-0.531958104
1026670	1027054	7	-	unknown	no_ovlp	CUTs	1.258869604	0.940556631	-0.007036866	-0.195017379	0.231730995
1037710	1038542	7	-	YGR271C-A, Y	ORF-T	ORF-T	0.230607174	-0.749029715	-0.680520344	-0.324496463	0.024180378
1039854	1043110	7	-	TAF1	ORF-T	ORF-T	0.206861034	-0.424268379	-0.347060568	-0.071585866	-0.045007477
1043766	1045510	7	-	RNH70	ORF-T	ORF-T	0.435990297	-0.256494105	-0.511570432	0.07072006	-0.157297789
1045582	1046574	7	-	YGR277C	ORF-T	ORF-T	0.01586274	-0.111009942	-0.208402686	-0.508996909	-0.198397459

1048758	1050102	7	-	SCW4	ORF-T	ORF-T	-0.274077548	-0.496364167	-0.086161653	0.012194365	0.041310878
1050702	1051766	7	-	PXR1	ORF-T	ORF-T	0.322378821	-0.21885316	-0.043449082	0.008341316	0.352819044
1051766	1052190	7	-	unknown	no_ovlp	SUTs	0.668565702	0.216660592	0.124736948	0.679167116	1.278776874
1057534	1058798	7	-	BGL2	ORF-T	ORF-T	-0.452466245	-0.230707368	0.052803865	-0.024689035	0.23567247
1058950	1060086	7	-	YGR283C	ORF-T	ORF-T	0.803972387	-0.006507297	-0.123548561	-0.363889396	0.514768708
1060086	1060342	7	-	unknown	no_ovlp	SUTs	0.311664996	0.348689731	-0.34623314	0.015794435	-0.31385382
1060486	1061614	7	-	ERV29	ORF-T	ORF-T	-0.884522466	-0.264690844	-0.10165298	-0.41305623	0.733925455
1061838	1063190	7	-	ZUO1	ORF-T	ORF-T	0.066751238	-0.358728338	-0.437612978	-0.160803029	-0.507933063
1063686	1065190	7	-	BIO2	ORF-T	ORF-T	-0.401274407	-0.241941311	0.537650473	1.201665412	0.775739861
1067150	1069702	7	-	unknown	no_ovlp	SUTs	-0.579403518	-0.436552273	0.623129486	0.833527069	-0.201193884
1073822	1076190	7	-	MAL11	ORF-T	ORF-T	0.107070874	-1.107014031	0.276720646	0.615991252	-0.857608778
1081270	1082782	7	-	unknown	no_ovlp	SUTs	0.107351633	-1.011609251	-0.225879094	-0.474551114	-0.766357721
14673	15089	7	+	ADH4	ORF-T	ORF-T	0.775780376	-0.231590627	-0.837499249	-0.73682183	-0.497201218
18905	20945	7	+	ZRT1	ORF-T	ORF-T	1.396070571	1.451130508	0.454627853	-1.089905094	-1.800175691
22609	23897	7	+	FZF1	ORF-T	ORF-T	0.732343266	0.588631622	0.532816555	-0.09770191	-0.345777789
23897	25593	7	+	HXK2	ORF-T	ORF-T	-0.230186107	-0.339990271	-0.432838887	-0.245333398	-0.270609756
27777	29073	7	+	SUT109	SUTs	SUTs	1.615629739	-0.092859033	-0.74985884	-0.264400982	0.102122784
31857	32873	7	+	RMR1	ORF-T	ORF-T	-0.85602233	-0.392126492	1.084019064	0.706847172	1.948886389
32873	33969	7	+	unknown	no_ovlp2	SUTs	-0.325234591	0.053157488	-0.001746775	0.071351613	2.561984494
33969	35417	7	+	ZIP2	ORF-T	ORF-T	-0.05523136	0.534619345	0.175058499	-0.104567859	2.565549663
35417	35657	7	+	unknown	no_ovlp	SUTs	-0.726122361	-0.993420795	0.193834538	0.378078775	0.056232233
35657	36785	7	+	PDE1	ORF-T	ORF-T	-0.429396639	-0.970008398	0.384202966	-0.257488025	-0.161534399
36785	37673	7	+	BRR6	ORF-T	ORF-T	0.402038604	-1.000763505	-0.517545063	-0.3958656	-0.258246918
39009	41217	7	+	GUS1	ORF-T	ORF-T	-0.015537801	-0.257525304	-0.518430112	-0.029533053	-0.558989317
41465	43289	7	+	RTF1	ORF-T	ORF-T	-0.297460405	-0.782465287	-0.329788228	-0.066658467	-0.178010848
43345	44601	7	+	TAD1	ORF-T	ORF-T	0.488557102	-0.11305655	-0.590338117	-0.307062828	-0.543890074
45433	48441	7	+	KAP114	ORF-T	ORF-T	0.024079607	-0.437199962	-0.596730175	-0.493311579	-0.279064983
48441	49553	7	+	DOC1	ORF-T	ORF-T	1.150850869	0.383316758	-0.399447502	-0.451786608	-0.026853124
49553	52529	7	+	CSE1	ORF-T	ORF-T	0.078595407	-0.520753985	-0.709965636	-0.252408814	-0.325004719
53457	56394	7	+	unknown	no_ovlp	SUTs	-0.129814007	0.478231307	0.446309315	0.154149096	1.67505077
56394	59002	7	+	ADE5%2C7	ORF-T	ORF-T	0.175412245	-0.052652455	-0.495610141	-0.17078007	0.05190031
59090	61938	7	+	SEC15	ORF-T	ORF-T	-0.256005486	-0.819900499	-0.350692254	0.084067598	-0.155342727
62202	63074	7	+	TAN1	ORF-T	ORF-T	0.197557417	-0.830341056	-0.630923757	-0.298404546	-0.14028319
63778	64418	7	+	SUT110	SUTs	SUTs	2.07784634	0.038025044	-0.124689975	-0.151916375	-1.427928441
67562	69490	7	+	SHE10	ORF-T	ORF-T	-0.097864404	-0.244531586	-0.051665337	0.176064669	0.249510801
69650	72642	7	+	VID30	ORF-T	ORF-T	-0.581959019	-0.631091677	0.589361452	0.235845822	0.240251358
73338	73794	7	+	YGL226W	ORF-T	ORF-T	0.075307421	-0.332671586	0.153002462	-0.276705598	0.240945994
74506	76866	7	+	SUT111	SUTs	SUTs	0.930038837	-0.430588509	-0.063564164	-0.091215609	0.045129291
76866	78026	7	+	VRG4	ORF-T	ORF-T	-0.482397425	-0.457754611	-0.748494828	0.108785829	0.21617124
78138	79210	7	+	unknown	no_ovlp	SUTs	-0.387143995	-0.590109483	-0.503630658	-0.31146764	-0.107154717
79210	79802	7	+	unknown	no_ovlp	CUTs	1.01081041	-1.068423518	-0.844385773	-0.033859523	0.004695746
81458	81850	7	+	unknown	no_ovlp	CUTs	2.473557517	-0.052571677	-1.71446856	-0.207124463	0.137193151
82458	82922	7	+	YGL220W	ORF-T	ORF-T	0.532466365	-0.20643735	0.11863599	-0.208924003	0.38865384
84626	87354	7	+	KIP3	ORF-T	ORF-T	-0.279662447	-0.241503107	0.054532036	-0.239587188	0.708578989
87522	87786	7	+	CLG1	ORF-T	ORF-T	-0.829015673	-0.044725358	-0.263887579	0.258953972	-0.428069903
91482	92522	7	+	VAM7	ORF-T	ORF-T	-0.168093326	-0.117487482	0.080358665	-0.041003174	0.369476171
92522	94579	7	+	NCS6	ORF-T	ORF-T	-0.009959333	-0.56433454	-0.451977173	0.006749057	0.227123384
93707	94579	7	+	unknown	no_ovlp2	SUTs	0.030064486	-0.046118578	-0.132313099	0.145247069	0.19167053
95147	95779	7	+	CUT126	CUTs	CUTs	2.603751125	0.175754824	-0.291981342	-0.591676798	-1.100664164
95779	97131	7	+	MIG2	ORF-T	ORF-T	0.593343338	-0.387161492	-0.499200945	-0.283796671	-0.644730809
97131	98691	7	+	SIP2	ORF-T	ORF-T	-0.533758994	-0.248016966	0.813236179	0.227246729	0.478349307
98899	102147	7	+	SPT16	ORF-T	ORF-T	-0.248704233	-0.473708507	-0.304449024	0.029158084	-0.337484947
114939	115931	7	+	unknown	no_ovlp	CUTs	1.971160203	-0.16069261	0.502993729	0.212279869	-0.373372452
115931	117667	7	+	ARO8	ORF-T	ORF-T	-0.267713186	-0.530645676	-0.600332381	-0.038286972	-0.699006075
122435	122859	7	+	unknown	no_ovlp	SUTs	0.505167983	-1.140753979	-0.020081031	0.324648024	-0.299677397
123595	124355	7	+	YIP4	ORF-T	ORF-T	-0.223625354	-0.097220475	0.405709109	-0.151892803	0.110548051
124355	124683	7	+	MDS3	ORF-T	ORF-T	-0.168314736	-0.212520562	0.461538839	0.039723812	0.625753648
131499	136899	7	+	GCN1	ORF-T	ORF-T	0.15388843	-0.239728913	-0.627024092	-0.00017111	-0.416781763
144403	144779	7	+	unknown	no_ovlp	SUTs	-0.134752555	-0.252333301	1.27428952	0.153148573	-1.024430454
144779	145451	7	+	COX13	ORF-T	ORF-T	0.260397493	-0.091466355	0.410640497	0.166483294	-0.234258726
153203	154035	7	+	CUT129	CUTs	CUTs	2.953046851	-0.592776952	-1.005629411	-0.102451868	-0.307643938
157867	159211	7	+	GTS1	ORF-T	ORF-T	-0.113756491	0.023795417	-0.144312936	0.045980601	0.148027307
159515	162187	7	+	ATG1	ORF-T	ORF-T	-0.121241409	-0.731387247	0.273440617	0.121429081	0.48872251
165451	166731	7	+	unknown	no_ovlp	CUTs	1.360766993	-0.033203611	-0.350605201	-0.383353573	-0.251288414
166731	167435	7	+	CUT130	CUTs	CUTs	1.175847185	0.749886681	0.631211449	0.343097991	-0.586610495
167435	167835	7	+	MPT5, YGL17	ORF-T	ORF-T	0.846938794	-0.031525014	0.574637432	0.19421285	-1.070546037
172755	174547	7	+	unknown	no_ovlp	CUTs	1.692589309	0.514427123	-0.436408388	-0.570260254	0.069478193
174547	175531	7	+	BUD13	ORF-T	ORF-T	0.02345878	-0.244734386	0.420938944	-0.149751318	0.693730261
180683	182211	7	+	NUP49	ORF-T	ORF-T	-0.150356987	-0.633061511	-0.599692818	-0.156141534	-0.282389981
182371	184187	7	+	ROK1	ORF-T	ORF-T	0.448120821	-0.275752777	-0.53738629	0.100309029	0.459957659
186011	187547	7	+	SUA5, HUR1	ORF-T	ORF-T	0.028135156	-0.65068525	-0.71804907	-0.155563172	0.196036632
190371	191131	7	+	unknown	no_ovlp	SUTs	0.284062867	0.30022825	-0.241901168	-0.004206615	-0.109147197
191131	191979	7	+	CUP2	ORF-T	ORF-T	0.011689375	-0.171674956	0.110948037	-0.395894238	0.438162697
197979	199147	7	+	SUT1	ORF-T	ORF-T	0.487404073	-0.294682234	-0.09467219	0.448476347	-0.883700013
200467	202411	7	+	YGL160W	ORF-T	ORF-T	-0.415701163	-0.737597031	0.01241569	0.170228636	-0.336469183
202707	203963	7	+	YGL159W	ORF-T	ORF-T	0.367273827	-0.753113941	-0.405714792	-0.080038031	-1.491448759
203963	204819	7	+	YGLWtau2	other	other	0.503202941	-0.840383576	-0.486358618	-0.245452229	-1.50727081
204819	206651	7	+	unknown	no_ovlp2	SUTs	-0.036679094	0.388597811	0.066378525	-0.121985828	-0.188377781
206651	208939	7	+	RCK1	ORF-T	ORF-T	-1.566463373	-0.514477937	2.081721875	2.712152182	-0.21864647
208659	208939	7	+	unknown	no_ovlp2	SUTs	-0.27986655	0.06418617	0.16366905	0.162233947	0.048291693
208939	210147	7	+	YGL157W	ORF-T	ORF-T	-0.093716213	-0.315634553	0.198779857	0.397696368	-0.645213208
210147	212907	7	+	AMS1	ORF-T	ORF-T	-2.045849623	1.483998645	3.677716361	1.502861967	1.920178405

213963	215611	7	+	CDC43	ORF-T	ORF-T	-0.410738176	-0.903393249	-0.482029548	-0.116878742	0.123129423
216267	217339	7	+	PEX14	ORF-T	ORF-T	-0.495340236	-0.342102499	0.478366691	-0.065564597	0.566723911
217483	220963	7	+	NUT1	ORF-T	ORF-T	0.196360848	-0.570974082	-0.420788165	-0.039316424	-0.335313726
225915	226355	7	+	CUT132	CUTs	CUTs	2.362081586	0.331218274	-0.600009832	-0.097636311	-0.532328128
226355	227667	7	+	ARO2	ORF-T	ORF-T	-0.2680113	-0.684733238	-1.060473675	-0.221378197	-0.232686576
228835	229563	7	+	CUT133	CUTs	CUTs	3.041256584	-0.060031931	-0.946023458	-0.32426672	-1.10730567
230219	231043	7	+	TIP20	ORF-T	ORF-T	-0.094269255	-0.469904791	-0.030301442	-0.136007604	0.059824957
234747	235923	7	+	unknown	no_ovlp	SUTs	0.605966658	0.305702469	0.003452244	0.22654237	0.854999975
238323	241187	7	+	HUL5	ORF-T	ORF-T	-0.003554775	-0.279161353	-0.062494659	-0.064370487	-0.136113969
245419	247339	7	+	FLC3	ORF-T	ORF-T	-0.284036025	-0.206624073	-0.106619428	0.168700588	0.491558494
249891	250107	7	+	SEC27	ORF-T	ORF-T	0.734374985	-0.524969643	-0.930929656	-1.222167414	-0.61398616
253083	254547	7	+	unknown	no_ovlp	SUTs	0.087456475	-0.194013125	0.297491697	-0.081930566	1.080441171
254595	255395	7	+	RPL1B	ORF-T	ORF-T	-0.740130025	-0.601711255	-0.187666889	-0.396730893	0.380398064
255395	257051	7	+	PCL10	ORF-T	ORF-T	0.206790946	-0.283524423	0.21375097	-0.302776661	0.360975611
257523	261587	7	+	ITC1, YGL132	other	other	0.047534102	-0.148436871	0.006227208	0.009937098	0.170716531
266115	267587	7	+	CEG1	ORF-T	ORF-T	0.263781744	-0.490802057	-0.354735453	-0.398646997	-0.48909562
269331	269819	7	+	CUT134	CUTs	CUTs	2.543681968	0.200872153	-0.485478243	0.057383258	0.378156688
270987	272387	7	+	SCS3	ORF-T	ORF-T	-1.009516359	-0.248455934	-0.497410443	-0.427851124	-0.079853573
272387	274787	7	+	MET13	ORF-T	ORF-T	-0.279457835	-0.262601913	0.072514096	0.057594372	0.251668295
277531	278475	7	+	RPS2	ORF-T	ORF-T	-0.410133682	0.049962645	-0.069761872	-0.07277658	0.084216377
280795	281155	7	+	CUT135	CUTs	CUTs	3.409281875	-0.038143876	-0.508287028	-0.566139458	0.078115307
284427	285915	7	+	ABC1	ORF-T	ORF-T	-0.073059941	-0.317654182	0.187635905	-0.268377524	-0.024269899
288443	289419	7	+	YGL117W	ORF-T	ORF-T	0.396167738	-0.382053443	-0.243005166	0.18611009	-2.171214135
289675	291739	7	+	CDC20	ORF-T	ORF-T	-0.218129211	-0.486549851	-0.061891354	-0.071558724	0.58774847
291931	293091	7	+	SNF4	ORF-T	ORF-T	-0.366686273	-1.029338652	-0.180116597	0.161398787	-0.593315917
293283	295699	7	+	YGL114W	ORF-T	ORF-T	-0.286165832	-0.582585049	-0.316084536	-0.007821447	0.021905122
295915	298163	7	+	SLD3	ORF-T	ORF-T	0.138114061	-0.1103649	-0.379571358	-0.418025047	0.315071873
299963	301539	7	+	NSA1	ORF-T	ORF-T	0.367291388	-0.04818253	-0.30977872	0.081582107	0.455675375
303643	304131	7	+	unknown	no_ovlp	CUTs	2.554332258	0.050492105	0.093756899	-0.328069089	0.591260037
306531	307267	7	+	MLC1	ORF-T	ORF-T	-0.031597149	-0.113053298	-0.184807138	-0.187569883	0.014112869
307387	308691	7	+	ARC1	ORF-T	ORF-T	-0.418837666	-0.371493182	-0.329353164	-0.072534708	-0.038750859
310923	311539	7	+	RPL28	ORF-T	ORF-T	0.927914344	-0.801480373	-0.099891071	-0.827322186	-0.113217732
312203	313075	7	+	YGL101W	ORF-T	ORF-T	0.309972459	-0.659940907	-0.79302726	-0.067749365	0.189179157
313211	314371	7	+	SEH1	ORF-T	ORF-T	-0.183387637	-0.413911526	-0.084416507	-0.375187586	-0.212636856
314619	316611	7	+	LSG1	ORF-T	ORF-T	0.173766406	-0.516069222	-0.892793664	-0.188961036	0.070715786
316795	317307	7	+	SNR82	other	other	0.443940769	-0.297192443	-0.347813145	-0.135480436	-0.608281263
316995	317307	7	+	unknown	no_ovlp2	CUTs	3.782660803	-0.980464128	-0.019610413	-0.10829174	-0.385329479
317307	317979	7	+	USE1	ORF-T	ORF-T	0.732903413	-0.764911306	-0.535444187	-0.022096836	-0.767867446
320003	320555	7	+	SUT114	SUTs	SUTs	1.659339624	-0.495467121	0.182541961	1.112267145	-0.197566017
321755	323347	7	+	SRM1	ORF-T	ORF-T	-0.282918801	-0.361814774	-0.314560339	0.299048569	0.356285826
324363	325347	7	+	SUT115	SUTs	SUTs	0.957659978	0.017348419	-0.171425766	-0.333653573	-0.023294859
325347	326083	7	+	TOS8	ORF-T	ORF-T	0.20992393	-0.207035171	0.068145774	0.483662841	0.270248204
334851	337683	7	+	SPC105	ORF-T	ORF-T	-0.179137136	-0.258120115	0.211630879	-0.088175688	0.65970723
337835	341851	7	+	NUP145	ORF-T	ORF-T	-0.061392978	-0.505020371	-0.575055529	0.063680985	-0.331609211
343299	345123	7	+	LIF1	ORF-T	ORF-T	0.406352439	-0.281502476	-0.302118965	-0.369866581	-0.350027443
345987	346331	7	+	YGL088W, SN	other	other	-0.253535169	0.316216244	0.03890411	-0.218916116	-0.12059926
346123	346331	7	+	unknown	no_ovlp2	CUTs	3.760760411	-0.865031829	-0.042228734	-0.097703989	0.130303439
347203	349451	7	+	MAD1	ORF-T	ORF-T	-0.094340828	-0.642491214	-0.117183227	-0.026137431	-0.126087423
349659	350619	7	+	YGL085W	ORF-T	ORF-T	0.138594633	-0.672979999	-0.122178115	-0.336278391	0.005957502
353003	355611	7	+	SCY1	ORF-T	ORF-T	-0.537167325	-0.292975747	-0.315281806	-0.258359479	0.58891607
355755	357147	7	+	YGL082W	ORF-T	ORF-T	-0.321770326	-0.652126463	0.053006528	-0.060177943	0.036579657
357147	358571	7	+	YGL081W	ORF-T	ORF-T	-0.031319311	1.444910467	1.738114143	0.255217276	3.149871289
358571	359291	7	+	FMP37	ORF-T	ORF-T	-0.388675956	-0.910360897	0.01314857	0.355141112	-0.01094392
359435	360251	7	+	YGL079W	ORF-T	ORF-T	0.120721711	-0.498529419	-0.024085174	-0.384692688	-0.145891736
366883	367491	7	+	CUT140	CUTs	CUTs	1.921753463	0.414060192	-0.502970966	-0.142161416	0.845554085
368475	368747	7	+	HSF1	ORF-T	ORF-T	-0.20577087	-0.823921983	-0.111343713	-0.17649588	-0.955049063
371579	374195	7	+	AFT1	ORF-T	ORF-T	-0.207235987	-0.971157517	-0.246598847	0.002639568	-0.10329632
375051	375835	7	+	MNP1	ORF-T	ORF-T	-0.592215566	-0.57838615	-0.316103362	0.074924826	0.117214634
376059	377307	7	+	NPY1	ORF-T	ORF-T	-0.001423478	-0.28540108	-0.351327922	-0.192994476	-0.61112736
377451	379827	7	+	SGF73	ORF-T	ORF-T	0.016888178	-0.446142283	-0.346098524	-0.128330601	-0.283830679
383443	385179	7	+	PUS2	ORF-T	ORF-T	-0.068773284	-0.494496029	-0.413801368	-0.388933585	0.165489279
385179	388835	7	+	PYC1	ORF-T	ORF-T	0.025680165	-0.097281508	0.357778114	0.400655698	0.499238604
389947	392203	7	+	YBP2	ORF-T	ORF-T	0.25326307	-0.229128894	0.055071329	-0.25188187	0.127203563
392203	393731	7	+	YGL059W	ORF-T	ORF-T	0.013521845	-0.629855254	0.178467569	-0.053075447	0.236997772
393939	394915	7	+	RAD6	ORF-T	ORF-T	0.119610635	-0.727851894	-0.691430067	-0.421464175	-0.344227007
398299	398579	7	+	unknown	no_ovlp	CUTs	1.112455522	-0.474519665	-0.154779188	-0.766817232	-0.475973722
398579	400467	7	+	OLE1	ORF-T	ORF-T	-0.318211525	-0.133195819	-0.550299925	0.050916692	0.056608042
402715	403531	7	+	unknown	no_ovlp	SUTs	0.743244575	0.247057671	0.419391967	0.191381271	-1.933974848
405139	405755	7	+	unknown	no_ovlp	SUTs	-0.26651894	-0.614070463	-0.067345858	-0.015984067	-0.757169354
405755	406795	7	+	TYW3	ORF-T	ORF-T	-0.279247121	-0.725404207	-0.458795817	0.100685756	-0.147399438
411499	412179	7	+	ALG13	ORF-T	ORF-T	-0.054693746	0.241414049	0.750186793	-0.365319767	0.863303855
413763	413987	7	+	unknown	no_ovlp2	SUTs	0.363463534	-0.362305551	-0.426701295	-0.013309961	-0.451798226
413987	415915	7	+	RIM8	ORF-T	ORF-T	0.156067581	-0.7434013	-0.289358747	0.133089438	-0.773679926
417475	418643	7	+	DST1	ORF-T	ORF-T	0.121472917	-0.185752262	-0.405927393	-0.252757004	-0.131353238
418819	419459	7	+	YGL041W-A	other	other	1.326987417	0.32068219	-0.265291106	0.140829734	-0.135634639
420915	421355	7	+	CUT142	CUTs	CUTs	2.558385683	0.224508648	-0.120245805	-0.306633677	-1.250266336
423859	425027	7	+	YGL039W	ORF-T	ORF-T	0.321067387	-0.053742536	-0.294643971	-0.149676603	-0.234066515
427243	427683	7	+	CUT143	CUTs	CUTs	2.61799561	0.073252503	-0.255019218	-0.125474155	0.798622561
428579	431363	7	+	YGL036W	ORF-T	ORF-T	-0.414586376	-0.303472961	0.185908857	0.323863201	0.634699305
433523	435099	7	+	SUT119	SUTs	SUTs	0.68081493	-0.368732815	0.548781376	0.384200159	-0.044069722
436195	436763	7	+	unknown	no_ovlp	CUTs	1.449036086	0.928661046	0.607167903	0.023099799	-0.638210845
439099	439339	7	+	RPL30	ORF-T	ORF-T	1.219577802	-0.675472039	0.288664109	-0.539943825	0.112414379

440027	440619	7	+	CGR1	ORF-T	ORF-T	0.910898574	-0.06536252	-1.737413823	-0.855305796	-0.012072919
443635	445891	7	+	unknown	no_ovlp	CUTs	1.404632377	0.06768515	-0.951132301	-0.620447425	0.473965287
448787	450043	7	+	unknown	no_ovlp	SUTs	0.999564751	-0.09396186	-1.225541425	-0.558704386	0.022534666
452347	454659	7	+	STT3	ORF-T	ORF-T	-0.619903694	-0.220422078	-0.098286506	0.035536547	-0.040462178
454843	457171	7	+	ALK1	ORF-T	ORF-T	-0.431422356	-0.183576141	-0.156663695	-0.135683833	0.583088783
458083	459099	7	+	CKB1	ORF-T	ORF-T	0.064819005	-0.392495375	-0.668252048	-0.008707834	-0.28593533
459875	461459	7	+	ATE1	ORF-T	ORF-T	-0.288787923	-0.353230002	0.142238793	0.0246088	-0.054036992
461627	465115	7	+	KAP122	ORF-T	ORF-T	0.448951436	-0.512871393	-0.755685096	-0.153619424	-0.172079164
465755	468891	7	+	PUF4	ORF-T	ORF-T	-0.172863898	-0.258489381	-0.782432273	-0.035864857	-0.330456907
472027	472827	7	+	unknown	no_ovlp	CUTs	1.122827661	0.502714239	-0.421880343	-0.577296678	-0.13094042
472827	474387	7	+	ERG4	ORF-T	ORF-T	-0.047019591	-0.512321879	-0.665685742	-0.352100631	-0.312716488
475523	476275	7	+	YGL010W	ORF-T	ORF-T	-0.027946256	-0.533396403	0.801072674	0.008927467	0.3780324
483963	484987	7	+	CUT144	CUTs	CUTs	3.128572942	0.219083277	-0.463408147	-0.543300532	-0.971308382
485515	489555	7	+	YGL006W-A,	other	other	-0.234267882	-0.846142095	-0.026998104	0.28149111	-0.323552303
490763	492419	7	+	unknown	no_ovlp	CUTs	1.480228104	0.26206884	-0.202341007	-0.06849547	-0.071375478
494507	495387	7	+	ERP6	ORF-T	ORF-T	-0.230915144	-0.808709126	-0.38287355	-0.114529014	-0.500314599
497915	498435	7	+	unknown	no_ovlp	SUTs	0.239328764	-0.77372006	0.297658726	-0.105438369	-0.818634599
500163	502651	7	+	CUL3	ORF-T	ORF-T	-0.133791423	-0.252120108	0.05632999	-0.034586193	0.310504817
502899	504571	7	+	PEX31	ORF-T	ORF-T	-0.182160821	-0.540724121	-0.975349438	-0.158669607	0.334452319
506075	506971	7	+	PRP18	ORF-T	ORF-T	0.1252198	-0.182702026	0.245010799	-0.193042321	0.121809035
506971	508083	7	+	MUQ1	ORF-T	ORF-T	0.082297912	-0.475157426	-0.777497344	-0.131161951	-0.402816181
508763	509899	7	+	CUT145	CUTs	CUTs	1.322981089	-0.031267549	0.422683705	-0.215922904	-0.218268404
511475	512947	7	+	NMA2, YGR0	ORF-T	ORF-T	-0.539287777	-0.565329701	-0.088488839	-0.097328848	-0.152044783
513163	514355	7	+	YGR012W	ORF-T	ORF-T	-0.461829908	-0.536096615	-0.407113723	-0.083931371	0.244364036
514539	516683	7	+	SNU71	ORF-T	ORF-T	0.02381147	-0.165569256	0.019039766	-0.083833784	0.277892938
516683	521003	7	+	MSB2	ORF-T	ORF-T	-0.133308179	-0.178612263	-0.803722914	-0.365141856	-0.008966019
522315	523627	7	+	YGR016W	ORF-T	ORF-T	0.166980755	-0.204745122	-0.068310502	-0.300125329	-0.251959714
523627	524795	7	+	YGR017W	ORF-T	ORF-T	-0.095630751	-1.114851767	-1.103044964	-0.392192934	-0.790287743
525211	526739	7	+	UGA1	ORF-T	ORF-T	-0.728105832	-0.116938026	0.849780216	0.568378329	0.437824775
527651	528555	7	+	YGR021W	ORF-T	ORF-T	-0.826666704	-0.56615369	0.320682052	-0.58911207	0.3178045
529091	531139	7	+	MTL1	ORF-T	ORF-T	-0.365888275	-0.877648452	0.161254154	0.278466156	0.024911287
532843	533955	7	+	YGR025W, YG	other	other	-0.382134745	-0.54832843	-0.026253547	-0.324560587	0.158448576
535131	535595	7	+	unknown	no_ovlp	SUTs	0.532497686	-1.383885625	0.079497714	-0.016382317	-0.86768702
542203	543443	7	+	MSP1	ORF-T	ORF-T	-0.435636547	-0.82545777	-0.038641538	0.031539487	-0.344228305
543738	544419	7	+	ERV1	ORF-T	ORF-T	1.191349785	0.35269332	-0.179211657	-0.10606781	-0.396925264
545139	545387	7	+	CUT146	CUTs	CUTs	1.616344065	-1.094402491	-1.019750923	-0.565258386	-0.846056377
545387	545643	7	+	SNR46	other	other	0.260962515	-0.35359582	-0.059833728	-0.196571635	0.00198115
546027	546363	7	+	unknown	no_ovlp2	CUTs	3.164870545	0.21754866	-1.274166931	-0.660945666	-0.993633752
546363	547851	7	+	YGR031W	ORF-T	ORF-T	0.351751728	0.224208838	0.063961492	0.059423204	-0.309297913
547851	554067	7	+	GSC2	ORF-T	ORF-T	-0.606008817	-0.127381156	0.988614007	1.048111743	-0.767838985
555835	556315	7	+	RPL26B	ORF-T	ORF-T	0.474980036	-1.035523103	-1.034556742	-0.666463637	-0.04407891
559211	559555	7	+	SUT122	SUTs	SUTs	1.600545088	-0.701073126	-0.572697436	-0.16818065	-0.179267569
560563	561499	7	+	ORM1	ORF-T	ORF-T	-0.353434815	-1.170507425	-0.625713214	-0.367298786	-0.023615255
567763	568179	7	+	unknown	no_ovlp	CUTs	2.172753659	0.33712036	0.218411584	-0.286202501	-0.779161402
574707	574931	7	+	unknown	no_ovlp2	SUTs	0.75291446	0.493680391	0.404153365	-0.040855817	-1.722049242
574931	575339	7	+	YGR039W, KS	other	other	-0.113441186	-0.463693985	0.234118257	-0.506963342	-1.132687417
575379	577107	7	+	KSS1	ORF-T	ORF-T	0.603779568	-0.197190703	-0.448848123	-0.423586386	-0.47546309
576883	577107	7	+	unknown	no_ovlp2	SUTs	0.456424392	-0.007507752	-0.25938613	-0.131715842	-0.087950129
577107	578651	7	+	BUD9	ORF-T	ORF-T	-0.064076813	-0.478200537	-1.289599879	-0.308864848	0.039448113
578651	579451	7	+	unknown	no_ovlp2	SUTs	0.160502384	-0.758090834	-1.198007536	-0.298017398	-0.416294917
579451	580363	7	+	YGR042W	ORF-T	ORF-T	0.491192147	0.086307921	-0.037562877	-0.214648631	0.135772375
582067	582891	7	+	unknown	no_ovlp	CUTs	1.556822279	-0.315691508	-0.36031799	-1.013629889	-1.725811337
584379	584779	7	+	unknown	no_ovlp2	CUTs	2.137507236	0.058910226	0.275768068	0.025775254	-0.231685842
584779	586315	7	+	TAM41	ORF-T	ORF-T	0.021818555	-0.562370823	0.225766191	0.301854925	-0.145022211
589771	590987	7	+	UFD1	ORF-T	ORF-T	0.321232045	-0.413134673	-0.233074538	-0.400524961	-0.509942022
591251	591987	7	+	SCM4	ORF-T	ORF-T	0.920081264	0.401193656	-0.411017233	0.692184501	-0.300486295
593259	594907	7	+	FMP48	ORF-T	ORF-T	-0.879873012	-0.951638034	1.566541615	1.611626014	0.079623284
596531	598835	7	+	YGR054W	ORF-T	ORF-T	-0.102642205	-0.226006015	-0.824600528	0.00946597	-0.47383061
598995	599395	7	+	unknown	no_ovlp	SUTs	0.087238839	0.620153394	0.488458435	0.26922537	0.050653148
599395	599691	7	+	MUP1	ORF-T	ORF-T	0.190637621	0.068472026	-1.078872176	-0.547590766	-0.213505982
601627	604563	7	+	RSC1	ORF-T	ORF-T	-0.674786789	-0.793363174	-0.459107987	-0.032247209	-0.197293756
605763	606123	7	+	unknown	no_ovlp2	CUTs	2.664338506	-0.499323815	-0.569830552	-0.607843998	-0.784906453
606123	607483	7	+	PEF1	ORF-T	ORF-T	0.151489937	0.010851031	0.027355664	-0.202356474	-0.344242888
607483	609403	7	+	SPR3	ORF-T	ORF-T	-0.123703482	0.919359711	0.57817392	0.062097248	-0.070406698
609139	609403	7	+	unknown	no_ovlp2	SUTs	0.034929525	0.524236349	0.12954038	-0.075084382	-0.047267179
609891	610099	7	+	unknown	no_ovlp	CUTs	2.681444347	-0.424923315	-0.849930455	-0.826905925	0.596744145
610507	611827	7	+	ERG25	ORF-T	ORF-T	-0.425683905	-0.071253241	-0.34382901	0.1422815	-0.149857905
616307	617827	7	+	CUT151	CUTs	CUTs	1.997753612	0.114015372	-0.730997325	-0.653891014	-0.505294823
620331	622379	7	+	SUT123	SUTs	SUTs	0.911454826	0.094175652	0.116595481	-0.557694789	-0.232169444
625251	627347	7	+	SUT124	SUTs	SUTs	0.097491135	0.279736404	1.708464762	1.560851543	0.940545856
627347	628571	7	+	unknown	no_ovlp	SUTs	0.350649193	-0.080677331	0.812197254	0.449750253	0.091597612
634275	635467	7	+	UPF3	ORF-T	ORF-T	0.428022201	0.007178257	-0.142213818	-0.35898453	0.051429824
635699	636451	7	+	SMD1	ORF-T	ORF-T	0.305653874	-0.704018244	-0.917084068	-0.346366817	-0.442050281
640819	641907	7	+	YGR079W	ORF-T	ORF-T	0.47553368	0.300746764	-0.247650853	-0.228808976	1.673398163
641995	643123	7	+	TWF1	ORF-T	ORF-T	-0.141325929	0.001150759	0.394597142	-0.000218037	0.227487386
644011	644755	7	+	TOM20	ORF-T	ORF-T	-0.375199019	-0.588388523	-0.467892364	-0.125371643	-0.032713311
650571	650835	7	+	unknown	no_ovlp	SUTs	-0.261937393	0.342940825	0.005146899	-0.036595824	0.000704448
650835	652083	7	+	SUT125	SUTs	SUTs	0.572064471	0.433013049	0.654339436	1.14270198	0.453891412
654595	655907	7	+	CTT1	ORF-T	ORF-T	-1.394699135	-2.410704047	1.042630288	1.032260008	0.133362647
656707	658715	7	+	NNF2	ORF-T	ORF-T	0.374967834	-0.046749528	-0.429903466	-0.105893084	-0.167465344
661915	662147	7	+	unknown	no_ovlp	SUTs	-0.966148233	-0.994741275	0.032488855	-0.881175144	-0.662027954
662147	666179	7	+	UTP22	ORF-T	ORF-T	0.09619302	-0.382487102	-0.858252183	-0.222987114	-0.331976627

666179	667979	7 +	PRP31	ORF-T	ORF-T	0.088269497	3.81E-05	-0.298992067	-0.385535525	0.095811647
668219	670067	7 +	DBF2	ORF-T	ORF-T	0.056931127	-0.071835952	0.335732966	0.14250665	0.385083149
670355	671915	7 +	YGR093W	ORF-T	ORF-T	-0.059871337	-0.271816473	0.107227615	0.067494072	0.429830012
672227	675611	7 +	VAS1	ORF-T	ORF-T	-0.330882959	-0.679406039	-0.821089937	0.002965781	-0.433491147
676627	677643	7 +	TPC1	ORF-T	ORF-T	-0.052620681	-0.57770424	0.052388049	-0.549300483	0.201693471
678235	678459	7 +	unknown	no_ovlp2	CUTs	2.086454195	-1.369772212	0.110762996	0.051239358	-0.108321038
678459	682371	7 +	ASK10	ORF-T	ORF-T	0.282302578	-0.154836386	-0.313892037	0.100592647	-0.453002636
687843	690179	7 +	TEL2	ORF-T	ORF-T	-0.221225636	-0.437385469	0.35656388	0.041313107	1.168526822
690179	693235	7 +	MDR1	ORF-T	ORF-T	-0.044542647	-0.336498126	0.107127581	0.079779823	-0.338120029
693315	694499	7 +	PCP1	ORF-T	ORF-T	-0.706549979	-0.629554281	0.266675879	0.011399169	0.304487906
695395	697403	7 +	NOP7	ORF-T	ORF-T	0.086554349	-0.35007701	-0.632331656	0.038531352	0.237672532
698555	699019	7 +	VMA21	ORF-T	ORF-T	0.477252592	-0.069452398	-0.056724619	-0.234574117	0.14435884
700075	700827	7 +	CUT153	CUTs	CUTs	2.399723456	-0.058219161	-0.437765553	-1.002678692	-0.217081441
701755	703259	7 +	unknown	no_ovlp	CUTs	1.310838699	0.031619099	-0.393628206	0.121101735	-1.029666693
703259	705243	7 +	CLB1	ORF-T	ORF-T	-0.268606041	-0.690865317	-0.724216411	0.06071202	0.734728599
713755	715803	7 +	YGR110W	ORF-T	ORF-T	-0.871218354	-0.359809736	1.960750361	1.098092559	1.984466043
715083	715803	7 +	unknown	no_ovlp2	SUTs	-0.320810585	0.091947361	1.152218137	0.768532563	0.543537768
715803	717155	7 +	YGR111W	ORF-T	ORF-T	0.216762267	-0.120732922	0.398936286	0.092518165	-0.274402661
717315	718715	7 +	SHY1	ORF-T	ORF-T	0.017021205	0.405062326	0.69738257	0.301431585	0.753336298
718843	720243	7 +	DAM1	ORF-T	ORF-T	-0.055374189	-0.38873699	-0.286306199	-0.311881227	-0.058405358
720347	724947	7 +	SPT6	ORF-T	ORF-T	-0.249178998	-0.509241281	-0.713426489	0.0290822	-0.285821054
727043	727387	7 +	RPS23A	ORF-T	ORF-T	0.191380637	-1.129849418	-0.87001697	-0.897103319	-0.481938438
729939	731363	7 +	SUT127	SUTs	SUTs	1.146778544	-0.04357489	-1.04228487	-0.535730293	0.432839835
731363	733515	7 +	SUT128	SUTs	SUTs	0.672121103	-0.630906163	-0.379061742	0.107246176	0.096657581
733515	733787	7 +	unknown	no_ovlp2	CUTs	1.568342034	-1.584836041	0.205446394	0.016212323	0.309304567
733787	735387	7 +	YGR122W	ORF-T	ORF-T	0.166931982	-0.771391109	-0.171732681	-0.177303057	0.139144174
738443	739411	7 +	SUT129	SUTs	SUTs	1.67488148	-0.298442944	-1.288375905	-0.918331308	-0.657195075
739411	739923	7 +	CUT155	CUTs	CUTs	2.010482329	-0.557564269	0.305740193	0.149599979	0.099541373
739923	741731	7 +	ASN2	ORF-T	ORF-T	-0.216317771	-0.292670231	-0.34200064	-0.084662281	-0.473484615
742131	745571	7 +	YGR125W	ORF-T	ORF-T	-0.092292598	-0.494758013	-0.1741644	-0.083717966	-0.111245791
745739	746579	7 +	unknown	no_ovlp2	SUTs	0.760741082	-0.024957392	-0.552388927	-0.425339543	-0.88233442
746579	747955	7 +	YGR127W	ORF-T	ORF-T	0.909934998	-0.168041627	-0.054167674	0.003624314	-0.726016759
750387	751347	7 +	SYF2	ORF-T	ORF-T	0.793749058	-0.0959374	0.126106675	-0.296410408	0.844933416
753091	754163	7 +	unknown	no_ovlp2	CUTs	2.19078464	0.677042164	-1.28170124	-0.860424545	-0.411781893
754163	755379	7 +	YGR131W	ORF-T	ORF-T	0.355721285	-0.070687047	-0.016163327	1.045525937	0.245997787
756947	757587	7 +	unknown	no_ovlp2	SUTs	-0.020282771	0.249922604	0.872949303	-0.362233871	0.342834844
757587	761347	7 +	CAF130	ORF-T	ORF-T	-0.093706423	-0.374311306	-0.717285363	-0.276987014	-0.553406436
761347	762243	7 +	PRE9	ORF-T	ORF-T	-0.021855622	-0.424894704	0.338583979	-0.075513516	-0.017093772
762435	763387	7 +	LSB1, YGR13	ORF-T	ORF-T	-0.023922006	-0.211572295	0.410269289	0.088420459	0.092253222
766707	767483	7 +	unknown	no_ovlp2	CUTs	2.221978971	-0.212708716	-0.207755042	-0.637377894	-0.625783333
767483	770379	7 +	CBF2	ORF-T	ORF-T	0.353184836	-0.385084876	-0.121881331	-0.051774457	0.689862966
770675	772403	7 +	VPS62	ORF-T	ORF-T	-0.439862955	-0.244549124	0.189002955	0.244151812	-0.067284864
772051	772403	7 +	unknown	no_ovlp2	SUTs	0.355375656	-0.439574332	-0.201749471	-0.379946698	-0.104191863
772403	773787	7 +	BTN2	ORF-T	ORF-T	1.63444321	-0.26817341	0.334664658	0.3658466	-1.120804498
774747	777915	7 +	SKN1	ORF-T	ORF-T	0.528168602	-0.084876472	-0.296292279	-0.024879107	-0.228254615
778187	779235	7 +	SUT131	SUTs	SUTs	0.628385102	0.177629213	0.700409222	0.651027243	-1.408340555
780187	781483	7 +	THI4	ORF-T	ORF-T	0.167159864	0.113089273	0.66545847	1.021751237	-1.497650998
781731	783995	7 +	ENP2	ORF-T	ORF-T	0.354576388	-0.304409011	-0.692461823	0.121099763	0.237897242
785939	786419	7 +	CUT156	CUTs	CUTs	1.392924213	-0.489105219	-0.618157028	0.731248448	0.3135502
788987	790523	7 +	YGR149W	ORF-T	ORF-T	-0.114691024	0.030904507	0.372302975	0.096957478	0.665685853
793283	793587	7 +	YGRWdelta2	other	other	0.891419124	-0.826660949	-0.049503052	-0.309751769	-0.160694319
793587	796083	7 +	unknown	no_ovlp2	SUTs	0.586747555	0.308157844	-0.410896748	0.022475926	0.356283729
796083	798499	7 +	YGR153W	ORF-T	ORF-T	-0.46526052	-0.272187873	0.810209718	0.433620637	1.029425323
796771	798499	7 +	unknown	no_ovlp2	SUTs	0.700950145	0.558442885	-0.349009253	-0.358413542	0.116059486
798499	800219	7 +	CYS4	ORF-T	ORF-T	-0.186125848	-0.669833232	-0.83117157	-0.162009437	-0.071144728
800411	801899	7 +	PTI1	ORF-T	ORF-T	-0.686693226	-0.570337921	-0.064989814	0.178679074	-0.331889556
802259	805227	7 +	CHO2	ORF-T	ORF-T	-0.449899096	-0.492859015	-0.412128352	-0.023764165	-0.145768919
809995	810763	7 +	YGR161W-C	ORF-T	ORF-T	-0.396071711	-0.843349159	0.813567144	0.593042977	-0.162421674
824003	827363	7 +	TIF4631	ORF-T	ORF-T	-0.087402681	-0.30524622	-0.690755123	-0.082603073	-0.278921776
827515	828659	7 +	GTR2, YGR16	other	other	-0.052441637	-0.78477729	-0.138938213	0.070301655	-0.265752792
829091	830315	7 +	MRPS35	ORF-T	ORF-T	-0.703643836	-0.821380836	-0.100211621	-0.166195793	-0.092354333
830459	832275	7 +	KRE11	ORF-T	ORF-T	0.425579155	-0.063386847	-0.850050569	-0.539205848	-0.314962097
832387	833347	7 +	CLC1	ORF-T	ORF-T	-0.442536719	-0.541581136	-0.206704801	-0.23275756	-0.406472198
837115	839707	7 +	PSD2	ORF-T	ORF-T	-0.602770296	-0.793456975	-0.667779984	-0.295732091	-0.165992278
843819	845235	7 +	RBG2	ORF-T	ORF-T	0.401467283	-0.1825866	-0.509520773	-0.131458849	-0.151213262
845723	846523	7 +	unknown	no_ovlp2	SUTs	-0.75140881	-1.124366221	0.298825531	0.293575538	0.072829487
846523	846843	7 +	YGR174W-A	ORF-T	ORF-T	-0.026530964	-0.258654186	1.542248374	0.822163415	-0.183327868
848707	849411	7 +	unknown	no_ovlp	SUTs	0.448732102	0.266989236	0.37265806	0.411315911	-0.273377543
850803	853227	7 +	unknown	no_ovlp	CUTs	2.260859533	0.150523674	-2.092999285	-0.193625852	-0.64128037
858251	858787	7 +	TIM13	ORF-T	ORF-T	-0.121146204	-0.121866362	0.033386543	-0.3212814	-7.61E-05
859835	860155	7 +	CUT159	CUTs	CUTs	2.935713125	0.149826574	0.344329168	0.289016176	0.694427663
867763	870083	7 +	TFG1	ORF-T	ORF-T	0.054584811	-0.35790825	-0.140132393	-0.052889795	-0.294529829
875395	876235	7 +	YGRWdelta3	other	other	0.786432978	0.11501015	0.613740289	-0.527407557	0.660192212
879635	880211	7 +	unknown	no_ovlp	CUTs	1.55997818	0.468348529	-0.724004016	-0.287344247	-1.267645454
880211	882467	7 +	HIP1	ORF-T	ORF-T	0.051749318	-0.626722308	-0.599090643	-0.070463298	-0.374036647
882571	884507	7 +	unknown	no_ovlp	SUTs	0.212695142	0.390757499	-0.165188216	-0.180296902	-0.04198378
884507	884867	7 +	CUT160	CUTs	CUTs	3.444077837	0.175764194	-0.99025107	-0.421725878	-0.392721329
888795	889699	7 +	SKI6	ORF-T	ORF-T	0.065951723	-0.304679103	-0.314238781	-0.100284525	0.224398021
894659	897243	7 +	YPP1	ORF-T	ORF-T	0.091530978	-0.593170254	0.087920005	-0.111106314	0.272468355
897483	900115	7 +	PMT6	ORF-T	ORF-T	-0.941552464	-0.824631917	-0.265046768	0.079173522	0.129968667
905195	905907	7 +	YGR203W	ORF-T	ORF-T	-0.933719928	-0.771195146	0.133698408	-0.078356561	-0.544673128
905907	908899	7 +	ADE3	ORF-T	ORF-T	-0.010507464	0.073187052	-0.4868888	0.020848114	-0.605271028

909099	910211	7	+	YGR205W	ORF-T	ORF-T	-0.647172929	-1.082282948	0.5437751	0.719491415	0.08736044
910443	910827	7	+	MVB12	ORF-T	ORF-T	-0.17321312	-1.009885182	-0.550700835	0.235632916	-0.169174073
911843	912835	7	+	SER2	ORF-T	ORF-T	0.613697344	0.110537306	-1.224269951	-0.642081677	-0.297173688
913499	915155	7	+	unknown	no_ovlp	SUTs	0.460619563	0.257918826	0.186269127	0.490101617	0.807563645
915155	916907	7	+	ZPR1	ORF-T	ORF-T	0.32817918	-0.048095058	-0.080635736	0.024189416	0.095339108
917115	918499	7	+	SLI1	ORF-T	ORF-T	0.207459193	-0.549696432	-0.185296423	-0.151757424	0.117839867
920667	921139	7	+	RPS0A	ORF-T	ORF-T	0.251514064	-1.015459379	-0.864209428	-1.051891383	0.399812872
922123	922635	7	+	RSM27	ORF-T	ORF-T	-0.967503724	-1.225598219	-0.099329651	-0.365626633	-0.063331103
924667	927579	7	+	CCH1	ORF-T	ORF-T	-0.17169403	0.11737074	-0.599904873	-0.643101587	0.080807304
932259	935979	7	+	CRM1	ORF-T	ORF-T	-0.112552451	-0.468182604	-0.112588832	0.160402096	-0.304190947
937139	938267	7	+	SUT138	SUTs	SUTs	0.086598598	0.64624242	0.167492545	-0.605892835	-1.265660902
939931	940931	7	+	PET54	ORF-T	ORF-T	0.142821951	-0.380967661	-0.245318139	-0.660954932	0.352202432
940931	945043	7	+	unknown	no_ovlp2	SUTs	-0.134801205	0.383927966	0.128043857	0.180652791	0.368018144
945043	947364	7	+	AMA1	ORF-T	ORF-T	-0.710610966	0.383039926	1.701874894	-0.016811457	1.872506441
947076	947364	7	+	unknown	no_ovlp2	SUTs	-0.534184923	0.197447029	0.21320368	-0.066166097	0.157638871
947364	948876	7	+	DIE2	ORF-T	ORF-T	-0.31123222	-0.266833247	-0.155039756	-0.057684084	-0.24602357
951404	951772	7	+	unknown	no_ovlp	CUTs	1.186019883	0.305096649	-0.152946658	-0.847631865	-0.108167473
951772	952460	7	+	BNS1	ORF-T	ORF-T	0.023031602	-0.552795116	0.438782109	0.450388778	1.332469531
953955	954699	7	+	NAS6	ORF-T	ORF-T	-0.608073979	-0.683741986	0.223546608	-0.237701895	0.481800193
958755	959883	7	+	unknown	no_ovlp	SUTs	0.716398013	-0.022140921	-0.026341615	-0.268061635	-0.888569579
959883	961403	7	+	YHB1	ORF-T	ORF-T	-0.117075237	-0.410707839	-0.251408792	-0.008939441	-0.338630727
968882	970618	7	+	SUT140	SUTs	SUTs	0.570890607	0.23035392	-0.272874924	-0.224251825	0.040832859
974266	974778	7	+	SUT141	SUTs	SUTs	0.793714086	-0.796516658	-0.527838716	-0.719060492	-0.908839713
977290	977930	7	+	FMP43	ORF-T	ORF-T	-0.22123014	-0.068501119	1.902834829	1.468588996	0.261775352
978346	978922	7	+	unknown	no_ovlp	CUTs	2.888429258	-0.13555864	-0.497903289	-0.648687878	-0.558556992
984850	985778	7	+	CPD1	ORF-T	ORF-T	0.074928008	-0.603851799	-0.087550579	0.158690142	0.14325362
985954	986794	7	+	SOL4	ORF-T	ORF-T	-0.1655128	0.081943168	2.100572313	1.723898112	-1.054277588
988002	989490	7	+	MGA1	ORF-T	ORF-T	-0.933736119	-0.248302396	0.62857801	1.191222217	-1.100016884
994338	995066	7	+	SUT142	SUTs	SUTs	1.213640958	-1.112341137	-0.047359443	0.066072121	0.098021665
995626	996826	7	+	unknown	no_ovlp2	SUTs	0.506356022	-0.442346749	-0.288315298	-0.092411732	0.080986533
996826	998402	7	+	GCN5	ORF-T	ORF-T	-0.246557591	-0.90643888	-0.44273918	-0.203717993	0.046409587
1000650	1000906	7	+	CUT166	CUTs	CUTs	2.513057944	0.109875313	0.101857181	-0.168127806	0.155976585
1000906	1002402	7	+	ENO1	ORF-T	ORF-T	-0.428120213	-0.139671564	-0.008012814	0.051077306	-0.245914342
1002522	1004226	7	+	unknown	no_ovlp	SUTs	-0.009829845	0.286236972	0.02398795	0.174387428	0.704730213
1004338	1006338	7	+	tT(UGU)G2, (	other	other	-1.330970727	0.216147097	3.124667173	0.808690452	-0.549777661
1011298	1012282	7	+	unknown	no_ovlp	SUTs	0.132026575	1.537485672	1.373371486	-0.481951192	-0.694007711
1012282	1012490	7	+	unknown	no_ovlp2	CUTs	4.454838267	6.599554587	2.123192875	-1.213994682	-5.157494853
1012490	1014218	7	+	TNA1	ORF-T	ORF-T	0.449952337	1.395327344	0.572605619	-0.185270942	-1.976499557
1016682	1018010	7	+	unknown	no_ovlp	SUTs	0.246564472	0.161883671	-0.214529537	0.087438142	0.348008431
1018010	1018298	7	+	CUT167	CUTs	CUTs	2.346890279	0.151047541	-0.622266813	-0.448884236	0.104332786
1022618	1024994	7	+	YGR266W	ORF-T	ORF-T	0.209128012	-0.1465112	0.284478818	0.208068361	0.608041388
1026154	1027266	7	+	unknown	no_ovlp	CUTs	1.913779396	-0.010258866	-0.736956736	-0.245521432	-0.162380717
1027266	1031618	7	+	YGR269W, Y1	other	other	0.066767321	-0.71066801	-0.347194931	-0.053343087	-0.248568049
1031786	1037834	7	+	SLH1	ORF-T	ORF-T	-0.018955125	-0.326627354	-0.288328637	0.104423174	-0.306076577
1038754	1039290	7	+	CUT168	CUTs	CUTs	2.653400852	0.521550564	0.037743776	0.217095963	-1.571581102
1043274	1043874	7	+	RTT102	ORF-T	ORF-T	0.3870149	-0.52209239	-0.430052733	-0.267194296	-0.225263285
1046738	1048570	7	+	CWC22	ORF-T	ORF-T	-0.031428073	-0.373776801	-0.318497378	0.022164263	-0.388736253
1052610	1057426	7	+	YOR1	ORF-T	ORF-T	-0.373115457	-0.525493223	-0.12735274	0.472719773	-0.027404745
1059058	1059746	7	+	SUT145	SUTs	SUTs	1.111365885	-0.778688365	-0.534715032	-0.307808405	-0.213881412
1060314	1060906	7	+	SUT146	SUTs	SUTs	2.219286679	0.015333511	-1.037891673	-0.192978571	-0.023046289
1070282	1072210	7	+	MAL13	ORF-T	ORF-T	0.596037902	-0.756092645	-0.152776686	0.667296172	-1.069609261
8173	11013	8	-	YHLComega1	other	other	0.507235527	-0.467517019	0.054411007	0.289876015	-0.59683363
14549	15837	8	-	SUT573	SUTs	SUTs	1.561574692	-0.132534064	-0.256170656	-0.323643179	-2.651703071
21061	21557	8	-	ARN1	ORF-T	ORF-T	0.186053582	-0.500387833	-0.071982969	-0.048065894	0.551193521
23517	25549	8	-	CBP2	ORF-T	ORF-T	0.025994861	-0.073526028	-0.077479911	-0.477366229	0.412511332
27989	32805	8	-	VMR1	ORF-T	ORF-T	-0.006226649	-0.206052822	0.335887008	0.405761054	-0.606412349
33045	34205	8	-	SBP1	ORF-T	ORF-T	-0.176799874	-0.446639207	-0.21803944	-0.036451231	-0.354475738
34749	36053	8	-	unknown	no_ovlp	SUTs	-0.002879063	-0.610159133	-0.601231165	-0.58633111	-0.283853562
36325	39517	8	-	GUT1	ORF-T	ORF-T	-0.236683067	-0.295576063	0.422220086	0.443341377	0.328156099
38621	39517	8	-	unknown	no_ovlp2	SUTs	-0.192527755	-0.512800256	0.082587141	-0.000146635	0.332183711
46341	47989	8	-	OCA5	ORF-T	ORF-T	0.050628293	-0.108793019	-0.061440006	-0.560461592	0.246466955
53125	54262	8	-	YHL026C	ORF-T	ORF-T	-0.072351243	-0.743960449	-0.742436751	-0.353474158	0.708331625
54262	55246	8	-	unknown	no_ovlp	CUTs	1.422253433	-0.284236709	-0.409256928	0.039323603	0.523711909
57502	58662	8	-	SUT574	SUTs	SUTs	1.215427726	0.2199235	-0.445256306	-0.948250348	-1.258537873
59110	62598	8	-	RMD11	ORF-T	ORF-T	0.079220003	-0.599470411	-0.19940057	-0.199986465	-0.029096957
64398	65910	8	-	FMP12	ORF-T	ORF-T	-0.024706946	-0.892437529	0.683429273	0.673624656	-0.205701682
66198	67534	8	-	OPI1	ORF-T	ORF-T	0.04236557	-0.386783046	-0.368319391	-0.17394331	-0.072543231
67646	70070	8	-	APM2	ORF-T	ORF-T	0.008771121	-0.121402028	0.191060614	-0.066619891	0.032349351
69582	70070	8	-	unknown	no_ovlp2	CUTs	3.172032854	0.112950065	-0.804364175	-0.24825028	-0.25780153
72006	75038	8	-	DUR3	ORF-T	ORF-T	0.3751764	-0.613668592	-0.391739174	-0.045653166	-0.487117614
76030	77350	8	-	YLF2	ORF-T	ORF-T	-0.686208597	-0.696636856	-0.219166521	-0.199280328	-0.281664092
77350	78366	8	-	OTU2	ORF-T	ORF-T	-0.15453145	-0.267711568	-0.289832013	-0.238533763	0.151293121
80438	81742	8	-	PRS3	ORF-T	ORF-T	0.085526339	-0.700425537	-0.29877801	-0.139301489	-0.208605568
81742	85182	8	-	YHL010C	ORF-T	ORF-T	-0.212622976	-0.504805709	0.159294039	-0.032855828	0.488015876
83918	85182	8	-	unknown	no_ovlp2	SUTs	-0.307067653	-1.010947086	-0.172637446	-0.073865996	0.05975753
92430	94998	8	-	YHL008C	ORF-T	ORF-T	-0.532402056	-0.970873012	0.074087968	0.723889396	0.182428289
94670	94998	8	-	unknown	no_ovlp2	SUTs	-2.229663033	-2.908644495	0.834601679	2.405284436	1.504485285
94998	98086	8	-	STE20	ORF-T	ORF-T	-0.180496518	-0.293752461	-0.353685014	-0.202050307	-0.018311872
98086	98846	8	-	SHU1, YHL0C	other	other	-0.319530075	-0.131369641	0.517352515	0.339618253	-0.167743474
100422	102038	8	-	LAG1	ORF-T	ORF-T	-0.7054647	-1.068329401	-0.643152972	-0.103179487	0.116606102
103190	103958	8	-	SUT576	SUTs	SUTs	1.187551877	-0.161454242	-1.523118856	0.122204963	0.473074927
109910	111326	8	-	YHR003C	ORF-T	ORF-T	1.238429803	-0.289504465	-0.455976901	-0.147287815	-0.468297094

111518	113198	8 -	NEM1	ORF-T	ORF-T	-0.288071664	-0.472362846	0.235124218	0.006835724	0.379721541
113390	115006	8 -	GPA1	ORF-T	ORF-T	0.184154332	-0.346881782	-0.566274339	-0.26997227	-0.403248189
115006	115382	8 -	unknown	no_ovlp	SUTs	0.148577992	0.082551217	-0.287749429	0.049883066	-0.134015946
115486	115934	8 -	MRS11	ORF-T	ORF-T	-0.567944823	-0.695355109	-0.32654087	-0.033568063	-0.28499863
116750	117486	8 -	unknown	no_ovlp	SUTs	0.547016733	0.204291124	0.770751805	0.64759447	0.363509682
119814	121822	8 -	ERG11	ORF-T	ORF-T	-0.352885919	-0.315993702	-0.730619027	0.009528914	-0.100157071
121822	122038	8 -	CUT639	CUTs	CUTs	1.880967388	-0.003381645	-0.0771921	-0.715240838	-0.395953364
122750	123662	8 -	YHR007C-A, :	other	other	-0.597217448	-0.044562799	1.072347791	0.327103109	0.700556164
123982	125918	8 -	YHR009C	ORF-T	ORF-T	-0.536624366	-0.35247423	0.024858511	0.002973275	0.259602304
128518	129318	8 -	CUT640	CUTs	CUTs	2.146645716	-0.234868732	-0.468060115	-0.299910252	-0.260194851
130614	131478	8 -	ARD1	ORF-T	ORF-T	-0.185741955	-0.493347129	-0.569390701	-0.076115784	0.228297111
132526	133326	8 -	unknown	no_ovlp	CUTs	1.856548546	0.018900683	-0.487190557	-0.362611553	-0.118607564
136686	138230	8 -	YSC84	ORF-T	ORF-T	-0.458906557	-0.255828604	0.684958624	0.440549594	0.214363518
139870	141446	8 -	ARG4	ORF-T	ORF-T	-0.443948988	-0.646270864	-0.68086916	-0.027927927	-0.187990299
141806	143614	8 -	DED81	ORF-T	ORF-T	-0.293586561	-0.278354739	-0.511941789	-0.192218946	-0.107044535
143614	144494	8 -	unknown	no_ovlp	SUTs	0.121384517	0.464568436	-0.107374373	-0.12616963	0.085025182
148110	148670	8 -	RPS27B	ORF-T	ORF-T	0.871688686	-1.317516929	-1.689054938	-1.132169079	-0.405384799
148734	149374	8 -	unknown	no_ovlp2	SUTs	-0.344546356	0.2570142	0.034464823	0.248053142	0.131865312
149374	151310	8 -	YHR022C	ORF-T	ORF-T	1.208332725	0.940214998	0.882254598	1.450854147	-1.140156491
150310	151310	8 -	unknown	no_ovlp2	SUTs	0.129357356	-0.650745095	-1.047440009	-1.08890417	0.217230118
157470	159206	8 -	MAS2	ORF-T	ORF-T	-0.137259079	-0.357794979	-0.629881704	-0.30631074	-0.505646185
161638	164766	8 -	RPN1	ORF-T	ORF-T	0.049233648	-0.200745838	0.054809588	-0.095651485	-0.111612574
164878	167470	8 -	DAP2	ORF-T	ORF-T	-0.392953098	-0.557980173	0.094157934	0.225169344	-0.299783486
167598	168566	8 -	YHI9	ORF-T	ORF-T	0.081916469	-0.696956007	-0.565970957	-0.431101093	-0.653202427
168734	170430	8 -	SLT2	ORF-T	ORF-T	0.354658415	-0.460688682	-0.35661572	-0.312311834	-0.237931355
170430	170670	8 -	unknown	no_ovlp2	SUTs	-0.105833565	-1.110131381	-0.810009027	-0.615800053	0.591290856
170670	172990	8 -	RRM3	ORF-T	ORF-T	-0.177713295	0.147060449	0.25512402	0.367771936	1.406012144
176926	178014	8 -	PIH1	ORF-T	ORF-T	-0.815252879	-0.406011566	0.461911426	0.227233669	0.793521641
178686	180038	8 -	unknown	no_ovlp	CUTs	1.554152226	0.317475775	-0.350911976	0.069730274	-0.251242496
180998	181702	8 -	unknown	no_ovlp	CUTs	1.505907868	0.638089455	-0.542519754	-0.36216732	0.900809315
184742	186910	8 -	MSC7	ORF-T	ORF-T	-0.393272978	-0.46440154	-0.627961476	0.191642355	-0.342411325
187054	187510	8 -	VMA10	ORF-T	ORF-T	-0.076767162	-0.307140623	0.103134496	0.071054258	0.092214814
188942	189750	8 -	SRB2	ORF-T	ORF-T	0.135567915	-0.365007985	-0.195654577	-0.006707647	-0.354528456
189750	190294	8 -	CUT642	CUTs	CUTs	1.554678078	-0.049871097	0.004670291	0.261489277	-0.131960398
192718	193638	8 -	DOG2	ORF-T	ORF-T	-0.162505826	0.231877574	0.625177326	0.211368041	-0.310880558
193966	195198	8 -	DOG1	ORF-T	ORF-T	0.629635978	-0.531075007	-0.215503911	0.120545042	-0.906034053
197230	198350	8 -	INM1	ORF-T	ORF-T	0.495421368	-0.250332693	-0.683554145	-0.075517329	-0.611007863
198590	201358	8 -	AAP1	ORF-T	ORF-T	-0.315488963	-0.527016477	-0.096590846	-0.277613546	-0.253293993
206022	207238	8 -	YHR049C-A	other	other	0.080596712	-0.435260553	0.519756164	0.395693315	0.742589206
208846	209286	8 -	CUT643	CUTs	CUTs	2.697703285	0.600808186	-0.213196438	0.217803133	0.169651714
209286	210374	8 -	unknown	no_ovlp	SUTs	0.526849363	0.194826588	-0.62425646	-0.090178339	-0.476197583
214246	216236	8 -	unknown	no_ovlp2	CUTs	1.860677153	-0.099900186	-0.10410965	-1.02873825	-0.728593522
216236	217885	8 -	RSC30	ORF-T	ORF-T	-0.170179384	-0.581313996	-0.466273473	-0.127944221	-0.243886123
218181	218837	8 -	CPR2	ORF-T	ORF-T	0.327633467	-0.183545295	-0.495132878	0.136646488	0.752659531
218837	219901	8 -	MED6	ORF-T	ORF-T	0.180753272	-0.100441679	-0.285833961	-0.074168909	0.013503223
221349	222517	8 -	GIC1	ORF-T	ORF-T	-0.290790936	-0.747035999	-0.624259159	-0.494749643	0.106109447
222781	223781	8 -	RPP1	ORF-T	ORF-T	0.524032337	0.350260894	-0.321151919	0.237069496	-0.152504469
223957	225205	8 -	PAN5	ORF-T	ORF-T	0.103769966	-0.543381342	0.015259912	0.089482099	0.06416959
225453	227189	8 -	SSZ1	ORF-T	ORF-T	-0.168733491	-0.074896864	-0.632063465	-0.13500047	-0.114364333
227429	229085	8 -	RRP3	ORF-T	ORF-T	0.569828657	0.10341639	-0.049671935	-0.013463543	0.175015039
230285	230757	8 -	unknown	no_ovlp	CUTs	2.770780091	-0.250795915	-1.681377039	-0.893831986	0.13914058
231245	231853	8 -	CUT644	CUTs	CUTs	3.607347506	0.326375819	-0.694331269	-0.181316155	0.254909967
233421	234685	8 -	RRP4	ORF-T	ORF-T	0.542917001	-0.151590571	-0.015156978	-0.179619283	0.611967099
234685	234901	8 -	unknown	no_ovlp2	CUTs	1.453408993	0.471017404	-0.991096198	0.213012826	0.902676591
234901	236045	8 -	YHR069C-A	other	other	1.627377928	0.109267856	-0.900500659	0.157852172	1.035750897
238205	238845	8 -	CUT645	CUTs	CUTs	2.781966056	0.222827411	-0.217174051	-0.09876009	-0.348412286
241853	242317	8 -	SUT580	SUTs	SUTs	2.144213304	-0.067087073	0.650004214	-0.016242109	-0.029838597
248445	249685	8 -	PPE1	ORF-T	ORF-T	-0.000419981	0.155864521	0.850913799	0.619798608	0.436333593
252277	255621	8 -	NMD2	ORF-T	ORF-T	0.157859926	-0.333045161	-0.488930761	-0.100466065	-0.145959239
255621	256333	8 -	SUT582	SUTs	SUTs	0.136280108	0.592550586	0.850180071	-0.006063028	0.376413062
258037	261845	8 -	IRE1	ORF-T	ORF-T	-0.084764885	-0.304702399	-0.424012385	-0.100275846	-0.151372857
266957	267221	8 -	YHR080C	ORF-T	ORF-T	-0.775603384	0.037606374	-0.003743194	0.695902991	0.39473275
270285	272413	8 -	KSP1	ORF-T	ORF-T	-0.115386308	-0.944697132	-0.096161188	-0.15674602	-0.654265328
272413	273405	8 -	unknown	no_ovlp	CUTs	2.129849118	0.407571336	-1.170517405	-0.51352086	-0.28695748
273405	274493	8 -	unknown	no_ovlp	SUTs	0.665839564	0.287122549	-0.584080962	-0.227106958	0.584054245
274493	276485	8 -	unknown	no_ovlp	SUTs	-0.080239703	0.376070473	0.001459851	-0.023763305	0.358147666
279549	281245	8 -	SUT583	SUTs	SUTs	1.873199451	0.372014984	-0.413964436	-0.038143434	-0.862374378
282517	283365	8 -	GAR1	ORF-T	ORF-T	-0.218200229	-0.645057471	-0.538229267	0.015206249	-0.140327921
283541	284621	8 -	YNG2	ORF-T	ORF-T	-0.097282475	-0.276407724	0.446114065	-0.147584978	0.268454672
284845	286789	8 -	MSR1	ORF-T	ORF-T	-0.130322307	-0.358493229	-0.778879756	-0.583790603	-0.611429706
286957	288877	8 -	HXT4	ORF-T	ORF-T	0.184036417	-0.925003237	0.090619885	0.425979919	-1.119752316
288877	289637	8 -	unknown	no_ovlp	CUTs	1.027224994	-0.284611704	-0.168431026	-0.100441256	-2.290842169
292661	292925	8 -	HXT1	ORF-T	ORF-T	1.464763617	0.214738797	-0.774592892	-0.641790202	-0.811500993
292925	293221	8 -	unknown	no_ovlp	SUTs	0.352966089	-0.626477692	0.132811375	-1.095303346	-1.382430432
293221	296141	8 -	unknown	no_ovlp	SUTs	-0.156878567	0.378424464	2.19864104	1.041280619	0.111578289
297277	298365	8 -	YHR097C	ORF-T	ORF-T	-0.048554063	-0.242225246	0.548652084	0.541947391	-0.350830724
299021	302021	8 -	SFB3	ORF-T	ORF-T	-0.457300574	-0.228958517	-0.392036863	-0.163772252	-0.090511346
314117	314717	8 -	YHR100C	ORF-T	ORF-T	0.387682054	0.221863571	0.050350054	0.083768678	0.508964111
314717	316229	8 -	BIG1	ORF-T	ORF-T	-0.126195083	-1.010938191	-0.120287206	-0.11795362	-0.127362952
315997	316229	8 -	unknown	no_ovlp2	CUTs	2.448917526	1.160084621	-0.228653008	-0.360083774	0.300452367
319365	319957	8 -	CUT649	CUTs	CUTs	0.579164048	-0.569296204	-0.046337618	0.349754707	1.037359219
324669	325429	8 -	unknown	no_ovlp	CUTs	1.443582419	0.22118655	-0.591791432	-0.222516576	0.616115518

326661	328101	8	-	CDC12	ORF-T	ORF-T	-0.05118699	-0.415338357	-0.142318226	-0.233467391	0.180421491
335701	336165	8	-	YHR112C	ORF-T	ORF-T	0.634300605	-0.980096017	0.180105534	-0.14885966	-0.087384855
340021	341541	8	-	DMA1	ORF-T	ORF-T	-0.548911222	-0.846282325	0.036560795	0.145442079	0.290786949
341541	342229	8	-	unknown	no_ovlp	SUTs	0.341144547	-0.28605764	-0.080928217	-0.039192151	0.882289039
344261	345645	8	-	ORC6	ORF-T	ORF-T	-0.206572857	-0.895756064	0.536797258	0.057101959	0.49111549
347869	349629	8	-	unknown	no_ovlp	SUTs	0.355972217	-0.133733239	-0.876783121	0.188195239	1.402003752
353061	353365	8	-	unknown	no_ovlp	CUTs	3.520030486	-0.329337788	-1.267698499	-0.555695328	0.178630634
354085	354613	8	-	SUT586	SUTs	SUTs	1.912204409	-0.101675429	-0.436488764	0.009470648	-0.252448314
356493	358197	8	-	SUT587	SUTs	SUTs	1.08493248	0.070267964	-0.452548932	-0.371291436	-2.245840324
360573	361701	8	-	unknown	no_ovlp	CUTs	2.251538873	0.35947871	-1.778786207	-0.653770853	0.326730456
362909	364221	8	-	ARP1	ORF-T	ORF-T	-0.398026858	-0.305651627	0.05995532	-0.070806589	0.562263749
365037	365477	8	-	unknown	no_ovlp2	SUTs	-0.445765826	-0.913918932	-0.000671736	0.240991875	-0.194092301
365477	368134	8	-	YHR131C	ORF-T	ORF-T	0.039512146	-0.594863238	-0.3613148	-0.118406666	-0.672351436
368134	368350	8	-	unknown	no_ovlp	SUTs	0.241389071	0.178475471	0.249558144	-0.220306056	0.70115153
368454	369822	8	-	ECM14	ORF-T	ORF-T	-0.085240725	-0.815734269	-0.324841224	0.109758622	-0.240566281
370526	371638	8	-	NSG1	ORF-T	ORF-T	0.213810271	-0.520629332	-1.011667805	-0.398198567	-0.400435699
372606	374430	8	-	YCK1	ORF-T	ORF-T	-0.615038189	-0.386693787	0.084777087	0.046108032	0.122868829
377230	377766	8	-	YHR138C	ORF-T	ORF-T	-0.025937944	-0.403198283	1.078355431	0.411168442	1.112265737
377766	378126	8	-	unknown	no_ovlp2	SUTs	-0.52621263	0.405491387	0.684516367	0.30892105	0.294855816
378126	381006	8	-	SPS100	ORF-T	ORF-T	-0.53506854	-0.229561451	0.553193817	0.275375391	1.075643718
379886	381006	8	-	unknown	no_ovlp2	SUTs	-0.131244487	0.381872598	0.197578551	0.018724324	0.087000325
381006	381286	8	-	CUT650	CUTs	CUTs	2.480686446	0.404952858	0.274621666	0.490482595	-0.490985763
382310	382814	8	-	RPL42B	ORF-T	ORF-T	0.982512356	-0.537031279	-0.716738877	-1.214061892	-0.217200006
383542	384670	8	-	SUT588	SUTs	SUTs	1.160627319	-0.890175814	-1.266886827	-0.554447454	0.547601044
386742	386958	8	-	SUT590	SUTs	SUTs	1.069219224	-1.461985278	-0.020708619	-0.045366531	0.047047926
387766	388758	8	-	DCD1	ORF-T	ORF-T	0.023220183	-0.665713082	-0.608542207	-0.672611689	0.215005909
392550	393318	8	-	MRPL6	ORF-T	ORF-T	-0.964184575	-1.012540833	0.048171572	-0.025830949	-0.181955192
394350	396790	8	-	SKG6	ORF-T	ORF-T	-0.055774199	-0.233683907	-0.524296418	-0.165025857	0.482389609
399222	401022	8	-	YHR151C	ORF-T	ORF-T	-0.289632071	-0.500505797	-0.159181902	-0.254794983	0.578199988
406190	406798	8	-	SUT591	SUTs	SUTs	1.726236005	1.050024937	0.767412558	0.223736638	-1.558137477
410406	410886	8	-	unknown	no_ovlp2	CUTs	1.566297704	0.501557787	0.732653719	-0.550297474	1.375755711
410886	413654	8	-	LIN1	ORF-T	ORF-T	-0.285221567	0.441089242	0.983141983	0.452191834	0.612689649
412446	413654	8	-	unknown	no_ovlp2	SUTs	-0.098484274	0.228440213	-0.11058213	-0.055029813	-0.057491125
415854	417318	8	-	KEL1	ORF-T	ORF-T	0.04743637	-0.245725143	-0.296151836	-0.022340795	-0.14436458
417318	419198	8	-	unknown	no_ovlp2	SUTs	-0.145839872	0.234701475	-0.036518738	0.037333171	0.179120646
419198	420270	8	-	PEX18	ORF-T	ORF-T	-0.045792324	0.968125526	1.988696556	1.086452703	0.070394655
422118	422526	8	-	YAP1801	ORF-T	ORF-T	-0.395712035	-1.304944888	0.099064708	0.40320626	-0.185461352
424454	429238	8	-	DNA2	ORF-T	ORF-T	-0.197036832	-0.422998151	-0.138153125	-0.258848034	-0.15449781
432318	437038	8	-	PRP8	ORF-T	ORF-T	0.070456303	-0.26899058	-0.556316948	0.252858672	-0.449486846
437038	439134	8	-	CDC23	ORF-T	ORF-T	0.020772922	-0.563846948	-0.363602694	-0.26019881	-0.069681765
441622	441942	8	-	CUT653	CUTs	CUTs	3.86592784	0.475182336	-1.145212446	-0.273943714	0.617894398
450262	450678	8	-	CUT655	CUTs	CUTs	2.861322955	-0.565695754	-0.517005118	0.051714074	-0.295083743
450678	452646	8	-	unknown	no_ovlp	SUTs	0.358561537	0.163537286	0.13531616	-0.104144346	-0.045933126
457360	458328	8	-	SUT592	SUTs	SUTs	-0.394663441	-0.654383202	-1.077398103	-1.413366551	-1.029764196
459000	462040	8	-	CUT656	CUTs	CUTs	2.394309368	0.281927783	-1.663832237	-0.316945782	-0.691673431
464192	464784	8	-	YHRCdelta12	other	other	0.769252814	-0.44450333	0.107117967	-0.345408621	-1.652762146
469760	470584	8	-	unknown	no_ovlp	CUTs	2.097342411	-0.146723826	-0.459375949	-0.172221221	1.144493695
476088	480736	8	-	KOG1	ORF-T	ORF-T	-0.259381283	-0.325290456	-0.317014996	-0.005430904	-0.23078215
481928	483848	8	-	GPI16	ORF-T	ORF-T	-0.160342898	0.025563827	-0.287226486	-0.078125509	0.084581304
483848	484592	8	-	SUT595	SUTs	SUTs	0.515021593	-0.223610122	0.209784187	0.006843384	0.696758057
486168	486640	8	-	CTF8	ORF-T	ORF-T	0.025568623	-0.429217118	-0.473022851	-0.475240202	0.103931155
487656	488288	8	-	EGD2	ORF-T	ORF-T	-0.212204493	-0.356346283	-0.577798329	-0.36660128	-0.27943121
488288	491648	8	-	SUT596	SUTs	SUTs	0.387658558	0.126675316	-0.232072153	-0.350185989	0.292788863
496200	497280	8	-	FMP22	ORF-T	ORF-T	-0.537287556	-1.177999065	0.205596934	-0.045052398	0.165857798
498456	498880	8	-	FMP34, YHR:	ORF-T	ORF-T	0.487603999	-0.272951005	-0.373787638	-0.42717587	-0.990357741
499872	501168	8	-	PPX1	ORF-T	ORF-T	0.156936073	-0.495614379	-0.653999695	-0.311998628	-0.308191009
505248	505528	8	-	RPS4B	ORF-T	ORF-T	1.135050205	0.003001486	-1.545983934	-1.406387595	-0.245405781
507728	508688	8	-	SUT597	SUTs	SUTs	1.551619695	0.557809523	0.336057224	0.616171296	1.522435801
514864	517264	8	-	SET5	ORF-T	ORF-T	-0.201921328	-0.549091227	-0.104766347	-0.079064885	-0.12947507
516568	517264	8	-	unknown	no_ovlp2	CUTs	1.244845308	-0.697855178	-0.342913532	-0.196457266	-0.361491447
518832	520528	8	-	unknown	no_ovlp2	SUTs	0.931281038	-0.19526511	-1.39613313	-0.055636968	-1.044446831
520528	521872	8	-	YHR210C	ORF-T	ORF-T	0.236304016	-0.23864202	0.247394303	0.278975924	-0.298429745
548712	549632	8	-	unknown	no_ovlp	SUTs	-0.367025625	-0.318138798	0.129671067	-0.007906487	-2.594487812
6465	7689	8	+	unknown	no_ovlp	SUTs	0.272602703	-0.103448611	0.044292644	-0.671834772	-0.778877854
11225	11537	8	+	SUT147	SUTs	SUTs	-0.647014705	-1.585525536	1.058909565	1.044492669	0.425986882
13553	14585	8	+	YHL044W	ORF-T	ORF-T	1.71868877	1.474843304	1.261418368	1.49280171	-2.646175469
15889	16729	8	+	YHL042W	ORF-T	ORF-T	0.927279068	0.012474465	-0.004961727	0.132035081	-1.850712703
21737	23033	8	+	YHL039W	ORF-T	ORF-T	0.689557766	0.295666971	-0.686806365	-0.137500019	0.167207526
26201	27953	8	+	MUP3	ORF-T	ORF-T	0.755294149	0.396218819	-0.099420471	-0.601045148	0.097963746
38849	40065	8	+	YHL030W-A	other	other	1.392939211	0.12616222	0.517159377	0.541081192	1.041897437
39529	40065	8	+	unknown	no_ovlp2	SUTs	0.890839871	0.49501268	0.547297294	0.478278841	1.706888288
40065	41633	8	+	ECM29	ORF-T	ORF-T	0.095564242	-0.024646421	0.033029261	-0.151634838	1.067301134
47465	48849	8	+	unknown	no_ovlp	SUTs	0.036643225	0.340631614	0.035342229	-0.156598476	0.153234426
48849	50665	8	+	WSC4	ORF-T	ORF-T	-0.848600587	-0.084685426	0.203167185	0.178444571	0.825174326
50993	53193	8	+	RIM101	ORF-T	ORF-T	-0.076147741	-0.479888265	-0.178236741	0.024604403	-0.204834349
54826	55946	8	+	SNF6	ORF-T	ORF-T	-0.200044922	-0.620783887	-0.176482244	-0.337457466	-0.098669383
57826	60146	8	+	SUT148	SUTs	SUTs	-0.392190377	-0.29072295	1.361454508	0.801316903	2.450894387
65786	67394	8	+	unknown	no_ovlp	CUTs	1.091160519	-0.00826775	0.728276279	0.861545197	0.273406658
67754	68986	8	+	unknown	no_ovlp	CUTs	2.78748658	0.098174574	-1.495088576	-0.320844937	0.319590653
69706	70282	8	+	YHL018W, YH	ORF-T	ORF-T	0.247841435	-0.029640087	0.36075311	-0.540063961	0.078697912
74826	75394	8	+	CUT171	CUTs	CUTs	1.269948858	0.199315767	-0.000240583	-0.384100381	-0.088302206
75394	76106	8	+	RPS20	ORF-T	ORF-T	-0.3092987	-0.324083602	-0.083558372	-0.116412396	-0.090475445

83682	95010	8	+	unknown	no_ovlp	SUTs	-0.178769466	0.306240927	0.800097954	0.256082122	0.322192727
96338	99146	8	+	SUT151	SUTs	SUTs	-0.152110345	0.22911317	0.002729473	0.081204429	0.456889263
99146	100530	8	+	MRP4	ORF-T	ORF-T	-1.067669927	-0.712211087	-0.370512141	-0.073631272	-0.052825462
102226	104058	8	+	HSE1	ORF-T	ORF-T	-0.219739049	-0.567523662	0.234729758	-0.206123317	0.011137514
104402	104938	8	+	RPL14B	ORF-T	ORF-T	0.395124927	-0.510965512	-0.985402772	-0.977763962	-0.267875262
106050	107506	8	+	OSH7	ORF-T	ORF-T	0.051305349	-0.239171216	0.233879586	0.018922914	0.002110318
107898	108354	8	+	QCR10	ORF-T	ORF-T	-0.0692608	-0.241960904	0.588117335	-0.182167576	0.163930735
108674	110002	8	+	LEU5	ORF-T	ORF-T	-0.196777921	-0.528747336	0.392625587	-0.015209744	0.153525641
113434	114050	8	+	unknown	no_ovlp	CUTs	1.825631229	0.304472467	-0.333925927	0.210276998	0.795597732
115506	115882	8	+	CUT173	CUTs	CUTs	3.029956738	0.52357659	-0.593406139	-0.267013101	-0.147019924
117786	119562	8	+	STP2	ORF-T	ORF-T	0.344173195	-0.275235614	-0.258684635	-0.08407583	-0.152035565
122162	122810	8	+	SUT153	SUTs	SUTs	0.475136914	0.308760962	0.201349566	0.137118941	-0.281664507
122810	123274	8	+	SUT153	SUTs	SUTs	2.582338625	-0.152400988	-0.943329482	-0.144005195	-0.018874452
126546	127114	8	+	RPL27A	ORF-T	ORF-T	1.351725337	-0.363435655	-1.107827905	-1.645000592	-0.496645263
127634	129674	8	+	DIA4	ORF-T	ORF-T	-0.448276282	-0.417301224	0.245579664	-0.243756348	0.633087242
129098	129674	8	+	unknown	no_ovlp2	SUTs	-0.146888657	-0.476209255	-0.877269526	-0.929969351	0.284567045
129674	130698	8	+	VPS29	ORF-T	ORF-T	0.067083343	-0.65533703	-0.109822421	-0.257656127	-0.046748107
130698	135226	8	+	unknown	no_ovlp2	SUTs	-0.200665086	0.363885867	0.400356323	-0.012526556	0.266758074
135226	136498	8	+	MIP6	ORF-T	ORF-T	-0.305720205	0.9939905	1.020554932	-0.523478668	-0.582401541
136498	138690	8	+	unknown	no_ovlp	CUTs	2.144496575	0.930641117	-0.958752025	-1.117861064	-1.928297549
138690	139954	8	+	YSC83	ORF-T	ORF-T	-0.438190529	-0.919899978	0.328387153	0.128450056	0.133830245
141650	142370	8	+	unknown	no_ovlp	CUTs	1.635778176	0.474532086	-1.236230176	-0.05645239	-0.69762034
143922	146146	8	+	YHR020W	ORF-T	ORF-T	0.207925483	-0.315114514	-0.782229547	-0.325937438	-0.419759126
146146	147778	8	+	YHRWtau3	other	other	-0.288759156	-0.399998386	0.619273132	-0.009697459	-1.146149657
151578	155178	8	+	MYO1	ORF-T	ORF-T	0.059721415	-0.045083859	0.006607333	-0.168687223	0.737552178
159394	160594	8	+	THR1	ORF-T	ORF-T	0.158710733	0.151370012	-0.661038998	-0.223618573	-0.263703342
160730	161706	8	+	PPA1	ORF-T	ORF-T	-0.028256076	-0.335143438	-0.223251948	0.033672467	-0.032581419
164978	166778	8	+	SUT155	SUTs	SUTs	1.370630857	0.251490953	-1.119039431	-0.167752361	0.998761592
167682	168386	8	+	YHR028W-A	other	other	2.406229986	-0.460429522	-0.836285471	0.129634248	0.20610685
170714	171354	8	+	CUT175	CUTs	CUTs	4.358538172	0.126617804	-0.980553266	-0.036458202	-0.224951602
173250	175306	8	+	YHR032W, YI	other	other	0.049561399	-0.162005456	-0.990370565	-0.159718096	0.019121524
175306	176930	8	+	YHR033W	ORF-T	ORF-T	0.070083875	0.171424625	0.387299932	0.940799989	1.919342325
178170	180234	8	+	YHR035W	ORF-T	ORF-T	0.170713641	-0.227996735	-0.386132887	-0.324002158	0.293826318
180234	181938	8	+	BRL1	ORF-T	ORF-T	0.156717907	-0.54331434	-0.419582109	-0.146996919	0.027585723
181938	183866	8	+	PUT2	ORF-T	ORF-T	-0.037498856	-0.537991884	-0.138227098	0.13333104	0.163155563
184066	184794	8	+	RRF1	ORF-T	ORF-T	-0.430429751	-0.801250965	0.285333382	-0.002549765	-0.472589386
187154	187594	8	+	unknown	no_ovlp	CUTs	2.663420533	0.277933347	-1.205891439	-0.123535877	-0.625711721
187922	189114	8	+	BCD1	ORF-T	ORF-T	0.097934531	0.075437651	-0.097821098	-0.111045508	0.868027517
190498	192794	8	+	NCP1	ORF-T	ORF-T	-0.682934038	-0.476307101	-0.387151989	0.306026908	0.21292235
195490	197330	8	+	YHR045W	ORF-T	ORF-T	-0.456817251	-0.712028959	-0.690404522	-0.048794068	-0.489286985
198610	199130	8	+	CUT178	CUTs	CUTs	3.00364039	0.369775546	-0.558440038	0.561374157	0.063345389
201642	204538	8	+	unknown	no_ovlp2	SUTs	0.845225179	0.247913292	-0.479958374	0.053152103	-0.522336378
204538	206418	8	+	YHR048W	ORF-T	ORF-T	-1.525183408	-1.377932533	0.412582898	1.119561267	-0.614468509
206418	207322	8	+	FSH1	ORF-T	ORF-T	0.548768539	-0.371857489	-0.639121773	0.187222765	-0.624585478
207506	209346	8	+	SMF2	ORF-T	ORF-T	-0.345755524	-0.422725189	0.082313145	0.168583646	0.349075482
209546	210218	8	+	YHR050W-A,	other	other	-0.445906201	-0.428784265	0.515876484	0.009019165	-0.188504596
210818	212106	8	+	CIC1	ORF-T	ORF-T	0.355115655	-0.372409531	-0.83537924	0.007783216	-0.017213995
217482	218993	8	+	unknown	no_ovlp	SUTs	-0.544996004	-1.228114499	-0.55582431	-0.189378736	0.804133538
218993	219537	8	+	CUT179	CUTs	CUTs	2.572005861	0.189312507	-0.441037877	0.262977652	0.280718561
220065	220537	8	+	FYV4	ORF-T	ORF-T	-0.83797653	-0.667297519	0.290908219	-0.184458589	-0.097696738
220745	221297	8	+	VMA22	ORF-T	ORF-T	0.326166508	-0.268872513	-0.225379939	-0.574447778	-0.479580828
221729	224513	8	+	SUT159	SUTs	SUTs	0.817905367	-0.277801087	-0.882037373	-0.190581007	0.397499563
229313	230793	8	+	SSF1	ORF-T	ORF-T	0.661301734	0.051658342	-0.732060332	-0.20280017	0.491354713
230953	232097	8	+	HTD2	ORF-T	ORF-T	-0.28079084	-0.43148858	-0.042692949	-0.069220047	0.163318932
232097	233337	8	+	DYS1	ORF-T	ORF-T	0.262010094	-0.37781204	-0.821425324	-0.209292715	-0.316152239
234889	236657	8	+	TRM5	ORF-T	ORF-T	0.065092564	-0.076704986	-0.196259932	-0.05741609	0.47838515
236657	237761	8	+	PCL5	ORF-T	ORF-T	-0.550689879	-0.724896642	-0.080084701	0.034064632	-0.544822352
239041	241393	8	+	ERG7	ORF-T	ORF-T	0.439200718	0.215869492	-0.561705108	-0.17943496	-0.687190436
241601	242001	8	+	NOP10	ORF-T	ORF-T	-0.274552804	-0.805570648	-0.023874381	-0.007983137	0.201569516
242497	245737	8	+	OSH3, YHR07	ORF-T	ORF-T	-0.034713012	-0.440878514	-0.253206636	0.102181716	-0.144733453
246161	248489	8	+	QNS1	ORF-T	ORF-T	-0.034139313	-0.474037182	-0.481826627	-0.065353872	-0.667862299
251089	252337	8	+	PTC7	ORF-T	ORF-T	-0.004134128	0.00883817	-0.13386056	-0.206415565	-0.490806024
256305	258161	8	+	YHR078W	ORF-T	ORF-T	0.638211126	0.020355922	-0.36861244	-0.195567862	-0.310981262
266497	267521	8	+	SUT160	SUTs	SUTs	0.919024247	0.473613412	0.294483012	-0.241660015	-0.051150191
267521	268153	8	+	LRP1	ORF-T	ORF-T	0.021748693	-0.859806918	-0.366874218	-0.021998615	0.575587914
272153	272633	8	+	SUT161	SUTs	SUTs	-0.171364456	0.034717965	0.667573264	-0.110202835	-0.18261484
272633	273705	8	+	SAM35	ORF-T	ORF-T	-1.00187745	-0.268153129	0.240360277	-0.120579078	0.488873259
273881	276545	8	+	STE12	ORF-T	ORF-T	-0.102607454	-0.486665468	-0.975073555	-0.354545253	-0.416010476
276785	277833	8	+	IPI1	ORF-T	ORF-T	0.620210932	-0.27005382	-1.122715658	-0.58814769	0.347970701
277945	279953	8	+	NAM8	ORF-T	ORF-T	-0.324010275	-0.224941778	0.02358045	-0.04576003	0.51140228
280761	281353	8	+	YHR087W	ORF-T	ORF-T	-1.020335849	-0.859684932	1.888371785	0.931735644	-0.019447638
281473	282561	8	+	RPF1	ORF-T	ORF-T	0.441509028	-0.476013344	-0.34260036	0.117798993	0.297366873
284889	285361	8	+	unknown	no_ovlp	CUTs	2.08922807	0.701583934	-0.238934345	0.237551676	0.863815074
289609	290593	8	+	unknown	no_ovlp	CUTs	1.884438883	0.561805771	0.100935874	0.012528949	-0.547428894
293193	293937	8	+	unknown	no_ovlp	CUTs	2.246194966	0.519653631	-1.225753246	-1.036280103	-2.068044217
293937	294561	8	+	SUT164	SUTs	SUTs	2.612067352	-0.832730049	-0.615636925	-0.698478727	-1.584186974
302721	310241	8	+	TRA1	ORF-T	ORF-T	-0.345720823	-0.597155419	-0.390405884	0.181496225	-0.0204912
316401	319809	8	+	KIC1	ORF-T	ORF-T	-0.005687031	-0.131047159	-0.347082835	0.068451466	0.033247585
320209	323145	8	+	SBE22	ORF-T	ORF-T	-0.027404886	-0.985830921	-0.635301778	0.15108455	0.237237974
323337	324497	8	+	GRE3	ORF-T	ORF-T	-0.226601222	-0.434960621	1.023486156	0.643683713	0.259338496
324497	325617	8	+	YPT35, TRR2	ORF-T	ORF-T	-0.338191051	-0.61039013	0.357752818	-0.051705962	0.177459265
328281	330177	8	+	GGA2	ORF-T	ORF-T	-0.240518154	-0.208770725	-0.177644664	-0.128477428	0.153372478

330177	331209	8	+	CTM1	ORF-T	ORF-T	-0.415335283	-0.421525016	-0.323433509	-0.290430609	-0.870476132
332265	333057	8	+	unknown	no_ovlp2	SUTs	-0.93889853	-0.746969688	0.568393301	0.016101138	0.701386083
333057	334401	8	+	UBA4	ORF-T	ORF-T	0.164400568	0.024693796	-0.220511101	0.041209312	0.012101077
336337	337953	8	+	YHR113W	ORF-T	ORF-T	-0.051967879	-0.072355167	-0.013285195	-0.08276854	0.508167213
337953	338793	8	+	BZZ1	ORF-T	ORF-T	-0.196145613	-0.60639408	-0.092134787	0.06543618	-0.078066319
341729	342353	8	+	COX23, TOM	ORF-T	ORF-T	-1.131710491	-0.754898224	-0.004111269	-0.618243104	0.659888783
345825	349433	8	+	SET1	ORF-T	ORF-T	-0.174007261	-3.584878763	-3.913912116	-0.158724304	0.374482841
349433	352513	8	+	MSH1	ORF-T	ORF-T	0.461464346	0.25302325	-0.361882334	-0.267607983	0.147330929
352689	353417	8	+	LSM12	ORF-T	ORF-T	-0.851929866	-1.023189241	-0.05006159	-0.093540973	0.323840737
353593	354505	8	+	YHR122W	ORF-T	ORF-T	-0.307741365	-0.259465016	-0.212503262	-0.345094453	0.518770769
354969	356233	8	+	EPT1	ORF-T	ORF-T	-0.442664483	-0.44295866	-1.137911131	-0.392119614	0.007542789
358113	358617	8	+	unknown	no_ovlp	SUTs	0.329406116	0.533861535	0.530289776	-0.142286798	-1.143828686
360881	361857	8	+	YHR127W	ORF-T	ORF-T	-0.561328536	-0.335808139	0.244472637	-0.206612027	0.989113943
362033	363001	8	+	FUR1	ORF-T	ORF-T	-0.003874801	-1.161901609	-1.39227075	-0.296428196	-0.45430814
364425	365073	8	+	SUT167	SUTs	SUTs	1.57715528	-0.696584245	-0.399967572	0.155221119	-0.320975084
368138	370034	8	+	unknown	no_ovlp	SUTs	0.139512916	0.297403623	0.107015244	0.253002506	0.786213309
370034	370538	8	+	IGO2	ORF-T	ORF-T	-0.422694283	-0.654426665	0.073021436	0.114392884	0.173478221
371794	372674	8	+	WSS1	ORF-T	ORF-T	-0.144892202	-0.699102932	-0.250320356	-0.158065611	-0.125299312
374234	375690	8	+	unknown	no_ovlp	SUTs	-0.330116781	0.383018835	0.38271255	0.260922766	0.833091272
375690	377394	8	+	ARO9	ORF-T	ORF-T	0.058279222	-0.961527112	-0.221387011	-0.050839515	0.584638667
378034	381554	8	+	SUT169	SUTs	SUTs	0.724821043	0.615013237	0.481435017	0.470956527	0.292226673
381554	381858	8	+	SNR32	other	other	4.121057515	1.928996522	-0.605686883	-0.27441499	0.059760938
383554	384570	8	+	CHS7	ORF-T	ORF-T	-0.553085815	-0.069200635	0.506379672	-0.046214446	0.273135782
385258	386730	8	+	DSE2	ORF-T	ORF-T	-0.067459147	-0.110955696	-0.434259145	-0.179470801	0.198703107
387194	387730	8	+	RPC10	ORF-T	ORF-T	-0.40089193	-0.581752646	-0.139424119	-0.198118331	0.174895212
390282	391930	8	+	CRP1	ORF-T	ORF-T	0.19377135	-0.124495492	-0.162425153	0.277586178	0.31063167
393514	394418	8	+	IMP3	ORF-T	ORF-T	0.579734138	-0.340636266	-0.638678303	0.036577682	0.37480764
397218	399090	8	+	PEX28	ORF-T	ORF-T	-0.231801172	-0.107718586	0.252994748	0.021191915	0.649229661
401354	402098	8	+	SPO12	ORF-T	ORF-T	-0.300762896	0.07891943	0.699086714	0.264874736	0.805799532
402938	406258	8	+	RTT107	ORF-T	ORF-T	-0.397479157	-0.395660793	-0.625135321	-0.004473115	0.287496597
407018	410994	8	+	YSP1	ORF-T	ORF-T	0.080254701	-0.242176689	-0.389482827	0.190145988	-0.309888858
411242	411594	8	+	SNR71	other	other	4.318025993	-1.445739142	-0.394427285	-0.365334347	-0.262450378
417490	420026	8	+	YHR159W	ORF-T	ORF-T	-0.63795063	-0.296228675	0.363792801	0.054005503	0.718609333
422698	422906	8	+	YHR162W	ORF-T	ORF-T	0.366403494	0.492016817	0.239950418	-0.118332571	-1.160371218
423730	424626	8	+	SOL3	ORF-T	ORF-T	-0.14850173	-0.069626524	0.057201599	-0.081024792	0.639280123
436746	437274	8	+	unknown	no_ovlp	SUTs	0.521292751	0.481509384	-0.358094256	-0.289707855	0.669973464
437274	437602	8	+	unknown	no_ovlp	CUTs	2.868596746	0.268085731	-0.517182842	0.060485468	0.678179011
439306	440882	8	+	THP2	ORF-T	ORF-T	0.163405014	-0.540831204	-0.921593702	-1.092268708	0.155960595
440202	440882	8	+	unknown	no_ovlp2	SUTs	-1.173758272	-0.129804161	0.588422581	-0.181071532	0.674566712
440882	442146	8	+	MTG2	ORF-T	ORF-T	-1.055573866	-0.48403099	0.348217868	0.186992312	0.81333265
442146	443562	8	+	DBP8	ORF-T	ORF-T	0.669986932	-0.082093445	-1.020970876	-0.301911916	0.595144167
443770	445514	8	+	NMD3	ORF-T	ORF-T	0.219141127	-0.466131346	-0.640797917	0.223805875	0.183205703
445514	447730	8	+	ATG7	ORF-T	ORF-T	-0.527450139	-0.467382038	0.992146182	0.296656509	1.311922541
448250	451298	8	+	SPC97	ORF-T	ORF-T	-0.162788622	-0.043607908	-0.152970823	-0.030439381	1.01987201
451298	452794	8	+	ENO2	ORF-T	ORF-T	-0.524990381	0.069504171	-0.193845622	-0.044068914	-0.056728424
452930	453674	8	+	CTR2, YHR17	other	other	-0.567031412	-0.755233425	0.127036415	0.049822637	0.299469191
454266	455532	8	+	FMO1	ORF-T	ORF-T	-0.888056617	-0.004678495	0.527937557	0.427337744	-0.925431178
457132	457980	8	+	YHR177W	ORF-T	ORF-T	0.323961148	0.857882951	1.006561952	1.495049719	-1.993340722
459300	461700	8	+	STB5	ORF-T	ORF-T	-0.419221089	-0.269846868	-0.022798643	-0.232611521	0.677929942
461700	462020	8	+	unknown	no_ovlp	CUTs	1.793128636	-0.422343402	-0.053405913	0.104123881	-0.506293395
462460	463804	8	+	OYE2	ORF-T	ORF-T	-0.297559859	-0.8922176	-0.527665791	0.116982826	-0.714714396
464956	465868	8	+	YHR180W	other	other	0.228995498	-0.896001734	0.356571972	0.386569892	-2.159586353
467228	468012	8	+	SVP26	ORF-T	ORF-T	-0.075432671	-0.156496132	-0.490914079	-0.535611426	-0.142737082
468012	469548	8	+	YHR182W	ORF-T	ORF-T	-0.309681786	-0.724490689	-0.598901164	-0.745543701	-1.410018745
470900	472596	8	+	GND1	ORF-T	ORF-T	-0.697552537	-0.261314944	-0.536840676	0.142835257	0.232670082
480964	482044	8	+	IKI1	ORF-T	ORF-T	-0.076940466	-0.420232689	0.263903658	-0.095681772	0.564633639
484028	484804	8	+	PTH1	ORF-T	ORF-T	-0.153955216	-0.349975976	0.276609925	0.001375085	0.165554471
484804	486316	8	+	ERG9	ORF-T	ORF-T	0.487135337	0.128530965	-0.12893948	0.165452061	0.017533594
486836	487740	8	+	YHR192W	ORF-T	ORF-T	0.51133005	-0.100721448	-0.29873524	-0.450148953	-0.272146049
488612	490500	8	+	MDM31	ORF-T	ORF-T	-0.018688238	-0.435116443	0.159532445	0.036888413	0.134762973
490604	491892	8	+	NVJ1	ORF-T	ORF-T	-0.218463613	-1.02083109	0.120476841	-0.006339335	-0.242551638
491668	491892	8	+	unknown	no_ovlp2	SUTs	-0.401081767	-0.646560513	0.275555641	0.186367108	-0.315381435
491892	493716	8	+	UTP9	ORF-T	ORF-T	1.129497188	-0.080275314	-0.611515195	-0.260680518	-0.089922137
493892	496292	8	+	RIX1	ORF-T	ORF-T	0.000852354	-0.396687108	-0.474042538	0.365056693	0.377740963
499060	499956	8	+	RPN10	ORF-T	ORF-T	0.011940506	-0.169630046	-0.039623091	0.056477159	-0.008177893
501348	502356	8	+	unknown	no_ovlp2	CUTs	2.297956726	0.273794253	-0.857196263	-0.591012942	-0.848540323
502356	504436	8	+	YHR202W	ORF-T	ORF-T	0.463265257	1.082470031	0.776016103	0.327519975	1.239321488
506292	508836	8	+	MNL1	ORF-T	ORF-T	-0.198602643	-0.460564288	-0.630753301	-0.372038736	0.192801004
508932	511996	8	+	SCH9	ORF-T	ORF-T	0.134543008	0.065579268	0.161341222	-0.227979236	0.418432682
512452	514796	8	+	SKN7	ORF-T	ORF-T	0.11454779	-0.319868854	-0.918479089	-0.246703583	-0.227596114
516596	517140	8	+	SUT173	SUTs	SUTs	1.245379931	-0.247329382	-0.135146352	-0.522115994	-0.60037501
517516	518884	8	+	BAT1	ORF-T	ORF-T	-0.395138797	-0.656489827	0.029513374	0.167713949	-0.402461039
519420	520340	8	+	CRG1	ORF-T	ORF-T	-0.179891025	0.44133533	1.209265292	0.173158962	0.055201527
522188	523060	8	+	SUT174	SUTs	SUTs	0.997584173	-0.165403904	1.261967867	1.273238479	-1.209798868
523060	529124	8	+	FLO5	ORF-T	ORF-T	0.245491155	-0.954516802	-0.528559375	-0.1135411	-0.604954855
525436	529124	8	+	unknown	no_ovlp2	SUTs	0.016517527	-1.510837548	-0.888929718	-0.193013229	-1.355012833
554172	556372	8	+	unknown	no_ovlp	CUTs	1.083142063	-0.66780364	-0.582215073	-0.59395248	-1.249763119
23085	23709	9	-	unknown	no_ovlp	SUTs	-0.684324199	-0.277783957	0.937269879	0.978302658	1.656829686
23709	26133	9	-	unknown	no_ovlp	SUTs	0.143239756	2.020202985	3.034222618	0.542511189	2.094179071
30381	31309	9	-	YIL166C	ORF-T	ORF-T	0.403419547	-0.391078311	0.28217574	-0.416409254	0.220170704
31309	33533	9	-	YIL166C	ORF-T	ORF-T	-0.332218864	0.849945484	1.047119022	0.016603088	3.121964384
32965	33533	9	-	unknown	no_ovlp2	SUTs	-0.144608078	0.206678746	-0.041358228	0.037401283	0.116218684

33533	34741	9	-	YIL165C, NIT	other	other	0.754092306	-0.004268758	-0.0439306	0.212505383	-1.293794276
34741	36045	9	-	SUT602	SUTs	SUTs	0.314279671	-0.238737155	1.041420468	1.117050047	-0.867976278
45285	45933	9	-	CUT659	CUTs	CUTs	4.771526682	0.402044931	-1.235081221	-0.35197785	0.454712683
46773	47565	9	-	COA1	ORF-T	ORF-T	0.031920542	0.045367041	-0.022557774	-0.422280763	-0.536701414
47565	48117	9	-	CUT660	CUTs	CUTs	1.397559977	-0.015644482	-0.071280149	0.093889349	-0.370568912
51429	51725	9	-	unknown	no_ovlp2	SUTs	-0.471261473	-1.385674545	1.120739205	0.698672277	0.987651135
51725	53765	9	-	GUT2	ORF-T	ORF-T	-0.275950883	-0.559634097	1.260415118	1.118750966	0.812079664
53925	55037	9	-	IMP2'	ORF-T	ORF-T	-0.331983041	0.150804128	-0.090496284	-0.291075652	-0.387684737
60885	62757	9	-	YIL151C, MC	other	other	0.021387879	-0.011118865	-0.413825851	-0.31754738	0.342499618
66797	68205	9	-	MLP2	ORF-T	ORF-T	-0.202051872	-0.302882697	-0.247679351	0.078395452	0.187345336
73429	73669	9	-	SLN1	ORF-T	ORF-T	-0.539072521	0.03601559	0.224518177	0.215091167	0.788566008
73997	75949	9	-	ECM37	ORF-T	ORF-T	0.561009912	0.141125589	-0.002518275	-0.054184922	0.554350227
75949	77341	9	-	PAN6	ORF-T	ORF-T	-0.04431115	-0.164446227	-0.282057435	-0.028944209	0.093836748
80325	83069	9	-	SSL2	ORF-T	ORF-T	0.251044371	-0.286889014	-0.190628018	-0.010436492	-0.098517774
84245	84893	9	-	unknown	no_ovlp	CUTs	1.098265824	0.130038636	-0.035403358	0.149821395	0.867079571
87893	88885	9	-	REV7	ORF-T	ORF-T	0.204027387	-0.440824076	-0.518935221	-0.473574334	-1.051406529
88885	89741	9	-	TPM2	ORF-T	ORF-T	-0.16022905	-0.883549329	-0.170449445	-0.313456817	-0.136759375
91445	92829	9	-	TMA108	ORF-T	ORF-T	-0.112366209	-0.134786836	-0.102629353	0.022969885	-0.200426444
93133	94293	9	-	unknown	no_ovlp	CUTs	1.902116089	0.273119515	-2.096564138	-0.79680196	0.269484347
94933	96429	9	-	VHS2	ORF-T	ORF-T	-0.328682137	-0.152983477	-0.309311742	0.023882131	0.087920105
96429	98373	9	-	unknown	no_ovlp	SUTs	-0.087479026	0.213238612	0.071327013	-0.153458664	0.234477476
99093	99389	9	-	RPL16A	ORF-T	ORF-T	0.783192719	-0.669898984	-1.618995768	-1.235276721	-0.191483313
99469	100757	9	-	CSM2	ORF-T	ORF-T	0.328145951	0.060403599	-0.037208367	-0.510758411	0.613296722
100517	100757	9	-	unknown	no_ovlp2	SUTs	-0.046889825	0.271352328	0.393091324	0.106437445	0.530109212
100757	102293	9	-	FKH1	ORF-T	ORF-T	-0.153630016	-0.274383682	-0.28470242	-0.354066309	0.956249639
106005	113341	9	-	TAO3	ORF-T	ORF-T	-0.16403366	-0.298846938	0.027379916	-0.067298618	0.290438221
116933	117669	9	-	YIL127C	ORF-T	ORF-T	0.350725053	-0.172250637	-0.588568949	-0.128570076	0.567181631
127341	127589	9	-	unknown	no_ovlp	SUTs	0.560578659	-1.499117103	0.347560271	-0.23639003	-0.500837747
129117	129781	9	-	unknown	no_ovlp	CUTs	2.172401858	0.225643066	-0.229739858	0.409915458	-0.336578061
136389	138053	9	-	RPI1	ORF-T	ORF-T	-0.771543113	-1.131164045	-0.863523099	0.462886413	-2.115719257
140533	141685	9	-	PRM5	ORF-T	ORF-T	0.140741068	-0.209260682	0.178364027	-0.059435674	-1.562227465
144261	148885	9	-	NUP159	ORF-T	ORF-T	-0.082153519	-0.58657586	-0.50097003	0.060191793	-0.225531194
149093	150005	9	-	POR2	ORF-T	ORF-T	0.402864759	0.386450223	-0.337113276	-0.109082936	-0.45981612
150005	151357	9	-	SUT607	SUTs	SUTs	0.64014976	0.231162449	0.218948517	0.238616798	-0.009846637
154213	154877	9	-	SUT608	SUTs	SUTs	-0.373124342	-1.044188195	0.446561534	1.025912066	0.517581876
154877	155861	9	-	SUT609	SUTs	SUTs	1.069835169	-0.42781727	0.036151815	-0.367588258	-0.176649336
158917	160245	9	-	SEC24	ORF-T	ORF-T	-0.406404526	-0.061518836	-0.285842112	-0.041874447	-0.081475556
163005	165957	9	-	PFK26	ORF-T	ORF-T	0.115889606	-0.679860835	0.512916313	0.374271931	-0.089914842
167421	169773	9	-	SLM1	ORF-T	ORF-T	-0.130474126	-0.532997532	0.528332178	0.450953284	0.116069089
169893	171533	9	-	SHQ1	ORF-T	ORF-T	0.451610713	0.252971256	-0.640693902	-0.225070491	0.534850513
172637	173629	9	-	YIL102C-A	ORF-T	ORF-T	0.639735109	-0.674116355	-0.355301664	-0.248371624	-0.158197688
176941	177429	9	-	XBP1, YIL100	other	other	-0.618126971	-0.504596024	1.598833229	1.481206166	1.390056366
179741	180261	9	-	FMC1	ORF-T	ORF-T	-0.434229477	-1.11406363	0.109474554	-0.183425241	-0.561979394
182013	183149	9	-	YIL096C	ORF-T	ORF-T	0.280364145	0.023049124	-0.260919075	0.06234477	0.583967073
186317	187685	9	-	LYS12	ORF-T	ORF-T	0.340582324	-0.650034918	-0.503241045	0.262823891	-0.420176979
187869	188853	9	-	RSM25	ORF-T	ORF-T	-0.583247157	-0.392250659	-0.141691077	-0.469712025	-0.117729937
190981	193237	9	-	YIL091C	ORF-T	ORF-T	0.576602524	-0.150093352	-0.672179206	-0.251795742	0.609451461
197837	199469	9	-	AVT7	ORF-T	ORF-T	-0.193675193	-0.220843535	-0.179920884	-0.009844509	-0.375296624
199557	200157	9	-	YIL087C, YIL(	other	other	-0.24551304	-0.627052058	0.715904944	0.175818586	-0.0599031
200469	202077	9	-	YIL086C, KTR	other	other	0.379281719	-0.644026864	-0.776378567	0.004289119	-0.214911878
202077	203285	9	-	SDS3	ORF-T	ORF-T	-0.052533547	-0.247986974	-0.245541729	0.014119932	0.016890732
203445	204765	9	-	YIL083C	ORF-T	ORF-T	0.297931265	-0.066561667	-0.112620455	0.049180883	0.101592737
210829	212029	9	-	AIR1	ORF-T	ORF-T	0.719388132	0.099729483	-1.352304784	-0.867555078	0.343718671
214861	216477	9	-	YIL077C	ORF-T	ORF-T	-0.287175715	-0.688182554	0.068046754	0.253687609	-0.121603521
217781	220757	9	-	RPN2	ORF-T	ORF-T	-0.097276536	-0.295838247	0.112802501	0.089021399	-0.079969954
221029	222565	9	-	SER33	ORF-T	ORF-T	-0.206772255	-0.444015279	-0.570879153	-0.136526588	-0.192699992
222565	222813	9	-	unknown	no_ovlp2	SUTs	-0.185624827	0.515597842	0.303826054	0.398633505	0.688859957
222813	226517	9	-	SPO22	ORF-T	ORF-T	-0.084355237	0.645532122	0.863244152	0.239161068	1.0043902
224573	226517	9	-	unknown	no_ovlp2	SUTs	0.047008352	0.574840851	0.229963227	-0.057374724	0.902157532
228445	229997	9	-	PCI8	ORF-T	ORF-T	-0.074691785	-0.383689422	0.318641446	-0.040729733	0.407350682
230157	231149	9	-	MAM33	ORF-T	ORF-T	-1.325977419	-1.198969058	0.321624077	0.155245134	0.325473767
231957	232381	9	-	RPS24B	ORF-T	ORF-T	0.478126342	-0.805469272	-0.759035132	-1.125423953	0.153209057
232989	235525	9	-	SEC6	ORF-T	ORF-T	-0.347193236	-0.716896371	-0.088247127	0.01154137	0.123928677
235677	237813	9	-	YIL067C	ORF-T	ORF-T	0.218540182	0.032586437	-0.472275998	-0.340401743	-0.627817743
241245	241813	9	-	FIS1	ORF-T	ORF-T	-0.279623734	-0.205951814	0.605835606	0.341468581	0.135381404
242757	243773	9	-	YRB2	ORF-T	ORF-T	-0.115790045	-0.491306581	-0.00158843	-0.066272563	0.264843657
243893	244493	9	-	ARC15	ORF-T	ORF-T	-0.122506282	-0.435488433	0.135114673	-0.086442627	0.059539069
244493	245573	9	-	SNP1	ORF-T	ORF-T	-0.362072391	-0.374885795	-0.107934652	-0.11211689	0.107762642
246221	247413	9	-	unknown	no_ovlp	CUTs	1.098879453	0.430912914	0.260083256	0.110144547	-0.381191539
252853	254101	9	-	YIL055C	ORF-T	ORF-T	0.450089675	-0.097343753	0.478851119	0.698533273	1.007399
256549	257061	9	-	RPL34B	ORF-T	ORF-T	0.077150298	-1.238565541	-0.762030983	-0.419448013	-0.522248678
257717	258349	9	-	MMF1	ORF-T	ORF-T	0.084686695	-0.306829518	0.22557845	0.11058343	0.25099246
258349	259909	9	-	CUT664	CUTs	CUTs	1.169781011	0.222142892	-1.24930068	-0.038231951	-0.845700102
265053	267845	9	-	YIL047C-A, S'	ORF-T	ORF-T	0.327404285	-0.261781757	-1.079505374	-0.421032034	-0.311163315
272869	273869	9	-	AGE2	ORF-T	ORF-T	0.027115083	-0.453383525	-0.668456491	-0.384088028	-0.419684535
273973	274949	9	-	CBR1	ORF-T	ORF-T	-0.160926036	-0.561777496	-0.404191776	-0.044755704	-0.034270759
274949	276317	9	-	PKP1	ORF-T	ORF-T	-0.121261694	-0.23187672	0.200852059	0.080857828	0.183819563
277277	278261	9	-	unknown	no_ovlp	CUTs	1.366104673	0.199906637	-0.376932956	-0.321570144	-0.731338671
279997	282893	9	-	NOT3	ORF-T	ORF-T	-0.202527299	-0.230498469	-0.234833191	0.20021649	-0.03921557
287621	289021	9	-	CKA1	ORF-T	ORF-T	-0.152623335	-0.8172062	-0.333240202	-0.189813463	-0.227622164
289317	290117	9	-	CAP2	ORF-T	ORF-T	-0.111705683	-0.412043453	0.120499724	-0.042349797	-0.422912383
290397	291773	9	-	BCY1	ORF-T	ORF-T	-0.575239388	-0.285594124	0.377895071	0.040581714	0.79955055

295965	300069	9	-	SSM4	ORF-T	ORF-T	-0.097109472	-0.326824647	-0.383023806	-0.11234097	0.078339784
300405	301357	9	-	unknown	no_ovlp	SUTs	0.623247014	-0.096258466	0.457836336	-0.000567403	-0.269238165
303525	304149	9	-	KRE27	ORF-T	ORF-T	-0.071271424	-1.119880954	-0.177145544	0.130313044	0.215871287
304341	307965	9	-	IRR1	ORF-T	ORF-T	-0.138710033	-0.040362614	-0.199126815	-0.007029832	0.732304731
308525	309245	9	-	YIL025C, YIL(	other	other	0.647017765	-0.745178908	0.236207576	-0.426072436	0.975181277
309245	310581	9	-	YKE4	ORF-T	ORF-T	-0.399458627	-0.397073966	0.485414914	-0.34433709	-0.419025792
313925	314893	9	-	YIL020C-A, H	ORF-T	ORF-T	0.024616801	-0.291358509	-0.495541219	-0.314043879	0.323066411
318061	321933	9	-	VID28	ORF-T	ORF-T	-0.103297592	-0.180368706	0.273212625	0.224134517	0.066342383
321109	321933	9	-	unknown	no_ovlp2	CUTs	2.585904036	-0.057095946	-0.638947958	-0.510241086	-0.415681084
325181	325517	9	-	YIL014C-A	ORF-T	ORF-T	-0.143966585	0.069990695	1.281627007	0.456875826	-2.260876934
328013	330101	9	-	PDR11	ORF-T	ORF-T	0.122606126	1.906388281	1.510888305	2.362276949	0.097998905
335637	336245	9	-	EST3	ORF-T	ORF-T	-0.242265015	-0.648150578	-0.512577779	-0.362316613	-0.617820016
336245	336677	9	-	unknown	no_ovlp2	CUTs	1.261099361	0.369157096	-0.349440134	-0.491898613	-0.980318285
336677	338613	9	-	YILCdelta5	other	other	0.163110016	0.150399491	0.567928783	0.678809611	-0.311106861
342893	343669	9	-	NAS2	ORF-T	ORF-T	0.113314412	-0.424281621	-0.123866012	-0.283554774	-0.315622477
347869	348373	9	-	BET1	ORF-T	ORF-T	-0.319679176	-0.919802325	0.308949693	-0.168454251	-0.011516936
349757	350093	9	-	CUT667	CUTs	CUTs	2.345517287	0.018995707	-0.017691824	0.181894265	0.181032792
350517	353629	9	-	INP51	ORF-T	ORF-T	-0.714990943	-0.630497364	0.05550455	-0.149798911	-0.042150236
355845	356973	9	-	SGN1	ORF-T	ORF-T	0.049633617	-0.281644853	-0.012385845	-0.447947994	-0.616557771
357389	360469	9	-	MPH1	ORF-T	ORF-T	-0.177514862	-0.211531502	0.04779588	-0.314523128	-0.035003763
362237	362981	9	-	CUT668	CUTs	CUTs	1.30575836	0.157280133	-0.302428814	0.564050532	0.771539872
367757	369957	9	-	PAN1	ORF-T	ORF-T	-0.719216698	-0.174534198	-0.178379396	0.102113239	-0.390170761
372997	374317	9	-	PRI1	ORF-T	ORF-T	0.202520404	-0.071995538	-0.210775045	-0.283603841	-0.042692392
377189	378277	9	-	STS1	ORF-T	ORF-T	0.400004199	-0.723121579	-0.137433474	0.072845417	-0.258450557
378277	379781	9	-	unknown	no_ovlp	SUTs	0.893414773	-0.062158911	-0.821049727	0.744240454	-0.566651019
381181	382157	9	-	CUT669	CUTs	CUTs	3.188444039	0.344539483	-0.587504663	-0.091280911	-0.491737629
383357	384101	9	-	MET28	ORF-T	ORF-T	-0.307574586	-0.100963181	0.916786231	0.394939465	-0.021052651
385637	386525	9	-	YIR018C-A	ORF-T	ORF-T	-0.349275212	-0.256832994	1.280598512	1.404772465	-0.955401491
389405	397077	9	-	MUC1	ORF-T	ORF-T	-0.214644019	-0.173385392	0.047523953	0.3651818	0.448305375
392925	397077	9	-	unknown	no_ovlp2	SUTs	-0.487400812	-0.507521205	0.328589661	0.338358685	0.34999014
398173	399821	9	-	unknown	no_ovlp	SUTs	0.917220583	0.176397272	-0.846818423	-0.715774235	-0.365985789
402669	403509	9	-	YIR023C-A, Y	other	other	0.75797314	-0.416599114	0.141296643	-0.528908924	-0.524408592
404773	405997	9	-	YVH1	ORF-T	ORF-T	0.491533636	-0.318540952	-0.805874847	-0.131933244	-0.086377479
405997	408821	9	-	DAL1	ORF-T	ORF-T	-0.718981115	0.667475568	1.330672644	-0.425290239	-0.726977683
406837	408821	9	-	unknown	no_ovlp2	SUTs	0.138010203	0.624560073	0.211880512	-0.351107852	-0.467844198
408821	410565	9	-	unknown	no_ovlp	CUTs	1.325571366	0.532253928	0.000308833	-0.966557961	-1.927654131
411973	412781	9	-	DCG1	ORF-T	ORF-T	0.230200933	0.431906546	0.127867016	-0.38271722	-2.196593937
412957	414765	9	-	DAL7	ORF-T	ORF-T	0.762320675	-0.411853933	-0.579646945	-0.661569412	-1.138465439
414909	415669	9	-	DAL3	ORF-T	ORF-T	-0.38485722	-0.260124052	0.191299128	-0.389102396	-0.428714865
419541	420765	9	-	LYS1	ORF-T	ORF-T	0.948807908	0.210253832	-0.268696468	-0.007406458	-0.240463868
420957	421813	9	-	YIR035C	ORF-T	ORF-T	1.852304635	0.819019254	-0.522586279	-0.248644314	-0.394637506
422037	422885	9	-	IRC24	ORF-T	ORF-T	-0.724920086	-0.457486341	0.332196911	-0.5417369	0.544718319
423677	424525	9	-	GTT1	ORF-T	ORF-T	-0.220336157	0.084743558	0.996820255	0.3272772	1.02338242
430381	432341	9	-	YPS6	ORF-T	ORF-T	-0.091952928	-0.056506551	0.788149506	0.458126917	-1.289059128
434885	436053	9	-	YIR042C	ORF-T	ORF-T	0.029235056	-0.362867057	0.039707207	-0.528059006	-2.614176427
436829	438197	9	-	YIR043C	other	other	0.472520611	-0.982468393	-0.769158464	-1.623899395	-2.064884629
437949	438197	9	-	unknown	no_ovlp2	SUTs	0.986851663	-0.953982575	-0.875225258	-1.675874624	-2.082365822
28961	30321	9	+	YIL168W, SDI	other	other	0.531510376	0.065864652	-0.127697462	-0.228498634	-1.90288503
31185	32449	9	+	SUT175	SUTs	SUTs	1.404580218	0.250899232	0.065064873	0.001278754	-2.586426453
34921	37473	9	+	SUT176	SUTs	SUTs	1.051295084	-0.086480982	-0.645804029	-0.268429021	-1.275966107
37473	39201	9	+	SUC2	ORF-T	ORF-T	-0.421038288	-0.496610775	1.339655672	1.454003313	-1.612788061
39441	41361	9	+	SUT177	SUTs	SUTs	1.305496762	0.175911191	-0.701258038	0.14373136	-0.843360817
41361	41793	9	+	unknown	no_ovlp2	SUTs	0.507453308	0.244656232	-0.618321188	0.12768073	-0.491214204
41793	44129	9	+	BNR1	ORF-T	ORF-T	-0.034303416	-0.364389021	-0.358287112	0.042105881	0.25607552
46209	46921	9	+	YIL158W	ORF-T	ORF-T	0.296962731	-0.494095208	-0.590819675	-0.155633434	0.018803337
47777	48033	9	+	YIL156W-A, \	other	other	0.645908509	0.023899791	0.370988039	-0.344777329	0.48111206
48033	51457	9	+	UBP7	ORF-T	ORF-T	-0.267805698	-0.098895068	-0.058758529	0.047572388	-0.463226866
53977	55201	9	+	unknown	no_ovlp	SUTs	0.707215239	-0.189595507	0.557499269	0.903161092	0.94055953
55201	56425	9	+	RRD1	ORF-T	ORF-T	-0.55252289	-0.254401787	0.149002593	0.045378285	0.026586583
56577	57385	9	+	YIL152W	ORF-T	ORF-T	0.252842165	0.096561305	0.177066658	-0.358838655	-0.389547828
68713	69161	9	+	RPL40A	ORF-T	ORF-T	-0.104848284	-1.257591426	-0.447825656	-0.555352698	0.036498026
76329	77369	9	+	SUT178	SUTs	SUTs	1.117424958	0.303239682	0.816891934	0.451726795	1.078367684
78001	80337	9	+	TID3	ORF-T	ORF-T	-0.046822153	-0.197868565	0.31542685	-0.103999911	0.492768738
83273	84945	9	+	CCT2	ORF-T	ORF-T	0.028396879	-0.316332955	-0.515158614	-0.1218733	-0.15757454
85257	87945	9	+	YIL141W, AXI	ORF-T	ORF-T	-0.430997307	-0.315031853	0.345434495	-0.082253792	0.238419056
88937	89689	9	+	unknown	no_ovlp	CUTs	1.743439106	0.566150249	-0.824082564	-0.18831464	-0.075467037
92969	93641	9	+	unknown	no_ovlp	CUTs	1.053362974	-0.107698969	0.199737883	-0.025022944	0.129363953
93641	94905	9	+	OM45	ORF-T	ORF-T	-1.058100024	-0.521781832	2.296702758	1.408369053	0.454312248
97257	97521	9	+	unknown	no_ovlp	CUTs	3.01884561	-1.255596503	-0.926395575	-0.465628712	-0.296955166
100009	100897	9	+	CUT187	CUTs	CUTs	1.217078728	0.12295433	-0.015561483	0.058598377	0.65102591
102721	105737	9	+	ASG1	ORF-T	ORF-T	-0.077968745	-0.501099175	-0.689153322	-0.222431294	-0.086596009
113737	117001	9	+	MET18	ORF-T	ORF-T	-0.232098776	-0.472567547	-0.28238297	0.051896452	0.028134447
117929	120641	9	+	STH1	ORF-T	ORF-T	-0.567297006	-0.942783491	-0.883372196	-0.141911184	-0.225737778
122409	126001	9	+	KGD1	ORF-T	ORF-T	-0.315128159	-0.243029712	0.472405125	0.139835284	0.083781185
126177	127249	9	+	AYR1	ORF-T	ORF-T	-0.686431781	-0.675318011	0.246477083	0.212825903	0.216966577
127657	128137	9	+	SIM1	ORF-T	ORF-T	0.081090604	-0.498130024	-0.389105079	-0.51065159	-0.435532996
130385	131817	9	+	POG1	ORF-T	ORF-T	0.3810566	-0.668353853	-0.181492685	-0.18700044	-0.49120731
132225	133729	9	+	QDR2	ORF-T	ORF-T	-0.316675437	-2.32464363	-1.892679037	-1.296079028	-1.26839044
134041	135249	9	+	unknown	no_ovlp2	SUTs	0.315117838	0.020513968	0.482518833	0.296988644	-0.716925371
135249	136929	9	+	QDR1	ORF-T	ORF-T	-0.374863381	-0.263016353	0.536735124	0.860504305	-1.648632738
136185	136929	9	+	unknown	no_ovlp2	CUTs	1.067508468	-0.642203157	-0.331697612	0.375496444	-1.853850166
139641	140601	9	+	RHO3	ORF-T	ORF-T	-0.272837088	-0.624003423	-0.503100857	-0.027621437	-0.106883194

142873	144201	9	+	HIS5	ORF-T	ORF-T	0.77665171	-0.124455033	-0.325497637	-0.175198538	-1.227922333
150529	151209	9	+	SDP1	ORF-T	ORF-T	-1.320554859	-0.778283663	1.35882495	1.264650887	1.1603783
151577	154977	9	+	HOS4	ORF-T	ORF-T	-0.152859577	-0.494870746	-0.213858496	-0.017834054	-0.202638516
155305	155889	9	+	COX5B	ORF-T	ORF-T	-0.426399645	-0.809996737	0.344544623	0.748734231	0.371421599
156025	157361	9	+	MNI1	ORF-T	ORF-T	0.017847874	-0.610620157	-0.586868875	-0.251246543	0.084558659
160689	163025	9	+	YIL108W	ORF-T	ORF-T	-0.052253088	-0.221947032	-0.092705762	0.143515487	-0.195400335
165001	166513	9	+	unknown	no_ovlp	SUTs	0.734979315	0.326388896	-0.363957737	-0.598473652	-0.420129672
166513	167545	9	+	MOB1	ORF-T	ORF-T	-0.248160545	-0.177858272	0.078941482	0.217897706	0.024656908
171697	173121	9	+	DPH1	ORF-T	ORF-T	0.419445625	-0.094902284	-0.619584745	-0.134468236	0.438332684
173849	174305	9	+	SUT181	SUTs	SUTs	0.6283768	-0.587566325	-0.333601812	-0.072905057	-0.07800792
174305	175257	9	+	unknown	no_ovlp2	SUTs	0.315566657	-0.234438927	0.156819089	0.173704539	0.08304802
175257	177809	9	+	YIL100W	other	other	0.50385242	0.340822997	-0.876137685	-1.188534138	-1.030594951
177441	177809	9	+	unknown	no_ovlp2	SUTs	0.513889375	0.412650308	0.593799137	0.180549178	-0.089398397
177809	180417	9	+	YIL100W, SG	other	other	-0.842739011	0.54259915	2.550685981	2.914071248	2.373344562
179681	180417	9	+	unknown	no_ovlp2	SUTs	-0.129681312	0.161256016	-0.113433835	0.009980765	0.259902094
180417	182049	9	+	FYV10	ORF-T	ORF-T	-0.033154117	-0.412378699	0.950880305	0.523048642	-0.264028346
183769	186441	9	+	PRK1	ORF-T	ORF-T	-0.0517226	-0.47207904	-0.329239719	-0.092388426	-0.443147596
187961	188417	9	+	CUT188	CUTs	CUTs	3.230648323	0.170756926	-1.666394038	-0.153800786	-0.17222464
189017	191105	9	+	YIL092W	ORF-T	ORF-T	0.200667964	-0.270192231	-0.356508863	-0.554987224	0.307277154
193449	195393	9	+	ICE2	ORF-T	ORF-T	-0.121542293	-0.527629483	-0.215563303	-0.135954512	0.33347012
195569	196617	9	+	YIL089W	ORF-T	ORF-T	0.763305077	-0.55703962	0.003965915	-0.189915077	-0.352071291
199633	200521	9	+	unknown	no_ovlp	CUTs	1.267034007	0.011353248	-0.186231925	0.297784104	-0.421851775
202257	202881	9	+	SUT182	SUTs	SUTs	2.167272411	-1.173384602	-1.504896417	-0.535141646	0.236889093
212465	214785	9	+	THS1	ORF-T	ORF-T	-0.21875033	-0.312743835	-0.490367771	-0.102245028	-0.207520778
216657	217705	9	+	SEC28	ORF-T	ORF-T	-0.272990387	-0.629569951	-0.095678842	-0.11476139	-0.162890007
220961	221473	9	+	CUT191	CUTs	CUTs	2.470618589	0.474569238	-0.05709744	0.154132388	0.604903978
222865	224473	9	+	SUT183	SUTs	SUTs	0.574911925	-0.41892633	-0.893488067	-0.273534384	-0.533958844
241161	242001	9	+	unknown	no_ovlp	SUTs	-0.038297164	0.498788287	0.056249617	0.476272073	0.062439769
242001	242705	9	+	YIL064W	ORF-T	ORF-T	0.35587042	-0.275801693	-0.286816376	0.161712955	0.858223015
243673	244097	9	+	unknown	no_ovlp	SUTs	-0.013395669	-1.172334764	0.618978263	-0.198615523	1.363086224
249681	252017	9	+	VHR1	ORF-T	ORF-T	0.036865256	-1.069046382	-0.29206575	-0.221323292	-0.671246428
253009	253265	9	+	unknown	no_ovlp	SUTs	-0.144567207	0.522298922	-0.428560627	-0.254614563	-0.192854121
255097	256017	9	+	RHR2	ORF-T	ORF-T	-0.448829719	-0.022666569	-0.549004233	-0.001761464	-0.292584499
257681	258809	9	+	unknown	no_ovlp	CUTs	1.403022673	-0.486486534	-0.030499037	-0.492475163	-0.318963032
258809	259889	9	+	PCL7	ORF-T	ORF-T	-0.043624552	-0.278125765	0.572680103	0.417594055	0.472080563
260209	260985	9	+	DFG10	ORF-T	ORF-T	0.822443456	-0.590212413	-0.222419468	-0.588448745	-0.197262912
261145	262057	9	+	NEO1	ORF-T	ORF-T	-0.233809436	-0.892350319	-0.398897439	0.018734963	-0.1839197
268385	268617	9	+	YIL046W-A	ORF-T	ORF-T	-0.563703424	-0.322233548	0.190518525	0.540955105	0.65741169
268617	270673	9	+	MET30	ORF-T	ORF-T	-0.030378201	-0.021978995	0.159826726	0.011043465	0.4188669
270969	272873	9	+	PIG2	ORF-T	ORF-T	0.234611783	-0.247242225	0.388071289	0.426859039	-1.432820992
274089	274593	9	+	CUT194	CUTs	CUTs	2.232326098	-0.25295903	-0.899548882	-0.268028516	0.139113496
276513	277593	9	+	GVP36	ORF-T	ORF-T	0.006348054	-0.001988111	0.123817082	-0.009784173	0.077618449
277713	278233	9	+	APQ12	ORF-T	ORF-T	0.836286418	-0.625304187	-0.658871503	-0.565623515	-0.375807308
278393	279857	9	+	TED1	ORF-T	ORF-T	-0.279641255	-0.52841177	-0.470709431	-0.051972777	-0.640093039
283137	284753	9	+	SUT186	SUTs	SUTs	0.007262117	-0.522848582	-0.620097384	-0.394118776	-1.622266954
285521	286953	9	+	CST6	ORF-T	ORF-T	-0.196506755	-0.874150908	0.034081383	0.239483996	-0.401769234
289257	290249	9	+	SUT187	SUTs	SUTs	1.00334771	-1.736488052	-1.783118689	-0.48299348	0.05398714
292457	295825	9	+	ULP2, YIL030	ORF-T	ORF-T	0.052910608	-0.315808521	-0.022152341	0.267426315	0.681753674
301577	303313	9	+	YIL028W	other	other	0.292765313	-0.260264367	0.732368369	0.264484005	0.518154078
304457	305593	9	+	unknown	no_ovlp	SUTs	0.657042453	-0.390344052	-0.075489299	0.281891616	-0.353008271
310433	311057	9	+	unknown	no_ovlp	SUTs	0.72545423	0.399906608	0.802883254	-0.024460884	-0.534630482
311057	312689	9	+	TIM44	ORF-T	ORF-T	-0.373546513	-0.57732637	-0.385480304	-0.18779542	-0.437864051
312849	313993	9	+	RPB3	ORF-T	ORF-T	-0.169619415	-0.677086895	-0.732067166	-0.018054789	-0.240081124
315089	316225	9	+	FAF1	ORF-T	ORF-T	0.406276901	-0.256676068	-0.606309053	-0.088345991	0.873300905
316769	317217	9	+	RPL2B	ORF-T	ORF-T	0.706353057	-0.844077796	-1.229521008	-1.096356133	-0.084544436
321433	322025	9	+	SNL1	ORF-T	ORF-T	0.376549955	-0.315960982	-0.504641801	-0.818442315	-0.274960154
322289	323321	9	+	unknown	no_ovlp2	SUTs	0.133911608	0.901194506	-0.673247425	-0.414537895	-1.213905475
323321	324177	9	+	BAR1	ORF-T	ORF-T	0.226061376	0.872317499	-0.590699045	-0.355218843	-1.102231537
325937	328105	9	+	MNT3	ORF-T	ORF-T	0.032124778	-0.77383929	-0.377077085	0.059281501	-0.227958565
333657	334489	9	+	TIR3	ORF-T	ORF-T	-1.480624149	-0.116898891	1.034910359	3.121731539	0.572732822
334873	335721	9	+	DOT5	ORF-T	ORF-T	0.440215919	0.36105985	0.30792975	-0.193178005	-0.117949972
337337	339089	9	+	SUT190	SUTs	SUTs	1.273914677	0.420567885	0.214436623	-0.257666891	-0.161090007
339089	339337	9	+	unknown	no_ovlp2	SUTs	-0.014583369	-0.349081685	-0.353736387	0.234876707	-0.360447185
339337	341657	9	+	FAA3	ORF-T	ORF-T	-0.147367951	-0.140402426	-0.780438572	0.272496446	-0.119741615
342497	342953	9	+	URM1	ORF-T	ORF-T	0.230690455	-0.171465966	-0.345480422	-0.506615172	-0.099736284
344041	345401	9	+	YIA6	ORF-T	ORF-T	-0.73852885	-0.637652111	0.404549744	0.310118035	0.436172998
345569	347985	9	+	EPS1	ORF-T	ORF-T	-0.663367254	-0.673534939	-0.250758816	0.059492211	0.085427282
349081	350089	9	+	CFD1	ORF-T	ORF-T	0.602271061	0.329290056	-0.089743494	0.092636657	0.513737526
350281	350601	9	+	YIL002W-A	ORF-T	ORF-T	-0.002171326	-0.374449377	-0.203863693	-0.203378162	-0.029173016
353857	355553	9	+	YIL001W	ORF-T	ORF-T	-0.152380522	-0.623634725	-0.166328481	-0.278141851	-0.070796585
357113	357561	9	+	SUT191	SUTs	SUTs	0.169120038	0.309570079	1.339433434	-0.385076372	-0.161016266
360841	362993	9	+	YIR003W	ORF-T	ORF-T	-0.359541447	-0.356257093	-0.246510507	-0.043558992	-0.345945202
363185	364633	9	+	DJP1	ORF-T	ORF-T	-0.012722836	-0.581594395	-0.417663697	-0.171516076	-0.313939501
364889	365377	9	+	IST3	ORF-T	ORF-T	0.219113697	-0.426346366	0.368879625	-0.119439823	-0.974601617
369185	370673	9	+	unknown	no_ovlp	SUTs	-0.250072538	0.191042913	0.005125294	0.163885174	0.310492538
370673	373105	9	+	YIR007W	ORF-T	ORF-T	-0.603419788	-0.402145397	0.574860745	0.547964566	-0.66666455
374473	375281	9	+	MSL1	ORF-T	ORF-T	0.432785196	-0.23667639	-0.0541855	-0.626089091	-0.335741881
375377	377265	9	+	DSN1	ORF-T	ORF-T	-0.111281996	-0.412065624	-0.052399835	0.106085717	0.654471089
378465	379849	9	+	SQT1	ORF-T	ORF-T	0.326966782	-0.231049975	-0.946320181	-0.329737656	-0.04563389
381089	381945	9	+	YIR014W	ORF-T	ORF-T	-1.324503457	-0.431512589	1.195754718	0.834092313	0.647704952
381945	383505	9	+	RPR2, YIR01	other	other	-0.320614489	-0.253402613	0.633516475	0.520169609	-0.531625871
383969	384705	9	+	SUT192	SUTs	SUTs	1.695426903	1.017511865	0.774767503	-0.227498158	0.08975146

384705	385769	9	+	YAP5	ORF-T	ORF-T	0.2022441	-0.234761517	0.268085038	0.013791885	-0.187763114
389433	393153	9	+	SUT194	SUTs	SUTs	-0.050081593	0.157459602	0.145984785	0.621998985	-0.351089814
397257	398393	9	+	MRS1	ORF-T	ORF-T	-0.457474383	-0.850596208	-0.411767737	-0.10624449	0.121767091
398713	399337	9	+	YIR021W-A, S	other	other	-0.11633131	-0.180999768	-0.339573963	0.065591348	0.021349037
399753	402777	9	+	DAL81	ORF-T	ORF-T	-0.075469142	-0.451964113	-0.673665791	-0.263430487	-0.440163416
403633	404889	9	+	MND2	ORF-T	ORF-T	0.233952707	-0.346490009	0.099273967	-0.196408917	-0.673949857
410889	411841	9	+	DAL2	ORF-T	ORF-T	0.114147831	0.130923649	0.215987759	-0.418226884	-2.16303498
415049	416689	9	+	unknown	no_ovlp	CUTs	1.861030959	0.054621801	-0.545764218	-0.338281881	-0.216188275
416689	417689	9	+	MGA2	ORF-T	ORF-T	0.561733047	0.329939212	-0.445919508	-0.153024445	0.495534411
421033	423073	9	+	SUT197	SUTs	SUTs	1.024258589	0.406050046	-0.860493789	-0.18386553	-0.36379244
423073	423761	9	+	YIR036W-A, I	other	other	-0.612305783	-0.317769254	0.312209889	-0.169011278	0.662840621
424793	430713	9	+	unknown	no_ovlp	SUTs	0.260737591	0.377565709	0.346659471	0.397876513	-0.276132462
22309	22749	10	-	CUT670	CUTs	CUTs	2.479662852	0.496178253	-0.000932883	0.072135715	-1.060329309
27093	28333	10	-	YJL216C, YJL2	other	other	1.032566308	0.127922607	-0.741409062	-1.174204762	-2.011983707
30077	30717	10	-	unknown	no_ovlp	CUTs	1.127769534	0.080653924	0.364280777	0.841010752	-0.978013867
33693	36301	10	-	OPT1	ORF-T	ORF-T	-0.870073774	-1.899559273	-1.00394757	0.198961438	0.291748712
36301	36653	10	-	unknown	no_ovlp	SUTs	0.271693754	-0.118995275	0.177816882	0.115767838	0.37417837
36669	37845	10	-	unknown	no_ovlp	SUTs	0.71441155	0.752336542	-0.07507073	-0.117364976	0.643714504
39965	41237	10	-	unknown	no_ovlp2	SUTs	-0.298220136	-0.629367244	-0.41322892	-0.249733088	0.312559842
41237	47437	10	-	LAA1	ORF-T	ORF-T	-0.20461176	-0.564838629	-0.459456851	0.003195876	-0.082661855
47437	49965	10	-	YJL206C	ORF-T	ORF-T	-0.272256024	-0.518150154	0.454861419	0.352618588	-0.460705024
49965	50261	10	-	NCE101	ORF-T	ORF-T	-0.28785506	-1.121945103	0.231293717	0.288122673	-0.107904962
50261	50573	10	-	unknown	no_ovlp2	SUTs	0.352717694	-0.15810679	0.556058034	0.185642469	0.504346093
50573	53157	10	-	RCY1	ORF-T	ORF-T	-0.039412535	-0.309561103	-0.024929552	0.054561552	-0.011393446
53605	54093	10	-	unknown	no_ovlp	CUTs	1.687584273	0.028013049	-1.259523646	-0.420621464	1.673941077
56341	58829	10	-	ACO2	ORF-T	ORF-T	0.778437216	-0.497413969	-0.905669892	0.350051374	-0.56674844
59493	60165	10	-	MBB1	other	other	0.228650529	-0.238624957	0.731540305	0.23106006	0.621345256
63045	63597	10	-	CUT671	CUTs	CUTs	2.982272633	0.172521264	-1.057372824	0.335665228	-0.698111008
67725	68805	10	-	ELO1	ORF-T	ORF-T	0.465562438	-0.063321955	-0.516619113	0.066177533	0.152526855
72629	73453	10	-	SOP4	ORF-T	ORF-T	-0.399581721	-0.566911013	0.204534884	-0.437334998	0.255191584
74773	75341	10	-	RPS22A	ORF-T	ORF-T	-0.657229466	-0.407763542	-0.22373928	-0.067493825	0.075577413
76597	79901	10	-	SWE1	ORF-T	ORF-T	-0.163860345	-0.596832937	-0.084579537	-0.216556435	0.834089179
79341	79901	10	-	unknown	no_ovlp2	CUTs	1.947611652	-0.293905664	0.55974812	-0.414499538	0.397173211
81821	83373	10	-	YJL185C	ORF-T	ORF-T	-0.460187378	0.826602111	2.034480096	0.77604601	1.793405061
82989	83373	10	-	unknown	no_ovlp2	SUTs	0.768059728	0.346687258	-0.331451791	-0.530717266	-0.219963482
83373	83813	10	-	unknown	no_ovlp	CUTs	1.423817904	-0.711237074	-0.366665368	-0.384032601	-0.209321208
87509	88573	10	-	ATP12	ORF-T	ORF-T	-0.950329319	-0.423279059	0.294965854	-0.438041227	0.400582154
89229	90109	10	-	ATG27	ORF-T	ORF-T	-0.582930506	-1.136193212	-0.422009867	-0.319051044	-0.043142443
91797	94597	10	-	SWI3	ORF-T	ORF-T	-0.448575302	-0.642956491	-0.3326789	-0.091072831	0.214190002
96101	96549	10	-	RFA3	ORF-T	ORF-T	0.147252054	-0.410761054	0.076549106	-0.137738681	0.54841032
96685	97485	10	-	CUT672	CUTs	CUTs	1.64045959	0.111019671	0.499832975	0.192787779	0.26153606
99557	100925	10	-	YJL171C	ORF-T	ORF-T	-0.000221153	-0.322469615	-0.492020966	-0.137649221	-0.241998549
100925	101774	10	-	unknown	no_ovlp	SUTs	0.360843751	0.80190907	0.01667563	0.510603247	0.065111086
102022	104526	10	-	SET2	ORF-T	ORF-T	-0.534464556	-0.404171097	-0.643055703	0.003197941	-0.071583921
106902	109622	10	-	HAL5	ORF-T	ORF-T	0.127725598	-0.699422408	-0.27605283	0.098891638	-0.458306947
109846	111366	10	-	TPK1	ORF-T	ORF-T	-0.690664951	-0.629489416	0.623157495	0.021612267	0.503583413
111366	113382	10	-	YJL163C	ORF-T	ORF-T	-1.312175692	-0.421462565	1.869468185	0.883180936	2.584640455
113718	115422	10	-	JJJ2	ORF-T	ORF-T	0.105442662	-0.35657527	-0.008526674	-0.3214332	0.170135707
116150	117190	10	-	SUT621	SUTs	SUTs	0.962613048	-0.044706337	0.114943455	-0.107777334	-0.621735658
118750	119118	10	-	YJL160C	ORF-T	ORF-T	2.368903628	-0.731370625	-0.447371081	0.440545866	-2.030021863
119118	119686	10	-	CUT674	CUTs	CUTs	2.80400361	0.508132851	0.370356672	0.121388833	-1.80636378
120582	121332	10	-	unknown	no_ovlp	SUTs	0.506857242	1.006363617	-0.419608727	-0.108579201	-0.897273871
122148	122980	10	-	CIS3	ORF-T	ORF-T	-0.402013629	-0.046715039	0.180900776	-0.074888879	0.230983303
123716	126364	10	-	unknown	no_ovlp	SUTs	0.070629753	-0.227596715	-1.553242818	-0.132446826	0.016152049
126364	129004	10	-	SSY5	ORF-T	ORF-T	-0.708188195	-0.419211061	0.309293258	0.012808133	0.687773857
129124	130780	10	-	FBP26	ORF-T	ORF-T	-0.091961576	-0.469475387	0.627040803	0.292790945	-0.187186934
131044	134052	10	-	VPS35	ORF-T	ORF-T	-0.351066661	-0.568467388	-0.143168167	0.031820133	0.112703128
134204	135924	10	-	INO1	ORF-T	ORF-T	-0.320384178	0.125109611	-0.619946406	-1.146875804	2.339744716
135924	136172	10	-	INO1	ORF-T	ORF-T	1.023917719	-0.642328204	-0.092738097	-0.285190639	0.376969499
136308	136828	10	-	SNA3	ORF-T	ORF-T	0.705916213	0.270026941	0.169056859	-0.058426179	-0.263683057
136828	139156	10	-	CUT675	CUTs	CUTs	0.249357586	0.396862689	0.362338532	-0.174820942	0.031807839
139428	139684	10	-	SNR128	other	other	5.06282739	-0.672193153	-0.023020275	-0.31497641	0.281752924
141220	142996	10	-	YJL147C	ORF-T	ORF-T	-0.047304638	-0.186997624	0.247510447	-0.277441649	-0.293751772
142660	142996	10	-	unknown	no_ovlp2	CUTs	2.195564462	0.41320716	0.155009122	-0.019568905	-0.245411783
144484	144868	10	-	SUT624	SUTs	SUTs	0.0601157	-1.350991246	0.196324701	0.65142399	0.490156847
146460	146772	10	-	unknown	no_ovlp	CUTs	1.982472447	-0.192015559	-0.464716812	-0.416165845	0.122152012
149420	150476	10	-	IRC9, YAK1	ORF-T	ORF-T	0.052682562	-0.709557817	0.536851375	0.638053045	-0.325899046
150884	153076	10	-	YUR1	ORF-T	ORF-T	0.199318067	-0.472758159	-0.575811174	-0.277575177	-0.212165244
153284	154756	10	-	TIF2	ORF-T	ORF-T	-0.04320994	-0.19711755	-0.443285639	-0.020233674	-0.426749898
154756	156148	10	-	GLG2	ORF-T	ORF-T	-0.633911588	-0.066117748	1.490925299	0.152784937	2.06709246
156780	157252	10	-	RPS21B	ORF-T	ORF-T	1.048208738	-0.597849582	-0.943505156	-1.207369785	-0.553948993
159452	159868	10	-	YJL133C-A	ORF-T	ORF-T	-0.829843159	-0.542934728	0.879300313	0.370420118	0.515301688
160524	161676	10	-	unknown	no_ovlp	CUTs	1.075043518	-0.025261872	-0.493216352	-1.042768713	0.778834401
164236	165372	10	-	YJL131C	ORF-T	ORF-T	-0.373071346	-0.698152891	0.28704365	-0.040666196	0.207793664
172428	172732	10	-	URA2	ORF-T	ORF-T	1.492266512	-0.806324496	-0.844200458	-0.633935719	-0.250218343
172732	173060	10	-	CUT680	CUTs	CUTs	1.012172998	-0.648973527	0.056513312	-0.363819178	-0.179593666
173548	177596	10	-	TRK1	ORF-T	ORF-T	0.070703435	-0.339475935	-0.447831377	-0.014952574	0.097292485
178004	180316	10	-	PBS2	ORF-T	ORF-T	-0.142638765	-0.400008444	-0.31934651	-0.114441283	-0.693965527
181324	181796	10	-	YJL127C-B	ORF-T	ORF-T	0.269116082	-0.673245699	-0.371869967	-0.094400151	-0.24869787
181796	184292	10	-	SPT10	ORF-T	ORF-T	0.080774016	-0.414455119	-0.371194271	-0.07944522	0.0129506
185532	186724	10	-	GCD14	ORF-T	ORF-T	0.12316862	-0.413449449	-0.154422733	0.041076881	0.612425442
186884	187812	10	-	LSM1	ORF-T	ORF-T	-0.197575099	-0.569211488	-0.277983087	-0.243110311	-0.012487759

187964	189444	10	-	YJL123C	ORF-T	ORF-T	-0.367188883	-0.196438197	0.261783323	0.146369319	0.430861432
189444	190140	10	-	unknown	no_ovlp	SUTs	0.152550711	0.176563001	-0.166810539	-0.287835062	0.06348487
190300	191100	10	-	RPE1	ORF-T	ORF-T	0.095854613	-0.129851812	-0.216522918	-0.356003975	0.140854456
191428	192348	10	-	YJL119C	other	other	0.769539189	0.041467767	-0.094133405	-0.356053805	0.597064948
193692	195292	10	-	NCA3	ORF-T	ORF-T	1.412909103	-0.431167135	1.089261112	0.957113184	-1.187457121
195068	195292	10	-	unknown	no_ovlp2	SUTs	0.8138954	-0.455758019	0.457453112	0.286017747	-0.089991756
195292	196772	10	-	SUT626	SUTs	SUTs	0.012284866	0.278924942	-0.164571577	0.144612796	0.471676765
209820	211660	10	-	GZF3	ORF-T	ORF-T	0.699739413	-0.502006126	-0.725419471	-0.71594013	-0.643842401
211932	217332	10	-	UTP10	ORF-T	ORF-T	0.067660492	-0.328078525	-0.494185633	0.201643127	0.037253347
220092	220684	10	-	PRM10, YJL1	ORF-T	ORF-T	0.681842322	0.258583101	0.304454568	-0.007031963	-0.513703342
226300	227044	10	-	SUT628	SUTs	SUTs	1.047272111	-0.535840015	-0.544751182	-0.658348017	-0.221044924
227916	228468	10	-	SNR37	other	other	-0.135731874	-0.442177474	-0.042463596	-0.069691116	-0.065798429
229620	231044	10	-	GSM1	ORF-T	ORF-T	0.026973673	0.476226622	1.60990423	0.567423209	1.314427684
234172	236500	10	-	GSH1	ORF-T	ORF-T	-0.186261226	-0.726943398	-0.193761747	0.198129254	0.564512658
236716	237028	10	-	unknown	no_ovlp	CUTs	2.147840288	-0.081428847	0.343307986	-0.001171959	-0.96493396
238404	239076	10	-	SUT629	SUTs	SUTs	1.015142229	1.206913197	0.744409349	0.831682789	1.880130388
251692	254468	10	-	KHA1	ORF-T	ORF-T	-0.071213643	-0.038327568	0.056403757	0.408536953	0.314290514
254628	256780	10	-	TOK1	ORF-T	ORF-T	-0.059915219	-0.353114536	0.271169889	0.054951588	0.116642838
260884	262556	10	-	GWT1	ORF-T	ORF-T	-0.708319695	-0.566148951	-0.070339529	0.013238171	0.160347033
262676	265052	10	-	DPB11	ORF-T	ORF-T	0.243768978	-0.447994396	-0.18073742	0.000209976	-0.149506287
267756	268332	10	-	CUT684	CUTs	CUTs	2.460600143	-1.009072993	-0.726598699	-0.20943479	-2.063252802
269804	272556	10	-	TRL1, YJL086	ORF-T	ORF-T	0.053468603	-0.467304965	-0.871581793	0.008326984	-0.231029918
275316	278108	10	-	ALY2	ORF-T	ORF-T	-0.056380075	-0.473595092	-0.123919951	0.051605578	0.040475565
279956	280708	10	-	CUT686	CUTs	CUTs	2.52998332	-0.553848115	-1.175974359	-0.125221429	-0.449058513
283604	285300	10	-	ARP4	ORF-T	ORF-T	0.669696037	0.403049572	-0.43575462	-0.140864502	-0.320351289
285444	289444	10	-	SCP160	ORF-T	ORF-T	-0.598285788	-0.100077945	-0.616537601	-0.060932626	0.061475005
289708	290932	10	-	PRY1	ORF-T	ORF-T	-0.248314062	-0.425259826	0.032185588	0.871375311	0.350709263
292692	294084	10	-	PRY3	ORF-T	ORF-T	-0.169827092	-0.571492686	-0.817885536	-0.491743263	0.152932519
294084	294460	10	-	CUT687	CUTs	CUTs	2.834552189	0.304565785	-0.311829145	0.240501835	0.172090047
299060	302868	10	-	SMC3	ORF-T	ORF-T	-0.955845897	-0.404972852	-0.351017838	-0.138381478	0.728411604
304636	305892	10	-	PSF2	ORF-T	ORF-T	0.075703252	-0.507539242	-0.411759865	-0.408196308	0.020942029
307940	310660	10	-	YJL070C	ORF-T	ORF-T	-0.400035879	-0.334138077	0.67219812	0.256147891	0.519142139
310836	312724	10	-	UTP18	ORF-T	ORF-T	0.582850361	0.113880082	-0.994274361	-0.305666086	0.323877647
312916	315580	10	-	YJL068C	ORF-T	ORF-T	-0.168252069	0.387164363	0.704625045	0.055274446	0.676725625
313956	315580	10	-	unknown	no_ovlp2	SUTs	0.234259129	0.096729584	0.248118368	-0.128120691	-0.045052772
315684	316500	10	-	MRPL8	ORF-T	ORF-T	-0.996255427	-1.034742457	-0.245780558	-0.00515937	-0.458863231
318964	319772	10	-	SUT631	SUTs	SUTs	1.629267164	0.19048824	-1.286102405	-0.727063375	-0.489461174
322212	323124	10	-	unknown	no_ovlp	CUTs	1.688838837	0.340135918	-1.173238626	-0.177081287	0.347906929
326124	328324	10	-	BIT61	ORF-T	ORF-T	0.338162752	0.118459884	-0.12488623	-0.554132776	-0.002752981
327924	328324	10	-	unknown	no_ovlp2	SUTs	-0.532664163	-0.873446786	0.862959355	-0.800811134	-0.743752728
329164	330300	10	-	IKS1	ORF-T	ORF-T	-0.942050545	-0.142461455	1.292015659	0.49740608	0.353080234
330300	333068	10	-	ZAP1	ORF-T	ORF-T	0.320796377	-0.509651129	-1.529316125	-0.607020033	0.355015375
333068	334076	10	-	unknown	no_ovlp	CUTs	1.585528309	-0.008688219	-0.79318479	-0.25816155	0.273869443
337108	337748	10	-	CUT689	CUTs	CUTs	1.327452753	-0.135858318	0.564632476	0.110975242	-0.984132518
340572	342220	10	-	SUT632	SUTs	SUTs	1.052588899	-0.618293855	-0.796707078	-0.205202491	0.073553584
347300	348972	10	-	UBX6	ORF-T	ORF-T	0.001231047	-0.948835484	-0.47987728	-0.045429603	-0.782494665
348972	349220	10	-	YJL047C-A, S	ORF-T	ORF-T	5.042373304	-0.492919946	-0.577728856	-0.507980434	0.098246706
349460	352212	10	-	RTT101	ORF-T	ORF-T	-0.246553209	-0.51394908	-0.067055872	0.082357398	0.07312459
358028	359700	10	-	GYP6	ORF-T	ORF-T	-0.051569534	-0.655716618	-0.195453011	-0.047788981	-0.21221984
364252	365428	10	-	unknown	no_ovlp	CUTs	1.752086821	0.638565565	-1.368402056	-0.508795742	-0.22231061
368388	373972	10	-	NUP192	ORF-T	ORF-T	0.020097883	-0.280104731	-0.366193267	0.143998369	-0.129083552
380220	381044	10	-	TAD2	ORF-T	ORF-T	0.410303127	-0.706753646	-0.615120098	-0.219091674	0.134022045
386268	387340	10	-	BET4	ORF-T	ORF-T	0.207966192	-0.132456348	0.331214453	-0.216938314	-0.25744892
388292	390916	10	-	VPS53	ORF-T	ORF-T	-0.281383596	-0.619170712	0.04336442	-0.048091178	-0.012253669
390916	393756	10	-	unknown	no_ovlp	SUTs	0.289768035	0.408728442	-0.148249604	0.151089657	0.263661304
395668	396484	10	-	APS3	ORF-T	ORF-T	-0.027789648	-1.154061596	-0.226671451	-0.089623624	-0.065533551
397220	398388	10	-	PET130	ORF-T	ORF-T	0.304156096	-0.587505996	0.378935942	0.232442642	-0.254876218
398812	402492	10	-	BBC1	ORF-T	ORF-T	-0.626389319	0.118845718	0.073763574	0.000170416	-0.151120163
404252	405108	10	-	SUT634	SUTs	SUTs	1.252352406	-0.576103726	-0.683427692	-0.091748991	-1.246245594
409364	411020	10	-	MAD3	ORF-T	ORF-T	0.014455719	0.159324994	0.388423385	-0.116767451	0.559320776
411156	413436	10	-	VTC4	ORF-T	ORF-T	-0.557318276	0.510685626	0.288853008	1.409294456	0.870940093
414116	414796	10	-	RPC17	ORF-T	ORF-T	0.749815816	0.119796864	-1.254545994	-1.040690554	0.224190217
417508	419596	10	-	NOP9	ORF-T	ORF-T	0.278592439	-0.559547705	-1.089872149	0.088557972	-0.293872207
419804	421700	10	-	CCT8	ORF-T	ORF-T	0.094151853	-0.213089837	-0.362376422	-0.066717629	-0.003285126
423124	424148	10	-	CTK2	ORF-T	ORF-T	0.235304029	-0.324823204	0.230193278	-0.18792156	0.338920785
431316	432204	10	-	SYS1	ORF-T	ORF-T	-0.356261979	-1.019200683	-0.090269185	-0.016291464	0.039529075
433108	434700	10	-	OST1	ORF-T	ORF-T	-0.388488671	-0.191187231	-0.564192246	-0.11359897	0.124982517
434700	435940	10	-	SUT636	SUTs	SUTs	0.054699498	0.11738605	-0.173243653	-0.136178984	0.259439421
436460	438620	10	-	unknown	no_ovlp	CUTs	1.125280995	0.218515621	-0.968658848	-0.22744315	0.192213416
440932	442548	10	-	YJR003C	ORF-T	ORF-T	0.309260903	-0.584354359	-0.36047602	-0.336262979	0.091335965
442740	445644	10	-	SAG1	ORF-T	ORF-T	-0.09860184	-0.693015128	-0.108934	-0.338214748	0.289469736
450372	450676	10	-	CUT691	CUTs	CUTs	3.252341873	-0.106979098	-1.64008906	-0.100563451	0.713985125
451452	451980	10	-	unknown	no_ovlp	CUTs	1.639568214	0.462281353	-0.220237471	0.662701876	0.671695832
453548	454756	10	-	TDH2	ORF-T	ORF-T	-0.706033344	-0.293264142	-0.249983695	-0.014640158	-0.441639721
457844	458396	10	-	SPC1	ORF-T	ORF-T	0.210378133	-0.716594009	-0.421140867	-0.213346372	-0.526087615
458540	459460	10	-	YJR011C	ORF-T	ORF-T	-0.374757126	-0.843242648	-0.174670197	0.028978518	-0.421975799
459668	460204	10	-	YJR012C	ORF-T	ORF-T	-0.216133462	-0.268842837	0.6718477	-0.208080254	-0.709405398
461292	461588	10	-	CUT692	CUTs	CUTs	3.03674929	-0.30428401	-0.488380242	-0.099537481	0.307377175
461588	462572	10	-	unknown	no_ovlp	CUTs	1.474159215	-0.059780342	-0.259425853	0.602251989	1.696606357
464292	466212	10	-	ILV3	ORF-T	ORF-T	-0.018892794	-0.449369337	-0.511793691	0.099029697	-0.379830323
466212	467052	10	-	ESS1	ORF-T	ORF-T	-0.100815926	0.215386843	0.34691221	0.050134956	-0.09094479
467204	468156	10	-	TES1	ORF-T	ORF-T	-0.311345546	0.05223352	0.517952179	0.128875004	0.891243664

468156	468484	10	-	unknown	no_ovlp2	SUTs	0.10592083	-0.282283139	-0.422614053	-0.455906893	0.305724985
468484	469580	10	-	REC107	ORF-T	ORF-T	-0.044443257	-0.198436909	0.285047862	0.00618345	0.02039403
470188	470988	10	-	MDE1	ORF-T	ORF-T	-0.206892827	-0.289376002	-0.087479724	0.004245247	-0.282628838
470988	471700	10	-	BNA1	ORF-T	ORF-T	0.430520168	-1.158969622	-1.505235573	-0.635672879	-4.530483027
472140	483964	10	-	unknown	no_ovlp	CUTs	2.709464062	-0.036548882	-1.709547143	-1.299386058	-3.245718799
483964	485772	10	-	YJR030C	ORF-T	ORF-T	-0.327325773	-0.197809767	0.212170052	-0.163368827	0.599474443
486452	490876	10	-	GEA1	ORF-T	ORF-T	-0.143102954	-0.395412304	-0.09775268	-0.033624225	0.550901246
492380	496468	10	-	RAV1	ORF-T	ORF-T	-0.438146867	-0.402100396	0.425645279	0.067816445	-0.017773216
496884	497204	10	-	SUT639	SUTs	SUTs	1.031296049	-0.130492054	0.184225793	-0.005697838	-0.026326037
497204	499932	10	-	unknown	no_ovlp	SUTs	0.443539956	0.33215412	0.453251605	-0.074547922	0.115441697
500596	503548	10	-	HUL4	ORF-T	ORF-T	-0.837567593	-0.674195636	1.021263417	0.614878651	1.224303199
510036	513820	10	-	URB2	ORF-T	ORF-T	0.369994024	-0.342838138	-0.490111327	-0.288776611	0.067007801
516316	517540	10	-	POL32	ORF-T	ORF-T	-0.051215168	-0.364090967	-0.483542731	-0.550918194	0.330522997
518588	519252	10	-	VPS55	ORF-T	ORF-T	-0.420596428	-0.372213311	0.362943482	-0.127807746	0.322897803
519404	521732	10	-	SSC1	ORF-T	ORF-T	-0.499300318	-0.149929344	-0.059832719	-0.009615337	-0.207549439
524988	525404	10	-	ANB1	ORF-T	ORF-T	-0.970228061	0.126070502	0.963907978	1.178582298	0.826528399
526708	528484	10	-	UTR1	ORF-T	ORF-T	-0.334858048	-0.119130236	0.076866019	0.102462407	0.144326337
529124	529652	10	-	CUT694	CUTs	CUTs	3.126363236	-0.103024527	-0.918965937	-0.34368625	-0.497295289
534836	535748	10	-	CUT695	CUTs	CUTs	3.048810135	0.191555009	-0.34824848	-0.63571735	0.520052181
541700	542684	10	-	YJR056C	ORF-T	ORF-T	1.040856776	0.273825418	0.357180349	0.009460262	0.31623358
544692	545420	10	-	APS2	ORF-T	ORF-T	-0.160018372	-0.797468013	-0.001845964	-0.189595764	-0.016708455
553380	554860	10	-	NTA1	ORF-T	ORF-T	-0.522701828	-0.905614517	0.221659788	0.009329036	-0.126876137
557636	559188	10	-	ARP3	ORF-T	ORF-T	-0.325069947	0.013344366	-0.045595012	-0.118059948	-0.057688947
566956	567436	10	-	YAE1	ORF-T	ORF-T	-0.124824887	-0.926081585	-0.106870218	-0.173768974	0.158288112
568644	569436	10	-	HAM1	ORF-T	ORF-T	0.41747265	-0.626043769	-1.588697157	-0.273384724	-0.807371482
569508	570676	10	-	LIA1	ORF-T	ORF-T	-0.485517237	-0.733925319	-0.627928426	-0.057584678	0.139037926
570900	572196	10	-	NPA3	ORF-T	ORF-T	0.2595261	0.049895836	0.061636451	0.028893247	0.225095083
572196	572964	10	-	OPI3	ORF-T	ORF-T	-1.04413137	-0.262184434	-0.137314811	-0.217159948	0.517348486
572964	573740	10	-	unknown	no_ovlp	CUTs	1.020993106	-0.23608021	-0.032501859	-0.697354261	-0.132588822
573740	575220	10	-	unknown	no_ovlp	SUTs	-0.294582927	0.20149956	-0.009825497	-0.028821046	0.363459226
575412	576668	10	-	CDC11	ORF-T	ORF-T	-0.244435627	-0.296997775	-0.035549651	-0.043982705	0.341311154
577004	578228	10	-	MIR1	ORF-T	ORF-T	-0.532814748	-0.100453549	0.172840749	-0.031395211	0.174491098
580316	582372	10	-	FMP26	ORF-T	ORF-T	-0.523282394	-0.371869731	0.063650515	0.075723273	-0.413836603
581652	582372	10	-	unknown	no_ovlp2	SUTs	0.102562605	-0.065185506	0.228398773	-0.256177052	0.24332542
582572	583556	10	-	ACF4	ORF-T	ORF-T	-0.241043064	-0.441880151	0.371770741	-0.060753739	0.313017554
585084	585468	10	-	YJR085C	ORF-T	ORF-T	-0.279521368	-0.282904264	0.494861925	0.036585304	0.419205895
586220	587412	10	-	YJR088C	ORF-T	ORF-T	-0.424761073	-0.524854344	0.12135649	-0.026866181	0.02155115
590804	594604	10	-	GRR1	ORF-T	ORF-T	-0.058084868	-0.290519327	-0.121426356	-0.048632808	-0.163887797
594836	598348	10	-	JSN1	ORF-T	ORF-T	0.073515173	-0.283369502	0.210385963	0.008638673	-0.378057623
603116	604252	10	-	FIP1	ORF-T	ORF-T	-0.008297912	-0.744972609	-0.053896129	-0.008128534	-0.214208236
604252	606004	10	-	IME1	ORF-T	ORF-T	-0.210415274	1.004037403	2.516669525	1.723925776	-0.35004873
606004	607308	10	-	SUT643	SUTs	SUTs	0.968102878	1.038963587	0.308062019	0.542928508	0.151312883
611252	612092	10	-	SUT644	SUTs	SUTs	1.825897833	-0.391422196	-0.641963777	-0.209452101	-0.106575888
613020	615212	10	-	YJR098C	ORF-T	ORF-T	0.189010356	-0.431255999	0.254465174	-0.308634342	-0.049466673
616300	617340	10	-	YJR100C	ORF-T	ORF-T	0.463481217	0.062194315	0.238750856	-0.160977014	-0.348734261
618996	620372	10	-	VPS25	ORF-T	ORF-T	0.190759647	-0.678730989	-0.018127812	-0.555605786	-0.528535917
619900	620372	10	-	unknown	no_ovlp2	CUTs	2.086046044	-0.374040073	-0.461515909	-0.050414133	-0.4928472
622468	623052	10	-	SOD1	ORF-T	ORF-T	-0.376581931	-0.30149006	0.020755791	-0.036174247	0.168031144
623052	623316	10	-	SUT645	SUTs	SUTs	-0.596753884	-0.797015421	-0.502594702	-0.676048239	-0.943818274
623316	624276	10	-	unknown	no_ovlp	SUTs	0.456481416	0.066836344	-0.63586621	0.056147737	-0.366553352
627564	628724	10	-	SUT646	SUTs	SUTs	1.006517826	-0.216158914	0.046904791	0.205911145	-0.330749417
629500	632980	10	-	CPA2	ORF-T	ORF-T	-0.125663558	-0.989905777	-1.017198844	-0.035882706	-0.957409153
635740	636820	10	-	YJR111C	ORF-T	ORF-T	0.077640317	-0.5996367	-0.045509842	-0.097611934	-0.12957582
638132	638996	10	-	RSM7	ORF-T	ORF-T	-0.905757959	-0.424061665	0.414323428	0.43208157	-0.22903303
639972	640412	10	-	CUT698	CUTs	CUTs	2.506251173	-0.181848909	-0.480713642	-0.9294823	0.014449876
640412	641796	10	-	SUT648	SUTs	SUTs	0.153532887	0.406680438	1.141585373	0.620817335	0.381614197
643372	644148	10	-	ILM1	ORF-T	ORF-T	-0.254344032	-0.785104604	-0.321336837	0.128137175	-0.165936813
644524	646564	10	-	JHD2	ORF-T	ORF-T	0.378572922	-0.065402582	0.494874698	0.621859581	0.239846908
650684	651252	10	-	CUT699	CUTs	CUTs	2.907590717	0.480657959	-0.735534219	0.186434611	0.111926425
652700	654268	10	-	YJR124C	ORF-T	ORF-T	0.366946913	-0.849250974	-1.211069183	-0.534013681	-0.429592953
654636	656012	10	-	ENT3	ORF-T	ORF-T	-0.042143178	-0.284142069	0.042323931	-0.064506808	0.01857812
656172	658668	10	-	VPS70	ORF-T	ORF-T	-0.565145264	-0.375849785	-0.071144496	0.144722231	0.203012652
660932	662828	10	-	RSF2	ORF-T	ORF-T	-0.194138222	-0.521889536	0.052841459	0.606713993	-0.086698762
662828	663756	10	-	unknown	no_ovlp2	SUTs	0.094812814	0.729643037	0.39068401	0.254234682	0.108875768
663756	665116	10	-	YJR129C	ORF-T	ORF-T	-1.326531504	0.386738432	1.034712003	0.611000708	0.829724537
665116	667188	10	-	STR2	ORF-T	ORF-T	0.343793567	0.043101776	-0.169328149	-0.275090318	-0.711725795
673628	675876	10	-	SGM1	ORF-T	ORF-T	-0.167061537	-0.843204122	-0.525341138	-0.194726227	-0.148302762
675876	676108	10	-	unknown	no_ovlp2	SUTs	0.261140076	-0.88838119	-0.359114817	-0.520941921	-0.169192524
676108	676812	10	-	MCM22	ORF-T	ORF-T	0.248001278	-0.425368207	0.028492537	-0.450421856	0.12954409
677348	678700	10	-	YJR136C	ORF-T	ORF-T	0.610767974	-0.256927621	-0.329511184	-0.442583404	-0.40516241
678876	683396	10	-	ECM17	ORF-T	ORF-T	-0.376494584	-0.108771483	-0.116354587	0.139466427	0.283029918
689356	690596	10	-	HOM6	ORF-T	ORF-T	-0.303120787	-0.695494162	-0.535834008	-0.05976264	-0.197226129
691468	695764	10	-	HIR3	ORF-T	ORF-T	-0.029918415	-0.099504915	-0.424943877	-0.290205603	-0.167179929
698220	700684	10	-	PMT4	ORF-T	ORF-T	-0.46007139	-0.410775671	-0.426900437	0.030439386	0.47538054
702788	703060	10	-	RPS4A	ORF-T	ORF-T	0.46273579	-1.24342986	-1.504236472	-1.225868155	-0.390799033
703516	705236	10	-	SUT650	SUTs	SUTs	2.007467692	-0.695627404	-0.805680269	-0.049843515	-0.60593635
712156	715828	10	-	DAN4	ORF-T	ORF-T	-0.384593352	-0.098178787	0.734687174	0.405649401	-1.777956962
718612	719196	10	-	unknown	no_ovlp	SUTs	0.815847751	-1.12651485	0.184451118	0.144611502	-0.058643201
23049	23849	10	+	YJL217W	ORF-T	ORF-T	-0.162670504	-0.379185517	0.750227168	0.787792021	-0.757469375
28473	29321	10	+	HXT8	ORF-T	ORF-T	0.394640389	-0.6626071	0.31422302	0.246881094	-1.813938464
31481	31825	10	+	unknown	no_ovlp2	CUTs	1.79909851	0.514037379	-0.383901358	-0.558224097	-1.590116665
31825	33393	10	+	YJL213W	ORF-T	ORF-T	0.645258653	0.243288576	0.966517366	1.545613343	-2.158604793

36481	36881	10	+	PEX2	ORF-T	ORF-T	0.276269577	0.571078471	0.209901703	-0.188210538	0.087100063
37913	39393	10	+	CBP1	ORF-T	ORF-T	-0.166805489	-0.739911146	-0.264252081	-0.410524152	0.123492608
47721	48265	10	+	unknown	no_ovlp	CUTs	2.133567175	0.746302172	-0.25267239	-0.020014703	0.029810604
53337	54617	10	+	PRP21	ORF-T	ORF-T	0.210975785	0.05077587	-0.136892608	-0.278057367	0.384760019
54265	54617	10	+	unknown	no_ovlp2	SUTs	-0.504914498	-0.620003939	0.363416006	-0.090292137	0.849563853
54617	56249	10	+	ECM25	ORF-T	ORF-T	-0.202605159	-0.443903081	-0.016492292	-0.022494774	0.110844351
60809	63577	10	+	PHO90	ORF-T	ORF-T	-0.502131438	-0.185618568	-0.230737767	0.345473779	0.662109546
63769	67601	10	+	UBP12	ORF-T	ORF-T	-0.052886962	-0.344533294	-0.556916398	-0.329201617	0.037643858
69305	70961	10	+	CDC6	ORF-T	ORF-T	0.059239422	-0.097019398	-1.298844318	-0.768831214	0.177286512
71377	71961	10	+	YJL193W	ORF-T	ORF-T	0.482166008	-0.697759001	-1.147291588	-0.807321613	-0.314049525
73761	74737	10	+	RPS14B	ORF-T	ORF-T	2.554773169	-0.83089732	-0.224624834	-0.30151291	0.103934622
75937	76336	10	+	RPL39	ORF-T	ORF-T	0.740733212	-0.960974284	-0.622309964	-1.143788607	-0.309628299
80137	82009	10	+	MNN5	ORF-T	ORF-T	-0.115600676	-0.438123698	-0.39036218	0.232709418	0.417890358
83417	83849	10	+	GON7	ORF-T	ORF-T	0.275707641	0.51205013	0.362143752	-0.21965239	0.261660006
84017	85425	10	+	MNN11	ORF-T	ORF-T	-0.64765942	-0.368725985	-0.29735442	-0.082203454	0.541858261
86073	87489	10	+	YJL181W	ORF-T	ORF-T	-0.601646927	-0.317886544	-0.069265734	0.011410101	0.558368616
87865	88745	10	+	unknown	no_ovlp	CUTs	1.974080087	0.475659964	-0.25765092	-0.651200899	0.274771941
88745	88977	10	+	PFD1	ORF-T	ORF-T	0.326055924	-0.249647125	-0.198368376	-0.321430284	0.189376536
90737	91097	10	+	RPL17B	ORF-T	ORF-T	-0.625751575	-0.29730118	-0.058441779	0.063667866	0.198514654
94241	94969	10	+	unknown	no_ovlp	SUTs	0.123263617	0.670435485	0.112091472	-0.038334606	0.530440716
94969	96097	10	+	KRE9	ORF-T	ORF-T	-0.697883421	-0.846357194	0.149926186	0.308226547	0.249171724
96705	96985	10	+	unknown	no_ovlp	CUTs	1.256268015	0.396427899	0.857502841	-0.097674845	1.2268569
97697	98937	10	+	CPS1	ORF-T	ORF-T	-0.33764658	0.004801395	-0.136970498	0.073664428	0.42098642
101193	101938	10	+	unknown	no_ovlp	CUTs	2.689358867	-0.243190892	-0.799567556	-0.395136485	-0.464862975
104962	106186	10	+	ERG20	ORF-T	ORF-T	-0.011699793	-0.204853904	-0.29055676	-0.065452038	-0.387608132
106338	106898	10	+	QCR8	ORF-T	ORF-T	-0.364915615	-0.450988294	0.72411576	0.222620737	0.095876751
109826	110506	10	+	CUT197	CUTs	CUTs	2.689167524	-0.836077646	-0.79927448	-0.076653985	-0.117392692
116690	117138	10	+	unknown	no_ovlp2	CUTs	2.445993314	0.076471518	-0.077714942	-0.242416106	-0.554293525
117138	118082	10	+	FMP33	ORF-T	ORF-T	-0.323968868	-0.659562196	1.658738626	0.605568732	-0.418129303
119506	120402	10	+	unknown	no_ovlp	CUTs	1.505319833	0.558803994	-0.395452542	0.137366516	-1.379188311
120402	121992	10	+	HSP150	ORF-T	ORF-T	-0.322356486	-0.145501307	0.110739619	0.010370975	0.021389881
123808	125536	10	+	SUT204	SUTs	SUTs	1.66047935	0.284341932	0.017013286	-0.16983353	0.038307637
126768	128392	10	+	unknown	no_ovlp	CUTs	1.441439367	-0.618767714	-0.826354615	-0.183683465	-0.068645061
131136	131720	10	+	unknown	no_ovlp	SUTs	0.08436731	-0.073984035	1.176389226	0.892731274	0.755011421
134216	134704	10	+	CUT200	CUTs	CUTs	3.113008669	0.640031789	-0.57937775	0.020734722	-0.080463372
136792	137216	10	+	unknown	no_ovlp	SUTs	-1.092129652	-1.51190158	0.721931828	-0.544231873	0.287394148
137344	139408	10	+	YJL150W, YJL	ORF-T	ORF-T	0.134158359	-0.275171771	0.214639588	-0.1524442	0.203293178
140376	141360	10	+	RPA34	ORF-T	ORF-T	-0.01363337	-0.709772965	-0.214855567	-0.082112864	0.718208138
143184	144944	10	+	IDS2	ORF-T	ORF-T	-0.019304103	-0.161484054	-0.000613922	0.112362581	0.438324941
145096	146128	10	+	SFH5	ORF-T	ORF-T	-0.249869261	-0.863687902	-0.301919215	-0.207150845	-0.04847294
146352	146880	10	+	YJL144W	ORF-T	ORF-T	0.610806527	0.293854193	1.639132315	0.861003281	0.180211396
147064	147872	10	+	TIM17	ORF-T	ORF-T	-0.733563968	-0.737904958	-0.170059096	0.021983111	0.38542172
150208	150864	10	+	unknown	no_ovlp	SUTs	-0.098671289	0.376402669	-0.018777214	-0.027384232	0.180850136
150864	151696	10	+	RPB4	ORF-T	ORF-T	-0.177382022	-0.901492898	-0.134808863	0.169886229	0.10502428
155000	155912	10	+	CUT204	CUTs	CUTs	1.99229228	0.663755636	-0.949952377	-0.384649171	-0.266118395
158144	159520	10	+	YJL135W, LCI	ORF-T	ORF-T	-0.390026658	-0.028886545	-0.216992652	-0.05088051	0.63071521
160464	161704	10	+	MRS3	ORF-T	ORF-T	-0.08706009	-0.851553657	-0.377234322	-0.078331783	-0.484777705
161920	164216	10	+	YJL132W	ORF-T	ORF-T	0.406379425	0.22090068	0.521111144	-0.057749728	0.572374873
173600	174032	10	+	unknown	no_ovlp	CUTs	1.782235734	-0.000661462	0.496894777	1.142399592	1.215144964
177824	178368	10	+	unknown	no_ovlp	SUTs	0.4019337	-0.65387097	0.145512771	0.822077286	1.07041441
180544	183208	10	+	CUT206	CUTs	CUTs	2.286389812	-0.030633307	-0.151003394	0.00533901	-0.649026847
184464	185544	10	+	NIT2	ORF-T	ORF-T	0.379173794	-0.060383219	0.176291193	-0.066788176	-0.038451655
186912	187240	10	+	CUT207	CUTs	CUTs	3.025901556	0.416429143	-1.207344037	-0.292039928	0.256225961
189696	190400	10	+	ALB1	ORF-T	ORF-T	0.224742784	-0.338285401	-0.687888079	-0.184727639	0.237812327
191680	192488	10	+	YJL118W	ORF-T	ORF-T	0.14091161	0.343648005	-0.199490714	-0.523808014	1.042972175
192488	193632	10	+	PHO86	ORF-T	ORF-T	-0.054089218	0.332966892	-0.127736904	0.586468254	0.245036914
196216	197160	10	+	ASF1	ORF-T	ORF-T	-0.08418483	-0.174527805	-0.629078603	-0.156002272	0.245086862
204960	205296	10	+	tD(GUC)J1	other	other	0.221310567	-0.104668183	1.269587912	0.642792397	0.39597559
205296	207592	10	+	MDV1	ORF-T	ORF-T	0.222346954	-0.417706754	-0.045836808	-0.112053242	-0.052945464
207832	209688	10	+	CCT7	ORF-T	ORF-T	-0.128270263	-0.58038997	-0.599938521	-0.157292158	-0.196095076
211976	212992	10	+	unknown	no_ovlp	SUTs	0.416638488	0.400623261	0.008651406	-0.08773069	-0.012818447
220536	223448	10	+	IME2	ORF-T	ORF-T	0.167090598	-0.392402527	0.233149138	0.137901543	1.629074036
223448	227304	10	+	SET4	ORF-T	ORF-T	-0.153002156	0.67228904	0.255935382	0.080839294	0.032639586
226704	227304	10	+	unknown	no_ovlp2	SUTs	-0.004725725	0.464501431	0.107915136	-0.097697564	0.101779093
227304	227960	10	+	PAM16	ORF-T	ORF-T	-0.694133007	-0.687119932	-0.045309899	0.054094784	0.245697423
231288	233848	10	+	MEF2	ORF-T	ORF-T	-0.19262361	-0.058394321	0.738237649	0.041067411	1.277199346
236840	239160	10	+	LSB6	ORF-T	ORF-T	0.345189511	-0.176016484	0.107948386	0.087115889	-0.460762138
239376	241896	10	+	unknown	no_ovlp2	SUTs	0.820290857	0.130666291	-0.174859014	-0.048011174	-0.033218029
241896	245328	10	+	SAP185	ORF-T	ORF-T	0.22928298	-0.560972996	-0.83274198	-0.267655584	0.551180701
245544	246320	10	+	PHS1	ORF-T	ORF-T	0.128312575	-0.077604412	0.127469398	0.081301363	0.411901916
246456	247040	10	+	MRPL49	ORF-T	ORF-T	-1.266010875	-1.05145221	0.674281375	0.086866628	0.542179612
247152	251752	10	+	BCK1	ORF-T	ORF-T	-0.3446894	-0.221186775	-0.18396125	-0.226057889	0.116969385
257232	261008	10	+	SRS2	ORF-T	ORF-T	-0.491801768	-0.536421024	-0.128366345	-0.015633346	0.625053722
265856	268776	10	+	SIP4	ORF-T	ORF-T	-0.728550784	0.107068023	2.241940817	1.665390062	3.783173534
268776	269896	10	+	ARG3	ORF-T	ORF-T	-1.842266305	-1.019314521	-0.255308669	-0.046766517	0.256978364
272752	274808	10	+	EXO70	ORF-T	ORF-T	-0.092356625	-0.751224216	-0.296535916	-0.283240322	-0.085056388
278800	281088	10	+	TAX4	ORF-T	ORF-T	-0.826987809	-0.587433706	0.323139092	0.393640171	0.721053598
281088	283584	10	+	IML2	ORF-T	ORF-T	-0.056743008	-0.855536976	-0.566978304	-0.016680976	-1.100563329
289688	292016	10	+	SUT209	SUTs	SUTs	0.585717307	0.053886487	-0.130991188	-0.677464908	-0.109358288
292016	295048	10	+	YJL077W-B, Y	other	other	0.357482625	0.147110401	0.062255043	0.082642381	0.127043761
295184	298640	10	+	NET1	ORF-T	ORF-T	-0.125505078	-0.178279373	-0.557258113	0.100867172	-0.255687223
303088	304200	10	+	JEM1	ORF-T	ORF-T	-0.783518771	0.064354444	0.113835187	-0.309089971	0.533872193

306088	307968	10	+	ARG2	ORF-T	ORF-T	-0.085497362	-0.057241476	-0.034429161	-0.490256992	0.57242764
310928	314136	10	+	unknown	no_ovlp	SUTs	0.106038994	0.4245076	-0.061914908	-0.102740989	0.04922641
314136	316696	10	+	unknown	no_ovlp	CUTs	1.147156093	0.347080153	-0.257750396	0.353158407	1.049726443
316696	317056	10	+	YJL062W-A	ORF-T	ORF-T	-0.693130913	-0.185093439	0.586143135	0.069702363	0.595089296
317056	319864	10	+	LAS21	ORF-T	ORF-T	-0.738587475	-0.345283557	-0.192827031	-0.320542348	0.352691506
319984	322424	10	+	NUP82	ORF-T	ORF-T	0.098178046	-0.55022506	-0.464895926	-0.234073609	-0.170620658
323352	324816	10	+	BNA3	ORF-T	ORF-T	-0.333076188	-0.165803315	0.074396528	-0.365548699	0.468336735
324936	326272	10	+	YHC3	ORF-T	ORF-T	-0.00109174	-0.48160007	0.078167733	0.046557655	0.253778756
329808	330240	10	+	unknown	no_ovlp	CUTs	1.382320322	-0.244442525	-0.711782273	-0.366731473	-0.91518511
333024	333336	10	+	unknown	no_ovlp	SUTs	-0.065753526	0.31392638	0.685176611	-0.115942098	0.236105985
333336	334152	10	+	YJL055W	ORF-T	ORF-T	0.060096895	-0.481615822	-0.354381843	-0.262814218	-0.588270789
334264	337192	10	+	TIM54	ORF-T	ORF-T	-0.051782878	-0.409572394	-0.511072756	-0.161424678	-0.396814429
335800	337192	10	+	unknown	no_ovlp2	SUTs	0.122464128	-0.461355485	-0.172699268	-0.213046779	-0.184704041
337992	338200	10	+	CUT210	CUTs	CUTs	1.896132358	0.225584268	0.018570671	-0.005732816	0.05807733
338200	339416	10	+	TDH1	ORF-T	ORF-T	-0.669009904	-0.735860924	0.072825295	0.443301538	-2.0015217
339720	342472	10	+	IRC8	ORF-T	ORF-T	-0.343505957	-0.317269398	-0.270133846	-0.160446311	0.915259396
342472	345760	10	+	MTR4	ORF-T	ORF-T	0.005347162	-0.33180348	-0.920029424	0.146038584	0.114161351
345952	347384	10	+	YJL049W	ORF-T	ORF-T	-0.158311825	-0.843952549	-0.020200948	-0.11628562	-0.514802774
349144	350088	10	+	CUT211	CUTs	CUTs	2.0577101	-0.689051885	-0.287421284	0.122511877	-0.30232154
352368	353808	10	+	YJL046W	ORF-T	ORF-T	-0.096570635	-0.305518202	-0.012587985	-0.195565863	-0.40497522
353808	355744	10	+	unknown	no_ovlp2	SUTs	-0.3199355	0.200937953	0.268067422	0.1316666	0.084856892
355744	361152	10	+	YJL045W	ORF-T	ORF-T	-0.067447331	0.472215883	1.793651364	0.121894578	-0.081071793
358120	361152	10	+	unknown	no_ovlp2	SUTs	0.38390866	0.452559407	-0.109907383	0.022967672	-0.24994435
361152	363224	10	+	MHP1	ORF-T	ORF-T	-0.578075908	-0.24109073	0.210297639	0.404442575	0.309314238
365904	367392	10	+	NSP1	ORF-T	ORF-T	0.166884268	0.021252065	0.018355679	-0.065815806	0.119783882
375048	375848	10	+	SUT210	SUTs	SUTs	-0.308430747	1.283522046	2.575581945	0.461773993	-1.571093726
378704	380248	10	+	SNX4	ORF-T	ORF-T	-0.014627275	-0.060648351	0.718839605	0.118475212	0.008136989
381288	383576	10	+	KAR2	ORF-T	ORF-T	-0.819259832	-0.387337988	-0.153804877	-0.253178914	-0.127476238
383808	386224	10	+	HCA4	ORF-T	ORF-T	0.140389831	-0.391571524	-0.520897068	0.236936336	0.626223497
387624	388424	10	+	MAD2	ORF-T	ORF-T	-0.09124334	-0.300186254	0.423571155	-0.341861313	-0.006869057
392344	393736	10	+	RNR2	ORF-T	ORF-T	0.078424783	0.328322418	0.080571502	0.349704652	-0.045985661
393928	395568	10	+	RRN7	ORF-T	ORF-T	0.03464428	-0.315859092	0.239956544	-0.303426757	1.297252976
402808	403400	10	+	MPS3, YJL01(	other	other	-0.720150873	-0.245535964	-0.248137926	-0.455565326	0.912393367
407504	409320	10	+	CCT3	ORF-T	ORF-T	-0.260724009	-0.504261811	-0.40078191	-0.003738326	-0.194202165
413656	414064	10	+	CUT213	CUTs	CUTs	1.860722249	0.587988026	0.380785381	0.77810392	0.142270346
421928	422432	10	+	YJLWtau4, YJ	other	other	0.800078008	-0.051252315	0.592614096	-0.303608509	-0.190356248
425024	428512	10	+	CYR1	ORF-T	ORF-T	-0.006020184	-0.236407133	-0.159492461	-0.10128189	-0.059647444
432600	433192	10	+	COX16	ORF-T	ORF-T	-0.824388443	-0.894267913	0.385201376	0.198321562	-0.039134504
435344	436064	10	+	PRE3	ORF-T	ORF-T	-0.056226158	-0.547281623	0.228204491	-0.311932627	-0.169092175
436160	436744	10	+	unknown	no_ovlp	SUTs	-0.357032917	-0.38080152	0.161336945	0.243388959	-0.191707703
436744	438720	10	+	AVT1	ORF-T	ORF-T	-0.299397884	-0.406204471	-0.01307478	-0.024162932	-0.062321113
438840	440976	10	+	MPP10	ORF-T	ORF-T	-0.025603939	-0.520594587	-0.542216029	0.081987095	0.247574319
445872	448080	10	+	APL1	ORF-T	ORF-T	-0.013653099	-0.51749567	-0.060157155	-0.081599407	-0.135148306
449200	450704	10	+	POL31	ORF-T	ORF-T	-0.109151447	-0.111691575	0.221980073	-0.188824674	0.492138814
450904	452096	10	+	SUI2	ORF-T	ORF-T	-0.271421972	-0.421110902	-0.505967827	-0.041146133	-0.459599167
452312	453624	10	+	YJR008W	ORF-T	ORF-T	0.05607284	-0.491297678	-0.006773129	0.351151115	-0.044700657
456040	456784	10	+	MET3	ORF-T	ORF-T	0.309524318	-0.693521412	-0.316379753	-0.112358863	-0.223751057
458584	458816	10	+	CUT214	CUTs	CUTs	2.873686491	0.231764608	0.684277069	0.303295181	0.238477395
459640	460376	10	+	unknown	no_ovlp	SUTs	0.31280589	-0.070820912	-0.336946537	0.489278557	-0.591540275
460376	461616	10	+	GPI14	ORF-T	ORF-T	-0.56006728	-0.4392984	0.244089922	0.012240246	-0.582485281
461776	462528	10	+	TMA22	ORF-T	ORF-T	0.003973524	-0.583152928	-0.470277457	-0.317927304	-0.409674892
463040	463640	10	+	YJR015W	ORF-T	ORF-T	-0.330215709	-0.286927644	-0.453610486	0.231797096	0.740254259
466512	467064	10	+	YJR018W, YJF	other	other	2.466894457	-1.059723716	-0.920856712	-0.472018472	-0.710828898
469752	470224	10	+	LSM8	ORF-T	ORF-T	-0.25836681	-1.170884088	0.25304803	-0.344899139	0.561045564
472168	485664	10	+	unknown	no_ovlp	CUTs	1.196056573	0.222070869	-0.648167775	-0.701197016	-0.761961638
491056	492384	10	+	CPR7	ORF-T	ORF-T	-0.19913384	-0.556996959	-0.370561734	-0.285846979	0.439018622
496608	497152	10	+	PET191	ORF-T	ORF-T	-0.626335011	-0.79995024	0.097317513	-0.378989964	0.111346474
497464	500744	10	+	RAD26	ORF-T	ORF-T	-0.255012125	-0.180490991	0.085012789	-0.030928655	-0.331285669
503904	504848	10	+	YJR039W	ORF-T	ORF-T	-0.194745663	-0.68786044	0.683936812	0.465138491	-0.198081005
507696	510144	10	+	GEF1	ORF-T	ORF-T	-0.253646351	-0.33369977	-0.705478676	-0.313891291	-0.860574847
514032	516400	10	+	NUP85	ORF-T	ORF-T	-0.192099554	-0.614246324	-0.259986454	-0.030744004	-0.030250609
522024	523928	10	+	TAH11	ORF-T	ORF-T	0.221805298	-0.049433129	-0.198198226	-0.148230397	0.469868643
526224	526848	10	+	CYC1	ORF-T	ORF-T	-0.170655681	-0.468298865	0.551999515	0.197539541	0.22788906
528680	529672	10	+	ISY1	ORF-T	ORF-T	-0.224991927	-0.58770965	0.306864148	0.176783884	0.29346486
529840	531432	10	+	OSM1	ORF-T	ORF-T	-0.095650571	-0.732146546	0.10374893	0.015496041	0.086279172
532008	533792	10	+	RAD7	ORF-T	ORF-T	-0.242443089	-0.506225433	0.18702564	0.035429321	0.066758732
533792	535984	10	+	BFA1	ORF-T	ORF-T	-0.262523483	0.145535229	0.080759056	-0.186087957	1.582240239
535984	537656	10	+	YJR054W	ORF-T	ORF-T	0.102123476	-0.254281046	-0.867820542	-0.828672524	0.265459021
538768	539672	10	+	HIT1	ORF-T	ORF-T	0.152757649	-0.557805568	-0.403714803	0.127727636	0.199651233
544056	544768	10	+	CDC8	ORF-T	ORF-T	-0.302423939	-0.6525738	-0.091198987	-0.532979018	0.296210074
545712	548424	10	+	PTK2	ORF-T	ORF-T	-0.413897948	-0.924306815	-0.394137104	0.365328685	-0.478862785
548664	550424	10	+	CBF1	ORF-T	ORF-T	-0.247515228	-0.815816794	-0.408531082	-0.005773035	-0.121657592
550424	553472	10	+	YJR061W	ORF-T	ORF-T	0.153496	0.35473162	0.319254042	0.701150461	0.247441098
555152	555720	10	+	RPA12	ORF-T	ORF-T	0.282536625	-0.499301901	-1.238319484	-0.534988767	0.030562635
555880	557656	10	+	CCT5	ORF-T	ORF-T	0.144520529	-0.359536149	-0.653640988	-0.129117627	-0.257587156
559392	566936	10	+	TOR1	ORF-T	ORF-T	-0.111637184	-0.332705503	-0.319625407	0.040910844	-0.110267919
567608	568768	10	+	RFC2	ORF-T	ORF-T	-0.035655114	-0.640952928	-0.128803579	-0.245423882	0.03339009
570864	571544	10	+	unknown	no_ovlp	CUTs	2.014083385	0.632747283	-1.406062739	-0.294479291	0.481785683
573184	573848	10	+	MOG1	ORF-T	ORF-T	-0.779957014	-0.600421993	0.126744894	0.173239873	0.319284551
573928	575360	10	+	HOC1	ORF-T	ORF-T	0.288631846	-0.053860253	-0.32737753	-0.207485076	-0.127728984
576920	578928	10	+	unknown	no_ovlp	SUTs	0.288220746	0.346643974	-0.146639746	0.126131995	0.132474649
578928	580856	10	+	BNA2, YJR07	other	other	0.899809705	2.549556378	1.352500096	-1.556724972	-1.67334841

580280	580856	10 +	unknown	no_ovlp2	CUTs	1.131893237	0.236658135	0.899877001	0.12776053	-1.456015725
581720	583000	10 +	CUT216	CUTs	CUTs	2.176265331	0.328750858	-0.451868045	-0.133440786	-0.499947258
583712	585144	10 +	CSN12	ORF-T	ORF-T	-0.043845219	-0.531240532	-0.292908316	-0.266549529	-0.62455921
586040	586664	10 +	unknown	no_ovlp	SUTs	-0.028225807	-1.488262506	-0.251493342	0.023079365	-0.414831755
587656	590632	10 +	BIR1	ORF-T	ORF-T	-0.02598834	-0.111377505	-0.273820612	-0.30948075	-0.208982711
598728	603200	10 +	BUD4	ORF-T	ORF-T	0.028373389	0.019843571	-0.550499453	-0.083476957	0.234974898
608232	608591	10 +	RPL43B	ORF-T	ORF-T	0.007821518	-1.079791821	-1.114983994	-0.78489256	-0.067285836
609472	611072	10 +	SFC1	ORF-T	ORF-T	-0.315832095	0.659245083	2.47303513	0.23693636	0.353090702
611072	612104	10 +	YJR096W	ORF-T	ORF-T	0.023749547	0.395509059	1.48138922	0.857598889	-0.03264825
612344	613592	10 +	JJJ3	ORF-T	ORF-T	0.340933471	-0.121973974	-0.671212178	-0.376534905	1.224830993
613040	613592	10 +	unknown	no_ovlp2	CUTs	1.654783614	-0.155671468	0.481279466	-0.582905909	0.614024997
615488	616368	10 +	YUH1	ORF-T	ORF-T	0.072964245	-0.236155923	0.036167462	-0.31429813	-0.377982969
617496	617840	10 +	unknown	no_ovlp	CUTs	1.871377564	-0.317459634	0.792015638	0.161949471	-0.10284902
618144	619176	10 +	RSM26	ORF-T	ORF-T	-0.913773184	-1.240117758	-0.249277858	-0.239422749	0.08469591
620568	622560	10 +	URA8	ORF-T	ORF-T	0.763869514	-0.406291741	-0.478434893	-0.021201891	-0.138558362
623184	623552	10 +	SUT217	SUTs	SUTs	0.502389544	0.8796832	-0.160020346	-0.193518933	-0.27690635
623552	624648	10 +	ADO1	ORF-T	ORF-T	-0.221023715	-0.073683245	-0.551819717	-0.156717012	0.1305215
624848	626168	10 +	ECM27, YJR1	other	other	0.006830117	-0.035145002	-0.19547	0.08922606	-0.086579996
628904	629328	10 +	ABM1	ORF-T	ORF-T	1.152221215	-0.879383864	0.077591964	0.122053986	-0.250750927
633512	634520	10 +	YMR1	ORF-T	ORF-T	-0.669362301	-0.572766556	0.238579454	-0.167511936	0.540118705
637024	637616	10 +	NNF1	ORF-T	ORF-T	-0.020195592	-0.250683283	0.015741223	-0.374582642	1.115400318
637776	638272	10 +	YJR112W-A	ORF-T	ORF-T	0.597245011	0.212180819	0.418654024	-0.266145234	0.17078479
639440	640048	10 +	YJR115W, YJF	ORF-T	ORF-T	-0.722146604	-0.877810501	-0.557956374	-0.730948211	-1.062031141
641984	642864	10 +	STE24	ORF-T	ORF-T	-0.113710824	-0.491827532	-0.120233428	-0.270114859	0.052304891
647400	649440	10 +	YJR120W, AT	ORF-T	ORF-T	-0.440781911	-0.116818272	0.255110354	0.06143822	0.128776671
649504	649752	10 +	unknown	no_ovlp	SUTs	-0.122168737	0.203102896	0.247419191	-0.004604412	0.143242933
649752	651328	10 +	CAF17	ORF-T	ORF-T	-1.459951017	-0.747897825	0.528690145	-0.15805832	0.851588734
651848	652696	10 +	RPS5	ORF-T	ORF-T	-0.484448921	0.010575761	-0.030843202	-0.046249622	-0.044720915
658888	659608	10 +	unknown	no_ovlp	CUTs	1.020169717	-0.280617177	-0.486989181	-0.695888958	0.355276145
663752	664472	10 +	CUT221	CUTs	CUTs	6.291550913	-0.460992823	-1.104502578	-0.387422479	-0.356803327
664992	666552	10 +	unknown	no_ovlp	CUTs	1.441124024	-0.184676545	-0.443001334	-0.255691443	0.777744579
667632	669464	10 +	MNS1	ORF-T	ORF-T	-0.357167771	-0.461137292	-0.508251868	-0.008617352	-0.102213906
669464	673704	10 +	NMD5	ORF-T	ORF-T	0.348503251	-0.567013162	-1.053320577	-0.098498937	-0.226055649
672896	673704	10 +	unknown	no_ovlp2	SUTs	-0.377639574	-0.616481226	-0.401954385	-0.382532897	-0.06705218
676960	677432	10 +	TIM8	ORF-T	ORF-T	-0.086515023	-0.653454308	-0.091484044	0.0221067	0.06593503
684560	689440	10 +	IML1	ORF-T	ORF-T	0.13925538	-0.147502397	-0.225292029	-0.514599912	0.247238531
695896	697208	10 +	YJR141W	ORF-T	ORF-T	0.278620593	-0.09688128	-0.424231595	-0.297236098	0.053633582
697208	698272	10 +	YJR142W	ORF-T	ORF-T	0.227636958	-0.306457974	-0.015757493	-0.416480404	-0.517890657
700856	701776	10 +	MGM101	ORF-T	ORF-T	-0.070206252	-0.187606687	0.127225365	-0.096062444	0.605593336
704120	707176	10 +	YJR146W, HN	ORF-T	ORF-T	-0.015920533	-0.512410363	-1.150851732	-0.427270727	0.201007769
705672	707176	10 +	unknown	no_ovlp2	SUTs	0.707703963	0.045450019	0.164791376	0.132831543	-0.314786066
707176	708776	10 +	YJR149W	ORF-T	ORF-T	0.962304858	0.908351934	0.633539104	-0.22046045	0.200339643
716928	719440	10 +	unknown	no_ovlp	SUTs	0.649569839	0.511622535	0.655987254	0.365395439	-0.772283375
719440	721336	10 +	DAL5	ORF-T	ORF-T	-0.665321264	-0.980843959	0.508395978	0.375177737	-0.31636113
3229	5749	11 -	YKL222C	ORF-T	ORF-T	0.347862923	-0.362799002	0.07706464	-0.316938684	-1.424639293
8901	11533	11 -	FRE2	ORF-T	ORF-T	1.415118831	0.557394549	-0.7372274	0.106254191	-0.669008592
11533	14485	11 -	SUT653	SUTs	SUTs	0.438667094	0.046969229	0.510252871	0.441724459	-1.430053109
17149	18413	11 -	SRY1	ORF-T	ORF-T	0.57935751	0.398270494	-0.005037874	-0.047857019	-4.311741526
18413	18781	11 -	unknown	no_ovlp	CUTs	1.023190174	-0.731537898	-0.13792334	-0.560094729	-2.70350439
22613	23829	11 -	SUT658	SUTs	SUTs	0.967253469	0.304069358	-1.257123556	-1.485229826	-1.914227365
24149	24893	11 -	CUT701	CUTs	CUTs	3.077118207	0.310286115	-0.340591643	-0.110279798	-0.060130756
26565	30709	11 -	YKL215C	ORF-T	ORF-T	-0.029286026	-0.203682198	-0.135298241	0.08296226	-0.604625325
30965	31749	11 -	YRA2	ORF-T	ORF-T	-0.050570887	-0.561703865	-0.189419444	-0.308484716	-0.015823888
31893	34141	11 -	DOA1	ORF-T	ORF-T	0.090564507	-0.489462988	-0.248805324	-0.280635275	-0.097304145
34141	36485	11 -	unknown	no_ovlp	SUTs	0.018653912	0.572966514	0.037177048	-0.023898329	1.383357945
36637	38205	11 -	TRP3	ORF-T	ORF-T	-0.18112662	-0.169518828	-0.705653501	-0.145107701	-0.211541043
38205	38885	11 -	CUT702	CUTs	CUTs	2.17033867	-0.065163472	-0.206969743	0.329668169	-0.080496404
42309	46341	11 -	STE6	ORF-T	ORF-T	0.358530139	-0.109096315	-0.941195189	0.194664248	-0.896915514
45285	46341	11 -	unknown	no_ovlp2	SUTs	0.503940901	-0.27340523	-1.111265028	-0.149179083	-0.934102238
48925	49837	11 -	ADD66	ORF-T	ORF-T	0.022940547	-0.94266211	-0.36914403	-0.23069781	0.071887503
61472	63536	11 -	TOR2	ORF-T	ORF-T	-0.401172304	-0.437447547	-0.205160845	-0.040300466	-0.439142713
63744	66288	11 -	MNN4	ORF-T	ORF-T	-0.039557911	-0.944642318	-0.289685048	0.219543979	0.170127769
69824	70488	11 -	unknown	no_ovlp	SUTs	-0.278773553	-0.288990436	-0.864167401	0.021399826	-0.118316287
70488	73952	11 -	PEX1	ORF-T	ORF-T	0.097332966	-0.022648412	0.151258842	0.076391389	-0.157049163
74792	75592	11 -	YKT6	ORF-T	ORF-T	-0.068356534	-0.665464604	-0.347073529	-0.3316842	0.210717986
75592	75944	11 -	unknown	no_ovlp	SUTs	0.206643284	-0.361553165	0.089962771	0.464034597	0.621047955
77120	78680	11 -	MST1	ORF-T	ORF-T	-0.622452582	-0.464642442	0.069638315	-0.245292422	0.465417443
78776	79920	11 -	SDS22	ORF-T	ORF-T	-0.14970486	-0.168771163	0.773696548	0.065903688	0.074120656
80032	80584	11 -	ACP1	ORF-T	ORF-T	-0.5343845	-0.746578226	0.018005511	-0.159978511	0.326849427
86968	88568	11 -	PXA2	ORF-T	ORF-T	0.329640012	-0.503792723	0.440893491	0.037742929	-0.341075781
88568	89184	11 -	PXA2	ORF-T	ORF-T	-0.122794343	0.257217613	0.746599551	1.080431448	0.655251927
89184	91144	11 -	YKL187C	ORF-T	ORF-T	-0.45597915	-0.437422656	1.275442007	0.850323424	-1.327039223
91144	92688	11 -	YKL187C	ORF-T	ORF-T	-0.565283865	-0.415082805	2.591090862	1.856445791	0.085395134
91576	92688	11 -	unknown	no_ovlp2	SUTs	0.190775796	0.429932108	0.84968349	0.74153086	0.091064695
93359	93672	11 -	MTR2	ORF-T	ORF-T	0.480403678	0.178838449	0.42771148	0.032059484	-0.40491072
95984	96280	11 -	CUT704	CUTs	CUTs	2.927660692	-0.27873748	-0.204096	-0.366084703	0.411020113
98408	1.00E+05	11 -	unknown	no_ovlp	CUTs	2.375675969	0.071938618	-0.760065516	-0.202831405	-0.393938731
1.00E+05	100736	11 -	unknown	no_ovlp	SUTs	0.476895491	0.429714446	0.459239844	0.195031715	0.593563195
110264	112512	11 -	COY1	ORF-T	ORF-T	0.131352885	-0.440034419	-0.443411766	-0.0903551	-0.256103742
112512	112896	11 -	unknown	no_ovlp2	SUTs	-0.0114142	0.54553313	0.151121143	-0.085817411	0.002642167
112896	115200	11 -	STE3	ORF-T	ORF-T	-0.172554638	1.16731095	1.290818196	-0.11711141	0.084729485
114888	115200	11 -	unknown	no_ovlp2	SUTs	0.903981444	0.293658019	0.50270203	-0.149641964	-0.171130066

118312	118560	11 -	LST4	ORF-T	ORF-T	1.111433987	0.756268348	-0.874949732	-0.834374156	-0.359360989
120312	122312	11 -	TPO5	ORF-T	ORF-T	-0.154006473	-0.193279632	-0.242055654	-0.128508828	0.051019466
131256	133640	11 -	YKL169C, KK(	other	other	-0.380421427	-0.777633421	0.157611116	0.040683497	-0.07278245
133640	134152	11 -	MRP49	ORF-T	ORF-T	-0.699281631	-0.853975594	0.369540081	0.052538548	0.099074886
134400	135856	11 -	TPK3, YKL16!	other	other	-0.048364317	-0.461421767	-0.731263734	-0.255826923	0.248302231
135856	137856	11 -	YKL165C-A	other	other	0.365337463	0.086939381	1.086281294	0.860375691	-1.263679879
137856	140736	11 -	MCD4	ORF-T	ORF-T	-1.07893953	-0.400870446	-0.879542824	-0.448433479	-0.122944444
140736	141616	11 -	tE(UUC)K	other	other	-1.81837393	-1.059361706	-0.462020643	-1.176327306	-0.57984184
141038	141616	11 -	unknown	no_ovlp2	SUTs	0.143638903	0.474636698	0.863500537	0.180449504	0.057071585
142888	143192	11 -	PIR1	ORF-T	ORF-T	2.286866963	-0.260158794	-0.389581011	0.53721625	-1.518400904
143520	143928	11 -	unknown	no_ovlp	CUTs	1.248081359	0.109203345	0.739765263	0.736470627	-0.317891622
143928	145576	11 -	unknown	no_ovlp2	SUTs	-0.039135041	0.316633107	-0.059674525	0.063888523	-0.011663048
145576	147520	11 -	YKL162C-A	other	other	0.481573948	1.129149935	1.739618948	0.3967235	-0.737499889
146216	147520	11 -	unknown	no_ovlp2	SUTs	0.218563056	0.286587613	-0.14037457	-0.147580036	-0.321039416
147520	148872	11 -	YKL162C	ORF-T	ORF-T	0.026850367	-0.563644159	1.065216995	0.619454409	0.342342994
149080	150760	11 -	YKL161C	ORF-T	ORF-T	-0.099638785	-0.131821014	-0.08273217	0.084208508	0.034945909
153672	154496	11 -	RCN1	ORF-T	ORF-T	0.090180609	-0.297565261	-0.148985974	0.108636428	-0.466914207
157744	158224	11 -	CUT705	CUTs	CUTs	2.571837037	0.153657826	-1.375327631	-0.352491857	1.465832157
159472	161368	11 -	RSM22	ORF-T	ORF-T	-0.7609169	-0.780732383	-0.227628641	-0.283563236	0.289592977
163512	164416	11 -	GPM1	ORF-T	ORF-T	-0.280754327	-0.190004463	-0.170009117	-0.138108082	-0.281631977
164816	166112	11 -	YKL151C	ORF-T	ORF-T	-1.073995336	-0.611033903	1.654633795	0.892704235	0.872694161
167544	168848	11 -	DBR1	ORF-T	ORF-T	0.307353943	-0.27930936	-0.367840309	-0.362168845	-0.121568032
171320	171528	11 -	SDH1	ORF-T	ORF-T	0.442355649	0.140616975	0.404874515	-0.897055899	0.130905353
171528	173016	11 -	unknown	no_ovlp	SUTs	-0.019608383	0.597938383	0.11309159	0.224486432	0.867685262
175824	176544	11 -	RPC25	ORF-T	ORF-T	0.506353769	-0.226037385	-0.520471127	-0.508278213	0.588598557
184536	185032	11 -	HSK3	ORF-T	ORF-T	0.494487316	-0.643298454	-0.805252974	-0.440130413	-0.636343776
185176	185800	11 -	MRPL31	ORF-T	ORF-T	-0.629213263	-1.484042474	-0.554610262	-0.266645341	-0.964251673
186358	188766	11 -	APL2	ORF-T	ORF-T	-0.042699875	-0.596202348	-0.140388078	0.041373366	-0.00193939
188934	191478	11 -	Oct-01	ORF-T	ORF-T	0.063349503	-0.251429535	-0.013674559	-0.100031041	0.608656208
191478	193422	11 -	YKL133C	ORF-T	ORF-T	-0.157728747	0.133178927	1.17485709	0.727656945	0.730697172
193110	193422	11 -	unknown	no_ovlp2	SUTs	0.146983155	0.506517173	-0.568008178	-0.691352758	-0.473931499
193422	195022	11 -	RMA1	ORF-T	ORF-T	0.432746985	-0.99082434	-0.766800001	-0.373127926	-0.355754282
195158	196086	11 -	SHE2	ORF-T	ORF-T	0.445602486	-0.369472063	-0.00216002	-0.241033257	0.07317458
196246	200222	11 -	MYO3	ORF-T	ORF-T	-0.117859196	0.018599157	0.12294531	-0.192755718	-0.283612111
200430	201454	11 -	PMU1	ORF-T	ORF-T	0.007855083	-0.387504647	-0.834333948	-0.628515123	-0.321108428
203830	204918	11 -	SUT663	SUTs	SUTs	0.225852959	-0.117179898	-0.229156655	0.686776698	1.003684133
212062	212694	11 -	SRP21	ORF-T	ORF-T	-0.101000604	-0.505470823	-0.192514353	-0.147436005	-0.05168497
218078	218894	11 -	VPH2	ORF-T	ORF-T	0.805142004	0.142901028	-0.553240724	-0.365930814	-0.661678461
220774	222734	11 -	PRR1, YKL11!	ORF-T	ORF-T	0.103933538	-0.353433129	-0.232599255	-0.396709855	-0.044233302
222918	224134	11 -	YKL115C, API	ORF-T	ORF-T	0.037387558	-0.150154411	-0.477790074	0.185365628	-0.331286558
224326	225542	11 -	RAD27	ORF-T	ORF-T	-0.222726841	-0.328651782	-0.721617463	-0.193276659	0.791404483
228510	229678	11 -	YKL111C, KTI	other	other	0.697524417	-0.218642929	0.015848413	-0.236648469	0.000433048
230230	230622	11 -	CUT707	CUTs	CUTs	2.739471423	-0.042661058	0.973875434	-0.159025898	0.381562558
235646	236694	11 -	YKL106C-A	ORF-T	ORF-T	1.605620186	-0.186152034	-0.201293064	-0.891196326	0.303679953
238598	242374	11 -	YKL105C	ORF-T	ORF-T	-0.840629463	-0.301763506	0.344805604	0.088503855	0.73456728
242630	245214	11 -	GFA1	ORF-T	ORF-T	-0.188915749	0.080207192	-0.151878338	-0.192860907	-0.027122051
245214	245542	11 -	unknown	no_ovlp2	CUTs	2.446870683	0.969388488	0.391486243	-0.419135115	-0.537378438
245542	247398	11 -	LAP4	ORF-T	ORF-T	-1.729537028	0.040831221	0.820934953	0.622214488	1.542009757
253086	255126	11 -	YKL100C	ORF-T	ORF-T	-0.3365278	-0.460672522	0.253474239	0.333672221	0.291900858
255294	256183	11 -	UTP11	ORF-T	ORF-T	0.187887965	-0.255035437	-0.112410703	-0.121229853	0.965843583
257615	258007	11 -	CUT708	CUTs	CUTs	2.73727751	-0.136893561	-0.844143652	-0.723909025	-0.753244119
258959	260055	11 -	SUT664	SUTs	SUTs	1.672761144	0.42199447	0.296295179	-0.151888732	-0.643226822
263903	265687	11 -	unknown	no_ovlp2	CUTs	2.009400691	0.735671588	-1.196629983	-0.647540739	-1.058733976
265687	269175	11 -	BUD2	ORF-T	ORF-T	-0.300771067	-0.415992886	0.066046315	-0.113828673	0.125873049
269311	270327	11 -	YKL091C	ORF-T	ORF-T	-0.110829193	-0.786644811	0.767741054	0.506863145	-0.066426331
270415	271367	11 -	SUT666	SUTs	SUTs	1.078965497	-0.910547177	-0.095497508	-0.164849641	-0.769996138
273831	274711	11 -	CUT710	CUTs	CUTs	1.761425597	-0.059975883	-0.64407906	0.035200059	0.570801598
276735	277607	11 -	CYT2	ORF-T	ORF-T	-0.646381382	-0.521264792	0.523271958	-0.01894043	0.210345891
278079	278295	11 -	SUT668	SUTs	SUTs	-0.727546427	-0.428499651	-0.091277602	0.109456273	-0.109414303
280567	282039	11 -	RRP14	ORF-T	ORF-T	0.544175843	-0.104901952	-0.713171303	-0.372697893	0.026838267
285463	285983	11 -	CUT711	CUTs	CUTs	2.366165597	0.106931145	-0.150584659	-0.040236983	0.606836341
292439	294039	11 -	PSY1, YKL07!	ORF-T	ORF-T	-0.2357822	-0.554001646	0.131963183	0.041203803	0.41688304
294151	295855	11 -	MUD2	ORF-T	ORF-T	0.440893351	-0.160356475	-0.524559298	-0.19254653	0.154569385
295855	298791	11 -	CUT713	CUTs	CUTs	1.7531733	0.373718538	0.229337334	0.381999998	-0.22239797
304399	306703	11 -	SUT670	SUTs	SUTs	0.940225202	-0.097422986	-0.588838218	-0.449600724	-0.219306468
307063	307647	11 -	CUT714	CUTs	CUTs	2.457829817	-0.083793523	-0.019468388	-0.372385853	0.181986257
309087	309511	11 -	SUT671	SUTs	SUTs	0.728761365	-0.333159503	0.2484859	0.301646109	0.033638707
313199	313839	11 -	unknown	no_ovlp	CUTs	2.269671956	0.281051196	0.224944916	-0.22024024	-1.872678593
315959	316767	11 -	YET1	ORF-T	ORF-T	-0.429721125	-0.60162357	0.409337201	0.061428247	-0.244107543
316767	316999	11 -	SUT673	SUTs	SUTs	1.110990457	-0.073070214	0.375556889	0.265675713	-0.440395593
320495	321319	11 -	YKL063C	ORF-T	ORF-T	-0.202594546	-0.402008509	-0.053934795	-0.269225943	0.208063435
325415	325911	11 -	CUT716	CUTs	CUTs	1.687017923	-0.878492567	-1.483265404	-0.403700308	-0.631497898
325999	327183	11 -	FBA1	ORF-T	ORF-T	-0.469430274	-0.042306296	-0.128767511	-0.091926796	-0.144608622
327183	327583	11 -	unknown	no_ovlp2	CUTs	1.521030553	0.211724665	-1.15895163	-0.797921764	-0.827197546
327583	329143	11 -	MPE1	ORF-T	ORF-T	0.221291017	-0.034911891	-0.445549186	-0.455775299	-0.661222879
331231	333671	11 -	NUP120	ORF-T	ORF-T	-0.122678908	-0.22978433	-0.283649324	-0.143652566	0.175515429
333871	334623	11 -	TMA19	ORF-T	ORF-T	-0.235181839	-0.216823323	-0.361248689	-0.226643062	-0.053200368
334623	335975	11 -	OAR1	ORF-T	ORF-T	0.714524299	0.794658557	0.457972915	0.034365631	0.680250423
336215	338503	11 -	DEF1	ORF-T	ORF-T	-0.44743024	-0.374507064	-0.418967323	-0.013853546	-0.19965518
338719	339159	11 -	MDM35	ORF-T	ORF-T	-1.043423115	-0.43782948	0.696470559	0.155396207	-0.156561416
339287	340143	11 -	ASK1	ORF-T	ORF-T	-0.034127625	-0.417269823	-0.313147729	-0.32406917	0.167246595
340143	340695	11 -	unknown	no_ovlp	SUTs	0.033197729	-0.246848151	-0.319045668	-0.510110079	-0.427648077

342327	345703	11 -	YKL050C	ORF-T	ORF-T	0.59377795	0.209831967	-0.237018103	-0.181147433	0.465623363
345487	345703	11 -	unknown	no_ovlp2	SUTs	0.093811872	-0.645356705	-0.070289238	-0.502371604	0.413229734
345703	346439	11 -	CSE4	ORF-T	ORF-T	-0.153681347	-0.519759276	0.38468323	-0.036364721	0.767494453
346711	348847	11 -	ELM1	ORF-T	ORF-T	-0.025664082	-0.174248122	-0.258778382	-0.370678169	0.196088696
350695	352495	11 -	DCW1	ORF-T	ORF-T	-0.48984421	-0.415398819	-0.223799363	0.041152175	-0.198208426
353559	354679	11 -	unknown	no_ovlp	CUTs	1.285643186	-0.392719742	-0.95800163	0.216561948	0.009857756
360439	361519	11 -	NFU1	ORF-T	ORF-T	0.169358084	-0.667133866	-0.030088474	-0.042166732	-0.109632127
361519	361967	11 -	SUT677	SUTs	SUTs	0.716169971	-0.092251923	0.808224519	-0.108821645	-0.443414339
368080	369200	11 -	unknown	no_ovlp	SUTs	0.414322289	-0.336475583	0.361310155	0.568065055	-0.085384016
379552	381680	11 -	IXR1	ORF-T	ORF-T	0.055162248	-0.113653078	-0.402599461	-0.001912386	-0.471864426
382240	384456	11 -	MAE1	ORF-T	ORF-T	0.46237324	-0.183030278	-0.616879888	0.014852688	-0.354329973
386280	386904	11 -	CUT719	CUTs	CUTs	3.889046544	-0.019140677	-1.839962784	-0.385895232	-0.137636428
388800	389664	11 -	GPX1	ORF-T	ORF-T	-0.085119649	-0.472552725	1.030415044	0.784724981	-1.065622957
391288	391944	11 -	PAN3	ORF-T	ORF-T	-0.232955556	-0.256535146	-0.008025324	0.010989922	-0.226020437
392072	393048	11 -	URA6, YKL02	other	other	-0.667842359	-0.572325143	-0.154367671	0.11321889	0.400710038
394320	396792	11 -	CDC16	ORF-T	ORF-T	-0.002982417	-0.570834197	-0.247427927	-0.267176947	0.172537847
396944	398256	11 -	CDC16, MAK	ORF-T	ORF-T	0.300684412	-0.26592684	-0.425311532	0.130036125	0.169012944
398448	401784	11 -	MAK11, SPT2	ORF-T	ORF-T	-0.103898877	-0.569455839	0.038146385	0.25870228	-0.041359991
403136	403552	11 -	YKL018C-A	ORF-T	ORF-T	-0.272604472	-0.563812899	0.440719206	-0.051203249	0.990733466
404800	406880	11 -	HCS1	ORF-T	ORF-T	0.340343046	-0.601530386	-0.623302451	-0.523809116	-0.973951073
406984	407760	11 -	ATP7	ORF-T	ORF-T	-0.317995163	-0.379443382	0.521619871	0.066696646	0.221243451
411184	416584	11 -	URB1	ORF-T	ORF-T	0.281452699	-0.27092475	-0.707662186	-0.059665808	0.076329579
416952	417696	11 -	ARC19	ORF-T	ORF-T	0.009978829	-0.305957999	-0.014488736	-0.248264003	-0.235487547
419712	421024	11 -	CCE1	ORF-T	ORF-T	-0.630158414	-1.034727878	0.053919219	0.315360936	0.400770713
424240	425616	11 -	UFD4	ORF-T	ORF-T	-0.22515632	-0.543883642	0.279918912	0.135880042	0.087469955
426808	428328	11 -	LAC1	ORF-T	ORF-T	-0.199072925	-0.424692224	-0.308490413	0.247071861	0.179734726
429800	430384	11 -	SFT1	ORF-T	ORF-T	1.3150461	-1.109897226	-0.742751999	-0.278206164	-0.339057194
430384	430776	11 -	SNR87	other	other	2.898330446	-0.528490165	-0.771026837	-0.525736215	-0.390971625
430776	430984	11 -	unknown	no_ovlp	CUTs	1.03773635	-0.160722606	-0.334565763	-1.34222306	0.942936886
432560	434640	11 -	BYE1	ORF-T	ORF-T	0.485305878	-0.702127376	-0.34800096	0.002537982	-0.035407527
436640	437160	11 -	MRP17	ORF-T	ORF-T	-0.757721742	-0.704771211	0.050532686	0.004061139	-0.171546158
438336	439064	11 -	MET14	ORF-T	ORF-T	-0.615926888	-0.474194704	-0.337816126	-0.168716719	0.016522253
440424	442432	11 -	VPS1	ORF-T	ORF-T	-0.220755458	-0.303214874	-0.289715984	0.046249626	-0.000929608
446344	447672	11 -	ECM9	ORF-T	ORF-T	-0.206301532	-0.203051031	0.622113088	0.123578858	-0.272987514
447672	447896	11 -	unknown	no_ovlp2	SUTs	-0.243568809	0.393167756	0.039218392	-0.268284475	-0.160942635
447896	449984	11 -	YKR005C	ORF-T	ORF-T	0.172572449	0.388330667	-0.122934148	-0.331376317	0.065032121
449984	450872	11 -	MRPL13	ORF-T	ORF-T	-0.792043384	-0.69590685	-0.240733337	-0.123288237	-0.111691875
450976	451624	11 -	unknown	no_ovlp	CUTs	2.3575328	0.262683101	-0.136978854	-0.110032421	0.635520022
454360	455064	11 -	FOX2	ORF-T	ORF-T	-1.492576229	-0.576024264	1.513641959	0.096878729	2.479669115
455064	458456	11 -	FOX2	ORF-T	ORF-T	-0.616252823	0.335870097	2.200417854	0.078724017	3.938277361
456912	458456	11 -	unknown	no_ovlp2	SUTs	-0.236964522	0.346657236	0.157221014	-0.003070564	0.061297499
458456	460992	11 -	TOF2	ORF-T	ORF-T	0.000563195	-0.075953878	-0.541388041	-0.115940985	0.854545484
461176	462440	11 -	YKR011C	ORF-T	ORF-T	-0.681221199	-0.556470956	1.121528104	0.274563439	0.712782693
462440	464784	11 -	YKR012C	other	other	-0.395778859	-0.031959081	-0.406224798	-0.263183431	0.353196557
464784	465840	11 -	YPT52	ORF-T	ORF-T	-0.028241522	-0.516962869	0.172236094	-0.057624215	-0.35707086
468536	468992	11 -	YKR015C	ORF-T	ORF-T	1.465263816	0.21965841	0.976171667	-0.196594685	-0.669127686
471152	473192	11 -	YKR017C	ORF-T	ORF-T	-0.02160063	-0.769922612	-0.11712816	0.078774962	-0.672476827
473192	475544	11 -	YKR018C	ORF-T	ORF-T	-0.121016625	-0.323171804	0.168032366	0.003545677	-0.081472536
475544	475792	11 -	unknown	no_ovlp2	SUTs	-0.433189397	-0.486863466	0.062527714	-0.035152201	0.85733481
475792	477704	11 -	IRS4	ORF-T	ORF-T	0.071267473	-0.233515538	-0.033407248	0.12380478	0.66707254
477704	478648	11 -	unknown	no_ovlp	CUTs	1.463667965	0.208155479	-0.043707077	-0.141355139	0.528606911
481712	482840	11 -	NTR2	ORF-T	ORF-T	-0.319384091	-0.697227864	-0.286673256	-0.263510605	-0.008311425
484744	487048	11 -	DBP7	ORF-T	ORF-T	0.27548645	0.062937262	-0.856568797	-0.321567636	0.477639937
488224	489400	11 -	GCN3	ORF-T	ORF-T	-0.100583608	-0.59164006	-0.680407792	-0.155835261	-0.301333272
492624	493296	11 -	CUT721	CUTs	CUTs	2.266076977	0.350854186	-0.996377681	0.009760123	0.105735571
497200	499456	11 -	SET3	ORF-T	ORF-T	-0.307350546	-0.614153748	0.186009898	-0.005308443	0.131121073
500784	506128	11 -	SPO14	ORF-T	ORF-T	0.084184973	-0.433005864	-0.331754017	0.161639829	-0.333842108
508232	509856	11 -	OPI8, CAF4	other	other	0.390910534	-0.301681059	-0.575161929	-0.346689402	-0.102543638
509856	510312	11 -	unknown	no_ovlp2	SUTs	0.000568296	-0.151668577	0.125415221	0.319873456	0.056620389
510312	511536	11 -	SPC34	ORF-T	ORF-T	-0.446798322	-0.566582099	0.230456814	-0.158223391	0.230274574
511536	512816	11 -	KAE1	ORF-T	ORF-T	0.151820166	-0.276018014	0.067956152	-0.049901452	0.211566645
513160	513792	11 -	YKRCdelta11	other	other	0.313227393	-0.789377437	-0.540898762	-0.479695422	-1.053522762
513792	514064	11 -	SUT679	SUTs	SUTs	0.641011447	0.193991156	0.444796868	-0.094411199	-0.042281667
519208	519472	11 -	SUT680	SUTs	SUTs	-0.964618418	-0.598148419	-0.815444055	-0.309461827	0.510437574
520424	521384	11 -	YKR043C	ORF-T	ORF-T	0.257870585	-0.134841568	-0.45805049	-0.263546055	-0.172699438
523720	524728	11 -	PET10	ORF-T	ORF-T	0.172207787	-0.521547686	0.177341293	0.08131213	-0.018388742
524904	526304	11 -	NAP1	ORF-T	ORF-T	0.007645142	-0.027869422	-0.148800234	0.130414792	-0.135162493
526400	526880	11 -	FMP46	ORF-T	ORF-T	0.589447594	1.303871827	1.877820405	0.694744267	0.579056345
526880	527504	11 -	SUT682	SUTs	SUTs	0.381454064	0.299954924	0.755788493	0.017171472	1.7341875
531832	533120	11 -	MRS4	ORF-T	ORF-T	0.123437936	-0.84008405	0.110769782	0.294114576	-0.873631567
533120	533608	11 -	unknown	no_ovlp2	SUTs	0.759468756	-0.429871567	-0.340085522	-0.038119672	-0.382540803
533608	535096	11 -	YSR3	ORF-T	ORF-T	0.054657075	-0.33017591	0.171238907	-0.080335821	0.418597903
544200	547616	11 -	DYN1	ORF-T	ORF-T	-0.124671247	-0.327367762	-0.129938634	-0.187837316	0.289468329
553872	554288	11 -	CUT723	CUTs	CUTs	2.862449617	0.020231002	-1.154830931	-0.218896817	0.519988964
556264	557000	11 -	CUT724	CUTs	CUTs	3.276426213	0.286061413	-0.169308644	-0.651035247	-0.569861893
558336	559008	11 -	SNR42	other	other	4.466807271	-0.537811933	-0.213254728	-0.051226818	-0.218549292
560288	561952	11 -	LAS1	ORF-T	ORF-T	0.305232001	-0.081563176	-0.096994822	-0.134374869	0.70394131
564776	565560	11 -	PAM17	ORF-T	ORF-T	-0.343971096	-0.542575579	0.178638875	0.054743572	0.358620071
565736	566872	11 -	CCP1	ORF-T	ORF-T	0.336938628	0.160078949	0.083223291	0.241438602	-0.523469168
569824	570560	11 -	BET3	ORF-T	ORF-T	0.063733518	-0.44981821	-0.076260668	-0.089866808	-0.016037163
572968	573912	11 -	unknown	no_ovlp	CUTs	1.427944849	0.116891839	-0.557177639	-0.062497566	0.437803109
574336	575672	11 -	DRE2	ORF-T	ORF-T	-0.216496643	-0.699308104	0.407179116	0.025493295	-0.259271283

575976	577872	11	-	SIS2, YKR073	other	other	-0.171342001	-0.243296244	-0.093002277	0.154799031	-0.223065851
579328	580464	11	-	YKR075C	ORF-T	ORF-T	0.422490546	-1.008380744	0.591780781	0.24110933	-0.400633718
582216	583024	11	-	CUT728	CUTs	CUTs	1.646282692	0.44998379	0.201446888	-0.126364594	0.614240447
586288	589200	11	-	TRZ1	ORF-T	ORF-T	0.388728876	-0.305495598	-0.51961963	-0.043354233	-0.006810607
591096	592208	11	-	RPF2	ORF-T	ORF-T	0.181812979	-0.528502029	-0.805624183	-0.066456924	0.413825301
595976	596536	11	-	DAD2	ORF-T	ORF-T	-0.12400093	-0.969551227	-0.272167012	-0.324654621	0.071165002
596624	599328	11	-	HBS1	ORF-T	ORF-T	-0.090516306	-0.492308279	-0.702581305	-0.133096659	-0.808059913
598600	599328	11	-	unknown	no_ovlp2	SUTs	-0.364835706	-0.40538781	-0.036994613	-0.435023297	-0.136776298
602784	603873	11	-	OMA1	ORF-T	ORF-T	0.56081578	-0.523737488	-0.54253637	-0.065141293	-0.308679864
604009	605065	11	-	TVP38	ORF-T	ORF-T	-0.364601613	-0.801439534	-0.322898063	-0.125746756	0.011329617
605257	608065	11	-	TGL4	ORF-T	ORF-T	0.056907693	-0.28651573	-0.155874059	-0.041310064	-0.422223419
610065	610825	11	-	unknown	no_ovlp	CUTs	1.165050315	-0.47110192	-0.443696662	-0.420316771	0.013203742
612090	613562	11	-	SRP40	ORF-T	ORF-T	-0.201657877	-0.479280688	-0.72399544	-0.01017887	0.328128252
613562	614626	11	-	CUT729	CUTs	CUTs	1.367818011	0.392171935	-0.105200557	-0.033860774	-0.529323385
618018	618394	11	-	RPL40B	ORF-T	ORF-T	0.932790657	-0.738243139	-1.137429896	-1.034584851	-0.178906244
624906	626138	11	-	unknown	no_ovlp	CUTs	1.243031648	0.174115629	1.012931974	0.597664162	-0.028778213
626138	626514	11	-	unknown	no_ovlp	SUTs	-0.216190064	0.751761112	0.032656385	-0.02028164	0.475736597
632690	634946	11	-	UBP11	ORF-T	ORF-T	-0.207565884	-0.150163683	0.999927632	0.671224715	0.215578936
638450	639962	11	-	SKG1	ORF-T	ORF-T	-0.313096056	-1.030899205	0.115571209	0.390340098	-0.137519079
14545	15889	11	+	COS9	ORF-T	ORF-T	0.465994069	-1.129909435	-0.799521442	-0.280583752	-2.805691696
18665	19833	11	+	SUT218	SUTs	SUTs	1.09608738	0.062149875	-0.302789372	-0.139637305	-3.42136435
22673	24209	11	+	JEN1	ORF-T	ORF-T	-0.936257142	0.084995599	3.499076233	3.21286719	1.947972585
25209	26457	11	+	URA1	ORF-T	ORF-T	-0.188673547	0.915711114	0.287843159	-0.049258143	0.716053731
34337	36681	11	+	SAC1	ORF-T	ORF-T	-0.308130287	-0.617089228	-0.467219472	0.022509272	-0.34018333
38585	38817	11	+	unknown	no_ovlp	CUTs	2.774456201	0.639441351	-0.469542842	0.123897305	-0.330048277
38953	42369	11	+	UBA1	ORF-T	ORF-T	-0.338488214	-0.202353336	-0.077929078	0.009400099	0.195326251
47137	48169	11	+	CBT1	ORF-T	ORF-T	-0.440285071	-0.824179645	0.741687425	-0.02133962	-0.772761627
48169	49033	11	+	YKL207W	ORF-T	ORF-T	-0.140343104	-0.438138569	-0.767608777	-0.230361792	-0.147531224
50017	53305	11	+	LOS1	ORF-T	ORF-T	0.038473946	-0.704631148	-0.690047704	-0.270954758	-0.153115438
53681	55593	11	+	EAP1	ORF-T	ORF-T	-0.130558375	-0.433231949	-0.131284278	0.175755412	-0.448596879
66508	67652	11	+	SUT221	SUTs	SUTs	1.211681585	-1.196308977	-0.556750955	-0.034960156	-0.687931036
69780	70412	11	+	SUT222	SUTs	SUTs	1.86094701	-0.240553523	-0.160085055	-0.701833397	0.347353188
74036	74852	11	+	unknown	no_ovlp	SUTs	0.550179356	0.270550716	0.754211422	-0.344765333	0.055803774
75796	77204	11	+	MIA40	ORF-T	ORF-T	-0.379313542	-0.619495291	0.366437252	0.010219504	0.16072504
81020	82724	11	+	DPH2	ORF-T	ORF-T	-0.313503245	-0.312070054	-0.291967299	0.019877616	0.750965553
83092	83724	11	+	CNB1	ORF-T	ORF-T	-0.362020301	-0.63230191	-0.071985076	0.005358339	0.073130641
84588	86124	11	+	HYM1	ORF-T	ORF-T	0.149131798	-0.653009086	-0.218763832	0.276889497	-0.708221444
87340	87900	11	+	unknown	no_ovlp	CUTs	3.107203316	0.375384757	-0.849392027	-0.971703744	-0.305272835
94164	96388	11	+	ASH1	ORF-T	ORF-T	-0.367220634	-0.536319582	-0.354780299	0.008522729	0.142279009
96644	98444	11	+	SPE1	ORF-T	ORF-T	-0.410595219	-0.858922816	0.032799262	0.217432354	-0.120222834
98572	99780	11	+	LOT5	ORF-T	ORF-T	-0.093491664	0.390241946	0.322405601	-0.245205148	0.123703685
100140	100356	11	+	FAS1	ORF-T	ORF-T	0.129567655	-0.493872439	-1.006683652	-0.212116276	-1.18475946
107116	108732	11	+	PRS1	ORF-T	ORF-T	-0.006080764	-0.371891587	-0.45316413	-0.222564107	0.127481999
109244	109588	11	+	RPL17A	ORF-T	ORF-T	-0.30293086	-0.13691184	-0.161342801	-0.208143711	-0.046878682
118748	120396	11	+	ZRT3	ORF-T	ORF-T	0.09112503	-0.599128275	-0.778852218	-0.585546285	0.272253538
122500	125740	11	+	SNU114	ORF-T	ORF-T	0.155364834	-0.385471529	-0.290234292	-0.005608238	-0.148093907
125740	127156	11	+	EBP2	ORF-T	ORF-T	0.273384107	-0.337849827	-0.798270221	-0.047229024	0.182825753
127412	130188	11	+	YKL171W	ORF-T	ORF-T	-0.504491441	-0.469752966	0.953490892	0.531475229	0.422689941
130564	131220	11	+	MRPL38	ORF-T	ORF-T	-0.573454718	-1.186003144	-0.283920161	-0.128304063	-0.09952361
134372	134684	11	+	unknown	no_ovlp	CUTs	2.179887689	0.461219609	0.26887391	-0.423024122	0.071003497
136356	137124	11	+	unknown	no_ovlp	CUTs	2.61398715	0.546515304	-1.766328485	-0.581833098	-2.572909582
143212	143596	11	+	SUT228	SUTs	SUTs	-0.943012266	-0.393339988	1.224204897	0.464690057	0.194370846
143596	144044	11	+	SUT228	SUTs	SUTs	2.194775912	-0.539279252	-0.366777439	-0.483822032	-0.656359056
144372	145772	11	+	PIR3	ORF-T	ORF-T	0.014627127	0.362630932	1.634068509	0.954710004	0.051892457
151220	152940	11	+	SUT229	SUTs	SUTs	1.084949492	-0.291506565	-0.502884701	-0.199662984	-0.427512472
153268	153796	11	+	ELF1	ORF-T	ORF-T	0.101835017	-0.426943687	-0.165421069	-0.346156474	-0.273195128
154988	155268	11	+	APE2	ORF-T	ORF-T	0.574596248	0.912839874	0.341054212	-0.157637842	-0.545252295
158580	159012	11	+	RPS27A	ORF-T	ORF-T	0.772169423	-1.078592248	-1.089404917	-0.911295863	-0.425970715
161532	162404	11	+	SRP102	ORF-T	ORF-T	0.130739155	-0.428016435	-0.806938167	-0.485113665	0.12490987
164948	165484	11	+	MCR1	ORF-T	ORF-T	3.704046068	-0.963523237	-1.696496045	-0.875723542	-0.325621909
171772	173964	11	+	AVT3	ORF-T	ORF-T	-0.266111341	-0.13430414	0.090447126	-0.002661248	0.209309499
174172	175796	11	+	RPT1, YKL14!	ORF-T	ORF-T	0.012926254	-0.369662394	-0.131935322	-0.02831577	0.019165142
176740	178444	11	+	LTV1	ORF-T	ORF-T	0.344679234	-0.057085651	-0.24469195	0.034100002	0.820552023
178444	179316	11	+	MRP8	ORF-T	ORF-T	-0.23258727	-1.065127318	0.191335075	0.136716079	-0.067946774
179644	180380	11	+	SDH3	ORF-T	ORF-T	-0.633824214	-0.177394438	1.073678902	0.342173191	0.731832304
180540	182596	11	+	TGL1	ORF-T	ORF-T	-0.34162875	-0.721304983	0.009313426	0.022791674	-0.397386234
182804	184580	11	+	CTK1	ORF-T	ORF-T	-0.209895136	-0.481795333	-0.141565331	-0.30706399	0.184528459
185924	186498	11	+	CMC1, YKL13	other	other	-0.04762519	-0.890399515	-0.272466209	-0.420747636	-0.625614616
188930	190610	11	+	unknown	no_ovlp	SUTs	0.300151158	0.007211628	-0.18177039	-0.467806566	0.359502626
191690	192698	11	+	unknown	no_ovlp	CUTs	1.800300629	0.368427081	-1.107594513	-0.048328337	-0.276826769
195322	195714	11	+	SUT231	SUTs	SUTs	0.830696928	-0.521025338	-0.845893831	-0.171384406	-0.297774811
196330	196730	11	+	unknown	no_ovlp	CUTs	1.295700583	-0.359564043	0.232150994	0.511819631	1.334067059
200378	201698	11	+	SUT232	SUTs	SUTs	0.900879384	-0.030714098	-0.063088215	-0.012940225	0.50761587
201698	202626	11	+	tK(CUU)K	other	other	0.457708521	-1.055642299	-0.333240024	0.052659724	-0.154761529
203154	205042	11	+	PGM1	ORF-T	ORF-T	0.62899904	0.328265673	-0.68006156	-0.501139524	-0.0015739
205154	207466	11	+	YPK1	ORF-T	ORF-T	-0.27062813	-0.620306028	0.003432801	-0.02058226	-0.138591963
207778	209922	11	+	RRN3	ORF-T	ORF-T	1.517954424	0.026476353	-0.281621724	-0.353159261	0.21267682
210130	212138	11	+	SSH4, YKL12	ORF-T	ORF-T	0.488494219	-0.470218347	0.31302757	0.521899062	-0.080853032
213578	216466	11	+	YKL121W	ORF-T	ORF-T	0.29984416	-0.143158983	0.328303337	0.098731284	-0.212576278
216906	218138	11	+	OAC1	ORF-T	ORF-T	-0.122257367	-0.693439064	-0.081040088	0.186415825	0.431756705
219930	220834	11	+	SBA1	ORF-T	ORF-T	-0.210090674	-0.349560076	-0.043410628	-0.013817306	-0.01623428
222890	223626	11	+	CUT228	CUTs	CUTs	2.161502204	-0.30543311	-0.708060439	-0.026304305	0.327278939

226066	228554	11 +	ABF1	ORF-T	ORF-T	0.153036824	-0.510556357	-0.499840041	-0.167827554	-0.218640811
229946	230386	11 +	SUT233	SUTs	SUTs	1.849493784	0.452014094	0.091707391	-0.086604412	-0.589511316
230978	231554	11 +	unknown	no_ovlp	SUTs	-0.351448351	-0.661260131	0.292067084	-0.119300641	-1.042206698
231554	233898	11 +	HAP4	ORF-T	ORF-T	-0.150840914	-0.61320382	0.153305014	-0.081014221	-0.016288921
234066	235554	11 +	SLD2	ORF-T	ORF-T	-0.18262525	0.154395595	0.247244452	-0.375641223	0.857084349
235554	237066	11 +	YKL107W	ORF-T	ORF-T	-0.395259966	0.759773484	0.665638746	0.080752752	0.420164789
236794	237066	11 +	unknown	no_ovlp2	SUTs	-0.251859887	0.172716898	0.266241417	0.014158175	-0.144658062
237066	238666	11 +	AAT1	ORF-T	ORF-T	0.510887111	-0.210960969	-1.344782065	-0.495568117	-0.494281774
238978	241202	11 +	unknown	no_ovlp	CUTs	3.000549447	0.746308155	-1.293535777	0.106040558	-1.057807706
245786	249826	11 +	CUT229	CUTs	CUTs	1.963601651	0.299499094	-0.40953127	-0.512844251	-0.523287694
249826	251994	11 +	HSL1	ORF-T	ORF-T	0.504253072	-0.608140912	-0.513014202	-0.549917838	-0.375287859
255386	255659	11 +	CUT230	CUTs	CUTs	2.956885196	0.113766034	-0.419326954	0.537960304	0.148216025
256395	257595	11 +	YKL098W	ORF-T	ORF-T	0.581405068	-0.076065433	-0.302033699	-0.338442797	-0.427061124
258859	259371	11 +	CWP2	ORF-T	ORF-T	-0.369017723	-0.274669869	0.328109466	-0.040078331	-0.072744354
259771	260739	11 +	unknown	no_ovlp	CUTs	1.262026417	-0.338549578	-0.491393406	0.166732552	-0.915145852
260739	261611	11 +	CWP1	ORF-T	ORF-T	-0.231028244	-0.235018007	0.06439939	-0.008588484	0.401784485
261771	262995	11 +	YJU2	ORF-T	ORF-T	0.454872193	-0.447063048	-0.250061884	-0.283989218	0.103587687
262995	264019	11 +	YJU3	ORF-T	ORF-T	0.200257923	-0.541583106	-0.428631974	0.122887933	-0.025854289
264355	265619	11 +	MBR1	ORF-T	ORF-T	-1.787050294	-1.389677739	2.292694641	1.713214916	2.493272857
269323	271531	11 +	unknown	no_ovlp	SUTs	0.628675386	0.152180157	0.034926396	0.122060274	0.203270055
271531	272843	11 +	CUE2	ORF-T	ORF-T	0.398615084	-0.875195503	-0.338503643	-0.12843382	-0.459314394
272843	274915	11 +	MIF2	ORF-T	ORF-T	-0.324736611	-0.044159164	0.103087966	-0.431608526	0.848362262
274915	276811	11 +	YKL088W	ORF-T	ORF-T	0.207107719	-0.24743794	-0.325434631	0.104379205	-0.260949226
278139	278651	11 +	SRX1	ORF-T	ORF-T	-0.722955157	-0.08028973	1.864350491	0.493480923	1.387753472
278651	279995	11 +	MDH1	ORF-T	ORF-T	-0.634637479	-0.682772633	0.522670059	0.295469632	0.165735603
279995	280563	11 +	HOT13, YKL0	other	other	-0.015071115	0.109463536	-0.149189801	0.427200902	0.264958502
282507	282755	11 +	TEF4, SNR38	ORF-T	ORF-T	-0.645269901	-0.282114558	-0.719862107	-0.201452945	-0.371959806
284571	285931	11 +	VMA5	ORF-T	ORF-T	-0.231471965	-0.264754214	-0.3179728	0.096659392	-0.165043575
286187	288243	11 +	SMY1	ORF-T	ORF-T	0.005205095	-0.750133078	0.033362743	-0.264462213	-0.102074362
288475	290787	11 +	DHR2	ORF-T	ORF-T	0.770834638	-0.069537672	-1.119486898	-0.315837439	0.896419239
291043	292523	11 +	YKL077W	ORF-T	ORF-T	0.078700258	-0.363165151	-0.425386299	-0.097292036	-0.143517656
296035	298803	11 +	LHS1	ORF-T	ORF-T	-0.941099221	-0.104891551	-0.939548705	-0.388903818	0.242399719
299083	301667	11 +	STB6	ORF-T	ORF-T	0.164768441	-0.307459383	-0.393527936	-0.113235595	-0.350287081
302299	303875	11 +	unknown	no_ovlp2	SUTs	-0.262625899	0.589841084	0.959672296	0.379873589	-0.149479988
303875	306931	11 +	YKL071W	ORF-T	ORF-T	-0.077091751	1.511078281	1.522201169	0.217255959	0.257539042
305667	306931	11 +	unknown	no_ovlp2	SUTs	-0.046252571	0.536024449	0.237971842	0.005747649	0.326719408
306931	307443	11 +	YKL069W	ORF-T	ORF-T	0.106738859	-0.961067584	-0.388642976	-0.562324808	-0.253122248
308803	309419	11 +	YKL068W-A	ORF-T	ORF-T	0.42655042	-1.184276586	-1.233694784	-0.69904022	-0.024858884
309867	312835	11 +	NUP100	ORF-T	ORF-T	0.030506744	-0.237106404	-0.462365412	-0.189384695	-0.058623259
314347	315115	11 +	YNK1, YKL06	other	other	-0.174344866	-0.428160662	0.592457602	0.081407719	0.057812985
315115	317171	11 +	YKL066W, YK	other	other	0.214864246	0.293487341	1.883744719	0.580247633	0.111159334
316035	317171	11 +	unknown	no_ovlp2	SUTs	0.402560486	0.307921035	-0.156568491	-0.144944417	-0.353172386
317171	320499	11 +	MNR2	ORF-T	ORF-T	-0.181527264	-0.639259217	-0.287989204	0.079018994	-0.425914319
322267	322747	11 +	unknown	no_ovlp	SUTs	0.20913321	-0.300336172	0.117715792	-0.106260696	-0.464158816
322875	324579	11 +	MSN4	ORF-T	ORF-T	0.366042178	-0.573999359	-0.190473046	-0.139465429	-0.326346395
325395	326003	11 +	YKL061W	ORF-T	ORF-T	0.275292046	-0.360736585	0.214307454	-0.242794167	-0.579820545
327771	328891	11 +	CUT231	CUTs	CUTs	2.899112826	0.298283352	-1.45302204	-0.058315492	-0.393598939
329779	330419	11 +	TOA2	ORF-T	ORF-T	-0.585694543	-1.424564367	-0.500625074	0.131158301	-0.393743183
334171	334923	11 +	unknown	no_ovlp	SUTs	0.336757224	0.429827936	-0.311227387	0.158933276	-0.350545284
334923	335643	11 +	CUT232	CUTs	CUTs	3.541611872	0.349550124	-1.013664223	-0.707129132	0.06287441
338843	339419	11 +	unknown	no_ovlp	SUTs	0.961453349	-0.386417239	0.253081694	-0.142432904	0.117138304
340859	342419	11 +	SFK1	ORF-T	ORF-T	0.096405114	-1.364211282	-0.316335915	-0.341821983	-0.732947144
349099	350763	11 +	YKL047W	ORF-T	ORF-T	0.353921204	-0.679590236	-0.377695673	-0.214276491	0.296123342
353075	354851	11 +	PRI2	ORF-T	ORF-T	0.326935495	-0.044483587	-0.148820791	-0.303628147	-0.114885561
355899	356179	11 +	YKL044W	other	other	0.631474661	0.063059622	-0.413424154	-0.765879164	-0.330807567
356179	356707	11 +	YKL044W, PH	other	other	0.173301901	-0.629074932	-0.05238844	-0.075882827	0.205611737
357987	359355	11 +	SPC42	ORF-T	ORF-T	-0.715286806	-0.778655859	-0.291257114	-0.178817548	0.235505433
359715	360555	11 +	VPS24	ORF-T	ORF-T	-0.364330002	-0.654129371	0.090684679	0.200988516	0.210801353
362115	364092	11 +	PTM1	ORF-T	ORF-T	-0.234310762	-0.544202762	-0.150328476	-0.015620665	-0.497496813
364572	365172	11 +	unknown	no_ovlp	CUTs	4.105634412	-0.277985265	-0.442441145	-0.474681724	-0.036367654
369444	371196	11 +	UGP1	ORF-T	ORF-T	-0.652160995	-0.801155168	0.768654465	0.606336479	0.243657576
371388	373836	11 +	TUL1	ORF-T	ORF-T	-0.269666013	0.037261476	0.344453623	0.153679174	0.174432689
374052	374924	11 +	YKL033W-A	ORF-T	ORF-T	-0.388448523	-0.832193701	-0.441763643	-0.074171117	-0.819181585
375156	378628	11 +	YKL033W	ORF-T	ORF-T	0.000389078	-0.394545363	-0.302420698	-0.176787505	0.076487003
379284	379596	11 +	unknown	no_ovlp	CUTs	1.493158016	0.140640453	-1.097473971	-1.35225264	-0.226217894
380652	381140	11 +	unknown	no_ovlp2	SUTs	-0.347000489	0.451841404	0.122712684	0.118453431	0.349967964
381140	383300	11 +	YKL031W	other	other	0.219555845	0.441386771	-0.35115339	0.394558361	0.66768042
382012	383300	11 +	unknown	no_ovlp2	SUTs	0.119239577	0.31098007	-0.004651813	-0.026794552	0.100650917
385380	388660	11 +	TFA1	ORF-T	ORF-T	-0.124815153	-0.139379478	-0.177405414	-0.157945861	-0.092016068
386980	388660	11 +	unknown	no_ovlp2	SUTs	0.365431811	-0.690141276	-0.826083858	-0.339668188	-0.650862486
393348	394324	11 +	YKL023W	ORF-T	ORF-T	-0.283021335	-0.53670087	-0.120211454	-0.072854704	-0.33155055
402196	403236	11 +	RAM2	ORF-T	ORF-T	-0.112017159	-0.343661905	-0.382073652	-0.205738478	-0.376611389
403708	404876	11 +	SWD2	ORF-T	ORF-T	0.374360139	-0.380828999	-0.494239327	-0.35968125	-0.148773469
408100	411252	11 +	PUT3	ORF-T	ORF-T	0.319961195	-0.491213068	-0.507315021	-0.463645656	0.244096661
416836	417908	11 +	unknown	no_ovlp	SUTs	-0.216712492	0.275953409	-0.077577592	0.030859601	-0.287375097
417908	419804	11 +	PRP40	ORF-T	ORF-T	-0.126839363	-0.147227284	0.169778075	0.003149588	0.213251988
425844	426716	11 +	MRT4	ORF-T	ORF-T	0.403361857	-0.850771674	-0.855380227	-0.477399716	-0.136150061
428924	429884	11 +	CAP1	ORF-T	ORF-T	-0.104845303	-0.086041218	0.205654974	-0.344032629	0.354269738
431676	432364	11 +	RPL14A	ORF-T	ORF-T	0.874072607	-0.731197332	-1.356978856	-1.368931263	-0.366356516
432476	434876	11 +	unknown	no_ovlp	SUTs	0.350656259	0.484038684	-0.018147665	-0.243998572	1.129347847
434876	436572	11 +	AUR1	ORF-T	ORF-T	-0.20645292	-0.359768345	-0.3788586	-0.065148393	0.35815098
437564	438420	11 +	DID4	ORF-T	ORF-T	-0.344167508	-0.431319643	0.059749282	-0.071653545	0.126984966

439420	439748	11	+	CUT238	CUTs	CUTs	1.705547885	0.107116347	0.538778336	0.591950025	-0.47924207
442668	444676	11	+	PAP1	ORF-T	ORF-T	-0.496477735	-0.657300888	-0.112759956	0.077579149	-0.033431904
444932	446452	11	+	OSH6	ORF-T	ORF-T	-0.130978415	-0.041752486	0.101988361	-0.333749662	0.084555707
451076	451700	11	+	MEH1	ORF-T	ORF-T	-0.140682976	-0.058829756	0.153198823	-0.077029163	0.133245307
451828	453820	11	+	RSC4	ORF-T	ORF-T	0.006524359	-0.45860088	-0.395450366	-0.101455827	-0.138494741
454532	454996	11	+	CUT239	CUTs	CUTs	3.157372271	-0.269385606	0.017961756	0.342114463	-2.593519401
461260	462356	11	+	SUT237	SUTs	SUTs	2.320012305	0.247870094	-2.029581286	-0.5885989	-1.02759433
463356	463604	11	+	PRY2	ORF-T	ORF-T	2.24773572	-0.414573526	-1.226977221	-0.015827369	-0.811046042
466076	467468	11	+	SUT238	SUTs	SUTs	-0.071459642	-0.834598835	-0.214783486	-0.04971285	-0.376234221
469324	471220	11	+	FMP13	ORF-T	ORF-T	-0.314686199	-0.34141103	0.74841049	0.212380095	0.42086079
475732	476084	11	+	CUT240	CUTs	CUTs	2.686152645	-0.221101766	-0.447922641	-0.387363519	-0.360415933
477940	478700	11	+	VPS51	ORF-T	ORF-T	-0.134689011	-0.38542234	0.268246088	-0.260419925	-0.087516016
478820	481820	11	+	ALY1	ORF-T	ORF-T	0.492524092	-0.1696511	0.038267349	-0.217760553	-0.092622169
483044	484716	11	+	YKR023W	ORF-T	ORF-T	-0.140433392	-0.357251195	-0.304224367	-0.254004698	-0.016971843
487388	488196	11	+	RPC37	ORF-T	ORF-T	0.170773369	-0.169778331	-0.421745396	-0.223276942	0.60470231
489564	490036	11	+	CUT241	CUTs	CUTs	3.56966318	0.047109429	-0.384988933	-0.414582509	-1.19454826
490988	493388	11	+	BCH2	ORF-T	ORF-T	-0.170308907	-0.673542293	-0.392079091	-0.144765419	-0.377847226
493524	497132	11	+	SAP190	ORF-T	ORF-T	0.106273892	-0.646502222	-0.531421976	-0.147985387	-0.499273178
499316	499884	11	+	unknown	no_ovlp	SUTs	-0.279137897	0.456279503	-0.013273796	-0.102543104	0.410107008
499884	500860	11	+	GMH1	ORF-T	ORF-T	-0.338528778	-0.959505361	-0.284735877	0.031818553	-0.111191412
506332	506540	11	+	unknown	no_ovlp2	CUTs	1.151757664	-1.558972449	-0.173408209	-0.709286918	-2.292254852
506540	507428	11	+	DAL80	ORF-T	ORF-T	-0.17119487	-0.622205565	0.305092613	-1.254017076	-2.531404564
507564	508340	11	+	DID2	ORF-T	ORF-T	-0.185647954	-0.583584087	0.102221545	-0.172637144	0.200023992
510404	511492	11	+	CUT242	CUTs	CUTs	1.070222603	0.327186101	-0.373166031	-0.202909112	0.802038288
513860	514620	11	+	SUT239	SUTs	SUTs	2.151451386	0.528741506	-0.46087171	-0.716164543	-1.060475145
514620	516668	11	+	GAP1	ORF-T	ORF-T	-0.715843789	-0.440431767	-0.453512349	-0.291182853	-0.320700569
516668	517836	11	+	unknown	no_ovlp2	SUTs	-0.391651836	0.300187664	0.197575497	0.250348432	0.315262322
517836	518956	11	+	YKR041W	ORF-T	ORF-T	-0.956604424	-0.100615676	0.071557376	0.196593033	-0.185601317
518956	520500	11	+	UTH1	ORF-T	ORF-T	-0.202542903	-0.031923547	0.158324015	0.121451468	-0.071342632
521580	523060	11	+	UIP5	ORF-T	ORF-T	0.61304444	-0.382038224	-0.843453707	-0.20355872	0.013791441
523868	525420	11	+	YKR047W	other	other	-0.087537231	0.302587598	-0.419118356	-0.034744395	0.202747225
524932	525420	11	+	unknown	no_ovlp2	CUTs	2.414510528	0.251456198	-0.39468456	0.282527307	0.028758823
526508	530196	11	+	TRK2	ORF-T	ORF-T	-0.223029391	-0.034912212	0.039970346	-0.072096595	0.415823451
530484	531948	11	+	YKR051W	ORF-T	ORF-T	-0.545453132	-0.626744858	0.128291005	-0.008786394	-0.29763577
533252	534628	11	+	unknown	no_ovlp	CUTs	1.070853564	-0.380545716	-0.00088302	0.305094828	-0.400215914
547820	549004	11	+	RHO4, TRM2	ORF-T	ORF-T	0.015212773	0.165171695	0.564364473	-0.000265165	0.768592869
549212	551068	11	+	TRM2	ORF-T	ORF-T	0.058949047	-0.305655058	-0.100707556	0.162995552	0.803792901
551324	551652	11	+	RPS21A	ORF-T	ORF-T	0.715155847	-0.108650411	-0.868619437	-1.096176678	-0.041468042
552356	554332	11	+	GLG1	ORF-T	ORF-T	-0.27718399	-1.276962302	0.005273689	0.498561129	-0.994753462
554556	556092	11	+	TIF1	ORF-T	ORF-T	-0.288553989	-1.212530727	-0.693870758	0.434321575	-0.425087261
556092	557052	11	+	unknown	no_ovlp2	SUTs	0.749192735	-0.328261956	-0.627224611	-0.247013754	0.69451042
557052	558636	11	+	KTR2	ORF-T	ORF-T	0.41930332	-0.659018428	-0.284231158	-0.201662259	-0.986839482
559260	560444	11	+	TFA2	ORF-T	ORF-T	-0.425805735	-0.370520345	-0.002534155	0.263423056	0.322497616
562164	564844	11	+	OAF3	ORF-T	ORF-T	-0.127396486	-0.343284101	-0.194309104	-0.138649528	0.337329769
565764	566164	11	+	unknown	no_ovlp	CUTs	2.365545998	0.041991665	-1.160918666	-0.016458828	-0.329883239
567476	569940	11	+	GPT2	ORF-T	ORF-T	-0.407504314	-0.545902823	0.565774253	0.47238183	-7.23E-05
571100	573172	11	+	MET1	ORF-T	ORF-T	0.329922809	0.083207281	-0.058022383	-0.293034989	0.089979714
573188	574484	11	+	YKR070W	ORF-T	ORF-T	-0.417947726	-0.65477074	-0.16936624	-0.110118633	-0.205030139
575916	576372	11	+	unknown	no_ovlp	CUTs	1.334296509	-0.490295361	0.443641423	0.127887842	0.71072008
578812	579436	11	+	YKR074W	ORF-T	ORF-T	-0.499093976	-1.273689234	-0.281329594	0.105346993	-0.040710898
580068	580460	11	+	YKR075W-A	other	other	2.171733796	0.26196525	-0.668407362	0.082832322	0.001657562
581004	581820	11	+	unknown	no_ovlp2	CUTs	1.266410792	-0.57399627	0.244256986	0.044622603	-1.228691075
581820	583068	11	+	ECM4	ORF-T	ORF-T	-0.286227034	0.418111102	1.818413128	1.053164014	-0.64729489
583276	584436	11	+	MSA2	ORF-T	ORF-T	-0.446897992	-0.23316762	0.553673698	0.133938543	1.077775545
584572	586372	11	+	YKR078W	ORF-T	ORF-T	0.123268253	-0.253851353	-0.065535432	-0.20362797	0.199091686
589908	591132	11	+	MTD1	ORF-T	ORF-T	1.023358703	0.266055727	-0.202096826	-0.432411807	-0.321958942
592436	596012	11	+	NUP133	ORF-T	ORF-T	0.283007851	-0.197453827	-0.378269013	-0.063595433	0.206401619
599484	600172	11	+	PRP16	ORF-T	ORF-T	0.097841125	-0.187278978	-0.363210134	-0.331933106	-0.008363917
605269	605725	11	+	SUT241	SUTs	SUTs	-0.593378287	-0.572550237	0.204768009	1.481267203	2.05993502
608509	610901	11	+	PXL1	ORF-T	ORF-T	-0.929941422	-0.543352182	-0.281611658	0.189094134	0.428028847
611133	612038	11	+	SRL3	ORF-T	ORF-T	-0.081773698	-0.333298256	0.500629106	0.140325753	0.340958353
614630	615286	11	+	unknown	no_ovlp	SUTs	0.737557138	-0.160702191	0.130641129	0.039953114	-0.950224135
615286	617190	11	+	PTR2	ORF-T	ORF-T	-0.769822958	-1.659746844	-0.203119689	-0.249509721	-0.174790445
619294	621278	11	+	MLP1	ORF-T	ORF-T	0.059518766	-0.554122849	-0.658234209	-0.096610906	-0.287602693
625630	625966	11	+	PCC1	ORF-T	ORF-T	-0.149935284	-0.398484028	0.092601934	-0.20231138	0.006687615
626222	630134	11	+	YKR096W	ORF-T	ORF-T	-0.070152524	-0.467559453	0.203608363	0.055489219	-0.419292428
630134	630654	11	+	unknown	no_ovlp2	SUTs	-0.426611049	-0.021184985	0.484480641	0.105203769	0.074655724
630654	631590	11	+	PCK1	ORF-T	ORF-T	-0.465758704	1.319721781	3.343461337	1.341795942	-0.029949302
631590	632646	11	+	PCK1	ORF-T	ORF-T	-0.03525729	0.35888227	2.145097845	1.242510722	-1.336927502
635350	637942	11	+	BAS1	ORF-T	ORF-T	0.120203167	-0.532635065	-0.15913765	-0.281829744	0.191243258
640190	641934	11	+	SIR1	ORF-T	ORF-T	-0.218533894	-0.437415537	0.33362338	-0.301584774	-1.360377255
642622	646966	11	+	FLO10	ORF-T	ORF-T	0.350793718	-0.046413296	0.146659159	-0.14801847	-1.560299764
650118	652702	11	+	NFT1	ORF-T	ORF-T	0.438856418	-0.453415062	-0.476180985	-0.357833284	-1.163652841
652702	653190	11	+	unknown	no_ovlp2	SUTs	0.254018546	-0.510867116	-0.010842865	-0.313474915	-1.647305988
653190	657622	11	+	NFT1, YKR10	ORF-T	ORF-T	0.488513436	0.202558557	-0.284867105	-0.348294328	-1.095670755
14557	16021	12	-	AYT1	ORF-T	ORF-T	0.358551406	-0.752919082	-0.488514258	-0.503314525	-2.625524642
16021	16517	12	-	unknown	no_ovlp2	SUTs	0.411633913	0.327916148	0.182827116	0.122611027	-0.446946377
16517	20917	12	-	MHT1	ORF-T	ORF-T	0.008993242	0.487371278	0.586114074	0.476831636	-0.549736244
17741	20917	12	-	unknown	no_ovlp2	SUTs	-0.791142692	0.377349291	1.345354582	0.656986849	0.304130762
20917	22685	12	-	GTT2, YLL05	other	other	0.728802067	0.523633641	0.122383807	-0.246243576	0.372594978
27277	29645	12	-	YLL056C	ORF-T	ORF-T	-0.01442508	-0.123363279	0.334305615	0.031979587	0.127745114
32325	35245	12	-	YLL054C	ORF-T	ORF-T	0.305370131	0.190110043	-0.313465452	-0.091841651	-0.686670386

37173	39541	12	-	FRE6	ORF-T	ORF-T	0.029077264	-0.652222463	-0.432004454	-0.161687017	-0.47210185
40221	40437	12	-	COF1	ORF-T	ORF-T	-0.803021159	-2.098385065	-1.012279331	-0.357619802	-0.326213514
41189	46453	12	-	YBT1	ORF-T	ORF-T	0.17142101	-0.301301572	-0.671254922	-0.037464368	-0.323151574
47757	48693	12	-	RPL8B	ORF-T	ORF-T	-0.334400656	-0.184094562	-0.315393114	-0.248862948	0.166061912
48693	49653	12	-	unknown	no_ovlp	SUTs	0.703837795	-0.163301052	0.502631566	0.638661063	1.106933413
52053	52605	12	-	ATG10	ORF-T	ORF-T	0.981235223	0.14617417	0.397393595	-0.811829435	-0.511859363
52773	53981	12	-	SDH2	ORF-T	ORF-T	-0.380730948	0.240166738	1.252547685	0.247118934	0.831125591
58925	63749	12	-	VPS13	ORF-T	ORF-T	-0.204865327	-0.231083279	0.134260272	0.325177967	-0.018266978
63933	65309	12	-	UBI4	ORF-T	ORF-T	-0.089521785	-0.516888434	0.596902752	0.355934132	0.476958572
65309	66525	12	-	ENT4	ORF-T	ORF-T	-0.131660885	-0.042298008	-0.22515106	0.05902068	-0.439922141
66701	68261	12	-	PRP19	ORF-T	ORF-T	0.017977084	-0.098470357	-0.04731051	0.025710721	0.14359124
70517	73189	12	-	RIX7	ORF-T	ORF-T	0.607900128	-0.058432449	-1.132578309	-0.355511918	0.069179069
73957	76821	12	-	YLL032C	ORF-T	ORF-T	-0.257940077	-0.661482722	0.490793338	-0.333848201	0.347446584
77069	80261	12	-	GPI13	ORF-T	ORF-T	-0.297834367	-0.355081898	-0.265811322	-0.287726734	0.735715406
81389	83957	12	-	unknown	no_ovlp	SUTs	0.868424695	0.289524404	0.002323049	0.385939489	-0.102648566
95429	97557	12	-	SSA2	ORF-T	ORF-T	-0.356557582	-0.475052783	-0.716940368	-0.105838318	-0.481859414
97861	98885	12	-	YLL023C	ORF-T	ORF-T	-0.32666229	-0.054105169	0.594981378	0.360117024	0.710801873
98981	100573	12	-	HIF1	ORF-T	ORF-T	-0.733105254	-0.299761208	-0.00343939	0.057048746	0.467420149
108429	109021	12	-	YLL020C, KN!	ORF-T	ORF-T	0.329439304	0.001500919	0.076971969	-0.082778863	0.346976716
109741	111645	12	-	DPS1	ORF-T	ORF-T	-0.193493332	-0.165047584	-0.468456389	0.047005587	-0.482025416
112173	114285	12	-	SUT686	SUTs	SUTs	2.608440678	1.791385071	-0.006282797	0.160660168	-2.452223726
115613	116125	12	-	CUT730	CUTs	CUTs	1.878066044	0.076645301	-0.014261627	0.320726187	0.179722784
124621	125005	12	-	PUF3	ORF-T	ORF-T	-0.448395178	-0.672141799	-0.485717193	-0.171743973	-0.904014641
129141	130853	12	-	PSR1	ORF-T	ORF-T	-0.231021429	-0.687517273	-0.309077072	0.040499148	-0.352657195
131093	131461	12	-	COX17	ORF-T	ORF-T	-0.329630115	-0.33208963	0.433319524	-0.205891625	-0.226019289
134125	136381	12	-	YLL007C	ORF-T	ORF-T	0.338610281	-0.235942624	0.014295973	-0.199674892	-0.406359568
146037	147701	12	-	unknown	no_ovlp	SUTs	0.286375541	0.416246421	0.068626701	-0.09866943	0.153971824
151325	153989	12	-	YLR001C	ORF-T	ORF-T	-0.410435891	-0.31359083	0.293449179	0.212631266	0.042123512
154245	156365	12	-	NOC3	ORF-T	ORF-T	0.414433298	-0.332303311	-0.830981378	0.045599336	-0.069237672
156757	157765	12	-	YLR003C	ORF-T	ORF-T	0.266710296	-0.340684587	-0.121940912	0.009024894	0.187056113
157765	161709	12	-	THI73	ORF-T	ORF-T	-0.765721921	0.28452052	0.696386031	0.01053921	-0.314638512
159813	161709	12	-	unknown	no_ovlp2	SUTs	0.284037582	0.290293759	-0.164290187	-0.17223911	0.821665948
161709	164061	12	-	SSK1	ORF-T	ORF-T	0.018780427	-0.513099581	-0.646213445	0.001470527	-0.3068352
165317	166189	12	-	PAM18	ORF-T	ORF-T	-0.404431841	-0.721658884	-0.907426712	-0.463548156	-0.134073498
167141	167805	12	-	TEN1	ORF-T	ORF-T	0.632898219	-0.506410851	0.407469522	-0.089879157	0.35208613
172053	174909	12	-	PPR1	ORF-T	ORF-T	0.55345287	0.361589087	-0.292484582	-0.177262676	0.049488129
176653	177445	12	-	PML1	ORF-T	ORF-T	-0.023116616	-0.549857716	-0.102781147	0.13207185	0.214353511
178725	179653	12	-	POM34	ORF-T	ORF-T	-0.587238487	-0.556952123	-0.306388152	0.026236184	0.114780198
181621	183437	12	-	YEH2	ORF-T	ORF-T	0.063362723	-0.085285726	-0.543275519	-0.330297227	-0.101670816
184117	184933	12	-	SDO1	ORF-T	ORF-T	0.785876619	-0.522255193	-0.579265147	-0.188790736	-0.290164865
185101	187341	12	-	IZH3	ORF-T	ORF-T	0.109251402	-0.776192906	-0.531084905	-0.259896471	-0.077122734
187341	193461	12	-	UBR2	ORF-T	ORF-T	0.124546848	-0.012332892	-0.079617634	-0.126608531	0.397692348
195781	196581	12	-	SED5	ORF-T	ORF-T	-0.187422008	-0.179937583	-0.233211744	-0.193107038	0.117872829
196797	198117	12	-	AAT2	ORF-T	ORF-T	0.058199579	-0.129739421	-0.521810869	-0.181364377	-0.494793194
199453	201437	12	-	ADE16	ORF-T	ORF-T	-0.20601767	-0.354670614	-0.114892797	-0.097748093	-0.44693795
201869	202621	12	-	RPL15A	ORF-T	ORF-T	-0.425786413	-0.20383476	-0.300629126	-0.139640692	-0.068043611
203189	204725	12	-	SUT687	SUTs	SUTs	0.418525335	-0.832554991	-0.984305529	-0.456084565	-0.229158413
210389	212125	12	-	SMF3	ORF-T	ORF-T	0.064459305	-0.633959666	-0.625007584	0.241889966	-0.79299548
212125	214725	12	-	MLH2	ORF-T	ORF-T	0.459135947	-0.04030521	0.208202237	0.016982787	-0.889586189
214493	214725	12	-	unknown	no_ovlp2	CUTs	2.361404291	0.146985638	0.226575355	0.408140841	0.14219602
221317	222165	12	-	YLR036C	ORF-T	ORF-T	-0.405477892	-1.190093241	-0.093284936	0.11396405	-0.4091535
222165	222981	12	-	unknown	no_ovlp	SUTs	-0.375485984	0.399287926	0.162853945	0.386672847	0.259538845
224613	225253	12	-	COX12	ORF-T	ORF-T	-0.242578243	-0.635588259	0.464475981	-0.202472135	-0.181044546
225373	228501	12	-	RIC1	ORF-T	ORF-T	0.064622099	-0.474562676	-0.278780221	-0.532869185	-0.054068601
228789	229693	12	-	YLR040C	ORF-T	ORF-T	3.039327177	1.559199867	-1.671055442	-1.069767338	-5.196955372
230101	231093	12	-	YLR042C	ORF-T	ORF-T	1.753821721	0.387278913	0.205879811	-0.621652421	-2.541193905
231597	232053	12	-	TRX1	ORF-T	ORF-T	-0.337760737	-0.664923526	-0.395140962	-0.782195632	0.081265262
232333	234133	12	-	PDC1	ORF-T	ORF-T	-0.41648493	-0.195455261	-0.069035626	-0.076639096	-0.192460737
234845	237717	12	-	STU2	ORF-T	ORF-T	-0.237793803	-0.233639483	0.147106488	-0.124685241	0.620254688
238005	241693	12	-	YLR046C, FRI	other	other	-0.285015112	0.079010959	0.139841436	-0.201858144	0.768678182
243781	245357	12	-	YLR049C	ORF-T	ORF-T	-0.001949153	-0.336534667	0.131243321	0.113241621	0.600521042
245493	246237	12	-	unknown	no_ovlp2	SUTs	0.54320738	0.008770331	-0.473189927	-0.257279416	0.401456809
246237	246997	12	-	FCF2	ORF-T	ORF-T	0.256119732	-0.47369186	-0.351959678	-0.230948618	0.634859626
248285	249173	12	-	unknown	no_ovlp	SUTs	-0.858603446	-0.272151319	1.899748588	0.780663824	-0.585853731
251237	253085	12	-	SPT8	ORF-T	ORF-T	-0.418875611	-0.432181355	-0.564833208	-0.084206559	-0.163056244
257853	259453	12	-	SHM2	ORF-T	ORF-T	0.823912473	0.166808037	-0.329127122	-0.22604571	-0.537179705
259453	260549	12	-	REX2	ORF-T	ORF-T	0.574309318	0.497360278	-0.285966744	-0.425581547	0.271193173
264733	265261	12	-	CUT733	CUTs	CUTs	3.92181124	0.514446044	-0.802678971	-0.046300975	-0.208812135
266269	266949	12	-	YLR065C	ORF-T	ORF-T	0.135575449	-0.459534023	-0.453584066	-0.218210101	0.157766127
267821	270797	12	-	PET309	ORF-T	ORF-T	-0.430082242	-0.591234898	-0.172730283	-0.480338754	0.191602347
271501	273925	12	-	MEF1	ORF-T	ORF-T	-0.72629	-0.662574663	-0.152168773	-0.130818792	-0.242816761
273925	275261	12	-	XYL2	ORF-T	ORF-T	-1.770186088	-0.284671952	2.045801293	0.83056581	0.121570976
275261	278653	12	-	RGR1	ORF-T	ORF-T	-0.025799128	-0.293391409	-0.289156738	0.280903421	-0.221575826
280949	282501	12	-	YLR073C	ORF-T	ORF-T	0.288764992	-0.237523172	-0.115799242	-0.292491201	0.52468041
281733	282501	12	-	unknown	no_ovlp2	SUTs	0.439087842	0.025081205	-0.306804956	-0.613073395	0.204308383
282501	282973	12	-	unknown	no_ovlp	SUTs	-0.217163768	0.045476749	-0.111247761	-0.119289694	-0.111159187
286429	286645	12	-	BOS1	ORF-T	ORF-T	-1.245334545	-0.743917469	0.042837216	0.298187017	0.546950053
292109	293629	12	-	SRL2	ORF-T	ORF-T	0.248379024	-0.624855094	-0.566396486	-0.008625072	-1.614592955
293973	296197	12	-	EMP70	ORF-T	ORF-T	-0.448649392	-0.652419799	-0.753549743	-0.058786624	-0.076873358
297749	300397	12	-	RAX2	ORF-T	ORF-T	0.019695041	-0.343390409	-0.562753333	-0.338682104	0.291905851
300565	302005	12	-	ARP6	ORF-T	ORF-T	0.09017863	0.006225279	0.191276645	-0.025579388	-0.2192032
313557	315933	12	-	CSF1	ORF-T	ORF-T	-0.10710771	-0.115622294	-0.397014592	-0.146831693	0.056281615

315933	318045	12 -	unknown	no_ovlp	SUTs	0.157344988	0.349724382	-0.037586823	-0.170420543	-0.179587224
318165	320357	12 -	ALT1	ORF-T	ORF-T	0.61051671	-0.562964069	-0.263559218	0.126096969	-0.518153876
322277	325157	12 -	SUT693	SUTs	SUTs	0.654044422	0.200959634	0.146203588	0.046013447	-0.508094612
326285	327229	12 -	NYV1	ORF-T	ORF-T	-0.406245594	-0.704277054	0.267353885	0.20208679	0.021732974
327621	329301	12 -	GIS3	ORF-T	ORF-T	0.090225314	-0.404636001	-0.377089054	-0.015356446	-0.085421058
330629	332165	12 -	IOC2	ORF-T	ORF-T	0.187896971	-0.187191259	-0.70842967	-0.267663779	-0.314048846
336133	337373	12 -	unknown	no_ovlp2	SUTs	0.398700307	-0.274362348	0.15706271	0.320490449	-0.257915096
337373	340005	12 -	CHA4	ORF-T	ORF-T	0.259370674	-0.46319698	-0.229193404	-0.17737544	-0.259307053
339541	340005	12 -	unknown	no_ovlp2	SUTs	-0.044705851	-0.381726775	-0.239750355	0.150770604	0.10489965
340005	341557	12 -	unknown	no_ovlp	SUTs	0.097358533	-0.071077796	-0.340447846	0.058030303	-0.168640021
342845	343781	12 -	YLR101C, AP	other	other	-0.087339017	-0.406024999	0.452479316	-0.309892418	0.537860917
343957	345997	12 -	CDC45	ORF-T	ORF-T	-0.190198483	-0.134907426	-0.163088783	-0.213169139	0.924503283
348221	348445	12 -	unknown	no_ovlp	CUTs	4.040469611	-0.371040419	-0.109213191	-0.19093719	-0.021398835
359813	363845	12 -	MDN1	ORF-T	ORF-T	0.270617681	-0.215955803	-0.612791393	0.157362022	0.239417266
363989	364293	12 -	CUT734	CUTs	CUTs	2.986857158	-0.14882259	0.204517415	0.424070984	0.144112673
366533	368285	12 -	YLR108C	ORF-T	ORF-T	0.070950878	-0.074028698	0.829365473	0.638929539	0.563259075
369549	370141	12 -	CCW12	ORF-T	ORF-T	-0.557753989	-0.499689503	0.038167333	-0.075737414	0.006763823
370141	370381	12 -	SUT695	SUTs	SUTs	0.823371791	-0.44211509	-0.162887451	-0.060713777	-0.871424252
370381	371021	12 -	SUT695	SUTs	SUTs	-0.020078278	0.125720155	-0.240452981	0.068379998	-0.605897647
374541	377301	12 -	AVL9	ORF-T	ORF-T	-0.041680041	-0.399535038	-0.389485047	-0.351757744	-0.457180151
382349	384869	12 -	CLF1	ORF-T	ORF-T	0.047828228	-0.487392438	0.061432338	0.178215336	-0.42270267
384573	384869	12 -	unknown	no_ovlp2	SUTs	0.021997262	-0.971756876	0.150384016	-0.140419035	-0.082721698
384869	385445	12 -	YLR118C	ORF-T	ORF-T	-0.031393442	-0.256871082	0.166413895	0.077757364	0.108188202
385445	386109	12 -	unknown	no_ovlp	SUTs	-0.094666692	0.109601664	0.038908029	0.086588503	-0.207801216
386173	388325	12 -	YPS1	ORF-T	ORF-T	0.06691161	-0.396507299	-0.242275561	-0.015766231	-0.188307787
388597	390293	12 -	YPS3	ORF-T	ORF-T	0.604100737	0.714683737	-0.066726511	0.045803515	-0.53272512
390293	390733	12 -	unknown	no_ovlp2	SUTs	0.42652852	0.419620005	0.50054073	0.481892042	-0.775072543
390733	393261	12 -	YLR122C, YLI	other	other	0.54740728	0.180037855	0.052165421	0.446610377	-1.013776631
391765	393261	12 -	unknown	no_ovlp2	SUTs	0.104432837	-0.728467634	0.255183759	0.656565922	-0.919474397
394725	395645	12 -	unknown	no_ovlp2	SUTs	-0.480013518	-0.779706923	-0.683257474	-0.282900205	-1.937925864
395645	398381	12 -	APC2	ORF-T	ORF-T	0.04103074	0.009194638	0.002625817	-0.014757095	-0.447880142
398381	399685	12 -	CUT735	CUTs	CUTs	0.37546838	0.298026253	-0.057071144	0.113258103	0.514725656
402741	404181	12 -	ZRT2	ORF-T	ORF-T	0.136869945	-0.903842523	-1.110746753	-0.124841447	-0.646934966
405701	408221	12 -	ACE2, YLR13	other	other	-0.347880261	-0.460792414	-0.141004133	-0.179358524	0.382053075
414557	415477	12 -	TIS11	ORF-T	ORF-T	1.878873198	-0.712599376	-1.426754924	-0.310753505	-0.279432032
415477	418077	12 -	unknown	no_ovlp	SUTs	0.842233465	0.043113958	-0.199116652	0.151400774	-0.118810954
421461	423933	12 -	SLS1	ORF-T	ORF-T	-0.574175891	-0.911638211	-0.34784134	-0.135925853	-0.037989708
423933	424677	12 -	unknown	no_ovlp	SUTs	0.360051542	0.409858335	0.445272358	-0.12142088	-0.602042624
429581	432021	12 -	ACF2	ORF-T	ORF-T	-0.195596003	-0.336015925	-0.141524715	-0.069969646	0.180379984
432741	433765	12 -	SPE4	ORF-T	ORF-T	0.37029322	-0.203029714	-0.506819561	-0.032092866	0.22417011
434061	434493	12 -	SMD3	ORF-T	ORF-T	0.346729179	-0.043201554	0.179227219	-0.11884752	0.309980162
437477	439885	12 -	YLR149C	ORF-T	ORF-T	-0.342321542	-0.540989219	1.595356761	1.034563357	0.872592243
439885	441349	12 -	unknown	no_ovlp2	SUTs	-0.010450335	0.600867838	-0.013762463	0.300548853	0.149345017
441349	442821	12 -	PCD1	ORF-T	ORF-T	-0.585687981	0.390164254	1.807177431	0.850979257	1.458610992
442821	444797	12 -	YLR152C	ORF-T	ORF-T	-1.101221744	-1.522279754	0.181489526	0.202173582	-0.390275177
445245	447653	12 -	ACS2	ORF-T	ORF-T	-0.309396838	-0.24691299	-0.333996176	0.074158536	-0.143466381
447941	448373	12 -	RNH203	ORF-T	ORF-T	0.125296586	0.108278816	0.44440664	-0.360414485	0.096395692
489877	490549	12 -	unknown	no_ovlp	SUTs	-0.323087499	-0.030012365	-0.543706497	-0.012570612	-0.142717086
491765	493285	12 -	MAS1	ORF-T	ORF-T	-0.695717204	-0.49305633	0.264547893	0.064188122	0.02501233
494525	495229	12 -	PUS5	ORF-T	ORF-T	-0.420761134	-0.917454212	0.473887888	-0.033900223	0.074345231
495397	498069	12 -	SEC10	ORF-T	ORF-T	0.361598959	-0.251760573	-0.234338853	-0.12912457	-0.002036241
499469	500277	12 -	YLR168C	ORF-T	ORF-T	0.286686541	-0.283222638	-0.222636223	-0.600830091	0.082948319
500277	501157	12 -	APS1	ORF-T	ORF-T	0.236159016	0.278316643	0.039633436	-0.07534223	-0.361788455
501157	502165	12 -	DPH5	ORF-T	ORF-T	0.776977009	0.081127661	-0.710114127	-0.156546252	-0.340732765
503373	504053	12 -	CUT737	CUTs	CUTs	1.963697731	0.434618061	-0.657621536	0.047460215	0.122009003
505317	505813	12 -	CUT739	CUTs	CUTs	3.732909723	0.334993047	-0.956263013	-0.583646021	0.044055959
507725	510757	12 -	RFX1	ORF-T	ORF-T	-0.287687864	-0.634830709	0.422257831	0.285261744	0.256067947
510189	510757	12 -	unknown	no_ovlp2	SUTs	0.522109953	0.177182802	0.64235758	1.194120078	0.519336794
513077	513885	12 -	TFS1	ORF-T	ORF-T	-0.265520581	-0.334344518	1.760404554	1.10816918	0.756988986
514021	514797	12 -	YLR179C	ORF-T	ORF-T	0.112055766	-0.007509168	-0.578043944	-0.223171968	0.059605068
516557	517741	12 -	VTA1	ORF-T	ORF-T	-0.430054475	-0.103234621	0.69122367	-0.05421601	1.073276099
520437	522061	12 -	TOS4	ORF-T	ORF-T	-0.982850091	-0.601649239	-0.246664292	-0.191575265	1.23647569
522637	523285	12 -	unknown	no_ovlp	CUTs	1.339610236	0.411911775	-0.945651652	-0.225842507	0.503055911
530693	534445	12 -	ATG26	ORF-T	ORF-T	0.006508326	0.352684814	0.521497197	-0.100527528	1.042252769
538549	539653	12 -	HCR1	ORF-T	ORF-T	-0.156173072	-0.628980492	-0.787024555	0.218476616	-0.741452871
539853	540581	12 -	UPS1	ORF-T	ORF-T	-0.550338597	-0.903518487	-0.434514564	-0.084420025	-1.238520352
540773	541581	12 -	YLR194C	ORF-T	ORF-T	0.017701412	0.068000462	0.206856597	0.097746786	0.417221063
541581	541853	12 -	unknown	no_ovlp2	SUTs	-0.155955756	-0.731289836	-0.587606736	0.189666846	-0.047623172
541853	543317	12 -	NMT1	ORF-T	ORF-T	-0.557671254	-0.408750393	-0.001659651	0.105364831	-0.307408641
547733	548677	12 -	YLR198C, PB	other	other	0.172620365	-0.405053591	0.318991022	0.046417623	0.374848437
549461	550341	12 -	COQ9, YLR20	other	other	-0.210074707	-0.958895856	0.023202863	0.093783343	-0.003210371
550605	552069	12 -	YLR202C, MS	other	other	-0.876304938	-0.620553366	0.220050278	0.090666573	0.303270856
553773	554221	12 -	HMX1	ORF-T	ORF-T	0.183905811	-0.135009279	0.054587156	-0.089566115	0.212946524
560725	561757	12 -	PNP1	ORF-T	ORF-T	-0.24760526	-0.279863395	0.149674896	-0.233069133	0.558077516
563485	564453	12 -	YLR211C	ORF-T	ORF-T	-0.244691504	-0.24653085	0.585942197	0.013539138	0.703505375
564741	566309	12 -	TUB4	ORF-T	ORF-T	0.229237365	-0.070737499	-0.252572783	-0.496826961	0.746048537
566309	567909	12 -	CRR1	ORF-T	ORF-T	-0.658014262	-0.054302084	0.65899633	0.43830172	0.190055643
570677	571869	12 -	CDC123	ORF-T	ORF-T	0.519315575	-0.24479554	-0.512870169	-0.224909079	0.156562164
572013	573245	12 -	CPR6	ORF-T	ORF-T	0.235384824	0.064423903	0.540676468	0.080705563	-0.04790282
573445	573781	12 -	YLR218C	ORF-T	ORF-T	-1.338553556	-0.966812036	-0.079761051	-0.397273497	0.572804497
578141	579077	12 -	RSA3	ORF-T	ORF-T	0.573165736	-0.195858948	-0.599850151	-0.18700198	0.335360334
579221	581805	12 -	UTP13	ORF-T	ORF-T	0.465474281	-0.215139208	-0.763365614	-0.095595736	0.200419838

582117	585821	12 -	YLR222C-A, I	ORF-T	ORF-T	0.48281188	-0.163500333	-0.287690249	-0.309117208	0.445942075
587613	588997	12 -	YLR225C	ORF-T	ORF-T	0.417092311	-0.311693586	-0.111218083	-0.229535655	-0.579967893
590469	592405	12 -	ADY4	ORF-T	ORF-T	0.454179039	-0.775196195	-0.431661232	-0.399842187	-0.928188733
599917	602677	12 -	YLRCtau1, EC	other	other	-0.385386748	-0.425831152	0.124684857	0.034714022	-0.115705002
603293	603837	12 -	SUT698	SUTs	SUTs	1.238725758	1.434563427	0.397542009	0.44109164	-1.511584709
604125	605021	12 -	CDC42	ORF-T	ORF-T	-0.423096083	-0.346399968	-0.011703437	0.064021015	0.224911534
605701	607197	12 -	BNA5	ORF-T	ORF-T	0.405171273	0.640165217	-0.144909844	0.372142293	-2.975551031
607197	611381	12 -	EST1	ORF-T	ORF-T	0.224422755	-0.381158445	0.070764821	0.425567129	0.632266378
609573	611381	12 -	unknown	no_ovlp2	SUTs	0.862841377	0.20643505	-0.136173284	1.460617989	1.547002751
616237	617333	12 -	LIP2	ORF-T	ORF-T	0.004616719	-0.042660822	-0.223583138	-0.036661243	-0.451898877
622901	623957	12 -	ARV1	ORF-T	ORF-T	0.183138584	-0.374039239	0.114525285	-0.260269418	0.298122515
625093	626341	12 -	MAP1	ORF-T	ORF-T	0.366173683	-0.45534667	-0.773824995	-0.241536807	-0.293383733
626341	626933	12 -	CDD1	ORF-T	ORF-T	-0.125509338	0.027300312	0.40325798	0.148219734	0.20694726
628589	633373	12 -	IRC20	ORF-T	ORF-T	0.704558663	-0.283294943	-0.019001559	-0.201648796	0.06620045
633373	634029	12 -	CUT741	CUTs	CUTs	1.78458596	0.318974375	-0.421376998	-0.535313347	-0.903631275
641397	642405	12 -	SUT701	SUTs	SUTs	1.054160549	-0.583698434	-1.321286899	-0.35576918	-0.056543053
644325	645069	12 -	NDL1	ORF-T	ORF-T	0.013619183	-0.588046262	0.154783556	0.106125307	0.362858992
645069	646029	12 -	unknown	no_ovlp	CUTs	1.582899044	-0.187591777	0.01801379	0.277127354	0.136939455
649725	656797	12 -	unknown	no_ovlp	CUTs	1.980416156	0.547238231	-1.156543413	-0.018049241	-0.050682597
657653	658085	12 -	CUT742	CUTs	CUTs	2.343514103	-0.813698049	-0.39858113	-0.266599652	-0.425890332
659941	660605	12 -	SUT702	SUTs	SUTs	0.815989569	-0.438966581	-0.857184389	-0.786619429	-0.690133572
663109	665045	12 -	HSP60	ORF-T	ORF-T	-0.599287285	-0.341474507	0.059077406	-0.076949214	-0.397290323
668013	668949	12 -	VPS63, YPT6	ORF-T	ORF-T	-0.170342293	-1.05180689	-0.418728493	-0.144186308	0.013230593
668949	669165	12 -	unknown	no_ovlp	SUTs	0.365004941	0.497441677	0.371805308	-0.031988539	-0.121806481
669349	669733	12 -	TMA7	ORF-T	ORF-T	-0.734039159	-1.41411487	0.08916035	0.255474034	-0.629121102
672333	672813	12 -	CUT744	CUTs	CUTs	3.462129638	1.031798024	-0.346650931	-0.201677873	0.321685367
674845	675461	12 -	NEJ1	ORF-T	ORF-T	0.63050544	-0.068437905	-0.176963474	-0.47457615	0.113590698
676109	677693	12 -	PDR8	ORF-T	ORF-T	-0.475821638	-0.860768903	0.130912597	0.595673568	0.261617107
678973	679957	12 -	SUT703	SUTs	SUTs	1.711187729	-0.03404028	-0.786120697	-0.791226369	-1.663864411
683677	687253	12 -	YCS4	ORF-T	ORF-T	-0.178017304	-0.189645049	-0.240783196	-0.382209338	0.39838253
688957	691165	12 -	PIG1	ORF-T	ORF-T	-0.570555126	0.002365364	1.145817744	0.631486262	1.583549399
694981	696925	12 -	DBP9	ORF-T	ORF-T	0.145314623	-0.433437281	-0.680771062	0.00538597	0.204371754
697085	699637	12 -	YSH1	ORF-T	ORF-T	-0.157020713	-0.66151619	-0.517376451	-0.204391568	-0.169419029
700005	704085	12 -	YLR278C	ORF-T	ORF-T	0.244017758	-0.47745036	-0.807158325	-0.275301998	-0.691216027
704085	704941	12 -	YLR280C, YLI	ORF-T	ORF-T	-0.00481449	0.887291303	0.722758031	0.247542322	-0.932308179
706117	708269	12 -	ECI1	ORF-T	ORF-T	-1.052112309	-0.319662482	1.332718718	0.575556068	1.558042799
707109	708269	12 -	unknown	no_ovlp2	SUTs	0.560602471	0.335018564	-0.853114067	0.129963425	-0.430242364
708269	710373	12 -	YLR285C-A, (	other	other	-0.128038332	-0.190371273	-0.108304708	-0.046897734	0.364423242
710765	712125	12 -	YLR287C	ORF-T	ORF-T	0.188070714	-0.217447889	-0.26719445	-0.03187654	0.508461514
712725	713221	12 -	RPS30A	ORF-T	ORF-T	0.451431959	-1.239325347	-0.813538348	-0.732465367	0.047507519
713381	714893	12 -	MEC3	ORF-T	ORF-T	0.352059824	-0.256862609	-0.033539286	-0.520635621	0.013596587
717029	717973	12 -	YLR290C	ORF-T	ORF-T	-0.267966279	-0.625837224	0.459185659	-0.282217441	0.587788784
718189	719501	12 -	GCD7	ORF-T	ORF-T	0.092973085	-0.220888045	-0.250903677	-0.007017031	-0.048192712
719677	720389	12 -	SEC72	ORF-T	ORF-T	0.480641654	-0.275005281	-0.670231694	-0.643573415	-0.454505118
720653	721509	12 -	GSP1	ORF-T	ORF-T	-0.099837092	-0.48184538	-0.617549888	-0.169802526	-0.187423477
721717	722445	12 -	YLR294C, ATI	ORF-T	ORF-T	0.181669688	-0.248419693	0.506776998	-0.413808324	0.227438685
724781	725429	12 -	YHC1	ORF-T	ORF-T	0.374505539	-0.079284843	0.16669126	-0.385337931	-0.07792959
727685	728309	12 -	unknown	no_ovlp	CUTs	1.746441275	0.097361708	-0.022511837	0.043996864	0.664462809
734997	737557	12 -	ACO1	ORF-T	ORF-T	-0.527144624	-0.081443088	0.197528476	0.097389898	0.169106018
738069	743901	12 -	STT4	ORF-T	ORF-T	-0.272899096	-0.283074489	-0.17593726	0.071809038	0.09203943
748893	751789	12 -	IMH1	ORF-T	ORF-T	-0.220276029	-0.749390464	-0.130597565	-0.194762829	-0.349026213
751989	757149	12 -	CDC25	ORF-T	ORF-T	-0.020629561	-0.572116513	-0.17337628	0.208157397	-0.156925265
757149	757565	12 -	YLR311C	other	other	-0.324266977	0.642469012	0.679363192	0.125838286	0.324360998
757565	758957	12 -	QNQ1	ORF-T	ORF-T	-0.263449204	-0.480664742	1.641676852	1.21494093	-0.069175832
760285	762507	12 -	SPH1	ORF-T	ORF-T	0.084649297	0.240483679	0.044419344	-0.503323249	0.660478774
762507	764195	12 -	CDC3	ORF-T	ORF-T	-0.359687149	-0.369001615	0.091720105	0.065541913	0.177911077
765187	766387	12 -	TAD3	ORF-T	ORF-T	0.234155567	-0.390084701	-0.52601407	-0.236434307	-0.012783972
769147	771763	12 -	BUD6	ORF-T	ORF-T	-0.241467101	-0.463965864	-0.525409487	0.028299212	-0.097798687
776267	777891	12 -	SFH1	ORF-T	ORF-T	-0.08927145	-0.61568259	-0.675212604	-0.177618996	0.015134177
777963	778971	12 -	CWC24	ORF-T	ORF-T	-0.157480299	-0.180869635	0.323732147	-0.546446877	0.909392973
780907	781435	12 -	RPL38	ORF-T	ORF-T	-0.351339425	-0.196407743	-0.135483953	-0.066224109	0.022217814
781435	781867	12 -	SUT704	SUTs	SUTs	1.700293105	0.481504928	0.277344139	-0.030327022	-0.131858067
782979	783459	12 -	TMA10	ORF-T	ORF-T	0.162690341	-0.289540967	1.623426087	1.476026444	0.124150035
786475	787363	12 -	CUT746	CUTs	CUTs	1.818863047	-0.258635323	-0.735761113	-0.306837016	0.065569886
789315	789875	12 -	CUT747	CUTs	CUTs	2.063513919	0.050969992	-0.224831845	0.117927623	1.078818034
794179	794451	12 -	unknown	no_ovlp	CUTs	3.852287446	-0.619712014	-0.603973896	-0.397700755	-0.414194604
794795	795211	12 -	SNR55, SNR5	other	other	1.421613199	-0.356636484	-0.392810873	-0.893534765	-0.933562879
795907	796395	12 -	RPS25B	ORF-T	ORF-T	0.474028781	-1.104532313	-0.653975548	-0.824368734	0.395155856
799651	802443	12 -	SGD1	ORF-T	ORF-T	0.297510065	-0.150939039	-0.623096008	0.006168326	0.661202691
802539	805163	12 -	VRP1	ORF-T	ORF-T	-0.431672225	-0.046669705	-0.284684147	-0.274930257	0.109935075
807691	808699	12 -	SUT707	SUTs	SUTs	2.042996339	-0.880019782	-0.940534203	-0.75837355	-1.691483334
826323	826579	12 -	KAP95	ORF-T	ORF-T	0.304408481	-0.804295769	-0.523804657	-0.527699539	-0.32071298
826899	828003	12 -	DIC1	ORF-T	ORF-T	0.929338073	-0.005854315	-0.062483659	0.042480809	-0.304117309
828003	828283	12 -	SUT708	SUTs	SUTs	0.345859951	-0.51000164	-0.511330422	-0.179575816	0.152396672
829363	830379	12 -	NIT3	ORF-T	ORF-T	-0.317818117	-0.11549434	0.093874419	-0.002220982	0.03145399
832723	833667	12 -	CUT751	CUTs	CUTs	1.981689679	-0.233250738	-0.477366623	0.001492855	0.248779036
836203	837427	12 -	TAL1	ORF-T	ORF-T	-0.458586228	-0.350818341	-0.361551014	-0.008726686	-0.163325624
837859	839331	12 -	ILV5	ORF-T	ORF-T	-0.258099748	0.110187147	-0.298800755	-0.247970108	-0.092961339
845347	845899	12 -	unknown	no_ovlp	CUTs	1.807449865	0.35373649	-0.57123564	-0.245449768	0.502658429
847075	849699	12 -	DCR2	ORF-T	ORF-T	-0.10092696	-0.322576412	-0.135588123	0.008513506	-0.293548181
849155	849699	12 -	unknown	no_ovlp2	SUTs	-0.065639288	-0.16064105	0.610649079	-0.004908907	0.579853819
852051	853195	12 -	NMD4	ORF-T	ORF-T	0.367443415	-0.957335621	-0.332242204	-0.146971264	-0.078726343

855267	855587	12	-	YLR364C-A	other	other	2.741996637	0.445897509	-0.199445686	-0.566631774	-0.263883622
858251	859259	12	-	unknown	no_ovlp	SUTs	0.460598166	0.243468621	1.00852922	0.812469997	1.229451586
861555	862291	12	-	ARC18	ORF-T	ORF-T	0.046491427	-0.060189111	0.176740775	0.050976482	0.099629241
866411	866803	12	-	CUT752	CUTs	CUTs	3.190752206	0.297558698	-2.159995602	-1.01036939	0.358495556
868659	871379	12	-	VID22	ORF-T	ORF-T	0.074786862	-0.083017256	-0.082658093	-0.094093148	0.684067542
872731	873587	12	-	PSY3	ORF-T	ORF-T	0.003089774	-0.545665944	0.295669006	-0.499902674	0.111874626
873587	875539	12	-	FBP1	ORF-T	ORF-T	-0.324709886	0.342431602	1.679979887	0.73644088	1.297807813
874827	875539	12	-	unknown	no_ovlp2	SUTs	-0.111425118	0.344594402	0.687044392	0.9748567	0.739037237
875539	877379	12	-	SEC61	ORF-T	ORF-T	-0.544718591	-0.313905193	-0.526068134	-0.243136882	-0.221112195
877379	877995	12	-	unknown	no_ovlp	CUTs	1.722832371	0.171894824	-0.641870143	-0.625463547	-0.117341077
877995	880275	12	-	unknown	no_ovlp	SUTs	0.082073052	0.257987791	0.272985259	-0.032086491	0.483135352
881915	884883	12	-	NAM2	ORF-T	ORF-T	-0.451197037	-0.746580688	-0.286906305	-0.383904049	-0.222841323
888795	892851	12	-	IKI3	ORF-T	ORF-T	0.06205756	-0.54362373	-0.896915241	0.011020943	0.077796968
892851	893427	12	-	IKI3, SWC7	ORF-T	ORF-T	0.318619293	0.285014077	-0.120354405	-0.368460791	0.63182659
896283	897747	12	-	REH1	ORF-T	ORF-T	0.081128097	-0.480725967	0.0993642	-0.188118581	0.106130072
899475	902723	12	-	STE23	ORF-T	ORF-T	-0.113347592	-0.537139441	-0.077293743	-0.054195432	0.17796388
902723	903395	12	-	unknown	no_ovlp	CUTs	2.274062176	-0.266389286	-0.512081039	-0.55937544	-0.079408724
904707	906891	12	-	YLR392C	ORF-T	ORF-T	-0.130205866	-0.704803051	0.301483217	0.448326202	-0.163655662
906891	909387	12	-	unknown	no_ovlp	SUTs	-0.104688379	0.413685501	0.113211535	0.266785992	1.209102407
909451	910027	12	-	COX8	ORF-T	ORF-T	-0.527666591	-0.535907201	0.532203792	-0.086712934	0.261837118
910211	912443	12	-	VPS33	ORF-T	ORF-T	-0.255644966	-0.235931284	-0.207979029	-0.162491864	0.060711281
912443	914915	12	-	AFG2	ORF-T	ORF-T	-0.07981759	-0.340268281	-0.904613063	-0.142059414	0.007633216
915051	919051	12	-	SKI2	ORF-T	ORF-T	-0.018123123	-0.021134764	-0.21668422	0.167048945	-0.162587528
919259	921667	12	-	BDF1	ORF-T	ORF-T	0.066203246	-0.548299213	-0.406671334	-0.198209375	-0.488260556
921667	922387	12	-	unknown	no_ovlp2	CUTs	1.876729608	0.320975007	-0.461335942	-0.267402003	0.268472644
922387	924476	12	-	DUS3	ORF-T	ORF-T	0.013081422	-0.222289962	-0.418109176	0.030154124	0.25196792
928236	929604	12	-	CUT753	CUTs	CUTs	1.833725767	0.156676458	-1.316868484	-0.808926592	0.176647447
931348	931748	12	-	RPL31B	ORF-T	ORF-T	0.238094792	-1.108336916	-1.317043708	-0.979001672	0.388486989
933860	934228	12	-	YLR408C	ORF-T	ORF-T	-0.399054504	-0.465416527	0.84168637	-0.430402154	0.857662046
934372	937292	12	-	UTP21	ORF-T	ORF-T	0.06053152	-0.371410553	-0.664727261	0.135918683	0.340976988
947188	948036	12	-	unknown	no_ovlp	SUTs	0.98874404	-0.506606965	0.141463287	-0.260067968	-1.070307213
949164	950020	12	-	unknown	no_ovlp	CUTs	1.707487573	0.203107653	-0.749666653	-0.559766284	-1.109845281
953260	954244	12	-	YLR414C, YLI	ORF-T	ORF-T	0.17475389	-0.135704866	-0.074863381	0.09630542	0.179092354
954292	955396	12	-	unknown	no_ovlp	SUTs	0.654962696	0.600418259	0.072111511	-0.210997614	0.239086429
956628	958156	12	-	CDC73	ORF-T	ORF-T	-0.438169432	-0.791252953	-0.291928063	0.193265379	-0.063281487
963124	963548	12	-	CUT754	CUTs	CUTs	2.463012698	0.064995688	-1.18503621	-0.977225657	-0.675971962
964860	965604	12	-	RPN13	ORF-T	ORF-T	0.131160617	-1.017604124	-0.390070315	-0.349619064	-0.108275784
971612	973188	12	-	ATG17	ORF-T	ORF-T	-1.050815286	-0.235165475	0.827123476	0.033575215	0.83009439
981700	982532	12	-	unknown	no_ovlp	SUTs	-0.08768651	-0.196050644	0.376355631	0.192764323	-0.558878974
982532	983196	12	-	unknown	no_ovlp	SUTs	-0.525126795	0.268254686	0.431172557	0.314977918	0.984493871
990164	990572	12	-	unknown	no_ovlp	CUTs	1.46064862	-0.831192614	-0.528576352	-0.798217541	0.579497249
1000196	1002084	12	-	ATG23	ORF-T	ORF-T	0.271206233	0.131191748	0.275928001	-0.15257668	0.344117146
1004132	1006108	12	-	CNA1, YLR43	other	other	0.635874972	-0.321395214	-0.348046939	-0.141513788	0.01660806
1007340	1011292	12	-	ECM30	ORF-T	ORF-T	-0.298002565	-0.48505676	0.128994025	0.270371061	0.096963291
1011468	1012076	12	-	YLR437C	ORF-T	ORF-T	0.135497526	-0.267209856	-0.281756704	-0.632320929	0.381186048
1013884	1014236	12	-	LSM3	ORF-T	ORF-T	-0.204122495	-0.401821954	-0.061802723	0.210072618	0.179426143
1015500	1017756	12	-	SEC39	ORF-T	ORF-T	-0.301303925	-0.58755314	-0.331797035	-0.175454711	-0.045909188
1017932	1018940	12	-	RPS1A	ORF-T	ORF-T	-0.148559158	0.136445706	-0.224088746	-0.237838837	-0.028351953
1019220	1022276	12	-	SIR3	ORF-T	ORF-T	0.019692948	-0.254491053	-0.06787399	-0.201360414	0.337539399
1025452	1026708	12	-	SUT711	SUTs	SUTs	2.776945011	-0.059663749	-2.895844926	-0.712029209	-0.941308149
1026884	1027940	12	-	VMA6	ORF-T	ORF-T	-0.083142332	-0.39729849	-0.187660561	-0.093917215	0.191489242
1029964	1030580	12	-	SUT712	SUTs	SUTs	1.975481393	0.540614075	-0.304697818	-0.732777627	0.604860557
1034612	1035780	12	-	SUT713	SUTs	SUTs	1.809320105	-0.539812139	-1.296182526	-0.314493538	-0.545980317
1039124	1041452	12	-	SST2	ORF-T	ORF-T	0.664282646	-0.795992482	-1.576178906	-0.745980673	-1.135475127
1041452	1043572	12	-	RIF2	ORF-T	ORF-T	-0.08629681	-0.836109867	0.337361263	0.281450544	0.446700261
1043020	1043572	12	-	unknown	no_ovlp2	CUTs	2.207500535	0.144825162	0.325868973	-0.253805654	0.128066836
1052332	1053164	12	-	YLRCdelta27	other	other	0.57196034	-0.946796183	-0.368415802	-0.046180821	-0.513957147
1055596	1056780	12	-	NBP1	ORF-T	ORF-T	0.306246093	0.072654474	-0.060242119	-0.30056086	-0.176300108
17953	19833	12	+	MMP1	ORF-T	ORF-T	0.612127559	-0.123856938	0.137296186	0.785961563	-0.668396447
22169	23489	12	+	SUT244	SUTs	SUTs	0.818284302	0.454291739	-0.103747663	0.098900067	-0.538202268
23489	25385	12	+	YLL058W	ORF-T	ORF-T	-0.528530674	0.501385689	0.967843745	0.644100393	0.957818973
26217	27273	12	+	SUT245	SUTs	SUTs	1.198523248	-0.188354787	-0.344803993	-0.150064213	-1.640448574
29585	31777	12	+	YCT1	ORF-T	ORF-T	0.173567723	-0.77336443	-0.126879834	1.284485018	-0.034312035
34953	40665	12	+	unknown	no_ovlp	SUTs	-0.163095382	0.437294105	0.070164462	0.022358282	-0.096412271
40665	41289	12	+	LDB18	ORF-T	ORF-T	-0.621784309	-0.614562391	0.248328365	0.137840174	0.166263602
46657	47337	12	+	unknown	no_ovlp	CUTs	2.175576488	0.320793342	0.599081819	0.263198415	1.115027231
49857	52105	12	+	FPS1	ORF-T	ORF-T	-0.231285243	-0.536648243	-0.411262362	-0.031908025	-0.086525169
65817	67761	12	+	unknown	no_ovlp	CUTs	2.69910748	0.258873323	-0.890082347	-0.153644157	0.482173233
68529	70537	12	+	GRC3	ORF-T	ORF-T	0.100572716	-0.012229379	-0.553235023	-0.171561782	0.893735447
73417	74681	12	+	IRC19	ORF-T	ORF-T	0.11151032	-0.131737189	0.084641588	0.052844773	-0.132410603
81337	83809	12	+	YLL029W	ORF-T	ORF-T	-0.379669248	-0.521492796	0.314918004	0.423502147	-0.082604095
84681	86969	12	+	TPO1	ORF-T	ORF-T	0.151050836	-0.716161011	-0.664092525	-0.192351047	-0.400910379
87209	88281	12	+	ISA1	ORF-T	ORF-T	0.434231949	-0.655555105	0.009532321	-0.049492703	-0.446380467
88585	91433	12	+	HSP104	ORF-T	ORF-T	0.092986175	-0.451260594	0.383817471	0.298329278	-0.094785551
93369	94793	12	+	PAU17	ORF-T	ORF-T	-0.033434061	-0.502409802	0.12696023	-0.026793747	-1.670692554
97825	99345	12	+	CUT246	CUTs	CUTs	2.410306633	0.150432773	-1.090097776	-0.529105433	-0.832182417
100809	105393	12	+	SPA2	ORF-T	ORF-T	-0.160096954	0.139993784	-0.711811055	0.046960954	-0.074613705
109241	109785	12	+	CUT248	CUTs	CUTs	1.624932704	0.498194126	0.635390658	0.386864903	0.509833628
111889	114513	12	+	YLL017W, SD	other	other	-1.226960305	-0.691715737	0.940876628	0.620321847	0.400616783
116321	121169	12	+	BPT1	ORF-T	ORF-T	-0.255448318	-0.397802786	-0.00784965	0.178785029	0.133673535
121313	121793	12	+	YLL014W	ORF-T	ORF-T	-0.391673701	-0.583744582	0.119482725	-0.202278208	-0.218087555
125513	127313	12	+	YEH1	ORF-T	ORF-T	0.906736505	0.708658656	-0.20425818	0.491725197	-0.944461525

127465	129073	12	+	SOF1	ORF-T	ORF-T	0.332697874	-0.029625997	-0.699484721	0.126813884	0.198354403
131705	134097	12	+	DRS1	ORF-T	ORF-T	0.259243587	-0.313205341	-0.688978982	0.063470949	0.152687895
136561	138913	12	+	MMM1	ORF-T	ORF-T	0.027451321	-0.139153326	0.203404154	0.087284637	-0.286729456
141081	142993	12	+	ORC3	ORF-T	ORF-T	-0.125937141	-0.555126994	-0.405996531	-0.632796391	-0.25096378
143185	146113	12	+	SFI1	ORF-T	ORF-T	0.468406084	-0.170978945	-0.206329147	-0.272089855	-0.028716429
146281	147657	12	+	RTT109	ORF-T	ORF-T	-0.622064479	-0.515395345	0.062956308	-0.331483248	0.25613374
147849	150313	12	+	DNM1	ORF-T	ORF-T	-0.343107691	-0.576076853	-0.076008967	0.028041445	0.078512844
151113	151737	12	+	SUT246	SUTs	SUTs	1.42912395	-0.491506399	-0.287227542	0.411852709	-1.597685481
158001	158281	12	+	unknown	no_ovlp	CUTs	1.720537849	0.222773893	0.131024946	0.154070607	-0.011883028
160009	161625	12	+	SSL1	ORF-T	ORF-T	-0.263214565	-0.602231949	-0.020806947	-0.004137829	-0.097048302
163601	164097	12	+	unknown	no_ovlp	CUTs	2.515264522	0.309784013	-0.753215068	-0.429987899	-0.115893347
164377	165465	12	+	NSE1	ORF-T	ORF-T	0.114735515	-0.380072563	0.038256338	-0.318212386	0.560082625
166377	167265	12	+	RLP24	ORF-T	ORF-T	0.515095455	-0.239105186	-0.607845674	-0.155489702	0.159339735
169049	169977	12	+	LOT6	ORF-T	ORF-T	-0.04849565	-0.339860774	0.68528472	0.035902203	0.098120689
175177	176769	12	+	BRE2	ORF-T	ORF-T	0.047667167	0.417552383	0.297054929	0.007371	1.023175879
177625	178705	12	+	MEU1	ORF-T	ORF-T	-0.221035755	-0.351749248	-0.149167705	-0.11421301	0.423732305
180017	181721	12	+	PSR2	ORF-T	ORF-T	-0.378183683	-0.312943779	-0.090136095	0.039277715	0.120170466
183593	184233	12	+	IRC25	ORF-T	ORF-T	0.235321675	-0.515933288	-0.017606553	-0.390347906	0.181180169
185057	185409	12	+	CUT249	CUTs	CUTs	3.090802822	-0.120779425	-0.517844846	-0.404917846	-0.385612536
187665	188113	12	+	CUT250	CUTs	CUTs	1.135149235	0.26270063	-0.643114816	-0.433343554	-1.157319131
188113	188865	12	+	CUT250	CUTs	CUTs	2.835629763	-0.829409529	-0.018102834	0.510181806	-0.939962318
193473	194393	12	+	CUT251	CUTs	CUTs	0.744415026	0.167219203	0.428223625	0.239943677	1.417614777
194393	195321	12	+	SNF7	ORF-T	ORF-T	0.009056484	-0.374171423	0.093656971	0.175587334	0.194620283
196833	197369	12	+	CUT252	CUTs	CUTs	2.090958481	-0.172249392	-1.964104682	-0.007044501	0.515225231
198761	199553	12	+	SNR30	other	other	0.267105296	0.061828515	-0.05810827	-0.112474164	-0.024293953
201681	202641	12	+	unknown	no_ovlp	CUTs	1.076491077	-0.092761897	0.344477241	0.731554972	-0.221042849
204993	208553	12	+	RAD5	ORF-T	ORF-T	-0.09342589	0.080893037	0.045617133	-0.081254493	0.716484156
208761	210457	12	+	RSC58	ORF-T	ORF-T	-0.232306605	-0.823661528	-0.72232987	-0.093093016	-0.341108513
221009	221737	12	+	unknown	no_ovlp	CUTs	1.662802221	-0.91247857	-1.895032073	0.271053497	-1.223205918
224001	226009	12	+	SUT250	SUTs	SUTs	1.125110916	0.505996539	0.535992201	0.046180618	-0.49165899
228729	230809	12	+	YLR041W	other	other	-0.239231613	-0.326973771	0.264305263	-0.535579209	-1.099797182
230513	230809	12	+	unknown	no_ovlp2	SUTs	-0.36054597	0.125282114	0.495954718	-0.041770465	-0.326793019
230809	231705	12	+	SUT251	SUTs	SUTs	1.702892016	-0.087964445	0.220728937	-0.241961144	-1.94324698
234881	235745	12	+	SUT252	SUTs	SUTs	2.209787349	0.211785098	-0.716853924	-0.116680001	-0.882221927
242321	242689	12	+	RPS0B	ORF-T	ORF-T	0.122761482	-1.266375624	-0.432814702	-0.921595119	0.37031584
245577	246297	12	+	SUT253	SUTs	SUTs	1.414362811	-0.556318758	-1.140595016	-0.509296402	-0.055734815
247185	248041	12	+	IES3	ORF-T	ORF-T	0.181322	-0.457264301	-0.254518245	-0.462573815	-0.316174609
252545	253785	12	+	unknown	no_ovlp	SUTs	0.445651327	0.43841224	0.334668525	0.757135967	1.377117965
253785	255137	12	+	ERG3	ORF-T	ORF-T	-0.36529189	-0.278324282	-0.666065533	0.116430361	-0.28077997
255289	257929	12	+	YLR057W	ORF-T	ORF-T	-0.005680362	-0.099684927	0.101534254	-0.023098107	0.510672761
259913	260953	12	+	SUT255	SUTs	SUTs	2.054464247	-0.251668751	-0.522529529	-0.716024451	-0.380404043
260953	262889	12	+	FRS1	ORF-T	ORF-T	-0.000319566	-0.099262093	-0.110774619	0.088871679	-0.030588297
263153	263608	12	+	RPL22A	ORF-T	ORF-T	1.617493305	-0.60670495	0.075851566	-0.520651569	-0.311551825
264633	265417	12	+	YLR063W	ORF-T	ORF-T	0.193329911	0.447350234	-0.119808196	-0.180568469	1.336963358
265417	266393	12	+	YLR064W	ORF-T	ORF-T	1.58668526	0.434901792	-0.325792586	-0.192664356	-0.266808103
267129	267825	12	+	SPC3	ORF-T	ORF-T	0.525599815	-0.716372221	-0.316571675	-0.330045639	-0.139478072
270985	271601	12	+	FYV7	ORF-T	ORF-T	0.406303363	-0.624324206	-0.559604517	-0.109899053	0.630836892
274113	275313	12	+	SUT256	SUTs	SUTs	0.989627636	-0.810941611	0.23369813	-0.082369252	-0.097219274
278857	281025	12	+	YLR072W	ORF-T	ORF-T	-0.1341734	-0.438194249	-0.251468167	-0.097185131	-0.011117816
281985	282913	12	+	SUT258	SUTs	SUTs	2.213382992	-0.326526955	-0.524895696	-0.151054125	0.194692215
282913	283745	12	+	RPL10	ORF-T	ORF-T	-0.44097686	-0.154443071	-0.057509683	-0.023058745	0.068891605
283913	285777	12	+	FMP25	ORF-T	ORF-T	-0.366632082	-0.761096998	-0.017328481	-0.110311119	0.306150207
286793	287841	12	+	SIC1	ORF-T	ORF-T	0.120898877	-0.110646867	-0.168767264	-0.123022224	0.184227411
288017	289321	12	+	EMP46	ORF-T	ORF-T	-0.849715062	-0.149470792	1.320725755	0.180410705	-0.60076039
290633	291745	12	+	GAL2	ORF-T	ORF-T	-0.446694274	-0.140215807	0.077815215	0.02736044	-0.063383922
300617	302201	12	+	unknown	no_ovlp	CUTs	1.807648409	0.341050514	-1.06209427	-0.579483784	0.163263188
302201	306697	12	+	SMC4	ORF-T	ORF-T	0.091089596	-0.167914336	-0.158242008	-0.253401169	0.24730987
316081	318137	12	+	GAA1	ORF-T	ORF-T	-0.107437099	-0.322799632	-0.556007391	-0.294481975	-0.111069681
320433	320697	12	+	XDJ1	ORF-T	ORF-T	0.476456235	-0.735488391	-0.25300838	-0.265646366	-0.296210295
322217	323521	12	+	unknown	no_ovlp2	SUTs	-0.161077087	0.102248698	-0.011989354	0.191018085	0.897614167
323521	326385	12	+	SUL2	ORF-T	ORF-T	0.046203274	-0.189329017	-0.34062409	-0.237658982	0.263853484
327609	328657	12	+	unknown	no_ovlp	CUTs	1.28067796	0.207674146	-0.049194414	-0.041109222	0.420022252
329713	329969	12	+	SUT260	SUTs	SUTs	-0.712166221	-0.603777869	0.847818654	1.28857409	1.714810196
332401	336145	12	+	KIN2	ORF-T	ORF-T	0.198476849	-0.038011062	-0.166264287	0.098632564	0.365096054
339409	341281	12	+	SUT261	SUTs	SUTs	0.648122626	-0.131276874	-0.454011138	-0.223101751	-0.496566422
341281	341801	12	+	YLR099W-A	ORF-T	ORF-T	-0.205794211	-0.271784676	-0.073016588	-0.543982129	-0.565425222
341801	342945	12	+	ERG27	ORF-T	ORF-T	0.565520856	0.006169377	-0.534040346	-0.099089865	0.125039459
346553	347041	12	+	YLR104W	ORF-T	ORF-T	-0.411513402	-0.55499254	-0.298621775	-0.606302275	0.037203025
364105	365441	12	+	REX3	ORF-T	ORF-T	0.471274478	-0.428000067	0.198358425	-0.096473253	0.292992315
366529	368689	12	+	unknown	no_ovlp	SUTs	-0.510772846	0.090840712	0.285767751	0.138503273	0.20210428
368753	369401	12	+	AHP1	ORF-T	ORF-T	-0.364659939	0.041082891	0.511004536	-0.059471785	0.300558158
370673	371281	12	+	YLR111W, YL	other	other	0.182938455	-0.091986615	-0.457235206	-0.458412525	-0.14812544
371281	373065	12	+	HOG1	ORF-T	ORF-T	0.002036868	-0.649288365	-0.540728497	-0.309507214	-0.398523583
373385	374337	12	+	unknown	no_ovlp	CUTs	1.546796369	-0.820582978	0.323311878	0.021025543	-0.200219033
377969	380945	12	+	CFT2	ORF-T	ORF-T	-0.011812473	-0.155730833	0.05150637	0.00982621	0.412738084
380689	380945	12	+	unknown	no_ovlp2	SUTs	0.493684653	-0.298147797	-0.419379615	-0.590517676	0.502463958
380945	382449	12	+	MSL5	ORF-T	ORF-T	0.210160494	-0.280620803	-0.295996023	0.005122501	0.109941426
385593	386257	12	+	SRN2	ORF-T	ORF-T	0.242958155	-0.538003138	0.347503854	0.117321026	-0.294268121
387673	388577	12	+	unknown	no_ovlp	SUTs	-0.463602126	0.054345134	0.620674578	0.759749276	2.472603759
388577	390417	12	+	YLR120W-A	other	other	0.654164134	-0.630915623	-0.507378076	0.085054659	-0.292860648
391833	393705	12	+	YLR124W, YL	other	other	1.711612367	1.510686884	0.000486791	0.219327061	-1.503635759
395801	396673	12	+	SUT263	SUTs	SUTs	0.382653118	-1.275872879	-1.37421355	-0.479963968	-0.423879713

398617	399649	12	+	DCN1	ORF-T	ORF-T	0.319418353	0.3603923	0.403042063	-0.53371839	-0.306658335
399649	402577	12	+	DIP2	ORF-T	ORF-T	0.479343985	-0.254921453	-0.871899862	-0.038959811	-0.163964526
404521	405121	12	+	CUT259	CUTs	CUTs	3.504194425	0.449403328	-1.571736764	-0.426579229	0.053701561
408393	410281	12	+	unknown	no_ovlp2	SUTs	-0.320374975	-0.453794366	-0.129528067	0.035009568	0.125273336
410281	412537	12	+	PDC5	ORF-T	ORF-T	0.419069248	0.187571603	-0.50243805	0.342511447	-0.045091135
413265	415609	12	+	SLX4	ORF-T	ORF-T	-0.00100919	-0.282652134	0.288212167	-0.180605111	0.248270655
415609	416505	12	+	CUT260	CUTs	CUTs	2.776719623	-0.346948933	-1.294228682	-0.168854461	-0.542620544
417009	418153	12	+	YLR137W	ORF-T	ORF-T	0.966466413	-0.434486946	-0.428986177	-0.161168339	-0.629069662
418297	421449	12	+	NHA1	ORF-T	ORF-T	-0.405204711	-0.393984672	-0.054073857	0.354230994	-0.190129528
423673	425161	12	+	YLR140W, RI	ORF-T	ORF-T	-0.436628679	-0.324848913	-0.100388266	-0.212333942	-0.373537586
425161	426753	12	+	PUT1	ORF-T	ORF-T	-0.203344985	0.200976673	2.091743192	1.709590787	0.908315982
427305	429553	12	+	YLR143W	ORF-T	ORF-T	0.231127732	-0.303227159	-0.709851945	-0.11776393	-0.262462308
432193	432817	12	+	RMP1	ORF-T	ORF-T	0.60090001	-0.190990818	-0.291998534	-0.44487674	0.138021145
433865	434145	12	+	YLR146W-A	ORF-T	ORF-T	0.713466789	-0.022913785	0.297144269	-0.241814658	0.056344378
434281	434665	12	+	unknown	no_ovlp	SUTs	-0.635343994	-0.555700707	0.381127885	-0.004283125	0.308939942
434665	437577	12	+	PEP3	ORF-T	ORF-T	-0.114994597	-0.478999118	-0.098523983	0.009263981	-0.257830768
440393	441489	12	+	STM1	ORF-T	ORF-T	-0.422179736	-0.243969444	-0.186362582	0.010641675	-0.032919256
445001	445273	12	+	unknown	no_ovlp	SUTs	0.606741055	0.418624278	0.521545104	0.104875709	-1.075416708
448041	448441	12	+	unknown	no_ovlp	CUTs	2.407451812	-0.036567174	-0.705846118	0.127939591	-0.48754686
449753	450481	12	+	SUT264	SUTs	SUTs	2.870978573	-0.3528439	-0.659781469	-0.245041426	-1.094539179
459905	460337	12	+	unknown	no_ovlp	SUTs	-3.296729544	-0.365262213	2.552078924	0.044599672	-0.238795485
460337	489473	12	+	unknown	no_ovlp	SUTs	0.685312388	-0.70822594	1.89924543	-0.759154509	-1.732107009
490937	491817	12	+	SUT265	SUTs	SUTs	-0.1585517	-0.697517246	0.284504064	-0.137689372	0.512714984
493553	494209	12	+	YLR164W	ORF-T	ORF-T	-0.603061649	-0.830914352	1.173688682	0.421612625	1.026980987
498153	498865	12	+	SUT267	SUTs	SUTs	1.496915328	0.023571344	0.391996683	0.012172817	-0.13517746
498865	499465	12	+	RPS31	ORF-T	ORF-T	-0.532628994	-0.142795711	0.015593239	-0.097082871	0.042325525
499537	500617	12	+	unknown	no_ovlp	SUTs	0.041318803	0.30843121	-0.151349015	0.200678097	0.304160964
500617	502345	12	+	unknown	no_ovlp2	CUTs	1.01951791	0.185416629	0.297389831	-0.501029362	0.140401852
502345	504609	12	+	YLR173W	ORF-T	ORF-T	0.302651425	-0.132315168	0.383488552	-0.009240027	0.515307645
504609	505929	12	+	IDP2	ORF-T	ORF-T	0.393732841	0.29145965	1.877747602	0.26691016	0.605834295
506097	507721	12	+	CBF5	ORF-T	ORF-T	-0.221324373	-0.908714219	-0.827734977	-0.133956491	-0.398893614
510737	511017	12	+	YLR177W	ORF-T	ORF-T	-0.721251484	-0.57123803	0.192573969	-0.217983683	-0.030929045
515217	516689	12	+	SAM1	ORF-T	ORF-T	0.048841181	-0.171169971	-0.831420617	-0.192796837	-0.057611282
517929	520473	12	+	SWI6	ORF-T	ORF-T	-0.135521475	-0.491424537	-0.080970791	0.016115748	0.205580267
522617	523057	12	+	RPL37A	ORF-T	ORF-T	1.121190838	-0.564941433	-1.640359716	-1.067080594	-0.312120435
523585	524465	12	+	EMG1	ORF-T	ORF-T	0.259354673	-0.078384164	-0.780346979	-0.391637321	0.27950607
524681	526249	12	+	SKG3	ORF-T	ORF-T	0.15409227	-0.414864997	-0.520419441	-0.426222606	-0.340267287
528233	530617	12	+	MDL1	ORF-T	ORF-T	-0.323964205	-0.247869921	-0.281369038	-0.209266845	0.02888146
534849	537041	12	+	MMR1	ORF-T	ORF-T	0.043357986	-0.01234435	-0.16512908	-0.179137987	0.131937164
537257	538537	12	+	PEX13	ORF-T	ORF-T	-0.412758191	-0.205419797	0.064458934	-0.124810189	0.395526661
540793	541769	12	+	SUT270	SUTs	SUTs	1.441389707	-0.096323361	-0.398541455	0.200018429	-0.108876774
542017	542745	12	+	CUT265	CUTs	CUTs	2.378971665	-0.100656327	-1.09204087	-0.202756229	-0.336149473
543913	545833	12	+	PWP1	ORF-T	ORF-T	0.151790792	-0.413839151	-0.774519709	0.081916238	0.256823732
546057	547793	12	+	SIK1	ORF-T	ORF-T	-0.044474795	-0.563386735	-0.617642524	-0.186060163	-0.260870488
548969	549537	12	+	YKE2	ORF-T	ORF-T	0.636609326	-0.124889756	-0.245882135	-0.501851864	-0.153182915
552249	552761	12	+	QRI5	ORF-T	ORF-T	-1.279905675	-0.937689321	0.239614674	-0.041773897	0.31438327
554057	554465	12	+	SUT271	SUTs	SUTs	0.232836439	-0.622256702	0.144371346	-0.175237245	-0.359746307
554465	556561	12	+	ENT2	ORF-T	ORF-T	-0.291843736	-0.443543411	0.301578333	0.12116196	-0.112525483
556753	559353	12	+	HRD3	ORF-T	ORF-T	-0.47030875	-0.509800969	-0.226641633	0.186939143	0.027352196
559441	560721	12	+	SEC13	ORF-T	ORF-T	-0.440865222	-0.633272727	-0.294848203	0.047101555	-0.08621298
561969	563569	12	+	CLB4	ORF-T	ORF-T	-0.29799518	-0.385145048	0.051759237	-0.25997686	0.973422662
566689	568481	12	+	SUT272	SUTs	SUTs	1.183561298	0.239901233	-0.039712048	0.358372974	-0.637130776
568481	570737	12	+	FRE1	ORF-T	ORF-T	-0.803962746	-1.919211267	0.018839569	-0.157380717	-1.207725641
573473	574129	12	+	unknown	no_ovlp	CUTs	1.523747026	0.180870919	1.096446836	0.121570713	0.663370147
574129	576497	12	+	MSC3	ORF-T	ORF-T	-0.503725075	-0.237520959	0.411157014	0.274065454	0.688128364
576697	577961	12	+	CCC1	ORF-T	ORF-T	-0.545663665	-1.343642221	-0.277750072	0.132649612	0.167802016
586153	586401	12	+	YLR224W	ORF-T	ORF-T	-0.214640376	-0.484133817	0.657614808	0.641874996	1.099668901
589321	591121	12	+	BUR2	ORF-T	ORF-T	0.021841641	-0.436763296	-0.312176626	-0.400576556	-0.172237227
591353	591793	12	+	SUT273	SUTs	SUTs	2.269751317	0.407268915	-0.432338948	-0.320697036	0.646548411
603889	604497	12	+	SUT275	SUTs	SUTs	0.102489135	-0.874907742	0.457518101	0.164392459	-1.40974674
610049	612305	12	+	TOP3	ORF-T	ORF-T	-0.371454317	-0.015724909	0.261451057	-0.092424545	0.96016664
612305	614361	12	+	THI7	ORF-T	ORF-T	-0.365723884	-0.525067003	0.071036506	1.273773318	0.843650635
614705	616233	12	+	FAR10	ORF-T	ORF-T	0.075700402	-0.562166435	-0.191141305	0.077742597	-0.551748788
617521	620249	12	+	VPS34	ORF-T	ORF-T	-0.067105261	-0.099391211	0.224055234	0.07166048	-0.035145467
620449	622889	12	+	YLR241W	ORF-T	ORF-T	-0.027640544	0.161288868	-0.029673008	-0.099770336	0.311691429
624145	625161	12	+	YLR243W	ORF-T	ORF-T	0.191975192	-0.307636058	-0.519488804	-0.198351441	0.292773966
626545	627097	12	+	unknown	no_ovlp2	CUTs	2.291025108	-0.052090195	-0.184301798	-0.656141296	0.024582382
627097	628281	12	+	ERF2	ORF-T	ORF-T	0.351783832	0.047602361	-0.129974091	-0.162730933	0.009275319
633697	636217	12	+	RCK2	ORF-T	ORF-T	-0.66125993	-0.379496176	0.079726079	0.417790989	0.029280782
636753	640089	12	+	YEF3	ORF-T	ORF-T	-0.393925542	-0.040417174	-0.248215447	-0.03459557	-0.102512931
640289	641129	12	+	SSP120	ORF-T	ORF-T	-0.330398809	-0.783542996	0.066202338	-0.091305704	-0.232322373
641129	641353	12	+	unknown	no_ovlp2	CUTs	1.119454526	-0.443190506	-0.045687777	-0.956502609	-1.641832018
641353	642321	12	+	SYM1, YLR25	ORF-T	ORF-T	0.135184816	-0.172257864	1.079668679	1.108267764	0.340160575
642569	644409	12	+	YLR253W	ORF-T	ORF-T	-0.467396208	-0.590547903	-0.349125994	-0.202973503	-0.365235383
645329	645697	12	+	unknown	no_ovlp	CUTs	2.605063571	-0.035307921	-0.500793854	-0.164028412	0.264088398
646129	648585	12	+	unknown	no_ovlp	SUTs	0.144350858	-0.317597299	-0.332086075	0.010078488	-0.524062057
657209	657833	12	+	unknown	no_ovlp	CUTs	1.885879346	-0.088647921	-0.602944399	-0.490116571	-1.342295076
658633	659905	12	+	YLR257W	ORF-T	ORF-T	-0.127634594	-0.665382135	0.24415908	-0.011240372	-0.07824639
660281	660665	12	+	CUT266	CUTs	CUTs	-0.279264661	-0.72693455	0.436704111	-0.055148	-0.013415505
660665	663201	12	+	GSY2	ORF-T	ORF-T	-0.449005106	-0.416545594	0.989479662	0.460706187	-0.158949988
665793	668065	12	+	LCB5	ORF-T	ORF-T	0.782368546	0.821890751	0.263431409	-0.111029269	0.759461308
669209	669417	12	+	SUT279	SUTs	SUTs	3.682316868	-0.139160205	0.121866713	0.098634064	0.21084777

671449	672801	12	+	RED1	ORF-T	ORF-T	-0.104762013	-0.439061604	-0.235269076	-0.393846178	0.881554179
673121	673985	12	+	RPS28B	ORF-T	ORF-T	-0.529730321	-0.088741092	-0.479007301	-0.561387564	0.428839946
674305	674945	12	+	unknown	no_ovlp	CUTs	2.122012623	-0.046341323	-0.538906547	-0.51468082	1.892117433
675681	676441	12	+	CUT268	CUTs	CUTs	2.733325194	0.771582567	-1.155966938	-0.287180403	-0.456624763
676441	677913	12	+	unknown	no_ovlp2	SUTs	0.366851067	0.40295053	-0.312811582	-0.263080029	-0.064788869
677913	680169	12	+	BOP2	ORF-T	ORF-T	-2.14765171	-0.550155397	3.235542235	2.945739099	2.1563141
679961	680169	12	+	unknown	no_ovlp2	SUTs	-0.55225211	0.119798124	0.694131163	1.023215504	0.415901402
680169	680953	12	+	SEC22	ORF-T	ORF-T	0.141199324	0.322394841	-0.050199621	0.121775651	-0.158259926
681097	682361	12	+	DCS1	ORF-T	ORF-T	-0.533765991	-0.453757331	0.85018936	0.372629218	0.425168948
682361	682593	12	+	unknown	no_ovlp2	SUTs	0.811998689	-0.387758536	-0.769332659	0.277738375	-0.63811267
682593	683593	12	+	YLR271W	ORF-T	ORF-T	0.23458723	-0.277456217	0.307493126	0.366451154	-0.869994055
691537	694025	12	+	CDC46	ORF-T	ORF-T	-0.039281571	-0.399548362	-0.165782229	-0.050459804	0.175031428
694489	695057	12	+	SMD2	ORF-T	ORF-T	0.393005286	-1.076977374	-0.513584416	-0.610776405	-0.358224846
699921	701305	12	+	SUT280	SUTs	SUTs	-0.424847215	0.911217227	1.381674941	1.41089413	2.281268588
704265	705193	12	+	unknown	no_ovlp	SUTs	-0.0941907	-0.543298261	-0.139771583	0.224809562	0.535919657
705193	706185	12	+	YLR279W, YL	other	other	0.197023317	-0.312207713	0.066633233	0.219779735	0.188828915
707345	708281	12	+	NNT1	ORF-T	ORF-T	-0.350769853	-0.742346302	-0.601914609	-0.423525773	-0.054697225
715113	717161	12	+	GUF1	ORF-T	ORF-T	-0.310851862	-0.299181385	0.061938899	-0.285213014	0.113674263
719681	719977	12	+	CUT269	CUTs	CUTs	2.840145213	0.429538519	-0.971233154	-0.416120635	-0.348321174
721737	722441	12	+	CUT270	CUTs	CUTs	2.154504221	0.192477774	-1.082210035	-0.649574004	0.052138647
722441	722705	12	+	unknown	no_ovlp2	SUTs	0.529154334	-0.153373402	-0.990621946	-0.600412403	-0.54455803
722705	723801	12	+	YLR296W	other	other	-0.194064449	0.215779481	0.164630458	0.122610184	0.242221609
723921	724729	12	+	YLR297W	ORF-T	ORF-T	0.413746857	-0.728974773	-0.090000706	-0.6087117	0.321073227
725889	728185	12	+	ECM38	ORF-T	ORF-T	-0.229765929	-0.328518928	-0.152733919	-0.064584928	-0.034956386
728857	730529	12	+	EXG1	ORF-T	ORF-T	-0.14302124	-0.021872391	-0.722198997	-0.45946293	0.081909756
730801	732009	12	+	unknown	no_ovlp	SUTs	0.159720411	-0.485301631	-0.592399415	-0.487276724	-0.369616832
732497	733961	12	+	MET17	ORF-T	ORF-T	-0.39594307	0.02745702	-0.293546718	-0.084274927	-0.327137401
738153	738545	12	+	CUT271	CUTs	CUTs	2.393018731	0.323562954	0.092319174	-0.483970304	-0.145023116
744297	745009	12	+	UBC12	ORF-T	ORF-T	0.431400215	-0.559280166	-0.14926034	0.07950022	-0.560700035
751089	752137	12	+	unknown	no_ovlp	SUTs	0.620516767	0.497620396	0.796735259	0.112046794	1.443213612
756833	759481	12	+	unknown	no_ovlp	SUTs	0.188823599	0.405048942	-0.18751911	0.147069979	-0.275306418
759481	760353	12	+	MRPL15	ORF-T	ORF-T	-0.820316604	-0.950772446	-0.130278853	0.072838553	-0.012677714
764767	766111	12	+	NKP2, YLR31	other	other	-0.646574492	-0.823030893	0.138362767	0.081186397	0.510368587
766111	766399	12	+	unknown	no_ovlp2	SUTs	-1.099768342	-0.634863694	0.84243843	0.402516043	1.209006082
766399	769207	12	+	EST2	ORF-T	ORF-T	-0.2039813	-0.577500305	-0.03439466	-0.758528589	0.203008038
771943	776311	12	+	MMS22	ORF-T	ORF-T	0.061981854	-0.479587228	-0.366545782	-0.276994819	-0.082161822
778063	779175	12	+	unknown	no_ovlp	SUTs	0.967338805	0.333319885	-0.742860905	-0.238941888	0.705464079
779175	780879	12	+	PEX30	ORF-T	ORF-T	-0.555510233	0.071997501	0.289914721	-0.007984839	0.909182542
782071	783031	12	+	YLR326W	ORF-T	ORF-T	0.602533529	0.170301354	0.672590442	-0.228105509	0.578484921
784711	786287	12	+	NMA1	ORF-T	ORF-T	-0.112434611	-0.810243865	-0.433460813	-0.191178444	-0.333370558
787623	789831	12	+	CHS5	ORF-T	ORF-T	-0.131447216	-0.405409521	-0.090867803	-0.098258856	-0.224909941
790279	792023	12	+	MID2	ORF-T	ORF-T	0.034977218	-0.354136849	-0.174621523	-0.167057296	-0.201135608
796791	797111	12	+	unknown	no_ovlp	CUTs	2.496404218	-0.607101627	-1.321710434	-1.042174748	-3.062352731
797423	799663	12	+	NUP2	ORF-T	ORF-T	-0.073537767	-0.542307438	-0.414648058	-0.072889589	0.027364108
803007	805831	12	+	unknown	no_ovlp	SUTs	-0.200399181	0.340191655	0.067486308	0.070660288	0.577413937
805831	806919	12	+	RPP0	ORF-T	ORF-T	-0.622921191	-0.192254668	-0.018681944	-0.065738243	0.154206456
808631	809751	12	+	unknown	no_ovlp	SUTs	0.455395073	0.248183293	-0.295159187	-0.30297529	-0.871944498
809751	815855	12	+	FKS1, YLR342	other	other	-0.285919845	-0.183181323	-0.256368515	-0.047883695	-0.210259149
815855	816111	12	+	YLR342W-A,	other	other	0.772722064	-0.596105216	-0.029679385	-0.19945327	0.577216254
818687	819271	12	+	unknown	no_ovlp	SUTs	0.894581099	0.348773435	-0.17524311	-0.505607474	-0.799469634
819327	819783	12	+	RPL26A	ORF-T	ORF-T	0.522564747	-0.741326267	-0.879612387	-0.995446567	-0.33172747
820439	822127	12	+	YLR345W	ORF-T	ORF-T	-0.357130011	-0.797375856	0.632705676	0.46106102	0.161263811
826879	827415	12	+	YLR349W	other	other	2.36471028	-0.195121031	-1.038476724	-0.473061644	-0.628680029
828407	828655	12	+	unknown	no_ovlp	SUTs	0.583540936	-0.633674983	0.447565616	0.140411162	-0.08561101
828655	829511	12	+	ORM2	ORF-T	ORF-T	0.116848195	-0.637260874	-0.152121118	-0.04743915	0.017923361
830983	833527	12	+	YLR352W	ORF-T	ORF-T	0.218265617	0.03358938	0.330276893	0.018133133	0.517481854
833911	836295	12	+	BUD8	ORF-T	ORF-T	-0.25033941	-0.133211999	-0.205166611	-0.21110847	0.788288384
837607	838239	12	+	CUT272	CUTs	CUTs	1.553681701	0.350445315	-0.166727069	-0.345746896	-0.352515263
839807	840279	12	+	CUT273	CUTs	CUTs	2.493640043	-0.098153076	0.285136367	0.084857226	-0.207940391
840279	841047	12	+	YLR356W	ORF-T	ORF-T	-0.857576232	-0.01308559	1.2237979	0.511215376	1.18126995
841287	844191	12	+	RSC2	ORF-T	ORF-T	-0.099843881	-0.336075863	-0.575747855	-0.164708325	-0.129869355
844191	845855	12	+	ADE13	ORF-T	ORF-T	0.552028069	-0.05351426	-0.085053834	-0.187785019	-0.528424302
846063	847575	12	+	VPS38	ORF-T	ORF-T	-0.089939474	-0.351148695	0.282887498	0.01209515	0.536218408
849839	852095	12	+	STE11	ORF-T	ORF-T	-0.009204606	-0.128199478	0.277234994	-0.125603015	0.076496793
853447	854047	12	+	YLR363W-A	ORF-T	ORF-T	0.336202079	-0.316952891	-0.402911956	-0.305878038	0.411849147
854047	854455	12	+	YLR364W	ORF-T	ORF-T	1.119935261	1.312141347	0.509808683	0.063184764	-0.08529429
855823	856103	12	+	YLR366W, RF	other	other	-1.427117259	-1.203374616	0.203409547	-0.06427645	-0.506134802
855879	856103	12	+	unknown	no_ovlp2	CUTs	1.896660779	-0.559436535	-0.552065293	-0.454405315	0.051298844
856719	857375	12	+	RPS22B, SNR	ORF-T	ORF-T	-0.326743121	-0.367197482	-0.372294879	-0.290816999	0.153898958
857375	859559	12	+	MDM30	ORF-T	ORF-T	0.158828797	-0.278823507	0.047159915	-0.188321355	0.031939297
859559	861591	12	+	SSQ1	ORF-T	ORF-T	-0.380474133	-0.809147999	0.283927777	-0.029803981	-0.256970686
862527	866951	12	+	ROM2	ORF-T	ORF-T	-0.050507351	-0.300015269	-0.274397475	-0.108183673	-0.221886043
867159	868655	12	+	SUR4	ORF-T	ORF-T	-0.198104288	-0.46623257	-0.725669927	-0.39879535	-0.220283985
871671	872823	12	+	STP3	ORF-T	ORF-T	0.090037311	-0.288228064	-0.291350475	-0.222380218	-0.12940555
873751	874967	12	+	SUT281	SUTs	SUTs	0.799804428	-1.043272852	0.047483287	-0.10011386	-0.004187738
877815	878175	12	+	unknown	no_ovlp	CUTs	1.422180704	0.296448832	-0.784226086	-0.410987009	-0.327166391
878175	879575	12	+	CSR1	ORF-T	ORF-T	-0.500420516	-0.319068511	-0.451333047	-0.028992576	0.154196359
879575	881991	12	+	CTF3	ORF-T	ORF-T	-0.09914344	-0.252396887	-0.007715397	-0.174210146	0.510003912
885215	888735	12	+	SMC6	ORF-T	ORF-T	-0.353787121	-0.252824255	0.059733646	-0.199870603	0.712367603
893591	896407	12	+	VAC14	ORF-T	ORF-T	0.052822812	0.078816574	0.010373735	0.075203823	0.456814208
898151	898655	12	+	RPS29A	ORF-T	ORF-T	0.277025394	-1.696757184	-0.817972112	-1.156810007	-0.010113807
899207	899591	12	+	SNR34	other	other	2.96389467	-2.652112576	0.036124987	-0.421363844	0.197038246

902855	903135	12	+	ECM19	ORF-T	ORF-T	-1.764502972	-1.486767034	0.430591198	-0.21731905	0.199251531
903655	904655	12	+	CCW14	ORF-T	ORF-T	-0.329894731	-0.009936935	0.30155564	-0.132686817	0.295900833
907047	909527	12	+	ATP10, CST9	ORF-T	ORF-T	0.010278227	-0.116566776	0.260349439	-0.205923728	0.236967656
910271	911095	12	+	SUT282	SUTs	SUTs	1.650352985	-1.626624117	-0.827128057	-0.226520733	-0.076434094
921111	924936	12	+	unknown	no_ovlp	SUTs	0.091859846	0.350073393	-0.007019348	0.149625047	0.433566252
924936	927136	12	+	YLR402W, SF	other	other	0.128366437	-0.60485709	-0.312063532	-0.174428861	-0.268128287
928752	929800	12	+	YLR404W	ORF-T	ORF-T	-0.932465243	-0.76485562	0.407720467	-0.223615166	-0.749678839
929800	931072	12	+	DUS4	ORF-T	ORF-T	0.363327111	-0.051254964	-0.458486241	-0.489753291	0.226794103
932736	933800	12	+	YLR407W	ORF-T	ORF-T	0.209751876	-0.313613759	-0.150409775	-0.088537976	0.39985745
937504	941064	12	+	VIP1	ORF-T	ORF-T	-0.082920142	0.10786712	0.221291689	-0.006235247	0.387068205
948344	949304	12	+	YLR412W	ORF-T	ORF-T	-0.265532033	-1.218599805	-0.105707616	0.023107024	-0.298741511
951104	953320	12	+	YLR413W	ORF-T	ORF-T	-0.084910515	-0.881105339	-1.385653353	0.286187862	-0.189103551
955000	956800	12	+	VPS36	ORF-T	ORF-T	0.363982901	-0.698716893	-0.230216261	-0.168601995	-0.314575478
958384	962784	12	+	YLR419W	ORF-T	ORF-T	0.100739936	-0.289321773	-0.4372044	0.101935137	0.051465963
963736	964960	12	+	URA4	ORF-T	ORF-T	-0.035154178	0.566804257	-0.119023167	-0.204637757	-0.073330385
965792	971752	12	+	YLR422W	ORF-T	ORF-T	0.137389014	-0.275875521	-0.212707351	-0.041749156	-0.00119119
971752	973304	12	+	unknown	no_ovlp2	CUTs	1.772783852	-0.158788519	-1.59795215	-0.725119516	-0.881002031
973304	975528	12	+	SPP382	ORF-T	ORF-T	0.127492703	-0.516345763	-0.496050517	-0.792951507	-0.217426352
982744	986888	12	+	TUS1	ORF-T	ORF-T	-0.041198329	-0.214263695	0.152340383	0.127353006	0.009460223
987048	988184	12	+	YLR426W	ORF-T	ORF-T	0.078189334	-0.42322405	-0.393612585	-0.424109402	-0.290125698
988344	990600	12	+	MAG2	ORF-T	ORF-T	-0.159018606	-0.30651561	-0.063509194	-0.115892398	0.216789025
990752	992904	12	+	CRN1	ORF-T	ORF-T	-0.313059391	-0.155169204	-0.554343379	-0.011536303	-0.046292974
993152	997440	12	+	SEN1	ORF-T	ORF-T	-0.008013696	-0.043931696	-0.136273346	0.027438161	0.411041655
1002328	1004208	12	+	IMD3	ORF-T	ORF-T	-0.243737928	-0.597219993	-0.775387502	0.186524821	0.47491937
1004392	1006360	12	+	unknown	no_ovlp	SUTs	0.128764625	0.473802754	0.477536581	0.05521787	1.128699922
1006360	1007160	12	+	TSR2	ORF-T	ORF-T	0.236734053	-0.601169459	-0.700610783	-0.333978806	0.224085535
1010976	1011632	12	+	unknown	no_ovlp	SUTs	0.252298018	-0.195306543	-0.347503032	0.343084002	-0.489800897
1011632	1012456	12	+	unknown	no_ovlp2	CUTs	1.095212391	-0.991665755	0.183253103	0.047817312	0.144323246
1012456	1013776	12	+	CAR2	ORF-T	ORF-T	-0.330298192	-0.65831448	0.722924182	0.16405237	1.879927415
1014448	1015568	12	+	MRPL4	ORF-T	ORF-T	-0.428133623	-0.518820908	-0.234128186	-0.208250822	-0.074297178
1022488	1024072	12	+	ECM7	ORF-T	ORF-T	-0.145222189	-0.094694329	0.091403059	-0.240487904	0.569148977
1025152	1027360	12	+	YLR446W	ORF-T	ORF-T	0.136747237	-0.056278383	1.25933501	0.400001462	1.606723258
1027112	1027360	12	+	unknown	no_ovlp2	CUTs	1.829063705	0.480250854	-2.06166358	-0.773885523	-0.43596843
1028144	1028776	12	+	CUT277	CUTs	CUTs	0.777625033	0.533664088	-0.151075365	-0.029710804	-0.690067189
1028864	1029256	12	+	RPL6B	ORF-T	ORF-T	0.360064291	-0.630797664	-0.532463445	-0.886383188	-0.304620281
1030080	1030816	12	+	SUT284	SUTs	SUTs	-0.465263381	0.384503621	0.097797414	-0.025213603	1.270878458
1030816	1032096	12	+	FPR4	ORF-T	ORF-T	0.597467027	0.246522633	-0.5409282	-0.16479479	0.034051195
1032456	1033488	12	+	HMG2	ORF-T	ORF-T	-0.686589075	-0.736488396	0.070421601	0.36131792	0.763349063
1036000	1038904	12	+	LEU3	ORF-T	ORF-T	0.129995033	-0.562683872	-0.255132167	0.02755366	0.269848394
1041760	1042576	12	+	CUT278	CUTs	CUTs	2.267780908	-0.248333242	-1.672319293	-0.616536234	-0.710069993
1043944	1045880	12	+	FMP27	ORF-T	ORF-T	-0.215793238	-0.419958381	0.151987975	0.124351245	0.224094131
1053400	1054912	12	+	YLR455W	ORF-T	ORF-T	-0.544620463	-0.485933311	0.241423677	-0.239841999	0.398773463
1054912	1055792	12	+	YLR456W	ORF-T	ORF-T	-0.269481489	-0.243935709	0.598568927	0.439795404	1.22378232
1057328	1058576	12	+	GAB1	ORF-T	ORF-T	-0.591551518	-0.60221081	-0.317267738	-0.024807712	-0.049193842
11469	13269	13	-	ERO1	ORF-T	ORF-T	-0.245793861	-0.423619358	-0.194478032	-0.206817912	-0.323286167
14301	14765	13	-	COX14	ORF-T	ORF-T	-0.724371992	-0.96314386	0.328984945	0.084839219	-0.187703203
14949	16773	13	-	MSC1	ORF-T	ORF-T	-1.012361688	-1.284249361	1.988409268	1.501070301	1.188890168
18853	20477	13	-	ERG13	ORF-T	ORF-T	-0.025705533	0.023132583	-0.400413263	0.170196436	0.210273116
20685	21725	13	-	PGA3	ORF-T	ORF-T	0.353037924	0.149931819	-0.069760794	-0.276850389	0.075387889
23357	23709	13	-	TUB3	ORF-T	ORF-T	0.622743886	0.280540746	-0.649008991	-0.897262993	0.883299437
24029	25733	13	-	PHO84	ORF-T	ORF-T	-3.345528967	2.31251292	3.122696043	4.759741258	5.107965378
27909	29869	13	-	NDI1	ORF-T	ORF-T	-0.504814932	-0.165990881	0.899822504	0.357725948	-0.004107372
32877	33909	13	-	unknown	no_ovlp	CUTs	1.354230477	-0.552404834	-0.330373594	-0.262021621	-1.812915582
40109	41853	13	-	VAN1	ORF-T	ORF-T	-0.191242883	-0.714521334	-0.343778252	-0.132289415	-0.258193617
41981	43581	13	-	TAF8	ORF-T	ORF-T	-0.156061463	-0.507612022	-0.048898376	-0.014938982	-0.065006561
43581	44293	13	-	unknown	no_ovlp	SUTs	0.141883006	0.498996338	0.658971756	0.290507755	0.258261281
45925	46573	13	-	SUT716	SUTs	SUTs	1.499163386	-1.746105008	-0.494045945	-0.096399266	0.116212145
49757	50981	13	-	COQ5	ORF-T	ORF-T	-0.543123714	-0.490754562	0.459486135	0.093554898	0.529727323
55277	56301	13	-	PML39	ORF-T	ORF-T	0.242617724	-0.171948482	-0.172950539	-0.140482839	-0.227341777
57789	58701	13	-	SEC65	ORF-T	ORF-T	0.069734517	-0.759667272	-0.459167815	0.01917958	-0.11069179
58701	62357	13	-	MDM1	ORF-T	ORF-T	-0.080814836	-0.564979308	-0.444852503	-0.277147462	0.401121257
62485	67709	13	-	NUP188	ORF-T	ORF-T	-0.234970058	-0.484679194	0.038650747	0.23971533	0.180664129
67709	67949	13	-	SNR85	other	other	2.716300339	-0.67798234	0.179889878	-0.056770088	0.1106645
69661	70133	13	-	YML101C-A,	other	other	-0.741809532	-0.891430049	-0.11767211	-0.331918682	-0.859849549
70133	71445	13	-	unknown	no_ovlp	SUTs	-0.310204094	0.34176905	0.166937948	0.066632954	0.354876868
74301	76989	13	-	ARG81	ORF-T	ORF-T	0.916774912	-0.343397352	-0.545148489	-0.262468626	-0.395933219
78261	79709	13	-	VPS9	ORF-T	ORF-T	-0.158179404	-0.552995893	-0.255030603	-0.435518933	0.145250087
80581	82133	13	-	RAD10	ORF-T	ORF-T	0.500243962	-0.138604819	0.115456799	-0.239947375	-0.007408807
82133	82877	13	-	unknown	no_ovlp	CUTs	1.341626786	0.37094513	-0.563999531	-0.500872736	1.158206776
85901	86773	13	-	PRE8	ORF-T	ORF-T	-0.209547125	-0.740257499	0.118984028	-0.174988645	-0.182956999
89813	91045	13	-	RPM2	ORF-T	ORF-T	-0.006434865	-0.549882542	0.518392205	0.054991581	-0.102631152
91045	92581	13	-	YML089C	other	other	0.301594517	0.292664625	1.169159782	-0.32659374	0.200594642
91597	92581	13	-	unknown	no_ovlp2	SUTs	0.749055145	0.35332983	-0.267194621	-0.328675055	0.056879683
92581	94341	13	-	unknown	no_ovlp2	CUTs	2.274110593	0.493373077	-0.340782181	-0.508738677	0.038840959
94341	95381	13	-	YML087C	ORF-T	ORF-T	-0.556735885	-1.075814112	0.222913177	0.507554335	1.062972256
95685	97525	13	-	ALO1	ORF-T	ORF-T	-0.593859897	-0.436544621	-0.013326564	0.395330226	0.153503737
97797	99261	13	-	TUB1	ORF-T	ORF-T	-0.399219701	-0.356674169	0.170422789	0.113564852	0.796376756
103829	104221	13	-	ATP18	ORF-T	ORF-T	-0.49937766	-0.428090486	0.504033628	-0.278922276	0.224057436
110501	110837	13	-	CUT761	CUTs	CUTs	3.328777866	0.665893798	0.258799735	-0.197580928	-0.313402533
110837	111653	13	-	unknown	no_ovlp	SUTs	0.595121283	-0.731612983	-1.165742521	-0.275391034	-0.889898046
112421	115357	13	-	WAR1	ORF-T	ORF-T	0.237954249	-0.476651723	-0.613306728	-0.294230105	0.009677604
115589	119045	13	-	HMG1	ORF-T	ORF-T	-0.117387086	-0.305800093	-0.372811625	0.193354336	-0.195070268

120013	121365	13	-	FPR3	ORF-T	ORF-T	0.235484313	-0.199275986	-0.609906087	-0.075496085	-0.348533456
123741	124165	13	-	RPL6A	ORF-T	ORF-T	0.634723839	-0.772777487	-1.33384593	-1.425499846	-0.201437482
124589	129557	13	-	TCB3	ORF-T	ORF-T	-0.289689799	-0.527500719	-0.732699779	-0.114991233	0.060735429
129717	131717	13	-	COG8	ORF-T	ORF-T	-0.437360895	-0.55139652	-0.068747	-0.191285693	-0.377754592
131717	133549	13	-	unknown	no_ovlp	SUTs	0.336336931	0.352964432	0.856343174	0.698130069	-0.169080045
139005	140061	13	-	ERV41	ORF-T	ORF-T	-0.135220808	-0.750138352	-0.507932364	-0.088052711	-0.703577515
141485	141997	13	-	SMA2	ORF-T	ORF-T	1.436069566	0.274666286	0.116626423	-0.513938518	-0.545571666
144965	145957	13	-	TEM1	ORF-T	ORF-T	-0.333442385	-0.232683018	0.484901158	-0.306678618	0.901607196
147445	148701	13	-	MFT1	ORF-T	ORF-T	0.525592839	-0.136715187	-0.411934035	-0.257887542	-0.492690885
148869	151669	13	-	PIF1	ORF-T	ORF-T	0.206513131	0.039479726	-0.275433915	-0.469041458	0.901204361
157509	158285	13	-	NTE1	ORF-T	ORF-T	-0.195248	-0.664862728	-0.117960387	-0.132743523	0.228034349
163725	164269	13	-	IMD4, SNR54	ORF-T	ORF-T	-0.350289882	-0.640799864	-0.926565633	-0.221056868	0.379992998
165420	167389	13	-	CYB2	ORF-T	ORF-T	-1.481565005	-0.302148048	2.613999539	2.720147223	0.592754301
168957	169821	13	-	YML053C	ORF-T	ORF-T	-0.432105638	-0.714673938	0.276996209	-0.070685766	0.559696942
169821	172533	13	-	SUT719	SUTs	SUTs	0.439393896	0.301519164	-0.161113094	-0.232874803	0.256988717
176301	178173	13	-	RSE1	ORF-T	ORF-T	0.154944069	-0.163511088	-0.006890312	-0.238890752	0.179529734
190117	191781	13	-	RRN11	ORF-T	ORF-T	0.586712776	0.137061678	-0.41532531	-0.461514944	1.109826342
194853	195789	13	-	VPS71	ORF-T	ORF-T	0.158906455	-0.415255016	-0.082222306	0.012286724	0.099497875
202573	204237	13	-	YMD8	ORF-T	ORF-T	0.001895603	-0.360897058	-0.571182337	-0.132457781	0.033586432
204237	205437	13	-	YML037C	ORF-T	ORF-T	-0.273276667	-0.402509015	-0.008308962	-0.453321647	0.068198821
206117	209061	13	-	AMD1	ORF-T	ORF-T	-0.028414589	-0.419211286	-0.066346007	-0.22734111	-0.153742852
212501	213965	13	-	RAD52	ORF-T	ORF-T	-0.095888361	-0.429806544	0.063953611	-0.115284916	0.080996802
219085	219845	13	-	SUT720	SUTs	SUTs	1.660531614	-0.711832774	-0.49798267	-0.126727672	0.7963915
219845	220733	13	-	unknown	no_ovlp	CUTs	1.260986945	-0.285749574	-0.964530665	-0.691418947	-0.767207927
223381	223829	13	-	RPS18B	ORF-T	ORF-T	0.575251849	-1.117334318	-1.074129445	-1.282756685	0.26331738
224301	225237	13	-	YML6	ORF-T	ORF-T	-0.698544924	-0.572146724	0.001752788	-0.119837792	0.210153059
226941	228701	13	-	NSE5	ORF-T	ORF-T	-0.079396443	-0.636374831	-0.403841079	-0.689962831	0.194440031
229645	230789	13	-	UNG1	ORF-T	ORF-T	-0.251344942	0.166280519	-0.201885655	0.230470941	0.250868334
230789	231445	13	-	unknown	no_ovlp	SUTs	0.02650563	-0.477404385	-0.377652769	-0.382317034	0.365558498
234653	236085	13	-	YML018C	ORF-T	ORF-T	0.261414482	-0.265950181	-1.127004694	-0.889874978	0.307779685
240573	241797	13	-	PPZ1	ORF-T	ORF-T	-0.16510469	-0.536086149	-0.040540408	-0.375495029	-0.22671489
241965	243053	13	-	TAF11	ORF-T	ORF-T	0.397256735	0.601963157	0.181841854	-0.257994021	0.469753167
244973	245845	13	-	unknown	no_ovlp	CUTs	1.88981501	0.584142409	-1.477209461	-0.429474776	0.745910353
246757	247453	13	-	RAD33	ORF-T	ORF-T	-0.202550018	-0.356082864	-0.10790809	-0.463646701	0.159841043
251125	251549	13	-	YML009C-A,	other	other	-1.030547465	-0.671498368	0.299905648	-0.196421754	0.597173151
251749	253021	13	-	ERG6	ORF-T	ORF-T	-0.397954763	-0.676098749	-0.464689895	0.001850896	-0.228479513
253021	253269	13	-	YML007C-A	ORF-T	ORF-T	0.897172962	-0.210143326	-0.015521016	-0.0986686	-0.579827298
256525	258949	13	-	GIS4	ORF-T	ORF-T	0.171279012	-0.877089111	-0.54302114	0.14747002	-0.234258199
258573	258949	13	-	unknown	no_ovlp2	SUTs	0.353124259	-0.819410026	-1.366899948	-0.106923682	-0.800548812
259405	260045	13	-	SUT722	SUTs	SUTs	2.065534231	-0.097450631	-0.247745403	-0.542929786	-0.555218666
261565	262701	13	-	GLO1	ORF-T	ORF-T	-0.03576437	-0.415371801	0.30330046	0.045124483	-0.503966941
262701	263157	13	-	SUT723	SUTs	SUTs	-0.127442235	-0.562530517	0.437481172	-0.09745828	-0.439163718
265581	267493	13	-	SUT724	SUTs	SUTs	0.92402075	0.359525209	-0.004292321	-0.353246268	-0.03335549
268861	271485	13	-	CDC5, YMR0	other	other	0.001346431	-0.352062574	-0.593505713	-0.229438316	0.241492759
271485	271909	13	-	SUT725	SUTs	SUTs	0.540845722	0.824685398	-0.13893515	-0.416760673	-0.293675579
277469	279373	13	-	PLB2	ORF-T	ORF-T	-0.529040651	-1.24659343	-1.242314352	0.458479232	1.069200382
280253	282781	13	-	PLB1	ORF-T	ORF-T	0.048174886	-0.133467507	-0.465825089	0.045104994	-0.854040862
282781	284621	13	-	SUT726	SUTs	SUTs	1.195568418	0.099410203	-0.087773861	-0.171534999	-0.438682912
286717	287365	13	-	CUT764	CUTs	CUTs	0.16167304	0.497345374	1.617281834	1.429671104	-0.608065435
294989	296789	13	-	SEC59, YMR0	ORF-T	ORF-T	-0.349740451	-0.462964971	-0.23314607	0.053340795	0.442628246
300685	302573	13	-	ERG5	ORF-T	ORF-T	-0.082918629	-0.514559737	-0.675480112	0.168605027	0.063177129
303117	305821	13	-	SOK2	ORF-T	ORF-T	0.025697092	-0.667523546	-0.699705522	-0.142377568	-0.579469737
308045	308781	13	-	unknown	no_ovlp	CUTs	1.345618574	0.334005078	1.169920226	2.000547724	-1.662046318
311269	312029	13	-	SUT728	SUTs	SUTs	3.077909038	-0.035712371	-0.486408539	0.109714988	-2.830210736
316925	318445	13	-	MAC1	ORF-T	ORF-T	-0.299764347	-0.504078228	-0.0701758	0.104105069	0.179056261
319389	321069	13	-	MSS1	ORF-T	ORF-T	-0.767278354	-1.573180566	-0.029558616	0.451748362	-0.104112275
324149	325549	13	-	PEX12	ORF-T	ORF-T	-0.40743705	-0.645852874	-0.131311206	-0.259885096	0.109449179
328573	330349	13	-	FAR8	ORF-T	ORF-T	-0.279071985	-0.939176189	0.185683412	0.353080767	-0.272352104
332021	334813	13	-	YMR031C	ORF-T	ORF-T	-0.582332607	-0.914827833	0.444597876	0.62856795	0.093642172
339429	340933	13	-	YMR034C	ORF-T	ORF-T	-0.223223142	-0.083962109	0.623375793	-0.40226278	2.026710461
341749	343557	13	-	MIH1	ORF-T	ORF-T	0.085338124	-0.054208433	0.556297271	0.060664057	0.639776529
344141	346925	13	-	MSN2	ORF-T	ORF-T	-0.126819382	-0.550461789	-0.358681957	-0.289049741	-0.220095952
347413	348285	13	-	CCS1	ORF-T	ORF-T	0.024749809	-0.303039428	-0.688695793	-0.014928877	-0.300277203
348477	349733	13	-	SUB1	ORF-T	ORF-T	-0.267298673	-0.667092605	0.105376084	0.026539896	-0.293739811
350957	351941	13	-	ARA2	ORF-T	ORF-T	0.042221971	-0.812773499	0.21204063	-0.201812926	-0.380088158
352413	352829	13	-	CUT767	CUTs	CUTs	1.777404858	-0.172231622	-0.166098926	-0.550595032	-0.053430234
352829	353117	13	-	unknown	no_ovlp	SUTs	0.772325728	0.183786481	-0.59462391	-0.067861862	-0.229329968
354405	355125	13	-	CUT768	CUTs	CUTs	0.786082421	0.399307445	0.76069558	1.142417082	0.751973308
356853	362917	13	-	unknown	no_ovlp	SUTs	0.810805832	-0.413689519	-0.494288267	-0.462082584	-0.367818331
363293	366781	13	-	NUP116	ORF-T	ORF-T	-0.122192303	-0.229976046	-0.286824735	0.101127306	-0.286009143
367933	370549	13	-	ERB1	ORF-T	ORF-T	0.411641666	-0.057742026	-0.876757841	-0.049289811	-0.030339072
378621	379165	13	-	unknown	no_ovlp	CUTs	1.088638853	0.025771971	0.93254323	0.334734718	0.117719404
380029	380365	13	-	unknown	no_ovlp2	SUTs	0.894192648	0.182842725	-0.304732508	-0.486558481	0.340829675
380365	382909	13	-	YMR052C-A,	ORF-T	ORF-T	-0.456390181	-0.069537768	0.968778874	0.434565919	0.8532844
386013	388053	13	-	BUB2	ORF-T	ORF-T	-0.66503658	-0.564789998	0.117311804	0.033453126	0.637259536
387061	388053	13	-	unknown	no_ovlp2	SUTs	-0.189258336	-0.43899981	0.680896651	0.32244221	0.529527163
391517	392533	13	-	SAM37	ORF-T	ORF-T	-0.131522424	-0.849270131	-0.000834275	-0.446647856	0.000849396
395005	396397	13	-	ECM40	ORF-T	ORF-T	0.148736209	-0.693805334	-0.407132057	-0.001319753	-0.82471639
396397	396837	13	-	CUT769	CUTs	CUTs	1.510996325	0.529408485	-0.177429955	-0.475783475	-0.153795596
404373	405597	13	-	UBX4	ORF-T	ORF-T	0.48935589	-0.038605828	-0.25482221	-0.389758201	-0.223016332
405597	406045	13	-	CUT771	CUTs	CUTs	1.282706875	-1.091894433	-0.126955537	-0.19564537	-0.109597553
406045	409173	13	-	unknown	no_ovlp	SUTs	0.089390209	0.388777045	0.037204078	-0.001274483	0.460089354

409269	410453	13 -	unknown	no_ovlp	SUTs	-0.172417661	0.464709552	0.290246466	0.383761151	0.394563049
410741	411325	13 -	TVP18	ORF-T	ORF-T	-0.124183733	-0.234761086	0.452286651	-0.236490144	0.593098922
412173	412989	13 -	IRC21, YMR0	ORF-T	ORF-T	0.297431314	-0.35012984	0.162190658	-0.342920841	0.162730523
412989	413485	13 -	YMR074C	ORF-T	ORF-T	0.77273031	-0.133638613	-0.406760927	-0.119480705	-0.378958598
416013	420109	13 -	YMR075C-A,	other	other	-0.403643603	-0.317427557	-0.252112125	-0.0037185	0.560707581
421325	422181	13 -	VPS20	ORF-T	ORF-T	0.301789439	-0.470587761	0.136414509	0.187750606	-0.36079932
422365	424741	13 -	CTF18	ORF-T	ORF-T	-0.634846351	-0.04602137	0.042527119	-0.14267364	1.137997643
428541	429709	13 -	NAM7	ORF-T	ORF-T	0.195002331	-0.03696427	-0.340171262	-0.008983686	0.065555286
430477	431277	13 -	ISF1	ORF-T	ORF-T	-0.537981213	-0.94715357	1.13363375	1.400595319	-0.170530532
433117	434205	13 -	SUT729	SUTs	SUTs	1.017587634	-0.951624319	-0.064702874	0.146098043	-1.201209428
443301	445245	13 -	VBA1	ORF-T	ORF-T	-0.502032567	0.017529839	-0.291008996	-0.052728136	0.599375234
445485	448309	13 -	YTA12	ORF-T	ORF-T	-0.13407267	-0.383105808	-0.027045844	-0.055440264	-0.605083569
449973	451413	13 -	NPL6	ORF-T	ORF-T	1.096759157	0.685128328	-0.535433926	-0.114290636	-0.340350892
451557	453557	13 -	AIP1	ORF-T	ORF-T	-0.198209033	-0.175604574	0.374590795	-0.110778561	0.130845842
456901	457197	13 -	unknown	no_ovlp2	SUTs	-0.471855017	-1.613720595	0.241814295	1.082608825	0.520405543
457197	457973	13 -	SNO1	ORF-T	ORF-T	-0.54296092	-1.846573145	0.572044711	1.250655788	0.76006858
459365	460501	13 -	MTG1	ORF-T	ORF-T	0.585355767	-0.063808434	0.22780621	-0.339008124	-0.012869047
460709	462717	13 -	YMR098C	ORF-T	ORF-T	-0.362498329	-0.600608345	-0.075586228	-0.104830054	-0.480933196
463853	464837	13 -	YMR099C	ORF-T	ORF-T	0.308648285	-0.540406461	-0.215174027	-0.027630828	-0.292032381
464837	465901	13 -	SUT730	SUTs	SUTs	0.143297282	0.104753162	-0.477389177	-0.217985675	1.117347724
469709	475501	13 -	YMR102C	ORF-T	ORF-T	0.577094051	-0.57936229	-0.755791103	-0.337150086	-0.2971696
472645	475501	13 -	unknown	no_ovlp2	SUTs	0.08560916	-0.28412507	0.260064285	0.446928307	-0.854834188
475757	477661	13 -	PGM2	ORF-T	ORF-T	-1.57712357	-1.756235522	1.196697092	1.46949156	0.066141183
477661	480061	13 -	YKU80	ORF-T	ORF-T	-0.267592902	-0.069210077	0.168005498	0.265093606	0.541583711
482045	482389	13 -	unknown	no_ovlp	CUTs	1.761457213	0.162263553	0.905101141	0.889298756	-2.181578378
482389	483709	13 -	unknown	no_ovlp	SUTs	0.027999517	0.230050775	0.131959756	0.263535244	-0.324707488
489565	489845	13 -	SUT731	SUTs	SUTs	-0.373903363	-0.614611915	-0.58029393	-0.095844796	-0.131313346
490397	492045	13 -	HFD1	ORF-T	ORF-T	-0.117073362	-0.554857306	0.689665995	0.233225624	-0.003019642
492253	493989	13 -	YMR111C	ORF-T	ORF-T	-0.112893561	-0.286656311	-0.066409636	-0.332415258	-0.449856293
493989	494453	13 -	MED11	ORF-T	ORF-T	0.142904529	-0.243087877	-0.088280042	-0.216241719	0.045692227
494453	494797	13 -	unknown	no_ovlp	SUTs	0.28907712	-0.66386202	-0.079215647	-0.733772717	-0.130664519
496221	497493	13 -	YMR114C	ORF-T	ORF-T	-0.137984989	-0.120554751	0.887481487	0.240856269	0.531310524
500165	500717	13 -	ASC1, SNR24	ORF-T	ORF-T	-0.525028564	0.173573975	-0.396826501	-0.168423102	0.211954599
500717	501909	13 -	SPC24	ORF-T	ORF-T	-0.170495494	-0.39734153	0.269753841	-0.624762211	0.526603367
507341	509325	13 -	ADE17	ORF-T	ORF-T	0.996511083	0.996539973	0.113530855	-0.082349091	-0.248024875
509677	510677	13 -	RPL15B	ORF-T	ORF-T	-1.108683141	-0.770224845	-0.288918543	-0.236350742	0.554996643
510405	510677	13 -	unknown	no_ovlp2	CUTs	1.851382183	-0.066852621	-0.783114941	-0.296047622	0.073835842
512077	513293	13 -	CUT775	CUTs	CUTs	0.987093252	0.167701936	0.500325288	0.241870925	-0.117858116
513293	515701	13 -	unknown	no_ovlp	SUTs	0.261611643	0.554219702	0.004675133	-0.138459073	0.021992214
520701	523373	13 -	DLT1	ORF-T	ORF-T	0.218481067	-0.496741305	-0.408866284	-0.153760372	-0.425379195
522037	523373	13 -	unknown	no_ovlp2	SUTs	-0.19190795	-0.858397489	-0.652614214	-0.451884129	0.943432361
533069	534741	13 -	RRB1	ORF-T	ORF-T	-0.044309107	-0.186116197	-0.527451571	-0.018343712	0.393982577
534741	535621	13 -	JLP2	ORF-T	ORF-T	-0.032947559	-0.480268457	0.42221773	-0.401289844	0.087586142
536045	537485	13 -	SUT732	SUTs	SUTs	0.618939419	0.093775133	1.425913076	1.400169564	1.644196053
538557	540085	13 -	GID8	ORF-T	ORF-T	0.066953615	-0.365164852	-0.079198725	0.019079953	-0.228367253
540085	540613	13 -	unknown	no_ovlp	SUTs	0.433331792	0.91876469	-0.503442333	-0.119728559	0.033916696
542981	545317	13 -	PSO2	ORF-T	ORF-T	-0.381962969	-0.32607426	0.666508838	-0.000332912	0.368383561
545021	545317	13 -	unknown	no_ovlp2	CUTs	1.414014825	0.023894498	-0.038548191	-0.040828958	1.120595109
545317	545709	13 -	CUT776	CUTs	CUTs	3.305355786	0.021042829	-0.041426569	-0.090409183	0.111965022
550797	551301	13 -	RPL13B	ORF-T	ORF-T	0.846966813	-0.710390346	-1.134659413	-1.02035958	-0.337562779
554597	556701	13 -	NDE1	ORF-T	ORF-T	0.302923858	-0.018897377	-0.074107682	-0.078484896	-0.192712828
557381	558589	13 -	TIF34	ORF-T	ORF-T	-0.342255186	-0.601138712	-0.587168223	-0.148864726	-0.318359004
559749	560629	13 -	CUT777	CUTs	CUTs	1.219422433	-0.707245719	-0.466634188	-0.499653557	-0.403492615
561949	562885	13 -	IMP1	ORF-T	ORF-T	-0.509704861	-0.114066559	0.568719016	0.458453296	0.341807864
565677	568365	13 -	YMR153C-A,	other	other	0.627653834	-0.073217903	-0.125355386	-0.181923273	-0.66165104
570229	571157	13 -	TPP1	ORF-T	ORF-T	-0.366496046	-1.199817229	0.03620436	-0.092349198	0.027106725
571157	572093	13 -	FMP39	ORF-T	ORF-T	-0.540668374	-0.48597199	0.252567973	0.017616461	0.160684439
573109	573357	13 -	unknown	no_ovlp2	SUTs	-0.443828207	-0.633206026	0.3004333	-0.143883314	-0.052542536
573357	574861	13 -	ATG16	ORF-T	ORF-T	-0.025650021	-0.589875423	0.168051872	-0.295700433	-0.24101205
576813	577453	13 -	CUT779	CUTs	CUTs	2.265059937	-0.047671184	-1.99286024	-0.207686728	-0.163899415
578765	584045	13 -	DNF3	ORF-T	ORF-T	0.026951964	-0.510022257	-0.527341086	-0.103866021	-0.490626932
584045	587181	13 -	INP2	ORF-T	ORF-T	-0.608161851	-0.065562394	0.302637661	-0.436483392	1.056577671
586533	587181	13 -	unknown	no_ovlp2	SUTs	-0.147381902	-0.665766884	0.762861906	-0.043568497	0.654442129
587181	589637	13 -	MSS11	ORF-T	ORF-T	-0.132938638	-0.539385728	0.072100903	0.330048567	-0.061934445
591741	592749	13 -	PAH1	ORF-T	ORF-T	0.060758103	-0.767864858	-0.098227244	0.375311858	0.042534247
593157	594725	13 -	YMR166C	ORF-T	ORF-T	-0.072873961	-0.172865745	-0.105384761	0.008611488	-0.427709348
596509	599181	13 -	CEP3	ORF-T	ORF-T	0.227432725	-0.606247593	-0.649948921	-0.149484126	-0.17341653
599349	600885	13 -	ALD3	ORF-T	ORF-T	-0.149435896	0.425985836	1.488919137	0.487182121	1.207696159
600885	601493	13 -	unknown	no_ovlp2	SUTs	-0.234665094	0.378653251	0.038069574	0.118925177	0.258160449
601493	603621	13 -	ALD2	ORF-T	ORF-T	-0.229088676	0.307237358	-0.062546138	0.065816168	-0.019051537
603213	603621	13 -	unknown	no_ovlp2	SUTs	0.019353082	0.316735515	0.770821349	-0.090647241	0.420688223
603621	605701	13 -	YMR171C	ORF-T	ORF-T	0.107660926	-0.494620622	-0.202773903	-0.135831606	-0.105744573
610029	610493	13 -	PAI3	ORF-T	ORF-T	-0.304713672	0.25024447	2.419254554	0.697436306	-0.064739925
610493	611541	13 -	unknown	no_ovlp	SUTs	0.582889476	0.317945543	0.520053426	0.465684741	-0.506956619
618893	619493	13 -	SUT734	SUTs	SUTs	1.892385993	-0.565767629	-1.152215248	-0.054245169	-0.212446582
622149	623357	13 -	CTL1	ORF-T	ORF-T	0.999873896	-0.41607819	-0.339841968	-0.274041734	-0.179621821
623357	624253	13 -	YMR181C	ORF-T	ORF-T	-0.210912901	-0.612567931	0.581315172	0.334507187	0.695663785
624253	625277	13 -	RGM1	ORF-T	ORF-T	0.489556497	-0.253637745	0.558667254	0.281270653	1.097367954
625277	625597	13 -	unknown	no_ovlp	SUTs	0.921081929	-1.109886078	0.146600021	-0.497792893	-0.199445978
626693	627877	13 -	SSO2	ORF-T	ORF-T	0.063783006	-0.296807543	-0.276565428	-0.197345433	-0.169286986
628381	628733	13 -	CUT780	CUTs	CUTs	2.748608794	-0.145570721	-0.012830143	-0.546627074	0.308006921
634549	636181	13 -	YMR187C	ORF-T	ORF-T	0.181620839	0.154438852	0.283577658	-0.362361389	0.25059055

636181	637061	13	-	MRPS17	ORF-T	ORF-T	-0.97484192	-0.94062111	0.210211285	0.059643021	-0.00331094
640789	645413	13	-	SGS1	ORF-T	ORF-T	-0.048080838	0.145914412	0.122788905	-0.08600193	0.735315951
646445	646853	13	-	CUT781	CUTs	CUTs	3.122088461	0.135044074	-1.28195049	-0.214078558	-0.770690536
649421	649813	13	-	CUT782	CUTs	CUTs	3.840202704	0.416435897	-0.072816567	0.002018762	-1.229484806
652357	653605	13	-	YMR194C-B,	other	other	-1.773243268	-1.079488699	1.649420722	0.910074499	-0.890820545
653181	653605	13	-	unknown	no_ovlp2	SUTs	-0.481837808	-0.224542542	-0.380067537	-0.25706239	-0.402549946
655117	656005	13	-	CUT783	CUTs	CUTs	2.329358057	-0.410223616	-1.18390112	-0.870312078	-1.730400898
658389	659229	13	-	VTI1	ORF-T	ORF-T	-0.046484973	-0.609123719	0.33050088	0.114921833	-0.19602437
661093	661613	13	-	CUT785	CUTs	CUTs	2.729238803	0.142836093	-1.034028218	-0.207874632	-0.04273576
665669	667397	13	-	RAD14	ORF-T	ORF-T	-0.306369851	-0.517239601	0.224452377	-0.091522721	0.267410654
667141	667397	13	-	unknown	no_ovlp2	SUTs	0.173897319	0.102272347	0.223130301	-0.389310937	0.144119139
667397	668237	13	-	CUT786	CUTs	CUTs	0.817501231	0.084630972	-0.419709579	-0.241985881	-0.07394048
669725	671517	13	-	INP1	ORF-T	ORF-T	-0.585936189	-0.409751932	0.522991735	-0.024688339	0.457266624
671701	674845	13	-	PFK2	ORF-T	ORF-T	-0.246631682	-0.295611569	-0.276051801	-0.023947604	-0.363858037
677109	683989	13	-	HFA1	ORF-T	ORF-T	0.011227724	-0.098797342	-0.365961594	-0.138402698	0.358506226
685805	687301	13	-	YMR209C	ORF-T	ORF-T	-0.261682171	-0.622570382	-0.163282651	-0.149526491	0.243107115
687709	688845	13	-	SUT736	SUTs	SUTs	0.978827846	-0.343358735	-0.696451612	-0.330936734	-0.140648226
688845	690437	13	-	unknown	no_ovlp	SUTs	0.235862484	0.34355175	-0.082504427	-0.246422875	0.075651647
690597	693173	13	-	EFR3	ORF-T	ORF-T	0.09994851	-0.485174982	-0.412835105	-0.130716608	-0.064006692
698709	701213	13	-	SKY1	ORF-T	ORF-T	0.225501962	-0.450783984	-0.288330582	-0.144621934	-0.501342574
703461	706917	13	-	TRS130	ORF-T	ORF-T	-0.314590745	-0.806267051	0.021233104	0.012607771	0.207870434
710341	711949	13	-	unknown	no_ovlp	CUTs	2.191976583	0.286995397	-0.800956133	0.170221678	0.139551187
713909	715405	13	-	FMP42	ORF-T	ORF-T	-0.137186731	-0.430998262	-0.464379959	-0.309224809	-0.032048678
715573	716333	13	-	FSH2	ORF-T	ORF-T	0.097844772	-0.097253112	0.094970758	-0.145507863	0.088949858
718373	720685	13	-	MRE11	ORF-T	ORF-T	-0.105734193	-0.314380066	-0.230032775	-0.336859957	-0.535768928
720781	721189	13	-	MRPL44	ORF-T	ORF-T	-0.112165726	-0.350660384	-0.03031157	-0.286005676	-0.057648929
721533	722445	13	-	TMA29	ORF-T	ORF-T	0.319443644	-0.02786585	-0.074480897	0.110684027	0.10022246
722445	724421	13	-	TAF7	ORF-T	ORF-T	-0.031444084	-0.005565317	-0.163152434	-0.132339058	-0.109614434
725837	731221	13	-	RRP5	ORF-T	ORF-T	0.070723298	-0.121950204	-0.639831271	0.036693931	-0.01109279
731445	732077	13	-	SUT737	SUTs	SUTs	0.39105492	-0.97862302	-0.677256397	-0.569880631	-1.632506915
741333	742773	13	-	RNA1	ORF-T	ORF-T	-0.125754574	-0.438001091	-0.379266375	0.000862931	-0.268959127
745325	746029	13	-	unknown	no_ovlp	CUTs	1.906177846	0.246605849	-0.463595205	0.205181283	0.802599091
748181	749757	13	-	RNT1	ORF-T	ORF-T	0.578461148	-0.158675745	-0.433795658	-0.099652028	0.420095938
749893	751285	13	-	CUS1	ORF-T	ORF-T	-0.13248599	-0.480335045	-0.011648367	-0.366153929	0.410911144
753741	754333	13	-	RPL20A	ORF-T	ORF-T	0.500324913	-0.6465651	-0.936644435	-0.824051294	-0.103541077
754669	756301	13	-	ZRC1	ORF-T	ORF-T	-0.386631295	-0.691000222	-0.652189231	-0.330213718	0.347075833
758373	758989	13	-	YMR244C-A	ORF-T	ORF-T	0.062912871	-0.179619563	0.344962418	0.041675042	-0.307178787
759909	760141	13	-	CUT788	CUTs	CUTs	2.227108993	-0.215161272	0.340049658	-0.469032999	-0.271693864
760141	761781	13	-	unknown	no_ovlp	SUTs	-0.135612924	0.376640333	0.331875236	0.062845618	0.126127452
761957	763109	13	-	SNR86	other	other	-0.394720107	-0.317002933	-0.114780192	-0.197307583	-0.192516345
763341	768061	13	-	RKR1	ORF-T	ORF-T	-0.152225771	-0.298001364	-0.192943695	0.196467668	0.04441198
768877	770493	13	-	unknown	no_ovlp	SUTs	0.163227768	-0.774407685	0.350687762	0.522825504	0.123923792
773341	774061	13	-	CUT790	CUTs	CUTs	0.777590823	-0.942997647	-0.142050631	0.420486192	0.028365247
775253	777237	13	-	YMR252C, YI	ORF-T	ORF-T	-0.037968449	-0.609331209	-0.15302798	0.252338102	-0.232714485
778709	779165	13	-	COX7	ORF-T	ORF-T	-0.028244094	-0.60373022	0.589212279	0.167656343	0.178573792
781117	782413	13	-	PET111	ORF-T	ORF-T	-0.177680419	-0.9592824	-0.445401785	-0.357176553	-0.618179294
782597	784325	13	-	YMR258C	ORF-T	ORF-T	0.419183081	-0.373005748	0.059036138	0.185969223	-0.00212726
784549	788933	13	-	YMR259C	ORF-T	ORF-T	0.433744891	-0.366046067	-0.362330727	-0.004370492	0.322310682
788933	789189	13	-	unknown	no_ovlp	SUTs	-0.56741563	-0.075833383	-0.054813954	0.145039064	0.188464349
789285	789869	13	-	TIF11	ORF-T	ORF-T	0.014239126	-0.432363531	-0.152191267	-0.021652695	-0.33605321
790069	793501	13	-	TPS3	ORF-T	ORF-T	-0.449893435	-0.715354176	0.021330541	0.338687791	-0.228944535
796541	797901	13	-	YMR265C	ORF-T	ORF-T	0.003138261	-0.614186915	0.013807415	0.314427937	0.156066089
802757	804301	13	-	PRP24	ORF-T	ORF-T	0.395208041	-0.004252259	-0.090649234	-0.052515405	0.329947301
805245	806533	13	-	RRN9	ORF-T	ORF-T	0.172821299	-0.721920685	-0.485430277	-0.643417933	-0.14925877
807757	808117	13	-	URA10	ORF-T	ORF-T	0.083665161	-1.35760073	0.771650292	0.237742861	0.735699008
809453	810821	13	-	SCS7	ORF-T	ORF-T	-0.055451268	-0.703646987	-0.637595657	0.008118931	-0.525080195
811165	814165	13	-	ZDS1	ORF-T	ORF-T	0.139045877	-0.249348353	-0.621607678	-0.410614265	-0.35311921
814333	815357	13	-	RCE1	ORF-T	ORF-T	-0.5058326	-0.743224388	-0.341152359	-0.181277211	-0.208787583
815557	818621	13	-	BUL1	ORF-T	ORF-T	-0.176578364	-0.231533699	-0.099303425	-0.042453578	0.324756059
819109	819877	13	-	unknown	no_ovlp	SUTs	0.168120164	0.288379568	-0.47849235	-0.025889141	0.47160802
827053	832317	13	-	CAT8	ORF-T	ORF-T	-1.130134939	0.135180727	2.47012673	2.046982854	-0.616685087
831437	832317	13	-	unknown	no_ovlp2	SUTs	0.14030245	0.445750487	0.220662942	-0.089760482	0.332913887
832317	832949	13	-	unknown	no_ovlp	CUTs	1.105959296	0.719733852	0.727551075	0.558364344	0.105973068
833245	836853	13	-	AEP2	ORF-T	ORF-T	-0.461087558	-0.795498509	-0.200253317	-0.21964758	-0.099237336
835149	836853	13	-	unknown	no_ovlp2	SUTs	0.259153916	-0.150813248	-0.037755188	-0.11873209	-0.032907556
839997	841693	13	-	NGL2	ORF-T	ORF-T	0.032344743	-0.373062747	-0.347380605	0.116654805	-0.25652311
842333	845389	13	-	DSS1	ORF-T	ORF-T	-0.425636676	-0.557493802	-0.295599867	-0.182229158	-0.202938526
849853	851645	13	-	HAS1	ORF-T	ORF-T	-0.048775353	-0.38260647	-0.737152109	-0.113279167	0.118849785
854437	854933	13	-	SUT739	SUTs	SUTs	1.618011069	0.507794799	0.014087895	-0.786940625	-0.882431972
855341	856821	13	-	YMR293C	ORF-T	ORF-T	-0.002555869	-0.266479807	0.283735479	-0.13150924	-0.147737469
858149	858949	13	-	YMR295C	ORF-T	ORF-T	-0.108466406	-0.335670002	-0.033174154	-0.033575639	-0.000597439
859141	861061	13	-	LCB1	ORF-T	ORF-T	-0.330663515	-0.169680392	-0.600366419	-0.394549222	-0.467878137
864301	865365	13	-	DYN3	ORF-T	ORF-T	0.331948633	-0.193833703	-0.122260341	-0.474735941	0.439324806
865477	867221	13	-	ADE4	ORF-T	ORF-T	0.474809093	-0.232152003	-1.157402987	-0.064353045	-0.672795974
867509	869661	13	-	ATM1	ORF-T	ORF-T	-0.25805891	-0.001748264	0.19809256	-0.048388766	0.463454449
870013	872669	13	-	YME2	ORF-T	ORF-T	-0.077513675	-0.341510597	0.334563592	0.179609925	-0.005106411
873357	874173	13	-	ADH2	ORF-T	ORF-T	-0.020101774	-0.059431923	1.599002533	0.813508777	1.767411876
874197	874549	13	-	CUT791	CUTs	CUTs	2.476605557	0.010148145	0.693414358	0.639935547	0.987901046
878885	880397	13	-	YMR304C-A,	ORF-T	ORF-T	-0.617164902	-0.524205102	0.123994882	0.151632365	0.321785123
884181	886445	13	-	unknown	no_ovlp	CUTs	2.101543086	-0.374072387	-1.041726105	-0.695598901	-0.110554845
888821	892397	13	-	PSE1	ORF-T	ORF-T	-0.017928597	-0.561290769	-0.743647887	-0.075169406	-0.406225339
892709	895485	13	-	NIP1	ORF-T	ORF-T	-0.002941836	-0.631091321	-0.76733903	-0.121840442	-0.389946196

895677	896733	13	-	YMR310C	ORF-T	ORF-T	0.375490524	-0.138127812	-0.309570688	-0.215265397	0.338155326
896805	897645	13	-	GLC8	ORF-T	ORF-T	-0.071644717	-0.459027383	0.385929179	0.404706716	0.01115772
899325	901333	13	-	TGL3	ORF-T	ORF-T	-0.022045413	-0.516528609	-0.194204717	-0.135920389	-0.259804434
902085	904509	13	-	unknown	no_ovlp	CUTs	1.1161666	-0.168438083	-0.347063168	0.25486805	-0.59392367
907229	910741	13	-	YMR316C-B	other	other	0.513785639	-0.586129532	-0.43517351	-0.002980591	-0.285800376
910901	912277	13	-	ADH6	ORF-T	ORF-T	-0.411055145	-0.799853865	-0.395535613	-0.075422238	-0.093348065
912701	915077	13	-	FET4	ORF-T	ORF-T	0.080967733	-0.555023982	-0.891895484	-0.446012087	0.007938291
914717	915077	13	-	unknown	no_ovlp2	CUTs	2.755656465	0.031853635	-0.006880193	-0.434078628	-0.732862059
10153	11449	13	+	YML131W	ORF-T	ORF-T	0.343487893	0.53070757	1.142832545	0.673913326	-0.959498596
13497	14081	13	+	SUT285	SUTs	SUTs	1.438457784	-0.559525111	0.249016218	0.237063647	-0.300612451
15041	15505	13	+	CUT280	CUTs	CUTs	2.565945717	0.049085044	-0.772048976	0.099408546	-0.821907885
17057	18817	13	+	RSC9	ORF-T	ORF-T	-0.323766623	-0.790999326	-0.315177503	-0.022081935	0.064875658
20777	21881	13	+	unknown	no_ovlp	SUTs	0.461557316	-1.219369076	-1.480627543	0.35881283	0.135851193
23897	25689	13	+	CUT281	CUTs	CUTs	0.8785894	-1.076472863	-0.643963633	-1.202372662	-1.435336535
26881	28025	13	+	GTR1	ORF-T	ORF-T	0.243756429	-0.098973759	0.231025608	-0.525824835	0.951035858
30609	31745	13	+	YML119W	ORF-T	ORF-T	0.462959261	0.441879866	0.216226276	-0.198872732	0.384877889
32305	34113	13	+	NGL3	ORF-T	ORF-T	0.437490619	0.279056539	0.680091759	0.42497628	-1.948698376
34113	37841	13	+	NAB6, YML1	ORF-T	ORF-T	-0.402797869	-0.507022819	-0.02837415	0.176154949	-0.134521938
38129	39977	13	+	ATR1	ORF-T	ORF-T	0.243207047	-0.66917609	-0.779819244	-0.155936356	-0.401570663
44009	44929	13	+	DAT1	ORF-T	ORF-T	0.323442516	0.349442188	0.14607061	0.07809382	0.592320514
45049	45601	13	+	CTK3	ORF-T	ORF-T	0.506703342	-0.303934984	0.138094134	-0.313569263	0.125066389
46833	49761	13	+	BUL2	ORF-T	ORF-T	0.065355145	-0.357172117	-0.647160563	-0.338433487	-0.286189611
51609	54545	13	+	ZDS2	ORF-T	ORF-T	-0.030511025	0.18473133	0.386192412	-0.021018777	1.064319539
54737	55329	13	+	YML108W	ORF-T	ORF-T	0.140230237	-0.820932056	-0.44335797	-0.451263151	0.452621463
56529	57601	13	+	URA5	ORF-T	ORF-T	-0.29392708	-0.395913047	-0.269648058	-0.100448919	0.108891831
58873	59369	13	+	CUT283	CUTs	CUTs	2.171104362	-0.209021607	-0.607520814	-0.193252206	0.144361365
67465	68305	13	+	unknown	no_ovlp	SUTs	-0.627632768	0.199824519	0.173379906	0.215551695	0.195454257
68305	69753	13	+	CAC2	ORF-T	ORF-T	-0.392537304	-0.003876681	0.244419545	0.276540883	0.854667823
70353	70593	13	+	YML100W-A	ORF-T	ORF-T	-0.348284024	0.361375766	0.129197419	-0.170701351	0.321510407
70593	71297	13	+	TSL1	ORF-T	ORF-T	-0.368229692	-1.040551778	0.507607209	0.660817169	0.219770024
77249	77929	13	+	TAF13	ORF-T	ORF-T	-0.059682194	-0.69317308	-0.399404094	-0.19017046	-0.171637938
79897	81601	13	+	YML096W	ORF-T	ORF-T	-0.102949343	-0.391823573	-0.376631338	-0.190667074	0.384181786
82385	82937	13	+	GIM5	ORF-T	ORF-T	0.977163914	0.346202567	-0.015300101	-0.481398195	0.024119999
83089	85889	13	+	UTP14	ORF-T	ORF-T	0.477639367	-0.241979168	-0.594299621	0.007427773	0.159999512
92201	94329	13	+	UFO1	ORF-T	ORF-T	-0.718634166	-0.114223579	0.042539272	-0.000267578	0.915171726
97713	97945	13	+	SUT287	SUTs	SUTs	-0.386068632	-1.421905617	1.684540494	1.398627246	0.692345424
99713	101121	13	+	unknown	no_ovlp	SUTs	0.690992252	0.133780642	0.301874082	0.237733414	-0.60296143
101825	103985	13	+	YML082W	ORF-T	ORF-T	0.457270631	0.058837312	-0.648203235	-0.282694346	-0.171148177
104641	108593	13	+	YML081W	ORF-T	ORF-T	0.080002427	-0.328298069	-0.553596839	-0.151243447	0.059081457
108697	110081	13	+	DUS1	ORF-T	ORF-T	0.319925965	-0.082849816	-0.356756113	-0.262555889	0.904905231
110249	110993	13	+	YML079W, C	other	other	-0.183911725	-0.221210213	-0.361529381	-0.326613479	-0.691559874
111881	112401	13	+	BET5	ORF-T	ORF-T	0.417859829	-0.324753463	-0.821191747	-0.368436357	-0.39578213
115689	116833	13	+	unknown	no_ovlp	CUTs	1.11935657	0.2220424	-1.1190277	-0.023135826	0.756789934
119545	120257	13	+	SUT288	SUTs	SUTs	0.678716314	-0.763340415	0.597950556	0.075751673	0.607547054
121561	123001	13	+	SUT289	SUTs	SUTs	0.870812998	0.078740787	-0.758684044	0.155099615	-0.794763386
129785	130377	13	+	CUT285	CUTs	CUTs	2.67333549	-0.601118694	-1.05304158	-0.149309137	0.192708965
133473	135297	13	+	DAK1	ORF-T	ORF-T	0.125754966	0.182350294	0.290450473	0.39813651	-0.33012557
135465	137233	13	+	POB3	ORF-T	ORF-T	-0.259940038	-0.395060352	-0.236290599	-0.214283196	-0.072213469
137489	139105	13	+	ITT1	ORF-T	ORF-T	0.015795659	-0.469590762	-0.069465413	-0.473345349	0.087290665
142129	145041	13	+	ORC1	ORF-T	ORF-T	-0.220567514	-0.503698048	-0.475196388	-0.331298825	0.523930941
146433	147361	13	+	RPS1B	ORF-T	ORF-T	-0.418017343	-0.437244582	-0.31917229	-0.214846501	0.044751698
151913	153145	13	+	OGG1	ORF-T	ORF-T	-0.248075723	0.113823977	-0.391372629	0.055516572	1.169278216
158681	159329	13	+	unknown	no_ovlp	SUTs	0.080636358	1.588514592	2.517069182	2.483011219	-1.34497116
159329	159977	13	+	SML1	ORF-T	ORF-T	0.181595454	-0.016283247	0.116639359	-0.048371862	0.370087287
160129	160593	13	+	CMP2	ORF-T	ORF-T	-0.150059295	-0.198994306	0.064377874	-0.049448209	-0.431456928
164777	165361	13	+	SPC2	ORF-T	ORF-T	0.468952622	-0.312600781	-0.0088104	-0.485166229	-0.03022585
167697	168353	13	+	SUT295	SUTs	SUTs	0.502938698	-0.21533708	0.444880854	0.443066376	-0.730646262
170377	171417	13	+	SUR7	ORF-T	ORF-T	-0.29696956	-0.530899047	-0.224238152	0.146825456	0.20333052
171529	173009	13	+	GAL80	ORF-T	ORF-T	0.012388274	-0.260572316	-0.12368977	-0.384188094	-0.199062709
173009	174209	13	+	YML050W	ORF-T	ORF-T	-0.045009043	-0.07010628	0.30487361	-0.390926884	0.178438386
178393	179977	13	+	GSF2	ORF-T	ORF-T	-0.137682486	-0.146085227	-0.231949185	0.058394522	0.14795831
181433	183593	13	+	PRP39	ORF-T	ORF-T	0.486360275	-0.34702943	-0.698849615	-0.588116619	-0.73322351
192713	193777	13	+	CAT2	ORF-T	ORF-T	0.65968055	1.102008201	3.083377975	1.784602714	2.868660643
193777	194961	13	+	CAT2	ORF-T	ORF-T	0.079754689	0.537851219	2.166632976	1.331990519	1.839918116
196273	202649	13	+	unknown	no_ovlp	SUTs	0.575543213	0.258696011	-0.636969286	0.093610844	-0.608684244
204449	205001	13	+	CUT288	CUTs	CUTs	3.033761234	-0.067217928	-1.020728705	-0.175767758	0.52073731
205617	206097	13	+	CGI121	ORF-T	ORF-T	0.315567859	-0.228644377	-0.322080847	-0.497829914	-0.395831247
206217	206697	13	+	unknown	no_ovlp	CUTs	1.306280193	-0.414587988	-0.68992595	-0.426058138	-0.513395625
209449	212321	13	+	SRC1	ORF-T	ORF-T	-0.118336178	-0.362999976	0.081177733	0.027981664	0.571505499
214169	216233	13	+	NDC1	ORF-T	ORF-T	-0.348520402	-0.160628538	-0.193528157	0.071091739	0.314264861
216417	217153	13	+	YML030W	ORF-T	ORF-T	-0.422913173	-0.999720465	0.028638688	0.079890877	-0.124892849
217297	220105	13	+	USA1	ORF-T	ORF-T	-0.046862513	-0.23835996	-0.071836732	-0.116346081	0.039177442
220105	220841	13	+	TSA1	ORF-T	ORF-T	-0.661217202	-0.33961609	0.022206377	-0.286036567	0.30594187
221337	222649	13	+	YOX1	ORF-T	ORF-T	-0.135871423	-0.011451432	-0.443677078	-0.21868986	0.577508443
225889	226313	13	+	RPS17A	ORF-T	ORF-T	0.724542031	-1.019560873	-1.129345603	-0.896556729	-0.443510004
228889	229705	13	+	APT1	ORF-T	ORF-T	-0.254514171	-0.589531355	-0.699530073	-0.184285416	0.144470492
229825	230985	13	+	unknown	no_ovlp	SUTs	0.446392158	0.471313757	-0.080348846	-0.092924987	-0.108333257
230985	233313	13	+	YML020W	ORF-T	ORF-T	0.250765907	0.147572332	0.035352866	-0.105702625	0.10855429
233457	234729	13	+	OST6	ORF-T	ORF-T	-0.313445596	-0.741914765	-0.486304318	-0.137228539	0.130165796
236617	236969	13	+	PSP2	ORF-T	ORF-T	-0.2616631	-1.099200389	-0.345352943	-0.010046778	0.079696133
242017	242841	13	+	unknown	no_ovlp	CUTs	2.072283219	-0.668779735	-0.695719795	-0.18933154	0.671537957
243225	244065	13	+	unknown	no_ovlp2	SUTs	0.378912146	0.311772287	-0.023222762	0.105620715	0.453173445

244065	246081	13	+	UBX2	ORF-T	ORF-T	0.253244109	-0.035141275	-0.106639377	-0.181754714	-0.182461152
246081	246953	13	+	ERV25	ORF-T	ORF-T	-0.493123982	-0.559702791	-0.29868939	-0.184971581	0.294354924
247665	251097	13	+	SPT5, YML00	ORF-T	ORF-T	-0.155469565	-0.268993096	-0.494601173	0.10322632	-0.21480979
253241	253729	13	+	SUT296	SUTs	SUTs	1.030494166	0.574035617	-0.818382014	-0.286342201	-0.022426376
253729	256025	13	+	YAP1	ORF-T	ORF-T	0.111095251	-0.133963534	0.254351422	-0.189706976	0.045909539
260145	261689	13	+	TRM12	ORF-T	ORF-T	0.875756257	0.149149775	-0.13413702	-0.154516456	-0.3881216
262793	263497	13	+	unknown	no_ovlp	SUTs	-0.431441444	-0.299786425	1.306248206	0.045276133	-0.410285602
263497	266817	13	+	YML003W, YI	ORF-T	ORF-T	0.710654969	-0.054491982	0.144176831	-0.050297777	-1.20481806
267089	267913	13	+	YPT7	ORF-T	ORF-T	-0.411995114	-0.350257694	0.071584059	-0.032138196	0.101237661
272113	272913	13	+	MIC17	ORF-T	ORF-T	0.312419397	-0.124392981	0.05884064	0.056601314	0.447264001
273113	273737	13	+	YMR003W	ORF-T	ORF-T	-0.432521157	0.056075099	0.117011172	-0.390024486	1.186673278
273977	275705	13	+	MVP1	ORF-T	ORF-T	0.106230426	-0.170237147	0.291001137	0.101663829	0.256480874
275993	276769	13	+	TAF4	ORF-T	ORF-T	-0.416311373	-0.417594677	-0.064838308	0.046379859	0.29083755
283249	283609	13	+	ADI1	ORF-T	ORF-T	0.414501656	-0.566289063	-0.092434337	0.102363603	-0.587859741
283609	284881	13	+	unknown	no_ovlp2	CUTs	1.427650633	-0.000927417	-0.322509847	0.13433053	-0.38502175
284881	286337	13	+	YMR010W	ORF-T	ORF-T	-0.432955332	-0.241772614	-0.477545903	-0.268912192	-0.057019723
288065	289817	13	+	HXT2	ORF-T	ORF-T	-1.424944665	-0.927297206	1.591537172	1.648264458	1.041336598
291017	295129	13	+	CLU1	ORF-T	ORF-T	-0.481649075	-0.490450291	-0.442924532	-0.120172709	-0.257996603
298865	300529	13	+	BUD22	ORF-T	ORF-T	0.421699077	-0.224249811	-0.697529838	-0.259826593	0.123377567
302777	307345	13	+	unknown	no_ovlp	SUTs	0.887230114	0.45067047	-0.485891268	-0.261355688	-0.109114942
312273	314985	13	+	STB4	ORF-T	ORF-T	0.093359433	-0.520920539	-0.219301734	-0.132588124	-0.02938784
315361	316993	13	+	FMS1	ORF-T	ORF-T	-0.436043633	-0.251185041	0.230598992	0.73973528	-0.74750634
318657	319321	13	+	UBC7	ORF-T	ORF-T	-0.200035553	-0.494875244	-0.240171103	-0.127979532	-0.310426002
321905	322273	13	+	MRPL3	ORF-T	ORF-T	-0.649073188	-0.889443322	-0.201438815	-0.049112168	-0.248058779
323225	324233	13	+	CSI1	ORF-T	ORF-T	0.682160977	0.657853534	0.767996755	-0.158139064	-0.75513337
325809	327361	13	+	YMR027W	ORF-T	ORF-T	-0.287576837	-0.335038544	-0.028762624	-0.04342047	-0.018563376
327481	328665	13	+	TAP42	ORF-T	ORF-T	-0.005330796	-0.860608625	-0.092016635	-0.277715277	-0.333137203
330409	330633	13	+	CUT293	CUTs	CUTs	1.62414908	0.203411151	0.115551508	-0.288141827	-0.089409928
330633	332009	13	+	RSF1	ORF-T	ORF-T	0.03395016	-0.132383423	0.446853952	0.281012806	-0.205786492
335209	337441	13	+	HOF1, YMR0	other	other	-0.065415526	-0.050810352	-0.172130223	0.125183277	0.903908376
337913	339353	13	+	ARP9	ORF-T	ORF-T	-0.098726658	-0.721339139	-0.742357053	5.22E-05	-0.361653992
341113	341833	13	+	IMP2	ORF-T	ORF-T	-0.325673415	0.22925599	0.692767008	-0.308898322	1.393407473
343761	344289	13	+	SUT299	SUTs	SUTs	1.365701931	-0.148276899	0.628915627	0.088145255	0.434962461
347545	349049	13	+	CUT294	CUTs	CUTs	1.681258917	-1.006670844	-0.735644167	-0.284961793	-0.304217896
349993	350929	13	+	YET2	ORF-T	ORF-T	-0.050272048	-0.674548274	0.308184991	0.358472165	0.81759081
352545	353497	13	+	ARG80	ORF-T	ORF-T	0.213299547	-0.162718856	-0.329082461	0.029601513	-0.367813495
353497	355105	13	+	MCM1	ORF-T	ORF-T	0.114022761	-0.29106603	-0.470548498	-0.276001973	-0.293687216
355361	363001	13	+	IOC4	ORF-T	ORF-T	-0.441506845	-0.726935805	-0.088458538	0.047582864	0.247153743
357013	363001	13	+	unknown	no_ovlp2	CUTs	3.020772881	-0.628331752	-0.568516399	-0.611439849	-0.191819238
366977	368089	13	+	CSM3	ORF-T	ORF-T	-0.258715383	-0.373070341	-0.333405894	-0.294075649	0.143701744
370737	378809	13	+	SUT301	SUTs	SUTs	0.164004927	0.528532542	0.429876181	0.412344858	-0.209740755
378809	379241	13	+	unknown	no_ovlp	CUTs	2.435112233	0.362756646	-0.184017999	0.427507784	0.410025188
379593	380937	13	+	FAR3	ORF-T	ORF-T	0.335404422	-0.68639234	-0.186354957	-0.105417233	-0.167807079
383097	386097	13	+	STV1	ORF-T	ORF-T	-0.455014784	-0.521404281	-0.398512462	0.065889436	0.090966402
388777	390873	13	+	FET3	ORF-T	ORF-T	-0.237937889	-1.494161554	-0.992874653	0.320581492	-0.2604127
391073	391561	13	+	SEN15	ORF-T	ORF-T	0.707732695	-0.572938449	-0.307123314	-0.191853014	0.126103686
392753	393977	13	+	RNA14	ORF-T	ORF-T	0.017420156	-0.644751866	-0.571852463	-0.061833697	-0.115340572
397041	397945	13	+	RIM9, AEP1	ORF-T	ORF-T	0.011842615	-0.283440159	0.023387778	-0.212191889	0.054274351
399577	399865	13	+	unknown	no_ovlp2	SUTs	-0.593788861	-0.495753921	0.417171877	-0.03115952	0.308171422
399865	401297	13	+	KAR5	ORF-T	ORF-T	0.546964535	-0.286109952	-0.667519601	-0.422281245	-0.166936738
401553	404281	13	+	SOV1	ORF-T	ORF-T	0.238462344	-0.753724095	-0.30510476	-0.269578176	-0.407415281
405745	406281	13	+	unknown	no_ovlp2	CUTs	1.204371683	0.040732161	0.371226968	-0.543975409	-0.257359248
406281	408985	13	+	AVO2	ORF-T	ORF-T	-0.476236113	-0.362321908	0.755126669	-0.115514063	1.104176803
407585	408985	13	+	unknown	no_ovlp2	SUTs	0.671200146	-0.156864313	-0.416636018	-0.174787065	0.081680828
408985	410689	13	+	MOT3	ORF-T	ORF-T	-0.286386412	-0.713544102	-0.270136676	0.148711796	-0.087226839
411545	412257	13	+	ABF2	ORF-T	ORF-T	-0.219740059	-0.807486557	0.282241298	-0.175466378	-0.035409635
413121	416137	13	+	RCO1	ORF-T	ORF-T	0.233017062	-0.476334408	-0.232731628	-0.098204539	-0.152479186
422377	422865	13	+	unknown	no_ovlp	CUTs	1.280861553	-0.144799975	0.286033359	0.828580298	-0.599193333
425169	426385	13	+	SEC14	ORF-T	ORF-T	0.08763119	-0.195546583	-0.417429039	-0.108735248	-0.379734418
429953	431153	13	+	SUT302	SUTs	SUTs	0.948784596	-0.341398648	-1.106060483	-1.32452374	-0.64431379
434153	434737	13	+	ADH3	ORF-T	ORF-T	2.025437512	0.896323201	-0.611107146	-0.611481371	-1.26155928
436009	436329	13	+	unknown	no_ovlp2	CUTs	1.456445807	0.471159774	-0.717318049	-0.491174247	-0.821364223
436329	439033	13	+	YMR084W, Y	ORF-T	ORF-T	-0.226223264	-0.361730926	0.910517297	1.191251864	0.640598925
438777	439033	13	+	unknown	no_ovlp2	SUTs	-0.157270583	0.134833608	0.216831239	0.306184328	0.420462845
439153	441785	13	+	YMR086W	ORF-T	ORF-T	-0.559440241	-0.322328059	0.062722862	0.292743652	-0.073016938
441785	442097	13	+	unknown	no_ovlp2	SUTs	-0.838950255	-0.60951905	-0.204944679	0.236811014	-0.157971686
442097	443409	13	+	YMR087W	ORF-T	ORF-T	-0.027577421	-1.001714175	-0.350241869	-0.234877119	-0.00764791
448473	449241	13	+	YMR090W	ORF-T	ORF-T	0.335819825	-0.304740009	-0.464384918	-0.600303627	-0.658501562
453977	455673	13	+	UTP15	ORF-T	ORF-T	0.339069245	-0.014027396	-0.63964192	-0.084863682	0.533846483
455809	457569	13	+	CTF13, SNZ1	ORF-T	ORF-T	-0.08641349	-0.214492031	-0.273030866	-0.609354134	0.533021559
465017	465385	13	+	SUT304	SUTs	SUTs	0.009241938	-0.590397861	0.409068854	-0.046735773	0.168405089
465969	468361	13	+	MUB1	ORF-T	ORF-T	0.112378749	-0.552000107	-0.269165624	-0.013763162	-0.343430177
472929	474105	13	+	unknown	no_ovlp	CUTs	1.966919051	-0.135389662	-0.537351602	-0.054359714	0.343934869
475913	476289	13	+	unknown	no_ovlp	CUTs	3.714811985	0.357372442	0.573962849	-0.197917411	-0.7642597
478001	479529	13	+	unknown	no_ovlp	CUTs	1.441516758	0.21025153	0.308175035	0.499356653	0.740173829
480881	481217	13	+	SUT305	SUTs	SUTs	0.094616221	0.490115485	1.879802636	1.302832353	-2.415276254
482529	482953	13	+	unknown	no_ovlp2	SUTs	0.286774907	0.500260886	1.255032672	0.884428512	-0.438949799
482953	483865	13	+	SPG4	ORF-T	ORF-T	-1.687272109	-0.599226855	4.810443247	3.630543527	0.512124312
483553	483865	13	+	unknown	no_ovlp2	SUTs	-0.254050299	0.533359937	0.922521986	1.203762629	-0.560446645
483865	486337	13	+	ILV2	ORF-T	ORF-T	-0.207197857	-0.613309194	0.065056636	0.02214863	-0.336052554
486561	490337	13	+	MYO5	ORF-T	ORF-T	-0.186651223	-0.286622325	-0.605067087	-0.207448089	-0.300479442
493481	494953	13	+	CUT299	CUTs	CUTs	1.002686142	0.192023936	0.330454456	0.168997401	0.160239173

494953	496329	13	+	FOL3	ORF-T	ORF-T	0.206982643	-0.508006945	-0.375172511	-0.215690431	-0.657395226
497665	499305	13	+	FMP24	ORF-T	ORF-T	0.191925241	-0.028881342	0.592715694	0.227798652	0.191845569
501097	501825	13	+	CUT300	CUTs	CUTs	3.05924279	0.291618609	-1.293783121	-0.611449251	0.78373337
502137	503361	13	+	YMRWdelta1	other	other	0.253090922	-0.279498353	0.418204619	-0.407641471	0.878799499
505161	507353	13	+	ASI1, YMR11	ORF-T	ORF-T	-0.20617436	-0.471719279	-0.121004626	-0.068055731	-0.318032929
509713	511281	13	+	SUT306	SUTs	SUTs	0.040014551	0.038378918	-0.647371825	-0.260432766	-0.007166213
511281	511809	13	+	YMR122W-A	ORF-T	ORF-T	-0.253214098	0.117057296	0.188739951	-0.101272835	0.043070695
513545	514033	13	+	PKR1	ORF-T	ORF-T	0.466946817	-0.113109222	-0.502529124	-0.699719332	-0.120060947
514465	516705	13	+	YMR124W	ORF-T	ORF-T	-0.028724819	-0.260216142	0.07262803	-0.130631589	-0.103205468
517369	517896	13	+	STO1	ORF-T	ORF-T	0.963731031	-0.083478466	-0.511591617	-0.719449463	-0.099821805
522217	522865	13	+	SUT308	SUTs	SUTs	1.204796638	0.011912912	0.444054664	0.009809189	-0.026294314
523665	527577	13	+	ECM16	ORF-T	ORF-T	0.35013021	-0.070305211	-0.630579421	-0.063222031	0.266976933
527769	531921	13	+	POM152	ORF-T	ORF-T	-0.132958625	-0.29054372	-0.551705204	0.126531997	0.023235429
532057	533097	13	+	YMR130W	ORF-T	ORF-T	-0.324001999	-0.701868506	-0.294980735	-0.305497149	-0.127427996
534953	535609	13	+	CUT301	CUTs	CUTs	3.092046531	-1.33618383	-1.713030241	-0.307703554	-0.102065062
537793	538817	13	+	ERG29	ORF-T	ORF-T	0.435312531	-0.736411196	-0.291074153	0.246495232	0.40213233
540593	541121	13	+	unknown	no_ovlp	SUTs	0.06076226	0.351300764	-0.043966634	-0.160127076	-0.184366559
541121	542977	13	+	GAT2	ORF-T	ORF-T	-0.428344698	-1.06058719	-0.21777784	0.100508356	0.257104635
545169	545937	13	+	CIN4	ORF-T	ORF-T	0.174530221	0.102208716	0.188958911	-0.675607613	0.244698137
545937	547377	13	+	RIM11	ORF-T	ORF-T	-0.530740074	-0.67694284	0.358868914	0.147180837	-0.076123834
547673	550001	13	+	SIP5	ORF-T	ORF-T	0.030768043	-0.552789113	0.326658942	-0.074644104	-0.205242584
551953	552529	13	+	RPS16A	ORF-T	ORF-T	0.471700117	-1.055274083	-1.224091431	-0.817752313	-0.342808813
553257	554529	13	+	YMR144W	ORF-T	ORF-T	-0.598461653	-0.383964167	0.431733463	-0.22809696	1.16941311
556809	557489	13	+	SUT309	SUTs	SUTs	0.289926778	0.463706794	0.747066227	-0.473550804	0.264106388
557489	557833	13	+	CUT303	CUTs	CUTs	3.511444053	0.967641194	-0.065028676	-0.185774901	0.849752661
559177	559777	13	+	YMR147W	ORF-T	ORF-T	2.176644768	1.785664296	0.169493965	0.153761707	0.96734222
560121	560929	13	+	YMR148W	ORF-T	ORF-T	-0.141250018	-0.152943918	0.650095843	0.239041634	1.129648134
560929	561977	13	+	SWP1	ORF-T	ORF-T	-0.24878163	-0.379982109	-0.370107991	0.027059984	-0.039232952
563097	564217	13	+	YIM1	ORF-T	ORF-T	0.405570236	-0.298959436	0.483420932	0.156001917	-0.193951926
564401	566057	13	+	NUP53	ORF-T	ORF-T	-0.3076826	-0.369855696	-0.055169103	0.028632549	0.324167929
568593	570329	13	+	YMR155W	ORF-T	ORF-T	0.302038999	-0.04085872	0.047639198	-0.003385972	-0.157870038
572217	572801	13	+	MRPS8	ORF-T	ORF-T	-0.559793606	-0.8324947	-0.602148619	-0.518411264	-0.174946789
575033	577681	13	+	YMR160W	ORF-T	ORF-T	-0.344068524	-0.349393331	0.420886131	-0.236794774	0.238833092
577681	578793	13	+	HLJ1	ORF-T	ORF-T	-0.081228626	-0.961045935	-0.575217878	-0.051162138	-0.406455836
584377	585489	13	+	CUT304	CUTs	CUTs	2.27742725	0.363190928	-1.016735599	0.364381788	-0.512248777
590457	590977	13	+	unknown	no_ovlp	CUTs	1.564803905	0.958840852	-0.19996865	0.796074247	0.015780562
594881	597897	13	+	MLH1	ORF-T	ORF-T	0.042803427	-0.510206803	-0.234765673	-0.12289617	-0.147463701
599361	600177	13	+	unknown	no_ovlp	CUTs	1.398187706	-0.277626939	-1.105344258	-0.428312388	0.032941154
606121	607953	13	+	HOT1	ORF-T	ORF-T	-0.087455562	-0.511325569	-0.082378675	-0.445603737	0.248174688
608649	610113	13	+	DDR48, YMR	ORF-T	ORF-T	-0.383793708	-0.752513282	-0.223626188	0.091914727	-0.12385908
611705	616057	13	+	ECM5	ORF-T	ORF-T	0.146697039	-0.096058134	0.130471198	-0.315318406	-0.781945765
616433	618297	13	+	MMT1	ORF-T	ORF-T	0.273263017	-0.163861205	-0.307496788	0.052341615	-0.226904379
618457	619433	13	+	YMR178W	ORF-T	ORF-T	0.03035051	-0.22784997	-0.097077363	0.022360918	-0.408764487
619433	619801	13	+	unknown	no_ovlp2	SUTs	0.589984262	-0.103955683	-0.512778928	-1.150070481	0.060081607
619801	622225	13	+	SPT21	ORF-T	ORF-T	-0.119531018	-0.169491474	-0.379095561	-0.484516466	0.446671651
622697	623465	13	+	unknown	no_ovlp	SUTs	0.829615376	-0.695998394	1.153140607	0.324656749	-0.485121093
623465	626257	13	+	YMR182W-A	ORF-T	ORF-T	1.167196725	-0.656340293	-0.61092516	-0.521334524	-0.948962273
626361	626729	13	+	SNR83	other	other	-0.829202156	-0.470733814	-0.019161818	0.069835763	0.196103101
626913	627345	13	+	unknown	no_ovlp	CUTs	1.638495992	-0.007755595	-0.730052487	-0.092826516	-0.538971892
628145	628881	13	+	ADD37	ORF-T	ORF-T	-0.259469408	0.265752685	0.342811146	-0.526316102	0.62507428
628969	632041	13	+	YMR185W	ORF-T	ORF-T	0.109452071	-0.219311033	0.148762039	-0.10730419	0.955718365
632329	634569	13	+	HSC82	ORF-T	ORF-T	0.00792414	-0.191610945	-0.145211839	-0.097027386	-0.278897442
637401	640697	13	+	GCV2	ORF-T	ORF-T	0.774985638	-0.597582876	-0.894009113	0.04813734	-0.720020424
645657	646937	13	+	SPG5	ORF-T	ORF-T	-0.49441284	0.506992047	1.894172244	0.40383122	2.076213245
647041	647745	13	+	GYL1	ORF-T	ORF-T	0.328923361	0.256529304	-0.419108434	0.095571102	-0.336780018
650033	650945	13	+	MRPL24	ORF-T	ORF-T	-1.153622267	-1.025818186	0.575418748	0.092702015	-0.211517246
651121	651633	13	+	RPL36A	ORF-T	ORF-T	0.885894215	-0.844787851	-1.108328294	-1.122466841	0.004305631
652289	653009	13	+	CUT306	CUTs	CUTs	6.03390546	-0.569561559	-1.138124188	-0.457215195	-0.261840651
653201	653945	13	+	SUT312	SUTs	SUTs	0.874244167	-0.828525899	-0.908315082	-0.71036036	-0.475607933
653945	654545	13	+	ICY1	ORF-T	ORF-T	0.178564099	-0.749297799	-0.243487413	-0.139446115	-0.388104492
656329	658465	13	+	YMR196W	ORF-T	ORF-T	-0.386084004	-0.322709199	1.621511561	0.993623703	1.442396453
659473	662449	13	+	CIK1	ORF-T	ORF-T	0.025890692	0.048772619	0.02028018	-0.594418273	1.081534178
661705	662449	13	+	unknown	no_ovlp2	SUTs	0.742097319	0.305297226	-0.558743225	-0.626491165	0.272311894
662449	664401	13	+	CLN1	ORF-T	ORF-T	-0.041419651	-0.42682466	-0.781840746	-0.432755963	-0.297709663
664649	665865	13	+	ROT1	ORF-T	ORF-T	-0.227664128	-0.775565652	0.039634323	0.209101152	-0.097849017
667489	668321	13	+	ERG2	ORF-T	ORF-T	-0.262125016	-0.245576061	-0.209921114	-0.00078859	0.251373037
668433	669889	13	+	TOM40	ORF-T	ORF-T	-0.655891228	-0.549274883	-0.169391312	-0.062890395	0.003333655
675177	675721	13	+	unknown	no_ovlp2	CUTs	1.44065065	-0.330689087	-0.429923993	-0.221230136	0.082554139
675721	677057	13	+	YMR206W	ORF-T	ORF-T	-1.076773942	-0.844506794	2.542922252	2.607391243	-0.485768777
684409	685881	13	+	ERG12	ORF-T	ORF-T	-0.326372962	-0.605841339	-0.557228104	-0.120120554	-0.455894599
687505	688937	13	+	YMR210W	ORF-T	ORF-T	-0.699867502	-0.536896068	0.803823649	0.364459954	0.248112388
689097	690657	13	+	DML1	ORF-T	ORF-T	0.679242509	-0.048392592	0.108124287	-0.210456508	0.212889819
693369	695177	13	+	CEF1	ORF-T	ORF-T	0.291369412	-0.20402535	0.114827761	0.019041515	-0.102353018
695321	696577	13	+	SCJ1	ORF-T	ORF-T	-0.324360362	-0.158367834	-0.706376609	-0.239060382	-0.084288918
696745	698497	13	+	GAS3	ORF-T	ORF-T	-0.505334014	-0.586975849	-0.949297635	-0.01359992	0.856614733
701673	703465	13	+	GUA1	ORF-T	ORF-T	0.07577514	-0.346164737	-0.54152766	0.055922651	0.184310369
707073	710497	13	+	ESC1	ORF-T	ORF-T	-0.116491836	-0.524347208	-0.178610524	0.133878314	-0.394077358
712305	713761	13	+	ERG8	ORF-T	ORF-T	0.160018154	-0.316923378	-0.276132408	-0.162979028	-0.251292504
715665	716209	13	+	CUT309	CUTs	CUTs	2.262386594	-0.559769344	-1.303460751	-0.598788775	-0.432483623
716513	718569	13	+	UBP8	ORF-T	ORF-T	0.458419694	-0.342299898	-0.867487936	-0.607682486	-0.341979708
720945	723265	13	+	unknown	no_ovlp	SUTs	0.163109044	-0.080126163	-0.413762453	-0.255939957	-0.090243102
724625	725745	13	+	MTF1	ORF-T	ORF-T	-0.35047721	-0.851787087	-0.020118543	-0.52516563	0.212685069

731449	732369	13 +	SUT313	SUTs	SUTs	0.574323551	0.62453666	0.054021179	0.258475771	-0.562697382
732465	732881	13 +	RPS10B	ORF-T	ORF-T	0.750258481	-0.363766298	-1.199400647	-1.107504292	0.192395398
733249	733505	13 +	unknown	no_ovlp2	CUTs	1.001641272	-0.392586611	-1.270124702	-0.44429751	-0.569657204
733505	736857	13 +	PEP5	ORF-T	ORF-T	-0.218447095	-0.434464969	-0.243739454	-0.35023818	-0.240438468
739177	740025	13 +	TRI1	ORF-T	ORF-T	1.041620823	-0.199737339	-1.163695412	-0.361025588	-1.327535054
740185	741465	13 +	RNH1	ORF-T	ORF-T	0.016288865	-0.988872272	-0.816341852	-0.148326509	-0.596316292
742961	743681	13 +	unknown	no_ovlp2	SUTs	0.061405392	-0.362726237	0.185193113	-0.168313418	-0.199894701
743681	746081	13 +	BCH1	ORF-T	ORF-T	-0.64771404	-0.557526628	0.225626322	0.218794595	0.266570912
746257	747833	13 +	DFG5	ORF-T	ORF-T	-0.569962504	-0.439698932	-0.161197567	0.079932489	0.078297548
748209	748777	13 +	unknown	no_ovlp	CUTs	2.052491274	0.177635321	-0.781774801	-0.293903652	-0.409494904
751657	752953	13 +	YHM2	ORF-T	ORF-T	-0.666241162	-0.363884132	-0.351023859	0.187205777	0.350052215
757297	758489	13 +	unknown	no_ovlp	SUTs	-0.644448124	1.037307483	0.461210418	0.271370944	-0.020012069
758489	758993	13 +	unknown	no_ovlp	CUTs	3.02161539	0.214822595	-0.319555331	-0.024293714	-0.419892689
759553	762073	13 +	FAA4	ORF-T	ORF-T	0.795457448	0.196672015	-0.921990397	-0.193813479	-0.122920527
763377	763961	13 +	SUT314	SUTs	SUTs	0.108213556	-0.076701705	0.622491956	0.290243734	2.173506673
770761	772697	13 +	GAD1	ORF-T	ORF-T	-1.050710634	-0.651095802	1.837795736	1.331229016	1.222037774
772697	773313	13 +	unknown	no_ovlp2	SUTs	-0.403447941	0.306013299	0.31211166	0.509603145	0.582227944
773313	774177	13 +	GTO3	ORF-T	ORF-T	-0.345774056	0.501157512	0.437822475	1.124576804	0.384899726
774177	774657	13 +	SUT315	SUTs	SUTs	-0.259595389	-0.254699119	-0.524183396	-0.628727174	-0.830510132
774713	775217	13 +	HOR7	ORF-T	ORF-T	-0.555353375	-0.330988969	0.491310757	0.212974379	0.187089723
777953	778697	13 +	GFD1	ORF-T	ORF-T	0.301455263	-0.337984609	-0.300297866	0.131784683	-0.586290602
784529	785057	13 +	SUT319	SUTs	SUTs	1.447213088	-0.063909147	0.289218446	0.314692425	0.402424959
789105	789841	13 +	unknown	no_ovlp	CUTs	2.242800263	0.418621567	-1.114399603	0.043633157	0.06162336
793745	794913	13 +	YMR262W	ORF-T	ORF-T	0.12276723	-0.562425084	0.005275732	0.000801789	0.003979622
794913	795617	13 +	SAP30	ORF-T	ORF-T	0.228806836	-0.03656678	0.373657465	0.262719286	0.696551043
795769	796465	13 +	CUE1	ORF-T	ORF-T	-0.234606614	-0.538676467	-0.27100117	-0.175235545	-0.109829498
798497	801601	13 +	RSN1	ORF-T	ORF-T	-0.509889822	-0.71850767	-0.825518976	0.348500553	-0.177769241
801689	802857	13 +	PPA2	ORF-T	ORF-T	-0.781238445	-0.82787433	0.076450232	0.038061605	0.256671385
804433	805137	13 +	TMA23	ORF-T	ORF-T	0.915624963	0.034075027	-0.308350401	-0.339307952	-0.127183829
806729	807865	13 +	unknown	no_ovlp	CUTs	1.110544701	0.191458333	-0.507378622	-0.590818775	-0.310196344
809177	809561	13 +	unknown	no_ovlp	CUTs	1.327933817	0.306385437	0.501531559	0.19031343	-1.229814526
811185	812289	13 +	unknown	no_ovlp	SUTs	0.124579632	0.344010906	0.497259822	1.035314221	0.454959468
818809	820049	13 +	DSK2	ORF-T	ORF-T	-0.233777354	-0.163454327	0.017501851	-0.254378952	-0.075530924
820161	822553	13 +	FCP1	ORF-T	ORF-T	0.003769018	-0.20217103	-0.512874923	-0.020392655	0.224440403
822737	826009	13 +	YMR278W	ORF-T	ORF-T	0.136215365	-0.075689085	0.266770802	0.269572587	0.487106519
832257	833329	13 +	GPI12	ORF-T	ORF-T	-0.323801459	-0.865324051	-0.233962486	0.172516165	0.122829435
835353	836065	13 +	unknown	no_ovlp	CUTs	2.156986205	0.321527807	-0.516656292	0.293346958	0.005808883
838153	840089	13 +	YKU70	ORF-T	ORF-T	-0.302373833	-0.643793276	0.188163815	-0.180482823	0.139020486
841929	842329	13 +	MRPL33	ORF-T	ORF-T	-0.380029985	-0.53415133	0.116951547	0.171986121	0.065244819
845569	848513	13 +	HSH155	ORF-T	ORF-T	-0.032061884	-0.014739317	-0.35238609	-0.103236302	0.07626031
848681	849961	13 +	ABZ2	ORF-T	ORF-T	0.762278369	0.668783493	0.202039658	-0.38251865	0.168616399
852553	854401	13 +	YMR291W	ORF-T	ORF-T	-0.594730033	-1.215149717	0.082020867	0.576349479	-0.529973056
854913	855393	13 +	GOT1	ORF-T	ORF-T	-1.133792451	-1.292988345	0.125009921	0.548581504	0.994577499
856985	858153	13 +	JNM1	ORF-T	ORF-T	0.188880686	-0.234932742	0.073551435	-0.36282882	-0.123000462
859281	859841	13 +	unknown	no_ovlp	CUTs	1.447306192	0.237887198	-1.36983592	-0.021304237	1.446438411
861457	861889	13 +	CUT311	CUTs	CUTs	1.971281482	-0.27709179	0.248996848	-0.13060989	0.587655718
861889	863649	13 +	PRC1	ORF-T	ORF-T	-0.457799452	-0.070571953	0.343217091	0.325651486	0.201798825
863801	864385	13 +	LIP1	ORF-T	ORF-T	-0.0573318	-0.723793819	0.038385786	0.030205495	0.164129834
867561	868089	13 +	unknown	no_ovlp	CUTs	3.040802024	0.809900448	-0.304524815	0.273681021	0.106193311
873009	874345	13 +	SUT320	SUTs	SUTs	1.702669513	0.668969271	-0.421022808	-0.735262871	0.592085988
874793	878873	13 +	UBP15	ORF-T	ORF-T	-0.206423225	-0.070339528	0.425096463	0.118260231	0.359050831
886913	888881	13 +	GAS1	ORF-T	ORF-T	-0.432258347	-0.426331574	-0.541697214	-0.177585896	-0.070900473
896953	898409	13 +	unknown	no_ovlp	SUTs	0.618020654	0.263517446	-0.124989237	0.270782278	0.289841989
898409	899345	13 +	ELP6	ORF-T	ORF-T	-0.180726547	-0.35991012	-0.145734868	-0.287259161	0.160970189
901649	902569	13 +	PRE5	ORF-T	ORF-T	0.47237358	-0.159450932	0.129031634	-0.271787809	-0.368866197
902729	903953	13 +	YMR315W	ORF-T	ORF-T	-0.853459253	-0.472437853	0.140303082	0.18516078	0.093557761
904769	905913	13 +	DIA1	ORF-T	ORF-T	1.131835325	-0.319861598	-0.809875667	0.04738064	-1.103641224
910385	915409	13 +	SUT322	SUTs	SUTs	0.151410738	-0.362327807	0.504709357	0.643743048	0.329594958
915409	917409	13 +	YMR320W	other	other	1.960629556	0.18405933	-0.685234213	-0.17098209	-0.961458802
12981	16645	14 -	unknown	no_ovlp	SUTs	0.71874787	-0.675088145	-0.065918262	-0.530639613	-1.567982148
17821	19333	14 -	RPD3	ORF-T	ORF-T	-0.150591728	-0.860165438	-0.429079726	-0.056128058	-4.637918653
22685	23485	14 -	PEX6, MDJ2	ORF-T	ORF-T	0.006429144	-0.467927654	-0.411026021	-0.465598431	-0.944160984
27293	28365	14 -	PFA3	ORF-T	ORF-T	0.006373131	-0.150085279	-0.014545607	-0.018233013	-0.587389668
28765	31469	14 -	FIG4	ORF-T	ORF-T	-0.152548147	-0.430396265	-0.059734298	-0.403133337	-1.204782762
31469	31765	14 -	CUT795	CUTs	CUTs	2.169121287	-0.428229051	0.054558887	0.355017413	-0.224060984
33205	34269	14 -	KRE1	ORF-T	ORF-T	-0.453689978	-0.406000832	0.019883169	0.345078478	-0.245870519
42029	43117	14 -	PHA2	ORF-T	ORF-T	0.304640821	-0.296257826	-0.159206416	-0.163198043	-0.315490982
43221	44285	14 -	ATP11	ORF-T	ORF-T	-0.511101149	-0.468256748	-0.167895694	-0.145762164	-0.366646713
45229	48045	14 -	YNL313C	ORF-T	ORF-T	0.002199146	0.106122043	-0.490801864	0.024253145	0.544079986
49333	51693	14 -	SKP2	ORF-T	ORF-T	-0.393617402	-0.602503146	-0.192186847	-0.174685802	0.235988491
51821	52461	14 -	ZIM17	ORF-T	ORF-T	-0.273094602	-0.27553625	0.350115189	-0.470099857	0.820057321
53989	55933	14 -	KRI1	ORF-T	ORF-T	0.594467697	-0.341118697	-0.553231877	-0.103976095	0.140582104
56109	57733	14 -	MCK1	ORF-T	ORF-T	-0.486302646	-0.208301286	-0.022056682	0.175846036	0.280131976
58781	59845	14 -	YNL305C	ORF-T	ORF-T	-0.389275866	-0.207632394	0.586321906	0.489980155	0.49319623
62374	62942	14 -	RPS19B	ORF-T	ORF-T	0.47412912	-0.89170957	-0.868852722	-0.78542715	-0.1955647
63390	64590	14 -	RPL18B	ORF-T	ORF-T	3.902425801	-0.71635379	-0.57183533	0.008662529	-0.441105901
64590	64926	14 -	CUT797	CUTs	CUTs	1.533157853	-0.068321459	0.161072188	-0.417944905	-0.514208132
67582	68415	14 -	CUT798	CUTs	CUTs	2.981516438	0.337871065	-1.076807109	-0.107760781	1.083384771
71511	76615	14 -	MON2	ORF-T	ORF-T	-0.063324629	-0.385552254	-0.435117651	-0.086171764	-0.236642413
78511	80431	14 -	RIM21	ORF-T	ORF-T	-0.129441727	-0.765628728	-0.109412977	-0.020143553	-0.571777635
84119	86015	14 -	MID1	ORF-T	ORF-T	-0.314163786	-0.296245878	-0.108571723	-0.268188262	0.595433614
86831	87279	14 -	CUT799	CUTs	CUTs	2.55021884	0.020591984	-0.489073217	-0.388848744	0.88468539

89583	89887	14	-	CUT800	CUTs	CUTs	3.388708268	0.639927409	0.295170251	-0.269551462	-0.740667395
89887	90751	14	-	unknown	no_ovlp	SUTs	0.333164683	0.397591394	-0.342239164	-0.222637189	0.012873849
103055	104063	14	-	MRPL10	ORF-T	ORF-T	-0.565499675	-0.700210525	0.08831451	-0.06869315	0.301553688
104959	107087	14	-	WSC2	ORF-T	ORF-T	-0.414429092	-0.738521714	-0.072550023	-0.192251222	0.482744552
107559	108167	14	-	CUT801	CUTs	CUTs	2.49765213	-0.373701405	-0.666903579	-0.318700866	0.090446791
108999	110503	14	-	ERG24	ORF-T	ORF-T	-0.082633418	-0.645860083	-0.471842399	-0.034266308	0.33023154
110887	112895	14	-	SUT743	SUTs	SUTs	1.627856949	-0.297718206	-0.800693837	-0.451880764	-0.186136869
115775	116471	14	-	unknown	no_ovlp	SUTs	0.381093495	0.521357264	0.112189373	0.585705775	0.299240664
117087	118863	14	-	YNL276C	other	other	0.275858796	-0.616994737	-0.797599273	-0.48841709	-0.415255921
118863	119095	14	-	unknown	no_ovlp	SUTs	0.331424769	-0.023226538	0.493461682	0.032105099	0.303927257
120991	122239	14	-	GOR1	ORF-T	ORF-T	-0.512283322	-0.346144859	1.521895617	0.867719199	0.849734224
126743	129183	14	-	SEC2	ORF-T	ORF-T	-0.161040484	-0.432848713	-0.290248499	-0.224236839	-0.048309698
129375	135647	14	-	BNI1	ORF-T	ORF-T	0.119849049	-0.379643678	-0.318249247	0.04794411	-0.11503722
135647	135871	14	-	unknown	no_ovlp2	SUTs	-0.342620466	0.619372779	0.543343959	0.289713993	0.638829584
135871	138079	14	-	ALP1	ORF-T	ORF-T	0.096582104	0.322130288	1.109578036	1.010265663	0.444030386
137719	138079	14	-	unknown	no_ovlp2	SUTs	0.511988955	-0.361883445	-0.39344205	0.265932253	-1.332565495
144135	145143	14	-	IST1	ORF-T	ORF-T	-0.070289854	-0.53593993	0.195999357	0.124933251	0.40066885
145431	146599	14	-	PDR17	ORF-T	ORF-T	-0.224741773	-0.761146505	-0.030468275	-0.376838843	0.098231689
146807	147983	14	-	YIF1	ORF-T	ORF-T	-0.298279447	-0.266737084	-0.330677833	0.011013417	0.123727716
154415	154783	14	-	CUT803	CUTs	CUTs	2.148547083	-0.143623353	-1.03958153	0.155874963	-0.781461399
156503	157239	14	-	YNL260C	ORF-T	ORF-T	0.133745318	-0.861499919	-0.38043766	-0.426442565	-0.9736078
157559	158007	14	-	unknown	no_ovlp2	SUTs	-0.174278241	-1.097446589	0.271456339	-0.118960513	-0.10782711
158007	160399	14	-	DSL1	ORF-T	ORF-T	0.211863088	-0.430189496	-0.436104837	-0.137432081	-0.263774768
160631	164359	14	-	SIP3	ORF-T	ORF-T	-0.010919788	-0.031930268	0.056790539	-0.229809507	0.294622395
167055	167815	14	-	GIS2	ORF-T	ORF-T	0.102734802	-0.822257133	-0.729961929	-0.087080325	-0.227995927
167815	169279	14	-	YNL254C	ORF-T	ORF-T	0.672275562	-0.065310542	-0.511017426	-0.61522862	0.230434897
169279	169527	14	-	unknown	no_ovlp	CUTs	1.38937964	0.040713757	-1.50043145	-1.415826364	1.311578411
171295	172303	14	-	MRPL17	ORF-T	ORF-T	-1.391430497	-1.101289876	0.154426852	-0.108343668	0.128214541
172439	174639	14	-	NRD1	ORF-T	ORF-T	1.651510914	-0.633102956	-0.464688553	-0.188112077	-0.252727694
178879	181167	14	-	MPA43	ORF-T	ORF-T	-0.135832436	-1.099724638	-0.31556529	-0.172393243	-0.066774592
181167	182647	14	-	RPA49	ORF-T	ORF-T	0.406382082	-0.096383246	-0.627315378	-0.017942049	0.189943158
184783	185239	14	-	CUT804	CUTs	CUTs	3.700160637	0.272496972	-1.481383655	-0.066631805	0.663614035
185607	186887	14	-	CWC25	ORF-T	ORF-T	0.095908778	-0.324435706	-0.526596529	-0.034981219	0.21274144
187071	187535	14	-	SUI1	ORF-T	ORF-T	-0.549062749	-0.723166484	-0.5174512	-0.32581102	0.323395194
187535	189719	14	-	unknown	no_ovlp	SUTs	-0.005262062	0.494928039	0.078941147	0.078075454	0.856445654
193207	194823	14	-	unknown	no_ovlp	CUTs	3.198708184	0.351842479	-1.744872436	-1.276373285	-0.979050017
196231	198015	14	-	ZWF1	ORF-T	ORF-T	-0.578679104	-0.670412988	0.133403944	-0.087135753	0.169911555
198399	200031	14	-	NAR1	ORF-T	ORF-T	-0.070566302	0.167784257	0.229743967	0.014016669	0.727647113
201119	201967	14	-	SUT744	SUTs	SUTs	1.872782171	-0.013163267	-0.913905106	-0.310581062	-0.197596672
204999	206695	14	-	SUT745	SUTs	SUTs	0.388126018	-1.082596282	-0.688378512	-0.309602812	-0.614222256
215967	217255	14	-	PDR16	ORF-T	ORF-T	0.517815573	-0.754099139	-0.659958573	-0.402778175	-0.520482586
217255	218727	14	-	ELA1	ORF-T	ORF-T	0.424949061	0.144930328	0.092315069	-0.381901033	-0.242171992
218919	220415	14	-	URE2	ORF-T	ORF-T	-0.002144088	-0.354229644	-0.297990309	-0.180525707	-0.315804464
220591	224503	14	-	JJJ1	ORF-T	ORF-T	0.026464241	-0.020223049	-0.00635822	0.091119107	0.554998563
222479	224503	14	-	unknown	no_ovlp2	SUTs	-0.404044236	-0.994503886	-0.025120689	-0.252904107	0.099811372
224647	227183	14	-	SQS1	ORF-T	ORF-T	0.005587743	-0.370465073	-0.074364209	0.071972799	0.011475711
230127	230719	14	-	SNR19	other	other	0.893662654	0.214136033	-0.072282777	0.010294043	0.312720131
233695	234095	14	-	POP1	ORF-T	ORF-T	0.300112897	-0.42017872	-0.783773854	-0.853614136	-0.838771155
235903	237807	14	-	ALG9	ORF-T	ORF-T	-0.803884797	-0.495672524	-0.132480592	-0.080041392	0.297339605
239191	239895	14	-	unknown	no_ovlp	CUTs	1.59187805	0.206031476	-0.265728629	0.855229477	0.902510556
240943	241311	14	-	CUT805	CUTs	CUTs	2.480717419	-0.04658222	-0.266174591	0.155816274	0.626514857
241311	245551	14	-	unknown	no_ovlp	SUTs	0.535569549	0.419172014	0.016709626	0.828794235	0.7827064
246343	247199	14	-	YNL213C	ORF-T	ORF-T	-0.264705759	-0.400652629	0.178675943	-0.359964249	0.269801017
249831	251719	14	-	SUT746	SUTs	SUTs	1.354300691	-0.594701116	-0.486049563	-0.226581847	-0.081476428
252031	255023	14	-	unknown	no_ovlp	SUTs	0.644294104	-0.083625362	-0.641869793	-0.75710465	-0.284534595
256719	258167	14	-	RTT106, YNL	ORF-T	ORF-T	-0.308672822	-0.772534416	-0.742370459	-0.194755097	-0.055215356
258487	260623	14	-	YNL205C, SP	other	other	0.660857415	0.098923255	-0.581749847	0.128599844	-0.068477907
260375	260623	14	-	unknown	no_ovlp2	SUTs	0.274436951	-0.855033942	-0.672879894	-0.123930296	0.129011157
260623	263463	14	-	PSY2	ORF-T	ORF-T	0.208269424	-0.499584433	-0.591295887	-0.156894502	-0.091563034
263631	264543	14	-	YNL200C	ORF-T	ORF-T	0.196883634	0.699094093	1.13053149	0.748873407	-0.006714867
264863	266663	14	-	GCR2, YNL19	ORF-T	ORF-T	0.427867158	-0.515213869	-0.137232794	-0.191123944	-0.269836126
269695	269959	14	-	WHI3	ORF-T	ORF-T	-0.607014359	-0.689848837	-0.202813132	0.073085746	0.151301635
271327	271543	14	-	unknown	no_ovlp2	SUTs	-0.811760321	-0.636761822	1.845836577	0.179882398	0.500272759
271543	272527	14	-	YNL195C	ORF-T	ORF-T	-1.702442363	-0.74535464	3.321984751	2.61251365	0.990147566
272527	273743	14	-	YNL194C	ORF-T	ORF-T	-1.474966515	-1.416200346	2.447644267	2.646806731	0.629097863
275295	276023	14	-	CUT806	CUTs	CUTs	2.380837574	-0.657044683	-0.843114614	-0.352275302	-0.556952178
276023	276799	14	-	unknown	no_ovlp	SUTs	-0.074281909	0.5330891	0.054755275	-0.064807528	-0.075217616
279911	281943	14	-	unknown	no_ovlp	CUTs	1.162760823	0.419772934	-0.171809265	0.38437097	0.129536829
292071	292767	14	-	MRPL19, YNI	ORF-T	ORF-T	-0.961381895	-0.774880451	-0.001267743	0.052173647	-0.098187511
293007	295663	14	-	NPR1	ORF-T	ORF-T	-0.376922719	-0.894639204	-0.206807316	-0.077273467	-0.260362059
295887	297687	14	-	IPI3	ORF-T	ORF-T	0.584647657	-0.322762767	-0.891250817	-0.317868478	0.206398069
299575	301135	14	-	RHO5, YNL17	other	other	-0.090479984	-0.573390163	-0.268945168	-0.146843553	0.154618298
300863	301135	14	-	unknown	no_ovlp2	CUTs	2.508386229	0.082296412	0.707930316	-0.094997495	0.052770496
301135	301671	14	-	unknown	no_ovlp	SUTs	0.188829878	-0.17073476	0.041706779	0.090817397	-0.650978073
303463	304655	14	-	MRPL22	ORF-T	ORF-T	-0.480409991	-0.738833523	-0.038548732	-0.104203745	-0.160110466
305759	307151	14	-	YNL176C	ORF-T	ORF-T	-0.594735716	-0.390793435	0.389118175	-0.006334316	0.654228932
307295	308647	14	-	NOP13	ORF-T	ORF-T	0.202099211	-0.082617886	-0.766416634	-0.031646916	0.492872156
308863	310079	14	-	MDG1	ORF-T	ORF-T	-0.069070373	-0.495215762	0.122676832	0.293125323	0.267622771
316047	317735	14	-	PSD1	ORF-T	ORF-T	-0.264626436	-0.527287207	-0.991582309	-0.126224076	0.026437378
317887	318855	14	-	FMP41	ORF-T	ORF-T	0.091599603	-0.030167013	0.090432138	-0.374213626	-0.17570078
320199	321719	14	-	SKO1	ORF-T	ORF-T	-0.185821365	-0.599172823	-0.141905055	0.051253459	-0.252915039
322135	323583	14	-	BNI5	ORF-T	ORF-T	0.307736877	-0.143284378	-0.233332974	-0.163740856	0.314574217

325183	326479	14	-	IBD2	ORF-T	ORF-T	0.75872937	-0.010080796	-0.326372657	-0.870842709	0.595687217
326623	330111	14	-	RIA1	ORF-T	ORF-T	0.284340701	0.17071897	-0.180325801	-0.034267468	0.466836321
335175	335503	14	-	SUT747	SUTs	SUTs	-0.182228529	-0.774135122	0.467342003	0.080410555	0.071138819
335503	336047	14	-	SUT747	SUTs	SUTs	0.120945735	-0.34033826	-0.0968588	-0.027073234	-1.088423704
338175	339383	14	-	ASI2	ORF-T	ORF-T	-0.525033501	-0.573246659	0.109915769	-0.308090489	0.136913421
340951	342159	14	-	NSG2	ORF-T	ORF-T	0.333355298	-0.111538051	0.060217335	-0.025352503	-0.035707691
343423	346095	14	-	YCK2, GIM3	ORF-T	ORF-T	-0.207680621	-0.607709811	-0.439409316	-0.138838752	-0.318573808
347655	348575	14	-	RPC31	ORF-T	ORF-T	-0.118615311	-0.608609758	-0.916053862	0.051150574	0.385938174
349247	349783	14	-	PGA2	ORF-T	ORF-T	0.214306432	-0.669178257	-0.36200854	-0.469151202	0.145642944
349783	351839	14	-	ALF1	ORF-T	ORF-T	0.078157005	0.476171573	-0.008423489	-0.276439616	0.593385467
350719	351839	14	-	unknown	no_ovlp2	CUTs	1.491182433	-0.13860346	-1.221997279	-0.395210992	-1.600435436
354503	355159	14	-	YNL144C	ORF-T	ORF-T	-0.093848021	-1.177297821	0.084039149	0.475306453	-1.881638888
355847	356375	14	-	unknown	no_ovlp2	CUTs	2.540527111	-0.341597147	-1.440983078	-0.829240091	-1.193631396
356375	356879	14	-	YNL143C	other	other	0.608752304	-0.953812995	-0.658914446	-0.576861393	-0.657972181
362119	365807	14	-	YNL140C, RL	ORF-T	ORF-T	-0.063384593	-0.588253428	-0.4762562	0.113242068	-0.339268779
366167	366503	14	-	CUT809	CUTs	CUTs	2.239354873	0.369584431	0.483718013	0.162459612	0.018116104
368431	370151	14	-	NAM9	ORF-T	ORF-T	-0.686316416	-0.807814093	-0.078175806	0.098769897	-0.323172963
371671	372255	14	-	FPR1	ORF-T	ORF-T	-0.356948941	-0.623035488	-0.060705875	0.025056436	-0.11841883
372383	373647	14	-	YNL134C	ORF-T	ORF-T	-0.638194839	-0.425732323	0.224285806	-0.07036446	0.225779612
374087	374719	14	-	FYV6	ORF-T	ORF-T	0.485212488	-0.054059521	0.211932155	-0.493399256	0.116489025
379487	380687	14	-	CPT1	ORF-T	ORF-T	-0.243593056	-0.32290288	-0.255153683	-0.183011082	0.243488682
382519	383703	14	-	unknown	no_ovlp	CUTs	1.642210281	0.094528338	-0.571879246	-0.475589934	-1.26277546
390047	392367	14	-	ESBP6	ORF-T	ORF-T	-0.264503704	-0.849956701	0.177206263	0.061252821	0.097423015
392367	394327	14	-	unknown	no_ovlp	SUTs	0.257349015	0.412376791	-0.444162569	0.006104199	0.10581257
397807	398327	14	-	YNL122C	ORF-T	ORF-T	-0.436725044	-0.51702784	0.244656151	0.202625805	0.15591178
398455	400583	14	-	TOM70	ORF-T	ORF-T	-0.523931697	-0.608264705	-0.063522651	0.301226826	0.004384105
404799	405615	14	-	DCP2	ORF-T	ORF-T	0.387647437	-0.36915342	-0.62940876	-0.19549473	-0.558848162
407575	408095	14	-	CUT811	CUTs	CUTs	2.393825277	0.034622794	-0.305360755	-0.272820206	0.270157077
410007	412095	14	-	YNL115C	ORF-T	ORF-T	-0.136944401	0.299432115	0.798581753	0.372987988	0.654686505
416703	417327	14	-	CYB5	ORF-T	ORF-T	0.28142844	-0.917697603	-1.131446061	-0.143252711	-1.013022314
417711	418575	14	-	NOP15	ORF-T	ORF-T	0.473581185	-0.51990777	-0.647037416	-0.349498465	0.185789609
418863	419863	14	-	YNL108C	ORF-T	ORF-T	0.556582051	0.209633885	-0.822583305	-0.126891264	-0.304924251
420895	424575	14	-	INP52	ORF-T	ORF-T	-0.018758603	-0.003511597	-0.23127142	-0.217765255	0.478980785
426919	427239	14	-	LEU4	ORF-T	ORF-T	2.357174165	-0.160300636	0.474934351	-0.45427071	-0.528636371
434119	434559	14	-	CUT812	CUTs	CUTs	2.648563647	-0.585112862	-0.625712696	-0.334365488	-0.319423907
438311	439311	14	-	OCA1	ORF-T	ORF-T	-0.007396663	-0.27228223	-0.213326096	-0.21717603	0.301855569
439495	440663	14	-	RAS2, YNL09	other	other	-0.676566449	-1.105829721	-0.116746982	0.012342219	-0.30028088
441335	442455	14	-	PHO23	ORF-T	ORF-T	0.059066751	-0.304026903	0.253859114	-0.289871028	0.126977104
443839	444183	14	-	RPS7B	ORF-T	ORF-T	0.074430532	-0.807563012	-1.299637245	-0.71895805	-0.362884741
444775	447047	14	-	YNL095C	ORF-T	ORF-T	-0.255215375	-0.143383602	-0.180641634	-0.055913525	1.317644581
449879	452071	14	-	unknown	no_ovlp	SUTs	0.836228057	0.445095619	0.356390585	0.313260571	-0.011250388
459079	459431	14	-	CUT813	CUTs	CUTs	2.846520961	0.421486078	-0.211707193	0.794545457	-1.604211867
466039	466615	14	-	SUT748	SUTs	SUTs	0.57707246	-0.128695565	-0.212033884	-0.159541648	0.933063588
469983	471159	14	-	END3	ORF-T	ORF-T	-0.025909822	-0.596479414	-0.197109949	-0.02861485	-0.21346139
475999	476655	14	-	SWS2	ORF-T	ORF-T	-0.530254246	-0.931080186	-0.181762843	0.083228091	-0.011898452
476831	478127	14	-	EOS1	ORF-T	ORF-T	-0.114647927	-0.443744175	-0.373930724	-0.43254041	0.156317015
478399	479215	14	-	TPM1	ORF-T	ORF-T	-0.04963756	-0.106309142	-0.065611401	-0.191140638	0.008106699
480359	480967	14	-	unknown	no_ovlp	CUTs	1.866618262	-0.654616341	0.60645543	0.905084421	-0.138400834
483823	485311	14	-	CUT815	CUTs	CUTs	1.732331581	0.291872921	-1.811844259	-0.361285165	0.49025482
486671	488175	14	-	MLF3	ORF-T	ORF-T	-0.147570638	-0.311047916	0.230158339	0.175034912	0.046192454
490015	491247	14	-	unknown	no_ovlp	CUTs	1.537889437	-0.322194775	0.256780366	0.228888155	0.46725829
491247	493631	14	-	unknown	no_ovlp	SUTs	0.144093527	0.18138419	-0.259751697	-0.002193419	0.213478235
494519	494983	14	-	RPL16B	ORF-T	ORF-T	0.576772288	-0.849015424	-0.753554533	-1.266736388	0.222290136
495535	498503	14	-	FKH2	ORF-T	ORF-T	0.331714917	0.076024061	-0.180781793	-0.436648804	0.667580463
501783	502959	14	-	unknown	no_ovlp	SUTs	0.589600081	0.291353036	-0.050483991	0.046275839	0.489012185
505752	507264	14	-	YDJ1	ORF-T	ORF-T	0.160230279	-0.140620762	-0.121953572	-0.026428823	-0.207425672
508608	510264	14	-	GCD10	ORF-T	ORF-T	0.273821303	-0.243941659	-0.274197852	-0.181666184	0.63429915
512552	514944	14	-	ARP5	ORF-T	ORF-T	-0.003711856	-0.335794809	-0.427220104	-0.216029809	-0.191525837
515664	516776	14	-	YNL058C	ORF-T	ORF-T	0.120542066	0.085947155	0.026079362	-0.108468557	0.225379974
517872	518920	14	-	POR1, tD(GU	other	other	-0.49029738	0.149652446	0.616563873	0.166657949	0.818354012
518920	522248	14	-	unknown	no_ovlp	SUTs	-0.170335863	0.040601923	0.935128245	0.016285896	0.878284465
525464	525808	14	-	CUT816	CUTs	CUTs	2.55815472	-0.132967644	-0.437841985	-0.105099097	-0.874270334
529160	529560	14	-	CUT817	CUTs	CUTs	4.216405355	-0.11461658	-2.03402362	-0.391753593	0.037821608
530728	531432	14	-	SUT749	SUTs	SUTs	2.327834286	-0.360524135	-0.369309671	0.055757549	0.249974785
531432	532648	14	-	unknown	no_ovlp	SUTs	0.539346626	0.140679051	-0.398501243	0.036517917	-0.299834881
534024	534848	14	-	YNL050C	ORF-T	ORF-T	0.724741052	-0.210635825	-0.362028785	-0.217771894	0.400075231
537384	537920	14	-	SFB2	ORF-T	ORF-T	-0.231925411	-0.00162242	-0.489647856	-0.117010052	-0.158769212
539840	541904	14	-	SLM2	ORF-T	ORF-T	-0.158068644	-0.470913594	-0.23479969	0.00578093	0.410320403
541904	542784	14	-	unknown	no_ovlp	SUTs	0.610893639	0.004143068	0.396457685	0.225398655	0.447516007
544432	544944	14	-	unknown	no_ovlp	CUTs	2.549518601	0.187219251	-1.514867319	-0.687287621	-0.789068688
544944	549312	14	-	YNL043C	other	other	0.077784599	0.325396559	1.085647146	0.662454288	0.85190367
546048	549312	14	-	unknown	no_ovlp2	SUTs	0.009590346	0.45178612	0.606476821	0.419777487	0.149200425
551496	552032	14	-	COG6	ORF-T	ORF-T	-0.010794474	-0.234432638	-0.254393425	-0.302895675	-0.216674168
557784	559168	14	-	IDH1	ORF-T	ORF-T	0.238405425	-0.432483679	0.000423749	-0.113369742	-0.01314929
567664	567992	14	-	unknown	no_ovlp	SUTs	0.511926179	-0.518901109	0.268879986	0.151043829	-0.648020392
568320	569768	14	-	YNL035C	ORF-T	ORF-T	-0.235266791	-0.405877355	-0.188530081	-0.086747072	-0.088381169
569768	574175	14	-	unknown	no_ovlp	CUTs	1.338660316	-0.160070355	-0.613890342	-0.745916531	-0.699351871
575387	576096	14	-	HHT2	ORF-T	ORF-T	-0.436728671	-0.873181636	-0.081926303	-0.213892787	-0.085613103
577208	578808	14	-	KTR5	ORF-T	ORF-T	0.153574857	-0.653918847	-0.313951406	-0.110939319	0.271706137
583920	585382	14	-	SSN8	ORF-T	ORF-T	-0.117882852	-0.590567376	0.349582036	-0.045153755	-0.01311392
586344	586895	14	-	YNL024C-A	ORF-T	ORF-T	-0.567750548	-0.929684743	0.199572504	-0.034009538	-0.199931093
586968	587864	14	-	YNL024C	ORF-T	ORF-T	1.598452266	-0.656263649	-1.583518634	-0.93967535	-0.709431887

588224	591192	14 -	FAP1	ORF-T	ORF-T	0.228193888	0.210466467	-0.373787783	-0.101510611	0.531903382
591304	592895	14 -	YNL022C	ORF-T	ORF-T	0.165026735	-0.573049893	-0.240388715	-0.095781234	0.433320593
595432	597648	14 -	ARK1	ORF-T	ORF-T	0.417704051	-0.134786836	-0.220687021	0.128604025	-0.110107212
604064	605248	14 -	SUT751	SUTs	SUTs	0.065714427	0.436814104	-0.054723097	0.344619318	-0.319628156
611816	613176	14 -	YNL011C	ORF-T	ORF-T	-0.18675905	-1.059537468	0.146304889	0.10925589	0.181376061
616160	618280	14 -	ASI3	ORF-T	ORF-T	-0.219494271	-0.405982605	0.11822951	0.041572513	-0.200856703
618432	619744	14 -	SIS1	ORF-T	ORF-T	0.351480983	-0.568957123	0.031603875	0.066440641	-0.726016512
621240	622480	14 -	MRP7	ORF-T	ORF-T	-0.690735725	-0.74035138	0.036698584	0.012861908	-0.040779381
624576	625888	14 -	PET8	ORF-T	ORF-T	0.321307205	-0.39586912	-0.694987852	-0.23653948	-0.495955439
626056	627184	14 -	RLP7	ORF-T	ORF-T	0.008278048	-1.005805731	-0.682436936	0.072382551	-0.011747229
629448	631168	14 -	CIT1	ORF-T	ORF-T	-0.350386967	-0.382124241	0.832269914	0.391083206	-0.07306263
631168	631808	14 -	unknown	no_ovlp	CUTs	2.092972743	0.420135144	-0.12959671	-0.558321941	-0.829389785
632864	633504	14 -	ATO2	ORF-T	ORF-T	0.499477014	0.283131408	1.996451394	1.210286735	0.73651268
634248	635328	14 -	RPC34	ORF-T	ORF-T	0.450136848	0.010001817	-0.484267125	-0.033752757	0.021964162
635896	636752	14 -	SUT753	SUTs	SUTs	1.028334321	-1.164548913	-0.197342173	-0.412523654	-0.556703042
638888	640192	14 -	ATG3	ORF-T	ORF-T	-0.035738221	-0.225533893	0.495896602	0.079672243	0.037973424
644248	646968	14 -	PRP2	ORF-T	ORF-T	-0.115219223	-0.160029637	-0.223245777	-0.11888742	0.216133391
648936	651752	14 -	PHO91	ORF-T	ORF-T	0.067030413	-0.121864963	-0.625158767	-0.088819148	-0.303180335
661912	662352	14 -	ACC1	ORF-T	ORF-T	-0.351699801	-0.022973091	0.025614721	0.668598245	-0.279197257
663072	665048	14 -	CUT818	CUTs	CUTs	2.299608951	0.045136316	-1.341322891	-0.239377767	-0.306180794
667408	668120	14 -	ATP23	ORF-T	ORF-T	-0.784269539	-0.771703132	-0.21481141	-0.610841197	0.188278978
669656	670224	14 -	MRPL50	ORF-T	ORF-T	-0.59584184	-0.633018369	0.204222119	-0.396569255	0.37907126
672968	674736	14 -	YNR025C, SE	other	other	0.150473906	-0.403473042	-0.480633649	-0.065948288	-0.211959804
677104	678528	14 -	YNR029C	ORF-T	ORF-T	-0.052035187	-0.553328267	-0.367624679	-0.165120091	0.127019706
680608	685696	14 -	SSK2	ORF-T	ORF-T	-0.378901861	-0.65408231	-0.460494133	0.072708651	-0.235791272
687120	687464	14 -	HUB1	ORF-T	ORF-T	0.930873618	-0.038483479	-0.178317522	-0.105897079	-0.017327509
687464	687696	14 -	unknown	no_ovlp	SUTs	0.129616315	-0.348232144	-0.60301649	0.167053316	-0.17914172
689560	689976	14 -	unknown	no_ovlp	CUTs	1.406856288	-0.133430479	-0.528727285	0.636443224	0.705048442
691488	691952	14 -	unknown	no_ovlp	CUTs	1.624691182	-0.301292296	0.626859591	0.568665079	-1.562569963
693024	694080	14 -	ARC35	ORF-T	ORF-T	-0.002970986	-0.44303616	-0.085720457	-0.082198891	-0.578951105
694208	694832	14 -	YNR036C	ORF-T	ORF-T	-0.64379024	-0.577483117	0.083458496	0.06459712	0.091740879
694984	695352	14 -	RSM19	ORF-T	ORF-T	-0.811943428	-0.082530557	-0.074430387	0.090686985	0.26184583
697560	699472	14 -	ZRG17	ORF-T	ORF-T	0.085905345	-0.155161454	-0.577626631	-0.313152358	-0.027907869
700512	701680	14 -	COQ2	ORF-T	ORF-T	0.130031361	0.143056778	-0.11180233	-0.280620347	-0.127993944
707168	707528	14 -	CUT819	CUTs	CUTs	2.664215792	-0.442527469	-1.065688315	-0.378795095	0.351589583
712936	713712	14 -	MSO1	ORF-T	ORF-T	-0.20131158	-0.507925588	0.135943517	0.057746896	-0.321307403
715440	715880	14 -	LYS9	ORF-T	ORF-T	1.100831191	-0.271300782	-0.412244803	-0.346426682	-1.130931124
716376	719080	14 -	BRE5	ORF-T	ORF-T	-0.185801159	-0.500848887	-0.663853797	-0.266204732	-0.46675674
719344	720760	14 -	POP2	ORF-T	ORF-T	-0.248627636	-0.139709433	-0.205499552	-0.096151115	-0.031548134
722312	723160	14 -	NOG2, SNR1	ORF-T	ORF-T	0.445446857	-0.357837201	-1.09452036	-0.301632757	0.152030095
723304	724344	14 -	ESF2	ORF-T	ORF-T	0.728925771	0.296336934	-0.625099229	-0.074063598	0.220632727
727016	727464	14 -	unknown	no_ovlp2	SUTs	0.394990508	0.326565881	0.612150731	-0.044122743	-0.566014832
727464	728224	14 -	YNRCdelta9	other	other	0.325862076	-0.319159832	0.79716943	1.159073516	0.139755606
727792	728224	14 -	unknown	no_ovlp2	CUTs	1.058054077	0.213323778	-0.207167814	0.824304553	-0.022153211
728224	730640	14 -	HOL1	ORF-T	ORF-T	-0.321502705	-0.667183341	-0.124030395	0.050043998	-0.454925428
733240	733824	14 -	BIO5, BIO4	ORF-T	ORF-T	-0.020218912	0.24737086	0.804141838	-0.066901875	1.194497223
735896	736488	14 -	CUT820	CUTs	CUTs	2.530442476	0.259511288	-0.518946357	0.072555496	-0.753882227
740160	742248	14 -	SUT757	SUTs	SUTs	0.355322427	-0.365362424	-0.662692689	-0.447643284	-0.913444625
742568	743616	14 -	YNR061C	ORF-T	ORF-T	-0.115691156	-0.446614082	0.141381459	0.103824297	0.316503775
743616	748904	14 -	unknown	no_ovlp2	SUTs	-0.200502319	0.551386714	0.70706634	0.042209544	0.06140127
748904	750264	14 -	YNR064C	ORF-T	ORF-T	-1.098019787	1.672696998	3.369126232	1.02038828	0.039998441
750032	750264	14 -	unknown	no_ovlp2	SUTs	-0.04099625	0.393254185	0.150245598	-0.035442164	-0.0115084
750264	755248	14 -	YNR065C, YN	ORF-T	ORF-T	-0.387318695	0.366460913	0.234725991	-0.019902355	-0.869334061
755712	759144	14 -	DSE4	ORF-T	ORF-T	-0.593024586	-0.643490795	-0.967037982	-0.556691011	0.062868508
759904	762864	14 -	YNR068C, BS	other	other	1.423574448	0.246704161	-0.049782423	-0.341564135	-3.410454561
764728	771664	14 -	unknown	no_ovlp2	CUTs	1.4794069	1.090814247	-0.519792198	-0.024117012	-2.167168819
771664	776576	14 -	YNR073C	ORF-T	ORF-T	-0.051765738	-0.328962987	-0.170481596	0.250481114	0.27013571
776344	776576	14 -	unknown	no_ovlp2	CUTs	2.379157477	1.991587074	-0.714678745	-1.425271423	-2.571286033
776576	777520	14 -	unknown	no_ovlp2	SUTs	-0.664284601	0.370835768	0.473302206	1.007649616	0.856832236
777520	779680	14 -	AIF1	ORF-T	ORF-T	0.371467198	0.384773381	-0.278462375	-0.031611435	-1.515461847
779200	779680	14 -	unknown	no_ovlp2	CUTs	2.88150533	-0.184409889	-0.665790268	-0.301044824	-3.286208941
22985	24025	14 +	unknown	no_ovlp	SUTs	0.397821087	-0.260335531	-0.418256198	-0.971466451	-1.036082127
24025	27265	14 +	EGT2	ORF-T	ORF-T	-0.553987025	-0.328734724	-0.624425995	-0.082056665	0.058181826
31913	33297	14 +	LEM3	ORF-T	ORF-T	-0.173707099	-0.90159186	-0.666827951	-0.154740526	-0.240331992
34681	37553	14 +	VNX1	ORF-T	ORF-T	-0.443085772	-0.355169463	-0.073843751	0.150477311	0.500973265
37649	38729	14 +	YNL320W, YN	other	other	-0.239830727	-0.618620474	-0.269508147	-0.45120221	-0.545769186
40617	42121	14 +	PFS2	ORF-T	ORF-T	-0.024410411	-0.463497502	-0.015318635	0.073507759	-0.41500914
44433	45281	14 +	DAL82	ORF-T	ORF-T	0.228496413	-0.021910003	0.224320428	-0.104735853	0.13194921
48409	49305	14 +	RFA2	ORF-T	ORF-T	0.004482614	-0.150030084	0.169010414	-0.241398585	0.451577156
51929	52641	14 +	unknown	no_ovlp	CUTs	1.661746702	-0.143923	-1.282869507	-0.257334556	-0.364155542
52641	54121	14 +	STB1	ORF-T	ORF-T	-0.388105508	-0.132059744	0.023047842	-0.24883633	0.375489697
58145	58841	14 +	MRPS18	ORF-T	ORF-T	-0.719801677	-0.504311567	0.060603504	-0.055219753	0.309433282
60257	61674	14 +	YPT11, YNL3	ORF-T	ORF-T	-0.660806804	-0.272196035	0.163924344	0.123055131	0.72336594
65658	66314	14 +	YNL300W	ORF-T	ORF-T	-0.610170966	-0.182400115	0.358335825	-0.33429356	0.911153327
66314	68531	14 +	TRF5	ORF-T	ORF-T	0.289148954	0.047666619	-0.62016103	-0.020998489	1.122692185
68731	69603	14 +	CLA4	ORF-T	ORF-T	0.280718931	-0.248334343	-0.482329802	-0.362541026	-0.349415381
76827	77867	14 +	YNL295W	ORF-T	ORF-T	0.011939207	-0.220805743	0.052015467	-0.013322559	0.078047984
80611	82611	14 +	MSB3	ORF-T	ORF-T	-0.099779215	-0.513140237	0.190051897	0.015744821	-0.017428047
82803	84147	14 +	PUS4	ORF-T	ORF-T	0.157669553	-0.379765815	-0.649806666	-0.198902266	0.285734432
86203	87347	14 +	RFC3	ORF-T	ORF-T	-0.293303329	-0.387217655	-0.179695238	-0.078966203	-0.000828624
87819	88835	14 +	PCL1	ORF-T	ORF-T	-0.407295306	-0.317095117	-0.868463927	-0.839221472	0.331004588
89363	90187	14 +	CUT314	CUTs	CUTs	0.298886669	0.627435556	0.079555254	0.126783166	-0.154126718

90187	91571	14	+	CAF40	ORF-T	ORF-T	0.223481898	-0.41088618	-0.341909295	-0.09195769	0.072547153
91811	94971	14	+	SEC21	ORF-T	ORF-T	-0.337497203	-0.308963753	-0.307283783	0.033851265	-0.197221463
95099	96155	14	+	CUS2	ORF-T	ORF-T	0.236075563	-0.068140443	0.000236118	-0.388753037	-0.327762055
96435	102579	14	+	unknown	no_ovlp	SUTs	-0.518249774	-0.957847571	0.695509457	-0.371336294	-0.735271988
104275	104739	14	+	CUT315	CUTs	CUTs	1.982214215	0.283078527	-0.271516699	-0.423981673	-0.120281157
107683	108427	14	+	POP3	ORF-T	ORF-T	0.389684481	0.206342595	-0.371317589	-0.346897148	0.304472328
108427	109115	14	+	HCH1	ORF-T	ORF-T	0.630228146	-1.062202256	-0.973684918	-0.294029685	-0.566544785
113163	115323	14	+	CAF120	ORF-T	ORF-T	-0.232173354	-0.473794149	-0.600618995	-0.060730261	0.249315638
116483	117251	14	+	unknown	no_ovlp2	SUTs	0.141822451	0.647865534	1.300333568	0.369237608	1.288465324
117251	119067	14	+	MET2	ORF-T	ORF-T	1.077819481	0.918705307	0.38815042	0.121263394	0.250198879
119067	121091	14	+	BOR1	ORF-T	ORF-T	0.052611204	-0.399961703	-0.537038506	-0.254476949	-0.050102281
122851	126699	14	+	TOF1	ORF-T	ORF-T	-0.292797824	-0.17366758	-0.341597831	-0.277905622	0.718792958
138507	140603	14	+	LYP1	ORF-T	ORF-T	-0.016251053	-0.804390086	-0.696957288	0.228020227	-0.569548897
140795	144299	14	+	PIK1	ORF-T	ORF-T	0.035580993	-0.296561083	-0.223960513	-0.126215976	-0.082038686
145483	145931	14	+	CUT317	CUTs	CUTs	3.102480208	0.491865266	-1.261831152	-0.062625679	-0.06981024
148219	151403	14	+	POL2	ORF-T	ORF-T	-0.134969795	-0.077213311	-0.339500182	-0.094213944	0.745460433
154699	154915	14	+	unknown	no_ovlp2	SUTs	-0.107414955	-0.186181136	-0.148067305	-0.101694728	0.765614504
154915	156659	14	+	ORC5	ORF-T	ORF-T	0.827990475	-0.218773635	-0.381763114	0.052215396	0.232039336
160643	161491	14	+	unknown	no_ovlp	CUTs	1.718053356	0.253257196	-1.280492433	-0.220605095	0.661684334
164587	167139	14	+	FOL1	ORF-T	ORF-T	0.387241841	-0.079325757	-1.022368772	-0.270505224	0.287976094
168067	168571	14	+	CUT319	CUTs	CUTs	1.634010579	-0.3443439	-0.770447607	0.134206476	0.119495772
169315	169987	14	+	unknown	no_ovlp2	CUTs	1.723387438	-0.095284443	-0.169958535	0.033220521	-0.030091685
169987	171443	14	+	TEX1	ORF-T	ORF-T	-0.439117827	-0.806538039	-0.0481146	-0.345412238	-0.567912344
174907	175187	14	+	CUT320	CUTs	CUTs	3.346105543	0.509410728	-0.239449669	-0.295386063	0.451546514
175187	179459	14	+	RAD50	ORF-T	ORF-T	-0.16410069	-0.3645237	0.278266088	-0.338535431	0.133607409
182891	185283	14	+	YNL247W	ORF-T	ORF-T	-0.251703025	-0.34822931	-0.446252118	0.068867246	0.01455916
185603	186651	14	+	VPS75	ORF-T	ORF-T	-0.730455468	-0.711297885	-0.28605689	-0.115673225	0.644830558
188019	190963	14	+	SLA2	ORF-T	ORF-T	-0.633514667	-0.246652242	-0.343362981	0.152200431	0.118519061
191315	192459	14	+	ATG2	ORF-T	ORF-T	-0.706529843	-0.538965401	0.682490659	0.37092189	0.210431256
198187	199435	14	+	CUT321	CUTs	CUTs	1.878132711	-0.005454064	-0.554918776	-0.238939608	-0.702920943
200475	202011	14	+	LAP3	ORF-T	ORF-T	-1.056732743	-1.020206317	-0.126171076	-0.023022039	0.28725161
202179	204899	14	+	KEX2	ORF-T	ORF-T	-0.118468435	-0.414905325	-0.492926678	-0.0591804	0.143160825
204899	205139	14	+	unknown	no_ovlp2	SUTs	-0.247202586	0.31289644	0.023505247	0.195652612	0.212492877
205139	206907	14	+	YTP1	ORF-T	ORF-T	0.409454781	1.621105477	2.155570999	1.841756408	2.320797642
206907	209947	14	+	SIN4	ORF-T	ORF-T	-0.186204153	-0.417560226	-0.307740442	0.00404819	-0.345827836
210195	211587	14	+	YNL234W	ORF-T	ORF-T	-0.485979297	-0.87679679	0.575232813	0.613916989	0.313273285
211755	214675	14	+	BNI4	ORF-T	ORF-T	-0.492976968	-0.254727094	0.089993715	-0.129519471	0.458959024
214883	215923	14	+	CSL4	ORF-T	ORF-T	0.336385582	-0.409533753	-0.393586398	-0.223352527	0.061437225
217739	218387	14	+	CUT322	CUTs	CUTs	2.400192082	0.101246831	-0.42930135	-0.013350382	0.440753696
220675	221539	14	+	unknown	no_ovlp	CUTs	1.839937089	0.07279615	-0.037591291	0.536850486	1.219981333
227355	228883	14	+	ATG4	ORF-T	ORF-T	-0.230463871	-0.129573463	0.587977346	0.400764412	0.650599022
229067	229907	14	+	SSU72	ORF-T	ORF-T	0.526214031	-0.690438402	-0.268002712	-0.206654454	-0.178853649
234051	234331	14	+	CUT325	CUTs	CUTs	1.065616587	-1.035094506	-1.146854956	0.007413832	-1.000108583
234331	235819	14	+	ADE12	ORF-T	ORF-T	0.694291559	-0.313626749	-0.177504156	-0.32075199	-0.577513583
238043	240267	14	+	MGS1	ORF-T	ORF-T	-0.052917805	-0.076428782	-0.270512865	-0.433230008	0.744740817
240267	241419	14	+	YNL217W	ORF-T	ORF-T	-0.136714953	-0.571423051	-0.502218617	0.144578699	0.28171704
241587	244307	14	+	RAP1	ORF-T	ORF-T	-0.216438566	-0.528976574	-0.758115305	-0.209017572	0.179489401
244411	245443	14	+	IES2	ORF-T	ORF-T	0.232766778	-0.548362224	-0.123720629	-0.012937253	-0.203354608
245443	246475	14	+	PEX17	ORF-T	ORF-T	-0.182287386	-0.259253947	0.775138449	0.25832807	0.953745187
247411	249955	14	+	VID27	ORF-T	ORF-T	0.018778494	-0.018406432	0.121305551	-0.144757297	0.442809824
252051	254035	14	+	SSB2	ORF-T	ORF-T	-0.396127533	-0.407036627	-0.463280456	-0.120037519	-0.104228256
254395	255131	14	+	YNL208W	ORF-T	ORF-T	-0.250657055	0.206780298	0.174112517	0.233010286	0.167784513
255243	256723	14	+	RIO2	ORF-T	ORF-T	0.285657035	-0.201649617	0.016366769	-0.062841053	0.81247478
263587	264819	14	+	unknown	no_ovlp	CUTs	1.020230286	0.466669188	-0.119432894	-0.57435185	-0.797162506
270203	271219	14	+	SUT325	SUTs	SUTs	1.350039471	-0.319805034	0.179525751	0.385159725	-0.023124096
271987	272531	14	+	SUT326	SUTs	SUTs	1.752574814	1.243889873	-0.146940367	0.243643691	0.691472208
274243	276371	14	+	YNL193W	ORF-T	ORF-T	-0.014478706	-0.152549362	0.619044494	0.288301555	0.39916424
276035	276371	14	+	unknown	no_ovlp2	CUTs	1.279751747	0.645283435	-0.306480618	0.349932949	-0.942065793
276371	280003	14	+	CHS1	ORF-T	ORF-T	-0.096327501	-0.44465508	-0.421591832	0.016336996	-0.255846677
280411	281595	14	+	DUG3	ORF-T	ORF-T	0.541239212	0.117149472	0.254479749	-0.817039291	0.761916253
282155	283035	14	+	YNL190W	ORF-T	ORF-T	-0.147312035	0.058646306	0.008412632	0.049099497	-0.077976068
283763	284067	14	+	unknown	no_ovlp	SUTs	-0.289120675	0.180894886	0.515211913	0.037110427	0.348692705
284067	286035	14	+	SRP1	ORF-T	ORF-T	-0.203990267	-0.391803729	-0.09950204	0.071883651	-0.045158765
286315	287563	14	+	KAR1	ORF-T	ORF-T	-0.342364094	-0.581015571	-0.607372533	-0.720849114	0.092815926
288051	289315	14	+	YNL187W	ORF-T	ORF-T	1.034677824	0.989061113	-0.051459236	-0.153639524	0.09344382
289315	291987	14	+	UBP10	ORF-T	ORF-T	0.39729893	-0.053762139	-0.543756519	-0.407127894	-0.256631654
298267	299667	14	+	YNL181W	ORF-T	ORF-T	0.593138438	0.169548879	-0.08083967	-0.216857187	0.34374111
301123	302667	14	+	CUT329	CUTs	CUTs	1.364354674	-0.034199564	0.276854982	-0.20114509	-0.428861711
302667	303467	14	+	RPS3	ORF-T	ORF-T	-0.32598179	-0.441277111	-0.213759515	-0.310244907	-0.111322945
307363	308347	14	+	unknown	no_ovlp	CUTs	2.57720015	0.10170884	-0.330002602	-0.108039225	1.119897377
308899	311211	14	+	unknown	no_ovlp	CUTs	1.236045565	0.126820075	-0.833708952	-0.403074698	0.199743159
311211	316171	14	+	APC1, YNL17	other	other	-0.318337391	-0.2764813	-0.160707188	-0.186997027	0.716865925
318019	319683	14	+	SUT327	SUTs	SUTs	0.468103807	-0.489826936	-0.482116815	-0.270270451	0.500867094
323803	325243	14	+	YNL165W	ORF-T	ORF-T	0.104593786	0.132986192	0.434261655	-0.118059796	1.032745411
329299	330331	14	+	unknown	no_ovlp	SUTs	0.822217994	0.491296811	-0.705453952	-0.412790397	1.665085855
330331	330923	14	+	YNL162W-A	ORF-T	ORF-T	1.126060679	0.101512082	-0.072825236	-1.296792557	0.937544225
331283	332075	14	+	RPL42A	ORF-T	ORF-T	0.449995839	-0.662839078	-0.79881062	-0.659815842	0.051885611
332251	332475	14	+	unknown	no_ovlp	SUTs	-0.414214591	1.159513827	0.757278442	0.833440671	0.315703944
332475	336499	14	+	CBK1	ORF-T	ORF-T	-0.366644163	-0.385161147	-0.184187375	-0.30434164	0.288332461
335235	336499	14	+	unknown	no_ovlp2	SUTs	0.686613522	0.076065806	-0.107069437	-0.138248898	0.560478018
336499	337843	14	+	YGP1	ORF-T	ORF-T	-0.350226341	-1.122917849	0.003785826	0.046577922	0.056024896
339579	340347	14	+	PGA1	ORF-T	ORF-T	0.149950465	-0.318336594	0.08010031	-0.22085114	0.016271921

340347	340947	14	+	IGO1	ORF-T	ORF-T	0.141210515	-0.955380863	-0.445889462	0.126940662	0.159670443
342387	343443	14	+	YNL155W	ORF-T	ORF-T	-0.164852661	-0.429770226	0.495920822	-0.433392978	1.098686913
346299	347611	14	+	YNL152W	ORF-T	ORF-T	-0.022824868	-0.655977735	-0.328439606	-0.229742883	-0.233766787
348763	349163	14	+	CUT332	CUTs	CUTs	2.583410249	0.112495478	-0.678706303	-0.219982172	0.784218529
350587	350859	14	+	unknown	no_ovlp	CUTs	2.416889048	-0.178617697	-0.070880797	0.089964609	0.439138843
351067	351427	14	+	LSM7	ORF-T	ORF-T	-0.405510072	-1.529576683	-0.248094917	0.181593245	-0.201448177
351427	352363	14	+	YNL146W	ORF-T	ORF-T	0.50469199	0.802004912	0.87394505	0.210127772	0.728771936
352363	352707	14	+	MFA2	ORF-T	ORF-T	-0.184303439	-0.055269198	-0.717223639	0.111216572	-0.584335595
355843	357155	14	+	SUT329	SUTs	SUTs	-0.056246976	-0.921132812	-0.23659032	-0.135324464	-0.771017012
357155	357387	14	+	MEP2	ORF-T	ORF-T	1.708713644	-0.282543305	0.027089627	0.362846155	-0.371322146
359603	360763	14	+	AAH1	ORF-T	ORF-T	0.047901442	-0.561144667	-1.46721018	0.365362111	0.430778578
366115	366635	14	+	YSF3	ORF-T	ORF-T	-0.016053064	-0.981256434	0.263819072	-0.087500812	0.127702694
366723	368515	14	+	SRV2	ORF-T	ORF-T	-0.628420621	-0.230070391	-0.056049913	0.017314693	-0.022252533
370363	371771	14	+	EAF7	ORF-T	ORF-T	0.237136514	-0.571676975	-0.303156165	-0.214842315	-0.253758876
372435	373995	14	+	SUT330	SUTs	SUTs	0.745629154	0.279102219	-0.466777672	-0.100686944	0.799413977
373995	374331	14	+	SUT330	SUTs	SUTs	1.580769071	-0.721535919	0.047183273	0.069442467	0.267326578
375243	378563	14	+	KRE33	ORF-T	ORF-T	0.145141294	-0.149356704	-0.918298059	0.130618739	-0.013135675
378731	379531	14	+	TOM22	ORF-T	ORF-T	-0.317091871	-1.05437693	-0.670975405	-0.11093305	-0.40344222
381123	381339	14	+	unknown	no_ovlp2	CUTs	1.998913617	-0.626045838	-0.850775739	-1.085173405	-0.336029154
381339	382339	14	+	NRK1	ORF-T	ORF-T	0.127512271	-0.269993107	-0.320346924	-0.433517323	-0.891855693
383907	387115	14	+	FAR11	ORF-T	ORF-T	0.336388406	-0.192047062	-0.121982405	0.326815676	-0.54334737
387203	389843	14	+	SPC98	ORF-T	ORF-T	-0.213255604	-0.528826384	-0.377682197	-0.119027754	0.160300918
392883	394419	14	+	NAF1	ORF-T	ORF-T	0.557826241	0.193069951	-0.643966317	-0.459398848	0.376262968
394675	397763	14	+	NMA111	ORF-T	ORF-T	0.000801198	-0.097507814	-0.471602973	0.087877814	0.17867037
401011	402651	14	+	NCS2	ORF-T	ORF-T	0.471702894	-0.053618666	-0.33087965	0.087068492	0.716120221
402651	406307	14	+	unknown	no_ovlp2	SUTs	-0.116503932	0.3711998	-0.04934993	-0.062162177	0.084110107
406307	408307	14	+	MLS1	ORF-T	ORF-T	-0.219702666	1.069048277	2.580573631	0.30726085	0.197939522
408091	408307	14	+	unknown	no_ovlp2	SUTs	-0.284589302	0.946732916	0.879471232	0.01661416	0.017987458
408307	410075	14	+	DMA2	ORF-T	ORF-T	-0.136129389	0.169433251	0.596872223	0.113623511	1.170392
412731	413339	14	+	RPC19	ORF-T	ORF-T	0.140581014	-0.106761916	-0.682517205	-0.415764947	0.18154453
413603	414915	14	+	DBP2	ORF-T	ORF-T	0.556264636	-0.620550888	-1.046099728	-0.114273027	0.189617446
417835	418219	14	+	CUT335	CUTs	CUTs	3.589290888	-0.023225658	-1.395080738	0.094965554	0.140792912
418803	419123	14	+	unknown	no_ovlp	CUTs	1.570549027	-0.046279236	0.28684971	0.846821935	0.319752875
420083	420979	14	+	YAF9	ORF-T	ORF-T	0.352036808	-0.055936723	-0.050053333	-0.211537797	0.365904383
427595	429363	14	+	MET4	ORF-T	ORF-T	0.449304729	-0.253687259	-0.131237645	-0.24068709	-0.242789299
430035	432683	14	+	POL1	ORF-T	ORF-T	-0.434449839	-0.05937466	-0.909252715	-0.181727235	0.430815299
434811	437355	14	+	AVT4	ORF-T	ORF-T	-0.258336298	-0.700792306	-0.419866243	-0.379685828	-0.18194473
437579	438443	14	+	YNL100W	ORF-T	ORF-T	-0.074365707	-0.903135516	0.114480483	0.059432989	-0.241674314
439539	441131	14	+	unknown	no_ovlp	CUTs	1.411057525	0.193673801	-0.770828504	0.570893812	0.628675607
441131	441995	14	+	unknown	no_ovlp	SUTs	0.818467349	-0.978759909	-0.370014426	0.158803935	0.021343332
447355	449411	14	+	APP1	ORF-T	ORF-T	0.010669286	-0.333624019	0.05205605	-0.004924847	0.014626199
449411	450827	14	+	unknown	no_ovlp2	SUTs	-0.709808642	0.304530838	2.388095902	1.800658412	0.709867121
450827	452283	14	+	YNL092W	ORF-T	ORF-T	-0.679412883	0.029885101	1.31360565	0.40004759	1.57363344
452283	454507	14	+	NST1	ORF-T	ORF-T	-0.041250967	-0.344681603	-0.076700676	-0.003237164	-0.134389839
456427	457499	14	+	RHO2	ORF-T	ORF-T	0.036567678	-0.03971503	0.104760838	-0.31383114	0.686748567
457659	459683	14	+	TOP2	ORF-T	ORF-T	-0.344549434	0.023769159	0.288833752	0.002918093	1.270230706
462411	466131	14	+	TCB2	ORF-T	ORF-T	0.003264218	-0.116809469	-1.132365481	0.069885081	0.020415734
466131	466971	14	+	YNL086W	ORF-T	ORF-T	0.800062775	0.800912981	0.691855865	0.138910606	-0.014475728
466755	466971	14	+	unknown	no_ovlp2	SUTs	0.960853778	0.719886583	-0.206736383	-0.667462406	-0.480239654
466971	469907	14	+	MKT1	ORF-T	ORF-T	-0.574142581	-0.544765334	-0.274829165	0.137612588	-0.273231771
471379	473179	14	+	SAL1	ORF-T	ORF-T	-0.591775334	-0.363752331	0.146588522	-0.113270269	0.060350772
473435	476091	14	+	PMS1	ORF-T	ORF-T	-0.3779795	-0.184018621	0.04382268	-0.336592183	0.619591789
478427	479699	14	+	unknown	no_ovlp	CUTs	1.74618955	0.443715577	-1.528481544	-0.399576292	0.89669306
479699	481267	14	+	unknown	no_ovlp2	SUTs	-0.600568176	-0.318815282	-0.01291178	-0.06048222	0.299686895
481267	483067	14	+	APJ1	ORF-T	ORF-T	0.651860904	-0.259692554	0.037897273	0.526874548	-0.594762729
483331	485355	14	+	MKS1	ORF-T	ORF-T	-0.164747777	-0.780560622	-0.096369047	-0.091712416	-0.182494817
485555	486499	14	+	IMP4	ORF-T	ORF-T	0.518962101	-0.829249098	-0.561496982	0.055613074	0.268631482
488387	490187	14	+	MSK1	ORF-T	ORF-T	-0.296881657	-0.658802864	-0.348974794	-0.095434094	-0.440102615
490315	491307	14	+	RNH201	ORF-T	ORF-T	-0.55669348	-0.292571933	-0.26576536	-0.16043748	0.473011452
491483	493131	14	+	LAT1	ORF-T	ORF-T	-0.387668966	-0.344722522	-0.122842937	0.178683723	0.044874094
493323	493691	14	+	TOM7	ORF-T	ORF-T	-0.535012184	-0.846345128	-0.597917306	-0.288497413	-0.278884438
499075	499635	14	+	unknown	no_ovlp	SUTs	0.089734861	0.341743734	0.396000958	-0.239519583	-0.310221501
499635	500675	14	+	RPL9B	ORF-T	ORF-T	-0.799688704	-0.364003177	-0.611783401	-0.245514182	-0.38717747
500931	501155	14	+	SUN4	ORF-T	ORF-T	-0.183587769	-0.776881351	-1.272232598	0.262715766	-0.900724212
503643	505588	14	+	AQR1	ORF-T	ORF-T	0.197261422	0.3107384	-1.0036413	-0.554250126	0.719184006
507620	508828	14	+	MTQ1	ORF-T	ORF-T	0.6334657	-0.349021425	0.03633978	-0.117499273	-0.164324667
510500	512532	14	+	NOP2	ORF-T	ORF-T	0.065635504	-0.284326326	-0.54901373	0.140034878	0.02420467
515196	516084	14	+	YNL057W	other	other	-0.025785994	-0.424512675	0.150818835	0.1353719	-0.334930821
517204	518020	14	+	OCA2	ORF-T	ORF-T	-0.204803092	-0.093616087	0.366989858	-0.165192535	0.369619202
522252	525060	14	+	unknown	no_ovlp	SUTs	-0.197108037	-0.97499833	0.297538776	-0.19759567	-0.989613786
526044	529844	14	+	VAC7	ORF-T	ORF-T	-0.618542737	-0.356615765	0.118442681	0.047953609	0.14319283
529844	531540	14	+	MSG5	ORF-T	ORF-T	0.286446237	-0.389406382	-0.743669055	-0.439263549	-0.038768151
531700	532476	14	+	COX5A	ORF-T	ORF-T	-0.150253467	-0.299150001	0.210643627	0.121796525	0.339670702
532668	533260	14	+	COG5	ORF-T	ORF-T	0.775508903	-0.384342332	-0.48803086	-0.364150823	-0.520449104
538156	539924	14	+	ALG11	ORF-T	ORF-T	0.291538938	-0.107496545	-0.27546074	-0.315455716	-0.211859927
542228	542980	14	+	unknown	no_ovlp2	SUTs	-0.453149294	-0.929460623	0.255281524	0.00459651	0.521292655
542980	545092	14	+	YNL045W	ORF-T	ORF-T	0.038122032	-0.152339522	0.422618115	0.402852926	-0.089670263
545380	546060	14	+	YIP3	ORF-T	ORF-T	-0.377627458	-0.376554681	-0.412188149	-0.139471934	0.123039728
546580	547076	14	+	unknown	no_ovlp	SUTs	-0.411532064	-0.512163939	0.921878472	0.168103728	0.063135883
547964	549380	14	+	BOP3	ORF-T	ORF-T	0.405371785	-0.781083013	-0.400616124	-0.012575739	-0.35529008
552244	555076	14	+	YNL040W	ORF-T	ORF-T	0.70272916	0.605956717	-0.29042696	-0.066838397	0.211375307
555076	556868	14	+	BDP1	ORF-T	ORF-T	0.263778346	0.155693474	-0.157170142	0.016788929	0.370693098

557028	557900	14 +	GPI15	ORF-T	ORF-T	-0.542284497	-1.135692898	-0.113258045	0.105856508	0.52449682
559476	559748	14 +	unknown	no_ovlp	CUTs	1.052546651	0.663866567	0.264113415	-0.328369563	-0.077262013
559748	560612	14 +	NCE103	ORF-T	ORF-T	0.460277213	-0.293739791	1.728878894	0.755794446	0.866162712
568036	568276	14 +	unknown	no_ovlp	SUTs	-0.019337391	0.477259361	0.118137837	-0.603241889	0.592444219
568276	568796	14 +	CUT340	CUTs	CUTs	3.128368916	0.342998851	-1.328452598	-0.378576242	-0.512972309
574411	575417	14 +	SIW14	ORF-T	ORF-T	0.288265662	-0.48146877	0.052861903	-0.204416384	0.038507316
576708	577228	14 +	HHF2	ORF-T	ORF-T	-0.446829527	-0.792138677	-0.431049093	-0.096796279	-0.177589874
579060	579484	14 +	CUT341	CUTs	CUTs	1.304341047	-0.794659762	-0.072951615	-0.119814306	-0.141232085
579484	580524	14 +	CRZ1	ORF-T	ORF-T	-0.021727719	-0.872950905	-0.29059804	0.366647479	-0.584074981
581916	583508	14 +	SAM50	ORF-T	ORF-T	-0.126655814	-0.078789121	0.21119554	-0.106275376	0.010696836
585588	585948	14 +	NME1	other	other	1.220119003	0.39840697	0.009523272	-0.2452132	0.278612064
586100	586408	14 +	SNR66	other	other	0.915502061	-1.486714409	0.213791621	0.03013213	-0.214422023
586175	586408	14 +	unknown	no_ovlp2	CUTs	4.430954848	-0.81562486	-0.268547403	-0.208406537	-0.06423778
593188	595403	14 +	HDA1	ORF-T	ORF-T	0.033452026	-0.761972417	-0.618313438	-0.220241725	-0.304508975
597860	599684	14 +	unknown	no_ovlp	SUTs	0.806091008	-0.367501982	-0.336547788	-0.373085051	-1.233199974
602812	604692	14 +	PUB1	ORF-T	ORF-T	-0.285659659	-0.355202618	-0.303841643	-0.017660902	-0.342078615
604844	605316	14 +	unknown	no_ovlp	SUTs	-1.052330783	-0.539635173	0.239489768	0.244833953	0.157945103
605316	605804	14 +	PBI2	ORF-T	ORF-T	-0.057459559	0.518153986	0.897125486	0.25578834	0.647147513
605804	606140	14 +	unknown	no_ovlp2	SUTs	0.863290679	0.261434327	-0.874418796	-0.467791843	0.19566002
606140	609452	14 +	HEF3	ORF-T	ORF-T	0.40269155	0.510160515	1.072831817	0.646734859	1.041751949
611004	611860	14 +	SPO1	ORF-T	ORF-T	0.196896486	1.654447581	2.11801986	-0.198221695	0.180214749
613580	614484	14 +	YNL010W	ORF-T	ORF-T	0.167949169	-0.18664323	-0.535990586	-0.11554017	-0.161231844
614484	614748	14 +	unknown	no_ovlp2	CUTs	3.28963419	0.595051451	0.068043402	-0.232986815	-0.779989138
614748	616924	14 +	IDP3	ORF-T	ORF-T	0.329057105	-0.328806952	0.261063101	-0.078980767	-0.379471127
620068	621324	14 +	LST8	ORF-T	ORF-T	0.099500283	-1.09338546	-0.326286081	-0.058693817	-0.329682247
622884	623300	14 +	HRB1	ORF-T	ORF-T	0.377976041	-0.241421962	-0.653170878	-0.388579393	-0.631972766
627412	628740	14 +	DOM34	ORF-T	ORF-T	0.344633008	-0.779880505	-0.432172457	-0.066780835	-0.713637656
629172	629612	14 +	SUT337	SUTs	SUTs	0.585367132	0.409977967	0.694140876	-0.589009856	-0.654234531
631348	632860	14 +	unknown	no_ovlp	SUTs	0.298287063	0.365463659	0.523888894	0.341702647	0.052713791
632860	634060	14 +	SUT338	SUTs	SUTs	-0.404621524	-0.295769442	0.002717923	0.112553175	-0.447798673
635556	636804	14 +	YNR004W	ORF-T	ORF-T	0.796603148	-0.041570765	-0.23715183	0.085318145	-0.675393
636988	638948	14 +	VPS27	ORF-T	ORF-T	0.245550136	-0.358079173	-0.109602427	-0.128552593	-0.421049048
640356	643580	14 +	LRO1	ORF-T	ORF-T	0.192770237	0.232007272	-0.748489027	-0.272025163	-0.264282078
642476	643580	14 +	unknown	no_ovlp2	SUTs	-0.297687209	-0.340083692	-0.611992172	-0.721842508	1.091971423
643708	644316	14 +	CSE2	ORF-T	ORF-T	0.69012169	-0.135831356	-0.222732557	-0.069976838	-0.429810118
647380	649020	14 +	URK1	ORF-T	ORF-T	0.12179931	-0.448723804	-0.313045744	-0.013951094	0.407055665
652420	653276	14 +	YNR014W	ORF-T	ORF-T	-0.472414831	-1.378810203	1.063727621	0.644340445	0.112290152
653380	654588	14 +	SMM1	ORF-T	ORF-T	0.450578385	0.488336068	0.136096943	0.097157129	0.734379289
662300	662716	14 +	TIM23	ORF-T	ORF-T	0.606744695	-0.223213577	-0.422312115	-0.371154219	-0.196769209
664244	665068	14 +	YNR018W	ORF-T	ORF-T	-0.274683347	-0.362352423	-0.035570283	0.220044557	-0.32674555
665308	666268	14 +	ARE2	ORF-T	ORF-T	0.197092837	-0.219623099	-0.673013873	0.344353266	-0.241667096
668356	669828	14 +	YNR021W	ORF-T	ORF-T	-0.514273325	-0.725906831	-0.376781023	0.187200305	-0.064655991
670388	672156	14 +	SNF12	ORF-T	ORF-T	-0.280425228	-0.675480857	0.087458874	-0.167740311	0.18583195
672412	673068	14 +	YNR024W	ORF-T	ORF-T	0.871483712	-0.087840907	-0.275557298	-0.332772335	-0.304890578
674908	676108	14 +	unknown	no_ovlp2	SUTs	0.180619611	-0.036238018	-0.207930118	0.079467451	0.079101522
676108	677204	14 +	CPR8	ORF-T	ORF-T	-0.401294079	-0.565036281	-0.030472633	0.358977795	-0.110554614
678748	680588	14 +	ALG12	ORF-T	ORF-T	-0.505266605	-0.225113614	-0.200486309	-0.267053943	0.172205345
685940	687252	14 +	PPG1	ORF-T	ORF-T	-0.456758176	-0.758965425	-0.037213038	-0.360850484	0.094778517
687636	690068	14 +	ABZ1	ORF-T	ORF-T	0.745756346	0.667840554	0.219396867	0.309443434	0.017650101
690276	691548	14 +	SOL1	ORF-T	ORF-T	-0.288778254	-0.725678642	0.386450141	0.303445671	-0.001611016
692252	692548	14 +	YNR034W-A	ORF-T	ORF-T	0.820847405	-1.111474585	0.32193361	0.095011532	-0.745487336
692548	693004	14 +	unknown	no_ovlp	SUTs	-0.149015552	-0.968845065	0.662335761	0.879327633	-1.736125341
694372	695596	14 +	unknown	no_ovlp	SUTs	0.768409866	0.544188846	0.205545226	0.314759674	-0.123650276
695596	697612	14 +	DBP6	ORF-T	ORF-T	0.516944787	-0.487188549	-0.230474198	0.038680059	0.152174552
699700	700516	14 +	YNR040W	ORF-T	ORF-T	-0.521845955	-0.12748717	0.191040875	-0.901339553	0.956138584
701884	703100	14 +	MVD1	ORF-T	ORF-T	0.083207902	-0.053487114	-0.263469099	0.099383734	0.195506428
703100	703356	14 +	unknown	no_ovlp2	SUTs	0.740775971	-0.238847645	-0.397707594	-0.146338966	0.142504239
703356	705652	14 +	AGA1	ORF-T	ORF-T	0.442368804	0.356015424	-0.183437626	0.031946068	0.243367415
706116	707756	14 +	PET494	ORF-T	ORF-T	-0.405569708	-0.775289047	0.576642025	0.374079928	0.060774233
707756	708324	14 +	TRM112	ORF-T	ORF-T	0.131076809	-0.035089325	-0.440012742	0.024852977	0.22518336
708468	709980	14 +	YNR047W	ORF-T	ORF-T	-0.13493188	-0.130467911	-0.005919429	-0.04968787	-0.239095771
711612	712972	14 +	YNR048W	ORF-T	ORF-T	-0.04372334	-0.564104546	-0.182621063	-0.261540255	0.242040006
715708	716124	14 +	unknown	no_ovlp	SUTs	0.936872226	-1.76090258	-0.170617141	-0.090427523	-1.224997442
716124	716516	14 +	SNR49	other	other	2.895356195	-2.348058577	-0.517168406	-0.464830796	-0.045905267
723388	724036	14 +	CUT351	CUTs	CUTs	3.146961715	0.684178089	-1.354568135	-0.370785592	1.423050566
724500	725980	14 +	SUT339	SUTs	SUTs	1.549838276	-0.550190317	-0.201874365	-0.171409011	-0.208709409
730900	733268	14 +	SUT342	SUTs	SUTs	1.164418327	-0.282928089	0.003098907	0.141545103	-2.547059265
733268	734012	14 +	unknown	no_ovlp2	SUTs	0.551168153	0.095331567	-0.347172076	-0.313981983	-0.660044528
734012	736748	14 +	BIO3	ORF-T	ORF-T	0.155496373	0.422024766	0.476166023	0.035660612	0.811709566
735804	736748	14 +	unknown	no_ovlp2	SUTs	0.335443733	0.114774561	0.204527519	0.050885602	-0.35169927
736748	738756	14 +	MNT4	ORF-T	ORF-T	-0.340440502	-1.320873614	-0.838967361	-0.19281055	-0.532348031
743812	746324	14 +	SUT344	SUTs	SUTs	0.285050698	0.028229707	-0.295601014	-0.16079542	-0.453981789
746940	750356	14 +	YNR063W	ORF-T	ORF-T	0.34063328	-0.311169918	-0.555264947	-0.129524633	-0.768528307
749708	750356	14 +	unknown	no_ovlp2	SUTs	0.535803867	-0.185252403	-0.592371815	-0.352471857	-0.829781558
755244	755972	14 +	unknown	no_ovlp	SUTs	0.046278208	-0.384301615	0.751843389	-0.076145194	-0.720022458
763076	764044	14 +	CUT353	CUTs	CUTs	1.397104739	0.308074081	-0.076904909	-0.086101344	-2.653871046
765572	769036	14 +	unknown	no_ovlp2	SUTs	-0.897216944	-0.393472131	0.382724209	0.514572303	0.601671609
769036	778524	14 +	YNR070W	ORF-T	ORF-T	-0.213308758	-0.082572148	0.776838679	-0.422663937	-0.02965943
770060	778524	14 +	unknown	no_ovlp2	SUTs	-0.518230826	-0.315631559	-0.101640573	0.269303778	-0.200109113
5469	6901	15 -	SUT761	SUTs	SUTs	0.311175069	-1.524970652	-0.928033209	-0.263212699	-1.626292758
6901	7925	15 -	SUT762	SUTs	SUTs	-0.399236808	-0.101129256	0.01576557	-0.225463076	-0.704790664
14621	15813	15 -	YOL159C-A	ORF-T	ORF-T	0.248944428	-0.565830641	0.494092018	0.062382859	-2.552834023

17053	18029	15	-	YOL159C	ORF-T	ORF-T	0.681989954	0.054142864	0.502201069	-0.13613293	-3.405143689
19205	21445	15	-	ENB1	ORF-T	ORF-T	0.193420463	-0.714399952	-0.425653296	0.227990507	-0.52732653
28837	31685	15	-	HPF1	ORF-T	ORF-T	-0.610825352	-0.18249442	0.918652974	1.002017768	1.612974249
31685	32125	15	-	unknown	no_ovlp	SUTs	0.787281004	0.173430337	-0.292680694	0.051994082	-0.545085543
36981	38637	15	-	YOL153C	other	other	-0.188991953	-0.728873556	0.474214977	0.92468193	-0.038249577
40029	40285	15	-	unknown	no_ovlp	CUTs	2.253429648	0.527699401	0.468434498	0.156939766	-1.508681752
40789	44710	15	-	SUT765	SUTs	SUTs	1.034045977	0.435662731	-0.259637523	-0.112257339	-0.698468934
45742	47614	15	-	SPT20	ORF-T	ORF-T	-0.068380898	-0.40074936	-0.317701878	-0.212325955	-0.285026428
47790	48670	15	-	PEX11	ORF-T	ORF-T	0.19511121	-0.042293123	0.288856521	-0.023575893	0.765090465
49446	52838	15	-	CTR9	ORF-T	ORF-T	-0.126771761	-0.472574449	-0.353442525	0.124653513	-0.232904616
54526	55150	15	-	RIB4	ORF-T	ORF-T	-0.06812016	1.221763875	0.285194777	0.078571848	0.420105509
59094	59798	15	-	unknown	no_ovlp	SUTs	0.46182251	0.844591568	0.122382184	0.40934797	0.632351781
60270	61110	15	-	CDC33	ORF-T	ORF-T	-0.215216531	-1.003025767	-0.676412018	-0.176851338	-0.151037087
61262	65414	15	-	YOL138C	ORF-T	ORF-T	0.046211328	-0.242278319	-0.191398061	-0.336106426	0.112574955
67230	68974	15	-	PFK27	ORF-T	ORF-T	0.52745726	-0.19751801	-0.115470303	-0.204846801	0.058575959
69246	70046	15	-	MED7	ORF-T	ORF-T	-0.33079132	-0.0045996	0.478628445	-0.146706175	0.152505618
71262	72366	15	-	unknown	no_ovlp	CUTs	2.345709692	1.090304622	0.161128865	-0.227130319	-2.019597771
72990	73902	15	-	SUT766	SUTs	SUTs	2.34034583	0.420038103	0.431360425	0.71406761	0.663526957
78110	79806	15	-	YGK3	ORF-T	ORF-T	0.483399076	-0.143137111	-0.50353643	-0.367556811	0.393675812
79478	79806	15	-	unknown	no_ovlp2	CUTs	1.979901135	-0.944964601	-0.702635765	-0.773419928	-0.119046871
81558	82998	15	-	MDH2	ORF-T	ORF-T	-0.71860365	0.368065442	1.075364318	0.322636184	0.631423697
85342	86774	15	-	TRM11	ORF-T	ORF-T	0.124467472	-0.161703507	-0.77067628	-0.202850442	1.078333277
89526	91454	15	-	SMF1	ORF-T	ORF-T	-0.259281122	0.214094141	0.330644415	0.21342103	0.21833495
92446	92846	15	-	RPS19A	ORF-T	ORF-T	0.446814092	-0.631174419	-0.648716097	-0.767469125	-0.19335059
92910	93198	15	-	unknown	no_ovlp	SUTs	-0.383400517	0.265111541	0.303598461	0.167105703	0.313387828
94174	94430	15	-	RPL18A	ORF-T	ORF-T	-0.638629066	-0.689830189	-0.193479633	-0.098104972	-0.458612917
94718	96398	15	-	MCH4	ORF-T	ORF-T	0.254494389	-0.578794341	0.037061849	-0.097543327	-0.254454444
100814	101230	15	-	CUT823	CUTs	CUTs	1.535795721	-0.913678972	-1.336774233	-0.650052194	-0.600596127
103286	103886	15	-	YOL114C	ORF-T	ORF-T	1.21878623	-0.542274347	0.007837462	0.148438894	0.149188758
105662	106278	15	-	CUT824	CUTs	CUTs	2.765761385	0.399934525	-0.307368643	-0.311075322	0.782669128
108190	108958	15	-	MDY2	ORF-T	ORF-T	-0.186602483	-0.697638241	0.118538053	0.135246845	0.117521742
111238	111886	15	-	INO4	ORF-T	ORF-T	-0.249166152	-0.530797422	-0.479677971	-0.22118691	-0.320636485
114006	115958	15	-	WSC3	ORF-T	ORF-T	0.42657512	-0.020995933	0.181079783	0.066177911	-0.054442172
115958	123630	15	-	unknown	no_ovlp	CUTs	2.370598281	0.541834364	-1.706429945	-0.647235943	-0.47733547
125982	126702	15	-	TPT1	ORF-T	ORF-T	-0.67027238	-0.784762587	-0.201114571	-0.32317119	0.251272262
127366	128182	15	-	unknown	no_ovlp	SUTs	0.508790747	0.367752798	0.367899921	0.375533323	0.406055863
132462	136070	15	-	YOL099C, YO	other	other	-0.090112898	-0.461948438	-0.558783057	-0.1692737	-0.544060762
136454	137846	15	-	WRS1	ORF-T	ORF-T	0.335821163	0.049417707	-0.912511716	-0.247137287	-0.49880337
137990	139070	15	-	COQ3	ORF-T	ORF-T	-0.453986223	-0.587059991	0.504996208	0.491327008	0.270559047
139166	141374	15	-	HMI1	ORF-T	ORF-T	-0.12348374	-0.325740825	-0.19214164	-0.173579186	0.194904401
141550	142606	15	-	RFC4	ORF-T	ORF-T	0.080812698	-0.50590523	-0.436304765	-0.565199253	-0.252804414
146878	147174	15	-	CUT825	CUTs	CUTs	2.35247278	0.724857957	-0.126878576	-0.170868334	-0.401644003
150286	153518	15	-	HAL9	ORF-T	ORF-T	-0.26944046	-0.349506787	-0.105886989	0.080653647	0.003408566
153758	154790	15	-	MPD2	ORF-T	ORF-T	0.425550773	-0.516609913	-0.272972349	-0.328568228	-0.425612189
155214	158918	15	-	YOL087C	ORF-T	ORF-T	0.111980854	-0.205031942	-0.165825691	-0.018027645	-0.379312253
159462	160630	15	-	ADH1	ORF-T	ORF-T	-0.615315913	-0.127951228	-0.099438086	0.174968484	-0.297123432
161430	161878	15	-	YOL085C	other	other	-1.714630058	-1.272109623	1.661316331	1.617617749	0.857151585
167502	167862	15	-	CUT827	CUTs	CUTs	2.314218793	1.085040277	1.058427916	0.833549958	-2.192301999
169998	170798	15	-	unknown	no_ovlp	SUTs	0.442951006	-0.573968562	0.047757826	0.215664853	-0.155827412
175430	176438	15	-	unknown	no_ovlp	SUTs	0.847779893	0.876532593	0.257396142	-0.435188442	3.035753753
180366	181478	15	-	REX4	ORF-T	ORF-T	0.360843402	-0.096927134	-1.324703752	-0.654031333	0.402982061
181478	182750	15	-	unknown	no_ovlp	SUTs	-0.192649677	0.167491939	0.468405353	0.071150071	2.373120805
184814	185182	15	-	unknown	no_ovlp	CUTs	2.983365318	0.234572944	1.044557854	-0.093769418	1.39137919
185726	186782	15	-	BRX1	ORF-T	ORF-T	0.246963068	-0.491225054	-0.590541378	-0.017141189	0.370473875
192030	193782	15	-	YOL075C	ORF-T	ORF-T	-1.586594606	-0.420840549	0.12671025	1.012834552	1.38042915
193782	194822	15	-	YOL073C	ORF-T	ORF-T	-0.517033639	-0.340778821	0.430144675	0.272788067	0.337592073
197174	198742	15	-	NBA1	ORF-T	ORF-T	-0.077560184	0.108760548	0.049356982	-0.152181477	0.845389091
200158	202534	15	-	HST1, RTG1	ORF-T	ORF-T	0.067140595	-0.605016052	-0.457320201	-0.028140042	-0.174116184
202654	205902	15	-	RIB2	ORF-T	ORF-T	0.079596562	-0.128899466	-0.617719031	-0.334265216	-0.061622932
204622	205902	15	-	unknown	no_ovlp2	SUTs	-0.336399283	-0.500240363	0.363652924	-0.113808023	0.561093963
206006	207206	15	-	MET22	ORF-T	ORF-T	-0.037681627	-0.368961784	-0.953007076	-0.258282396	-0.913701514
207350	210334	15	-	CRT10	ORF-T	ORF-T	0.24365817	-0.321121848	-0.296120408	0.029222253	-0.065824684
210470	212014	15	-	APM4	ORF-T	ORF-T	0.18960928	-0.230533635	-0.27619067	-0.272452541	-0.184153637
216190	216750	15	-	MAM3	ORF-T	ORF-T	0.064791593	-0.638371435	0.02954523	-0.032315574	-1.302988655
219150	220526	15	-	SUT768	SUTs	SUTs	1.100953135	0.36737055	0.239326612	0.104292228	0.811417401
224406	226478	15	-	THI20	ORF-T	ORF-T	-0.219617301	0.20526532	0.520886406	0.408263375	-0.488839596
231302	231782	15	-	DDR2	ORF-T	ORF-T	-1.463531558	-2.375451746	1.398118489	0.995847999	-0.036083695
232326	233694	15	-	SPE2	ORF-T	ORF-T	0.054589519	-0.369348338	-0.103748129	0.14066196	0.061058167
234246	234494	15	-	CUT831	CUTs	CUTs	2.85901911	0.244577852	-0.226335316	-0.414041655	0.28591993
240102	241350	15	-	YOL048C	ORF-T	ORF-T	-0.185398611	-0.018713337	1.223267756	0.401485654	0.128208944
241350	243094	15	-	unknown	no_ovlp	SUTs	-0.136178316	0.506069321	1.076161452	0.011571461	0.163496841
248350	249606	15	-	NTG2	ORF-T	ORF-T	-0.09076359	-0.228457213	0.223051107	-0.094578408	-0.564513783
251158	252694	15	-	NOP12	ORF-T	ORF-T	0.271351256	-0.354366563	-0.557026251	-0.128710748	0.149833171
253086	253630	15	-	RPS15	ORF-T	ORF-T	-0.326614186	0.021024265	-0.122798129	-0.199068495	0.061176115
253630	254918	15	-	unknown	no_ovlp2	SUTs	0.279880716	0.59031265	-0.401743368	-0.142522623	0.104200425
254918	256118	15	-	YOL038C-A	ORF-T	ORF-T	0.403836055	-0.094963474	-0.039738781	-0.463257287	0.323880176
255094	256118	15	-	unknown	no_ovlp2	SUTs	0.385138129	0.032327716	-0.548135082	-0.306210794	0.735151625
264654	265174	15	-	CUT832	CUTs	CUTs	2.686210383	0.540258199	-0.098804112	0.536146146	-0.280472437
267566	267950	15	-	SIL1	ORF-T	ORF-T	1.632671819	-0.094749844	0.045116725	-0.147353013	0.010072784
269774	271582	15	-	YOL029C, YA	other	other	0.535537252	-0.288878105	-0.75672993	-0.313960892	-0.157740705
271830	273782	15	-	MDM38	ORF-T	ORF-T	-0.20316617	0.008643145	-0.089737369	-0.084398863	0.046218319
273926	274390	15	-	MIM1	ORF-T	ORF-T	0.02392197	0.010435974	0.644489751	0.263493419	0.383566388

280174	281526	15 -	YOL022C	ORF-T	ORF-T	0.397566693	0.230424358	-0.006780732	0.005741931	0.156232962
282310	285462	15 -	DIS3	ORF-T	ORF-T	0.147574275	-0.337363373	-0.55611932	0.023055962	-0.110530546
290726	292126	15 -	TLG2	ORF-T	ORF-T	0.017465124	-0.516362284	0.346769973	-0.234280445	0.683365963
294662	296134	15 -	CMK2	ORF-T	ORF-T	-0.077034323	-1.025104527	-0.562657153	0.282149818	-0.789359876
301334	303198	15 -	HRD1	ORF-T	ORF-T	-0.481155564	-0.273568762	0.192233642	0.216882304	0.204067784
303382	304110	15 -	HTZ1	ORF-T	ORF-T	-0.779673816	-0.889973253	-0.236505807	-0.110908819	0.414732347
304110	304886	15 -	unknown	no_ovlp	SUTs	0.459929219	0.418983592	-0.00394785	-0.666328748	-0.463996648
309270	310166	15 -	MDM12	ORF-T	ORF-T	0.127478576	-0.123660243	0.564800749	-0.180258545	-0.199124647
310990	312462	15 -	CSI2	ORF-T	ORF-T	-0.230069774	-0.338378318	-0.259860951	-0.666987276	0.408383738
312926	315494	15 -	TOP1	ORF-T	ORF-T	0.058532562	-0.213931808	-0.386017727	-0.325763782	0.195420538
315750	316214	15 -	RPB11	ORF-T	ORF-T	-0.096028412	-0.443710612	-0.244149438	-0.236448581	0.038711978
321742	323006	15 -	PFA4	ORF-T	ORF-T	-0.367659227	-0.372959029	-0.194041114	-0.133206895	-0.044342347
324366	324630	15 -	IZH2	ORF-T	ORF-T	1.663097772	-0.087855273	-0.152871224	-0.287319885	-0.328826069
326150	326430	15 -	SUT769	SUTs	SUTs	0.550017837	-1.071490657	0.120493018	-0.46440768	0.247219495
332286	333390	15 -	CUT833	CUTs	CUTs	1.654105341	-0.243769222	-0.454447707	-0.452174952	0.483216048
335878	337598	15 -	DNL4	ORF-T	ORF-T	-1.191623952	0.087161727	1.746445667	-0.241147259	0.06441049
337382	337598	15 -	unknown	no_ovlp2	SUTs	0.118893619	-0.079063783	0.288539891	0.477864899	-0.232302982
337598	338766	15 -	YOR006C	ORF-T	ORF-T	0.635214194	-0.348511659	-0.715923913	-0.373723511	-0.070330224
338766	340046	15 -	SGT2	ORF-T	ORF-T	0.735350137	0.115710514	-0.118246275	-0.125259258	-0.56349832
340998	342638	15 -	SLG1	ORF-T	ORF-T	-0.080915656	-0.764972717	-0.295849776	0.172686809	-0.287910206
342638	343430	15 -	unknown	no_ovlp	SUTs	0.712599678	0.401689188	-0.159346794	0.670705713	-0.51316952
346326	347294	15 -	TIR2	ORF-T	ORF-T	-0.379390638	-1.137308105	-0.297301458	0.948728845	-0.106079692
351118	354118	15 -	unknown	no_ovlp	SUTs	0.905514052	0.301132984	-0.858869706	-1.106616487	-0.892166253
356190	357214	15 -	SUT772	SUTs	SUTs	1.205534376	-0.97504045	-0.436141415	-0.435502057	-0.077736867
360310	361198	15 -	ERP4	ORF-T	ORF-T	-0.121910258	-0.511793699	0.099105072	-0.286184847	0.540040014
363566	364134	15 -	CUT834	CUTs	CUTs	2.423273426	0.423231805	0.694697912	0.397421815	-0.87839733
370438	370878	15 -	HSP10	ORF-T	ORF-T	-0.660186682	-0.34565031	0.618793333	0.354792688	0.330543575
370878	371390	15 -	SUT774	SUTs	SUTs	1.815265516	0.113872259	0.998858044	0.32923284	2.092365431
372670	373494	15 -	YOR021C	ORF-T	ORF-T	0.210810348	-0.474384045	-0.481937845	-0.291737458	0.309485775
373774	375878	15 -	YOR022C	ORF-T	ORF-T	-0.017729462	-0.057840069	0.37556274	-0.005439909	0.046295773
376014	377814	15 -	AHC1	ORF-T	ORF-T	-0.165048648	-0.182945964	0.462095072	0.223594485	0.218961415
380142	380702	15 -	CUT836	CUTs	CUTs	2.750992972	-0.733647975	-0.824456764	-0.33381523	-0.121694136
383294	384646	15 -	CIN5	ORF-T	ORF-T	-1.24043414	-2.081284017	1.560589455	1.365526189	0.369202527
389678	391334	15 -	HMS1	ORF-T	ORF-T	0.007124062	-1.496012851	-0.539074186	0.315100531	-0.84970416
392358	394766	15 -	unknown	no_ovlp2	SUTs	0.181977795	-0.381792647	-0.297240851	-0.246747194	0.049134276
394766	397638	15 -	AKR2	ORF-T	ORF-T	-0.197743061	-0.687623317	-0.236608458	0.284863796	0.191150232
397366	397638	15 -	unknown	no_ovlp2	SUTs	0.162395641	-0.293156304	-0.257042332	-0.506448196	0.044641969
397638	400110	15 -	YOR034C-A,	other	other	0.182401807	-0.377591156	0.104189257	0.099884288	-0.308778073
400110	401374	15 -	SUT776	SUTs	SUTs	-0.00777175	-0.778325161	-0.454669608	-0.16374903	0.240950935
402750	405430	15 -	HIR2	ORF-T	ORF-T	-0.212952495	-0.737647867	-0.379179947	-0.120355986	0.0263082
406982	407614	15 -	CUT839	CUTs	CUTs	2.568083913	-1.070407163	-1.578543925	-0.518939062	-0.75499809
407790	408126	15 -	SNR9	other	other	3.72696432	-0.91490898	-0.250969843	-0.032759642	-0.15193952
409190	409878	15 -	CUT841	CUTs	CUTs	5.365624566	-0.476161128	-1.430987856	-0.471737801	-0.661421567
412014	412390	15 -	unknown	no_ovlp	CUTs	1.603115602	-0.082975482	0.439886029	1.380906517	1.389261906
414406	415950	15 -	DBP5	ORF-T	ORF-T	0.023605259	-0.345172593	-0.24389445	0.033324997	-0.04171972
416190	417838	15 -	STD1	ORF-T	ORF-T	-0.077335544	-0.590261955	-0.506172931	-0.029077325	-0.25933065
418582	421702	15 -	RAT1	ORF-T	ORF-T	-0.115580362	-0.369323893	-0.486696051	-0.028403317	0.068034178
421886	422526	15 -	unknown	no_ovlp2	SUTs	0.676689876	0.046510056	0.130035263	-0.082331609	-0.745022415
422526	424646	15 -	RSB1	ORF-T	ORF-T	-0.241700501	-0.109441452	1.1265281	1.125416999	0.371306983
423942	424646	15 -	unknown	no_ovlp2	CUTs	1.400622332	0.108426953	-1.122773325	-0.217403964	-0.306954756
424798	426126	15 -	YOR051C	ORF-T	ORF-T	0.04708371	-0.373360637	-0.832310436	-0.152990783	-0.185421962
426518	427366	15 -	YOR052C	ORF-T	ORF-T	0.061730963	-0.653452495	0.455263093	0.158391413	0.085724871
427838	430006	15 -	VHS3	ORF-T	ORF-T	-0.430373432	-0.453919606	0.412905465	0.258124375	0.203419983
430198	431710	15 -	NOB1	ORF-T	ORF-T	0.365545221	-0.074747062	-0.137395487	0.066280713	0.562101975
434622	436486	15 -	ASE1	ORF-T	ORF-T	0.160873685	-0.162224421	-0.309779548	-0.325297522	0.459916678
438862	441190	15 -	YOR059C, YC	ORF-T	ORF-T	0.460983984	-0.724152594	-0.358895653	-0.094971553	-0.180172987
442646	443606	15 -	YOR062C	ORF-T	ORF-T	-0.793107607	-0.987062259	0.735109053	0.409816679	0.28284913
443606	444222	15 -	unknown	no_ovlp	CUTs	1.215515964	0.005459528	0.34389756	0.084958808	-1.140731328
445998	446766	15 -	YNG1	ORF-T	ORF-T	0.414518763	0.010011571	0.185784436	-0.518062007	0.453085305
451702	453518	15 -	ALG8	ORF-T	ORF-T	-0.336716163	-0.512835333	-0.803691998	-0.037190686	-0.246174972
455894	457958	15 -	GYP1	ORF-T	ORF-T	-0.050500873	-0.412439216	0.168437383	0.101293515	-0.089588501
458758	461718	15 -	THI71	ORF-T	ORF-T	0.515888686	0.459254344	0.048748516	0.339572006	-3.291779184
461718	462494	15 -	unknown	no_ovlp	CUTs	1.153152484	1.827296699	0.380721896	0.053611003	-1.406076539
463654	463982	15 -	unknown	no_ovlp	CUTs	2.396697438	0.855906985	0.149091104	0.273772198	-2.388217979
466622	467638	15 -	CDC21	ORF-T	ORF-T	-0.720791415	-0.576990008	-0.204122848	-0.044026168	0.270092322
469302	471662	15 -	SKI7	ORF-T	ORF-T	0.011542095	-0.419771072	-0.080113371	-0.149880536	0.078515513
473406	474446	15 -	ATX2	ORF-T	ORF-T	0.392923962	-0.094481342	-0.882061905	-0.543338288	-0.326461185
476822	479214	15 -	TGL5, YOR08	other	other	0.037067356	0.025197495	-0.189753087	0.435770367	0.381295992
479214	481150	15 -	unknown	no_ovlp	SUTs	-0.195388089	0.511338841	0.536826805	0.065556902	0.192129896
483118	486974	15 -	TCB1	ORF-T	ORF-T	-0.330173569	-0.347155568	-0.114380443	0.038213445	0.165568876
489942	490886	15 -	VPS21	ORF-T	ORF-T	-0.064049844	-0.91271252	-0.158103958	0.128110952	-0.201745492
491094	493014	15 -	PTC5	ORF-T	ORF-T	0.23419165	0.030706716	-0.00743228	0.09918935	-0.042067572
493686	494550	15 -	CUT842	CUTs	CUTs	2.292926777	0.156904791	-0.73062077	-0.315692761	0.085726499
497350	502486	15 -	YOR093C	ORF-T	ORF-T	-0.140683281	-0.551743846	-0.436674602	-0.307318322	-0.064908041
503430	504494	15 -	RKI1	ORF-T	ORF-T	0.189800886	-0.46271859	-0.580463113	-0.21873763	0.337644914
506926	507558	15 -	YOR097C	ORF-T	ORF-T	-0.362961814	-0.478393336	0.55305918	-0.16629187	0.592930353
509966	511254	15 -	NUP1	ORF-T	ORF-T	0.042249021	0.160419813	-0.345076932	-0.199077062	0.288251333
516894	517206	15 -	OST2	ORF-T	ORF-T	0.633383783	-0.42294853	0.003763077	-1.098000215	-0.185591398
517206	518478	15 -	unknown	no_ovlp	CUTs	1.244438148	0.108024798	-0.574075736	0.162335174	1.399172408
519854	520422	15 -	CUT843	CUTs	CUTs	3.130721535	-0.271190297	-0.94849125	-0.536035278	-0.612556566
521494	522630	15 -	SUT779	SUTs	SUTs	1.776093049	-1.163331367	-0.822266623	-0.205104641	-0.27509306
530502	531262	15 -	CUT844	CUTs	CUTs	2.41235808	0.243442822	-0.453277745	-0.374454441	0.509351758

538574	539510	15	-	TRS33	ORF-T	ORF-T	0.797271613	-0.361805677	-0.497819727	-0.39763617	-0.196580959
539662	544414	15	-	RPO31	ORF-T	ORF-T	0.195311682	-0.188392042	-0.706465592	-0.024251228	0.065418262
548038	550278	15	-	RIO1	ORF-T	ORF-T	0.413261687	-0.355861981	-0.252862408	-0.19605698	-0.084446087
552662	552886	15	-	YOR121C, PF	other	other	0.647075515	-0.361736967	-0.088692296	-0.287407985	-0.214259109
553118	554646	15	-	LEO1	ORF-T	ORF-T	-0.309112269	-0.784938865	-0.296107807	0.020209547	-0.263466586
554758	558750	15	-	UBP2	ORF-T	ORF-T	-0.647996746	-0.538279518	-0.11690664	-0.053871641	-0.124297317
558894	559798	15	-	CAT5	ORF-T	ORF-T	-0.141268366	-0.193045513	0.441873803	0.138414178	0.042286642
559870	560718	15	-	IAH1	ORF-T	ORF-T	-0.617016275	-0.245107442	0.389318672	-0.18511767	0.796942128
564414	566222	15	-	ADE2	ORF-T	ORF-T	0.773883132	-0.075382563	-0.446796875	-0.228488975	-0.812528812
566686	569854	15	-	YOR129C	ORF-T	ORF-T	0.048904394	0.161038052	-0.232627823	-0.48695364	0.562615009
569630	569854	15	-	unknown	no_ovlp2	SUTs	0.205229578	-0.137434553	-1.058089834	-0.169475247	-1.048103956
569854	570878	15	-	ORT1	ORF-T	ORF-T	0.131400814	-0.85817533	-1.242962192	-0.343605712	-1.698736583
572166	572926	15	-	YOR131C	ORF-T	ORF-T	-0.152010553	-0.794459383	0.014244652	-0.485508771	0.108054038
574142	574790	15	-	CUT848	CUTs	CUTs	3.381809435	0.04558331	-1.4255114	0.485034072	0.687468782
578542	579870	15	-	SUT782	SUTs	SUTs	0.877101086	-0.602364977	-0.719482487	-0.809381587	-0.376954049
583446	584038	15	-	SIA1	ORF-T	ORF-T	0.159732199	-0.712747588	-0.49931183	-0.145968458	-0.870073506
584246	586390	15	-	RUP1	ORF-T	ORF-T	-0.127565063	-0.463691841	-0.006013846	0.079292207	-0.052033764
589710	592630	15	-	ARP8	ORF-T	ORF-T	0.036153418	-0.117977594	-0.093292288	-0.025395278	0.096782301
601398	602406	15	-	THI80	ORF-T	ORF-T	0.315607053	-0.372724179	-0.61615514	-0.713498833	-0.159215592
602606	605286	15	-	ELG1	ORF-T	ORF-T	0.029060818	-0.370218852	-0.251228502	-0.57542257	0.558960697
605286	606182	15	-	PNO1	ORF-T	ORF-T	0.662141794	-0.228324833	-0.561803299	-0.245322264	0.124877686
608518	609214	15	-	SPP2	ORF-T	ORF-T	0.262521333	-0.474821028	0.17837292	-0.211346212	0.102954743
609534	611430	15	-	SMP3	ORF-T	ORF-T	0.106577193	0.037538303	0.021371518	0.071334008	-0.498382468
612974	616950	15	-	RPB2	ORF-T	ORF-T	-0.187024382	-0.365645286	-0.420309772	0.107614208	-0.203501383
617374	618454	15	-	YOR152C	ORF-T	ORF-T	0.149845232	-0.221159912	0.744944349	0.525481437	1.458749678
618454	619198	15	-	CUT849	CUTs	CUTs	-0.162284762	0.425391532	-0.288236693	0.99949738	1.267256566
626534	627990	15	-	ISN1	ORF-T	ORF-T	-0.186670608	-0.159468113	0.332000478	0.16673498	0.407838982
628142	630654	15	-	NFI1	ORF-T	ORF-T	-0.111606026	-0.575952082	-0.407329396	-0.185324741	-0.156374157
630854	631822	15	-	PUP1	ORF-T	ORF-T	-0.221234363	-0.388597555	-0.016023329	-0.171392483	-0.222009501
633054	633630	15	-	SME1	ORF-T	ORF-T	0.434517067	-0.235813247	-0.255815911	-0.204256591	0.309112864
636838	638766	15	-	PNS1	ORF-T	ORF-T	-0.528476576	-1.042414342	0.594787172	0.698029962	0.790499678
639406	642126	15	-	YRR1	ORF-T	ORF-T	-0.365030839	-0.50374715	0.336782474	-0.13433699	0.196226775
643166	644342	15	-	YOR164C	ORF-T	ORF-T	-0.132206944	-0.513169171	-0.01057372	-0.041125422	0.286885
646982	648718	15	-	SWT1	ORF-T	ORF-T	-0.008021186	-0.51871307	-0.679141191	-0.626467077	-0.282220826
648718	649030	15	-	RPS28A	ORF-T	ORF-T	-0.449224284	-0.364081927	0.150962653	-0.091451419	0.125536499
651830	653974	15	-	YOR169C, LC	other	other	0.092407314	-0.421845212	0.306481213	0.17598241	0.313495374
656374	658470	15	-	unknown	no_ovlp	SUTs	0.242933161	0.407025459	-0.162231495	0.226034897	0.327293311
661758	662158	15	-	ALE1	ORF-T	ORF-T	-0.348972542	-0.966090914	-0.163350533	0.252273042	-0.398169551
667526	670614	15	-	GAC1	ORF-T	ORF-T	-1.392070676	-1.218060683	1.642288633	2.142573485	-0.821032268
671582	672414	15	-	SYC1	ORF-T	ORF-T	0.204837276	-0.52402562	-0.269202482	-0.202579076	-0.307941811
674174	675670	15	-	DCI1	ORF-T	ORF-T	0.259802306	-1.031020212	-0.094336028	-0.125695641	-0.429253941
676918	677526	15	-	unknown	no_ovlp	SUTs	0.169589194	0.448035107	-0.149716091	0.158419993	0.6126619
678382	678838	15	-	RPS30B	ORF-T	ORF-T	1.158499368	-1.079004149	-0.478128983	-0.919826315	-0.037834902
680430	680870	15	-	unknown	no_ovlp	CUTs	4.601330163	-0.97956073	-1.200865628	-0.598537834	-0.610856912
681398	682166	15	-	GSP2	ORF-T	ORF-T	-1.191505506	-0.502963767	1.51989007	0.537009096	2.007342783
682902	683574	15	-	CUT854	CUTs	CUTs	2.242780782	0.100048068	-0.114890088	-0.265905156	-0.247556479
689158	689366	15	-	SUT783	SUTs	SUTs	1.720383848	-0.118023824	0.511549412	0.713675521	-0.217429018
691550	692182	15	-	SUT785	SUTs	SUTs	0.542445554	-0.372419783	0.386713174	0.318654788	-0.827722937
697910	702134	15	-	THI72	ORF-T	ORF-T	0.203945505	0.413541095	0.154593454	0.027283429	-1.229620819
711670	712598	15	-	TOA1	ORF-T	ORF-T	-0.224149528	-0.440761069	0.050771271	-0.073804132	-0.051832617
715438	716862	15	-	LIP5	ORF-T	ORF-T	0.30310202	-0.222258571	-0.325213195	-0.290939101	0.039936541
718766	720222	15	-	BFR1	ORF-T	ORF-T	-0.605500543	-0.395915122	-0.301346901	-0.052383388	-0.03090585
720398	721718	15	-	MRM1	ORF-T	ORF-T	-0.635859496	-0.487163834	-0.551999276	-0.348951384	-0.391678292
721718	722598	15	-	SUT786	SUTs	SUTs	0.551182033	-0.1369414	-0.531162548	-0.054547356	0.188716606
725518	727238	15	-	FMP38	ORF-T	ORF-T	-0.303132794	-0.432919865	0.0003828	-0.369557394	0.125528995
729822	733502	15	-	RET1	ORF-T	ORF-T	0.468146319	-0.210835006	-0.367469781	0.059131066	-0.067759298
736302	737878	15	-	NPT1	ORF-T	ORF-T	0.68185822	-0.052910544	-0.672022395	-0.285076262	-0.850749749
738814	741766	15	-	MGM1	ORF-T	ORF-T	-0.008764757	-0.362967508	0.116159797	0.044428948	-0.254244544
744278	745350	15	-	SAS5	ORF-T	ORF-T	0.116746511	-0.250743673	0.249046625	0.060162409	0.158923189
745350	746398	15	-	unknown	no_ovlp	SUTs	-0.055430443	0.690654836	0.657745482	0.078371911	0.170792439
746590	747318	15	-	YOR215C	ORF-T	ORF-T	0.139962126	-0.538557161	0.652645119	0.080567989	0.041781155
747430	749038	15	-	RUD3	ORF-T	ORF-T	0.212348005	-0.086098746	-0.378967314	-0.410299661	0.079941588
752734	755062	15	-	STE13	ORF-T	ORF-T	0.389673817	-0.461486277	-0.499323383	0.154590354	-0.464587243
756310	757606	15	-	MCT1	ORF-T	ORF-T	-0.134537035	-0.410706809	0.54021725	-0.054780498	0.242734462
759310	759526	15	-	SNR35	other	other	0.649750788	-0.4435351	0.007663532	-0.14462575	0.149000945
760710	761302	15	-	RPB8	ORF-T	ORF-T	-0.283218651	-0.044570844	-0.255281134	-0.151313538	0.314143991
761470	762126	15	-	ISU2	ORF-T	ORF-T	0.336906423	-0.320696492	-0.135345401	-0.332857769	-0.38435738
766742	768046	15	-	YOR228C	ORF-T	ORF-T	-0.141449371	-0.090650511	0.607818486	0.252370224	0.155413874
769022	769990	15	-	CUT859	CUTs	CUTs	2.174866703	0.199627631	0.061546465	0.360470208	-1.170324575
769990	772222	15	-	unknown	no_ovlp	SUTs	0.277764273	0.305458782	-0.031549819	-0.127607818	0.142510497
778854	779400	15	-	RPL33B	ORF-T	ORF-T	0.199794001	-1.389498793	-1.014370415	-1.17795992	-0.812088181
780854	781542	15	-	CUT861	CUTs	CUTs	2.483596527	0.049516101	-0.868342132	-0.488072941	-0.776890242
784110	784622	15	-	CUT862	CUTs	CUTs	3.204467739	0.010533767	-0.132830944	-0.093456377	-0.168467452
789902	792262	15	-	PUS7	ORF-T	ORF-T	0.016697453	-0.337558409	-0.789670612	-0.099154054	-0.060077279
793966	795542	15	-	DGA1	ORF-T	ORF-T	-0.256637416	-0.330037281	0.23801863	0.136510865	0.03015186
795686	796870	15	-	YOR246C	ORF-T	ORF-T	-0.18444677	-0.58290421	-0.629909627	-0.301868358	0.02141394
798630	800734	15	-	APC5	ORF-T	ORF-T	0.336713287	-0.098111161	0.160334824	-0.167580541	-0.491742937
800894	802334	15	-	CLP1	ORF-T	ORF-T	0.244853509	-0.589100552	-0.120548059	-0.186193606	-0.084927344
802470	803486	15	-	YOR251C	ORF-T	ORF-T	0.291709258	-0.085287867	0.124433812	-0.123864724	0.021762366
804943	807063	15	-	SEC63	ORF-T	ORF-T	-0.359923015	-0.650355474	-0.751870361	-0.090072372	-0.379483872
808223	810807	15	-	TRE2	ORF-T	ORF-T	-0.064202621	-0.497498299	-0.114348886	0.058325935	-0.501467187
812319	813751	15	-	RPT4	ORF-T	ORF-T	-0.244223168	-0.303756445	0.243590083	0.040738693	-0.091727468

815815	817047	15	-	RPN8	ORF-T	ORF-T	-0.128170635	-0.527176995	0.156021248	-0.152839449	0.140108815
817935	818367	15	-	unknown	no_ovlp	CUTs	2.840684062	-0.107120662	-0.896051529	-0.752077225	-0.164720137
820319	820767	15	-	unknown	no_ovlp	CUTs	1.611311092	0.4024718	0.010700118	0.085840291	-1.021028269
822399	825455	15	-	HRK1	ORF-T	ORF-T	-0.019070477	-0.374068768	-0.368636723	-0.223827394	-0.819418029
827911	830743	15	-	VPH1	ORF-T	ORF-T	0.043075249	-0.153738357	-0.232800317	-0.07910319	-0.303095338
831047	832103	15	-	FSF1	ORF-T	ORF-T	0.033976898	-0.23857382	-0.879443086	-0.362692415	-0.09626812
834343	836503	15	-	TPO4	ORF-T	ORF-T	-0.310089704	-0.701189372	-0.720511635	-0.035275643	-0.82582863
836503	837039	15	-	SUT791	SUTs	SUTs	-0.923124343	-0.370541067	1.246360307	0.654546155	-0.450612647
838975	841087	15	-	RIM20	ORF-T	ORF-T	0.046952884	-0.320298783	-0.13641291	-0.273107943	-0.155390487
841911	842183	15	-	SNR31	other	other	0.184165618	-0.284697204	-0.122216327	0.005993091	-0.139407662
843583	844743	15	-	RFM1	ORF-T	ORF-T	0.044761861	-0.623688352	-0.167147413	-0.069905343	-0.548782803
844879	845847	15	-	FSH3	ORF-T	ORF-T	-0.655557533	-0.858106551	0.620857919	0.399808405	-0.673455
846175	847183	15	-	PLP2	ORF-T	ORF-T	-0.04141548	-0.850377096	-0.282587486	-0.291391148	0.024189243
848919	849399	15	-	unknown	no_ovlp	CUTs	1.583130335	-1.192792611	0.20647307	0.146839815	0.488229767
850935	851847	15	-	YOR287C	ORF-T	ORF-T	0.58841979	0.087255809	-0.579935688	-0.112482129	0.578487024
852039	853055	15	-	MPD1	ORF-T	ORF-T	-1.110479641	-0.663568577	-0.178595332	0.206830337	0.456986761
853055	853895	15	-	unknown	no_ovlp	SUTs	0.576801635	0.213870988	-0.829463068	-0.331999082	-0.303564772
855047	860375	15	-	SNF2	ORF-T	ORF-T	-0.00515805	-0.290838717	-0.271786426	0.166478082	-0.169209702
865615	866575	15	-	YOR292C	ORF-T	ORF-T	0.223573151	-0.483395393	0.811284199	0.077000545	0.420269056
868951	869927	15	-	unknown	no_ovlp	CUTs	1.505731978	-0.181516914	-0.513976763	-0.439790324	-0.135981667
874647	875351	15	-	TIM18	ORF-T	ORF-T	-0.156292908	-0.312003716	-0.191584653	-0.107084715	-0.227769455
876975	877759	15	-	MBF1	ORF-T	ORF-T	0.340937751	-0.634822898	0.215015606	-0.083883863	-0.532209393
880271	880631	15	-	unknown	no_ovlp	CUTs	1.857290377	-0.231182621	-0.652338423	-0.363425185	0.730925992
880631	882439	15	-	SUT793	SUTs	SUTs	0.145657766	0.41689582	-0.142934069	0.008017671	0.161329072
888183	888775	15	-	YOR304C-A	ORF-T	ORF-T	-0.113538237	-0.653958651	0.243406792	-0.295776567	0.641045814
889791	891495	15	-	MCH5	ORF-T	ORF-T	-0.346529784	-0.418046004	-0.331197352	0.336434027	-0.546611973
892679	894239	15	-	SLY41	ORF-T	ORF-T	0.034738775	-0.675245843	-0.300033741	0.108454947	-0.302653153
894239	896407	15	-	SNU66	ORF-T	ORF-T	0.20973637	-0.237183656	-0.151903536	-0.364313857	0.247076178
896599	898391	15	-	YOR309C, NC	ORF-T	ORF-T	0.15340252	-0.333207427	-0.703083097	-0.108960751	-0.409284998
898847	899975	15	-	HSD1	ORF-T	ORF-T	0.285206332	0.133307065	-0.405432598	-0.303529243	-0.164851447
900759	901263	15	-	RPL20B	ORF-T	ORF-T	0.907867271	-0.402848028	-0.766005725	-0.868645141	-0.084952332
906103	907695	15	-	COT1	ORF-T	ORF-T	0.813860474	0.122198241	-0.545686457	0.259330207	-1.060598164
907695	909119	15	-	YOR316C-A	ORF-T	ORF-T	-0.069044838	0.096457122	0.903559956	0.498230058	-0.288008172
908239	909119	15	-	unknown	no_ovlp2	SUTs	0.97157439	0.161592297	0.188243968	-0.447783783	-0.073217581
911663	912311	15	-	YOR318C	other	other	1.73008663	0.457648435	-0.417130732	-0.56975235	-1.60003338
913503	915207	15	-	GNT1	ORF-T	ORF-T	-0.254190222	-0.481009026	-0.393061157	-0.103460158	0.087059622
918447	921303	15	-	LDB19	ORF-T	ORF-T	-0.037233084	-0.055460687	-0.146284219	0.128522676	0.010325552
921439	922927	15	-	PRO2	ORF-T	ORF-T	0.053364364	-0.753153014	-0.56148151	-0.164149601	-0.577460577
923095	925183	15	-	FRT1	ORF-T	ORF-T	-0.252316735	-0.596271275	-0.052217514	-0.031369706	0.0626021
930591	931207	15	-	SNC2	ORF-T	ORF-T	-0.216280714	-0.623788863	-0.195316022	0.108987814	-0.164659518
938231	939423	15	-	SCD5	ORF-T	ORF-T	0.197799215	-0.29981253	-0.27923294	-0.014431857	-0.552223278
939591	943399	15	-	MIP1	ORF-T	ORF-T	0.147869218	-0.468108905	-0.58634159	0.055564885	-0.039343123
944103	944399	15	-	CUT868	CUTs	CUTs	3.283590345	0.739637314	-2.255491886	-0.308010223	0.352484062
946015	949223	15	-	ALA1	ORF-T	ORF-T	-0.274053468	-0.544286585	-0.918550645	0.076206539	-0.353037962
953455	953991	15	-	CUT869	CUTs	CUTs	3.889490998	-0.403819095	-1.05963045	-0.203049202	-0.878758258
956807	958199	15	-	unknown	no_ovlp2	SUTs	0.375486545	0.330559146	0.93992934	-0.093400209	-0.371675849
958199	959047	15	-	UBC11	ORF-T	ORF-T	-0.276934154	0.50828271	0.330161964	0.434485994	0.532034695
959047	960239	15	-	RPA43	ORF-T	ORF-T	0.474801403	-0.572475702	-0.707583771	-0.04771913	-0.185448296
960239	960671	15	-	CUT871	CUTs	CUTs	2.564310081	0.320039362	-1.321329538	-1.302681586	-2.378228494
966479	967775	15	-	YOR342C	ORF-T	ORF-T	0.05279527	-0.925706681	-0.815614581	-0.256361914	-0.302541915
967775	969583	15	-	YOR343C	other	other	0.394060406	0.945643493	1.203914823	0.961136276	-0.796364514
968647	969583	15	-	unknown	no_ovlp2	SUTs	0.722180749	0.558608934	0.728141177	0.786258039	-0.404080368
969583	970015	15	-	SUT795	SUTs	SUTs	0.722932361	-0.030378449	-0.037288312	-0.140128976	-0.765759098
976607	978311	15	-	TYE7	ORF-T	ORF-T	0.734599846	-0.335601283	-0.230091242	-0.248009668	-0.700155939
980967	981423	15	-	SUT796	SUTs	SUTs	-0.089386432	-0.771427384	0.622568678	-0.221004441	0.01794582
985127	986519	15	-	PYK2	ORF-T	ORF-T	0.821946037	0.801488562	0.164182393	0.536006696	-2.637696884
986719	988799	15	-	PUT4	ORF-T	ORF-T	-0.114770672	-0.443021621	-0.133128916	0.332960336	-0.634121287
992431	992743	15	-	unknown	no_ovlp2	SUTs	0.970145083	0.152388364	0.005610099	-0.175248808	0.335172284
992743	994871	15	-	MNE1	ORF-T	ORF-T	0.155597623	-0.304663489	-0.364299861	-0.391778089	-0.202493748
998311	1000871	15	-	SOG2	ORF-T	ORF-T	0.090194947	-0.596007807	0.093939103	-0.028464851	-0.169852027
1001023	1003247	15	-	MSC6	ORF-T	ORF-T	-1.010927657	-0.964141985	0.010397518	-0.061205801	0.083528544
1003247	1004415	15	-	CUT873	CUTs	CUTs	0.900510631	0.230213876	-0.198287657	-0.755982716	-0.678778264
1009207	1009727	15	-	SNX3	ORF-T	ORF-T	0.110347887	-0.887512631	0.064066563	-0.520176598	0.099349344
1010335	1010791	15	-	CUT874	CUTs	CUTs	3.140213425	0.042883562	-1.549046306	-0.390654137	0.466070593
1013135	1015047	15	-	PDE2	ORF-T	ORF-T	0.022016126	-0.635585766	-0.426674898	-0.302011487	-0.365057919
1015263	1017575	15	-	PRT1	ORF-T	ORF-T	-0.196660564	-0.429498492	-0.441998752	-0.110861459	-0.213810237
1017719	1018799	15	-	PRE10	ORF-T	ORF-T	0.063167702	-0.311909273	0.032096855	-0.156927776	0.034874385
1019887	1023319	15	-	PIP2, YOR36[illegible]	other	other	-0.51550771	-0.654105482	0.165585107	0.06667533	-0.23115474
1026791	1027983	15	-	SUT800	SUTs	SUTs	-0.033267363	-1.78191955	-0.993444734	-0.600734387	0.187524615
1028095	1028687	15	-	RPS12	ORF-T	ORF-T	-0.461113773	-0.219857458	-0.030711785	-0.08625507	-0.035341615
1028999	1031319	15	-	MRS6	ORF-T	ORF-T	-0.098658091	-0.486117599	-0.382532599	-0.118889387	0.022632804
1033511	1034287	15	-	GPB1	ORF-T	ORF-T	-0.222265771	-0.606397262	-0.022431968	0.053319103	-0.154492691
1035103	1036543	15	-	NDD1	ORF-T	ORF-T	-0.116935809	-0.295109664	0.521122674	0.185873696	0.360059286
1038319	1039447	15	-	CUT875	CUTs	CUTs	0.439691328	0.162760916	1.729670987	2.306230786	1.34460144
1041543	1043111	15	-	GDH1	ORF-T	ORF-T	-0.882821617	0.045794873	0.03348615	0.192360182	0.21382982
1043111	1044455	15	-	unknown	no_ovlp	CUTs	1.102074275	0.407565065	0.398527818	0.574901725	-0.63144415
1047935	1050855	15	-	YOR379C	other	other	-0.167470976	-0.231175568	-0.116050102	-0.099392288	0.137477841
1050855	1052239	15	-	unknown	no_ovlp	SUTs	0.042650824	0.473958931	0.239062823	0.101965787	0.21703695
1060239	1064399	15	-	SUT802	SUTs	SUTs	0.095682001	-0.50130678	-0.078486964	0.061914579	-0.495993776
7089	8233	15	+	BDS1	ORF-T	ORF-T	0.166474517	-0.413621071	-0.508391779	-0.637871659	-1.671722635
15761	16809	15	+	SUT348	SUTs	SUTs	1.506813177	0.093907379	0.450921386	0.30871811	-2.111583527
18121	18737	15	+	unknown	no_ovlp	SUTs	-0.349949652	-0.165314215	1.773571444	0.909217704	-2.121521265

21865	22353	15	+	SUT349	SUTs	SUTs	2.335645422	-0.626466321	0.01491157	0.44339045	-1.991046018
28833	32777	15	+	unknown	no_ovlp	SUTs	0.632877746	-0.023229334	-0.565820514	-0.413575741	-1.596287975
34633	35481	15	+	ZPS1	ORF-T	ORF-T	1.294218501	-2.072597595	-3.161342089	-1.181761774	1.15982855
38857	39281	15	+	unknown	no_ovlp	CUTs	1.673717125	-0.441941159	-0.037153849	0.371797939	-0.62087447
40673	43682	15	+	FRE7	ORF-T	ORF-T	0.07066006	1.356023508	0.474552323	-0.138644299	-0.072862125
42690	43682	15	+	unknown	no_ovlp2	SUTs	-0.184987825	0.458201693	0.29977262	0.197733418	0.043755486
43682	44730	15	+	GRE2	ORF-T	ORF-T	-0.496530094	-0.51379756	-0.025893537	-0.067374422	1.049646438
44906	45746	15	+	DCP1	ORF-T	ORF-T	0.123333863	-0.619022212	-0.758766352	-0.245562752	-0.099647984
48874	49442	15	+	PSF3	ORF-T	ORF-T	0.279288984	-0.255937961	-0.209213369	-0.013646619	0.176086571
53066	54594	15	+	NOP8	ORF-T	ORF-T	0.119841027	-0.301438533	-0.296096617	0.190724409	0.591167919
55530	56338	15	+	RRP40	ORF-T	ORF-T	0.089484244	-0.149012589	-0.297210091	-0.230202459	0.22671372
56338	58754	15	+	PPM2	ORF-T	ORF-T	0.342955007	-0.000476199	-0.812340733	0.034656011	0.446082095
58754	60042	15	+	ARG8	ORF-T	ORF-T	-0.62966084	-1.28048852	-0.726007106	-0.081281196	-0.598195246
61346	62098	15	+	unknown	no_ovlp	CUTs	1.786954461	0.630213764	-1.19107711	-0.27580404	0.643506187
65578	67338	15	+	BSC6	ORF-T	ORF-T	0.369072333	-0.227700695	-0.573178907	-0.21353413	-0.403308635
69370	70322	15	+	unknown	no_ovlp	SUTs	0.744421111	0.25767836	0.366763115	0.789317195	1.024891157
70322	71090	15	+	HRT1	ORF-T	ORF-T	-0.390868746	-0.718386626	0.28052164	-0.169623378	-0.163848344
74130	77354	15	+	ALR1	ORF-T	ORF-T	0.450470689	-0.039605341	-0.314756731	0.291489933	0.067960618
77554	78258	15	+	VPS68	ORF-T	ORF-T	-0.675514202	-0.395953167	0.141202691	-0.035256256	0.539527102
80306	80778	15	+	RPL25	ORF-T	ORF-T	1.392858237	-0.955551846	-1.894481945	-1.588652471	-0.455081706
81386	83578	15	+	unknown	no_ovlp	SUTs	-0.267692424	0.290318358	0.086613381	0.020096701	0.327641831
83578	85258	15	+	TRM13	ORF-T	ORF-T	0.672564186	0.161421528	-0.438616213	-0.299132876	0.174067972
87474	89578	15	+	HRP1	ORF-T	ORF-T	0.314469661	-0.298047654	-0.627841518	-0.078692446	-0.110125805
90058	91546	15	+	unknown	no_ovlp	CUTs	1.386026003	0.117470394	-1.725351737	-0.995108683	-0.475828962
96658	97330	15	+	SUT355	SUTs	SUTs	1.631151692	-0.761023509	0.198400581	-0.153610862	-0.431010737
97330	101218	15	+	RRI2	ORF-T	ORF-T	0.522128198	0.029882601	0.192863802	-0.117492062	0.07258288
99690	101218	15	+	unknown	no_ovlp2	SUTs	0.107672646	-0.295766676	-0.076131569	-0.093232969	0.176666966
101434	103354	15	+	PAP2	ORF-T	ORF-T	-0.01299952	-0.729707026	-0.929751382	-0.271677131	-0.310537676
104242	106426	15	+	SKM1	ORF-T	ORF-T	0.122926027	-0.787516145	0.008159362	0.013043553	-0.015850191
106562	108266	15	+	MSB4	ORF-T	ORF-T	-0.228369562	-0.123980541	-0.042590477	-0.155757731	0.597474841
109170	109978	15	+	SHR5	ORF-T	ORF-T	-0.37581589	-0.073495396	0.525680957	-0.197168252	0.409067426
110226	110866	15	+	ZEO1	ORF-T	ORF-T	-0.327299297	-0.399894814	0.150260139	-0.154898807	-0.145476658
111546	112114	15	+	unknown	no_ovlp	SUTs	0.450857496	0.66333875	-0.172236988	0.275742495	0.130314352
112114	113282	15	+	YOL107W	ORF-T	ORF-T	-0.287098809	-0.541472766	-0.224656242	-0.085281274	-0.274248727
118034	123810	15	+	unknown	no_ovlp	CUTs	1.90754141	0.982098699	-0.752580205	-2.189011343	-2.007733881
123810	125978	15	+	ITR2	ORF-T	ORF-T	0.048112332	-0.372184188	-0.79838563	-0.12424952	-0.3993575
129002	132586	15	+	PKH2	ORF-T	ORF-T	-0.101203564	-0.443434802	0.259820075	-0.026713219	-0.012520835
136074	136946	15	+	CUT357	CUTs	CUTs	1.884968479	-0.156518369	-1.560525757	-0.532683504	-0.425187151
138010	138298	15	+	CUT358	CUTs	CUTs	2.036886617	0.11947855	-0.107843743	-0.653420812	-0.63372127
142794	143818	15	+	TRM10	ORF-T	ORF-T	0.323795773	-0.197712609	-0.568774478	-0.069446472	-0.570086126
144178	145170	15	+	YOL092W	ORF-T	ORF-T	0.392436258	-0.061106686	-0.475233564	0.076000151	0.049547807
147434	150354	15	+	MSH2	ORF-T	ORF-T	-0.305709167	-0.115429762	-0.247141248	-0.13161664	0.170310218
159130	159538	15	+	YOL086W-A	ORF-T	ORF-T	0.184594596	-0.393271665	0.326480014	-0.211034287	0.462853183
161474	162114	15	+	unknown	no_ovlp	CUTs	1.786402656	-0.227107814	-0.509873586	-0.619329139	-0.829110744
162114	165314	15	+	PHM7	ORF-T	ORF-T	-1.595915337	-0.97218632	2.119128458	2.453197998	1.577620092
165314	165610	15	+	unknown	no_ovlp2	SUTs	-0.281348275	0.188377048	0.558034283	2.139601669	0.059211516
165610	167050	15	+	YOL083W	ORF-T	ORF-T	0.212369689	1.04313568	1.644725451	0.711631537	-0.809731397
168354	170082	15	+	ATG19	ORF-T	ORF-T	0.09300574	0.043035615	0.696433917	0.142575717	0.155459578
170306	170818	15	+	CUT362	CUTs	CUTs	1.868330265	0.12957222	0.132925872	-0.209946659	-0.064823219
170818	172858	15	+	IRA2	ORF-T	ORF-T	-0.337186169	-0.626958155	0.375609011	0.668413008	-0.087414042
181690	185426	15	+	AVO1	ORF-T	ORF-T	0.028735834	-0.381101895	-0.314611578	-0.332307094	0.198366739
185426	185746	15	+	ATP19	ORF-T	ORF-T	-0.122873526	-0.253242592	0.056958022	-0.098774147	0.225359571
187010	189578	15	+	MDM20	ORF-T	ORF-T	0.487620318	-0.198269275	-0.800313132	-0.55462965	-0.114435708
191626	194106	15	+	unknown	no_ovlp	CUTs	2.582849542	0.488224844	-0.375015386	-1.700481521	-0.756512091
194962	196402	15	+	THP1	ORF-T	ORF-T	0.155066428	-0.370105811	-0.02683599	-0.432345612	-0.273889932
196514	197170	15	+	EMI5	ORF-T	ORF-T	0.15192848	-0.223909949	0.420889471	-0.129173348	-0.384671505
198938	200442	15	+	NUF2	ORF-T	ORF-T	-0.208320218	-0.29528692	0.051151575	-0.460863716	0.591329316
202050	203586	15	+	unknown	no_ovlp	CUTs	1.794248777	0.263153263	-0.344592046	-0.08474197	0.45985896
212202	213898	15	+	PRS5	ORF-T	ORF-T	-0.167488609	-0.399333018	-0.293502173	-0.117744149	0.042032014
216602	217130	15	+	unknown	no_ovlp	CUTs	1.385093369	0.759535106	0.170492764	-0.442334451	-0.527866249
217130	218730	15	+	GPD2	ORF-T	ORF-T	-0.158135424	-0.748029908	-0.39691257	0.145108466	-0.880525146
219138	220618	15	+	ARG1	ORF-T	ORF-T	-1.092892625	-0.887843178	-0.890682345	-0.257191558	-0.143980061
220730	223098	15	+	YOL057W	ORF-T	ORF-T	-0.415528483	-0.40405083	-0.204781329	-0.037139014	0.184965158
223218	224298	15	+	GPM3	ORF-T	ORF-T	0.663662576	-0.052217436	-1.07227445	-0.352790215	-0.454608154
228570	229946	15	+	PSH1	ORF-T	ORF-T	0.089991347	-0.309585552	0.00788747	-0.086959886	0.332990541
230194	231370	15	+	YOL053W	ORF-T	ORF-T	0.054120948	-0.390017933	0.071761919	0.273949153	0.001290439
234114	234362	15	+	unknown	no_ovlp	CUTs	2.123501765	0.182033293	-0.181628662	-0.125574759	-1.014879203
234554	234762	15	+	unknown	no_ovlp2	CUTs	3.659180353	-0.215445385	-0.440438879	-0.238241148	-0.466783891
234762	238394	15	+	GAL11	ORF-T	ORF-T	-0.019571134	-0.46072234	0.041168788	0.120044507	0.367857666
238578	240218	15	+	GSH2	ORF-T	ORF-T	-0.370427524	-0.495537486	-0.025032543	-0.097184827	-0.232066179
241602	243082	15	+	SUT357	SUTs	SUTs	0.394795156	0.24680483	1.817500328	1.104775476	-0.607963115
243082	246930	15	+	PSK2	ORF-T	ORF-T	0.095686622	-0.292789532	0.084415989	0.040651006	0.258174327
247098	248402	15	+	PEX15	ORF-T	ORF-T	-0.296114363	-0.664839616	0.095257534	0.073584202	0.363322368
249770	250898	15	+	NGL1	ORF-T	ORF-T	0.240403123	-0.481935095	-0.30007078	-0.204456433	-0.68604195
254242	254858	15	+	RPP2A	ORF-T	ORF-T	-0.504185952	-0.770528914	-0.20361055	-0.198544766	-0.328729762
255306	256146	15	+	PRE6	ORF-T	ORF-T	0.145436944	-0.127880017	0.04306981	-0.362132914	0.048677836
256354	258162	15	+	YOL036W	ORF-T	ORF-T	0.272554049	-0.310463958	0.20089567	-0.087337323	-0.017915883
259618	259882	15	+	unknown	no_ovlp	CUTs	5.387624551	0.133780643	-0.529954834	-0.201910579	-0.303414523
259882	265178	15	+	SMC5	ORF-T	ORF-T	-0.231902171	-0.237940059	0.15268647	-0.310719055	0.603834876
263258	265178	15	+	unknown	no_ovlp2	SUTs	-0.812981162	-0.427558515	-0.038477735	-0.255078818	-0.47221333
265346	266250	15	+	OPI10	ORF-T	ORF-T	0.542188278	-0.135806135	0.797628416	0.547486495	-0.504572478
267810	268146	15	+	CUT366	CUTs	CUTs	1.735123266	-0.654248837	0.156952994	-0.161016128	-0.27818278

268146	269842	15	+	GAS5	ORF-T	ORF-T	0.231492115	0.476680008	0.542858593	0.30052074	0.523776536
273978	274674	15	+	unknown	no_ovlp	SUTs	0.486378722	-0.001914606	0.105438624	0.442122405	0.137250689
274674	274906	15	+	unknown	no_ovlp2	CUTs	3.413129669	-0.424711793	-0.840977797	-0.481300947	-0.736038079
274906	276994	15	+	LAG2	ORF-T	ORF-T	-0.204643217	-0.421917314	0.298817054	0.010785313	-0.335040828
278050	280218	15	+	IFM1	ORF-T	ORF-T	-0.461634747	-0.168104594	0.074040345	-0.000552161	-0.434534064
281674	282098	15	+	unknown	no_ovlp	CUTs	2.179020043	0.039551235	0.21652879	-0.819916639	-0.551145012
286042	288066	15	+	TAT2	ORF-T	ORF-T	-0.238494234	-0.529671314	-0.702929751	-0.237856199	-0.424680326
288066	288466	15	+	unknown	no_ovlp2	SUTs	-0.361754848	0.339787795	-0.170649444	0.292881623	0.1652837
288466	288770	15	+	YOL019W-A	ORF-T	ORF-T	0.14189684	0.549240155	0.100660788	-0.024982116	0.433429245
288770	290770	15	+	YOL019W	ORF-T	ORF-T	-0.310975252	-0.48862743	-0.543314238	-0.145330253	-0.075258208
292610	294786	15	+	ESC8	ORF-T	ORF-T	-0.335650433	-0.322654047	0.132498899	-0.308623283	0.27433727
296458	299506	15	+	IRC10	ORF-T	ORF-T	-0.605685455	-0.837457266	0.342941189	0.315325517	-0.81621923
298826	299506	15	+	unknown	no_ovlp2	SUTs	-0.176067246	0.376732151	0.27646393	0.24824522	-0.232292235
299506	300306	15	+	YOL014W	ORF-T	ORF-T	0.980680796	-0.238621286	-0.989630924	-0.104116397	-1.738282199
301098	301338	15	+	YOL013W-A,	ORF-T	ORF-T	-0.727324292	-1.1835498	-0.581328337	-0.790799233	-0.00877742
303434	304618	15	+	SUT358	SUTs	SUTs	0.791675632	0.136297793	-0.191866979	0.672310724	-0.232053393
304618	305354	15	+	PLB3	ORF-T	ORF-T	0.023357599	0.958386071	0.46686789	1.080630045	0.975828297
305354	307698	15	+	PLB3	ORF-T	ORF-T	0.449543747	-1.119810156	-0.968282752	-0.607402884	-0.551002022
307898	309138	15	+	RCL1	ORF-T	ORF-T	0.454309181	0.199869482	-0.586677634	-0.414111454	0.494290924
310306	311010	15	+	COQ10	ORF-T	ORF-T	0.275970937	-0.164872176	0.081102609	-0.422924318	-0.481697296
315818	316322	15	+	CUT368	CUTs	CUTs	2.100339077	0.074794244	-0.746558558	-0.225006735	0.468995814
316730	321666	15	+	SIN3	ORF-T	ORF-T	-0.265031754	-0.356900472	-0.197216135	0.159939842	0.038178019
324786	325746	15	+	PHO80	ORF-T	ORF-T	0.253019884	-0.851123945	-0.133994451	-0.08650359	-0.269208427
326266	326802	15	+	unknown	no_ovlp2	SUTs	0.163524001	0.204268382	0.565449909	-0.222043104	-0.166898894
326802	329026	15	+	RRP6	ORF-T	ORF-T	-4.457152328	-0.511707627	-0.046976621	0.011123333	0.088441743
329098	329386	15	+	unknown	no_ovlp	SUTs	0.414698937	0.129793688	-0.404707464	-0.002673951	-0.168057705
329386	331154	15	+	ALG6	ORF-T	ORF-T	-0.097847634	-0.477309305	-0.354696933	-0.250066367	-0.428979
331458	332330	15	+	YSP3	ORF-T	ORF-T	-0.110072651	-0.097244639	1.396397951	0.570988652	2.210773286
332330	333098	15	+	YSP3	ORF-T	ORF-T	-0.214978607	-0.016557889	0.828547536	0.285811379	1.20039489
333570	334506	15	+	UTP23	ORF-T	ORF-T	0.558831481	-0.157591255	-0.92320048	-0.507469857	0.257179991
335354	337202	15	+	SUT359	SUTs	SUTs	2.527878498	-0.271292032	-1.968972906	-0.109540939	-1.068540803
342890	345938	15	+	YOR008W-B,	other	other	1.501319838	-1.002602161	-1.094417669	-0.292942712	-0.920268009
345690	345938	15	+	unknown	no_ovlp2	CUTs	1.535369536	-1.199370655	-1.712503116	-0.670048535	-1.713879954
349858	353922	15	+	AUS1	ORF-T	ORF-T	-1.103638343	-0.183533628	0.728797767	1.189737371	-1.608369681
356538	357426	15	+	YOR012W, IR	other	other	1.083310101	1.057483928	0.118212202	0.546563292	-1.351505026
357426	360106	15	+	RTS1, YOR01	other	other	-0.231967354	-0.444183531	-0.286328601	-0.139854935	-0.479247789
361386	363986	15	+	PET127	ORF-T	ORF-T	-0.780111692	-0.947880008	-0.147216813	0.097009609	0.169927876
364330	367034	15	+	ROD1	ORF-T	ORF-T	0.253436907	-0.208556728	0.081510705	0.018221369	-0.225772142
367034	367842	15	+	unknown	no_ovlp2	SUTs	0.25492624	0.434309007	0.208688102	0.29548967	-0.103068806
367842	371538	15	+	YOR019W	ORF-T	ORF-T	-1.026174393	-0.355138317	2.549563035	2.287035008	0.657483075
370418	371538	15	+	unknown	no_ovlp2	SUTs	-0.288638853	0.333449294	0.369969092	0.08467828	0.162084922
371538	372162	15	+	YOR020W-A	ORF-T	ORF-T	0.197513756	-0.024426772	1.275963966	0.552253601	0.431639132
373794	374778	15	+	unknown	no_ovlp	CUTs	1.259262696	0.351266059	-0.733320067	1.159810114	-0.906474163
378234	380986	15	+	HST3	ORF-T	ORF-T	-0.28247709	-0.431367303	-0.013771881	-0.243275817	0.56651787
379642	380986	15	+	unknown	no_ovlp2	SUTs	-0.042662063	-0.272282629	-0.167767525	-0.084978791	0.446485049
380986	382946	15	+	STI1	ORF-T	ORF-T	0.008644613	-0.181873617	0.073760902	0.018161135	-0.535725375
385178	386738	15	+	SUT362	SUTs	SUTs	-0.327353047	-0.396057344	1.130259634	0.822780364	-0.590826595
386738	389570	15	+	DFG16, CRS5	ORF-T	ORF-T	0.08807337	-0.295709442	-0.051653036	-0.059868828	-0.509852149
389570	389890	15	+	YOR032W-A	ORF-T	ORF-T	1.707834383	1.434328205	0.562255542	0.692440847	0.421813087
392250	392522	15	+	unknown	no_ovlp	CUTs	2.914220281	0.095591645	0.104971468	-0.388880915	-0.388171867
394890	398562	15	+	CUT372	CUTs	CUTs	0.815977066	0.338978103	-0.693301547	-0.552348603	-0.0558346
400338	401282	15	+	PEP12	ORF-T	ORF-T	-0.141645435	-0.478335737	0.297560282	0.076924413	-0.394160882
401570	402730	15	+	CYC2	ORF-T	ORF-T	-0.258157371	-0.59807209	-0.265854245	-0.180465181	-0.505324505
405618	406842	15	+	CKB2	ORF-T	ORF-T	0.015024147	-0.682157159	-0.178693185	-0.250826731	0.002335009
406842	408378	15	+	GLO4	ORF-T	ORF-T	-0.188768332	0.560818781	1.022207331	0.625425374	0.231068944
407890	408378	15	+	unknown	no_ovlp2	SUTs	-0.409721679	0.397439639	0.530614551	0.613734825	0.049958865
408378	409586	15	+	CUE5	ORF-T	ORF-T	-0.758544521	-0.444617977	0.450624683	0.254917919	0.205953023
410506	412482	15	+	WHI2	ORF-T	ORF-T	-0.389167671	-0.937474615	-0.432561406	-0.101259756	-0.544957697
412730	413642	15	+	IRC23	ORF-T	ORF-T	0.135940066	-0.817997462	-0.231252453	0.026887873	0.028696796
413810	414210	15	+	TOM6	ORF-T	ORF-T	-0.5669784	-0.756437535	-0.157650218	0.168411025	0.001055935
418394	418866	15	+	CUT373	CUTs	CUTs	1.707077795	-0.649851494	-0.453477147	0.303475867	-0.153481192
421906	424738	15	+	SUT366	SUTs	SUTs	0.546769833	-0.115927364	-0.287207718	-0.269343823	-1.030630267
426474	427018	15	+	unknown	no_ovlp	CUTs	2.709438886	0.008568601	-1.545895054	-1.004671905	-0.524732983
427602	428258	15	+	YOR053W	other	other	0.756840788	-0.374518876	-0.097599914	-0.366941873	0.056387777
427858	428258	15	+	unknown	no_ovlp2	CUTs	2.15544042	-0.402428691	0.961368254	1.020371164	2.054989416
430282	431098	15	+	unknown	no_ovlp	CUTs	1.380037496	-0.029693422	-0.172116247	0.250017018	1.285492465
432138	433450	15	+	SGT1	ORF-T	ORF-T	0.134126341	-0.025528813	0.118263059	-0.167718681	0.241164739
435882	438746	15	+	CUT376	CUTs	CUTs	0.638059622	0.674889627	0.111778259	0.056571944	-0.538059079
438890	439282	15	+	unknown	no_ovlp	CUTs	1.540539438	-0.056579312	0.709819448	1.348606506	1.174078365
441386	442778	15	+	CKA2	ORF-T	ORF-T	0.12389027	-0.408645256	-0.597100704	0.027543319	-0.236390658
444186	444666	15	+	SUT367	SUTs	SUTs	-0.28289041	-0.057282205	0.910984532	0.046920421	-0.761379466
444666	445970	15	+	RPL3	ORF-T	ORF-T	-0.549858032	-0.259045399	-0.136838379	-0.168105395	-0.182801572
447114	448890	15	+	CYT1	ORF-T	ORF-T	-0.170158176	-0.156966327	0.385192208	0.380890115	-0.164619165
449370	451458	15	+	MSA1	ORF-T	ORF-T	0.048926306	-0.15962677	-0.009477607	0.183034043	-0.136379031
453706	454826	15	+	VPS5	ORF-T	ORF-T	0.290905199	-0.341262339	-0.263373185	-0.147639614	-0.294309112
458210	458650	15	+	CUT378	CUTs	CUTs	1.801656989	-0.144049127	0.08398405	0.04008979	-0.196166698
461850	462290	15	+	unknown	no_ovlp	CUTs	2.62191533	0.461969508	1.03362492	0.18410696	-1.936217431
462626	463922	15	+	CUT379	CUTs	CUTs	3.321497924	1.025118084	0.026798821	-0.10900232	-3.410952069
464754	466642	15	+	SGO1	ORF-T	ORF-T	-0.329024096	0.079098572	0.021909053	-0.07678288	1.462418798
468154	469298	15	+	UFE1	ORF-T	ORF-T	0.075855003	-0.471170424	0.263614972	-0.279753625	0.468844366
471882	472698	15	+	RTS2	ORF-T	ORF-T	0.222061348	-0.228490466	-0.079008934	-0.399821724	1.272844121
472698	473442	15	+	BUD21	ORF-T	ORF-T	0.173606028	-0.785332429	-0.531318671	-0.142132186	0.320019956

474602	476946	15	+	DIA2	ORF-T	ORF-T	0.929195409	0.337392928	-0.231469405	-0.025245858	-0.510419415
479482	480554	15	+	unknown	no_ovlp2	SUTs	-0.674949392	-0.390034588	-0.41875822	-0.090240547	-0.246934451
480554	481842	15	+	YOR084W	ORF-T	ORF-T	-0.161700419	-0.02863338	-0.197255137	-0.261565167	0.025131584
481978	483186	15	+	OST3	ORF-T	ORF-T	-0.451282885	-0.333221604	-0.357535514	-0.044779387	0.033244063
487634	489866	15	+	YVC1	ORF-T	ORF-T	-0.496374602	-0.654850741	-0.238318509	-0.107369062	-0.15592511
493386	494530	15	+	TMA46	ORF-T	ORF-T	0.143254576	0.042814197	-0.584433296	-0.139899266	0.249472907
494786	496354	15	+	ECM3	ORF-T	ORF-T	0.107456991	-0.843423258	-1.256129556	-0.673904851	-1.23351637
502706	503546	15	+	ARF3	ORF-T	ORF-T	0.254728471	-0.767418444	-0.709836549	-0.166084528	-0.269798721
504754	505738	15	+	unknown	no_ovlp	SUTs	0.643246981	0.354905156	1.292535113	0.920232374	0.794290624
505938	506346	15	+	RPS7A	ORF-T	ORF-T	0.691129737	-1.085875613	-1.368396246	-0.891288839	-0.490995296
511642	513098	15	+	KTR1	ORF-T	ORF-T	-0.347392158	-0.293110327	-0.350217942	-0.056609505	0.002758385
515146	516458	15	+	RAS1, YOR10	other	other	-0.513734059	-1.50543731	-0.937587336	-0.00207878	0.41726836
517458	520082	15	+	PIN2, YOR10	ORF-T	ORF-T	0.200320848	-0.237387421	-0.150224896	-0.271442663	-0.330942353
518970	520082	15	+	unknown	no_ovlp2	SUTs	0.172645845	-0.332722067	-0.212723126	-0.352079281	0.340500572
521090	522594	15	+	RGS2	ORF-T	ORF-T	1.168208883	-0.246190897	-1.794276213	-0.92651627	-0.406646023
522962	525026	15	+	LEU9	ORF-T	ORF-T	-0.004727756	-1.34551641	-1.311769279	-0.200219262	-0.559716801
525202	528522	15	+	INP53	ORF-T	ORF-T	-0.083932187	-0.398412489	-0.479105682	0.002640175	-0.276597763
528522	528802	15	+	unknown	no_ovlp2	SUTs	-0.193453253	-0.186916738	-0.484307999	-0.11249206	-0.507683109
528802	530282	15	+	TFC7	ORF-T	ORF-T	-0.653937284	-0.947078843	0.049196304	0.063966603	0.247858554
530418	531474	15	+	YOR111W	ORF-T	ORF-T	-0.012012181	-0.085732172	0.035199145	0.121207323	-0.243075642
531474	533850	15	+	CEX1	ORF-T	ORF-T	-0.37319285	-0.666359464	-0.274796743	-0.270026055	0.040043001
533978	536778	15	+	AZF1	ORF-T	ORF-T	-0.07325779	-0.560655684	0.185834622	0.251747912	-0.15523867
537474	538682	15	+	YOR114W	ORF-T	ORF-T	-0.112603065	0.253052726	0.831305538	-0.028572788	1.3745674
544954	546506	15	+	RPT5	ORF-T	ORF-T	0.151679724	-0.074440611	-0.01537371	-0.022131987	-0.371643262
546666	548602	15	+	YOR118W	ORF-T	ORF-T	-0.021323111	-0.46701314	0.068268444	0.037895264	0.533632151
550498	551058	15	+	SUT369	SUTs	SUTs	0.814888504	-0.533329045	-0.143130679	-0.312380553	0.02076975
551058	552210	15	+	GCY1	ORF-T	ORF-T	-0.351364696	0.176980564	1.993358887	0.874137692	1.105218447
553130	553970	15	+	CUT382	CUTs	CUTs	3.218439415	0.424590761	-0.573730579	0.010498431	0.185051761
559954	560914	15	+	unknown	no_ovlp	SUTs	0.352790423	-0.087347278	-0.116054447	0.062337117	-0.075978947
560914	564386	15	+	RGA1	ORF-T	ORF-T	0.206180905	-0.248017079	-0.295285082	-0.170088182	-0.17286712
571130	572466	15	+	SUT370	SUTs	SUTs	2.011400671	-0.392837108	-1.272605983	-0.08137725	-2.350115559
573130	574002	15	+	VPS17	ORF-T	ORF-T	-0.481247272	-0.434653541	0.195898698	0.030353626	0.077602946
575026	577818	15	+	EFT1	ORF-T	ORF-T	-0.478178156	-0.327058339	-0.631197351	-0.198534269	-0.238509523
578442	580146	15	+	BAG7	ORF-T	ORF-T	1.899375837	1.063875186	0.422898385	0.89886535	0.004952658
580146	581458	15	+	IDH2	ORF-T	ORF-T	-0.041056564	-0.439086456	0.045790237	-0.059076147	-0.092621402
584274	585186	15	+	CUT383	CUTs	CUTs	2.438187378	0.163706043	-1.488087238	-0.026717214	-0.431347762
586666	587322	15	+	SFL1	ORF-T	ORF-T	-0.660534023	-0.723638387	-0.337348194	-0.14286231	0.156335522
592850	594290	15	+	LSC1	ORF-T	ORF-T	-0.568131935	-0.088282737	0.863527743	0.329448183	0.753633696
606578	608650	15	+	MDM32	ORF-T	ORF-T	0.008389654	-0.63608123	0.31672188	-0.013271564	-0.048491541
611978	612586	15	+	MRPL23	ORF-T	ORF-T	-0.775659757	-0.736725935	0.092264445	0.032797022	-0.206751538
617186	617866	15	+	unknown	no_ovlp	CUTs	2.336962189	0.05426357	0.057773107	-0.472827612	-0.078317176
618714	619690	15	+	SUT372	SUTs	SUTs	0.873328135	-0.082868404	-0.155826966	-0.029104386	0.129061201
619690	624490	15	+	PDR5	ORF-T	ORF-T	0.15427178	-0.06484774	-0.589178222	0.146811449	-0.55445892
624722	626658	15	+	SLP1	ORF-T	ORF-T	0.429784549	-0.316624433	-0.499385928	-0.484157736	0.38993273
632162	633186	15	+	PET123	ORF-T	ORF-T	-1.234000931	-1.014139704	0.13892636	0.156327457	0.14035162
633786	636834	15	+	MTR10	ORF-T	ORF-T	0.28225774	-0.0778515	-0.419452966	-0.156791905	-0.251461843
636834	637066	15	+	unknown	no_ovlp2	SUTs	0.158493836	0.926854435	0.069969763	0.583854241	-0.021425483
637066	638970	15	+	YOR161W-A,	other	other	-0.100559671	0.728330637	0.185469487	-0.022270813	0.399236575
638282	638970	15	+	unknown	no_ovlp2	SUTs	-0.058406635	0.416562529	0.109075518	-0.258662479	0.040740117
638970	641026	15	+	SUT373	SUTs	SUTs	0.6330164	0.125612853	-0.121891116	0.785565757	0.99777895
642394	642722	15	+	CUT386	CUTs	CUTs	1.880866175	0.262173879	-0.008873267	-0.601813298	-0.626502296
642722	643378	15	+	DDP1	ORF-T	ORF-T	0.150414882	0.471981734	0.293862053	0.006288712	-0.149277833
644538	647066	15	+	SEY1	ORF-T	ORF-T	-0.436875506	-0.596261128	-0.264341004	0.106694747	-0.199022725
649298	651866	15	+	GLN4	ORF-T	ORF-T	-0.076517623	-0.20858355	-0.542976476	0.132999249	-0.387801977
654186	656722	15	+	YRM1	ORF-T	ORF-T	-0.157823844	-0.542512526	-0.513977053	-0.048266005	-0.403168382
657266	658442	15	+	DCS2	ORF-T	ORF-T	-0.44834622	-0.294732163	2.007901028	1.848809439	-0.13263116
658658	659722	15	+	MED4	ORF-T	ORF-T	-0.044194424	-0.597223227	-0.052781511	0.075403355	0.158095753
661930	662306	15	+	CUT387	CUTs	CUTs	0.74647127	-0.036872331	0.507469781	0.11253373	-0.105624285
662306	663690	15	+	HEM15	ORF-T	ORF-T	-0.536858808	-0.630156233	-0.312832845	-0.009506053	-0.374700364
667874	670370	15	+	SUT374	SUTs	SUTs	0.924621195	-0.703968146	-1.627258725	-1.533664693	-0.574692422
672682	673530	15	+	SUT375	SUTs	SUTs	1.310146447	0.760485695	0.73471073	1.17036626	-1.6152318
675882	677914	15	+	LAS17	ORF-T	ORF-T	-0.311565778	-0.203802203	-0.821853241	-0.143718044	-0.672411418
679282	680650	15	+	FYV12, SER1	other	other	-0.11110036	-0.739501541	-0.400421256	-0.176852601	-0.38677391
680650	682386	15	+	unknown	no_ovlp2	SUTs	0.416286279	0.323233347	-0.175884881	-0.485735042	-0.019816834
682386	683962	15	+	YOR186W	ORF-T	ORF-T	0.053205001	-0.175945589	0.95270542	0.991042613	1.320876862
683618	683962	15	+	unknown	no_ovlp2	SUTs	0.519501261	0.662153288	0.763682179	-0.444263997	-0.352296882
683962	685482	15	+	TUF1	ORF-T	ORF-T	-0.935196266	-0.435261264	0.005779125	-0.016063911	0.003671551
685690	687146	15	+	MSB1	ORF-T	ORF-T	-0.288871435	-0.133587621	-0.270774711	-0.258212043	0.770260285
689618	690194	15	+	IES4	ORF-T	ORF-T	-0.388924968	-0.959758817	-0.146509116	0.178705497	-0.672028293
692402	697466	15	+	RIS1	ORF-T	ORF-T	0.338601383	-0.094648318	-0.508607913	-0.14141269	-0.538270049
701826	703034	15	+	SUT377	SUTs	SUTs	0.068633506	-0.449672189	0.099188974	0.04401101	-1.429877887
710418	711690	15	+	PEX27	ORF-T	ORF-T	-0.764345137	-0.815251202	1.077975566	0.395521496	0.299919949
712810	715570	15	+	SLK19	ORF-T	ORF-T	-0.175515659	-0.114632823	0.255870409	-0.140020289	0.690813748
717074	718498	15	+	MCA1	ORF-T	ORF-T	-0.089762879	-0.15145355	-0.364199574	-0.12627849	-0.109480495
720386	720754	15	+	unknown	no_ovlp	CUTs	1.531942925	0.042038328	-1.428513096	-0.380392949	1.296408447
721946	722690	15	+	HIS3, YOR20:	ORF-T	ORF-T	0.240870533	-0.223964647	-0.34387595	-0.811663061	-0.12381113
722898	725442	15	+	YOR203W, D	ORF-T	ORF-T	-0.00135184	-0.217783575	-0.653563061	-0.089786603	-0.279237109
727498	729706	15	+	NOC2	ORF-T	ORF-T	0.330392621	-0.36701047	-0.638757917	0.036760258	0.02841493
733746	736298	15	+	PTP2	ORF-T	ORF-T	-0.104407677	-0.508078834	0.257667675	0.079790776	-0.007503117
738274	738850	15	+	RPB10	ORF-T	ORF-T	0.07881736	-0.552568312	-0.183110985	0.199195106	0.094779259
742770	744330	15	+	STE4	ORF-T	ORF-T	0.052092353	-0.673721154	-0.806295375	-0.148041509	-0.500234932
749202	752138	15	+	RFC1	ORF-T	ORF-T	-0.284523199	-0.248836632	-0.198652946	0.011297463	0.258749476

755322	756298	15	+	RCN2	ORF-T	ORF-T	0.259923569	-0.614897639	0.015075446	0.207293664	-0.523565175
757866	758274	15	+	ODC2	ORF-T	ORF-T	0.572796198	-0.973897492	0.247561055	0.001557459	0.103904318
759762	760778	15	+	YOR223W	ORF-T	ORF-T	-0.468253576	-0.025437331	0.9121674	0.407841496	0.31473957
761578	762458	15	+	unknown	no_ovlp	CUTs	1.889902727	0.343852283	-0.985846672	-0.860992438	-0.106495956
762458	763810	15	+	YOR227W	ORF-T	ORF-T	0.076594441	-0.479390194	-0.160941051	-0.043005549	-0.050243803
768338	770002	15	+	WTM2	ORF-T	ORF-T	-0.684545458	-0.212581771	0.334906461	0.477583753	0.572699109
770698	772298	15	+	WTM1	ORF-T	ORF-T	0.206966141	-0.12752057	0.115940042	0.42406146	0.069030335
772482	773258	15	+	MKK1	ORF-T	ORF-T	0.549519928	0.091732461	-0.040142389	-0.409060797	0.03258686
774474	775394	15	+	MGE1	ORF-T	ORF-T	-0.37761734	-0.587420607	-0.363655256	0.147500313	-0.0020303
775706	778314	15	+	KIN4	ORF-T	ORF-T	0.018316874	-0.219244486	-0.354803213	-0.315762385	0.558400011
780106	780458	15	+	unknown	no_ovlp	SUTs	0.909335855	-0.808899623	-1.149225675	-0.935285444	-0.513377445
780594	780906	15	+	unknown	no_ovlp	SUTs	0.128118634	-0.180756542	0.491638608	-0.204734051	-0.429219015
780906	781610	15	+	DFR1	ORF-T	ORF-T	-0.668123253	-0.115161476	-0.335815171	0.036999775	0.240979454
781610	783666	15	+	HES1	ORF-T	ORF-T	0.601789701	0.661305383	0.619979414	0.775877194	-0.728618738
783282	783666	15	+	unknown	no_ovlp2	SUTs	-0.188700292	0.095023128	0.304018861	-0.093877149	-0.083410448
783666	784658	15	+	YOR238W	ORF-T	ORF-T	0.481850963	0.19300825	-0.12187418	-0.221115596	-0.528865002
784858	786946	15	+	ABP140	ORF-T	ORF-T	-0.151575464	-0.61671503	-0.584365671	-0.157928489	-0.384897459
787178	788882	15	+	MET7	ORF-T	ORF-T	0.223526873	-0.576713011	-0.514066139	-0.260977922	-0.20644995
792490	794026	15	+	ESA1	ORF-T	ORF-T	0.136536652	-0.606412312	-0.023038676	-0.049898431	-0.083439678
797450	798530	15	+	SRL1, YOR24	ORF-T	ORF-T	-0.265222037	-0.248408496	0.017793551	-0.158366409	0.131915662
802514	803642	15	+	unknown	no_ovlp	SUTs	0.799125453	0.336289165	-0.541811642	-0.127849249	0.124937841
803642	804275	15	+	TMA16	ORF-T	ORF-T	0.455678268	-0.133657603	-0.506239699	-0.091171714	0.072768129
804363	805011	15	+	NAT5	ORF-T	ORF-T	0.265805495	-0.994532948	-0.405331555	-0.231791721	-0.198981137
810995	811523	15	+	CDC31	ORF-T	ORF-T	-0.219377572	-0.863960718	-0.315325937	0.034154274	-0.726120659
811675	812395	15	+	HNT3	ORF-T	ORF-T	0.870133678	-0.068554498	-0.140399846	-0.336988618	-0.512069558
813947	815923	15	+	GCD1	ORF-T	ORF-T	0.260365276	-0.473634005	-0.586853385	-0.537262467	-0.066420956
817243	818459	15	+	YOR262W	ORF-T	ORF-T	0.060101984	-0.044774843	0.259128306	-0.118514014	0.856068081
818819	820171	15	+	DSE3	ORF-T	ORF-T	-0.121664359	-0.840073062	0.190582671	-0.024347262	-0.048052767
820435	820907	15	+	RBL2	ORF-T	ORF-T	-0.055780873	-0.401323215	0.081265114	-0.384064325	-0.570356349
820907	822363	15	+	PNT1	ORF-T	ORF-T	0.091203457	-0.430847759	-0.28365799	-0.264734002	-0.342544729
825251	826331	15	+	CUT389	CUTs	CUTs	1.671139891	0.337097396	0.027016495	-0.654505531	-0.918054604
826331	828019	15	+	PAC1	ORF-T	ORF-T	-0.223890943	-0.391527467	-0.269077946	-0.103386434	-0.165863057
831091	832315	15	+	unknown	no_ovlp	SUTs	0.251236703	0.344956787	-0.272723567	0.64370237	1.515972319
832347	832611	15	+	SNR8	other	other	-0.134379566	-0.223903413	0.035528479	-0.098419777	0.106542001
832611	833931	15	+	YTM1	ORF-T	ORF-T	0.55962033	-0.014788962	-0.601020576	0.160790277	0.16715327
837011	837731	15	+	MOD5	ORF-T	ORF-T	-0.484585604	-0.985464223	-0.571852742	-0.235167663	-0.371006512
841283	841915	15	+	CAF20	ORF-T	ORF-T	-0.03685337	-0.766578458	-0.837648327	-0.355382275	-0.208111421
842411	842779	15	+	SNR5	other	other	2.394107442	-1.969136522	-0.257625386	-0.173956922	0.172349191
842955	843779	15	+	HEM4	ORF-T	ORF-T	-0.825697678	-0.732537054	0.202570598	0.202521333	-0.033134777
844891	845579	15	+	CUT390	CUTs	CUTs	1.26766996	0.214102184	-0.501309001	0.00996277	-0.915507766
847363	848291	15	+	YOR283W	ORF-T	ORF-T	0.181875347	-0.816728907	-0.889135984	-0.341088162	-0.024614764
848435	849499	15	+	HUA2	ORF-T	ORF-T	0.357830327	-0.335688508	-0.16950558	-0.485218326	0.531486159
849627	850123	15	+	YOR285W	ORF-T	ORF-T	-0.445038174	-0.358119674	0.969390565	0.074139932	0.452352502
850275	850971	15	+	FMP31	ORF-T	ORF-T	-0.194672115	-0.694690006	-0.150982344	-0.056449737	0.089971483
853379	854179	15	+	YOR289W	ORF-T	ORF-T	0.023428954	-0.726685815	0.308588806	0.457745853	-0.697909162
861035	865651	15	+	YOR291W	ORF-T	ORF-T	0.160781454	-0.106903985	-0.290192553	-0.043339364	0.340375238
867147	867595	15	+	RPS10A	ORF-T	ORF-T	0.909059525	-0.428742197	-0.839956765	-0.935493927	-0.087099519
868307	869099	15	+	RRS1	ORF-T	ORF-T	0.254658629	-0.431748267	-0.616922251	-0.300873361	0.480895332
869099	870123	15	+	unknown	no_ovlp2	SUTs	0.03533392	-0.15651017	-0.146515651	-0.742109791	0.048458097
870123	874251	15	+	YOR296W	ORF-T	ORF-T	0.371505527	0.026937111	-0.581927136	-0.190846594	-0.416236013
878355	880675	15	+	BUD7, YOR3(	ORF-T	ORF-T	0.173926288	-0.44184978	-0.564228064	-0.130587036	-0.723971595
880955	882659	15	+	RAX1	ORF-T	ORF-T	-0.208049187	-0.012411201	-0.59826961	-0.36421659	0.122172816
882659	884203	15	+	YOR302W, CI	ORF-T	ORF-T	-0.230384099	-0.292757154	-0.30499991	-0.133320952	0.116515424
884435	887947	15	+	ISW2	ORF-T	ORF-T	0.083593017	-0.208315636	-0.574265018	-0.20595621	-0.26845417
888971	889835	15	+	YOR305W	ORF-T	ORF-T	0.08243617	-0.569212091	-0.015083334	-0.179314939	0.131237402
904667	906203	15	+	SFG1	ORF-T	ORF-T	-0.053187644	-0.497670721	-1.820780154	-0.686781668	-0.211149407
907867	909267	15	+	SUT382	SUTs	SUTs	0.522964407	0.393820503	1.023944208	0.06350738	-0.513039122
909267	911691	15	+	FAA1	ORF-T	ORF-T	-0.102063567	-0.933586341	-0.264952037	0.146799254	-0.439547018
912795	914019	15	+	HSH49	ORF-T	ORF-T	0.082410848	-0.615863645	0.032294442	-0.162059977	0.167565349
915987	918323	15	+	PMT3	ORF-T	ORF-T	-0.817290417	-0.063375635	-0.24633952	0.32042767	0.752737613
925523	930779	15	+	MYO2	ORF-T	ORF-T	-0.398194296	-0.206407983	-0.260578858	-0.111695414	0.048091973
931043	931635	15	+	unknown	no_ovlp2	SUTs	0.271018057	0.338921509	0.138874112	-0.059640848	-0.128670039
931635	933547	15	+	PDR10	ORF-T	ORF-T	0.025333135	1.687854405	2.580652613	2.41058156	3.21054878
933547	936723	15	+	PDR10	ORF-T	ORF-T	0.037234097	0.277789231	0.561551393	0.550188655	1.334771974
939611	939891	15	+	unknown	no_ovlp	CUTs	2.102177134	0.525981272	0.251815387	0.301212264	0.577348093
943627	944419	15	+	VMA4	ORF-T	ORF-T	-0.048785811	-0.329989847	-0.728387588	-0.056509632	-0.069869959
944419	946091	15	+	MRS2	ORF-T	ORF-T	-0.255131124	-0.494295285	-0.707882933	-0.379405928	0.155039015
949755	954003	15	+	KRE5	ORF-T	ORF-T	-0.097384495	0.108313621	-0.446878806	-0.164843025	-0.24294372
954347	956851	15	+	TEA1	ORF-T	ORF-T	0.351345544	-0.471237277	-0.795259626	-0.260960141	-0.243914041
956851	958187	15	+	YOR338W	ORF-T	ORF-T	0.724735252	0.98350596	1.560669438	1.460366035	1.310948256
960875	966259	15	+	RPA190	ORF-T	ORF-T	0.131876964	-0.398383314	-0.543580112	0.15465982	-0.103177419
968171	969763	15	+	unknown	no_ovlp	CUTs	1.254143811	0.235487459	0.438993989	0.610426252	-0.885586123
969763	971083	15	+	unknown	no_ovlp	CUTs	2.128063032	1.530413354	0.095287592	0.138298741	-2.46874767
978499	979571	15	+	SUT386	SUTs	SUTs	0.198038847	-0.023509874	-0.683260926	-0.224026529	-0.579464656
979571	980603	15	+	CUT394	CUTs	CUTs	2.079030864	-0.58571064	-0.812886259	-0.161019546	-2.618320684
981659	984947	15	+	REV1	ORF-T	ORF-T	-0.424487651	-0.340802191	-0.021589401	-0.014576716	0.405805082
988707	989763	15	+	SUT387	SUTs	SUTs	0.349564151	-0.229883224	0.609141956	0.628488235	-0.795084305
989763	992891	15	+	CIN1	ORF-T	ORF-T	1.092664848	-0.312391864	-0.686493069	-0.179878124	0.130522484
995083	998291	15	+	CUT395	CUTs	CUTs	1.007812256	-0.539000324	0.272781352	0.146268735	0.219178841
1001083	1001547	15	+	CUT396	CUTs	CUTs	2.545612746	0.209393841	-0.52652257	0.518371324	0.697251227
1003971	1004611	15	+	unknown	no_ovlp2	CUTs	1.380814823	-0.768547182	0.26186025	-0.302852358	0.363717018
1004611	1006867	15	+	GDS1	ORF-T	ORF-T	-0.683875535	-0.934452638	-0.317977206	-0.230618529	0.270480577

1007155	1009235	15 +	YOR356W	ORF-T	ORF-T	0.160961918	-0.04823348	0.069862016	0.485771329	-0.29756881
1010083	1011067	15 +	HAP5	ORF-T	ORF-T	-0.776886483	-0.571969889	0.449843335	-0.108583559	0.129570387
1011067	1013051	15 +	VTS1	ORF-T	ORF-T	0.452383368	0.042677519	-0.976682224	-0.672896474	0.527360262
1019107	1020003	15 +	unknown	no_ovlp	CUTs	1.491446798	0.830174823	0.5768078	0.757701858	0.459988192
1023451	1025651	15 +	YOR364W, Y(	other	other	1.701571359	-0.153509319	-0.207769205	-0.386099855	-0.61528565
1026003	1026643	15 +	SCP1	ORF-T	ORF-T	-1.111262166	-1.143754264	0.185678843	0.170579862	0.417707786
1026643	1028403	15 +	RAD17	ORF-T	ORF-T	1.084491173	0.729523255	0.36771441	-0.316902812	-0.1231587
1028075	1028403	15 +	unknown	no_ovlp2	SUTs	0.428129655	0.737139844	-0.298421868	0.180297164	-0.235548561
1031563	1031915	15 +	CUT398	CUTs	CUTs	3.076356021	-0.202495668	-0.907239841	0.095259365	0.558565378
1036795	1039475	15 +	NUD1	ORF-T	ORF-T	-0.472814785	0.010909024	-0.111476733	0.093947868	0.776009772
1039787	1041539	15 +	ALD4	ORF-T	ORF-T	-0.998860648	-0.939059997	1.983839145	1.371057845	1.477126052
1043635	1044731	15 +	unknown	no_ovlp	CUTs	1.051051456	0.187592353	-0.028471502	0.148826396	-0.58276403
1044731	1048075	15 +	YOR376W-A,	other	other	0.442141976	-0.457657648	-0.213737796	0.19849475	-0.673309977
1048075	1049515	15 +	unknown	no_ovlp2	SUTs	0.179361014	0.415567929	-0.0033491	-0.017923632	-0.036981715
1049515	1051051	15 +	YOR378W	ORF-T	ORF-T	-0.001097516	0.75053195	0.009913399	0.404072812	0.052348021
1051051	1052971	15 +	RDR1	ORF-T	ORF-T	-0.517136066	-0.912736365	-0.004953634	0.203272149	-0.031992943
1054819	1057771	15 +	FRE3	ORF-T	ORF-T	1.306887407	0.303212333	-0.516098253	0.284000048	-2.470653178
1059219	1059987	15 +	FIT2	ORF-T	ORF-T	-1.01518627	-0.036626725	2.035698373	1.770549414	-0.300513782
1060643	1061003	15 +	unknown	no_ovlp	SUTs	-0.208459139	0.724732418	-0.370092819	-0.315412046	-0.40902835
1064611	1065043	15 +	unknown	no_ovlp	CUTs	1.501863839	-0.590623802	0.469692527	-0.326155921	-0.292436966
1065043	1066211	15 +	YOR385W	ORF-T	ORF-T	0.904674577	0.274368394	-0.506737569	0.556943998	-1.140964587
1066827	1068931	15 +	PHR1	ORF-T	ORF-T	0.675437459	0.159405826	0.319599223	0.338465286	-1.462397461
17845	22325	16 -	unknown	no_ovlp	SUTs	0.912526273	-0.604437876	-0.519452477	-0.216221599	-0.644479395
24357	24701	16 -	SUT803	SUTs	SUTs	0.461415631	-1.888145712	-1.765056176	0.310241483	0.633952343
28605	29829	16 -	YPL272C	ORF-T	ORF-T	0.878799641	-0.393903453	-0.259207699	-0.284308895	-0.400049128
39549	40237	16 -	unknown	no_ovlp	CUTs	2.522564885	0.012213583	-0.346658587	0.947873379	0.042919019
43117	44533	16 -	YPL264C	ORF-T	ORF-T	0.593031659	-0.036393467	-0.194782699	-0.525247121	0.238266904
44533	46517	16 -	KEL3	ORF-T	ORF-T	0.602598955	0.128160252	-0.37088881	0.031896439	0.214135243
46517	47189	16 -	SUT805	SUTs	SUTs	-0.110108129	-0.325337669	1.247710571	-0.056712844	-0.338149316
51053	52677	16 -	APM1	ORF-T	ORF-T	0.273701893	-0.488694989	-0.536187339	-0.059086558	-0.581163687
53429	63061	16 -	THI21, YPLC(	other	other	0.407674363	0.119038405	-0.14091603	-0.274883691	-0.316568587
56037	63061	16 -	unknown	no_ovlp2	SUTs	0.103431741	-0.470119353	0.437014518	-0.079773135	-0.32058426
64773	66853	16 -	CLN2	ORF-T	ORF-T	-0.289798178	0.206413849	0.229458669	-0.193223247	0.66960674
70949	73317	16 -	VIK1	ORF-T	ORF-T	-0.505044395	-0.238115713	0.63330085	-0.135737602	1.403482821
73069	73317	16 -	unknown	no_ovlp2	SUTs	-0.981891233	-0.115026156	0.538408608	0.201148568	0.289858022
73317	73917	16 -	YAH1	ORF-T	ORF-T	-0.302181361	0.005691465	-0.455204338	-0.305299832	-0.113663994
74149	74813	16 -	ICY2	ORF-T	ORF-T	-0.339379571	-0.677533253	0.219902523	-0.429187531	-0.635741728
74813	75501	16 -	unknown	no_ovlp	SUTs	0.468380265	0.315601517	0.162535727	0.035944316	-0.127206381
75981	76229	16 -	RPL36B	ORF-T	ORF-T	0.636598663	-0.65655867	-1.012943687	-0.944070398	-0.060866834
76373	76693	16 -	unknown	no_ovlp2	SUTs	-0.341585779	0.47338659	-0.093868174	0.460168129	0.182535665
76693	79589	16 -	GYP5	ORF-T	ORF-T	-2.863935423	0.186496735	2.728160984	1.294408183	2.714812777
79589	82333	16 -	GAL4	ORF-T	ORF-T	0.133522501	-0.281713663	0.012199392	0.268173158	-0.860177699
82533	84293	16 -	YPL247C	ORF-T	ORF-T	-0.572531831	-0.763223509	0.533683371	0.507035299	-0.181570403
84413	85341	16 -	RBD2	ORF-T	ORF-T	-0.626076502	-0.040002322	0.275915918	-0.4264347	0.645388392
86861	88053	16 -	HUT1	ORF-T	ORF-T	0.218807041	-0.195282734	-0.602284862	-0.392643786	-0.219149919
90397	95149	16 -	IQG1	ORF-T	ORF-T	0.193191322	0.115062495	-0.11200181	-0.218980193	0.344988011
95285	96173	16 -	CIN2	ORF-T	ORF-T	-0.333098475	-0.278377789	-0.271885789	-0.315514793	-0.394009135
96381	98677	16 -	HSP82	ORF-T	ORF-T	0.269428745	-0.3414581	0.525816117	0.0048471	-0.232353313
101485	102765	16 -	YPL236C	ORF-T	ORF-T	0.304923231	-0.341656637	-0.031434606	-0.049133403	-0.334805599
104813	105493	16 -	TFP3	ORF-T	ORF-T	-0.186191523	-0.315498593	-0.17291212	0.055694624	0.183536803
105493	105941	16 -	CUT879	CUTs	CUTs	2.10303463	-0.077154993	-0.260001387	-0.595732729	-0.100561808
106213	106765	16 -	unknown	no_ovlp	CUTs	2.477587175	0.15789226	-0.829711444	-0.390901967	0.084745709
107733	108245	16 -	CUT880	CUTs	CUTs	3.045888641	0.365100245	-0.976308622	-0.040863674	-0.03700491
108245	109013	16 -	unknown	no_ovlp	SUTs	-0.063077052	0.471465446	0.11745147	-0.022058201	0.031288554
117733	118005	16 -	CUT881	CUTs	CUTs	3.094906656	0.474586069	-1.398520228	-0.424595084	-1.293577323
120069	121317	16 -	ALG5	ORF-T	ORF-T	-0.388382398	-0.508954986	-0.548224609	-0.342309416	0.725494316
126477	128277	16 -	MMT2	ORF-T	ORF-T	-0.16352732	-0.678911781	0.174180208	0.425207986	-0.54238887
128493	131413	16 -	GRE1	ORF-T	ORF-T	-0.929404159	-0.939742964	2.061976171	0.354067773	0.862915356
129229	131413	16 -	unknown	no_ovlp2	CUTs	1.440410716	0.269772464	-0.233468165	-0.248052688	0.51406609
131685	132197	16 -	unknown	no_ovlp	CUTs	3.715933977	0.099315277	-0.564548029	-0.092638524	0.357119079
132197	132717	16 -	SUT809	SUTs	SUTs	2.740710073	0.378147928	-0.471840712	-0.039978632	-0.536199374
134901	135461	16 -	CUT884	CUTs	CUTs	2.220756584	0.10922865	-0.960595141	0.018278448	1.31131561
139565	143205	16 -	BMS1	ORF-T	ORF-T	0.126719388	-0.215203284	-0.811695456	0.014618377	0.251687231
145453	147157	16 -	SUT810	SUTs	SUTs	0.115208785	-0.631232644	0.338547422	0.920016205	1.087788759
148477	150285	16 -	THI6	ORF-T	ORF-T	0.174763173	-0.078616385	-0.070669774	-0.01572009	-0.29495071
151349	153157	16 -	PUS1	ORF-T	ORF-T	0.494063789	0.085073163	-1.026538164	-0.297849262	0.089859441
154213	156221	16 -	SRP72	ORF-T	ORF-T	-0.207810824	-0.769147011	-0.265791891	-0.07244721	-0.172382257
156221	156445	16 -	unknown	no_ovlp2	SUTs	-0.220205707	0.204105786	-0.624400986	-0.349385071	1.102601741
156445	158885	16 -	IPL1	ORF-T	ORF-T	0.123099116	0.059952228	0.297202175	-0.629650239	0.837196205
157653	158885	16 -	unknown	no_ovlp2	SUTs	0.758769136	0.376543077	-0.5769567	-0.33191596	-0.011927443
158885	159573	16 -	CUT885	CUTs	CUTs	2.129538022	0.119667111	-0.440594655	0.017319841	0.47708987
162517	163629	16 -	YPL206C	ORF-T	ORF-T	-0.29729422	-0.531670587	0.00603084	0.065863516	0.608427411
163629	164053	16 -	CUT886	CUTs	CUTs	-0.480170328	-0.859454369	1.132786979	0.2229227	-0.263394432
167677	169397	16 -	AFT2	ORF-T	ORF-T	0.067743659	-0.712522733	-0.361138811	-0.455418906	0.052925504
169397	169693	16 -	unknown	no_ovlp2	SUTs	0.563366345	-0.084235554	-0.579193695	-0.592312802	-0.683728484
169693	171973	16 -	YIG1	ORF-T	ORF-T	0.781861765	-0.09729476	0.525502835	-0.304742215	-0.221816272
171189	171973	16 -	unknown	no_ovlp2	SUTs	0.753225206	-0.148608703	-1.069767386	-0.065765115	0.044296824
171973	172773	16 -	YPL199C	ORF-T	ORF-T	-0.222281578	-0.55207253	-0.062459803	0.061424265	0.25436697
175661	175997	16 -	unknown	no_ovlp	CUTs	1.5866192	-0.215573787	-0.732448345	-0.130962014	0.197210156
183109	184677	16 -	PRM3, YPL19	other	other	0.064584708	-0.055956187	0.448539811	0.080553822	-0.320431464
184877	187837	16 -	NAB3	ORF-T	ORF-T	0.224781116	-0.153995463	-0.302607874	-0.097733807	-0.190469125
188085	188605	16 -	YPL189C-A	ORF-T	ORF-T	-0.021380657	-0.920273982	0.723361476	0.129914392	0.246041868

190733	191205	16	-	CUT888	CUTs	CUTs	2.188045428	0.001174844	0.289721252	-0.393342684	-0.020106553
194317	195845	16	-	UIP4	ORF-T	ORF-T	-0.961797599	-1.227033467	1.899745395	1.419470038	0.445444121
198461	198861	16	-	MRN1	ORF-T	ORF-T	0.499819777	-1.083899192	0.273463174	0.01393082	-0.213411877
199437	202533	16	-	YPL183C	ORF-T	ORF-T	0.532085782	-0.319055841	-1.012526374	-0.144973323	0.148003421
204269	204957	16	-	CUT890	CUTs	CUTs	1.670195226	0.348616899	-0.02038045	0.486697566	0.50334783
206877	207669	16	-	CUT891	CUTs	CUTs	2.595619791	0.264065609	-1.387802558	-0.340438923	0.601374643
212941	214165	16	-	CUP9	ORF-T	ORF-T	-0.006519125	-0.51125907	0.142209028	0.120572364	-0.149886569
215725	218405	16	-	TRE1	ORF-T	ORF-T	-0.421030462	-0.212384617	0.29615237	0.137164141	0.127421569
220341	222909	16	-	NIP100	ORF-T	ORF-T	0.075389163	-0.552600247	-0.281140664	-0.274382643	0.040151285
224309	225765	16	-	COX10	ORF-T	ORF-T	-1.108569399	-0.949847694	0.205237916	-0.062722238	0.050038215
225765	226053	16	-	unknown	no_ovlp2	SUTs	-1.030462539	0.056547171	0.071179887	0.014694462	-0.023112699
226053	227477	16	-	OYE3	ORF-T	ORF-T	-0.393454639	1.105810343	2.312764612	2.082556565	0.414536249
228837	230869	16	-	MEX67	ORF-T	ORF-T	-0.01655282	-0.690807164	-0.391526616	-0.140302428	-0.274158928
232549	237141	16	-	REV3	ORF-T	ORF-T	0.603247095	-0.1066949	-0.546541725	-0.572331195	-0.255295279
237637	239157	16	-	unknown	no_ovlp	CUTs	2.683029895	0.667230673	-0.551303755	0.719679339	-1.492824528
239157	241709	16	-	SET6, MLH3	ORF-T	ORF-T	-0.550812578	-0.633917548	0.683149614	0.335011474	1.301694279
241709	242749	16	-	SVS1	ORF-T	ORF-T	-0.072152258	0.013084623	0.521601165	0.046836948	0.328546701
242749	244077	16	-	YPL162C	ORF-T	ORF-T	-0.581115576	-0.551722507	0.172531242	0.143452203	0.680014624
244277	246277	16	-	BEM4	ORF-T	ORF-T	0.013439852	-0.534399641	-0.174948082	-0.052640822	0.191036053
251213	251781	16	-	PET20	ORF-T	ORF-T	0.500806044	-0.092814926	-0.165966075	0.120540885	0.040078042
252045	254421	16	-	YPL158C	ORF-T	ORF-T	-0.701604665	-0.831555146	-0.025119846	0.009480881	0.463094541
255829	256933	16	-	unknown	no_ovlp2	SUTs	0.19506592	-0.012949273	0.417052563	0.968454315	-2.272735127
256933	259357	16	-	KIP2	ORF-T	ORF-T	-0.667389072	-0.63923477	0.289153202	0.303276869	0.64992854
259589	261189	16	-	PEP4	ORF-T	ORF-T	-0.255339694	-0.111871898	0.554169243	0.275998946	0.45182705
261669	263613	16	-	RAD53	ORF-T	ORF-T	-0.383195101	-0.214540161	-0.138749295	-0.33825309	0.739199204
266109	267565	16	-	PRP46	ORF-T	ORF-T	0.120038284	-0.740940746	-0.348850633	0.104241495	0.121354324
270333	271013	16	-	unknown	no_ovlp	CUTs	1.697257522	0.127089205	-1.074755833	-0.097439078	0.267094033
272117	272925	16	-	PPT2	ORF-T	ORF-T	0.497290333	-0.319686938	0.211718292	0.075986105	0.544972606
276029	277581	16	-	NOP53	ORF-T	ORF-T	0.177753666	-0.813163561	-0.65397909	-0.220854411	-0.136484715
277877	279933	16	-	KES1	ORF-T	ORF-T	-0.348315418	-0.841083977	-0.469205398	-0.005697327	-0.179812347
281077	281389	16	-	unknown	no_ovlp	SUTs	-0.078159598	-0.046991949	-0.280218427	-0.40808614	0.172088742
284813	286309	16	-	YPL141C	ORF-T	ORF-T	0.117712871	-0.096146662	0.316901808	-0.069933969	0.974192105
287341	289237	16	-	MKK2	ORF-T	ORF-T	0.489119021	0.100480398	0.341420845	0.21525731	0.041075785
289581	291109	16	-	UME1	ORF-T	ORF-T	-0.512614984	-1.128217077	-0.224857529	0.187643744	0.187688894
291181	292445	16	-	SPP1	ORF-T	ORF-T	-0.058091935	-0.250024527	0.300406931	0.139124517	0.392861162
296789	297309	16	-	GIP3	ORF-T	ORF-T	0.377229296	-0.114061109	0.065449583	0.182181729	0.332299144
298525	299637	16	-	unknown	no_ovlp2	SUTs	-0.114041312	0.946079	1.733661158	1.240577724	2.187603823
299637	301317	16	-	RDS2	ORF-T	ORF-T	-0.045834828	-0.513722888	0.078881464	-0.100016084	0.261146263
306493	308301	16	-	TBF1	ORF-T	ORF-T	-0.488601491	-0.325518324	-0.308105046	-0.015467614	0.638827622
308525	309637	16	-	HHO1	ORF-T	ORF-T	-0.292470736	-0.106319135	-0.00096328	-0.249836413	0.675373698
317469	319485	16	-	RNY1	ORF-T	ORF-T	-0.752406425	-0.629299538	1.137462828	0.701223111	0.472217608
319013	319485	16	-	unknown	no_ovlp2	SUTs	0.003305993	-0.188449464	0.100320423	-0.123099406	0.236032701
319485	320773	16	-	TFB2	ORF-T	ORF-T	0.054052389	-0.162597491	-0.100388131	-0.077321695	0.202373758
324381	326293	16	-	DBP1	ORF-T	ORF-T	-1.151892624	0.008862588	1.735280391	1.186782428	3.051792816
327693	328909	16	-	IDI1	ORF-T	ORF-T	0.141087207	-0.521765763	-0.495778029	0.002950756	-0.065363307
335357	335765	16	-	BEM3	ORF-T	ORF-T	-0.413996309	-1.113705254	-0.078976932	0.026333566	-0.367572978
335765	337381	16	-	YPL113C	ORF-T	ORF-T	-0.163389294	0.954867056	1.529444224	0.287916074	0.161307095
337157	337381	16	-	unknown	no_ovlp2	SUTs	-0.250289757	0.346908982	0.030632812	0.063091091	0.038145613
337381	338677	16	-	PEX25	ORF-T	ORF-T	0.106299681	-0.598387289	-0.105531961	0.012012955	0.402783861
340997	344061	16	-	GDE1	ORF-T	ORF-T	-0.085116218	0.368822721	0.399423986	0.083432728	-0.304309109
345941	347471	16	-	YPL109C	ORF-T	ORF-T	0.500974108	-0.044659253	0.351411843	0.132127169	-0.511602971
347655	348919	16	-	SUT812	SUTs	SUTs	1.117395227	-0.119142524	0.006246377	-0.347818269	0.431607164
350119	352375	16	-	SSE1	ORF-T	ORF-T	-0.170255158	-0.440882382	-0.281562642	0.045471217	-0.357430772
352783	355431	16	-	YPL105C	ORF-T	ORF-T	-0.287644758	-0.551715441	-0.328117434	0.091065563	-0.327347534
357767	359439	16	-	FMP30	ORF-T	ORF-T	0.280548598	0.213093931	-0.119347845	-0.419921287	-0.309073346
363303	364055	16	-	FMP14	ORF-T	ORF-T	-0.186413257	-0.928617682	-0.067969113	0.138856945	-0.762964228
364191	364791	16	-	MGR2	ORF-T	ORF-T	0.166312463	-0.148255875	0.258898552	-0.252945926	-0.225717318
366423	366775	16	-	ERI1	ORF-T	ORF-T	-0.293902048	-0.334710369	0.485196598	0.552816059	0.320724018
368031	369439	16	-	EEB1	ORF-T	ORF-T	-0.542196032	-1.511523923	-1.25087306	-0.786280086	-2.192428394
369775	370679	16	-	SEC62	ORF-T	ORF-T	-0.434442765	-0.223229014	-0.179000235	-0.336312892	0.238775774
374575	374991	16	-	unknown	no_ovlp	CUTs	1.316170196	0.119563348	0.60885276	-0.309449235	-0.085697398
377999	378399	16	-	RPS6A	ORF-T	ORF-T	0.665266622	-0.573157738	-0.766537963	-0.688485286	-0.1417903
381047	381383	16	-	RLM1	ORF-T	ORF-T	-0.72098156	-0.761727009	0.245671567	0.011147113	-0.125109118
382743	382983	16	-	CUT896	CUTs	CUTs	2.452896857	-0.357005701	-0.988068032	-0.385503166	0.02796192
384663	386487	16	-	ELP3	ORF-T	ORF-T	-0.031870871	-0.385923765	-0.323964855	0.117101814	0.222545641
392583	394031	16	-	unknown	no_ovlp	SUTs	0.396281125	0.226363834	0.931091481	0.784337072	1.487944242
396607	398127	16	-	SEN54	ORF-T	ORF-T	-0.365288777	-0.62423562	-0.107509907	0.02721067	0.081367051
400655	404215	16	-	MOT1	ORF-T	ORF-T	-0.022842378	-0.506954353	-0.868575092	-0.019142146	0.169944779
407743	408815	16	-	ATP4	ORF-T	ORF-T	-0.160628912	-0.382178217	0.379020725	-0.115567054	0.440836915
409055	410063	16	-	YPL077C	ORF-T	ORF-T	0.478263818	-0.228228461	-0.170033801	-0.523840372	-0.77275885
410623	411591	16	-	CUT897	CUTs	CUTs	2.261545441	-0.039296924	-0.788684564	0.078240332	-1.395971346
419967	420559	16	-	YPL071C	ORF-T	ORF-T	-0.248390294	-0.733935288	-0.079576307	-0.307424866	-0.470730104
422727	423911	16	-	BTS1	ORF-T	ORF-T	0.20628988	-0.673906186	-0.477314819	-0.420127271	0.322041597
424199	425143	16	-	YPL068C, YPI	ORF-T	ORF-T	0.125583819	-0.335432272	0.088881115	-0.340417829	0.381943066
425143	425895	16	-	YPL067C	ORF-T	ORF-T	0.19455184	0.316420854	0.184034718	-0.290432288	-0.640403693
428655	429623	16	-	CWC27	ORF-T	ORF-T	0.378135935	-0.317821718	-0.062575439	-0.187786145	-0.072720625
431095	431543	16	-	CUT898	CUTs	CUTs	1.540874929	-0.413038349	-0.597680744	0.193929615	0.373988661
445615	450543	16	-	PDR12	ORF-T	ORF-T	0.760170465	-0.086404935	-0.562081526	0.113515656	-1.173962398
451463	453231	16	-	SUR1	ORF-T	ORF-T	-0.078437684	-0.930079605	0.029023965	0.064385206	-0.248725784
453231	453495	16	-	unknown	no_ovlp2	SUTs	0.699860712	0.179668729	-0.123008515	-0.596217166	0.155066062
453495	454319	16	-	YPL056C	ORF-T	ORF-T	0.60855296	0.229681079	-0.040112901	-0.760939266	0.169739142
453919	454319	16	-	unknown	no_ovlp2	SUTs	-0.318950865	0.215740015	0.232422606	-0.217032796	0.447675787

454319	454991	16	-	LGE1	ORF-T	ORF-T	-0.139886693	-0.009644196	0.031873868	-0.192592593	0.242314073
457015	458527	16	-	KTR6	ORF-T	ORF-T	-0.132622313	-0.540963065	-0.00477381	-0.205757848	0.0808487
459215	459807	16	-	SUT817	SUTs	SUTs	1.828120098	0.032862095	-0.460819565	0.188389977	-0.595251339
460623	462159	16	-	MNN9	ORF-T	ORF-T	-0.345262802	-0.695218678	-0.654043175	-0.375657174	0.11296726
462327	463839	16	-	DIG1	ORF-T	ORF-T	-0.202100788	-0.660940063	-0.601723689	-0.017666946	-0.498738415
466367	466991	16	-	ELC1	ORF-T	ORF-T	0.033712772	-0.133808629	0.673265304	-0.383054664	0.828401165
472935	474903	16	-	SSN3	ORF-T	ORF-T	0.096130262	-0.509062606	-0.691131761	-0.037110337	-0.519778838
474903	476015	16	-	unknown	no_ovlp2	SUTs	-0.338792335	-0.624825728	0.133098421	-0.173115647	0.443245748
476015	479015	16	-	ISM1	ORF-T	ORF-T	-0.389630693	-0.496217063	0.039137921	-0.124116218	-0.088819034
481319	481991	16	-	EGD1	ORF-T	ORF-T	-0.220961984	-0.643405923	-0.420022843	-0.208123332	-0.341735837
481991	483047	16	-	SUT818	SUTs	SUTs	0.56369384	0.362820834	0.126796143	0.179245141	1.021410487
483047	486943	16	-	unknown	no_ovlp	SUTs	0.284056597	0.234696105	-0.271022398	-0.36467901	-0.307286876
489055	491599	16	-	SVL3	ORF-T	ORF-T	-0.077294636	-0.509248673	-0.820362922	-0.229304352	-0.207091813
491983	492911	16	-	PHO85	ORF-T	ORF-T	-0.271003434	-0.280648851	0.293027922	-0.000654206	0.080859622
492911	493303	16	-	CUT902	CUTs	CUTs	2.113989373	0.004075512	-0.525977498	-0.717232486	-0.709242106
496975	497703	16	-	CUT903	CUTs	CUTs	1.839514316	0.105655036	-0.672412739	0.193322324	0.950442311
500423	502247	16	-	SKS1	ORF-T	ORF-T	-0.092622379	-0.746071215	-0.610478703	-0.04002611	-0.244881152
502247	503559	16	-	YPL025C	other	other	0.592012047	0.502737273	-0.218403094	-0.469553724	-0.385238718
503231	503559	16	-	unknown	no_ovlp2	CUTs	1.162849596	0.001935966	-0.694073098	-0.646809504	-0.155313638
504311	506431	16	-	MET12	ORF-T	ORF-T	-0.077951647	-0.271487862	-0.196463416	0.137291384	-0.24287188
512223	514287	16	-	ULP1	ORF-T	ORF-T	0.103572007	-0.473480772	0.042614147	0.238406733	-0.180978008
516335	517215	16	-	VTC3	ORF-T	ORF-T	-1.352027132	0.731234022	0.736306557	2.063088933	2.263532216
518063	518551	16	-	unknown	no_ovlp2	SUTs	0.51348312	0.095631258	0.849697735	1.439438836	2.171883522
518551	520199	16	-	IRC15	ORF-T	ORF-T	0.628492954	-0.302640742	0.134144558	0.861435635	1.403430288
525487	526935	16	-	HST2	ORF-T	ORF-T	-0.653694428	-0.842695769	0.158589557	0.049360883	0.122688045
528831	529351	16	-	MRPS16	ORF-T	ORF-T	-0.371196145	-0.529352067	0.289070227	0.071933703	0.162379334
533455	534719	16	-	TAF3	ORF-T	ORF-T	-0.013631987	-0.316540019	0.393096512	-0.152974443	0.594548173
535615	538951	16	-	YPL009C	ORF-T	ORF-T	-0.017705138	-0.50177867	-0.545375223	-0.034212567	-0.300723821
541991	543863	16	-	TFC8	ORF-T	ORF-T	-0.448069817	-0.385014187	0.060629189	-0.187505588	0.15799833
543863	545655	16	-	unknown	no_ovlp	CUTs	1.272634029	0.365479853	-0.6542956	-0.338343075	-0.848334543
545655	550343	16	-	unknown	no_ovlp	SUTs	-0.322084608	0.296722001	0.165690497	0.017366748	0.414001245
550471	551711	16	-	LSP1	ORF-T	ORF-T	-0.174303559	-0.519632395	0.436926361	0.228654488	0.055320016
553231	554351	16	-	SNF8	ORF-T	ORF-T	-0.136538156	-0.305269853	0.148886735	-0.152155138	-0.184836113
556343	558055	16	-	SUT822	SUTs	SUTs	0.916073127	-0.092911495	-0.211399565	0.1347561	-1.299113967
562831	563815	16	-	YPR003C	ORF-T	ORF-T	-0.29547437	-0.644675049	0.419150257	0.365278609	-0.218021447
563959	565079	16	-	YPR004C	ORF-T	ORF-T	-0.302840463	-0.324837653	0.009405511	-0.078588444	-0.021183488
566751	567159	16	-	HAL1	ORF-T	ORF-T	0.681694794	-0.265076713	-0.323751868	-0.639652045	-1.049311147
567159	567863	16	-	ICL2	ORF-T	ORF-T	-0.037771499	0.894724585	1.149027085	0.624121172	-0.290648551
567863	568391	16	-	ICL2	ORF-T	ORF-T	0.112335992	0.138448621	1.137384351	1.176669228	-0.413479264
568391	569071	16	-	unknown	no_ovlp2	SUTs	-0.092812646	0.274995477	1.819698397	0.742671662	0.098441096
569071	572487	16	-	REC8	ORF-T	ORF-T	-0.388182481	0.241647525	0.904700531	0.429179419	0.276434411
570639	572487	16	-	unknown	no_ovlp2	SUTs	-0.137568237	0.424019263	0.374783816	-0.021254359	0.111088885
574055	575951	16	-	unknown	no_ovlp	CUTs	1.50241501	-0.194064576	-0.838856359	-0.048543671	-0.507004993
575951	577239	16	-	unknown	no_ovlp	SUTs	-0.141733657	0.436691955	0.326665348	0.070363077	0.312574633
577415	581247	16	-	RPA135	ORF-T	ORF-T	0.105130015	-0.227808634	-0.842852358	-0.172157256	-0.094222813
582223	582551	16	-	YPR010C-A	ORF-T	ORF-T	0.125786349	0.237656183	1.038457406	0.258288854	0.468696556
582791	584103	16	-	YPR011C	ORF-T	ORF-T	-0.309735289	-0.132955869	0.210296946	-0.083006222	-0.66016873
584831	585727	16	-	YPR013C	ORF-T	ORF-T	0.617905229	0.128705952	-0.500757161	0.17288257	-1.098649878
592183	593167	16	-	TIF6	ORF-T	ORF-T	0.047728444	-0.077841288	-0.447561743	0.02070989	-0.083761814
593295	593911	16	-	DSS4	ORF-T	ORF-T	0.796089106	0.367868571	0.035689287	-0.370179293	-0.178853614
600583	603559	16	-	AGC1	ORF-T	ORF-T	-0.182084699	-0.371591999	0.23659243	0.078086456	0.591699816
606023	607335	16	-	YPR022C	ORF-T	ORF-T	-0.122159246	-0.236868571	-0.095923111	-0.31184657	-0.214279423
608655	610071	16	-	EAF3	ORF-T	ORF-T	-0.464132742	-0.37203676	0.094276336	0.009418302	0.368179339
613503	614567	16	-	CCL1	ORF-T	ORF-T	0.015474916	-0.424604489	-0.022126863	-0.230485826	0.190198256
616863	618383	16	-	unknown	no_ovlp	CUTs	1.017046692	0.575963748	-0.989157001	-1.038477682	-1.320383423
622751	623183	16	-	CUT905	CUTs	CUTs	2.630468693	-0.143208939	-0.080231042	-0.549084884	0.082120229
624255	627007	16	-	APL4	ORF-T	ORF-T	0.177253419	-0.440082781	-0.278920452	-0.195850812	-0.306687287
630663	631239	16	-	unknown	no_ovlp	CUTs	2.008467627	0.463162637	-0.955319551	-0.181513845	0.561774145
637303	638999	16	-	HTS1	ORF-T	ORF-T	-0.044997382	-0.303387486	-0.318094	-0.228988607	-0.153454056
638999	639231	16	-	unknown	no_ovlp	CUTs	3.174309159	0.395100926	0.109565648	-0.712043174	0.188086428
639231	641095	16	-	unknown	no_ovlp	CUTs	1.009119691	0.471760818	-0.4931299	-0.041007588	1.946495384
643767	645247	16	-	SUT829	SUTs	SUTs	1.39695658	-0.464838833	-1.120784428	0.322657785	0.326611316
646439	647087	16	-	ERV2	ORF-T	ORF-T	-0.132578774	0.175673319	0.202884153	-0.186919872	-0.037331715
647087	648343	16	-	SUT831	SUTs	SUTs	1.613951373	-0.233644347	-1.843091681	-0.5043465	-0.121969115
652239	653775	16	-	PUF2	ORF-T	ORF-T	0.01842294	-0.251559085	0.296219711	0.336008974	0.131804445
655183	656591	16	-	MNI2	ORF-T	ORF-T	0.455475473	-0.276302035	-0.034260631	0.176769561	-0.124730684
658223	658959	16	-	unknown	no_ovlp	CUTs	1.338094754	-0.004955731	-0.620945737	-0.387996489	1.251119854
661055	664727	16	-	ATG11	ORF-T	ORF-T	0.021565738	0.142785931	0.696657895	0.049133259	0.822362345
665511	666039	16	-	NHP6A, YPR(	other	other	-0.521853136	-0.804405815	-0.466439509	-0.085716531	0.170406889
666039	667415	16	-	YPR053C	other	other	0.387522236	-0.511206808	-0.649656669	-0.112183631	-2.878235814
672727	673487	16	-	CUT908	CUTs	CUTs	3.622569959	0.285064185	-1.977993015	-0.44482262	-0.886324695
674687	675695	16	-	YPR059C, AR	other	other	0.485436747	-0.318085644	-0.492578493	-0.360206592	0.093688411
675879	676959	16	-	JID1	ORF-T	ORF-T	0.337902216	-0.278026497	0.909325382	0.212583938	-0.077548283
677599	678191	16	-	YPR063C	ORF-T	ORF-T	0.025338002	-0.170472902	-0.390776721	-0.042558118	0.120317745
678351	679295	16	-	SUT832	SUTs	SUTs	0.414032052	-0.113384817	0.09925487	-0.236311869	-0.264503522
681663	681959	16	-	SUT833	SUTs	SUTs	2.535256396	-0.008176563	-0.153134329	-0.570373186	-0.096837087
682775	684479	16	-	HOS1	ORF-T	ORF-T	0.100589083	-0.378166998	-0.419779305	-0.503475876	-0.539406367
684479	685503	16	-	SPE3	ORF-T	ORF-T	-0.20312778	-1.199410997	-0.908645889	-0.299176367	-0.523305534
691695	692487	16	-	LTP1	ORF-T	ORF-T	-0.029203334	-0.405916009	-0.061241501	-0.029933492	0.238855939
692671	694967	16	-	TKL1	ORF-T	ORF-T	-0.420939285	-0.003960321	-0.207625608	-0.057124149	-0.019308338
695647	696871	16	-	OPY2	ORF-T	ORF-T	-0.111030572	0.02070688	0.198326052	0.301033049	0.318560612
696871	697287	16	-	unknown	no_ovlp	CUTs	1.674326003	1.02319656	1.322074776	0.36717056	-1.167121082

699527	700023	16	-	CUT910	CUTs	CUTs	2.259498596	-0.135602317	-0.658191321	0.540325838	0.012390924
702087	704159	16	-	GRS2	ORF-T	ORF-T	-0.438301078	-0.050052602	0.279215135	-0.210919553	-0.027514187
704159	704655	16	-	DIB1	ORF-T	ORF-T	0.221669306	0.157441765	0.510742703	-0.345850494	0.611830853
708407	709887	16	-	YPR085C	ORF-T	ORF-T	-0.089782235	-0.470524792	0.156539259	0.079352828	0.04256073
711311	713055	16	-	SRP54	ORF-T	ORF-T	0.045486044	-0.206694545	-0.356687414	-0.003372951	0.013583506
716055	718551	16	-	YPR091C	ORF-T	ORF-T	-0.221699566	-0.473640422	0.101036026	0.026316469	-0.042384537
718551	719119	16	-	SNR51, SNR7	other	other	2.742570619	-0.385972894	-0.626317973	-0.472542499	-0.42867339
718903	719119	16	-	unknown	no_ovlp2	SUTs	0.70121822	-0.06784471	-0.137559496	-0.093796748	0.100349785
719367	720463	16	-	ASR1	ORF-T	ORF-T	0.494409666	-0.089655079	0.081384817	-0.314175463	-0.911831123
723095	724983	16	-	SYT1, YPR09(	ORF-T	ORF-T	-0.035361367	-0.336711362	-0.14732996	0.003675881	0.010379834
728759	729375	16	-	YPR098C	ORF-T	ORF-T	-0.444083588	-0.124430381	0.760794661	0.181015516	0.881823573
729375	730207	16	-	unknown	no_ovlp	SUTs	0.75363427	-0.284428238	0.149920905	0.599304195	0.175547328
731135	731775	16	-	RPL11A	ORF-T	ORF-T	-0.191690963	-0.124019684	-0.212305467	-0.153418567	-0.376401445
733239	736703	16	-	FHL1	ORF-T	ORF-T	-0.022505192	-0.199596143	-0.615237508	-0.16675402	0.234019204
736919	739647	16	-	COG4	ORF-T	ORF-T	0.187375524	-0.241402198	-0.330842504	-0.502137076	-0.04518237
741287	742135	16	-	YTH1	ORF-T	ORF-T	-0.222644498	-1.060045151	0.097957303	0.021845026	-0.047658974
745591	746847	16	-	RPC40	ORF-T	ORF-T	0.195463416	-0.302734118	-0.589852739	-0.184912788	-0.065368067
749175	751967	16	-	MRD1	ORF-T	ORF-T	0.478235343	0.018960587	-0.450245026	-0.068906588	0.497543498
752687	753007	16	-	CUT912	CUTs	CUTs	2.448401317	0.269516506	-1.499619168	-0.378429183	-0.473149329
758943	759439	16	-	CUT913	CUTs	CUTs	3.778443446	0.052798894	-1.141648873	-0.716803675	-0.608193846
766311	767431	16	-	CUT914	CUTs	CUTs	1.837887155	0.287738233	-1.424230745	-0.007590957	0.145541929
769071	769311	16	-	unknown	no_ovlp	SUTs	-1.25896097	-1.430283465	-0.394239355	-0.399033666	-0.035776374
769311	770687	16	-	SUT834	SUTs	SUTs	0.748642109	0.304929592	0.298681052	0.241157297	1.207403274
774311	775231	16	-	CLB5	ORF-T	ORF-T	-0.60826756	-0.434176248	-0.233945952	-0.29597303	0.935111971
776063	776999	16	-	unknown	no_ovlp	SUTs	0.531391341	0.143917557	0.806753035	0.468164309	-1.551307055
781319	781735	16	-	unknown	no_ovlp	SUTs	0.670912477	-0.840645355	-0.375521044	-0.489603724	-1.090894042
786911	787623	16	-	SUT837	SUTs	SUTs	-0.098241776	-1.311629703	-1.002276069	0.025525876	0.280155315
791127	792247	16	-	ANT1	ORF-T	ORF-T	1.310218111	1.032916012	-0.368109821	-0.596068184	0.619026001
793799	794519	16	-	YPR130C, NA	other	other	-0.330999084	-1.152064387	-0.082544642	-0.74072459	0.154102326
795887	797279	16	-	SPN1	ORF-T	ORF-T	-0.439303501	-0.395615622	0.040076906	0.262714941	0.413421101
801527	801959	16	-	CUT915	CUTs	CUTs	2.615471532	-0.388076484	-0.467676469	0.388274288	0.509742397
810847	812711	16	-	MEP3	ORF-T	ORF-T	-0.000299622	-0.451275017	-0.966601053	-0.532001187	-0.313096185
813071	814127	16	-	VPS66	ORF-T	ORF-T	-0.117098674	-0.581391417	-0.763864676	-0.326142572	-1.369684571
815623	817999	16	-	KAR3	ORF-T	ORF-T	-0.346418402	-0.166918017	0.082756395	-0.00615911	1.284997731
819687	821423	16	-	NOC4	ORF-T	ORF-T	0.326127693	-0.199597567	-0.206188678	0.125445572	0.119569918
821423	821735	16	-	CUT916	CUTs	CUTs	1.49702261	-0.638460901	-0.346715828	0.366961676	-0.956844386
824399	825439	16	-	YPR145C-A	ORF-T	ORF-T	0.02630398	0.2116509	0.73144745	1.471931375	-1.441200381
824919	825439	16	-	unknown	no_ovlp2	SUTs	0.73182097	-0.18628394	0.204361588	-0.213576757	-0.691672327
825439	826607	16	-	YPR146C, YP	ORF-T	ORF-T	0.37720055	0.311720863	0.078735655	0.045454629	-0.417010627
826719	828191	16	-	YPR148C	ORF-T	ORF-T	-0.2703198	-0.142252225	0.815303877	0.108505135	0.486801
828191	829551	16	-	SUT842	SUTs	SUTs	0.51081826	-0.322273015	0.120448327	-0.02076019	-0.534401358
830999	832151	16	-	unknown	no_ovlp	SUTs	0.454096	-0.07428147	1.59221945	1.190487915	0.611539786
834079	834303	16	-	CUT918	CUTs	CUTs	2.278837941	0.107443831	0.399812194	-0.023589938	0.139362152
835495	837463	16	-	NCA2	ORF-T	ORF-T	-0.192271584	-0.233386381	0.847354086	0.402313106	0.312207483
837647	840095	16	-	TPO3	ORF-T	ORF-T	0.293037398	-0.235172753	-0.183409622	-0.391901552	-0.137437266
840095	840767	16	-	unknown	no_ovlp	CUTs	1.456582509	0.578278525	-0.201884657	-0.369024993	-0.815580542
843815	850399	16	-	unknown	no_ovlp	CUTs	1.858385317	0.159139398	-0.935751754	0.138983316	0.260122803
864343	866415	16	-	SGV1	ORF-T	ORF-T	-0.12295243	-0.21112054	-0.202054967	0.025775424	0.010864944
866703	868311	16	-	ORC4	ORF-T	ORF-T	-0.029524944	-0.379710733	-0.350780973	-0.067120698	-0.208815201
868479	870039	16	-	TIF3	ORF-T	ORF-T	-0.156520255	-0.681259625	-0.483177177	-0.127529269	-0.197220697
874527	874871	16	-	CUT919	CUTs	CUTs	3.282256408	0.045762961	-0.168614469	0.347506302	0.163728189
876223	876647	16	-	MRP2	ORF-T	ORF-T	-1.321017638	-1.429453735	0.190516936	-0.118492766	0.301381806
876815	877663	16	-	MET16	ORF-T	ORF-T	0.091442102	0.20554751	-0.390135154	-0.429505054	-0.553939493
878031	878487	16	-	CUT921	CUTs	CUTs	3.359186122	0.344228151	-0.45143015	-0.379916595	0.295458281
881367	882919	16	-	unknown	no_ovlp	SUTs	0.702232092	0.421317108	0.312437224	0.590897294	-1.085735702
886479	887863	16	-	VPS4	ORF-T	ORF-T	0.200622342	-0.562434243	-0.683725923	0.005355129	-0.098424921
887863	888743	16	-	YPR174C	ORF-T	ORF-T	0.438909853	0.508817823	0.097909255	-0.365455447	0.388204674
890959	892087	16	-	BET2	ORF-T	ORF-T	-0.307977338	-1.223354853	-0.567005936	-0.289837269	-0.203537927
893751	895775	16	-	HDA3	ORF-T	ORF-T	0.274587029	-0.130516447	-0.11632729	-0.047656538	-0.157004338
897071	899735	16	-	SEC23	ORF-T	ORF-T	-0.388533481	-0.25039599	-0.489363058	-0.07172425	-0.158808523
900071	900495	16	-	CUT922	CUTs	CUTs	2.265716727	0.312582182	-0.477881937	-0.13635725	-0.034497297
900879	901543	16	-	CUT923	CUTs	CUTs	1.185696969	0.390467278	0.068666791	0.426032114	-0.593955238
909607	911015	16	-	PZF1	ORF-T	ORF-T	0.502804114	-0.138585723	-0.387753272	-0.777870877	0.171509607
911927	912503	16	-	MLC2	ORF-T	ORF-T	0.245562525	-0.224563959	0.219619422	-0.442087741	0.440463887
917055	919079	16	-	RPC82	ORF-T	ORF-T	0.349014556	-0.123879912	-0.592164237	-0.400212522	0.127280415
920935	924111	16	-	HPA2	ORF-T	ORF-T	0.722300862	0.867162509	1.125921178	0.530245291	-0.173800754
924111	927423	16	-	OPT2	ORF-T	ORF-T	-0.274117655	-1.559035032	-1.271964193	-0.480706942	-1.925122415
927423	928255	16	-	YPR195C	other	other	0.573199119	-0.274579513	-0.440952511	-0.408817514	-0.980118257
928255	928839	16	-	unknown	no_ovlp	SUTs	0.520672318	0.312605442	-0.613459627	-0.156480239	-0.386544779
933207	933735	16	-	CUT925	CUTs	CUTs	2.640205444	0.826582756	0.572314527	0.472524597	-2.733466318
935983	936863	16	-	SUT847	SUTs	SUTs	-0.311237091	-0.72034361	0.303003882	-0.140869418	-1.729720686
937999	939031	16	-	ARR1	ORF-T	ORF-T	-0.437932042	-1.129450952	-0.365106644	0.188693942	-0.843278009
22889	24761	16	+	SAM3	ORF-T	ORF-T	-0.629231561	-1.115896588	-0.579137914	0.011998232	-0.543481894
24977	26161	16	+	SAM4	ORF-T	ORF-T	-0.14751578	-0.757032821	-0.771613176	-0.196977382	-0.573487413
28617	30057	16	+	unknown	no_ovlp	SUTs	0.481141126	0.560627461	-0.248668489	-0.000463431	0.671257022
30057	30441	16	+	ATP15	ORF-T	ORF-T	-0.524883561	-0.459701874	0.650470496	-0.017002124	0.189637079
30441	32905	16	+	MDL2	ORF-T	ORF-T	-0.037185174	0.092271129	-0.134319278	0.006870547	-0.436355915
33017	35097	16	+	KAR9	ORF-T	ORF-T	-0.113182746	-0.175763891	-0.26228273	-0.233564725	0.014743286
35217	37873	16	+	PLC1	ORF-T	ORF-T	-0.103868	-0.163546922	0.007470993	0.339274273	0.242877763
38169	38833	16	+	ACM1	ORF-T	ORF-T	0.268701913	0.001190524	0.286823197	-0.257651028	0.684750665
39057	40145	16	+	DIM1	ORF-T	ORF-T	0.011547887	-0.046158448	0.037948751	0.296237771	0.777996823
40953	43145	16	+	DIP5	ORF-T	ORF-T	-0.444566065	-1.40047454	-0.190015403	0.151651616	-0.140566127

44641	45289	16	+	unknown	no_ovlp	CUTs	1.717681833	0.231635104	0.08783736	-0.685051155	1.04222779
46713	47273	16	+	CUT400	CUTs	CUTs	1.994487543	0.081151257	-0.029179853	-0.504626821	0.072969256
47273	48857	16	+	FUM1	ORF-T	ORF-T	-0.279727908	0.152460672	0.790514992	-0.007804687	0.075543731
49217	51137	16	+	YPL260W	ORF-T	ORF-T	0.052460521	-0.445764368	0.095472312	0.054403955	-0.497328209
52985	53505	16	+	SUT390	SUTs	SUTs	0.528185871	-0.05868463	0.071293282	0.294466254	-0.827236454
56457	63225	16	+	unknown	no_ovlp2	CUTs	1.385975066	0.73967853	-0.289010165	-0.652461559	-1.652730699
63225	64809	16	+	YPL257W	ORF-T	ORF-T	0.632432384	-0.292520991	-0.063428734	-0.134363749	-1.036293469
67705	69089	16	+	BBP1	ORF-T	ORF-T	-0.39403908	-0.386621883	-0.201202795	-0.606867293	0.207638774
69265	71009	16	+	HFI1	ORF-T	ORF-T	0.356721837	-0.924181263	-0.886779389	-0.395718074	-0.707430555
73769	75249	16	+	CUT401	CUTs	CUTs	0.540187565	0.442939583	0.422659567	-0.355548556	-0.38772393
75249	75969	16	+	SUT392	SUTs	SUTs	-0.109517909	-0.629712548	0.320264629	-0.149214348	-0.147849526
76705	79025	16	+	CUT402	CUTs	CUTs	2.13065854	-0.930259426	-1.233464671	-0.555505054	-0.280247941
84153	84505	16	+	unknown	no_ovlp	SUTs	0.107282653	0.376665941	0.040109266	0.17504425	0.848192656
84505	85537	16	+	CUT403	CUTs	CUTs	2.070421673	-0.356451847	-0.944730121	0.124397097	-0.720555779
85537	87009	16	+	YPL245W	ORF-T	ORF-T	1.47586467	-0.533575245	-1.054636109	-0.291779584	0.016297662
88441	90393	16	+	SRP68	ORF-T	ORF-T	0.083132219	-0.699464935	-0.506386412	-0.131141952	-0.269902728
95457	96417	16	+	SUT395	SUTs	SUTs	2.994044029	0.325801351	0.026103403	-0.35325156	0.316452721
99017	99425	16	+	YAR1	ORF-T	ORF-T	1.996811934	-0.216274632	-0.097363652	-0.900001969	-3.798247294
100441	101481	16	+	SUI3	ORF-T	ORF-T	-0.161940764	-0.723681977	-0.487141196	-0.175027749	-0.126750858
103145	104729	16	+	RVB2	ORF-T	ORF-T	0.003697335	-0.450890798	-0.469084921	0.15833441	-0.267046366
105801	107121	16	+	NSL1	ORF-T	ORF-T	0.254653246	-0.353189804	-0.27341704	-0.346410038	-0.309771052
107121	108265	16	+	SSO1	ORF-T	ORF-T	0.003451831	-0.638534485	-0.381880907	-0.078566141	0.006137176
108593	114409	16	+	FAS2	ORF-T	ORF-T	-0.484933514	-0.134866166	-0.663298183	0.000656617	-0.322944169
114921	115321	16	+	unknown	no_ovlp	SUTs	0.318942943	-0.316065155	0.964904284	1.062308721	0.73727504
115321	116449	16	+	YPL230W	ORF-T	ORF-T	-0.741023278	-1.228161673	2.125794163	1.908667299	1.211097853
117065	117553	16	+	YPL229W	ORF-T	ORF-T	0.170213985	0.020321792	0.621218477	0.223252768	0.254375459
118249	120161	16	+	CET1	ORF-T	ORF-T	-0.144301864	-0.704586956	-0.564709131	-0.119876146	-0.260515707
121705	125609	16	+	NEW1	ORF-T	ORF-T	0.132982508	-0.228269801	-1.074269092	-0.078713597	-0.111978253
125969	126577	16	+	YPL225W	ORF-T	ORF-T	0.05427729	-1.073352693	-0.290346458	0.067717903	-0.362049104
128249	129201	16	+	unknown	no_ovlp	SUTs	0.489281883	0.098282873	0.216020759	0.289134691	-0.144928063
130057	132801	16	+	FMP40	ORF-T	ORF-T	-0.610487581	0.241557586	1.278554041	1.215620847	1.165884319
132313	132801	16	+	unknown	no_ovlp2	SUTs	-0.59975647	-0.435831884	-0.106992157	0.156321826	-0.490275373
132801	135601	16	+	FLC1	ORF-T	ORF-T	0.027385093	-0.375282788	-0.342082484	-0.225981472	-0.517554912
135761	136569	16	+	RPL1A	ORF-T	ORF-T	-0.143276235	-0.578155161	-0.559071669	-0.455011497	-0.355458273
136721	138089	16	+	PCL8	ORF-T	ORF-T	-0.457569157	-0.710198067	0.430667228	0.164424297	0.191446833
138881	139537	16	+	SAR1	ORF-T	ORF-T	-0.40652723	-0.298583691	-0.241258534	-0.003494621	0.126976032
143401	143809	16	+	YPL216W	ORF-T	ORF-T	2.745474234	-0.804493215	-0.674625504	-0.794656679	-0.308679869
147393	148545	16	+	CBP3	ORF-T	ORF-T	-0.697647207	-1.111676511	-0.455735344	0.025348324	-0.385935755
150593	151417	16	+	LEA1	ORF-T	ORF-T	0.068736197	-0.445500347	-0.234944211	-0.224905143	0.01591769
153465	154273	16	+	NIP7	ORF-T	ORF-T	0.496740395	-0.398112242	-1.259896801	-0.426174455	0.013622197
157833	159665	16	+	RKM1	ORF-T	ORF-T	-0.10228546	-0.065302387	0.048291222	-0.310713737	0.618685785
159849	162449	16	+	TYW1	ORF-T	ORF-T	-0.098307416	-0.749430937	-0.497695113	0.127638067	0.286038124
164089	165889	16	+	HRR25	ORF-T	ORF-T	0.193191776	-0.564020835	-0.332309071	-0.083512471	-0.476494637
166097	167633	16	+	TPK2	ORF-T	ORF-T	-0.549375526	-0.860971907	0.377228844	0.177934514	0.561762741
169665	170425	16	+	CUT405	CUTs	CUTs	1.627774302	-0.298992103	-0.737734418	0.102370163	0.028137411
173113	173721	16	+	RPL7B, SNR5	ORF-T	ORF-T	2.549688346	-0.770540829	-0.130647334	-0.542404007	0.323928982
175017	175921	16	+	OXR1	ORF-T	ORF-T	-0.355439801	-0.708270931	0.765739418	0.465592397	-0.053403235
176225	179097	16	+	APL5	ORF-T	ORF-T	0.086125843	-0.232734022	0.011376671	-0.096706594	-0.164169337
179097	181385	16	+	DDC1	ORF-T	ORF-T	-0.32073202	-0.383062188	0.030293901	-0.521030971	-0.37142764
181385	182545	16	+	RSA1	ORF-T	ORF-T	0.601278796	-0.38669786	0.116660529	0.244884048	0.579470063
183481	184201	16	+	CUT407	CUTs	CUTs	1.80720311	0.478537561	-0.723977888	0.233296564	-0.305473315
189033	191001	16	+	GUP2	ORF-T	ORF-T	0.336296433	1.001452013	1.019891934	1.042527419	0.535720776
191401	192921	16	+	POS5	ORF-T	ORF-T	0.715077401	-0.041167136	0.325096202	-0.169137125	-0.192886132
199001	199529	16	+	YPL183W-A	ORF-T	ORF-T	-0.396517465	-1.076655647	-0.185416731	-0.122232287	-0.081869784
203265	205041	16	+	CTI6	ORF-T	ORF-T	0.089132537	-0.401945861	0.145901869	0.106899975	0.274300354
205249	208001	16	+	TCO89	ORF-T	ORF-T	-0.061522939	-0.310805504	-0.010233996	-0.057085065	-0.198011529
208001	208657	16	+	PPQ1	ORF-T	ORF-T	-0.01186397	-0.103483119	0.148096297	0.006339738	0.058530946
212105	212929	16	+	CBC2	ORF-T	ORF-T	-0.117323559	-0.883416652	-0.241993637	0.096764976	-0.415598389
214329	215777	16	+	unknown	no_ovlp	SUTs	0.105292472	0.185454867	0.145096731	-0.147511581	-0.450112711
215777	216481	16	+	unknown	no_ovlp	CUTs	2.284154442	0.434151903	-0.845627876	-0.134917172	0.355399645
218753	220185	16	+	SPT14	ORF-T	ORF-T	0.427520408	-0.245170893	-0.34572601	-0.161236521	-0.327799887
223105	224169	16	+	MRPL40	ORF-T	ORF-T	-0.501233608	-0.511013656	-0.337966755	-0.159813941	-0.728694613
226041	226617	16	+	CUT409	CUTs	CUTs	2.126307127	0.203644917	0.00317431	0.292416945	-0.247757775
228217	228937	16	+	DAP1	ORF-T	ORF-T	-0.524663469	-0.372147498	0.353756618	0.286822783	0.374781627
231145	232569	16	+	YPL168W	ORF-T	ORF-T	-0.011693986	-0.815800483	0.18128469	-0.16004996	-0.39061055
237313	239145	16	+	ATG29	ORF-T	ORF-T	-0.345562563	-0.462191861	0.525227838	0.136835382	-0.047010274
243233	245089	16	+	SUT397	SUTs	SUTs	0.728911869	-0.227477886	0.273204343	-0.362497683	0.779822482
246921	250449	16	+	CDC60	ORF-T	ORF-T	-0.159812197	-0.20813465	-0.446416831	0.088231023	-0.316637078
252065	252569	16	+	CUT411	CUTs	CUTs	3.139960902	0.176987352	-0.080099292	0.35618353	1.068266169
254721	256185	16	+	TGS1	ORF-T	ORF-T	-0.007204804	-0.456658798	-0.394099022	0.049040392	0.510910144
264977	266241	16	+	YPL152W-A,	other	other	0.229605251	-0.192049933	0.235838256	0.030171764	0.162886431
267769	271049	16	+	YPL150W	ORF-T	ORF-T	-0.037849338	-0.274200249	-0.050444494	-0.12032899	0.17764094
271265	272233	16	+	ATG5	ORF-T	ORF-T	0.391050709	-0.462196209	-0.55350541	-0.393870776	0.150057038
272233	273225	16	+	unknown	no_ovlp2	SUTs	0.821266471	0.144370857	-0.685080603	-0.660633036	0.437703206
273225	275873	16	+	PXA1	ORF-T	ORF-T	-0.256089829	0.215501559	1.393015529	0.278125866	1.323582734
280457	281017	16	+	POC4	ORF-T	ORF-T	0.576093648	-0.448755472	-0.022825456	-0.08081965	-0.050743754
282137	282673	16	+	RPL33A	ORF-T	ORF-T	0.784016166	-0.946616562	-0.812946662	-1.11546949	-0.15130484
291281	291793	16	+	CUT412	CUTs	CUTs	1.599454889	0.249422114	0.069333027	0.419682923	0.866908068
295889	297017	16	+	unknown	no_ovlp	SUTs	-0.020831678	0.33161041	-0.150835409	-0.22896878	0.273906404
297017	297497	16	+	SUT400	SUTs	SUTs	-0.390402169	-0.243217695	-0.396885077	-0.423942362	-1.036725825
297497	298417	16	+	ISU1	ORF-T	ORF-T	-0.221040507	-0.486708138	0.512755961	0.103701164	0.553828562
298553	300561	16	+	unknown	no_ovlp	SUTs	0.927853311	0.194332042	0.72736109	0.592853213	1.65545315

301601	302633	16	+	COX11	ORF-T	ORF-T	-0.158770137	-0.239495862	0.213839556	-0.332062827	0.209458033
303065	304113	16	+	RPL5	ORF-T	ORF-T	-0.213824927	-0.117326208	-0.052422256	-0.200232755	-0.052217013
304241	305249	16	+	unknown	no_ovlp	SUTs	-0.048356123	0.190490181	-0.038284889	0.217580402	-0.030650998
305425	306257	16	+	TAF14	ORF-T	ORF-T	0.102381368	-0.287883567	-0.3645488	0.013143379	-0.098706819
309217	310185	16	+	unknown	no_ovlp	SUTs	0.596607744	0.318814255	-0.58965068	-0.629652969	-0.515509248
310185	313001	16	+	NAN1	ORF-T	ORF-T	0.216103419	-0.311676033	-0.610241975	0.108085471	0.300141046
313241	316553	16	+	KAP120	ORF-T	ORF-T	0.45145128	-0.234270675	-0.353754475	0.003674483	0.132235105
316737	317569	16	+	SPC29	ORF-T	ORF-T	-0.168619388	-0.119245245	0.610060585	-0.35453012	1.2138428
319169	319505	16	+	CUT414	CUTs	CUTs	2.232549231	0.04694032	0.666724854	0.689419654	0.827195504
322169	326345	16	+	VPS30	ORF-T	ORF-T	-0.23757262	-0.712414716	0.046618152	-0.260670822	0.426240458
323745	326345	16	+	unknown	no_ovlp2	CUTs	2.051427468	0.328198222	-0.786331588	-0.653939027	-0.837536867
326553	327777	16	+	MRP51	ORF-T	ORF-T	-0.203702736	-0.603807695	0.032466343	0.375784689	-0.036435527
329425	331833	16	+	HOS3	ORF-T	ORF-T	-0.228000045	-0.241756616	0.441843577	0.066024471	0.806253955
335897	336545	16	+	YPL114W	other	other	2.341408368	-0.38829501	-0.771205857	-0.241650782	-0.289396453
339905	341057	16	+	CAR1	ORF-T	ORF-T	-0.23343141	-0.810097109	0.508748841	0.095808569	1.147608628
348507	350051	16	+	YPL108W, YP	ORF-T	ORF-T	0.184210262	-0.097474572	0.124481421	-0.065246393	0.071598823
352611	352995	16	+	SUT403	SUTs	SUTs	2.551719952	-0.389661415	-0.756291283	-0.321412327	0.180592413
355699	357803	16	+	MSD1	ORF-T	ORF-T	-0.602538229	-0.474887436	0.254755183	-0.315433193	0.320690954
360179	361691	16	+	ELP4	ORF-T	ORF-T	0.277270656	0.061966183	-0.115000883	-0.080972363	0.467952535
361835	363403	16	+	ATG21	ORF-T	ORF-T	0.208727926	-0.551601837	0.049922371	0.098962407	-0.179272263
364931	366531	16	+	MSY1	ORF-T	ORF-T	-0.261900429	-0.569094465	-0.043969155	-0.410653119	-0.350147003
366915	368083	16	+	PNG1	ORF-T	ORF-T	-0.156183843	-0.60702729	0.119137994	-0.066760259	-0.30548297
370907	373155	16	+	NOG1	ORF-T	ORF-T	0.062790031	-0.227601174	-0.704439163	0.07104905	0.04909481
373603	375499	16	+	SSU1	ORF-T	ORF-T	-0.393256345	-0.877710342	0.088173281	0.811135378	0.371463973
375251	375499	16	+	unknown	no_ovlp2	CUTs	1.118613931	0.094158719	0.129490116	-0.050256278	-0.335788526
375499	377059	16	+	GLR1	ORF-T	ORF-T	-0.127740123	-0.379807575	0.116769996	0.029678851	-0.084253122
379179	381539	16	+	SUT406	SUTs	SUTs	0.492007121	0.261923611	-0.55042452	-0.375681898	1.838209451
381539	383075	16	+	YPL088W	ORF-T	ORF-T	0.729696157	-0.188032554	-0.248631954	0.101522941	-0.376009163
383347	384547	16	+	YDC1	ORF-T	ORF-T	-0.244645917	-0.490504993	-0.29437965	0.123883313	-0.080831034
386899	388363	16	+	SEC16	ORF-T	ORF-T	-0.175918749	-0.257076449	-0.97607805	-0.302417529	-0.565887718
393995	396715	16	+	BRO1	ORF-T	ORF-T	-0.131815561	-0.405408301	0.04217584	-0.058795918	-0.237858903
397099	398147	16	+	unknown	no_ovlp	CUTs	1.475276425	0.392301269	0.142339396	0.109126441	0.932602963
404915	406243	16	+	RPS9A	ORF-T	ORF-T	1.941426276	-0.946523695	-0.491914111	-0.554988627	-0.382489519
406635	407123	16	+	RPL21B	ORF-T	ORF-T	0.825207722	-0.545453541	-1.130606264	-1.015508007	-0.702037649
409091	409659	16	+	CUT417	CUTs	CUTs	2.933662431	0.680536707	-0.947442762	-0.492131037	-0.147223747
410307	410947	16	+	GPI2	ORF-T	ORF-T	0.128404207	-0.230524033	-0.179194217	-0.213257889	-0.179669646
412299	415579	16	+	GCR1	ORF-T	ORF-T	1.046785477	-0.529713124	-1.047133927	-0.351944687	-0.676403326
415755	418139	16	+	YTA6	ORF-T	ORF-T	-0.052800641	-0.655698102	-0.029310018	-0.147702841	-0.094727711
418355	420043	16	+	UBP16	ORF-T	ORF-T	-0.027725871	-0.259868811	0.121980924	-0.439856176	-0.241379551
420763	423019	16	+	MUK1	ORF-T	ORF-T	0.066703561	-0.404633958	0.207620422	-0.047852293	-0.406189974
425819	426291	16	+	YPL066W	ORF-T	ORF-T	0.626934412	-0.540406906	0.590219378	0.135501536	-0.162782431
427915	428739	16	+	VPS28	ORF-T	ORF-T	-0.270987216	-0.471788561	0.612790389	-0.219813864	0.208310421
429819	431515	16	+	TIM50	ORF-T	ORF-T	-0.517692572	-0.660648231	-0.339731633	-0.072941756	-0.240198824
432331	432547	16	+	CUT418	CUTs	CUTs	2.238859929	0.048222005	-0.28109402	-0.409709602	-0.85185319
432547	434211	16	+	ALD6	ORF-T	ORF-T	-0.605463141	-0.254661849	0.495670551	0.276191458	0.095084777
434211	435811	16	+	LPE10	ORF-T	ORF-T	0.228716706	-0.182976659	0.290120273	0.38614458	0.380447696
444531	445227	16	+	GRX5	ORF-T	ORF-T	-0.589158595	-0.561849937	0.201399263	-0.149242226	-0.042191045
451579	452211	16	+	CUT419	CUTs	CUTs	2.12291147	0.543171231	-0.115581199	0.012419495	-0.830457106
453411	454987	16	+	SUT409	SUTs	SUTs	1.149249362	-0.056231856	-0.024461053	0.138277097	-0.183129507
454987	455611	16	+	unknown	no_ovlp2	CUTs	1.330780719	-0.27102439	-0.60361972	-0.082943861	-0.568861043
455611	457083	16	+	LEE1	ORF-T	ORF-T	-0.153650417	-0.297913531	1.631659345	1.342050165	-1.26556796
458795	459835	16	+	OAZ1	ORF-T	ORF-T	0.07139898	-0.531455319	-0.134033497	-0.374806372	0.393868111
459947	460715	16	+	ARL3	ORF-T	ORF-T	0.476735632	-0.611089567	-0.601522367	-0.505165561	-0.133661238
462451	464083	16	+	unknown	no_ovlp	SUTs	0.076940692	0.38031951	0.195222414	0.172813667	1.286994175
464083	464363	16	+	unknown	no_ovlp	SUTs	0.719015817	0.710805386	0.565736609	-0.147557062	-0.860167268
464363	465763	16	+	CAM1	ORF-T	ORF-T	-0.462831009	-0.231641442	-0.101053198	-0.012125575	-0.178280135
465891	466587	16	+	SGF11	ORF-T	ORF-T	-0.178395013	-0.604262202	-0.012479222	0.038052239	0.215658093
466747	467171	16	+	unknown	no_ovlp	SUTs	-0.203554885	0.205312336	0.267235255	0.055633223	0.255761373
467171	469891	16	+	VPS16	ORF-T	ORF-T	-0.014653787	-0.326598109	-0.06440241	-0.591360083	0.193041863
469891	472107	16	+	NOP4	ORF-T	ORF-T	0.271631216	0.085739985	-0.756687046	0.087296324	0.258029569
475171	475787	16	+	CUT422	CUTs	CUTs	1.716437802	-0.090167449	-0.45777706	-0.020259885	-0.461513863
479163	480259	16	+	YPL039W, YP	ORF-T	ORF-T	-0.532712846	-0.616231378	0.477727498	0.036909435	0.614092194
480515	481163	16	+	MET31	ORF-T	ORF-T	0.360822468	-0.417431475	-0.753703246	-0.479774971	-0.153921812
482507	483011	16	+	PMA2	ORF-T	ORF-T	0.167840156	-1.118649618	0.425784259	0.517599548	1.015025925
486411	487363	16	+	YPL034W	ORF-T	ORF-T	0.683645219	-0.132675319	-0.395112769	-0.285784596	-0.755855047
491995	492811	16	+	SUT412	SUTs	SUTs	1.740888166	-0.720084649	-1.524711191	-0.346633206	-0.049010104
493491	495315	16	+	TRM44	ORF-T	ORF-T	0.389628525	0.069880845	-1.323273896	-0.60680297	-0.038621806
495499	497723	16	+	SUV3	ORF-T	ORF-T	-0.112471432	0.050168021	-0.109058615	-0.317407735	0.137089554
498051	499387	16	+	ERG10	ORF-T	ORF-T	-0.138705919	-0.255270921	-0.379219097	0.28486962	0.116669394
503259	504355	16	+	RMI1	ORF-T	ORF-T	0.144301927	-0.9457359	0.28948435	0.118080455	0.396937243
506611	510211	16	+	RAD1	ORF-T	ORF-T	0.090952302	-0.40587801	-0.182441925	-0.05809073	-0.371335025
514507	515931	16	+	unknown	no_ovlp	CUTs	2.029347058	0.425200771	-0.543686968	-0.657773933	-0.32459514
517475	517739	16	+	CTF19	ORF-T	ORF-T	0.917008281	-0.091935766	0.126338676	1.01611901	0.512481595
520627	525059	16	+	SWI1	ORF-T	ORF-T	0.740156783	-0.314942075	-0.524572748	-0.486426225	-1.009871127
527523	528979	16	+	YPL014W	ORF-T	ORF-T	-0.674465182	-0.985678955	-0.830298545	-0.248009141	-0.046293495
529611	533515	16	+	RRP12	ORF-T	ORF-T	0.098738584	-0.329209927	-0.965193541	0.075410167	0.034135659
534963	535731	16	+	RET3	ORF-T	ORF-T	-0.373864462	-0.575038853	-0.062056599	-0.160932248	0.286019201
539187	542051	16	+	CHL1	ORF-T	ORF-T	-0.296661989	-0.316065475	0.043944713	-0.193302446	0.18049523
544059	544667	16	+	SUT414	SUTs	SUTs	0.120241993	-0.596092377	0.076465719	0.083335592	-0.791497248
544667	545491	16	+	NCR1	ORF-T	ORF-T	-0.838212855	-0.140462844	0.712941871	0.493180617	-0.545958489
548219	548491	16	+	unknown	no_ovlp2	SUTs	-0.786529325	-0.654706586	0.460742284	0.085507041	0.069952212
548491	550339	16	+	AEP3	ORF-T	ORF-T	-0.321765162	-0.626053303	0.347902959	0.202679337	-0.319974496

551619	552011	16	+	unknown	no_ovlp	SUTs	-0.215533475	0.416782531	-0.13388928	0.00207563	-0.070978342
552011	553435	16	+	ULA1	ORF-T	ORF-T	0.53328822	-0.689272562	0.174318052	0.107432038	0.004935252
554595	555779	16	+	HAT1	ORF-T	ORF-T	-0.155113918	-0.834288673	-0.76191707	-0.440296173	-0.481390512
555779	556339	16	+	unknown	no_ovlp2	SUTs	-0.046607842	0.295506666	-0.001748495	-0.106447538	-0.376024821
556339	558387	16	+	CIT3	ORF-T	ORF-T	-0.356157618	0.747670073	2.644011429	1.116316499	0.021547978
557979	558387	16	+	unknown	no_ovlp2	SUTs	-0.82054617	0.05272089	0.337818209	0.140318382	-0.289452623
558387	560115	16	+	PDH1	ORF-T	ORF-T	0.682462831	0.712151729	1.559794415	0.891497048	0.344850897
565291	565779	16	+	CUT423	CUTs	CUTs	2.019807408	0.205668471	-0.472609568	-0.311264541	-0.957531855
567219	568923	16	+	unknown	no_ovlp	CUTs	1.921509311	0.151019939	0.182614644	0.523055558	-0.518063239
572267	572707	16	+	unknown	no_ovlp	SUTs	0.125558289	-0.601880755	-0.564465595	-0.845683611	-0.117321244
572707	575219	16	+	HAA1	ORF-T	ORF-T	0.338948933	-0.61956358	-0.385873163	-0.297940878	-0.61525598
576467	577523	16	+	SUT2	ORF-T	ORF-T	0.416682215	-0.851237147	-0.913507197	-0.262077879	-0.912176724
581523	581987	16	+	CUT424	CUTs	CUTs	2.527263108	0.131679338	-0.277002393	-0.487628973	-0.118273312
583027	583627	16	+	SUT416	SUTs	SUTs	2.88333054	-0.171535684	-0.39559384	-0.180610321	-0.017382667
586571	589475	16	+	SUT417	SUTs	SUTs	1.520385492	0.184503617	-0.402184472	-0.448633644	-1.614657277
593419	594371	16	+	unknown	no_ovlp	SUTs	0.082940348	0.115071881	-0.048546137	-0.029978008	0.28846419
594371	596403	16	+	RLF2	ORF-T	ORF-T	-0.208521027	-0.126681036	-0.187781092	-0.172241005	0.462124071
596683	599707	16	+	CDC54	ORF-T	ORF-T	-0.127535545	-0.356203197	-0.151799928	-0.161263508	0.217446146
599827	600291	16	+	ATP20	ORF-T	ORF-T	0.523855157	0.111346624	0.496047151	0.048285084	-0.341237294
607539	608771	16	+	SUT420	SUTs	SUTs	2.169064934	0.201249484	-0.60432635	-0.732539222	-0.702585532
610307	613291	16	+	YME1	ORF-T	ORF-T	-0.222431361	-0.393254451	-0.039803132	-0.017892125	-0.123881112
614779	619027	16	+	ATH1	ORF-T	ORF-T	-0.327966115	-0.236646359	1.423354877	0.85674417	1.32809398
620187	621323	16	+	SUT421	SUTs	SUTs	-0.151380547	-0.912146496	0.52392888	0.374496508	-0.776441582
622995	623723	16	+	YOP1	ORF-T	ORF-T	0.801591136	-0.138375806	-0.375792812	-0.003066919	-0.33439058
627867	631587	16	+	CSR2	ORF-T	ORF-T	-1.284268658	-0.269459491	2.841713888	2.897058054	0.172238465
631339	631587	16	+	unknown	no_ovlp2	SUTs	0.087793262	0.732147499	-0.162329102	-0.008818988	0.050645391
631587	633795	16	+	NTO1	ORF-T	ORF-T	-0.14796798	0.049256808	0.030505242	-0.150174673	1.062007167
634091	637283	16	+	SRO7	ORF-T	ORF-T	0.255660767	-0.352286127	-0.293580729	-0.334627428	-0.51422728
639483	641179	16	+	ARP7	ORF-T	ORF-T	-0.308640903	-0.495801899	-0.587267635	-0.219115686	0.316337417
642091	643531	16	+	GLN1	ORF-T	ORF-T	0.035431771	0.054316204	-0.215890654	-0.229297844	-0.147427669
643787	645355	16	+	VMA13	ORF-T	ORF-T	0.061356386	-0.210239184	-0.017510907	-0.182489486	-0.17998897
645883	646443	16	+	YPR036W-A	ORF-T	ORF-T	-0.170139009	-0.140793743	0.209616955	0.102677806	-0.23516256
647315	648443	16	+	YPR039W, TI	ORF-T	ORF-T	-0.213854997	0.064104665	0.545725997	0.251092003	0.233410599
648595	650075	16	+	TIF5	ORF-T	ORF-T	-0.152670752	-0.883028236	-0.829277776	-0.104814608	-0.280880997
654115	654579	16	+	RPL43A	ORF-T	ORF-T	0.254621375	-0.70490638	-1.109921883	-0.475239151	-0.169559427
655059	656771	16	+	unknown	no_ovlp	SUTs	-0.039374575	0.378247506	-0.102286219	0.006438379	0.498240764
656771	657467	16	+	unknown	no_ovlp2	SUTs	0.09171711	-0.572524563	0.163507334	-0.484962923	0.6822776
657467	659171	16	+	MSF1	ORF-T	ORF-T	-0.558099992	0.1581461	0.897031995	-0.108305351	0.146496317
659171	661123	16	+	TAH18	ORF-T	ORF-T	0.397621356	0.050424365	-0.358461016	-0.532526135	0.621785295
664939	665603	16	+	MAK3	ORF-T	ORF-T	0.537600512	-0.523850035	-0.54020394	-0.361344384	-0.233269392
667635	670867	16	+	SEC8	ORF-T	ORF-T	-0.008026945	-0.391103893	-0.342971365	-0.094813111	-0.48409483
671123	673547	16	+	TFB4	ORF-T	ORF-T	-0.306981909	-0.87572399	-0.097820724	0.233767758	0.302087831
672331	673547	16	+	unknown	no_ovlp2	SUTs	-0.560585767	-0.907649302	-0.401328207	-0.163035782	0.145634972
673683	674755	16	+	YMC1	ORF-T	ORF-T	-0.023464648	-0.580332449	-0.673912945	-0.301401955	-0.261450202
675883	676291	16	+	CUT428	CUTs	CUTs	2.918834941	0.549032208	-0.908324417	-0.173713855	0.139368942
677155	677771	16	+	FCY1	ORF-T	ORF-T	0.077330481	-0.302403063	0.190234816	-0.045508697	0.549233492
679619	681067	16	+	ROX1	ORF-T	ORF-T	0.467880781	-0.440243778	-0.202051465	-0.207056487	-0.264369837
681067	682187	16	+	UBA3	ORF-T	ORF-T	0.025194482	-0.453593832	0.163563037	-0.065546197	-0.279932489
682187	682939	16	+	ISA2	ORF-T	ORF-T	-0.201424448	-0.545142179	0.153759798	-0.221398201	-0.143528437
685555	685883	16	+	unknown	no_ovlp	SUTs	0.571161248	-0.770538359	-0.492509077	-0.139951784	-0.430160683
685883	687931	16	+	MED1	ORF-T	ORF-T	0.235552713	-0.455685117	-0.330864643	-0.189612248	-0.095796418
688163	689331	16	+	YPR071W	ORF-T	ORF-T	0.800233301	-0.048849113	-0.436860486	-0.258455345	-0.152756869
690011	691931	16	+	NOT5	ORF-T	ORF-T	-0.06497762	-0.569843004	-0.357176634	-0.036911399	-0.198907139
695355	695691	16	+	CUT430	CUTs	CUTs	1.348682521	0.11708419	0.669333202	-0.53916514	-0.465866066
697123	698491	16	+	CUT431	CUTs	CUTs	2.374210157	0.560699124	0.665490412	0.043384427	-0.824776728
698795	700115	16	+	MRL1	ORF-T	ORF-T	-0.273496069	-0.276017249	0.21840535	0.056469439	-0.354384483
700563	702099	16	+	TEF1	ORF-T	ORF-T	-1.005785883	-2.148172604	0.096650872	0.500714226	-0.526219852
704939	706755	16	+	MDM36	ORF-T	ORF-T	0.345723293	-0.082038819	0.253276052	-0.365536824	0.173635005
706875	708531	16	+	YPR084W	ORF-T	ORF-T	0.440805089	0.171499561	-0.369000973	-0.028674629	-0.0964683
710067	711411	16	+	SUA7	ORF-T	ORF-T	0.226233124	-0.501069087	-0.214825496	-0.016010709	0.02599075
713243	716115	16	+	YPR089W	ORF-T	ORF-T	-0.025400211	-0.235232952	-0.059911851	-0.142389939	0.088861997
718667	719595	16	+	unknown	no_ovlp	SUTs	-0.086175876	-0.255031114	0.13018262	0.204522366	-0.073193944
719595	720115	16	+	CUT432	CUTs	CUTs	3.139427472	0.375022706	-0.654672759	0.063918438	-0.097025636
720611	721027	16	+	RDS3	ORF-T	ORF-T	0.940955595	-0.265185746	-0.265407756	-0.536562186	-0.975742766
725171	728691	16	+	YPR097W	ORF-T	ORF-T	0.039217675	-0.439348429	-0.42078846	-0.170770926	-0.140385129
729771	730307	16	+	MRPL51	ORF-T	ORF-T	-0.804326753	-0.737989591	-0.062547104	-0.217012559	0.181317079
730475	731163	16	+	SNT309	ORF-T	ORF-T	0.336114033	-0.429420319	-0.088329622	-0.62782344	0.183954047
732259	733275	16	+	PRE2	ORF-T	ORF-T	-0.006807749	-0.373838664	0.266545188	-0.184650656	-0.070988184
733411	734291	16	+	unknown	no_ovlp	SUTs	-0.301019408	-0.071609541	0.219014946	-0.034239608	0.693835172
740035	741483	16	+	ISR1	ORF-T	ORF-T	0.409771511	-0.277917704	0.046685876	-0.036789081	0.246185701
742371	743955	16	+	RPN7	ORF-T	ORF-T	-0.086898901	-0.593541064	0.056001342	-0.091162576	0.0234063
744483	745635	16	+	YPR109W	ORF-T	ORF-T	-0.635186366	-0.873261196	-0.041856901	-0.03396577	-0.128088788
747299	749227	16	+	DBF20	ORF-T	ORF-T	-0.343119623	-0.625829894	0.320527682	-0.232820148	0.511091883
752219	753019	16	+	PIS1	ORF-T	ORF-T	-0.14131106	-0.236218264	0.07409884	0.067313644	0.445848813
753259	754363	16	+	YPR114W	ORF-T	ORF-T	-0.758563738	-1.140524301	-0.802507959	-0.036653498	-0.256872027
754683	758363	16	+	YPR115W	ORF-T	ORF-T	-0.195202449	-0.457587037	0.104414977	0.369586568	0.352169739
758659	759883	16	+	YPR116W, YF	ORF-T	ORF-T	0.280985597	-0.123484828	-0.698595568	-0.376122744	-0.259981088
767731	769083	16	+	MRI1	ORF-T	ORF-T	-0.702333865	-0.707808028	-0.486119535	-0.142337238	0.238897212
771275	773587	16	+	CLB2	ORF-T	ORF-T	0.109120598	-0.384234318	-0.646792067	-0.208859484	0.333814958
777555	780347	16	+	YPRWdelta1(	other	other	0.918791449	-0.251217906	-0.213950754	-0.310290207	-1.418415372
782003	784379	16	+	unknown	no_ovlp2	SUTs	0.366608739	-0.34043083	-0.61245397	-0.491339768	-0.337904984
784379	785819	16	+	AXL1	ORF-T	ORF-T	0.572844605	-0.254725534	-0.76236255	-0.563583933	-0.475127254

786043	787651	16	+	CTR1	ORF-T	ORF-T	0.424019797	0.587014073	0.246422539	-0.308716049	-1.235500505
787875	789499	16	+	YLH47	ORF-T	ORF-T	0.209406835	-0.404637971	-0.948529006	-0.436055085	-0.496465662
790003	791195	16	+	YPR127W	ORF-T	ORF-T	-0.26711304	-0.297605022	0.305573028	0.114781178	0.151602471
792659	793811	16	+	SCD6	ORF-T	ORF-T	-0.323047033	-0.232290236	-0.327300299	0.079153909	0.196184238
795027	795395	16	+	RPS23B	ORF-T	ORF-T	0.36292817	-1.101184398	-1.030357416	-1.1275741	-0.097185419
797507	797875	16	+	TOM5	ORF-T	ORF-T	-0.760575139	-0.435655727	0.38809927	0.070597022	0.033266758
798051	798963	16	+	MSS18	ORF-T	ORF-T	-0.127346491	0.063149307	0.6834685	-0.078508011	0.537017243
799211	802067	16	+	CTF4	ORF-T	ORF-T	-0.274130982	-0.112569672	-0.268138758	-0.077084429	0.128561481
802315	804163	16	+	RRP9	ORF-T	ORF-T	0.051698263	-0.220095132	-0.924462173	-0.268273338	0.39238936
804571	811795	16	+	unknown	no_ovlp	CUTs	1.729873382	0.320775583	0.504135972	0.051200862	-0.71150199
812907	813315	16	+	unknown	no_ovlp	SUTs	-0.535060229	-0.69164565	0.081127084	-0.337477435	0.403000774
814307	815603	16	+	TAZ1	ORF-T	ORF-T	-0.438570058	-0.953651335	-0.21590202	-0.214274309	-1.199931722
818307	819227	16	+	RRP15	ORF-T	ORF-T	0.704312927	-0.368367213	-0.499670269	0.012202713	0.135885013
821731	822163	16	+	SNR45	other	other	5.925074101	-0.503494945	-0.390926666	-0.321184724	-0.072925231
822299	822539	16	+	unknown	no_ovlp	CUTs	1.784644858	0.458911355	-0.556829276	-0.70213952	-0.009501101
822539	824483	16	+	ASN1	ORF-T	ORF-T	-0.274296915	-0.417602469	-1.08686507	-0.365786967	-0.671832371
825435	826043	16	+	unknown	no_ovlp	CUTs	2.25115654	0.331639531	0.057466937	0.460373585	-0.974973707
828067	829523	16	+	SUT425	SUTs	SUTs	0.297563877	-0.664484733	-0.111106436	0.107651058	0.154813618
829523	829731	16	+	unknown	no_ovlp	SUTs	0.636577399	-0.687304502	0.037781904	0.010667985	-1.642179744
829731	830827	16	+	NCE102	ORF-T	ORF-T	-0.398783702	-0.607620571	0.347471046	-0.007404102	0.293612418
830827	831067	16	+	YPR150W	other	other	0.718000711	0.034946924	0.138899928	-0.017551855	-0.092811993
833123	833651	16	+	unknown	no_ovlp2	CUTs	1.722107686	0.148721895	1.297381834	0.768445501	1.315924029
833651	834547	16	+	YPR153W	ORF-T	ORF-T	-0.543935652	-0.246857407	0.332703227	0.150221082	0.521673936
834547	835315	16	+	PIN3	ORF-T	ORF-T	-0.232880752	-0.815555931	-0.106887971	0.355970887	-0.099413099
841235	842779	16	+	YPR157W	ORF-T	ORF-T	1.414872442	0.520574265	-0.709789538	-0.44369284	-0.883011356
843195	844171	16	+	YPR158W	ORF-T	ORF-T	1.227560943	0.150564742	0.199179476	-0.399757863	0.525902992
857363	860035	16	+	KRE6	ORF-T	ORF-T	0.067609807	-0.387974246	-0.447994697	-0.311189276	-0.250858928
861155	864155	16	+	GPH1, YPR16	ORF-T	ORF-T	-0.732165021	-1.037447452	1.166111796	0.996714744	0.003481111
870699	875163	16	+	MMS1	ORF-T	ORF-T	0.008557425	-0.308716363	-0.423512169	-0.366092909	0.144719068
875163	876291	16	+	RHO1	ORF-T	ORF-T	-0.255702726	-0.709274112	-0.341265032	-0.188202764	-0.24567207
876387	876819	16	+	unknown	no_ovlp	CUTs	1.29313845	0.190450357	0.04059326	-0.215492693	0.074872313
876819	877275	16	+	CUT434	CUTs	CUTs	2.677442238	0.240108443	-0.187803772	0.125069431	0.247404991
878075	878675	16	+	unknown	no_ovlp	SUTs	0.315988332	-0.293328284	-0.080107112	-0.520009064	-0.438718341
878675	880243	16	+	JIP5	ORF-T	ORF-T	0.508174432	-0.620711606	-0.64720247	-0.104809194	0.183645764
883499	883787	16	+	unknown	no_ovlp	SUTs	0.033678761	-0.493344712	0.032354619	-0.066843592	0.192452447
883787	885587	16	+	BSP1	ORF-T	ORF-T	-0.319501463	-0.666973999	-0.372287105	0.08221888	-0.532684625
885771	886419	16	+	YPR172W	ORF-T	ORF-T	-0.212673402	-0.150896808	0.499311431	-0.055946969	0.503580034
888971	891107	16	+	DPB2	ORF-T	ORF-T	0.411089505	0.159940167	-0.334546132	-0.355957206	0.623908973
892315	893811	16	+	PRP4	ORF-T	ORF-T	-0.135899802	-0.553535368	0.214234311	0.11310173	-0.096984879
895947	897067	16	+	AOS1	ORF-T	ORF-T	0.27035376	-0.012481967	0.264650172	-0.134434317	-0.579133159
900131	900523	16	+	SMX3	ORF-T	ORF-T	0.585451993	0.526678937	0.11070702	0.024874572	0.266438661
900723	901643	16	+	DPM1	ORF-T	ORF-T	-0.42069874	0.039193959	-0.085529629	-0.112436542	0.046887882
901643	902811	16	+	GDB1	ORF-T	ORF-T	-0.995877906	-0.498940236	1.828142423	1.304984123	0.667232034
906915	909731	16	+	ATG13	ORF-T	ORF-T	-0.463284522	-0.551054518	0.033792995	0.009900968	0.552865845
911363	911931	16	+	RPO26	ORF-T	ORF-T	-0.2296276	-0.664979998	-0.622235321	-0.219852464	0.203269941
912099	912635	16	+	unknown	no_ovlp	SUTs	-1.238648863	-0.797657516	-0.186892428	0.220283402	0.10982364
912635	917083	16	+	SKI3	ORF-T	ORF-T	0.01734942	-0.395283656	-0.453678001	0.085893521	-0.155491104
919315	920699	16	+	QCR2	ORF-T	ORF-T	-0.186011883	0.415056449	0.742010462	0.329265034	0.32665059
922211	922563	16	+	AQY1	ORF-T	ORF-T	-1.678477251	-0.020666328	1.739941107	1.131137536	2.006767377
927851	928707	16	+	unknown	no_ovlp	SUTs	-0.012353833	-0.646294246	-0.445128412	-0.648484416	-0.86343189
928707	931179	16	+	SUT430	SUTs	SUTs	0.416254266	-0.333655222	-0.806548993	-0.521131378	-1.511039605
931179	933163	16	+	YPR196W, YF	other	other	1.621639267	0.151079114	-0.923028719	-0.540833903	-1.516267301
933987	935699	16	+	SGE1	ORF-T	ORF-T	0.115817085	-0.328583643	-0.283787465	0.030776551	-0.707773778
936355	937771	16	+	unknown	no_ovlp	CUTs	2.181085441	0.401955194	-0.195664279	-0.414435284	-2.232766782

Table S4: List of enriched transcription factor binding sites

cluster	regulator	target_gene
Class I	MSN2	BDH2
Class I	AFT2	HSP26
Class I	ADR1	HSP26
Class I	MSN2	HSP26
Class I	NRG1	HSP26
Class I	STP1	NDE2
Class I	MSN2	NDE2
Class I	ADR1	NDE2
Class I	MSN2	YER079W
Class I	NRG1	AMS1
Class I	ADR1	AMS1
Class I	AFT2	FMP48
Class I	STP1	FMP48
Class I	NRG1	FMP48
Class I	ADR1	FMP48
Class I	MSN2	FMP48
Class I	NRG1	CTT1
Class I	MSN2	CTT1
Class I	ADR1	CTT1
Class I	AFT2	CTT1
Class I	ADR1	RIM4
Class I	MSN2	RIM4
Class I	STP1	RIM4
Class I	AFT2	RIM4
Class I	STP1	SDP1
Class I	AFT2	SDP1
Class I	MSN2	SDP1
Class I	ADR1	SDP1
Class I	AFT2	TIR3
Class I	AFT2	SIP4
Class I	STP1	SIP4
Class I	STP1	BOP2
Class I	AFT2	BOP2
Class I	NRG1	BOP2
Class I	ADR1	BOP2
Class I	STP1	HXT2
Class I	NRG1	HXT2
Class I	ADR1	HXT2
Class I	AFT2	HXT2
Class I	STP1	SPG4
Class I	AFT2	SPG4
Class I	ADR1	SPG4
Class I	MSN2	MLS1

Class I	MSN2	PHM7
Class I	NRG1	PHM7
Class I	ADR1	PHM7
Class I	ADR1	YPL230W
Class I	MSN2	YPL230W
Class I	NRG1	YPL230W
Class I	STP1	YPL230W
Class I	AFT2	YPL230W
Class I	STP1	CSR2
Class I	ADR1	CSR2
Class I	NRG1	CSR2
Class I	NRG1	PHO5
Class I	ADR1	YBR116C, TKL2
Class I	MSN2	YBR116C, TKL2
Class I	AFT2	YBR116C, TKL2
Class I	ADR1	SSE2
Class I	MSN2	SSE2
Class I	ADR1	SPL2
Class I	STP1	SPL2
Class I	NRG1	SPL2
Class I	MSN2	MNN4
Class I	ADR1	MNN4
Class I	AFT2	MNN4
Class I	STP1	MNN4
Class I	ADR1	MSC1
Class I	MSN2	MSC1
Class I	AFT2	PHO84
Class I	NRG1	PHO84
Class I	ADR1	CYB2
Class I	MSN2	CYB2
Class I	ADR1	ISF1
Class I	STP1	ISF1
Class I	MSN2	ISF1
Class I	STP1	YNL195C
Class I	MSN2	YNL194C
Class I	NRG1	YNL194C
Class I	AFT2	YNL194C
Class I	ADR1	YNL194C
Class I	STP1	YNL194C
Class I	ADR1	GAC1
Class I	MSN2	GAC1
Class I	STP1	GAC1
Class I	MSN2	GSP2
Class I	ADR1	GSP2
Class I	AFT2	GSP2
Class I	STP1	GSP2

Class I	MSN2	GYP5
Class III	HAP5	BDH1
Class III	HAP3	BDH1
Class III	ADR1	BDH1
Class III	MSN2	BDH1
Class III	MSN4	BDH1
Class III	MOT3	BDH1
Class III	MSN2	ECM1
Class III	MSN4	ECM1
Class III	ADR1	SPO7
Class III	RTG3	ECM13
Class III	RTG3	HEK2
Class III	SKN7	HEK2
Class III	ADR1	HEK2
Class III	MSN2	HEK2
Class III	MSN4	HEK2
Class III	ADR1	RCR1
Class III	MSN2	RCR1
Class III	MSN4	RCR1
Class III	MOT3	RCR1
Class III	ADR1	YBR030W
Class III	MSN4	YBR030W
Class III	HAP5	YBR030W
Class III	HAP3	YBR030W
Class III	MOT3	YBR030W
Class III	MBP1	RDH54
Class III	MSN4	RFC5
Class III	MBP1	RFC5
Class III	MOT3	SIF2
Class III	HAP5	CDC28
Class III	HAP3	CDC28
Class III	SKN7	CDC28
Class III	RTG3	KAR4
Class III	MSN2	BPH1
Class III	MSN4	BPH1
Class III	HAP5	BPH1
Class III	HAP3	BPH1
Class III	MSN4	YCR051W
Class III	MOT3	YCR051W
Class III	RTG3	YCR051W
Class III	MOT3	SOL2
Class III	SKN7	SOL2
Class III	MSN2	SOL2
Class III	MSN4	SOL2
Class III	ADR1	SOL2
Class III	HAP5	SRB8

Class III	HAP3	SRB8
Class III	MBP1	DTD1
Class III	MOT3	MRPL11
Class III	ADR1	MRPL11
Class III	ADR1	LYS20
Class III	SKN7	LYS20
Class III	MOT3	LYS20
Class III	HAP5	LYS20
Class III	HAP3	LYS20
Class III	MSN2	PCL9
Class III	MSN4	PCL9
Class III	ADR1	PCL9
Class III	RTG3	PCL9
Class III	SKN7	PCL2
Class III	RTG3	PCL2
Class III	MBP1	PCL2
Class III	MOT3	PCL2
Class III	MSN2	RAD55
Class III	MSN4	RAD55
Class III	MBP1	RAD55
Class III	HAP5	YDR119W
Class III	HAP3	YDR119W
Class III	MSN4	YDR119W
Class III	SKN7	YDR119W
Class III	ADR1	YDR119W
Class III	RTG3	YDR119W
Class III	MOT3	YDR119W
Class III	ADR1	RPA14, YDR157W
Class III	MSN4	RPA14, YDR157W
Class III	SKN7	RPA14, YDR157W
Class III	MSN2	RPA14, YDR157W
Class III	MBP1	UPC2
Class III	MOT3	UPC2
Class III	ADR1	UPC2
Class III	RTG3	UPC2
Class III	MSN2	HTA1
Class III	MSN4	HTA1
Class III	MBP1	HTA1
Class III	HAP5	HTA1
Class III	HAP3	HTA1
Class III	ADR1	HTA1
Class III	MBP1	RNH202
Class III	MOT3	RNH202
Class III	HAP5	BFR2
Class III	MOT3	YDR336W
Class III	MSN2	SPT3

Class III	MSN4	SPT3
Class III	ADR1	SPT3
Class III	MOT3	SPT3
Class III	ADR1	UTP5
Class III	SKN7	UTP5
Class III	MSN2	UTP5
Class III	MSN4	UTP5
Class III	HAP5	UTP5
Class III	HAP3	UTP5
Class III	ADR1	HPT1
Class III	RTG3	HPT1
Class III	MBP1	HPT1
Class III	MSN2	HPT1
Class III	MSN4	HPT1
Class III	HAP5	HPT1
Class III	HAP3	HPT1
Class III	ADR1	SYF1
Class III	SKN7	GPI19, THI74, LRS4
Class III	MSN4	DIG2
Class III	MSN2	DIG2
Class III	ADR1	DIG2
Class III	MBP1	DIG2
Class III	ADR1	IZH1
Class III	MOT3	SLF1
Class III	RTG3	IES6
Class III	HAP5	IES6
Class III	HAP3	IES6
Class III	MOT3	IES6
Class III	ADR1	GEA2
Class III	HAP5	GEA2
Class III	HAP3	GEA2
Class III	MSN2	GEA2
Class III	MSN4	GEA2
Class III	SKN7	PRE1
Class III	MSN2	PRE1
Class III	MSN4	PRE1
Class III	MBP1	BIM1
Class III	ADR1	PET117
Class III	RTG3	PET117
Class III	MSN2	PET117
Class III	MSN4	PET117
Class III	MSN2	PCL6
Class III	MSN4	PCL6
Class III	MOT3	PCL6
Class III	SKN7	RPS8B
Class III	MOT3	LCP5

Class III	MOT3	YER128W
Class III	RTG3	KIP3
Class III	HAP5	KIP3
Class III	HAP3	KIP3
Class III	MOT3	KIP3
Class III	MBP1	YPT32
Class III	MOT3	NUP49
Class III	HAP5	NUP49
Class III	HAP3	NUP49
Class III	ADR1	NUP49
Class III	MOT3	CDC20
Class III	HAP5	CDC20
Class III	HAP3	CDC20
Class III	MOT3	SRM1
Class III	SKN7	SPC105
Class III	SKN7	YGR017W
Class III	MOT3	YGR017W
Class III	ADR1	YGR017W
Class III	MSN2	PEF1
Class III	MSN4	PEF1
Class III	MOT3	PEF1
Class III	HAP5	PEF1
Class III	HAP3	PEF1
Class III	SKN7	PEF1
Class III	HAP3	UPF3
Class III	HAP5	UPF3
Class III	MSN2	UPF3
Class III	MSN4	UPF3
Class III	MBP1	DAM1
Class III	SKN7	DAM1
Class III	ADR1	YGR125W
Class III	MSN2	YGR125W
Class III	MSN4	YGR125W
Class III	MBP1	YGR125W
Class III	RTG3	YGR125W
Class III	ADR1	YGR131W
Class III	MSN2	YGR131W
Class III	MSN4	YGR131W
Class III	SKN7	YGR131W
Class III	MOT3	YGR131W
Class III	HAP5	YGR131W
Class III	HAP3	YGR131W
Class III	MOT3	PTI1
Class III	HAP5	RBG2
Class III	HAP3	RBG2
Class III	MOT3	YGR251W

Class III	HAP5	YGR251W
Class III	HAP3	YGR251W
Class III	MBP1	YGR251W
Class III	SKN7	WSC4
Class III	MSN2	WSC4
Class III	MSN4	WSC4
Class III	MOT3	WSC4
Class III	ADR1	WSC4
Class III	HAP5	RPL27A
Class III	HAP3	RPL27A
Class III	MSN2	RPL27A
Class III	MSN4	RPL27A
Class III	ADR1	RPL27A
Class III	ADR1	DIA4
Class III	HAP5	DIA4
Class III	MBP1	DIA4
Class III	MSN2	DIA4
Class III	MSN4	DIA4
Class III	MBP1	SMF2
Class III	MOT3	HTD2
Class III	HAP5	NOP10
Class III	HAP3	NOP10
Class III	SKN7	NOP10
Class III	RTG3	ERP5
Class III	MBP1	ERP5
Class III	MBP1	DSE2
Class III	ADR1	MTG2
Class III	SKN7	MTG2
Class III	MOT3	YHR182W
Class III	SKN7	YHR182W
Class III	SKN7	SNL1
Class III	MSN2	SNL1
Class III	MSN4	SNL1
Class III	RTG3	SNL1
Class III	ADR1	SNL1
Class III	ADR1	CFD1
Class III	RTG3	CFD1
Class III	HAP5	MRS1
Class III	HAP3	MRS1
Class III	RTG3	MRS1
Class III	MOT3	PRP21
Class III	MSN4	GON7
Class III	MSN2	YJL150W, YJL149W
Class III	SKN7	MRS3
Class III	MSN2	MRS3
Class III	MSN4	MRS3

Class III	RTG3	MRS3
Class III	ADR1	MRS3
Class III	MSN2	YJL062W-A
Class III	MSN4	YJL062W-A
Class III	ADR1	YJL062W-A
Class III	MOT3	IRC8
Class III	RTG3	IRC8
Class III	HAP5	IRC8
Class III	HAP3	IRC8
Class III	MSN4	IRC8
Class III	MSN2	RNR2
Class III	MSN4	RNR2
Class III	HAP5	RNR2
Class III	HAP3	RNR2
Class III	MBP1	AVT1
Class III	MOT3	AVT1
Class III	MSN2	AVT1
Class III	MSN4	AVT1
Class III	MSN2	POL31
Class III	MSN4	POL31
Class III	MBP1	POL31
Class III	MOT3	ISY1
Class III	RTG3	ISY1
Class III	MSN4	RPA12
Class III	MSN2	MOG1
Class III	MSN4	MOG1
Class III	RTG3	DPH2
Class III	MSN4	DPH2
Class III	MSN2	DPH2
Class III	MOT3	DPH2
Class III	SKN7	ASH1
Class III	ADR1	ASH1
Class III	MBP1	ASH1
Class III	MOT3	ASH1
Class III	MSN4	ASH1
Class III	HAP5	ASH1
Class III	HAP3	ASH1
Class III	ADR1	LOT5
Class III	MBP1	LTV1
Class III	RTG3	RRN3
Class III	MSN4	RRN3
Class III	MOT3	RRN3
Class III	MBP1	MIF2
Class III	ADR1	TUL1
Class III	HAP5	MSA2
Class III	HAP3	MSA2

Class III	RTG3	MSA2
Class III	MBP1	MSA2
Class III	MBP1	RAD5
Class III	RTG3	RAD5
Class III	MSN4	RPL10
Class III	HAP5	YLR099W-A
Class III	HAP3	YLR099W-A
Class III	ADR1	YLR099W-A
Class III	SKN7	YLR099W-A
Class III	MOT3	YLR099W-A
Class III	HAP5	IDP2
Class III	ADR1	IDP2
Class III	HAP3	IDP2
Class III	MOT3	IDP2
Class III	ADR1	YLR177W
Class III	SKN7	YLR177W
Class III	MSN2	MSC3
Class III	MSN4	MSC3
Class III	ADR1	MSC3
Class III	RTG3	CDC46
Class III	ADR1	CDC46
Class III	MSN2	CDC46
Class III	MSN4	CDC46
Class III	MBP1	CDC46
Class III	ADR1	YLR352W
Class III	MSN2	ROM2
Class III	MSN4	ROM2
Class III	ADR1	ROM2
Class III	RTG3	ROM2
Class III	HAP5	ROM2
Class III	HAP3	ROM2
Class III	MSN4	FPR4
Class III	MOT3	ZDS2
Class III	HAP5	ZDS2
Class III	HAP3	ZDS2
Class III	RTG3	ZDS2
Class III	MBP1	ZDS2
Class III	ADR1	ZDS2
Class III	MOT3	SPC2
Class III	MSN2	SUR7
Class III	MSN4	SUR7
Class III	MOT3	SUR7
Class III	SKN7	SUR7
Class III	ADR1	SUR7
Class III	ADR1	RIM9, AEP1
Class III	MBP1	RIM9, AEP1

Class III	SKN7	ADD37
Class III	MSN2	ADD37
Class III	MSN4	ADD37
Class III	ADR1	ADD37
Class III	ADR1	CIK1
Class III	MOT3	ERG2
Class III	MOT3	YHM2
Class III	MBP1	YHM2
Class III	HAP5	YHM2
Class III	HAP3	YHM2
Class III	MSN4	YHM2
Class III	MBP1	GOT1
Class III	MOT3	GOT1
Class III	MBP1	VNX1
Class III	MSN2	VNX1
Class III	MSN4	VNX1
Class III	ADR1	VNX1
Class III	SKN7	POP3
Class III	RTG3	POP3
Class III	MBP1	POP3
Class III	MBP1	POL2
Class III	ADR1	YNL247W
Class III	RTG3	YNL247W
Class III	MOT3	LAP3
Class III	RTG3	LAP3
Class III	ADR1	LAP3
Class III	MBP1	YNL181W
Class III	MOT3	YNL155W
Class III	MSN2	YNL155W
Class III	MSN4	YNL155W
Class III	ADR1	YNL155W
Class III	SKN7	SPC98
Class III	HAP5	SPC98
Class III	HAP3	SPC98
Class III	MBP1	SPC98
Class III	MBP1	POL1
Class III	HAP5	POL1
Class III	HAP3	POL1
Class III	ADR1	NIS1
Class III	MSN4	NIS1
Class III	MBP1	NIS1
Class III	MSN2	VAC7
Class III	MSN4	VAC7
Class III	MOT3	MSG5
Class III	RTG3	NRM1
Class III	MBP1	NRM1

Class III	HAP5	NRM1
Class III	HAP3	NRM1
Class III	MOT3	YNR018W
Class III	HAP5	YNR018W
Class III	HAP3	YNR018W
Class III	SKN7	YNR018W
Class III	MBP1	YNR018W
Class III	MSN4	YNR018W
Class III	MSN2	YNR018W
Class III	HAP5	PET494
Class III	HAP3	PET494
Class III	MSN4	SKM1
Class III	MSN2	SKM1
Class III	ADR1	SKM1
Class III	HAP5	YOL097W-A
Class III	HAP3	YOL097W-A
Class III	RTG3	YOL097W-A
Class III	ADR1	YOL097W-A
Class III	MBP1	AVO1
Class III	RTG3	AVO1
Class III	ADR1	AVO1
Class III	ADR1	BUB3
Class III	MSN2	BUB3
Class III	MSN4	BUB3
Class III	MSN2	IRC23
Class III	MSN4	IRC23
Class III	RTG3	IRC23
Class III	ADR1	IRC23
Class III	HAP5	VAM3
Class III	HAP3	VAM3
Class III	SKN7	RGS2
Class III	MOT3	RGS2
Class III	ADR1	RGS2
Class III	RTG3	RGS2
Class III	MOT3	YOR111W
Class III	MBP1	VPS17
Class III	MSN2	VPS17
Class III	MSN4	VPS17
Class III	MSN4	DFR1
Class III	HAP5	DFR1
Class III	HAP3	DFR1
Class III	MSN2	YOR238W
Class III	HAP5	CAF20
Class III	HAP3	CAF20
Class III	MOT3	HUA2
Class III	MSN2	HUA2

Class III	MSN4	HUA2
Class III	ADR1	HUA2
Class III	MBP1	HUA2
Class III	HAP5	KRE5
Class III	HAP3	KRE5
Class III	RTG3	KRE5
Class III	SKN7	HAP5
Class III	HAP5	SCP1
Class III	HAP3	SCP1
Class III	ADR1	SCP1
Class III	MOT3	ACM1
Class III	MBP1	ACM1
Class III	RTG3	ACM1
Class III	ADR1	SSO1
Class III	MBP1	SSO1
Class III	MSN4	SSO1
Class III	MSN2	SSO1
Class III	SKN7	YPL229W
Class III	ADR1	YPL229W
Class III	RTG3	YPL229W
Class III	MBP1	YPL216W
Class III	MBP1	RKM1
Class III	RTG3	CTI6
Class III	MOT3	CTI6
Class III	HAP5	CTI6
Class III	HAP3	CTI6
Class III	MSN2	CTI6
Class III	MSN4	CTI6
Class III	ADR1	CTI6
Class III	HAP5	POC4
Class III	HAP3	POC4
Class III	MBP1	POC4
Class III	SKN7	POC4
Class III	MSN4	SSU1
Class III	MSN2	SSU1
Class III	ADR1	SSU1
Class III	RTG3	SSU1
Class III	MOT3	SSU1
Class III	HAP5	SSU1
Class III	HAP3	SSU1
Class III	ADR1	ALD6
Class III	MSN2	ALD6
Class III	MSN4	ALD6
Class III	MOT3	OAZ1
Class III	RTG3	OAZ1
Class III	ADR1	YPL039W, YPL038W-A

Class III	ADR1	MCM16
Class III	RTG3	TAH18
Class III	MSN2	UBA3
Class III	MSN4	UBA3
Class III	MBP1	MRL1
Class III	MSN2	MRL1
Class III	MSN4	MRL1
Class III	ADR1	PIS1
Class III	MOT3	CTF4
Class III	MBP1	CTF4
Class III	MSN4	CTF4
Class III	SKN7	YPR153W
Class III	HAP5	MMS1
Class III	HAP3	MMS1
Class III	RTG3	NUT2
Class III	MSN4	SMX3
Class III	MSN2	SMX3
Class III	ADR1	SMX3
Class III	MOT3	PTA1
Class III	MSN2	LTE1
Class III	MSN4	LTE1
Class III	ADR1	ATS1
Class III	MSN4	SYN8
Class III	MBP1	BUD14
Class III	ADR1	BUD14
Class III	RTG3	BUD14
Class III	RTG3	YBL086C
Class III	MSN2	YBL086C
Class III	MSN4	YBL086C
Class III	ADR1	YBL086C
Class III	MBP1	YBL086C
Class III	ADR1	FUI1
Class III	MBP1	POL12
Class III	RTG3	POL12
Class III	MOT3	STU1
Class III	HAP5	STU1
Class III	HAP3	STU1
Class III	MBP1	STU1
Class III	ADR1	STU1
Class III	ADR1	YBL029C-A
Class III	MSN2	YBL029C-A
Class III	MSN4	YBL029C-A
Class III	RTG3	YBL029C-A
Class III	MBP1	YBL029C-A
Class III	SKN7	YBL029C-A
Class III	MOT3	YBL029C-A

Class III	HAP5	HAP3
Class III	HAP3	HAP3
Class III	MSN2	HAP3
Class III	MSN4	HAP3
Class III	RTG3	PDX3
Class III	MSN2	PDX3
Class III	MSN4	PDX3
Class III	HAP5	SCO1
Class III	HAP3	SCO1
Class III	MSN4	SCO1
Class III	ADR1	TCM62
Class III	MOT3	TCM62
Class III	MSN2	TCM62
Class III	MSN4	TCM62
Class III	MOT3	YBR062C
Class III	MOT3	NRG2
Class III	MSN2	NRG2
Class III	MSN4	NRG2
Class III	MBP1	NRG2
Class III	SKN7	NRG2
Class III	ADR1	NRG2
Class III	MSN2	ALG14
Class III	MSN4	ALG14
Class III	MBP1	ALG14
Class III	ADR1	ALG14
Class III	MOT3	PHO3
Class III	MSN2	MRPL36
Class III	MSN4	MRPL36
Class III	MBP1	NPL4
Class III	SKN7	STP22
Class III	SKN7	BUD23
Class III	MSN2	BUD23
Class III	MSN4	BUD23
Class III	MOT3	RSA4
Class III	SKN7	ASF2
Class III	MBP1	ASF2
Class III	MOT3	ASF2
Class III	MSN2	ASF2
Class III	MSN4	ASF2
Class III	MOT3	FAP7
Class III	MOT3	LUC7
Class III	MSN4	LUC7
Class III	MSN2	THI3
Class III	MSN4	THI3
Class III	ADR1	THI3
Class III	MOT3	THI3

Class III	HAP5	COX9
Class III	HAP3	COX9
Class III	RTG3	COX9
Class III	ADR1	SLC1
Class III	MSN4	SLC1
Class III	HAP5	SLC1
Class III	MOT3	PUS9
Class III	ADR1	RAD28
Class III	HAP3	RAD28
Class III	RTG3	NRG1
Class III	ADR1	NRG1
Class III	MSN2	NRG1
Class III	MSN4	NRG1
Class III	MBP1	NRG1
Class III	SKN7	NRG1
Class III	HAP5	NRG1
Class III	HAP3	NRG1
Class III	MSN2	TPI1
Class III	MSN4	TPI1
Class III	SKN7	TPI1
Class III	MOT3	TPI1
Class III	MSN2	SSS1
Class III	MSN4	SSS1
Class III	MBP1	SSS1
Class III	HAP5	SSS1
Class III	HAP3	SSS1
Class III	HAP5	HNT2
Class III	HAP3	HNT2
Class III	MOT3	PEP7
Class III	HAP5	PEP7
Class III	HAP3	PEP7
Class III	ADR1	SVF1
Class III	ADR1	NCB2
Class III	SKN7	NCB2
Class III	MSN2	NCB2
Class III	MSN4	NCB2
Class III	HAP5	NCB2
Class III	HAP3	NCB2
Class III	ADR1	YDR415C
Class III	MSN2	SSN2
Class III	MSN4	SSN2
Class III	ADR1	SSN2
Class III	SKN7	PFA5
Class III	MSN2	JIP4
Class III	MSN4	JIP4
Class III	RTG3	JIP4

Class III	ADR1	YDR476C
Class III	MOT3	SEC20
Class III	HAP5	SEC20
Class III	HAP3	SEC20
Class III	RTG3	GIN4
Class III	MBP1	GIN4
Class III	MSN2	GIN4
Class III	MSN4	GIN4
Class III	ADR1	GIN4
Class III	SKN7	APA2
Class III	HAP5	APA2
Class III	HAP3	APA2
Class III	ADR1	YEL047C
Class III	MBP1	YEL047C
Class III	MSN2	YEL047C
Class III	ADR1	FCY2
Class III	HAP5	YER078C
Class III	HAP3	YER078C
Class III	MBP1	YER078C
Class III	MSN2	YER078C
Class III	MSN4	YER078C
Class III	MSN2	GET2
Class III	MSN4	GET2
Class III	HAP5	PUP3
Class III	HAP3	PUP3
Class III	MSN2	PUP3
Class III	MSN4	PUP3
Class III	MBP1	PUP3
Class III	RTG3	PUP3
Class III	RTG3	PMD1
Class III	MOT3	PMD1
Class III	SKN7	FTR1
Class III	ADR1	FTR1
Class III	HAP5	FTR1
Class III	HAP3	FTR1
Class III	HAP5	YER156C
Class III	HAP3	YER156C
Class III	HAP5	RPL2A
Class III	HAP3	RPL2A
Class III	ADR1	RPL2A
Class III	MSN2	RPL2A
Class III	MSN4	RPL2A
Class III	MOT3	RPL2A
Class III	ADR1	RPL29, YFR032C-B
Class III	MSN2	RPL29, YFR032C-B
Class III	MSN4	RPL29, YFR032C-B

Class III	HAP3	RPL29, YFR032C-B
Class III	HAP5	RPL29, YFR032C-B
Class III	HAP5	NIF3
Class III	HAP3	NIF3
Class III	MBP1	EMP24
Class III	MSN2	EMP24
Class III	MSN4	EMP24
Class III	MBP1	SAE2
Class III	HAP5	RAD54
Class III	HAP3	RAD54
Class III	MBP1	RAD54
Class III	MSN4	RAD54
Class III	MOT3	GPI10
Class III	RTG3	GPI10
Class III	MOT3	MPS2
Class III	ADR1	MPS2
Class III	MBP1	MPS2
Class III	RTG3	MPS2
Class III	MOT3	PAC10
Class III	HAP5	PAC10
Class III	HAP3	PAC10
Class III	MBP1	PAC10
Class III	ADR1	PAC10
Class III	MBP1	YGR151C, RSR1
Class III	RTG3	YGR151C, RSR1
Class III	MSN2	PBP1
Class III	MSN4	PBP1
Class III	ADR1	PBP1
Class III	HAP5	PBP1
Class III	HAP3	PBP1
Class III	MSN2	RNR4
Class III	HAP5	RNR4
Class III	MBP1	RNR4
Class III	MSN4	PDX1
Class III	SKN7	YGR210C
Class III	ADR1	YAP1802
Class III	MSN2	YAP1802
Class III	MSN4	YAP1802
Class III	HAP5	YAP1802
Class III	HAP3	YAP1802
Class III	MSN4	APM2
Class III	MSN4	MSR1
Class III	MBP1	BIG1
Class III	MOT3	BIG1
Class III	MSN4	SPS100
Class III	RTG3	SPS100

Class III	MOT3	SPS100
Class III	ADR1	SPS100
Class III	MBP1	CSM2
Class III	MSN4	SDS3
Class III	MOT3	SDS3
Class III	RTG3	SER33
Class III	SKN7	SER33
Class III	MOT3	SER33
Class III	MOT3	MMF1
Class III	RTG3	MMF1
Class III	MSN4	MMF1
Class III	MSN2	MMF1
Class III	ADR1	MPH1
Class III	ADR1	TRK1
Class III	MOT3	TRK1
Class III	SKN7	TRK1
Class III	MSN2	TRK1
Class III	MSN4	TRK1
Class III	MBP1	LSM1
Class III	MOT3	RPC17
Class III	MBP1	RPC17
Class III	MSN2	RPC17
Class III	HAP5	GEA1
Class III	HAP3	GEA1
Class III	MSN2	OPI3
Class III	MSN4	OPI3
Class III	ADR1	YJR129C
Class III	RTG3	MCM22
Class III	MSN2	MCM22
Class III	MSN4	MCM22
Class III	MOT3	YKL115C, APN1
Class III	MBP1	RAD27
Class III	HAP5	RAD27
Class III	HAP3	RAD27
Class III	ADR1	OAR1
Class III	MOT3	OAR1
Class III	SKN7	CCP1
Class III	MSN2	CCP1
Class III	MSN4	CCP1
Class III	HAP5	CCP1
Class III	HAP3	CCP1
Class III	ADR1	GPI13
Class III	HAP5	HIF1
Class III	HAP3	HIF1
Class III	MBP1	HIF1
Class III	RTG3	HIF1

Class III	MSN2	PPR1
Class III	MSN4	PPR1
Class III	ADR1	PPR1
Class III	RTG3	IZH3
Class III	MOT3	IZH3
Class III	ADR1	IZH3
Class III	SKN7	IZH3
Class III	MSN4	IZH3
Class III	HAP5	IZH3
Class III	HAP3	IZH3
Class III	ADR1	RIC1
Class III	MBP1	YLR049C
Class III	MSN4	YLR049C
Class III	MSN2	YLR049C
Class III	ADR1	YLR049C
Class III	RTG3	YLR049C
Class III	SKN7	FCF2
Class III	MSN2	FCF2
Class III	MSN4	FCF2
Class III	ADR1	FCF2
Class III	MSN4	BUD20
Class III	HAP5	GIS3
Class III	HAP3	GIS3
Class III	HAP5	ICT1
Class III	HAP3	ICT1
Class III	ADR1	ICT1
Class III	SKN7	ICT1
Class III	MBP1	RNH203
Class III	ADR1	YLR194C
Class III	ADR1	YLR222C-A, IFH1
Class III	HAP5	YLR222C-A, IFH1
Class III	HAP3	YLR222C-A, IFH1
Class III	MOT3	YLR222C-A, IFH1
Class III	SKN7	YLR222C-A, IFH1
Class III	MSN2	YLR222C-A, IFH1
Class III	MSN4	YLR222C-A, IFH1
Class III	MBP1	EST1
Class III	MOT3	STT4
Class III	ADR1	CDC3
Class III	SKN7	CDC3
Class III	MSN2	CWC24
Class III	MSN4	CWC24
Class III	ADR1	ILV5
Class III	MSN2	ILV5
Class III	MSN4	ILV5
Class III	RTG3	ILV5

Class III	MOT3	IKI3, SWC7
Class III	MBP1	IKI3, SWC7
Class III	MSN2	IKI3, SWC7
Class III	MSN4	IKI3, SWC7
Class III	MBP1	AFG2
Class III	HAP5	YLR437C
Class III	HAP3	YLR437C
Class III	MOT3	YLR437C
Class III	ADR1	YLR437C
Class III	MSN2	YLR437C
Class III	MSN4	YLR437C
Class III	MBP1	YLR437C
Class III	RTG3	YLR437C
Class III	ADR1	TAF8
Class III	HAP5	TAF8
Class III	HAP3	TAF8
Class III	MOT3	MDM1
Class III	ADR1	COG8
Class III	MBP1	COG8
Class III	MBP1	PIF1
Class III	MOT3	PIF1
Class III	HAP5	SUB1
Class III	HAP3	SUB1
Class III	MOT3	SUB1
Class III	HAP5	MED11
Class III	HAP3	MED11
Class III	ADR1	RPL15B
Class III	SKN7	TIF34
Class III	ADR1	TIF34
Class III	MSN4	TPP1
Class III	RTG3	INP2
Class III	ADR1	INP2
Class III	MBP1	HFA1
Class III	MOT3	HFA1
Class III	MOT3	FSH2
Class III	MSN2	FSH2
Class III	MSN4	FSH2
Class III	MBP1	YMR244C-A
Class III	SKN7	YMR244C-A
Class III	ADR1	YMR244C-A
Class III	MSN2	YMR244C-A
Class III	MSN4	YMR244C-A
Class III	MSN2	YMR259C
Class III	MSN4	YMR259C
Class III	RTG3	YMR259C
Class III	MSN4	ATM1

Class III	ADR1	ADH2
Class III	MOT3	ADH2
Class III	MBP1	YIF1
Class III	HAP5	YNL254C
Class III	HAP3	YNL254C
Class III	ADR1	YNL254C
Class III	MSN2	YNL254C
Class III	MSN4	YNL254C
Class III	MOT3	YNL254C
Class III	MOT3	NAR1
Class III	RTG3	NAR1
Class III	ADR1	NAR1
Class III	MOT3	JJJ1
Class III	MSN4	IPI3
Class III	MBP1	IPI3
Class III	MOT3	NOP15
Class III	ADR1	NOP15
Class III	MSN4	EOS1
Class III	MOT3	EOS1
Class III	HAP5	EOS1
Class III	HAP3	EOS1
Class III	MOT3	YNL024C-A
Class III	MBP1	YNL024C
Class III	HAP5	RSM19
Class III	HAP3	RSM19
Class III	ADR1	COQ2
Class III	MSN4	COQ2
Class III	MOT3	ESF2
Class III	MOT3	MED7
Class III	RTG3	YGK3
Class III	ADR1	YGK3
Class III	MSN2	YGK3
Class III	MSN4	YGK3
Class III	ADR1	TRM11
Class III	RTG3	WRS1
Class III	MOT3	WRS1
Class III	MSN2	COQ3
Class III	MSN4	COQ3
Class III	MOT3	COQ3
Class III	RTG3	HMI1
Class III	MSN4	HMI1
Class III	ADR1	HMI1
Class III	SKN7	RIB2
Class III	ADR1	RPB11
Class III	MSN2	RPB11
Class III	MSN4	RPB11

Class III	MOT3	EXO1
Class III	MBP1	EXO1
Class III	MOT3	RAT1
Class III	MSN2	YOR052C
Class III	MSN4	YOR052C
Class III	SKN7	YOR052C
Class III	ADR1	YOR052C
Class III	RTG3	YOR052C
Class III	MOT3	YOR052C
Class III	HAP5	YNG1
Class III	HAP3	YNG1
Class III	MSN2	YNG1
Class III	MSN4	YNG1
Class III	ADR1	YNG1
Class III	MOT3	RPO31
Class III	ADR1	RPO31
Class III	RTG3	LEO1
Class III	MOT3	RUP1
Class III	SKN7	RUP1
Class III	MSN2	RUP1
Class III	MSN4	RUP1
Class III	MBP1	ELG1
Class III	HAP5	PNO1
Class III	HAP3	PNO1
Class III	ADR1	PNO1
Class III	ADR1	YOR152C
Class III	HAP5	YOR152C
Class III	HAP3	YOR152C
Class III	MSN4	YOR152C
Class III	SKN7	YOR152C
Class III	SKN7	ISU2
Class III	RTG3	ISU2
Class III	MOT3	ISU2
Class III	MSN2	ISU2
Class III	MSN4	ISU2
Class III	ADR1	FSH3
Class III	HAP5	FSH3
Class III	HAP3	FSH3
Class III	MOT3	SNU66
Class III	MSN2	TYE7
Class III	MOT3	GPB1
Class III	ADR1	GPB1
Class III	MSN2	RBD2
Class III	MSN4	RBD2
Class III	ADR1	YPL236C
Class III	MBP1	IPL1

Class III	ADR1	YIG1
Class III	RTG3	YIG1
Class III	SKN7	YIG1
Class III	HAP5	YIG1
Class III	HAP3	YIG1
Class III	MBP1	YIG1
Class III	SKN7	YPL158C
Class III	MOT3	YPL158C
Class III	ADR1	YPL158C
Class III	ADR1	HHO1
Class III	MOT3	HHO1
Class III	MBP1	HHO1
Class III	MSN2	TFB2
Class III	MSN4	TFB2
Class III	MOT3	TFB2
Class III	MOT3	YPL077C
Class III	MOT3	YPL056C
Class III	MBP1	YPL056C
Class III	SKN7	LGE1
Class III	MSN2	LGE1
Class III	MSN4	LGE1
Class III	MOT3	LGE1
Class III	MBP1	YPL041C
Class III	SKN7	YPL041C
Class III	MSN2	YPL041C
Class III	MOT3	YPL041C
Class III	MSN2	PHO85
Class III	MSN4	PHO85
Class III	MBP1	PHO85
Class III	MSN4	YPR011C
Class III	MOT3	YPR011C
Class III	HAP5	MEP3
Class III	HAP3	MEP3
Class III	MSN2	MEP3
Class III	MSN4	MEP3
Class III	MOT3	MEP3
Class III	ADR1	MEP3
Class III	MOT3	YPR146C, YPR147C
Class III	RTG3	MET16
Class IV	MSN2	YBR285W
Class IV	MSN4	YBR285W
Class IV	ADR1	YBR285W
Class IV	UME6	ADR1
Class IV	STP1	ADR1
Class IV	MIG1	ADR1
Class IV	MSN4	ADR1

Class IV	ADR1	ADR1
Class IV	MSN2	ADR1
Class IV	ADR1	CRF1
Class IV	MSN2	CRF1
Class IV	MSN4	CRF1
Class IV	STP1	CRF1
Class IV	MSN4	PDR15, YDR406W-A
Class IV	IME1	PDR15, YDR406W-A
Class IV	MSN2	PDR15, YDR406W-A
Class IV	MIG1	YEF1
Class IV	STP1	YEF1
Class IV	MSN2	YEF1
Class IV	MSN4	YEF1
Class IV	STP1	TIR1
Class IV	ADR1	TIR1
Class IV	MSN2	TIR1
Class IV	MSN4	TIR1
Class IV	SPT15	TIR1
Class IV	ADR1	FAA2
Class IV	ADR1	YAT2
Class IV	MIG1	YAT2
Class IV	SPT15	YAT2
Class IV	STP1	SHC1
Class IV	MSN2	SHC1
Class IV	MSN4	SHC1
Class IV	ADR1	SHC1
Class IV	MSN2	HSP12
Class IV	MSN4	HSP12
Class IV	ADR1	HSP12
Class IV	SPT15	HSP12
Class IV	UME6	RMR1
Class IV	IME1	RMR1
Class IV	MSN4	RMR1
Class IV	MSN2	RCK1
Class IV	MSN4	RCK1
Class IV	ADR1	RCK1
Class IV	SPT15	RCK1
Class IV	ADR1	YGL081W
Class IV	MIG1	YGR110W
Class IV	ADR1	FMP43
Class IV	MSN2	FMP43
Class IV	MSN4	FMP43
Class IV	MIG1	FMP43
Class IV	STP1	FMP43
Class IV	MSN4	SOL4
Class IV	ADR1	SOL4

Class IV	MSN2	SOL4
Class IV	SPT15	YHR140W
Class IV	MIG1	SUC2
Class IV	SPT15	SUC2
Class IV	MIG1	JEN1
Class IV	ADR1	JEN1
Class IV	STP1	JEN1
Class IV	UME6	JEN1
Class IV	MSN2	JEN1
Class IV	MSN4	JEN1
Class IV	SPT15	PUT1
Class IV	ADR1	YLR356W
Class IV	MSN2	YMR084W, YMR085W
Class IV	MSN4	YMR084W, YMR085W
Class IV	MIG1	YMR084W, YMR085W
Class IV	ADR1	SIP18
Class IV	MSN2	SIP18
Class IV	MSN4	SIP18
Class IV	STP1	SIP18
Class IV	ADR1	YMR206W
Class IV	MSN2	YMR206W
Class IV	MSN4	YMR206W
Class IV	MIG1	YMR206W
Class IV	MSN2	YTP1
Class IV	MSN4	YTP1
Class IV	ADR1	YTP1
Class IV	ADR1	YPT53
Class IV	MSN2	YPT53
Class IV	MSN4	YPT53
Class IV	STP1	YNL092W
Class IV	MSN2	YNL092W
Class IV	MSN4	YNL092W
Class IV	ADR1	NCE103
Class IV	MSN2	NCE103
Class IV	MSN4	NCE103
Class IV	ADR1	YOL083W
Class IV	MSN2	YOL083W
Class IV	MSN4	YOL083W
Class IV	UME6	YOR019W
Class IV	IME1	BAG7
Class IV	UME6	BAG7
Class IV	STP1	BAG7
Class IV	ADR1	BAG7
Class IV	MSN2	BAG7
Class IV	MSN4	BAG7
Class IV	ADR1	DCS2

Class IV	MSN2	DCS2
Class IV	UME6	YOR338W
Class IV	STP1	YOR338W
Class IV	ADR1	YOR338W
Class IV	MSN2	YOR352W
Class IV	MSN4	YOR352W
Class IV	UME6	YOR352W
Class IV	STP1	YOR352W
Class IV	IME1	YOR352W
Class IV	MIG1	YOR352W
Class IV	ADR1	ALD4
Class IV	MSN2	ALD4
Class IV	MSN4	ALD4
Class IV	MIG1	ALD4
Class IV	STP1	ALD4
Class IV	IME1	ALD4
Class IV	UME6	ALD4
Class IV	UME6	FIT2
Class IV	STP1	FIT2
Class IV	MIG1	FIT2
Class IV	IME1	FIT2
Class IV	ADR1	FIT2
Class IV	MSN4	FIT2
Class IV	SPT15	FIT2
Class IV	ADR1	PXA1
Class IV	MSN2	PXA1
Class IV	MSN4	PXA1
Class IV	MIG1	PXA1
Class IV	IME1	PXA1
Class IV	UME6	PXA1
Class IV	MIG1	CIT3
Class IV	STP1	CIT3
Class IV	ADR1	CIT3
Class IV	SPT15	PDH1
Class IV	MSN4	ATH1
Class IV	MSN2	ATH1
Class IV	STP1	ATH1
Class IV	MIG1	ATH1
Class IV	ADR1	ATH1
Class IV	ADR1	GDB1
Class IV	MIG1	GDB1
Class IV	STP1	GDB1
Class IV	MSN2	GDB1
Class IV	MSN4	GDB1
Class IV	SPT15	PRX1
Class IV	MSN2	PRX1

Class IV	MSN4	PRX1
Class IV	MIG1	PRX1
Class IV	UME6	PRX1
Class IV	STP1	PRX1
Class IV	ADR1	NTH2
Class IV	STP1	NTH2
Class IV	SPT15	ADY2
Class IV	ADR1	ADY2
Class IV	UME6	ADY2
Class IV	STP1	ADY2
Class IV	MSN2	ADY2
Class IV	MSN4	ADY2
Class IV	IME1	ADY2
Class IV	MIG1	ADY2
Class IV	ADR1	FMP45
Class IV	MSN2	UGX2
Class IV	MSN4	UGX2
Class IV	MIG1	UGX2
Class IV	ADR1	UGX2
Class IV	MSN4	MRK1
Class IV	MIG1	MRK1
Class IV	MSN2	MRK1
Class IV	ADR1	MRK1
Class IV	MIG1	YDR018C
Class IV	ADR1	YDR018C
Class IV	MSN4	YDR018C
Class IV	MSN2	YDR018C
Class IV	ADR1	FMP16
Class IV	SPT15	FMP16
Class IV	MSN4	FMP16
Class IV	MSN2	FMP16
Class IV	ADR1	PHM6
Class IV	MSN4	YDR461C-A
Class IV	STP1	YDR461C-A
Class IV	IME1	YDR461C-A
Class IV	UME6	YFR017C
Class IV	ADR1	YFR017C
Class IV	MSN2	YFR017C
Class IV	MSN4	YFR017C
Class IV	ADR1	YGR053C
Class IV	MSN2	YGR053C
Class IV	MSN4	YGR053C
Class IV	UME6	YGR053C
Class IV	MSN4	YGR201C
Class IV	ADR1	GUT2
Class IV	MSN2	GUT2

Class IV	MSN4	GUT2
Class IV	IME1	YIL057C
Class IV	ADR1	YIL057C
Class IV	MIG1	YIL057C
Class IV	MSN2	YIL057C
Class IV	MSN4	YIL057C
Class IV	STP1	YIL057C
Class IV	UME6	YIL057C
Class IV	UME6	GSM1
Class IV	ADR1	IME1
Class IV	MIG1	IME1
Class IV	MSN2	IME1
Class IV	MSN4	IME1
Class IV	STP1	YKL133C
Class IV	MSN2	TFS1
Class IV	MSN4	TFS1
Class IV	SPT15	TFS1
Class IV	ADR1	ECI1
Class IV	STP1	ECI1
Class IV	ADR1	QNQ1
Class IV	MSN2	QNQ1
Class IV	MSN4	QNQ1
Class IV	STP1	QNQ1
Class IV	MSN2	CAT8
Class IV	MSN4	CAT8
Class IV	ADR1	CAT8
Class IV	MIG1	CAT8
Class IV	STP1	CAT8
Class IV	IME1	CAT8
Class IV	UME6	CAT8
Class IV	SPT15	ATO2
Class IV	ADR1	ATO2
Class IV	MIG1	ATO2
Class IV	STP1	ATO2
Class IV	MSN2	ATO2
Class IV	MSN4	ATO2
Class IV	SPT15	HPF1
Class IV	MSN2	MDH2
Class IV	MSN4	MDH2
Class IV	ADR1	MDH2
Class IV	STP1	MDH2
Class IV	UME6	MDH2
Class IV	IME1	MDH2
Class IV	MSN2	IZH4
Class IV	MSN4	IZH4
Class IV	ADR1	IZH4

Class IV	MSN4	GRE1
Class IV	MSN2	GRE1
Class IV	ADR1	GRE1
Class IV	MSN2	UIP4
Class IV	MSN4	UIP4
Class IV	ADR1	UIP4
Class IV	UME6	OYE3
Class IV	STP1	OYE3
Class IV	MSN4	ODC1
Class IV	STP1	ODC1
Class IV	ADR1	ODC1
Class IV	UME6	ODC1
Class IV	ADR1	YPR015C
